# Bicyclic δ‑Thiolactone Glycomimetics: Stereoselective Synthesis and Discovery of Stereocontrolled Antiphage Activity

**DOI:** 10.1021/acs.joc.6c00212

**Published:** 2026-04-02

**Authors:** Karol Postrożny, Bartosz Kamiński, Aleksandra F. Koper, Roman Luboradzki, Zahra Badri, Jan Paczesny, Mykhaylo A. Potopnyk

**Affiliations:** † Institute of Organic Chemistry, 49559 Polish Academy of Sciences, Kasprzaka 44/52, Warsaw 01-224, Poland; ‡ Institute of Physical Chemistry, Polish Academy of Sciences, Kasprzaka 44/52, Warsaw 01-224, Poland; § Department of Organic Chemistry, Faculty of Chemistry, Ivan Franko National University of Lviv, Kyryla and Mefodiya 6, Lviv 79005, Ukraine

## Abstract

A stereocontrolled synthesis of bicyclic thiolactones from methyl α-mannopyranoside and methyl α-galactopyranoside was achieved via iodination, selective protection, Bernet-Vasella fragmentation, and Knoevenagel-*hetero*-Diels–Alder domino reaction under polar, anhydrous, and inert conditions. d-mannoside derivatives cyclized with complete diastereoselectivity, whereas d-galactoside analogues showed substituent-dependent stereochemical outcomes. Structural assignments were confirmed by two-dimensional NMR spectroscopy and X-ray analyses. Spontaneous tautomerization of thioamides generated C=N bonds and new stereocenters, providing a predictable route to carbohydrate-derived thiolactones. Among the synthesized compounds, thiolactone **29** selectively inactivated bacteriophage T4 (>2-log reduction in PFU) without affecting *Escherichia coli* viability. These results demonstrate that stereochemical control dictates antiphage activity, establishing a framework for designing selective, non-toxic virucidal agents.

## Graphical abstract

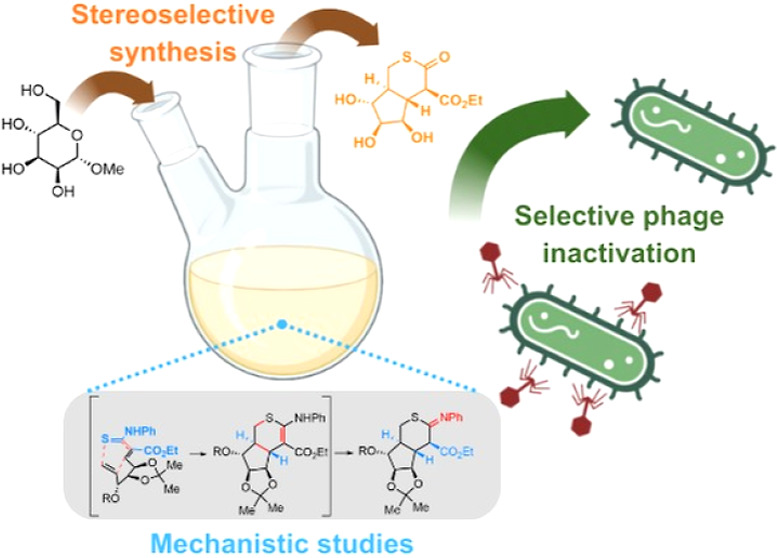

## Introduction

Carbohydrate–protein interactions underpin numerous biological processes, including immune recognition, microbial adhesion, viral infection, and cell signaling.^1,2^ However, the intrinsic structural complexity, conformational flexibility, and metabolic instability of native carbohydrates often hinder their direct use in mechanistic studies or therapeutic applications.^3,4^ These challenges have driven the development of glycomimetics—synthetic analogues that reproduce the key spatial and electronic features of carbohydrate epitopes while offering enhanced chemical stability and tunable physicochemical properties.^5–7^ By constraining the conformational landscape of sugar-like motifs, glycomimetics facilitate systematic investigation of recognition mechanisms and the rational design of selective carbohydrate-binding inhibitors.^8,9^ To improve the biological properties of carbohydrate mimics, various structural modifications have been introduced, including endocyclic oxygen replacement,^10–12^ glycosidic bond substitution,^13–15^ incorporation of lipophilic fragments,^16–18^ and replacement of remaining hydroxyl groups (Figure 1).^19,20^ Among these strategies, the use of bicyclic scaffolds offers an especially powerful approach to glycomimetic design, enabling precise control over stereochemistry and molecular rigidity while preserving essential hydroxyl or heteroatom arrays.^21–23^ Within this context, thiolactone frameworks are particularly attractive due to the distinctive stereoelectronic influence of sulfur substitution.^24^ Replacing oxygen with sulfur modulates ring strain, hydrogen-bonding capacity, and lipophilicity, often leading to improved stability and altered biological activity.^25^ Organic molecules bearing thiolactone functional groups have been mainly shown as a quorum sensing (QS) modulators.^26,27^ Affecting QS process controls not only bacteria group behavior,^28^ but is also associated with regulating virulence of bacteriophages, which uses environmental triggers to spread in bacterial cultures.^29^ Although carbohydrates participate in cell communication,^1^ their thiolactone derivatives have only been employed as intermediates for the synthesis of compounds of medicinal importance.^30,31^ In the present work, we evaluated the biological activity of newly synthesized thiolactone-based glycomimetics against both bacteria and bacteriophages (phages). Phages are ubiquitous across diverse environments and are responsible for eliminating up to 40% of bacterial populations daily,^32^ posing a significant challenge to bacteria-driven biotechnological processes. Industrial phage contamination was first reported in the dairy industry in 1935,^33^ and despite substantial advances, it continues to cause severe losses by reducing product quality and yield in bacteria-based biotechnological processes.^34^ Numerous antimicrobial agents, including Virkon (10,000 ppm potassium peroxymonosulfate), sodium hypochlorite (2500 ppm chlorine), and ethanol (75%), are commonly employed as disinfectants.^35,36^ However, a selective disinfectant capable of inactivating bacteriophages without harming beneficial bacteria remains elusive. Certain materials, such as gold nanoparticles, have demonstrated promising selectivity,^37^ but their high cost restricts practical application, underscoring the need for more affordable and efficient alternatives. Our synthesized thiolactone glycomimetics exhibit stereochemistry-dependent biological activity, selectively inactivating the T4 bacteriophage while preserving the viability of the host *Escherichia coli* strains (BL21 and C3000). *E. coli* BL21 is the most widely used bacterium. It is used as a host for recombinant protein production in pharmaceutical and industrial biotechnology. The strain exhibits superior biomass yield, reduced acetate formation, and higher protein expression capacity compared to other *E. coli* hosts, all of which are important factors in the biotechnology industry. *E. coli* C3000 serves as a laboratory reference strain and bacteriophage host for molecular biology applications. Both strains represent typical beneficial bacterial hosts used in research and industry.

**Figure 1:**
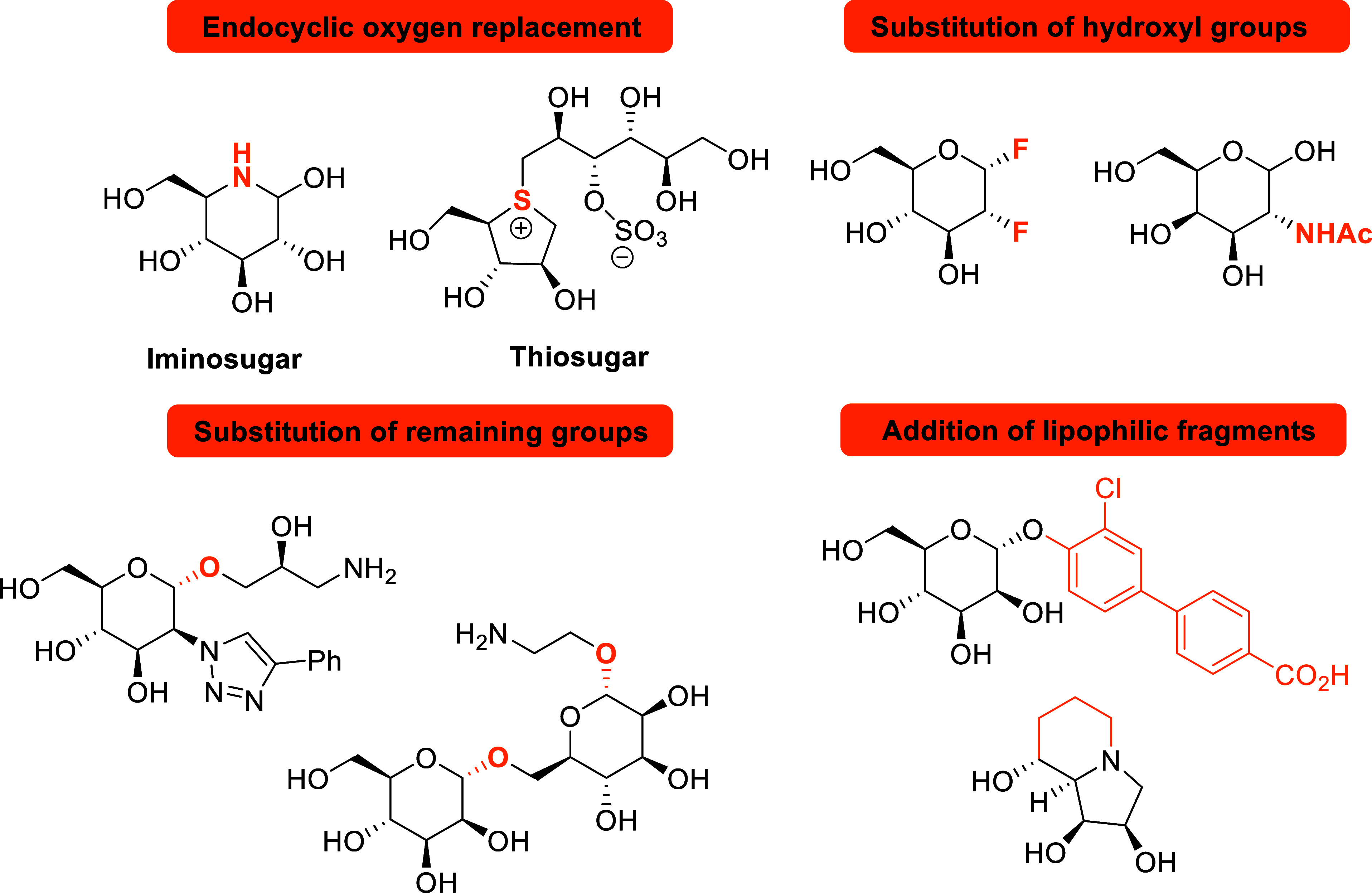
Examples of common glycomimetics modifications.

From a synthetic point of view, the complex architecture of glycomimetics often necessitates multistep synthetic routes, which can be streamlined through domino reactions. Tandem processes offer an efficient strategy for synthesizing complex molecules with enhanced overall yields and purity, while simultaneously reducing production time and costs.^38^ Among the various domino transformations employed in carbohydrate chemistry,^39–41^ the Knoevenagel-*hetero*-Diels–Alder (KHDA) reaction has emerged as a particularly efficient method, not only for the synthesis of sugar derivatives^42,43^ but also for their conjugation with nonsugar components to form polycyclic systems.^44–46^

Herein, we report the design, synthesis, theoretical investigation, and biological evaluation of a novel bicyclic thiolactone glycomimetic scaffold. The synthetic route features a domino KHDA reaction as the key step, enabling novel, efficient and diastereoselective synthesis of thiolactone derivatives from readily available methyl α-manno- and α-galactopyranosides. The resulting compounds were fully characterized, and their stereochemical configurations were confirmed by NMR spectroscopy and X-ray diffraction analyses. Finally, the biological activity of the synthesized thiolactones was evaluated against *E. coli* strains and their specific bacteriophages (T4 and MS2), revealing selective phage inactivation while maintaining bacterial viability. These findings highlight the potential of thiolactone-based glycomimetics as novel, selective antimicrobial agents, providing new insights into the structure–activity relationships governing their biological function.

## Results and Discussion

### Synthesis and Characterization

Our synthetic approach employed readily available methyl α-mannopyranoside (**1**) and methyl α-galactopyranoside (**2**) as starting materials. Iodination followed by isopropylidene protection afforded compounds **3** and **4**, with a higher yield observed for the d-mannoside derivative (Scheme 1). Subsequent transformations involved the protection of the remaining hydroxyl group using either a methyl (**5**, **7**) or *tert*-butoxycarbonyl (**6**, **8**) substituent. Bernet-Vasella fragmentation enabled the formation of aldehydes **9–12** (Scheme 1), which were immediately subjected to the KHDA reaction with thioamide **13** to afford the bicyclic, stable thioimidates **23–27**. The final step involved mild acidic cleavage, yielding the target thiolactones **28–32** (Scheme 2). Optimization of the domino reaction conditions (Table 1) using aldehyde **9** revealed that the process proceeds most efficiently in polar organic solvents at temperatures up to 70 °C. Moreover, the presence of a drying agent and an inert atmosphere proved essential for the reaction to occur (Entries 6 and 9). Further investigations demonstrated that the addition of bases (Entries 7 and 8), Lewis acids (Entry 12), or piperidine (Entry 13) accelerated the reaction and slightly improved the yield. However, we opted for neutral conditions and longer reaction times to preserve sensitive protecting groups.

**Scheme 1:**
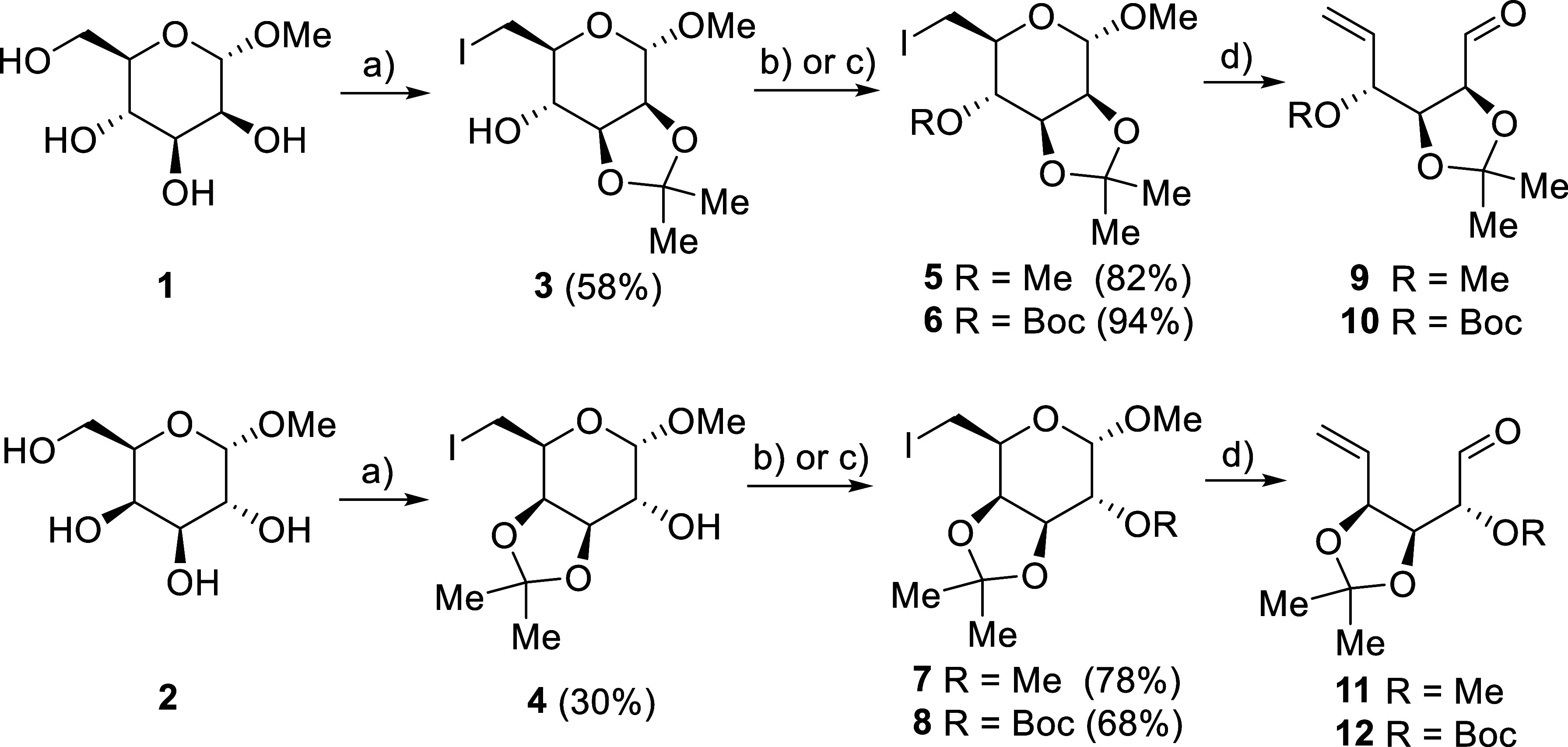
Synthesis of Sugar Enealdehydes^a^–^d^

a (1) I_2_, PPh_3_, Imidazole, THF, 60 °C, 2 h, (2) PTSA, 2,2-DMP/acetone, RT, 2 h, (3) Et_3_N, RT, 30 min.
b MeI, NaH, THF, 0 °C, 3-24 h.
c Boc_2_O, DMAP, DCM, RT, 30 min.
d Zn THF/H_2_O (9:1, v/v), ultrasound, 50 °C, 30 min.

**Scheme 2:**
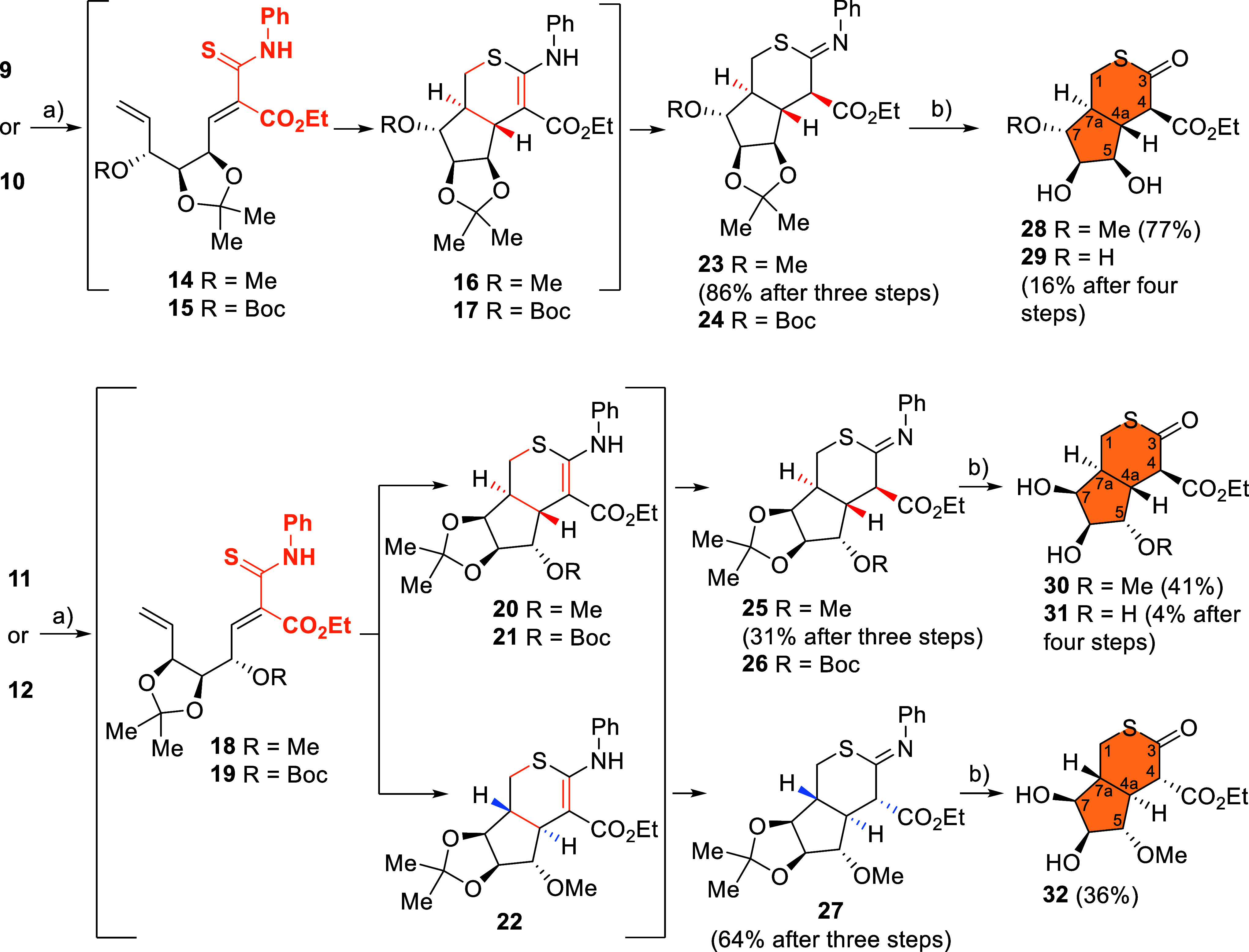
Synthesis of Bicyclic Thiolactone Glycomimetics via KHDA Reaction[Fn s2fn1]

a
**13**, MeCN, 70 °C, 2 h.
b Amberlyst 15, MeCN/H_2_O (9:1, v/v), 50 °C, 24 h.

**Table 1:**
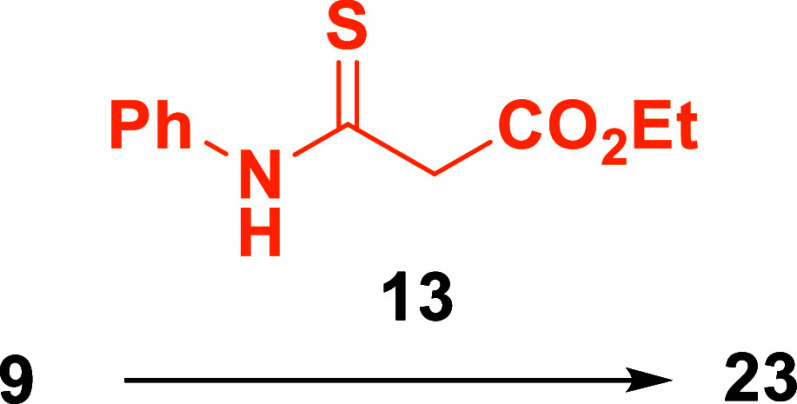
Optimization of KHDA Reaction Conditions with **9**

entry	equiv. 13	temp. [°C]	time	solvent	yield
1^a^	1.2	70	24 h	MeCN	40%
2^a^	1.2	25	24 h	MeCN	24%
3^a^	1.2	100	24 h	toluene	traces
4^a^	1.2	70	24 h	DMF	33%
5^a^	1.2	70	24 h	EtOH	35%
6^b^	1.2	25	15 min	neat	23%
7^a^ ^,^ ^c^	1.2	70	1 h	MeCN	37%
8^a^ ^,^ ^d^	1.2	70	1 h	MeCN	41%
9^e^	1.2	25	72 h	MeCN	traces
10^a^ ^,^ ^f^	1.2	100	1 h	MeCN	10%
11^a^ ^,^ ^d^ ^,^ ^f^	1.2	100	15 min	MeCN	35%
12^a^ ^,^ ^g^	1.2	70	24 h	MeCN	37%
13^a^ ^,^ ^h^	1.2	70	1 h	MeCN	33%
14^a^ ^,^ ^d^	2.5	70	1 h	MeCN	58%
15^a^	2.0	70	2 h	MeCN	86%

a 4A molecular sieves.

b Neat, grinding, Na_2_SO_4_.

c Cs_2_CO_3_ (2.0 equiv).

d Pyridine (2.0 equiv).

e 8 kbar.

f μW.

g ZnCl_2_ (50 mol %).

h Piperidine (10 mol %).

Cyclization of the d-mannoside derivatives **23** and **24** exhibited high diastereoselectivity, as only a single diastereomer was formed. Our extensive computational analysis suggests that the preferred product is favored over alternative diastereomers due to its thermodynamic stability. In the case of d-galactoside derivatives, high stereoselectivity was observed only for the Boc-protected compound **26**. In contrast, the methyl-protected aldehyde **11** afforded two separable diastereomers **25** and **27** in a 1:2 ratio. This interesting observation suggests a strong correlation between the size of the C-5 substituent and the stereochemical outcome of the reaction, rather than the influence of the carbohydrate ring itself. The domino reaction product undergoes spontaneous tautomerization, leading to the formation of a C=N double bond at C-3 and the creation of a new stereogenic center at C-4, whose configuration is determined by the spatial arrangement of the bridgehead protons. Similar tautomerization behavior has been previously reported in *thia*-Diels–Alder reactions involving noncarbohydrate compounds.^47^

The stereochemistry of the obtained compounds was determined by analysis of ^1^H–^1^H COSY and ^1^H–^1^H NOESY spectra of the final products. The bridgehead protons were assigned primarily through correlations between H-4a with the H-4 doublet, and H-7a with the diastereotopic H-1. The absence of through-space interactions between H-4a and H-7a indicated a *trans* relationship between these protons.

The known stereochemistry of the carbohydrate-derived cyclopentane ring enabled the determination of the absolute configurations at H-4, H-4a (via interaction with H-7), and H-7a (via interaction with H-4 and H-5) (Figure 2). The configurations of the d-mannoside-derived thiolactones **28** and **29** were additionally confirmed by X-ray diffraction analysis (Figure 3).

**Figure 2:**
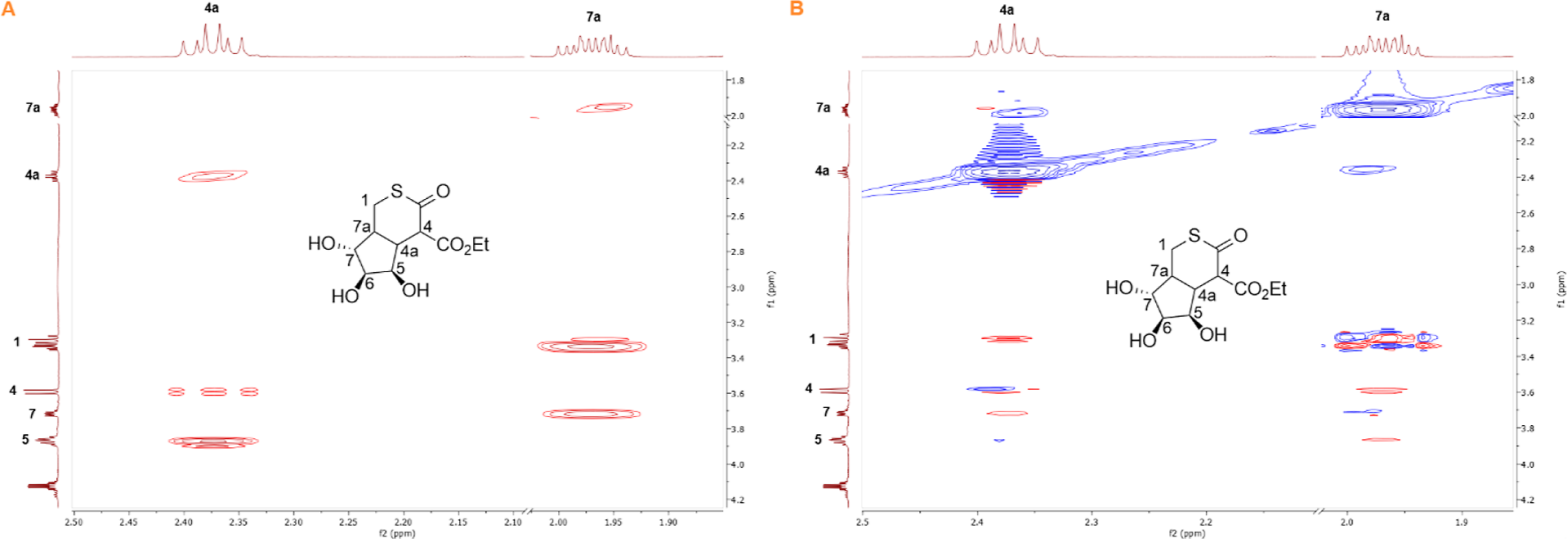
Assignment of configuration by ^1^H–^1^H COSY (A) and ^1^H–^1^H NOESY (B) nuclear magnetic resonance spectra analysis of **29**.

**Figure 3:**
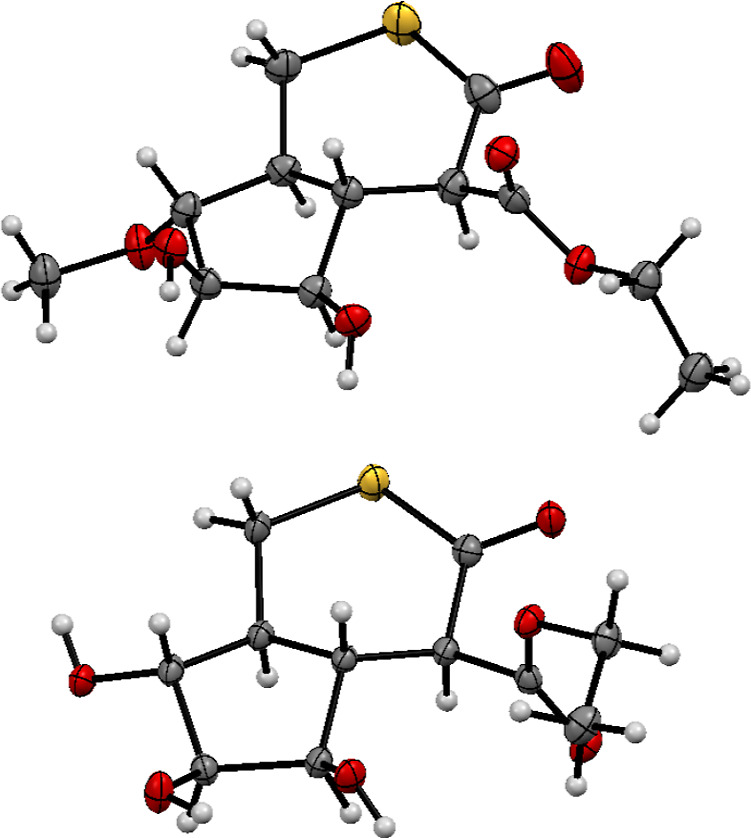
Crystal structures of compounds **28** (upper) and **29** (bottom).

### Theoretical Calculations

To rationalize the experimentally observed diastereoselectivity of the KHDA domino reaction and to gain insight into its kinetic and thermodynamic aspects, quantum chemical calculations were performed. Initial conformational sampling of the final domino reaction products derived from methyl α-manno- and α-galactopyranosides (**23/23a** and **25/27**, respectively, shown in Figure S3 in the Supporting Information) was carried out using the CREST (Conformer–Rotamer Ensemble Sampling Tool) program. Conformers within 3 kcal·mol^–1^ of the lowest-energy structure obtained in each run were selected for further refinement at the M06-2X/def2-TZVP level of theory employing the SMD solvation model (acetonitrile).^48,49^

For the mannoside derivatives, the lowest-energy conformer of **23** was calculated to be 8 kcal·mol^–1^ more stable than its diastereomer **23a**, consistent with the exclusive formation of **23** observed experimentally (see Table 2). This substantial thermodynamic difference supports the *exo*-selective cyclization pathway and the high stereoselectivity of the reaction.

**Table 2:** Computed Activation Barriers (Δ*G*‡) for the Diels–Alder Cycloaddition and Relative Free Energies (Δ*G*, Kcal mol^–1^) of the Corresponding Final Tautomerized Products **23**/**23a**, **25**/**25a**, and **27**/**27a** at the M06-2X/def2-TZVP Level of Theory with the SMD (MeCN) Solvation Model

system	path	Δ*G*‡ (TS-precomplex)	Δ*G*(product-unfavored product)
mannoside	23	19.7	–8.0
mannoside	23a	20.2	0.0
galactoside	25	21.2	–3.0
galactoside	27	22.6	–2.3
galactoside	25a	21.2	–0.4
galactoside	27a	22.6	0.0

In the case of the galactoside analogues, four possible isomers (**25**, **27**, **25a**, and **27a**, Figure S3 in Supporting Information) arising from different orientations in the cycloaddition step and subsequent tautomerization were examined. Among these, **25** and **27** were found to be the most stable, lying within 0.7 kcal·mol^–1^ of each other and approximately 3 kcal·mol^–1^ lower in energy than the remaining isomers. These small energy differences suggest that both **25** and **27** can coexist under equilibrium conditions, in agreement with the experimental isolation of two diastereomeric products.

To further assess the kinetic control, transition states, **14-TS**, **14a-TS**, **18-TS**, and **18a-TS** corresponding to the formation of the favored and unfavored cycloadducts were located. The barriers for the Diels–Alder cyclization were computed relative to their respective precomplexes, **14**, **14a**, **18**, and **18a**, shown in Figure S4 in the Supporting Information. The computed activation barriers for the Diels–Alder cycloaddition leading to the corresponding bicyclic cycloadducts (**16**/**16a** and **20**/**22**) were 19.7 and 20.2 kcal mol^–1^ for the pathways leading to **23** and **23a**, and 21.2 and 22.6 kcal mol^–1^ for the pathways leading to **25** and **27**, respectively. Although relatively high, these barriers are consistent with the experimental reaction temperature (70 °C), at which sufficient thermal energy is available to overcome them within the observed reaction times.

Taken together, these results suggest a dominant thermodynamic contribution to the observed selectivity, leading to the exclusive formation of the more stable **23** in the mannoside series and a mixture of **25** and **27** in the galactoside series.

To further rationalize the effect of the C-5 substituent size on the stereochemical outcome observed for the d-galactoside derivatives, additional thermodynamic and kinetic analyses were performed.

Computational analysis shows that **26** is thermodynamically favored over its diastereomer **26a** by 1.7 kcal mol^–1^, suggesting that the deprotection step proceeds from a single predominant precursor, resulting in the formation of one stereoisomer of the final unprotected product. Kinetic effects further reinforce this selectivity: while the difference in activation barriers for the formation of **25** and **27** (R = Me) is small (Δ*G*‡ ≈ 0.4 kcal mol^–1^), accounting for the lack of stereoselectivity, introduction of the bulkier Boc group increases this difference to approximately 3 kcal mol^–1^, rendering the formation of **21** kinetically favored as well. The combined kinetic and thermodynamic bias induced by the Boc substituent therefore explains the experimentally observed stereoselective formation of a single diastereomer in this series (see Table S3 and Figure S4 in the Supporting Information).

The computational data thus corroborate the stereochemical assignments derived from NMR and X-ray analyses and elucidate the energetic origins of the observed diastereoselectivity.

### Biological Activity

To check the effect of the synthesized compounds, samples containing 800 μL of buffer (PBS for *E. coli* and TM for T4), 100 μL of the compounds’ stock in 20% DMSO, and 100 μL of bacterial suspension (10^5^ CFU mL^–1^) or phage suspension (10^7^ PFU mL^–1^) were prepared. Final concentration of thiolactons: 1.0 mg mL^–1^; **28**, **30**, **32**: 3.45 mM; **29**, **31**: 3.64 mM. The control sample contained only buffer and a bacterial/phage suspension, as well as a control containing 100 μL of a 20% aqueous DMSO solution (without compounds). DMSO was added to increase the solubility of the studied bicyclic δ-thiolactone glycomimetics. Our controls and literature data confirmed that DMSO at a final concentration of 2% (v/v) did not affect the survival of *E. coli*, T4 (Figure 4), and MS2 (Figure S5, Supporting Information).^50^ After 24 h of incubation, colonies and plaques were counted (Figure 4).

**Figure 4:**
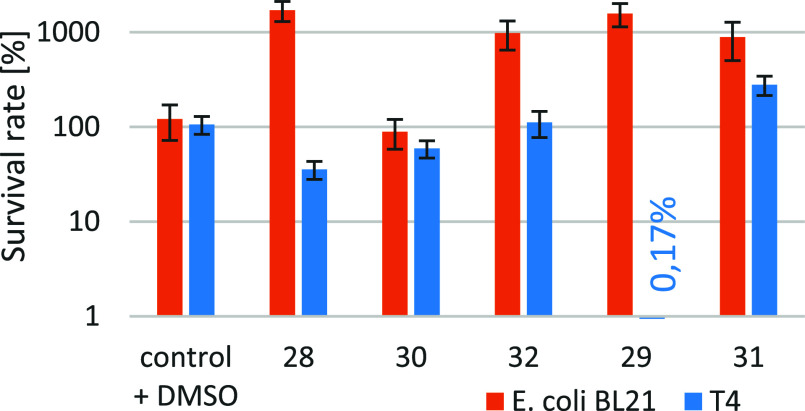
Effect of δ-thiolactone glycomimetics on phages and host bacteria *E. coli* BL21 (orange) and T4 bacteriophage (blue) after 24 h of incubation at room temperature. No negative effect on bacteria was observed; for most compounds, an increase in bacterial counts was noted. For the T4 phage, partial inactivation was observed for compounds **30** and **28** (41% and 64% inactivation, respectively), while the highest level of inactivation was achieved with compound **29** (2.7-log reduction).

No adverse effects of the studied thiolactones were observed against the bacteria. We consistently recorded increased bacterial counts (BL21 and C3000 strains), likely due to the addition of an extra portion of food source (i.e., the studied compounds) to the medium. Compounds **28**, **30**, **32**, and **31** had no or a negligible effect on the T4 and MS2 phages. The most interesting result was the substantial inactivation of T4, but not MS2, caused by compound **29**. As it was not harmful for the host, it could act as a potential antiphagent (antibacteriophage agent). This type of selectivity is in high demand for the biotech industry, where phage contamination is a significant threat to bacteria-based production.^34^

Although the exact mechanism of phage inactivation is not established, we present a possible hypothesis. Many phages encode virion-associated lysins (VALs), i.e., enzymes that are part of the virion structure (tail tip, baseplate, etc*.*) and act externally to degrade bacterial cell–wall or polysaccharide barriers.^51^ In bacteriophage T4, a three-domain baseplate protein with a central lysozyme domain facilitates peptidoglycan degradation during infection. Following post-translational cleavage, the N-terminal fragment (gp5) assembles into a trimeric puncturing device that penetrates the outer membrane and locally digests the peptidoglycan layer, thereby facilitating DNA injection into the bacterial cytoplasm.^52^

MS2 also expresses lysins, but acting from within, facilitating the release of progeny virions at the final stage of infection.^53^ Because lysozyme in T4 acts from the outside of the bacterial host, it is accessible to small-molecule inhibitors or blocking agents, thereby offering a plausible target for antiphage compounds. Such interactions with **29** could affect the enzyme’s activity or stability, leading to phage inactivation. Further biochemical and structural studies are needed to confirm this hypothesis.

## Conclusions

In this work, we developed a stereocontrolled synthesis of bicyclic thiolactones starting from commercially available methyl α-mannopyranoside and methyl α-galactopyranoside. The key step involves a Knoevenagel-*hetero*-Diels–Alder domino reaction. d-Mannoside derivatives cyclized with complete diastereoselectivity, whereas d-galactoside analogues displayed substituent-dependent stereochemical outcomes, confirmed by ^1^H–^1^H COSY, NOESY, and X-ray diffraction analyses. Spontaneous tautomerization to thioimidates generated C=N bonds and new stereogenic centers, providing a robust and predictable route to carbohydrate-derived thiolactones. Among the synthesized compounds, thiolactone **29** selectively inactivated bacteriophage T4 (>2-log reduction in PFU) without affecting the viability of host bacteria (*E. coli*). Other derivatives exhibited no significant antimicrobial activity or, in some cases, enhanced bacterial growth. These results demonstrate that the unique conformation and stereochemistry of compound **29** confer virucidal properties while remaining non-toxic, providing the first evidence of stereochemical influence on antiphage activity and guiding the design of selective, non-toxic antiphage agents.

## Experimental Section

### General Information

Commercially available reagents were used without further purification unless otherwise stated. Molecular sieves were dried before the reaction. All reagents and chemicals were purchased from commercial sources (TCI, Thermo Scientific, Aldrich, or Alfa Aesar). Analytical thin-layer chromatography (TLC) was conducted using TLC plates (Silica gel 60 F_254_, Merck, or Al_2_O_3_ ALUGRAM ALOX N/UV_254_). Visualization of TLC was achieved by UV light or cerium–molybdenum stain. Flash column chromatography was performed on silica gel 230–400 mesh or neutral Al_2_O_3_ with freshly distilled solvents. Flash chromatography was performed using silica gel 60 (230–400 mesh, Merck) with a Knauer Smartline system equipped with a Buchi fraction collector or a Grace Reverelis flash chromatography system featuring ELSD detection and Grace Resolv or Reveleris cartridges.

Nuclear magnetic resonance (NMR) spectra were recorded on a Varian AM-600 and Varian AM-500 (2D NMR) or Varian Mercury 400 MHz (1D NMR) spectrometers at room temperature using CDCl_3_ or Acetone-*d*_6_ as a solvent and referenced externally to SiMe_4_. High-resolution mass spectra (HRMS) were collected on a Synapt G2-S HDMS (Waters Inc.) mass spectrometer equipped with an atmospheric-pressure chemical ionization (APCI) ion source or electrospray ionization (ESI) ion source and quadrupole-time-of-flight (q-TOF) mass analyzer. The instrument was controlled, and the recorded data were processed using MassLynx V4.1 software package (Waters Inc.). Spectra were collected in ESI or APCI mode.

Optical rotation measurements were conducted for 10 mg mL^–1^ acetone solutions of synthesized materials in a 50 mm tube and recorded with a Jasco P-2000 polarimeter.

Single crystals of compounds **28** and **29** were grown by slow evaporation of their solution in acetone-*d*_6_/dichloromethane (1:3) mixture for structures **28** and **29** under ambient conditions. Single crystal X-ray diffraction measurements were carried out on an Agilent Supernova diffractometer, at 100 K with monochromated Cu Kα radiation (1.54184 Å). The data reduction was made by using *CrysAlisPRO* software.^54,55^ The structures were solved by direct methods and refined with the olex2.refine^56^ refinement package using Gauss–Newton minimization. All non-hydrogen atoms were refined as anisotropic, while hydrogen atoms were placed in calculated positions and refined in riding mode. Crystallographic data of compounds **28** and **29** have been deposited with the Cambridge Crystallographic Data Centre (CCDC) and can be obtained, free of charge, from CCDC via https://www.ccdc.cam.ac.uk/structures/.

### Synthesis

#### α-Methyl 6-Deoxy-6-iodo-2,3-isopropylidene Mannoside **(3)**

Methyl α-_D_-mannopyranoside **1** (10.0 g, 51.5 mmol, 1.0 equiv), imidazole (7.01 g, 103.0 mmol, 2.0 equiv), and triphenyl phosphine (20.3 g, 77.2 mmol, 1.5 equiv) were dissolved in anhydrous THF (150 mL) and heated to 60 °C in an oil bath. Iodine (19.7 g, 77.2 mmol, 1.5 equiv) in THF (50 mL) was added portion-wise, and the reaction was heated at reflux for 2 h. TLC showed full conversion of the starting material. The mixture was cooled to RT, and the solvent was removed under diminished pressure. The residue was redissolved in a mixture of acetone (150 mL) and 2,2-DMP (30 mL) and cooled to 0 °C. *p*-Toluenesulfonic acid (7.1 g, 41.2 mmol, 0.8 equiv) was added, and the mixture was stirred at room temperature for 2 h. Next, triethylamine (30 mL) was added and stirred at RT for 30 min, then the solvent was removed under diminished pressure. The crude residue was redissolved in DCM (25 mL), washed with NaHCO_3_ (20 mL), dried over Na_2_SO_4_, and the solvent was removed. Additionally, residue was washed with diethyl ether (3 × 50 mL) to remove triphenylphosphine oxide impurities. Flash column chromatography of the residue (SiO_2_, hexanes/EtOAc with gradient 0–20% of EtOAc) gave 9.94 g (58%) of the title compound as a white solid. [α]_D_^27^ = +42.2. ^1^H NMR (400 MHz, CDCl_3_) δ: 4.92 (s, 1H), 4.17–4.04 (m, 2H), 3.58 (dd, *J* = 10.6 Hz, *J* = 2.6 Hz, 1H), 3.53–3.43 (m, 5H), 3.31 (dd, *J* = 10.6 Hz, *J* = 7.1 Hz, 1H), 1.52 (s, 3H), 1.35 (s, 3H) ppm. ^13^C­{H} NMR (101 MHz, CDCl_3_) δ: 109.8, 98.5, 75.7, 73.2, 69.2, 55.6, 28.0, 26.1, 6.7 ppm. Data are consistent with previously characterized compound.^57^

#### α-Methyl 6-Deoxy-6-iodo-3,4-isopropylidene Galactoside **(4)**

Methyl α-_D_-galactopyranoside **2** (15.0 g, 77.2 mmol, 1.0 equiv), imidazole (10.5 g, 154.5 mmol, 2.0 equiv), and triphenyl phosphine (30.4 g, 115.9 mmol, 1.5 equiv) were dissolved in anhydrous THF (150 mL) and heated to 60 °C in an oil bath. Iodine (29.4 g, 115.9 mmol, 1.5 equiv) in THF (50 mL) was added portion-wise, and the reaction was heated at reflux for 2 h. The mixture was cooled to RT, and the solvent was removed under diminished pressure. The residue was redissolved in a mixture of acetone (150 mL) and 2,2-DMP (30 mL) and cooled to 0 °C. *p*-Toluenesulfonic acid (7.98 g, 46.3 mmol, 0.6 equiv) was added, and the mixture was stirred at room temperature for 2 h. Next, triethylamine (40 mL) was added and stirred at RT for 30 min, then the solvent was removed under diminished pressure. The crude residue was redissolved in DCM (25 mL), washed with NaHCO_3_ (20 mL), dried over Na_2_SO_4_, and the solvent was removed. Flash column chromatography of the residue (SiO_2_, hexanes/EtOAc with a gradient of 0–20% EtOAc) yielded 7.88 g (30%) of the title compound as a yellowish oil. [α]_D_^26^ = +118.8. ^1^H NMR (400 MHz, CDCl_3_) δ: 4.75 (d, *J* = 3.9 Hz, 1H), 4.31 (dd, *J* = 6.3 Hz, *J* = 2.0 Hz, 1H), 4.12 (ddd, *J* = 7.9 Hz, *J* = 5.7 Hz, *J* = 2.0 Hz, 1H), 3.85 (dd, *J* = 5.5 Hz, *J* = 3.9 Hz, 1H), 3.51 (s, 3H), 3.38–3.24 (m, 2H), 1.47 (s, 3H), 1.33 (s, 3H) ppm. ^13^C­{H} NMR (101 MHz, CDCl_3_) δ: 110.1, 98.5, 76.1, 73.9, 69.8, 68.9, 55.9, 27.7, 26.0, 3.1 ppm. Data is consistent with previously characterized compound.^58^

#### α-Methyl 6-Deoxy-6-iodo-4-methoxy-2,3-isopropylidene Mannoside **(5)**

To a solution of **3** (5.0 g, 14.5 mmol, 1.0 equiv) in distilled THF (5.0 mL) at 0 °C under an inert gas atmosphere, sodium hydride (0.87 g, 36.3 mmol, 2.5 equiv) was added. Then, iodomethane (1.8 mL, 29.1 mmol, 2.0 equiv) was slowly added. The reaction mixture was left stirring at 0 °C to RT for 24 h. Then, methanol (3.0 mL) was slowly added to the mixture in order to neutralize the remaining sodium hydride. After that, water and diethyl ether were added, and the aqueous phase was washed with diethyl ether (3 × 10.0 mL). The combined organic fractions were dried over Na_2_SO_4_, filtered, and then concentrated. The crude residue was purified by flash column chromatography (SiO2, EtOAc/hexanes with a gradient of 0–25% EtOAc) to yield 4.25 g (82%) of the title compound as a slightly yellow oil. [α]_D_^26^ = +40.3. ^1^H NMR (400 MHz, CDCl_3_) δ: 4.90 (s, 1H), 4.16 (dd, *J* = 6.8 Hz, *J* = 5.7 Hz, 1H), 4.09 (dd, *J* = 5.8 Hz, *J* = 0.8 Hz, 1H), 3.53 (s, 4H), 3.46 (s, 3H), 3.38 (dddd, *J* = 9.5 Hz, *J* = 7.4 Hz, *J* = 2.3 Hz, *J* = 0.6 Hz, 1H), 3.29 (dd, *J* = 10.3 Hz, *J* = 7.3 Hz, 1H), 3.10 (dd, *J* = 9.5 Hz, *J* = 6.8 Hz, 1H), 1.53 (s, 3H), 1.34 (s, 3H) ppm. ^13^C­{H} NMR (101 MHz, CDCl_3_) δ: 109.8, 98.8, 82.1, 78.6, 76.3, 68.4, 59.7, 55.9, 28.3, 26.6, 7.5 ppm. HRMS (ESI) *m*/*z*: [M + Na]^+^ Calcd for C_11_H_19_IO_5_Na 381.0175; Found, 381.0176.

#### α-Methyl 6-Deoxy-6-iodo-2-methoxy-3,4-isopropylidene Galactoside **(7)**

To a solution of compound **4** (1.92 g, 5.58 mmol, 1.0 equiv) in distilled THF (5.0 mL) at 0 °C under an inert gas atmosphere, sodium hydride (0.4 g, 16.7 mmol, 3.0 equiv) was added. Then, iodomethane (0.9 mL, 13.9 mmol, 2.5 equiv) was slowly added. The reaction mixture was left stirring at 0 °C for 3 h. Next, methanol (3.0 mL) was slowly added to the mixture in order to neutralize the remaining sodium hydride. Then, water and diethyl ether were added. The aqueous phase was extracted with diethyl ether (3 × 10 mL). The combined organic fractions were dried over Na_2_SO_4_, filtered, and then concentrated. Crude residue was purified by flash column chromatography (SiO_2_, EtOAc/hexanes with gradient 0–25% of EtOAc). 1.56 g (78%) of the title compound was obtained as a slightly orange oil. [α]_D_^26^ = +62.7. ^1^H NMR (400 MHz, CDCl_3_) δ: 4.75 (d, *J* = 3.6 Hz, 1H), 4.23 (dd, *J* = 5.6 Hz, *J* = 2.5 Hz, 1H), 4.18 (dd, *J* = 7.6 Hz, *J* = 5.5 Hz, 1H), 4.01 (ddd, *J* = 8.3 Hz, *J* = 5.8 Hz, *J* = 2.5 Hz, 1H), 3.44 (s, 3H), 3.38 (s, 3H), 3.34–3.24 (m, 3H), 1.45 (s, 3H), 1.27 (s, 3H) ppm. ^13^C­{H} NMR (101 MHz, CDCl_3_) δ: 109.2, 97.9, 78.9, 75.8, 73.8, 68.1, 58.8, 55.7, 28.2, 26.3, 2.5 ppm. HRMS (ESI) *m*/*z*: [M + Na]^+^ Calcd for C_11_H_19_IO_5_Na 381.0175; Found, 381.0179.

#### α-Methyl 6-Deoxy-6-iodo-4-(*tert*-butoxycarbonyl)-2,3-isopropylidene Mannoside **(6)**

A solution of iodide **3** (1.0 g, 2.91 mmol, 1.0 equiv) in DCM (5.0 mL) was added Boc_2_O (1.9 g, 8.72 mmol, 3.0 equiv) and DMAP (35 mg, 10 mol %) (color drastically changes from brown to bright yellow). The reaction was stirred at room temperature for 30 min, after which full conversion was observed. Next, water was added, and the aqueous phase was washed with DCM (3 × 5 mL), NaHCO_3_, and brine. The organic layers were dried over Na_2_SO_4_, filtered, and then concentrated. Purification by flash column chromatography (SiO_2_, EtOAc/hexanes with a gradient of 0–10% EtOAc) yielded 1.2 g (94%) of the desired product as a yellowish oil. [α]_D_^27^ = +21.7. ^1^H NMR (500 MHz, CDCl_3_) δ: 4.95 (s, 1H), 4.61 (dd, *J* = 10.1 Hz, *J* = 7.6 Hz, 1H), 4.21 (dd, *J* = 7.6 Hz, *J* = 5.4 Hz, 1H), 4.13 (d, *J* = 5.2 Hz, 1H), 3.69 (ddd, *J* = 9.7 Hz, *J* = 2.4 Hz, 1H), 3.51 (s, 3H), 3.32 (dd, *J* = 10.8 Hz, *J* = 2.4 Hz, 1H), 3.16 (dd, *J* = 10.8 Hz, *J* = 9.3 Hz, 1H), 1.56 (s, 3H), 1.49 (s, 9H), 1.34 (s, 3H) ppm. ^13^C­{H} NMR (126 MHz, CDCl_3_) δ: 152.8, 110.2, 98.5, 83.2, 77.2, 76.5, 76.1, 75.7, 68.4, 56.0, 27.9, 27.8, 26.5, 4.1 ppm. HRMS (ESI) *m*/*z*: [M + Na]^+^ Calcd for C_15_H_25_IO_7_Na 467.0543; Found, 467.0550.

#### α-Methyl 6-Deoxy-6-iodo-2-(*tert*-buthoxycarbonyl)-3,4-isopropylidene Galactoside **(8)**

A solution of iodide **4** (1.0 g, 2.91 mmol, 1.0 equiv) in DCM (5.0 mL) was added to Boc_2_O (1.9 g, 8.72 mmol, 3.0 equiv) and DMAP (35 mg, 10 mol %) (color drastically changes from brown to bright yellow). The reaction was stirred at room temperature for 30 min, after which full conversion was observed. Next, water was added, and the aqueous phase was washed with DCM (3 × 5 mL), NaHCO_3_, and brine. The organic layers were dried over Na_2_SO_4_, filtered, and then concentrated. Purification by flash column chromatography (SiO_2_, EtOAc/hexanes with a gradient of 0–10% EtOAc) yielded 880 mg (68%) of the desired product as a yellowish oil. [α]_D_^27^ = +125.3. ^1^H NMR (400 MHz, CDCl_3_) δ: 4.87 (d, *J* = 3.6 Hz, 1H), 4.69 (dd, *J* = 7.1 Hz, *J* = 3.6 Hz, 1H), 4.36–4.29 (m, 2H), 4.09 (ddd, *J* = 8.2 Hz, *J* = 6.2 Hz, *J* = 2.1 Hz, 1H), 3.42 (s, 3H), 3.40–3.33 (m, 2H), 1.51 (s, 3H), 1.47 (s, 9H), 1.33 (s, 3H) ppm. ^13^C­{H} NMR (101 MHz, CDCl_3_) δ: 153.0, 109.9, 97.6, 82.9, 74.0, 73.9, 73.6, 68.4, 55.9, 28.0, 27.8, 26.4, 2.3 ppm. HRMS (ESI) *m*/*z*: [M + Na]^+^ Calcd for C_15_H_25_IO_7_Na 467.0543; Found, 467.0544.

**Ethyl 3-(phenylamino)-3-thioxopropanoate (13)** was prepared using a modified literature procedure.^59^ A flask filled with an inert gas and equipped with a magnetic stirrer bar was charged with anhydrous EtOH (100 mL) and solid sodium (1.86 g, 80.7 mmol, 1.05 equiv). After sodium being fully dissolved, ethyl acetoacetate **S1** (9.7 mL, 76.8 mmol, 1.0 equiv) was added, and the mixture was allowed to stir for 15 min. Then, phenyl isothiocyanate **S2** (14.6 mL, 76.8 mmol, 1.0 equiv) was added dropwise over 20 min (color turned intense yellow), and the reaction was stirred for 24 h (the color slowly turned to dark orange). The reaction was diluted using EtOAc (50.0 mL) and acidified with 1 M HCl (aq) (50.0 mL). The phases were separated, and the combined organic phases were dried over Na_2_SO_4_, filtered, and concentrated in vacuo. Flash column chromatography of the crude product (SiO_2_, ethyl acetate/hexanes with gradient 0–25% of EtOAc) gave 14.7 g (86%) of the title product as an orange oil. ^1^H NMR (400 MHz, CDCl_3_) δ: 11.01 (s, 1H), 7.75 (d, *J* = 7.5 Hz, 2H), 7.40 (t, *J* = 7.9 Hz, 2H), 7.26 (t, *J* = 7.3 Hz, 1H), 4.26 (q, *J* = 7.2 Hz, 2H), 3.99 (s, 2H), 1.33 (t, *J* = 7.2 Hz, 3H) ppm. ^13^C­{H} NMR (101 MHz, CDCl_3_) δ: 192.3, 170.7, 138.7, 129.1, 127.1, 123.6, 62.2, 49.9, 14.2 ppm.

#### Ethyl (3a*R*,3b*R*,4*R*,7a*S*,8*R*,8a*S*,*Z*)-8-methoxy-2,2-dimethyl-5-(phenylimino)­octahydrothiopyrano­[4′,3′:3,4]­cyclopenta­[1,2-*d*]­[1,3]­dioxole-4-Carboxylate **(23)**

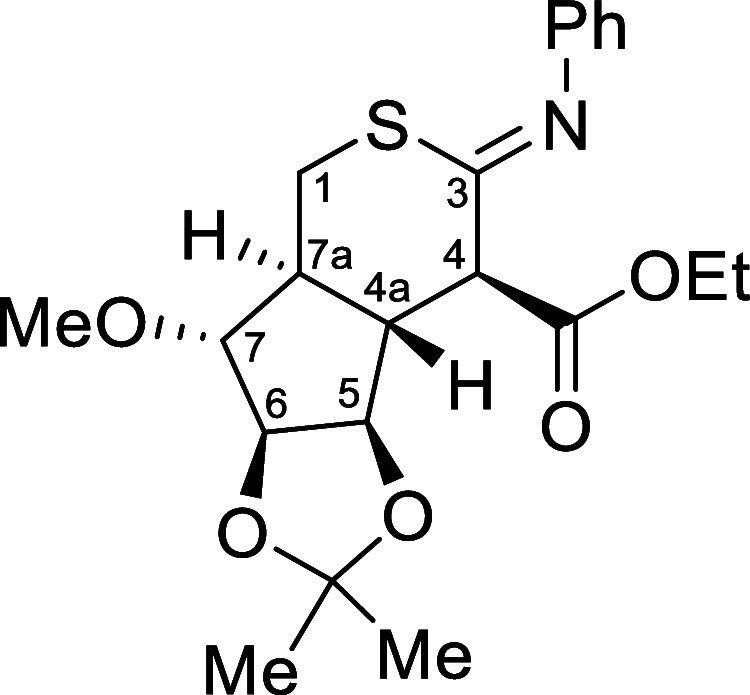



The iodomannopyranoside **5** (0.6 g, 1.68 mmol, 1.0 equiv) was dissolved in a mixture of THF (9.0 mL) and H_2_O (1.0 mL), and zinc (preactivated with 1:9 v/v solution of HCl/H_2_O, 1.10 g, 16.8 mmol, 10.0 equiv) was added. The flask was immersed in a sonication apparatus preheated to 55 °C. The solution was sonicated for 30 min, after which TLC revealed full conversion of the starting material (the product does not appear under UV). The suspension was passed through a plug of Celite using diethyl ether (20 mL). The combined filtrates were concentrated and dried under reduced pressure to give aldehyde **9** as a slightly yellow oil. To a mixture of freshly prepared aldehyde **9** in anhydrous acetonitrile (5.0 mL) and molecular sieves 4A under an inert gas atmosphere, compound **13** (0.75 g, 3.35 mmol, 2.0 equiv) was added. Reaction mixture was stirred at 70 °C in an oil bath for 2 h and left at RT for 24 h. Crude was absorbed on Celite and purified by flash column chromatography (SiO_2_, EtOAc/toluene with a gradient of 0–10% EtOAc) to yield 586 mg (86%, after three steps) as a yellow solid. [α]_D_^26^ = +45.6. ^1^H NMR (400 MHz, CDCl_3_) δ: 7.29 (t, *J* = 7.8 Hz, 2H, Ar), 7.08 (td, *J* = 7.4 Hz, *J* = 1.2 Hz, 1H, Ar), 6.77 (d, *J* = 8.0 Hz, 2H, Ar), 4.45 (dd, *J* = 7.7 Hz, *J* = 3.6 Hz, 1H, H-6), 4.38 (dd, *J* = 7.2 Hz, *J* = 7.2 Hz, 1H, H-5), 4.27 (q, *J* = 7.0 Hz, 2H, CH_2_CH_3_), 3.59 (d, *J* = 12.1 Hz, 1H, H-4), 3.46–3.40 (m, 4H, OCH_3_, H-7), 3.04 (dd, *J* = 11.8 Hz, *J* = 4.2 Hz, 1H, H-1′), 2.93 (dd, *J* = 11.7 Hz, *J* = 11.8 Hz, 1H, H-1), 2.33 (ddd, *J* = 12.7 Hz, *J* = 12.2 Hz, *J* = 6.9 Hz, 1H, H-4a), 2.11 (dddd, *J* = 13.3 Hz, *J* = 11.6 Hz, *J* = 9.6 Hz, *J* = 4.1 Hz, 1H, H-7a), 1.48 (s, 3H, CH_3_), 1.32–1.27 (m, 6H, CH_3_, CH_2_CH_3_) ppm. ^13^C­{H} NMR (101 MHz, CDCl_3_) δ: 169.7 (C-3), 160.2 (C=O), 150.0 (Ar), 128.8 (2C, Ar), 124.3 (Ar), 119.5 (2C, Ar), 114.1 (C^IV^), 89.2 (C-7), 84.0 (C-6), 82.6 (C-5), 61.2 (CH_2_CH_3_), 59.8 (C-4), 58.1 (OCH_3_), 48.0 (C-4a), 45.0 (C-7a), 31.9 (C-1), 27.2 (CH_3_), 25.2 (CH_3_), 14.2 (CH_2_ CH_3_) ppm. HRMS (ESI) *m*/*z*: [M + H]^+^ Calcd for C_21_H_28_NO_5_S 406.1688; Found, 406.1689.

#### Ethyl (3a*S*, 3b*R*, 7*S*, 7a*S*, 8*S*, 8a*R*, *Z*)-8-methoxy-2,2-dimethyl-6-(phenylimino)­octahydrothiopyrano­[3′,4’:3,4]­cyclopenta­[1,2-*d*]­[1,3]­dioxole-7-carboxylate (25) and Ethyl (3a*S*, 3b*S*, 7*R*, 7a*R*, 8*S*, 8a*R*, *Z*)-8-methoxy-2,2-dimethyl-6-(phenylimino)­octahydrothiopyrano­[3′,4’:3,4]­cyclopenta­[1,2-*d*]­[1,3]-dioxole-7-carboxylate **(27)**

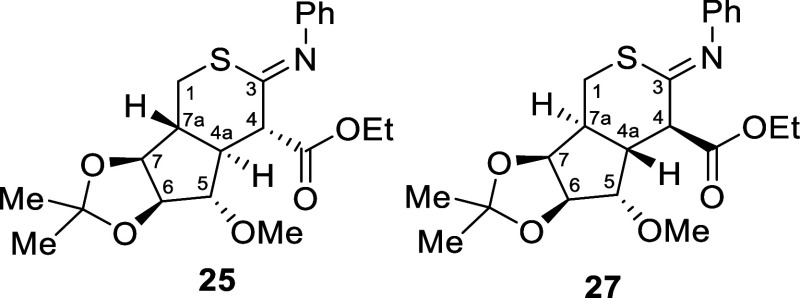



The iodogalactopyranoside **7** (1.1 g, 3.07 mmol, 1.0 equiv) was dissolved in a mixture of THF (9.0 mL) and H_2_O (1.0 mL), and zinc (preactivated with 1:9 v/v solution of HCl/H_2_O, 2.0 g, 30.7 mmol, 10.0 equiv) was added, and the flask was immersed in a sonication apparatus preheated to 50 °C. The solution was sonicated for 30 min, after which TLC revealed the full conversion of the starting material (the product does not appear under UV; the reaction mixture turns a bright gray color). The suspension was passed through a plug of Celite using diethyl ether (10 mL). The combined filtrates were concentrated and dried under reduced pressure to give aldehyde **11** as a yellow oil. To a mixture of freshly prepared aldehyde **11** in anhydrous acetonitrile (5.0 mL) and molecular sieves 4A under an inert gas atmosphere, compound **13** (1.37 g, 6.14 mmol, 2.0 equiv) was added. The reaction mixture was stirred at 70 °C in an oil bath for 2 h and at RT for 24 h. Crude product was absorbed on Celite and purified by flash column chromatography (SiO_2_, EtOAc/toluene with gradient 0–15% of EtOAc) to give separable diastereomers: 389 mg (31%, after three steps) of **25** and 798 mg (64%, after three steps) of **27** as yellow oils.

Analysis for **27**: [α]_D_^27^ = – 24.9. ^1^H NMR (600 MHz, CDCl_3_) δ: 7.29 (dd, *J* = 8.3 Hz, *J* = 7.4 Hz, 2H, Ar), 7.07 (tt, *J* = 7.6 Hz, *J* = 1.2 Hz, 1H, Ar), 6.77 (dd, *J* = 8.5 Hz, *J* = 1.2 Hz, 2H, Ar), 4.45 (dd, *J* = 7.7 Hz, *J* = 3.6 Hz, 1H, H-6), 4.31–4.24 (m, 2H, H-7, CH_2_CH_3_), 4.20–4.16 (m, 1H, CH_2_CH_3_), 3.67 (d, *J* = 11.8 Hz, 1H, H-4), 3.57 (dd, *J* = 9.7 Hz, *J* = 3.5 Hz, 1H, H-5), 3.39 (s, 3H, OCH_3_), 3.09 (dd, *J* = 11.8 Hz, *J* = 4.2 Hz, 1H, H-1′), 2.87 (dd, *J* = 12.0 Hz, *J* = 11.8 Hz, 1H, H-1), 2.39–2.32 (m, 1H, H-4a), 2.13–2.05 (m, 1H, H-7a), 1.52 (s, 3H, CH_3_), 1.33–1.27 (m, 6H, CH_3_, CH_2_CH_3_) ppm. ^13^C­{H} NMR (151 MHz, CDCl_3_) δ: 170.0 (C-3), 160.2 (C=O), 150.1 (Ar), 128.9 (2C, Ar), 124.3 (Ar), 119.5 (2C, Ar), 114.2 (C^IV^), 90.0 (C-5), 84.3 (C-6), 82.3 (C-7), 61.0 (CH_2_CH_3_), 59.8 (C-4), 58.1 (OCH_3_), 48.3 (C-4a), 44.3 (C-7a), 31.7 (C-1), 27.3 (CH_3_), 25.1 (CH_3_), 14.2 (CH_2_ CH_3_) ppm. HRMS (ESI) *m*/*z*: [M + Na]^+^ Calcd for C_21_H_28_NO_5_SNa 428.1508; Found, 428.1506.

Analysis for **25**: [α]_D_^22^ = −26.2. ^1^H NMR (600 MHz, CDCl_3_) δ: 7.29 (dd, *J* = 8.3, *J* = 7.4 Hz, 2H, Ar), 7.08 (tt, *J* = 7.3, *J* = 1.2 Hz, 1H, Ar), 6.80 (dd, *J* = 8.4, *J* = 1.2 Hz, 2H, Ar), 4.66 (dd, *J* = 5.3 Hz, *J* = 5.3 Hz, 1H, H-7), 4.50 (d, *J* = 5.6 Hz, 1H, H-6), 4.32 (dd, *J* = 10.8, *J* = 7.1 Hz, 1H, CH_2_CH_3_), 4.22 (dd, *J* = 10.8, *J* = 7.1 Hz, 1H, CH_2_CH_3_), 3.96 (d, *J* = 12.2 Hz, 1H, H-4), 3.51 (d, *J* = 4.4 Hz, 1H, H-5), 3.36 (s, 3H, −OCH_3_), 3.11 (dd, *J =* 12.1 Hz, *J* = 12.1 Hz, 1H, H-1′), 2.84 (dd, *J* = 11.9, *J* = 4.0 Hz, 1H, H-1), 2.55 (ddd, *J* = 12.4 Hz, *J* = 12.3 Hz, *J* = 4.4 Hz, 1H, H-4a), 2.34 (dddd, *J* = 12.4 Hz, *J* = 12.4 Hz, *J* = 4.5 Hz, *J* = 4.5 Hz, 1H, H-7a), 1.45 (s, 3H, CH_3_), 1.30 (s, 6H, CH_3_, CH_2_CH_3_). ^13^C­{H} NMR (151 MHz, CDCl_3_) δ: 170.4 (C-3), 161.3 (C=O), 150.1 (Ar), 128.7 (2C Ar), 124.2 (Ar), 119.7 (2C Ar), 110.9 (C^IV^), 85.1 (C-5), 81.7 (C-6), 80.6 (C-7), 60.9 (CH_2_CH_3_), 57.7 (OCH_3_), 55.0 (C-4), 44.3 (C-4a), 41.2 (C-7a), 28.8 (C-1), 25.9 (CH_3_), 23.8 (CH_3_), 14.4 (CH_2_ CH_3_). LRMS (ESI) *m*/*z*: [M + Na]^+^ Calcd for C_21_H_28_NO_5_S 406.17; Found, 406.17, [M + Na]^+^ = 428.15.

#### Ethyl (3a*R*, 3b*R*, 4*R*, 7a*S*, 8*R*, 8a*R*, *Z*)-8-((*Tert*-butoxycarbonyl)­oxy)-2,2-dimethyl-5-(phenylimino)­octahydrothiopyran­[4′,3′:3,4]­cyclopenta­[1,2-*d*]­[1,3]­dioxole-4-carboxylate **(24)**

The iodomannopyranoside **6** (1.3 g, 2.93 mmol, 1.0 equiv) was dissolved in a mixture of THF (9.0 mL) and H_2_O (1.0 mL), and zinc (preactivated with 1:9 v/v solution of HCl/H_2_O, 1.9 g, 29.3 mmol, 10.0 equiv) was added, and the flask was immersed in a sonication apparatus preheated to 50 °C. The solution was sonicated for 30 min, after which TLC revealed the full conversion of the starting material (the product does not appear under UV; the reaction mixture turns a bright gray color). The suspension was passed through a plug of Celite using diethyl ether (10 mL). The combined filtrates were concentrated and dried under reduced pressure to give aldehyde **10** as a yellow oil. To a mixture of freshly prepared aldehyde **10** in anhydrous acetonitrile (5.0 mL) and molecular sieves 4A under an inert gas atmosphere, compound **13** (1.14 g, 5.10 mmol, 2.0 equiv) was added. The reaction mixture was stirred at 70 °C in an oil bath for 2 h (color turns to bright yellow) and at RT for 24 h. The crude product was absorbed on Celite and purified by flash column chromatography (SiO_2_, EtOAc/toluene with a gradient of 0–20% EtOAc) to yield 1.01 g of the desired product, contaminated with an orange solid. Product **24** was not pure enough for analysis, and it was used in the next step without further purification.

#### Ethyl (3a*S*, 3b*R*, 7*S*, 7a*S*, 8*S*, 8a*S*, *Z*)-8-((*Tert*-butoxycarbonyl)­oxy)-2,2-dimethyl-6-(phenylimino)­octahydrothiopyran­[3′,4’:3,4]­cyclopenta­[1,2-*d*]­[1,3]­dioxole-7-carboxylate **(26)**

The iodogalactopyranoside **8** (2.8 g, 6.30 mmol, 1.0 equiv) was dissolved in a mixture of THF (9.0 mL) and H_2_O (1.0 mL), and zinc (preactivated with 1:9 v/v solution of HCl/H_2_O, 4.12 g, 63.0 mmol, 10.0 equiv) was added, and the flask was immersed in a sonication apparatus preheated to 50 °C. The solution was sonicated for 30 min, after which TLC revealed the full conversion of the starting material (the product does not appear under UV; the reaction mixture turns a bright gray color). The suspension was passed through a plug of Celite using diethyl ether (10 mL). The combined filtrates were concentrated and dried under reduced pressure to give aldehyde **12** as a yellow oil. To a mixture of freshly prepared aldehyde **12** in anhydrous acetonitrile (7.0 mL) and molecular sieves 4A under an inert gas atmosphere, compound **13** (2.8 g, 12.6 mmol, 2.0 equiv) was added. The reaction mixture was stirred at 70 °C in an oil bath for 2 h (color turns to bright yellow) and at RT for 24 h. Crude was absorbed on Celite and purified by flash column chromatography (SiO_2_, EtOAc/toluene with a gradient of 0–10% EtOAc). Due to significant contamination, the product was purified by flash column chromatography (Al_2_O_3_, EtOAc/hexanes with a gradient of 0–10% EtOAc) to yield 1.06 g of the title compound. Product **26** was not pure enough for analysis, and it was used in the next step without further purification.

#### Ethyl (4*R*, 4a*R*, 5*R*, 6*R*,7*R*, 7a*S*)-5,6-dihydroxy-7-methoxy-3-oxooctahydrocyclopenta­[*c*]­thiopyran-4-carboxylate **(28)**

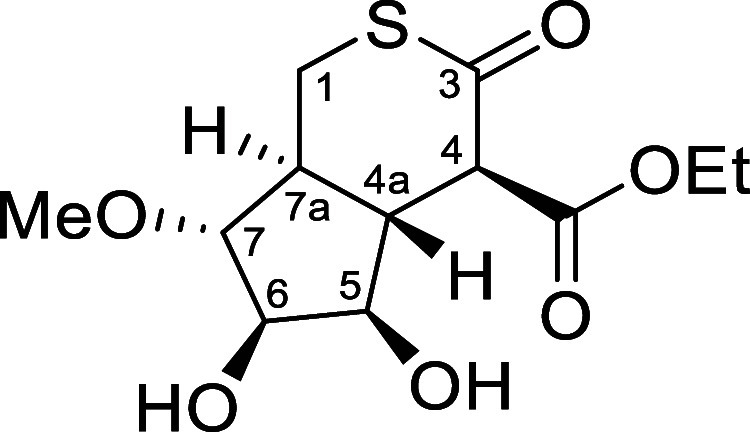



A solution of **23** (0.1 g, 0.25 mmol) in MeCN/H_2_O (9:1, v/v, 0.5 mL) was stirred with Amberlyst 15 resin (1.5 g per 200 mg of starting material) and the mixture was stirred at 50 °C in an oil bath for 24 h. The reaction mixture was filtered, and the volatiles were evaporated. Crude was purified by flash column chromatography (SiO_2_, EtOAc/hexanes with a gradient of 0–50% EtOAc) to yield 55 mg (77%) of the desired product as a yellowish solid. [α]_D_^27^ = +4.7. ^1^H NMR (600 MHz, Acetone-*d*_6_) δ: 4.12 (qd, *J* = 7.1 Hz, *J* = 2.0 Hz, 2H, CH_2_CH_3_), 3.91 (dd, *J* = 7.1 Hz, *J* = 7.1 Hz, 1H, H-6), 3.86 (dd, *J* = 8.8 Hz, *J* = 6.8 Hz, 1H, H-5), 3.60 (d, *J* = 11.9 Hz, 1H, H-4), 3.42 (s, 3H, OCH_3_), 3.39 (ddd, *J* = 8.9 Hz, *J* = 7.6 Hz, *J* = 3.5 Hz, 1H, H-7), 3.36 (d, *J* = 11.8 Hz, 1H, H-1′), 3.32 (dd, *J* = 11.5 Hz, *J* = 4.7 Hz, 1H, H-1), 2.43 (ddd, *J* = 12.1 Hz, *J* = 12.0 Hz, *J* = 8.9 Hz, 1H, H-4a), 2.03–1.99 (m, 1H, H-7a), 1.21 (t, *J* = 7.1 Hz, 3H, CH_2_CH_3_). ^13^C­{H} NMR (151 MHz, Acetone-*d*_6_) δ: 196.3 (C-3), 168.0 (C = O), 90.9 (C-7), 74.8 (C-6), 74.3 (C-5), 63.6 (C-4), 60.6 (CH_2_CH_3_), 57.1 (OCH_3_), 47.7 (C-4a), 39.9 (C-7a), 33.3 (C-1), 13.4 (CH_2_ CH_3_). LRMS (ESI) *m*/*z*: [M + Na]^+^ Calcd for C_12_H_18_O_6_SNa 313.07; Found, 313.07.

#### Ethyl (4*R*, 4a*R*, 5*R*, 6*R*, 7*R*, 7a*S*)-5,6,7-Trihydroxy-3-oxooctahydrocyclopenta­[*c*]­thiopyran-4-carboxylate **(29)**

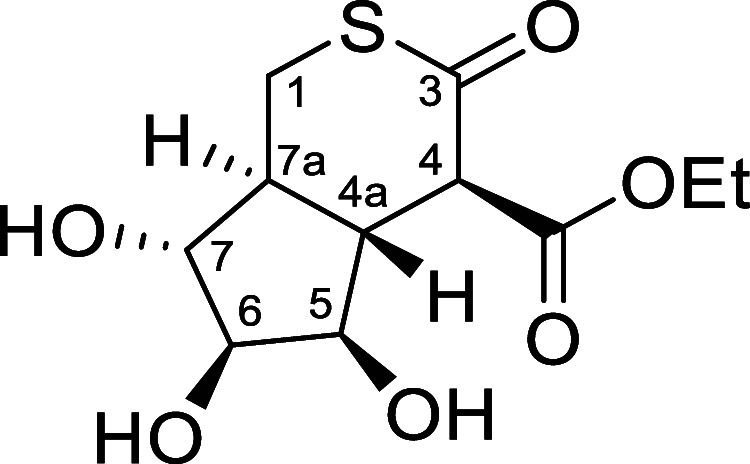



A solution of **24** (1.01 g) in MeCN/H_2_O 9:1 v/v (8.0 mL) was stirred with Amberlyst 15 resin (1.5 g per 200 mg of starting material) at 50 °C in an oil bath for 24 h. The reaction mixture was filtered, and the volatiles were evaporated. The crude product was purified by flash column chromatography (SiO2, EtOAc/hexanes with a gradient of 0–50% EtOAc) to yield 125.4 mg (16% after four steps) of the desired product as a brownish oil. Product crystallizes out of an acetone-*d*_6_/DCM mixture. [α]_D_^27^ = −6.1. ^1^H NMR (400 MHz, Acetone-*d*_6_) δ: 4.13 (qd, *J* = 7.1 Hz, *J* = 1.2 Hz, 2H, CH_2_CH_3_), 3.95–3.83 (m, 2H, H-5, H-6), 3.73 (dd, *J* = 8.2 Hz, *J* = 3.6 Hz, 1H, H-7), 3.60 (d, *J* = 12.0 Hz, 1H, H-4), 3.35 (dd, *J* = 11.5 Hz, *J* = 4.7 Hz, 1H, H-1), 3.31 (dd, *J* = 11.6 Hz, *J* = 11.5 Hz, 1H, H-1′), 2.38 (td, *J* = 12.2 Hz, *J* = 7.1 Hz, 1H, H-4a), 1.98 (dddd, *J* = 11.5 Hz, *J* = 8.1 Hz, *J* = 7.2 Hz, *J* = 4.7 Hz, 1H, H-7a), 1.22 (t, *J* = 7.1 Hz, 3H, CH_2_CH_3_) ppm. ^13^C­{H} NMR (101 MHz, Acetone-*d*_6_) δ: 197.4 (C-3), 169.0 (C=O), 82.4 (C-7), 78.2 (C-6), 74.3 (C-5), 64.5 (C-4), 61.6 (CH_2_CH_3_), 49.5 (C-4a), 42.3 (C-7a), 34.0 (C-1), 14.4 (CH_2_ CH_3_) ppm. HRMS (ESI) *m*/*z*: [M + Na]^+^ Calcd for C_11_H_16_O_6_SNa 299.0565; Found, 299.0566.

#### Ethyl (4*S*, 4a*S*, 5*S*, 6*S*, 7*S*, 7a*R*)-6,7-dihydroxy-5-methoxy-3-oxooctahydrocyclopenta­[*c*]­thiopyran-4-carboxylate **(32)**

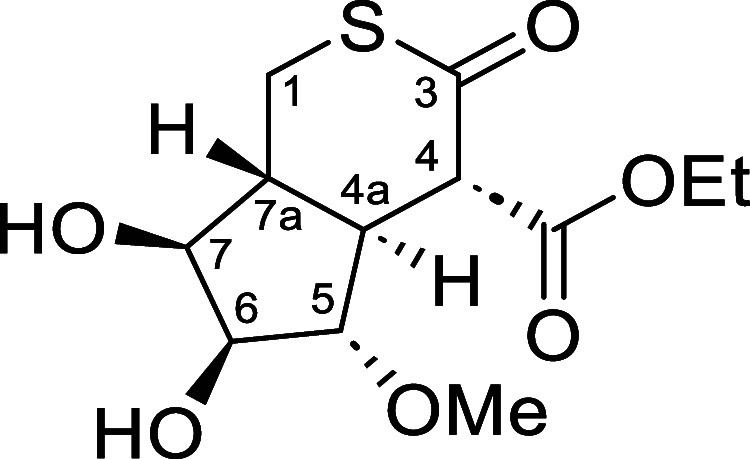



A solution of **27** (50 mg, 0.12 mmol) in MeCN/H_2_O 9:1 v/v (0.5 mL) was stirred with Amberlyst 15 resin (1.5 g per 200 mg of starting material) at 50 °C in an oil bath for 24 h. The reaction mixture was filtered, and the volatiles were evaporated under reduced pressure. The crude product was purified by flash column chromatography (SiO2, EtOAc/hexanes with a gradient of 0–60% EtOAc) to yield 12.7 mg (36%) of the desired product as a transparent oil. [α]_D_^22^ = −9.9. ^1^H NMR (500 MHz, Acetone-*d*_6_) δ: 4.22–4.12 (m, 3H, H-7, CH_2_CH_3_), 4.10 (dd, *J* = 4.9 Hz, *J* = 2.9 Hz, 1H, H-6), 3.67 (d, *J* = 12.6 Hz, 1H, H-4), 3.54 (dd, *J* = 11.8 Hz, *J* = 11.9 Hz, 1H, H-1′), 3.52 (dd, *J* = 7.2 Hz, *J* = 2.8 Hz, 1H, H-5), 3.31 (s, 3H, OCH_3_), 3.09 (dd, *J* = 11.5 Hz, *J* = 4.8 Hz, 1H, H-1), 2.90 (ddd, *J* = 12.6 Hz, *J* = 12.6, Hz, *J* = 7.3 Hz, 1H, H-4a), 2.38 (dddd, *J* = 12.3 Hz, *J* = 12.3 Hz, *J* = 4.5 Hz, *J* = 4.4 Hz, 1H, H-7a), 1.24 (t, *J* = 7.1 Hz, 3H, CH_2_CH_3_). ^13^C­{H} NMR (126 MHz, Acetone-*d*_6_) δ: 197.7 (C-3), 168.9 (C=O), 87.7 (C-5), 79.9 (C-6), 73.6 (C-7), 61.4 (CH_2_CH_3_), 60.6 (C-4), 57.5 (OCH_3_), 44.7 (C-4a), 41.9 (C-7a), 30.4 (C-1), 13.7 (CH_2_ CH_3_). HRMS (ESI) *m*/*z*: [M + Na]^+^ Calcd for C_12_H_18_O_6_SNa 313.0722; Found, 313.0721.

#### Ethyl (4*R*, 4a*R*, 5*S*, 6*S*, 7*S*, 7a*S*)-6,7-dihydroxy-5-methoxy-3-oxooctahydrocyclopenta­[*c*]­thipyran-4-carboxylate **(30)**

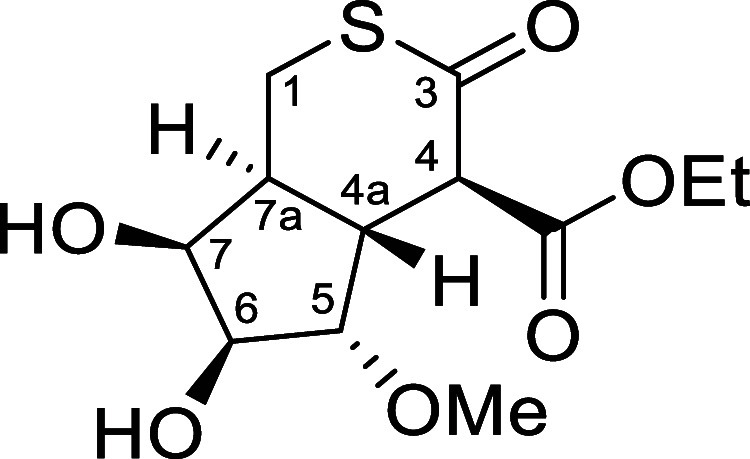



A solution of **25** (0.389 mg, 0.96 mmol) in MeCN/H_2_O 9:1 v/v (0.5 mL) was stirred with Amberlyst 15 resin (1.5 g per 200 mg of starting material) at 50 °C in an oil bath for 24 h. The reaction mixture was filtered, and the volatiles were evaporated. The crude product was purified by flash column chromatography (SiO2, EtOAc/hexanes with a gradient of 0–50% EtOAc) to yield 115 mg (41%) of the desired product as a yellow oil. [α]_D_^23^ = −63.2. ^1^H NMR (500 MHz, Acetone-*d*_6_) δ: 4.18–4.07 (m, 2H, CH_2_CH_3_), 3.94 (dd, *J* = 6.6 Hz, *J* = 2.5 Hz, 1H), 3.83 (dd, *J* = 9.1 Hz, *J* = 6.5 Hz, 1H), 3.54 (d, *J* = 11.6 Hz, 1H, H-4), 3.39–3.37 (m, 1H), 3.35–3.31 (m, 5H, OCH_3_, H-1), 2.22–2.13 (m, 2H, H-4a, H-7a), 1.23 (t, *J* = 7.1 Hz, 3H, CH_2_CH_3_). ^13^C­{H} NMR (126 MHz, Acetone-*d*_6_) δ: 197.3 (C-3), 169.0 (C=O), 92.4 (C-7), 75.4 (C-5), 75.3 (C-6), 64.6 (C-4), 61.6 (CH_2_CH_3_), 57.9 (OCH_3_), 46.6, 42.9, 33.5 (C1), 14.4 (CH_2_ CH_3_). HRMS (ESI) *m*/*z*: [M + Na]^+^ Calcd for C_12_H_18_O_6_SNa 313.0722; Found, 313.0726.

#### Ethyl (4*S*, 4a*S*, 5*S*, 6*R*, 7*S*, 7a*R*)-5,6,7-Trihydroxy-3-oxooctahydrocyclopenta­[*c*]­thio­[pyran-4-carboxylate **(31)**

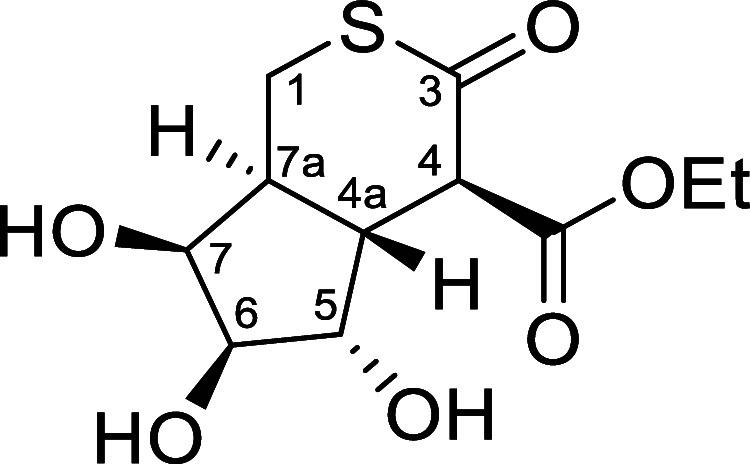



A solution of **26** (1.06 g) in MeCN/H_2_O (9:1, v/v, 8.0 mL) was stirred with Amberlyst 15 resin (1.5 g per 200 mg of starting material) at 50 °C in an oil bath for 24 h. The reaction mixture was filtered, and the volatiles were evaporated. The crude product was purified by flash column chromatography (SiO2, EtOAc/hexanes with a gradient of 0–50% EtOAc) to yield 67.3 mg (4% after four steps) of the desired product as a yellow oil. [α]_D_^27^ = −20.0. ^1^H NMR (400 MHz, Acetone-*d*_6_) δ: 4.24 (dd, *J* = 5.0 Hz, 1H, H-7), 4.20–4.08 (m, 2H, CH_2_CH_3_), 4.04 (dd, *J* = 5.0 Hz, *J* = 2.5 Hz, 1H, H-6), 3.96 (dd, *J* = 6.3 Hz, *J* = 2.6 Hz, 1H, H-5), 3.75 (d, *J* = 12.5 Hz, 1H, H-4), 3.52 (t, *J* = 11.9 Hz, 1H, H-1), 3.10 (dd, *J* = 11.6 Hz, *J* = 4.6 Hz, 1H, H-1′), 2.79 (ddd, *J* = 12.5, *J* = 12.5 Hz, *J* = 6.2 Hz, 1H, H-4a), 2.44 (dddd, *J* = 12.4 Hz, *J* = 12.3 Hz, *J* = 4.8 Hz, *J* = 4.8 Hz, 1H, H-7a), 1.22 (t, *J* = 7.1 Hz, 3H, CH_2_CH_3_) ppm. ^13^C­{H} NMR (101 MHz, Acetone-*d*_6_) δ: 198.1 (C-3), 169.0 (C=O), 81.6 (C-5), 77.9 (C-6), 72.9 (C-7), 61.4 (CH_2_CH_3_), 60.7 (C-4), 45.4 (C-4a), 41.4 (C-7a), 30.7 (C-1), 14.4 (CH_2_ CH_3_) ppm. HRMS (ESI) *m*/*z*: [M + Na]^+^ Calcd for C_11_H_16_O_6_SNa 299.0565; Found, 299.0567.

### Computational Methods

All DFT calculations were performed using the Gaussian 16 software suite.^60^ Initial conformational sampling was performed using the CREST^61^ program, which employs the semiempirical GFN2-xTB^62^ method to efficiently explore conformational space. Geometry optimizations and frequency calculations for relevant structures were performed at the density functional theory (DFT) level using the M06-2X/def2-TZVP level of theory^63,64^ with the SMD implicit solvation model^65^ in acetonitrile. Dispersion effects were included using Grimme’s D3 correction.^66^ Transition states were located and verified via frequency calculations, confirming the presence of a single imaginary frequency corresponding to the Diels–Alder reaction coordinate.

### Biological Activity

#### Consumables

50 mL sterile Falcon tubes (NeoCulture centrifuge tubes, made of PP, 50 mL, self-standing, sterile) and phage-safe^67^ 1.5 mL Eppendorf-type tubes (B-1429 and B-2278) were purchased from Bionovo (Poland).

#### Chemicals

LB medium and LB-agar −10 g L^–1^ of NaCl, 10 g L^–1^ of tryptone, and 5 g L^–1^ of yeast extract. For LB-agar, 15 g L^–1^ of agar was added to the LB medium. The media were ordered from Carl Roth (Germany). TM (*Tris*-Magnesium) buffer (pH 7.4) was prepared by mixing 10 mM *Tris* base, 5 μM CaCl_2_, 10 mM MgSO_4_, and deionized water (MiliQ water purification system). PBS (phosphate-buffered saline) was obtained by dissolving tablets ordered from MP Biomedicals (Germany).

Due to the poor solubility of the examined compounds, it was necessary to add dimethyl sulfoxide (DMSO) to facilitate sample preparation. The main stock was prepared by dissolving 10 mg of the compound in 200 μL of DMSO. Then 800 μL of distilled water was added. The concentration of a compound was 10 mg mL^–1^, and the concentration of DMSO was 20%. While preparing samples with bacteria and phages, 100 μL of the prepared stock was used, which was diluted ten times. The final concentration of the compound in the sample was 1 mg mL^–1^, with 2% DMSO.

We have also tested concentrations of 0.1 mg mL^–1^ and 0.5 mg mL^–1^, but no inactivation was observed in any of the experiments.

#### Bacteria

The *E. coli* strain BL21 (obtained from the Institute of Biochemistry and Biophysics in Warsaw, Poland) was used as the host for the T4 phage. For MS2, the *E. coli* C3000 strain (obtained from the Institute of Biochemistry and Biophysics in Warsaw, Poland) was used.

A single colony of the required strain was picked up from the stock plate and transferred to 10 mL of LB medium to prepare the bacterial cultures. This sample was then incubated overnight at 37 °C in an orbital shaker-incubator (ES-20, 200 rpm). The sample was refreshed by mixing 2.5 mL of the overnight culture with 7.5 mL of LB medium and incubating at 37 °C for approximately 1 h.

#### Titration for Bacterial Analysis

The synthesized compounds were evaluated against *E. coli* BL21 and *E. coli* C3000 (a Gram-negative bacterium) upon 24 h of incubation at room temperature while shaking (ES-20, 180 rpm). The initial concentration of *E. coli* cells was set at 10^5^ colony-forming units per milliliter (CFU mL^–1^). Samples contained: 100 μL of a 20% thiolactone aqueous solution, 100 μL of bacterial suspension, and 800 μL of PBS buffer. Control included: a) 100 μL of bacteria suspension and 900 μL of PBS buffer, and b) 100 μL of DMSO 20% aqueous solution, 100 μL of bacteria suspension, and 800 μL of PBS buffer. Samples were incubated for 24 h at room temperature on an orbital shaker (180 rpm).

LB-agar medium (20 mL) was poured into Petri plates and left to solidify. Dilutions of the bacterial solutions were prepared, and for each dilution, eight 5 μL droplets were spotted onto the LB-agar layer. After incubation of the plates at 37 °C for 24 h, the number of colonies was counted. The experiment was conducted in three biological replicates.

#### Bacteriophages

T4 (Straboviridae) and MS2 (Fiersviridae) phages were purchased from Phage Consultants (Poland).

#### Double Overlay Titration for Phage Analysis

The antiviral activity of the compounds (dissolved in 20% aqueous solution of DMSO) was assessed using bacteriophages T4 (initial titer ≈10^7^ plaque-forming units (PFU mL^–1^)). Samples contained: 100 μL of a thiolactone in 20% DMSO aqueous solution, 100 μL of phage suspension, and 800 μL of TM buffer. Final concentration of thiolactones: 1.0 mg mL^–1^; **28**, **30**, **32**: 3.45 mM; **29**, **31**: 3.64 mM. Control contained: a) 100 μL of phage suspension and 900 μL of TM buffer, and b) 100 μL of 20% DMSO aqueous solution, 100 μL of phage suspension, and 800 μL of TM buffer. Samples were incubated for 24 h at room temperature on an orbital shaker (180 rpm).

LB-agar medium (20 mL) was poured into Petri plates and left to solidify. Four mL of top LB agar (prepared with liquid medium and 0.5% agar) was mixed with 200 μL of the refreshed bacterial culture and poured onto the plate. Dilutions of the phage solutions were prepared, and from each dilution, eight 5 μL droplets were spotted onto the top agar layer. After incubation of the plates at 37 °C for 24 h, the number of plaques was counted. All titrations were performed in three biological replicates.

#### Results for *E. coli* C3000 and MS2 Phage

The same experimental procedure described above was applied to *E. coli* C3000 and its corresponding phage, MS2. Neither the bacterial strain nor the MS2 phage showed any statistically significant change in survival after 24 h incubation with any of the tested thiolactone compounds (1 mg mL^–1^, 2% DMSO, v/v). These data are presented in Figure S5 in the Supporting Information.

#### Statistical Analysis

All experimental results were analyzed using Microsoft Excel software. Data were expressed as mean ± SD. Dixon’s Q test was used to reject outliers. Statistical significance was evaluated using a two-sided Welch’s *t* test. *P < 0.05; **P < 0.01; ***P < 0.001.

## Supplementary Material





## Data Availability

The data underlying this study are available in the published article and its Supporting Information.

## Notes

Crystal data of compounds **28** and **29**; biological assay for final compounds and computational methods for cyclization reaction; NMR and HRMS spectra of all synthesized compounds Crystal data of **28** (CIF) Crystal data of **29** (CIF) Coordinates of computed molecules (PDF)
Cartesian Coordinates (ZIP)

## Notes

This manuscript is dedicated to the memory of Professor Mieczysław Mąkosza.
