# Supplementary material for: Bicyclic δ‑Thiolactone Glycomimetics: Stereoselective Synthesis and Discovery of Stereocontrolled Antiphage Activity
Source: J Org Chem. 2026 Apr 2;91(15):5366–78. doi: 10.1021/acs.joc.6c00212 (PMC13298889; doi:10.1021/acs.joc.6c00212)
Supplement: Supplementary file 1 [file jo6c00212_si_002.pdf]

Supporting Information

Bicyclic δ-Thiolactone Glycomimetics: Stereoselective Synthesis and Discovery of Stereocontrolled Antiphage Activity

Karol Postrożny[a], Bartosz Kamiński[b], Aleksandra F. Koper[a], Roman Luboradzki[b], Zahra Badri[a]*, Jan Paczesny[b]* and Mykhaylo A. Potopnyk[a,c]*

[a] Institute of Organic Chemistry, Polish Academy of Sciences, Kasprzaka 44/52, Warsaw, 01-224, Poland, Email: zahra.badri@icho.edu.pl; mykhaylo.potopnyk@icho.edu.pl

[b] Institute of Physical Chemistry, Polish Academy of Sciences, Kasprzaka 44/52, Warsaw, 01-224, Poland, Email: jpaczesny@ichf.edu.pl

[c] Department of Organic Chemistry, Ivan Franko National University of Lviv, Kyryla and Mefodiya 6, Lviv, 79005, Ukraine, Email: potopnyk@gmail.com

Contents

1. Synthesis S3
2. X-ray crystallographic analysis S4
3. Computational methods S7
4. Biological activity S12
5. Copies of NMR and MS spectra S13

1. Synthesis

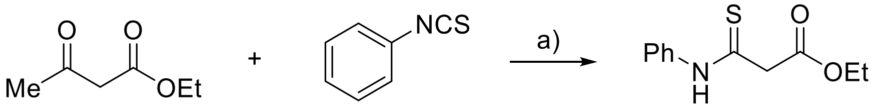

S1 **S2** **13**

Scheme S1. Synthesis of thioamide **13**. a) Na, EtOH, RT, 24 h.

2. X-ray crystallographic analysis

Table S1. Crystal data for compound **28**.

Compound	**28**
Empirical formula	$C_{12}H_{18}O_6S$
Moiety formula	$C_{12}H_{18}O_6S$
Formula weight	290.339
CCDC No	CCDC2499161
Wavelength	1.54184
Crystal system	orthorhombic
Space group	$P2_12_12_1$
Unit cell dimensions	a = 4.98339(13) Å
	b = 14.1219(3) Å
	c = 18.8381(4) Å
Volume	1325.73(6) Å^3
Z	4
Density Calc.	1.455 g/cm^3
Absorption coefficient	2.381 mm^{-1}
F(000)	619.559
Crystal	colourless needle
Crystal size	0.4 × 0.06 × 0.04 mm
Index ranges	-5≤ h ≤ 6, -17 ≤ k ≤ 17, -22 ≤ l ≤ 23
Reflections collected (all / independent)	21422 / 2525 [R_{int} = 0.1743]
Absorption correction	multi-scan
Refinement method	Gauss-Newton minimisation
Restraints / parameters	0 / 201
Goodness-of-fit on F^2	1.0477
Final R indices [$F^2 > 2\sigma(F^2)$]	R_1 = 0.0456, wR$_2$ = 0.1081
R indices (all data)	R_1 = 0.0503, wR$_2$ = 0.1143

Table S2. Crystal data for compound **29**.

Compound	**29**
Empirical formula	$C_{11}H_{16}O_6S$
Moiety formula	$C_{11}H_{16}O_6S$
Formula weight	276.312
CCDC No.	CCDC2499139
Wavelength	1.54184
Crystal system	orthorhombic
Space group	$P2_12_12_1$
Unit cell dimensions	a = 6.40693(6) Å
	b = 8.82808(8) Å
	c = 21.2055(2) Å
Volume	1199.40(2) $Å^3$
Z	4
Density Calc.	1.530 g/cm^3
Absorption coefficient	2.599 mm^{-1}
F(000)	587.482
Crystal	colourless block
Crystal size	0.3 × 0.3 × 0.3 mm
Index ranges	$-7 \leq h \leq 7$, $-10 \leq k \leq 10$, $-22 \leq l \leq 25$
Reflections collected (all / independent)	22747 / 2285 [R_{int} = 0.0483]
Absorption correction	multi-scan
Refinement method	Gauss-Newton minimisation
Restraints / parameters	0 / 192
Goodness-of-fit on F^2	1.0591
Final R indices [$F^2 > 2\sigma(F^2)$]	R_1 = 0.0233, wR_2 = 0.0605
R indices (all data)	R_1 = 0.0233, wR_2 = 0.0606

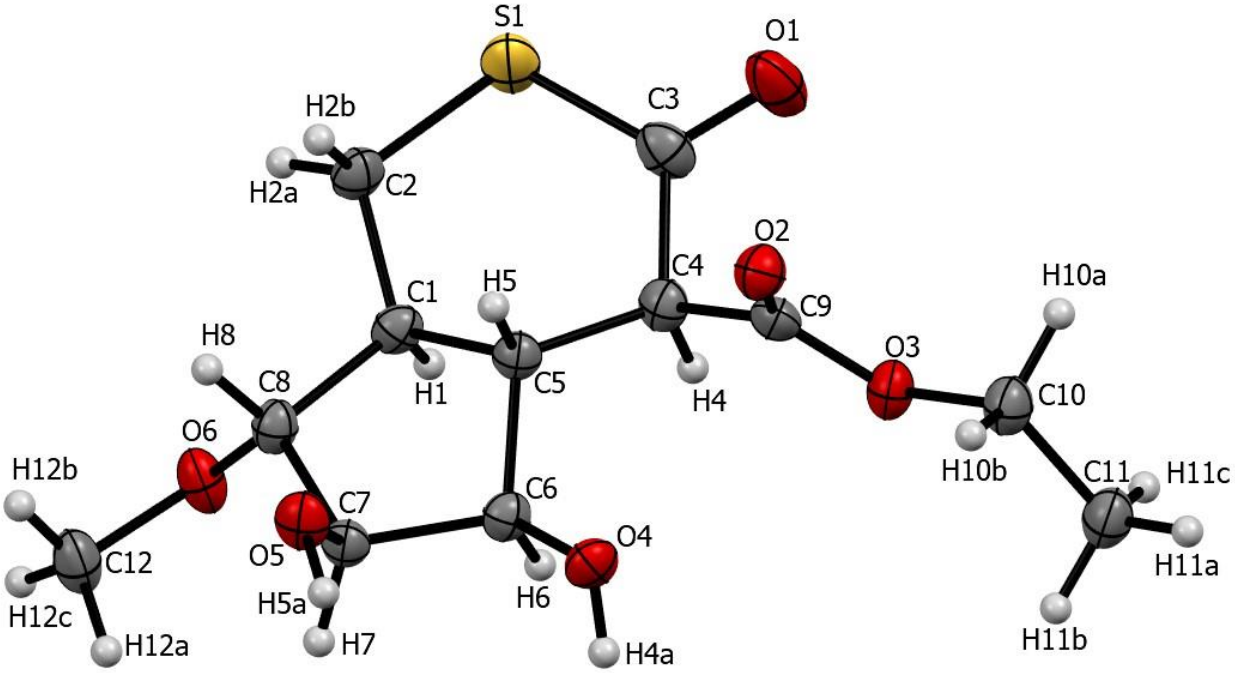

Figure S1. ORTEP diagram of compound **28**. The ellipsoid contour of probability level is 50%.

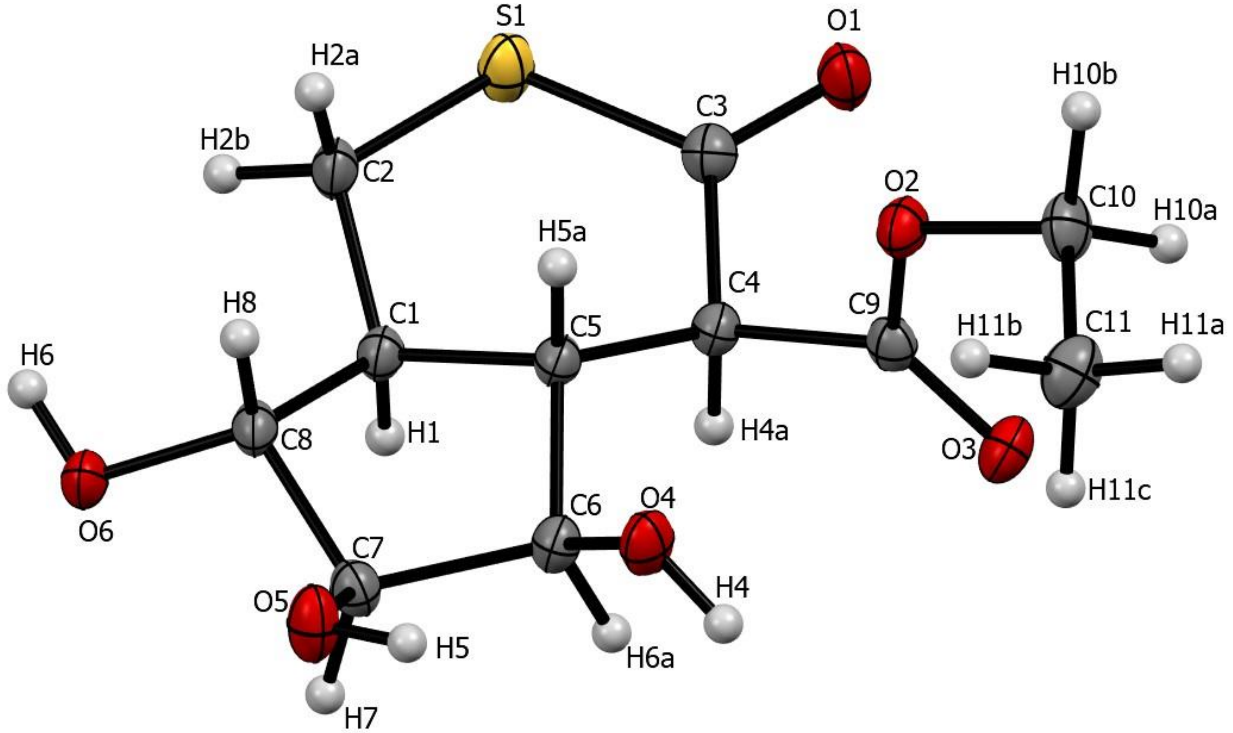

Figure S2. ORTEP diagram of compound **29**. The ellipsoid contour of probability level is 50%.

3. Computational methods

Table S3. Computed activation barriers (ΔG‡) for the Diels-Alder cycloaddition and relative free energies (ΔG, kcal mol^{-1}) of the corresponding final tautomerized products **26/26a** at the M06-2X/def2-TZVP level of theory with the SMD (MeCN) solvation model.

System	Path	ΔG‡ (TS-precomplex)	ΔG(Product-Unfavored Product)
Galactoside	**26**	19.7	-1.7
Galactoside	**26a**	22.7	0

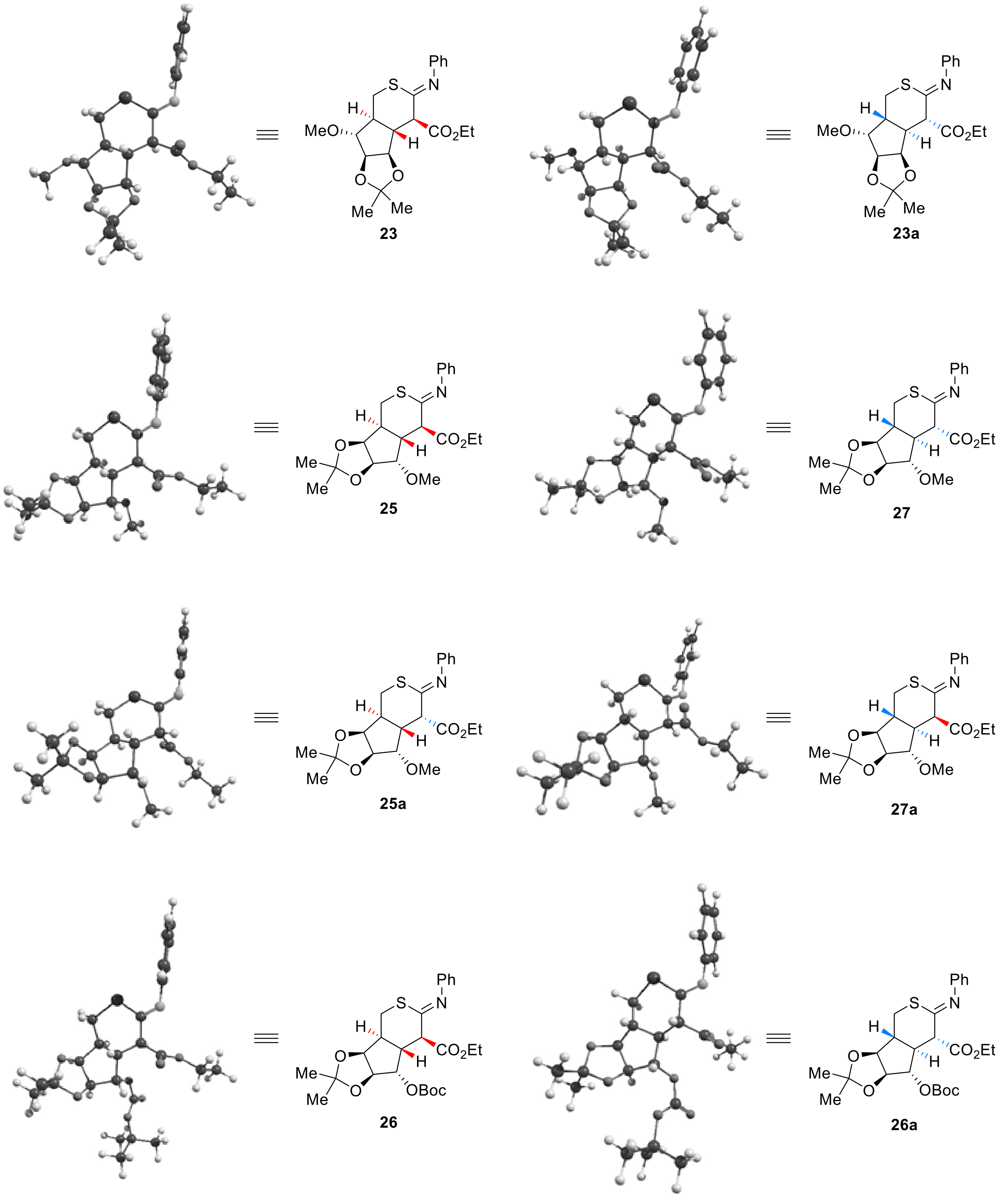

Figure S3. Optimized Structures of **23, 23a, 25**, **27**, **25a**, **27a**, **26**, and **26a** at M06-2X/def2-TZVP level of theory.

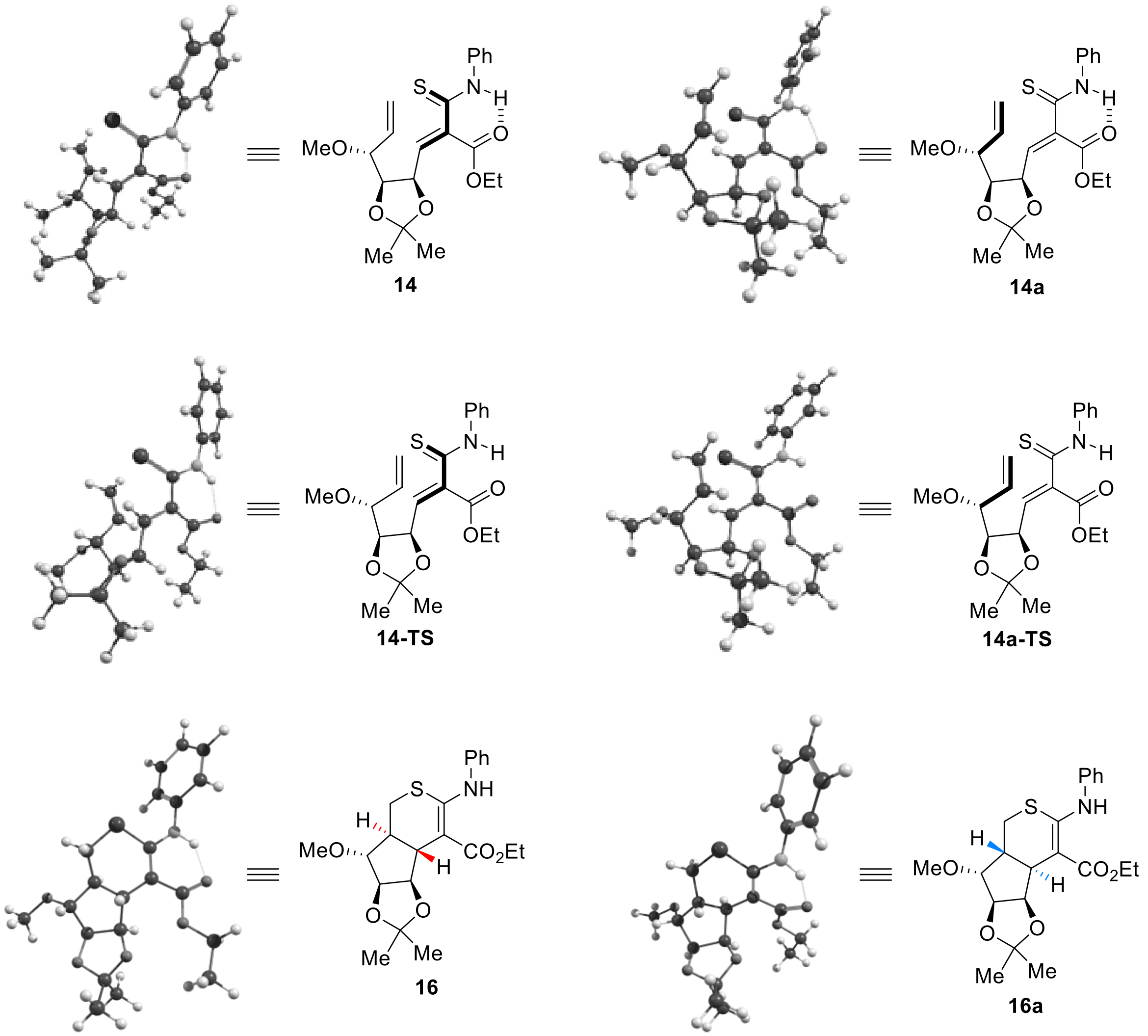

Figure S4. Optimized precomplex, transition state and intermediate structures at M06-2X/def2-TZVP level of theory.

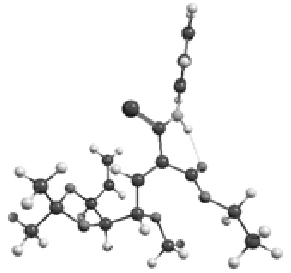

≡

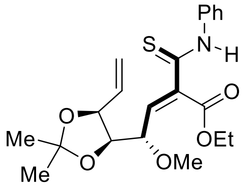

18

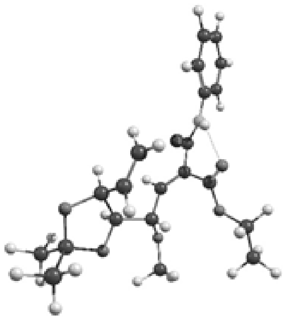

≡

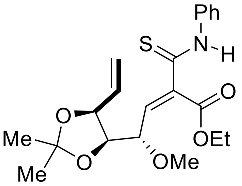

18a

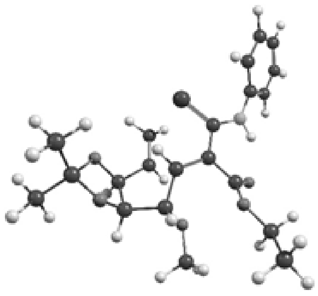

≡

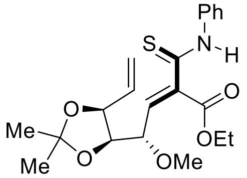

18-TS

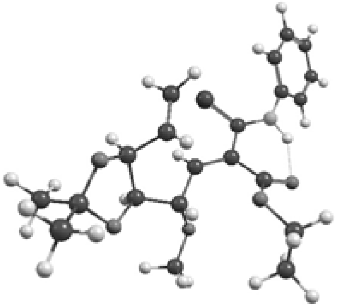

≡

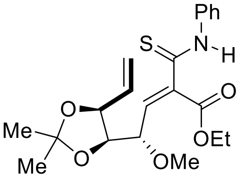

18a-TS

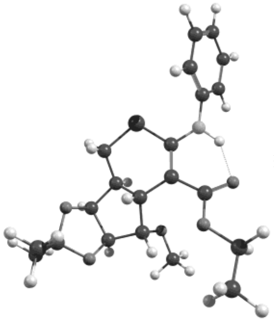

≡

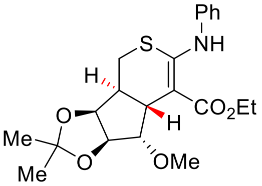

20

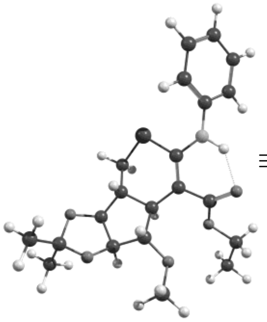

≡

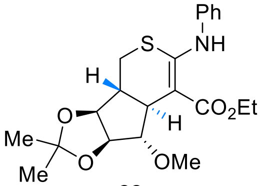

22

Figure S4. (continued).

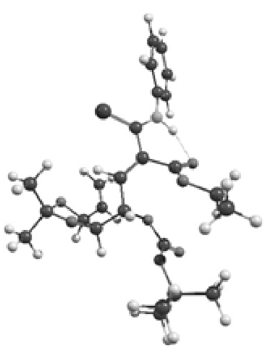

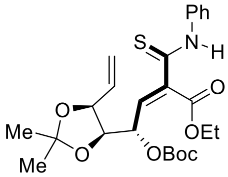

19

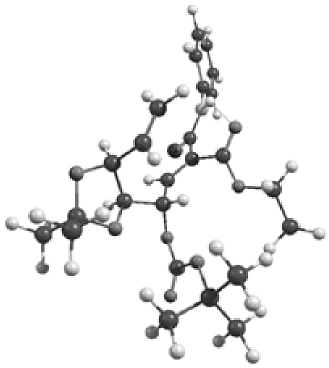

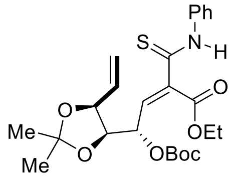

19a

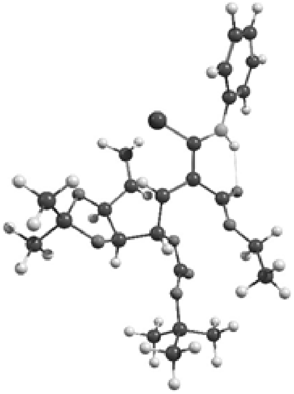

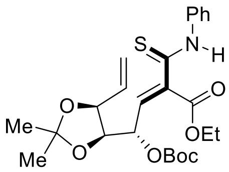

19-TS

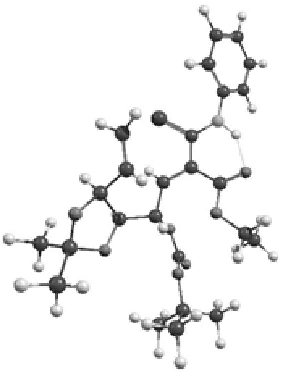

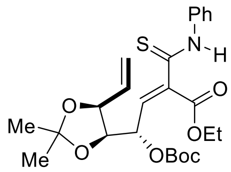

19a-TS

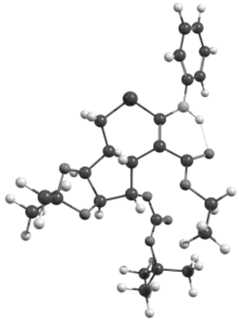

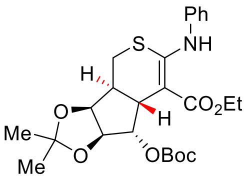

21

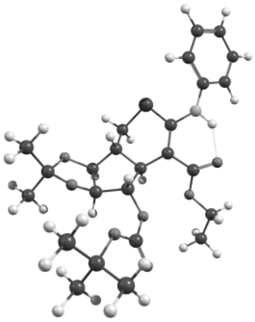

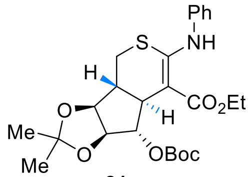

21a

Figure S4. (continued).

4. Biological activity

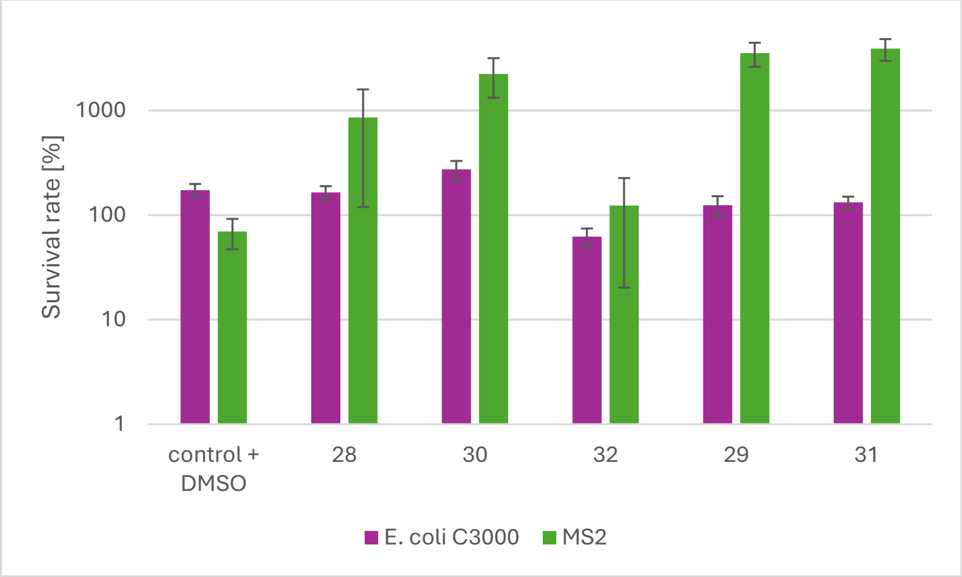

Figure S5. Effect of thiolactone glycomimetics on *E. coli* C3000 (violet) and phage MS2 (green).

5. Copies of NMR and MS spectra

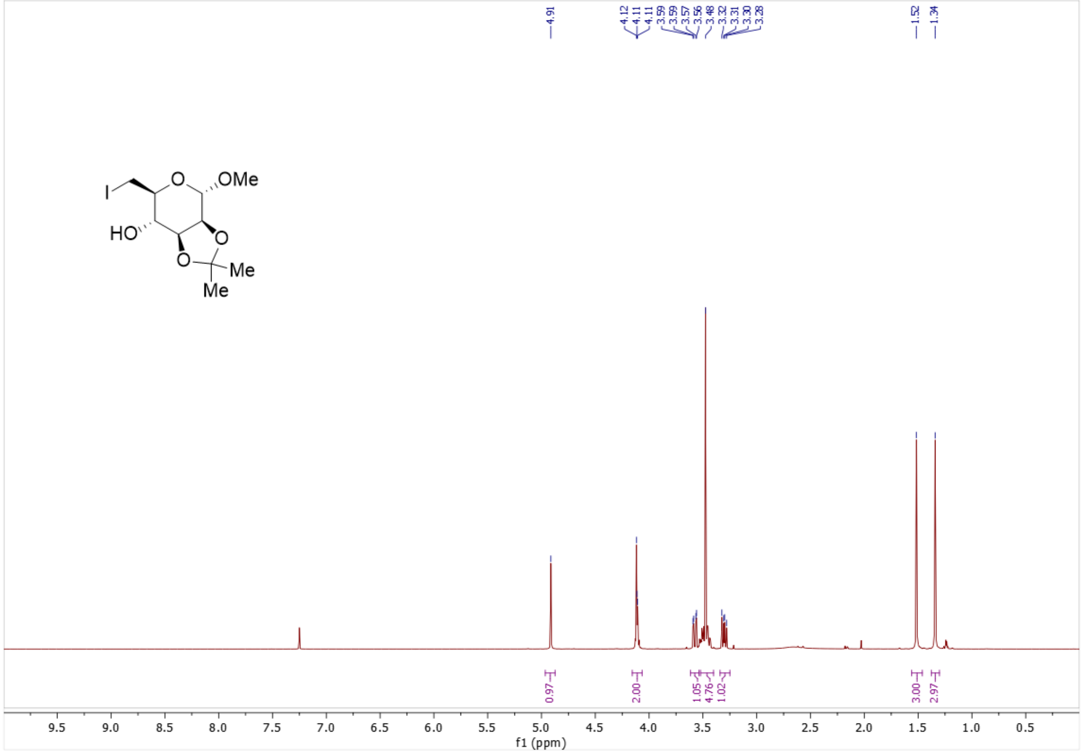

Figure S6. ^{1}H NMR (400 MHz, $CDCl_3$) spectrum of compound **3**.

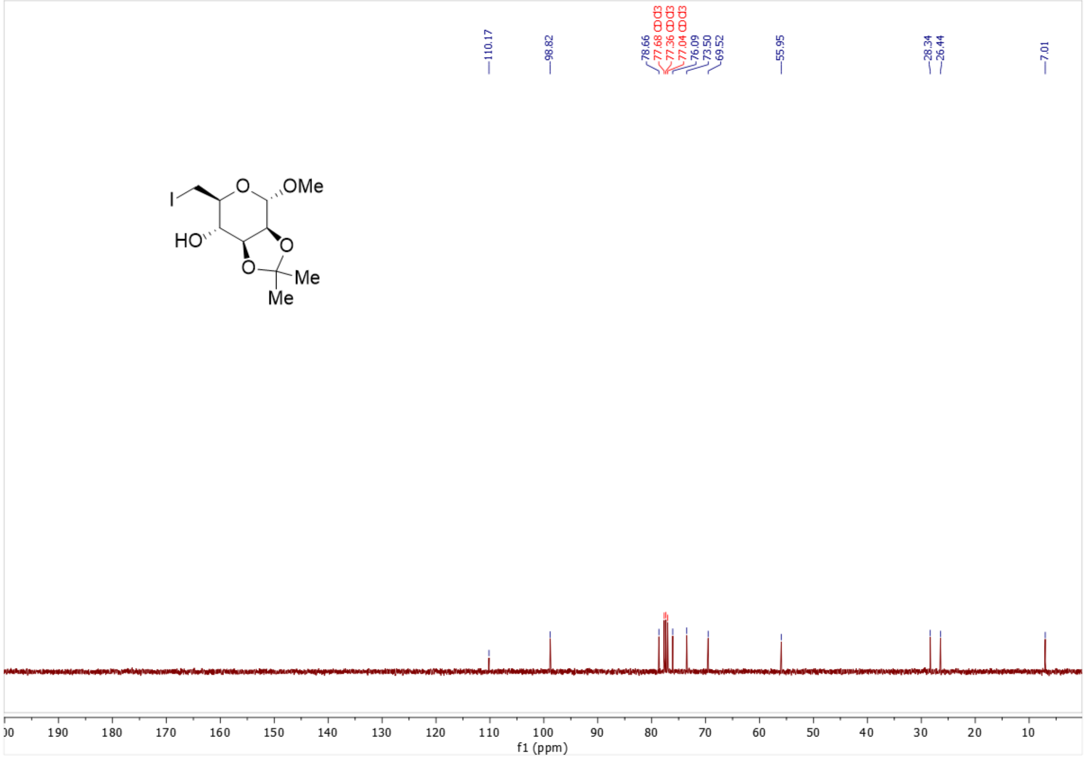

Figure S7. $^{13}C\{H\}$ NMR (101 MHz, $CDCl_3$) spectrum of compound **3**.

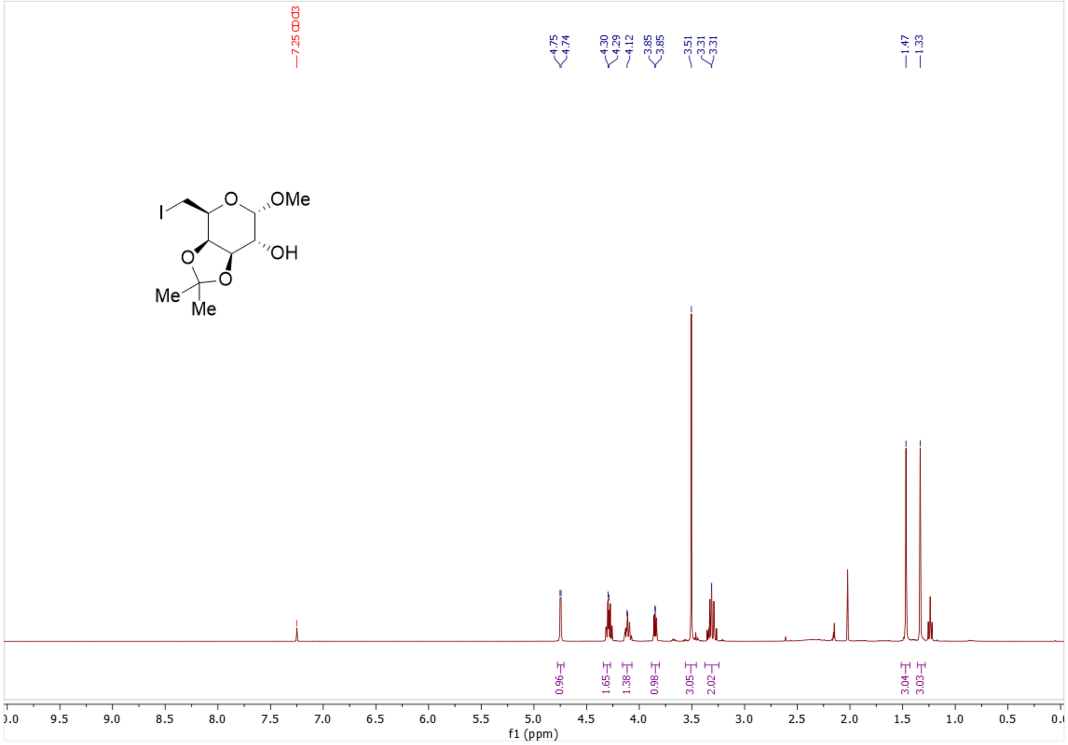

Figure S8. ^{1}H NMR (400 MHz, $CDCl_3$) spectrum of compound **4**.

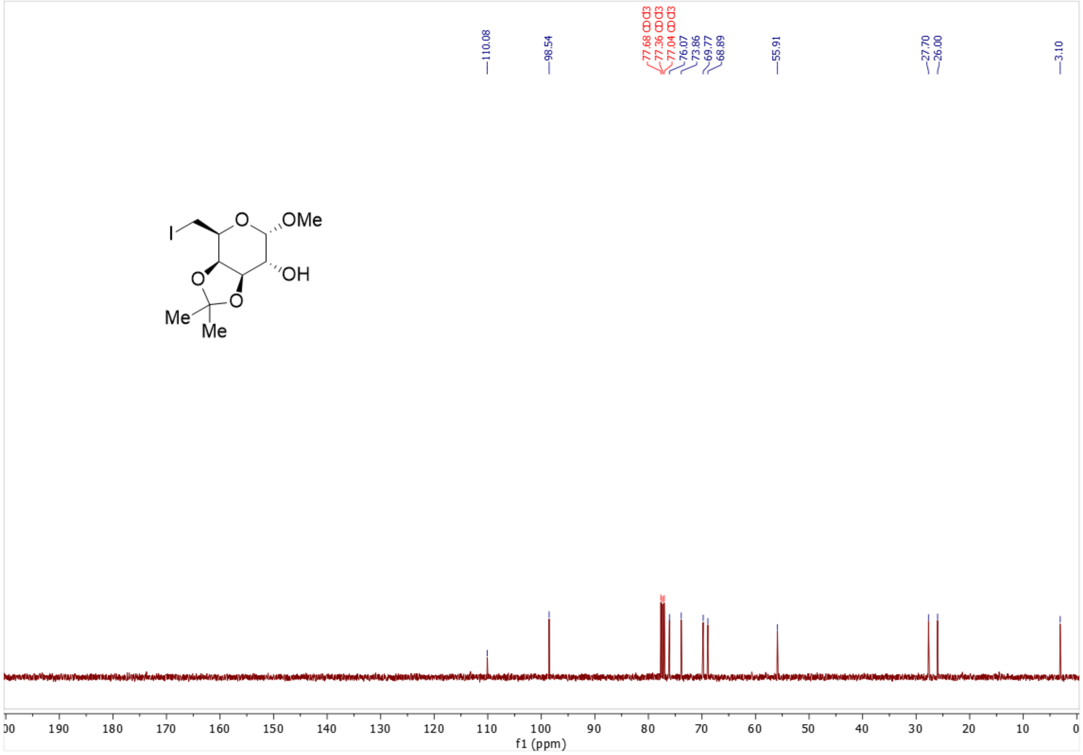

Figure S9. $^{13}C\{H\}$ NMR (101 MHz, $CDCl_3$) spectrum of compound **4**.

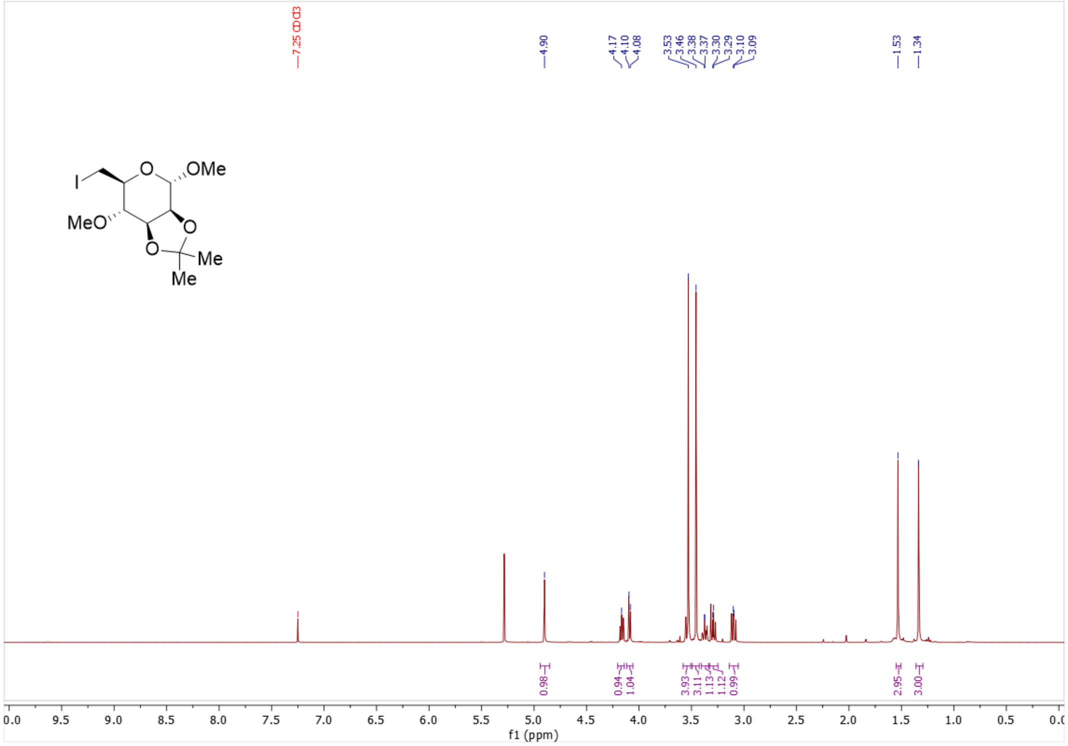

Figure S10. ^{1}H NMR (400 MHz, $CDCl_3$) spectrum of compound **5**.

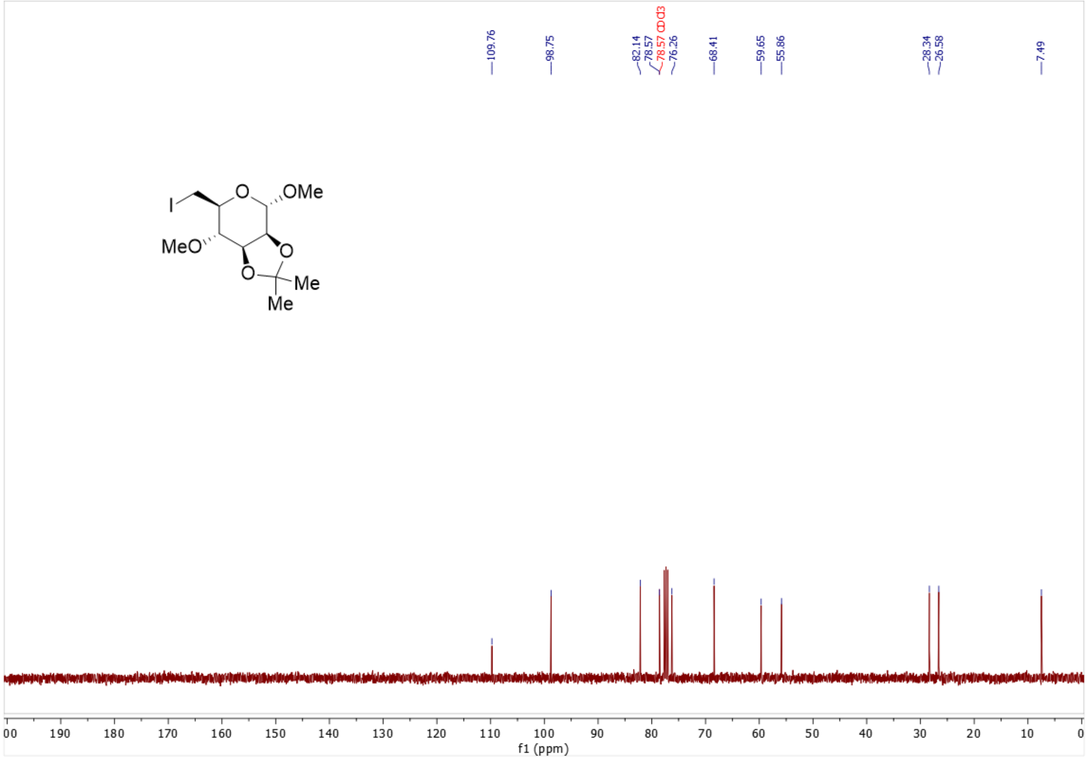

Figure S11. ^{13}C{H} NMR (101 MHz, $CDCl_3$) spectrum of compound **5**.

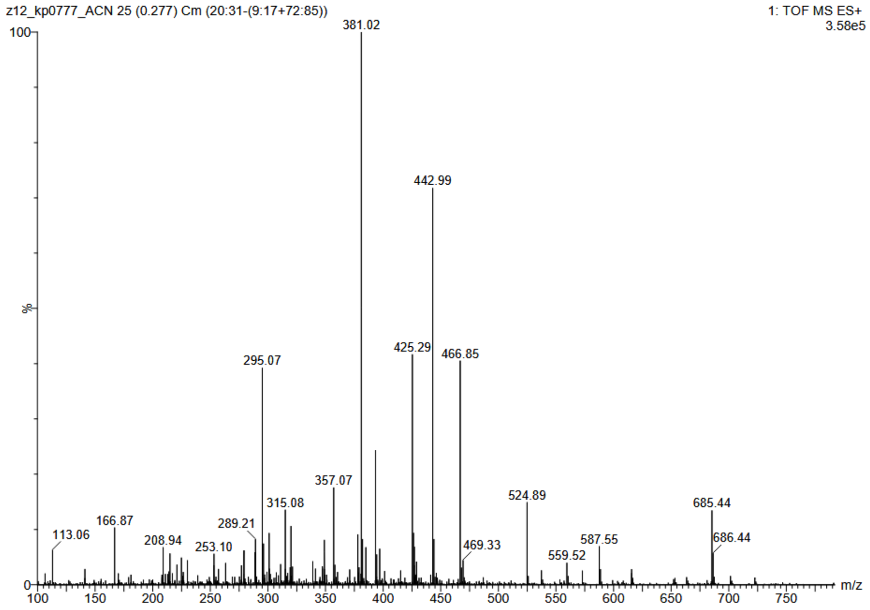

Figure S12. HR-ESI-MS spectrum of compound **5**.

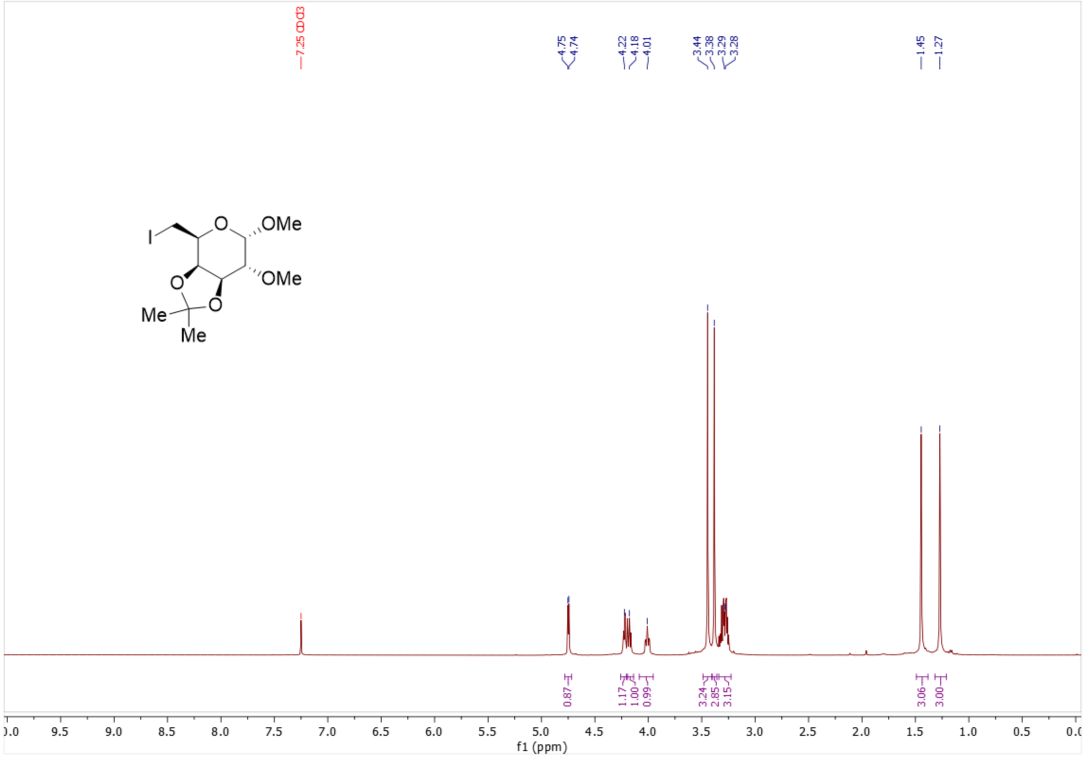

Figure S13. ^{1}H NMR (400 MHz, $CDCl_3$) spectrum of compound **7**.

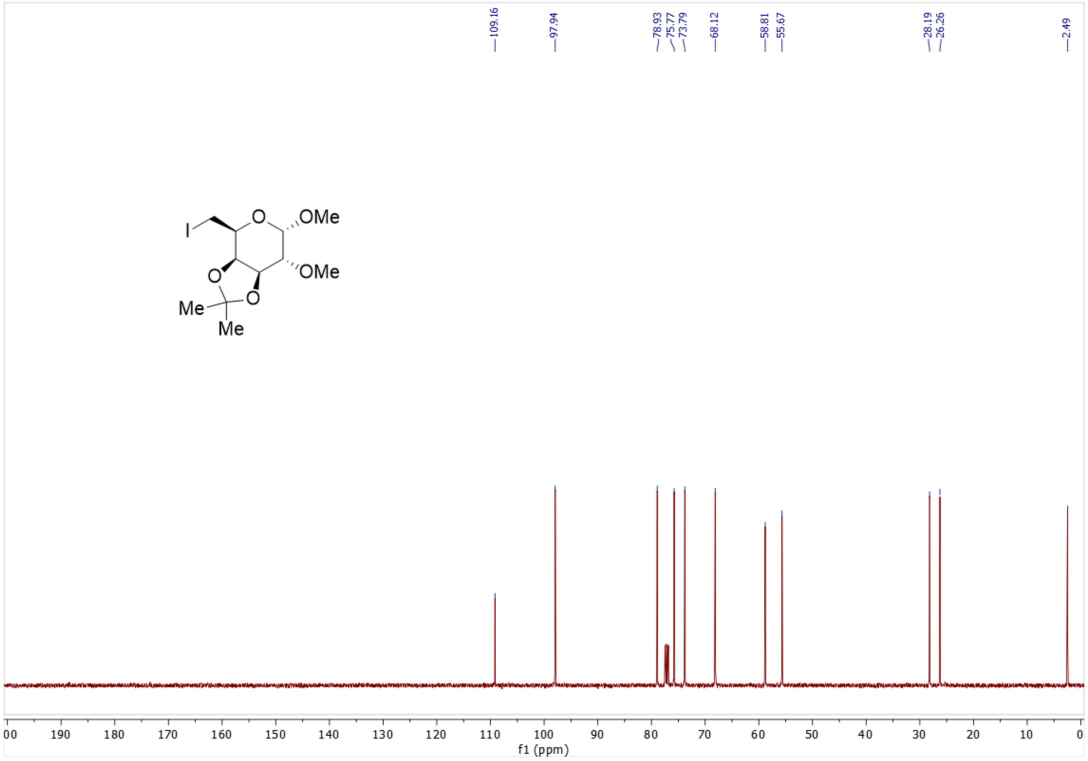

Figure S14. $^{13}C\{H\}$ NMR (101 MHz, $CDCl_3$) spectrum of compound **7**.

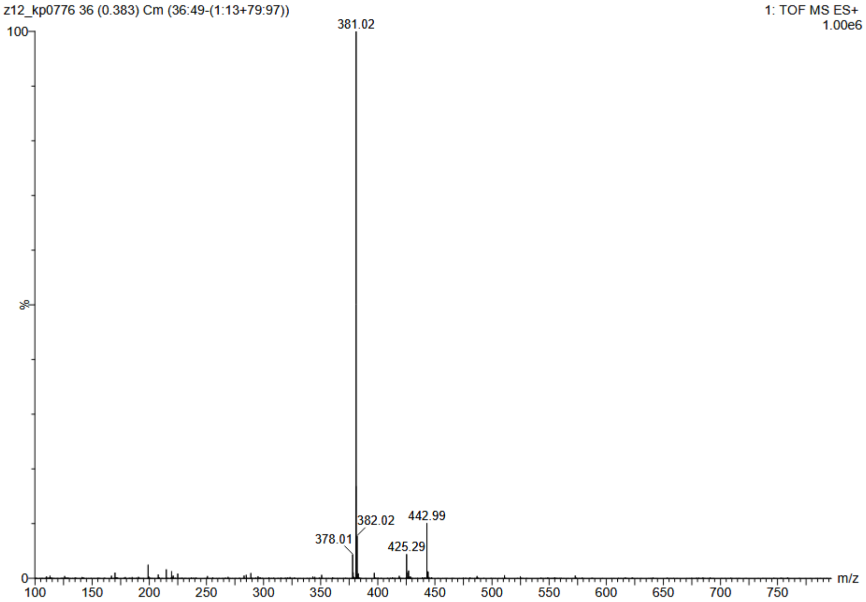

Figure S15. HR-ESI-MS spectrum of compound **7**.

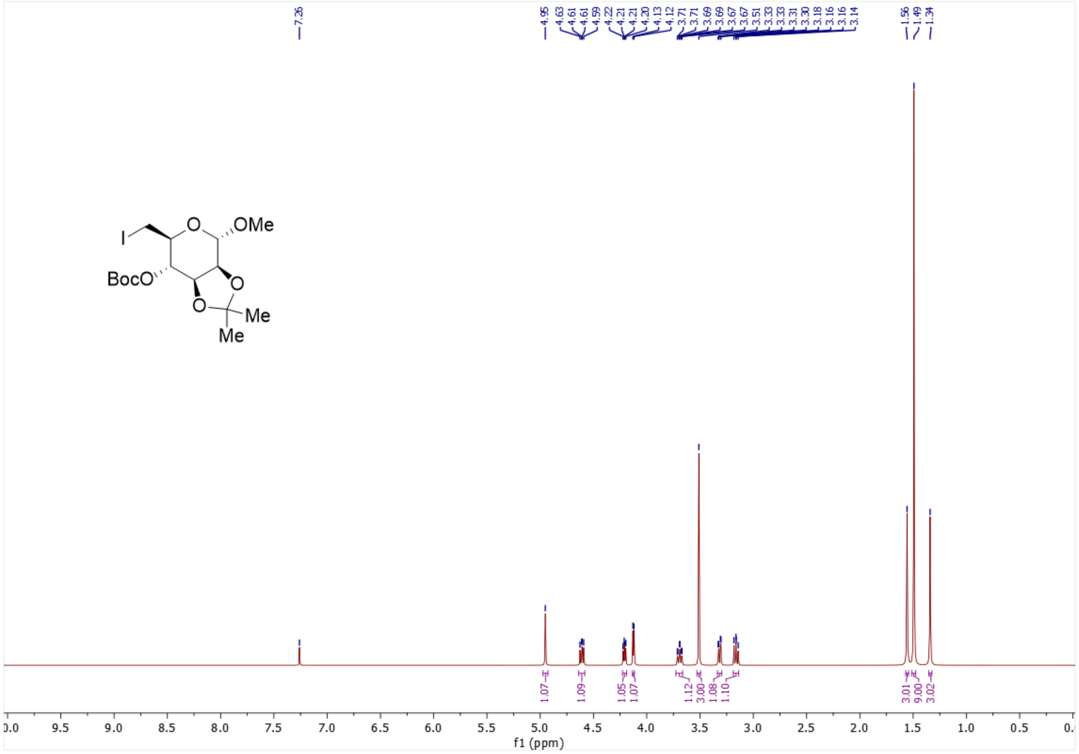

Figure S16. ^{1}H NMR (400 MHz, $CDCl_3$) spectrum of compound **6**.

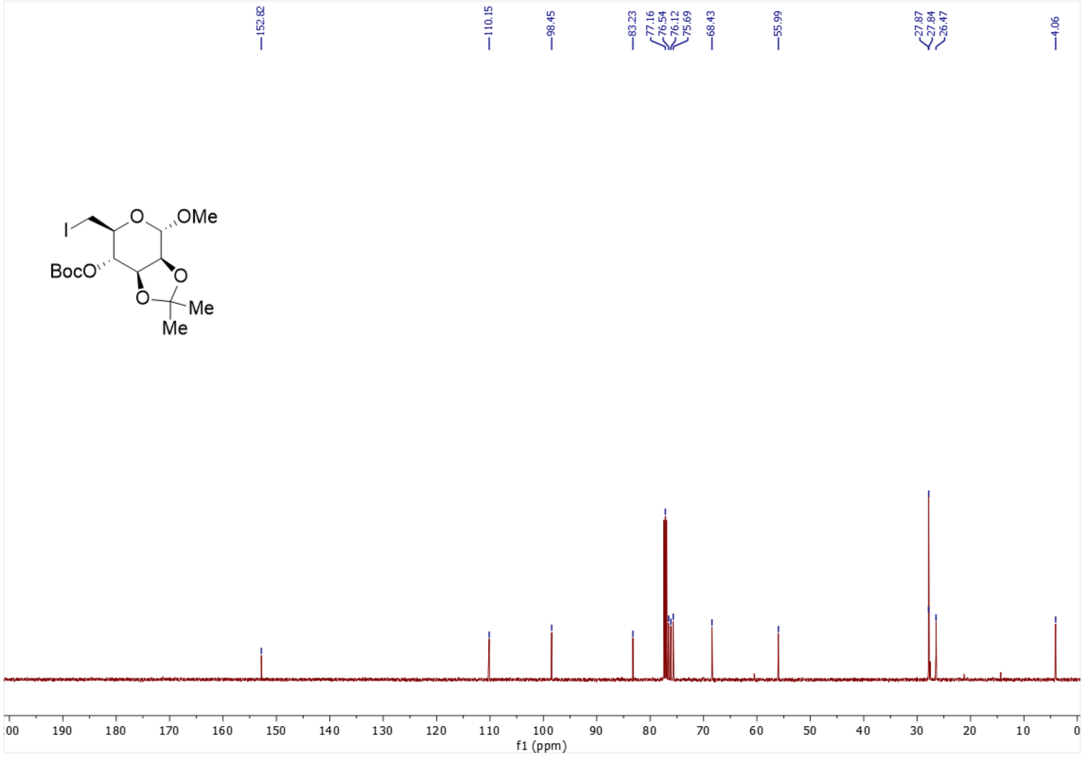

Figure S17. $^{13}C\{H\}$ NMR (101 MHz, $CDCl_3$) spectrum of compound **6**.

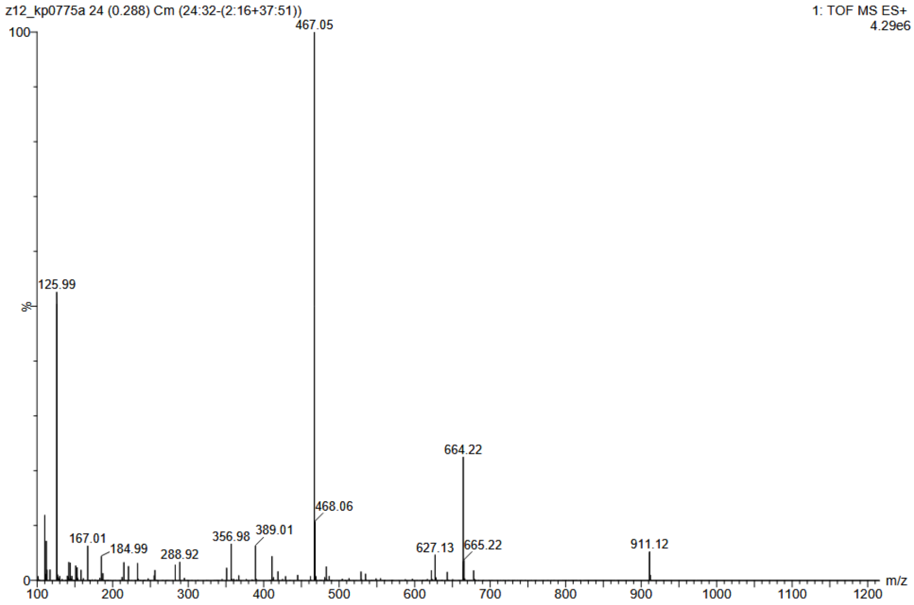

Figure S18. LR-ESI-MS spectrum of compound **6**.

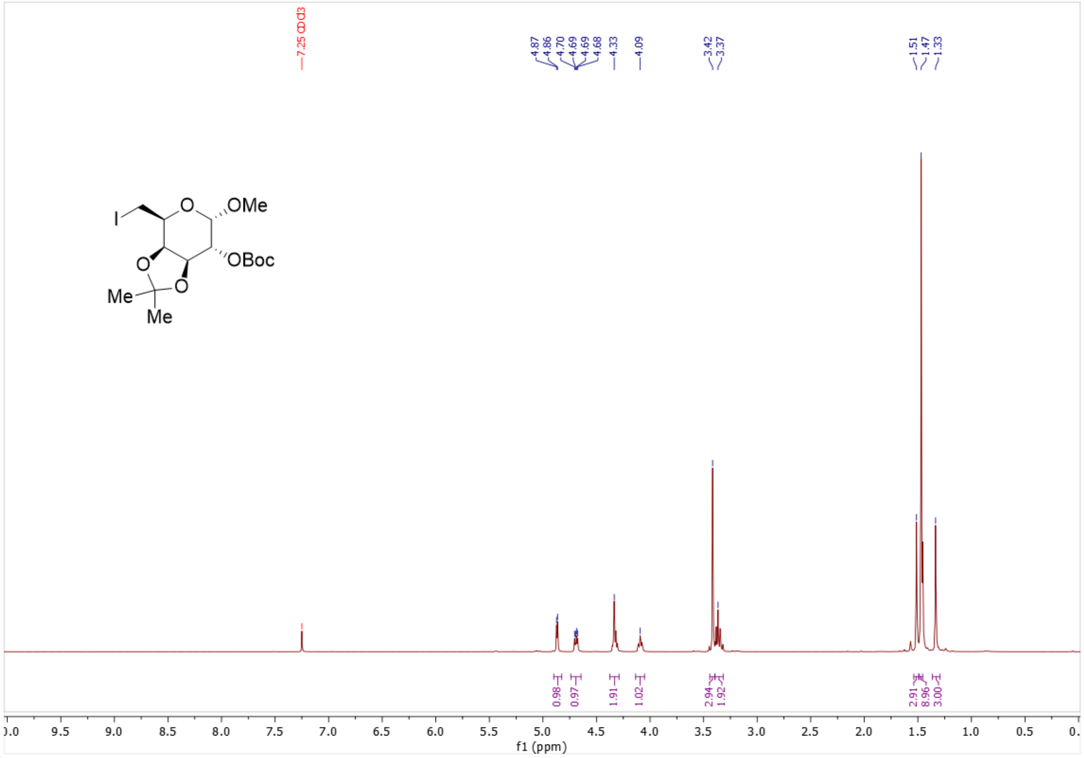

Figure S19. ^{1}H NMR (400 MHz, $CDCl_3$) spectrum of compound **8**.

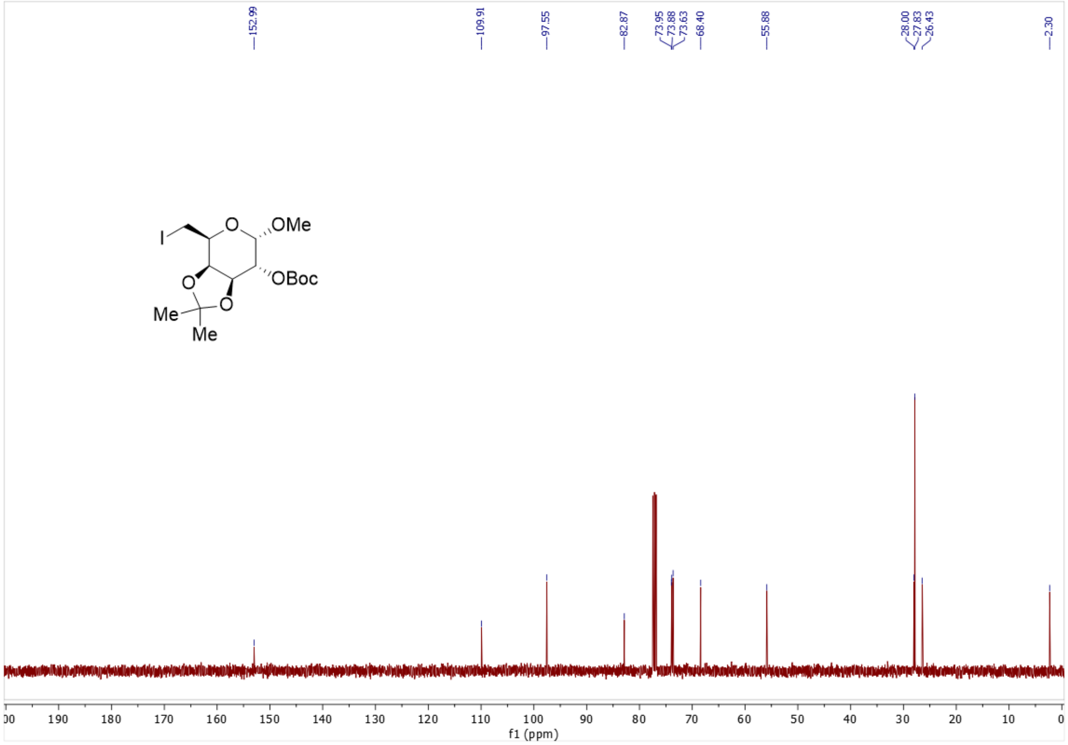

Figure S20. $^{13}C\{H\}$ NMR (101 MHz, $CDCl_3$) spectrum of compound **8**.

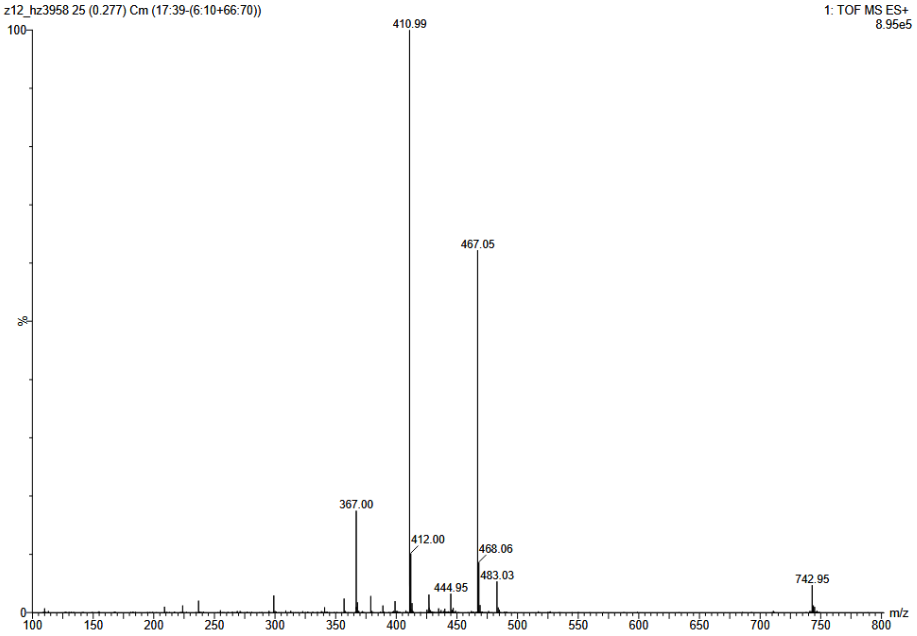

Figure S21. HR-ESI-MS spectrum of compound **8**.

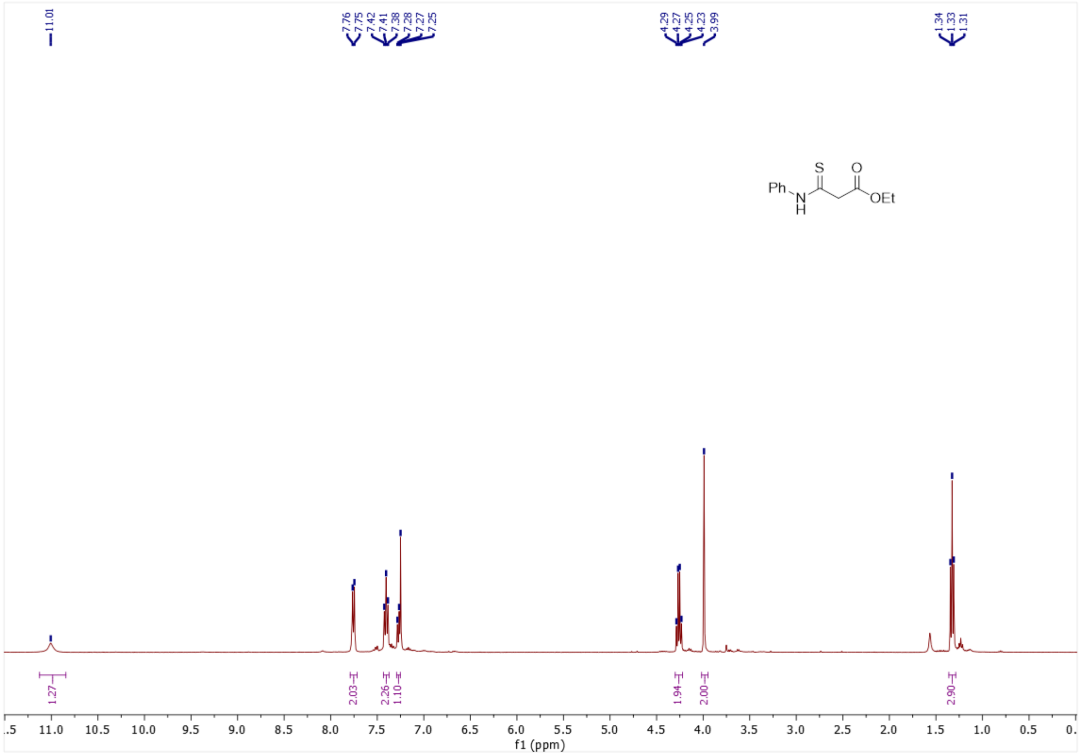

Figure S22. ^{1}H NMR (400 MHz, $CDCl_3$) spectrum of compound **13**.

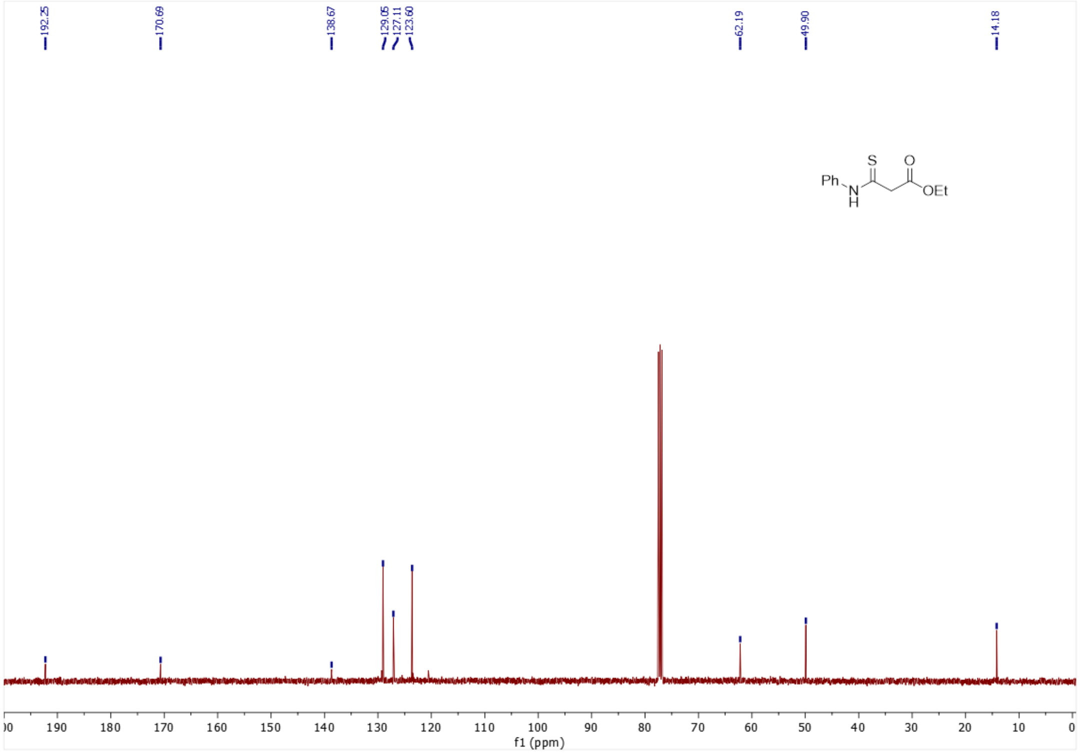

Figure S23. ^{13}C{H} NMR (101 MHz, $CDCl_3$) spectrum of compound **13**.

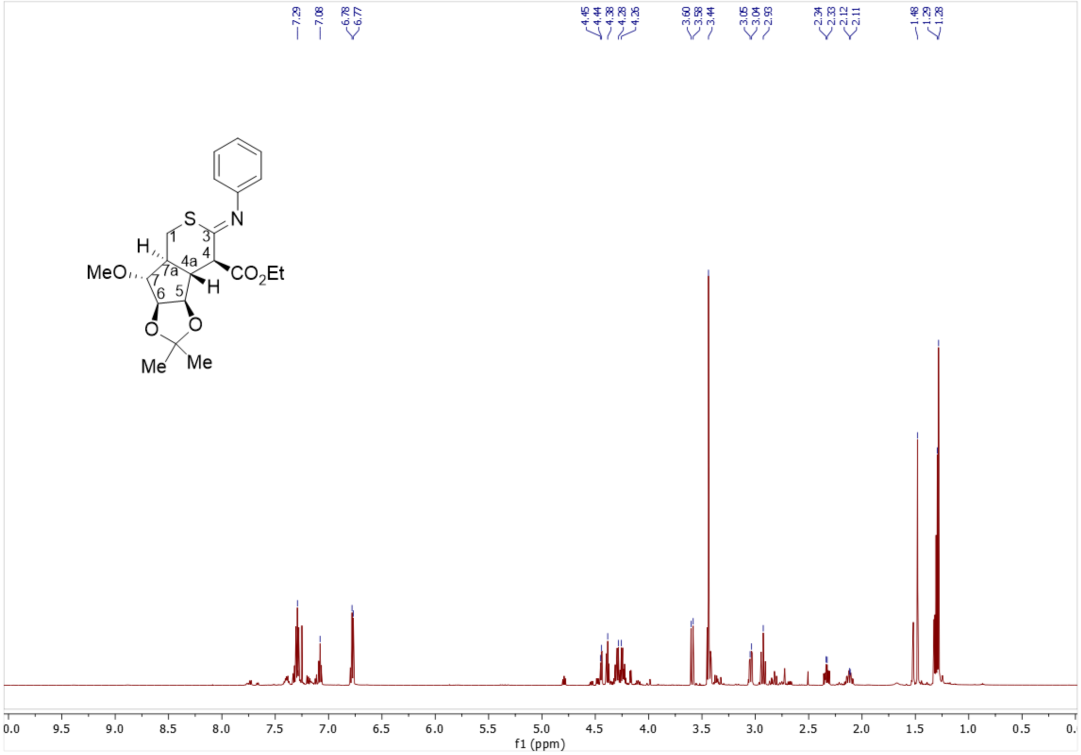

Figure S24. ^{1}H NMR (400 MHz, $CDCl_3$) spectrum of compound **23**.

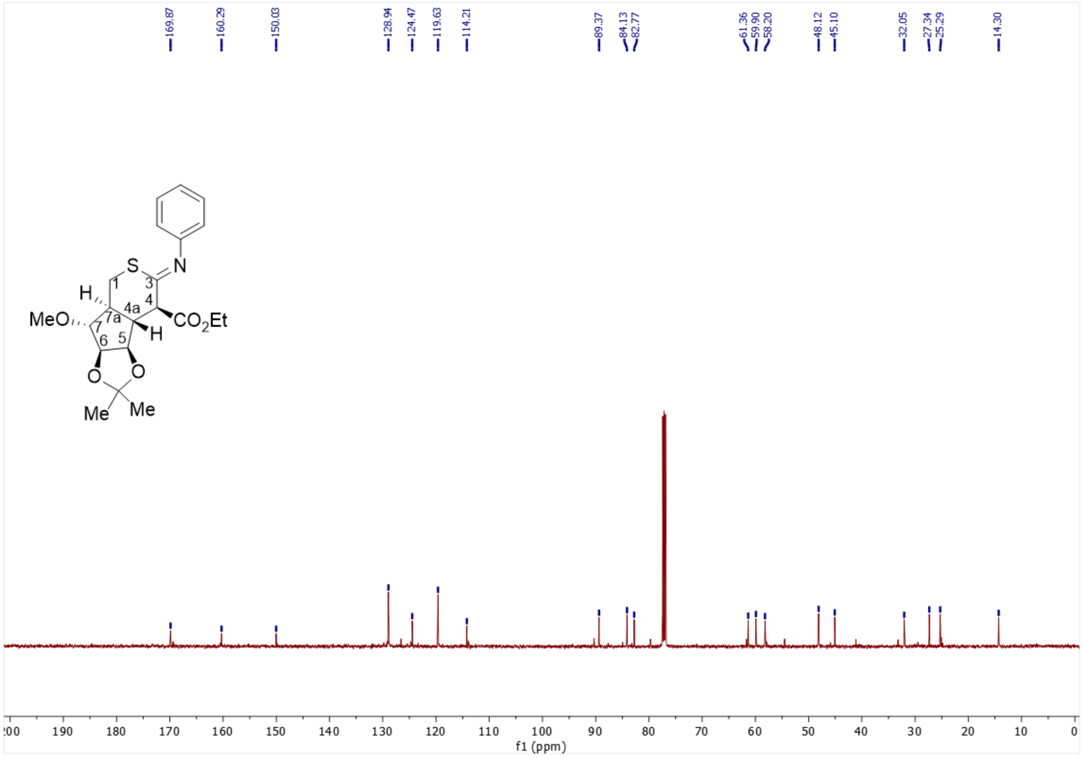

Figure S25. ^{13}C{H} NMR (101 MHz, $CDCl_3$) spectrum of compound **23**.

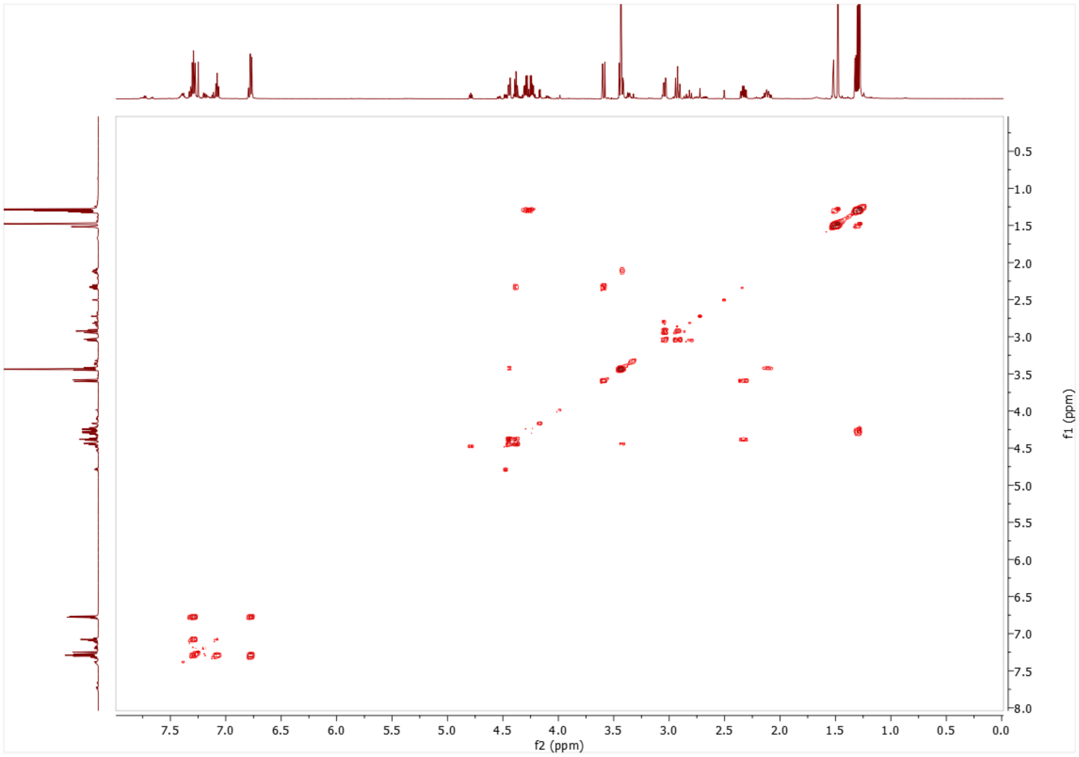

Figure S26. ^1H-^1H COSY NMR (600 MHz spectrometer, $CDCl_3$) spectrum of compound **23**.

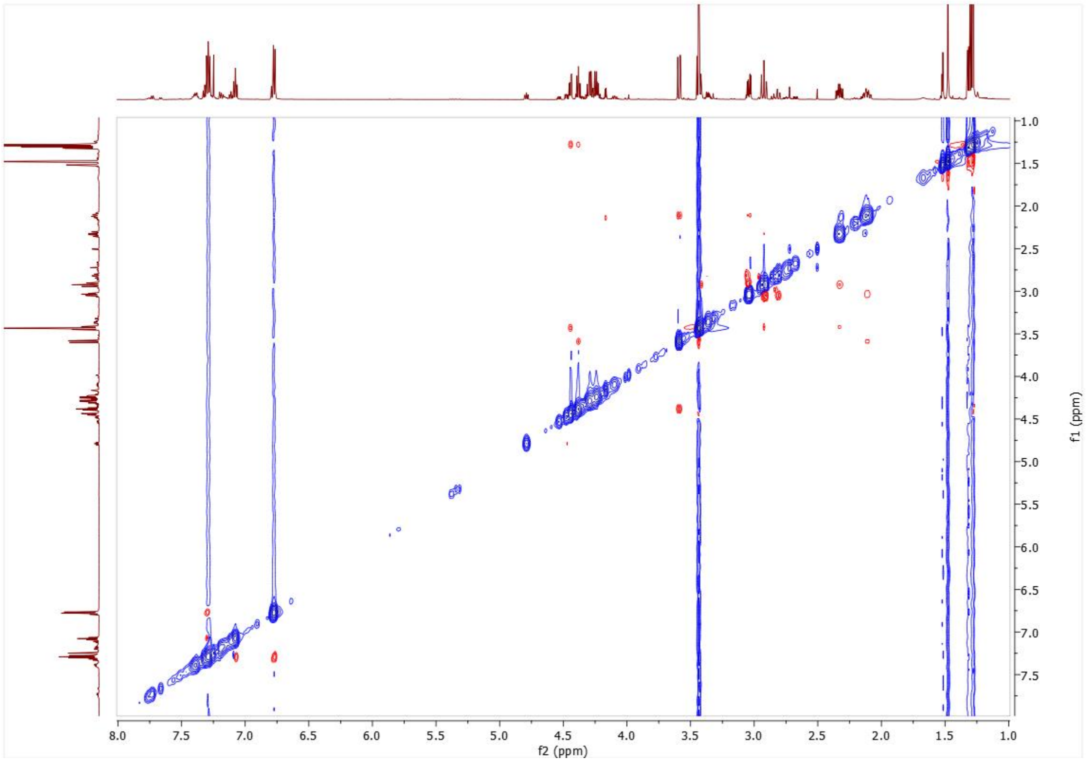

Figure S27. ^1H-^1H NOESY NMR (600 MHz spectrometer, $CDCl_3$) spectrum of compound **23**.

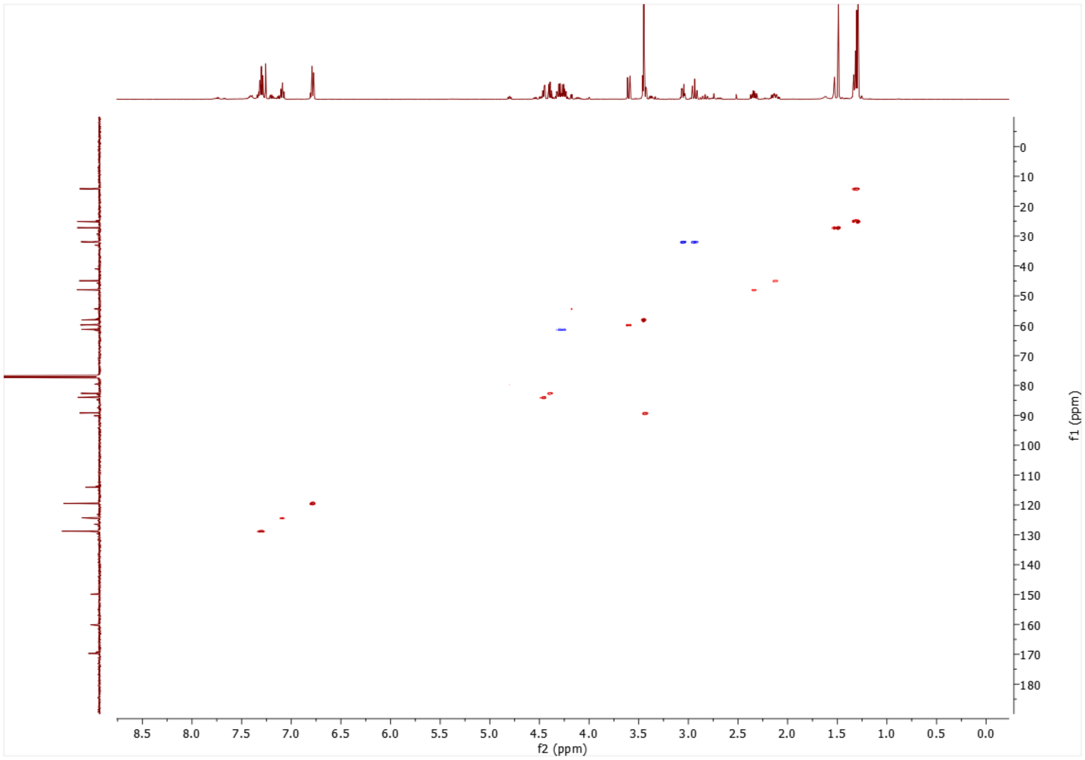

Figure S28. ^{1}H-^{13}C HSQC NMR (600 MHz spectrometer, $CDCl_3$) spectrum of compound **23**.

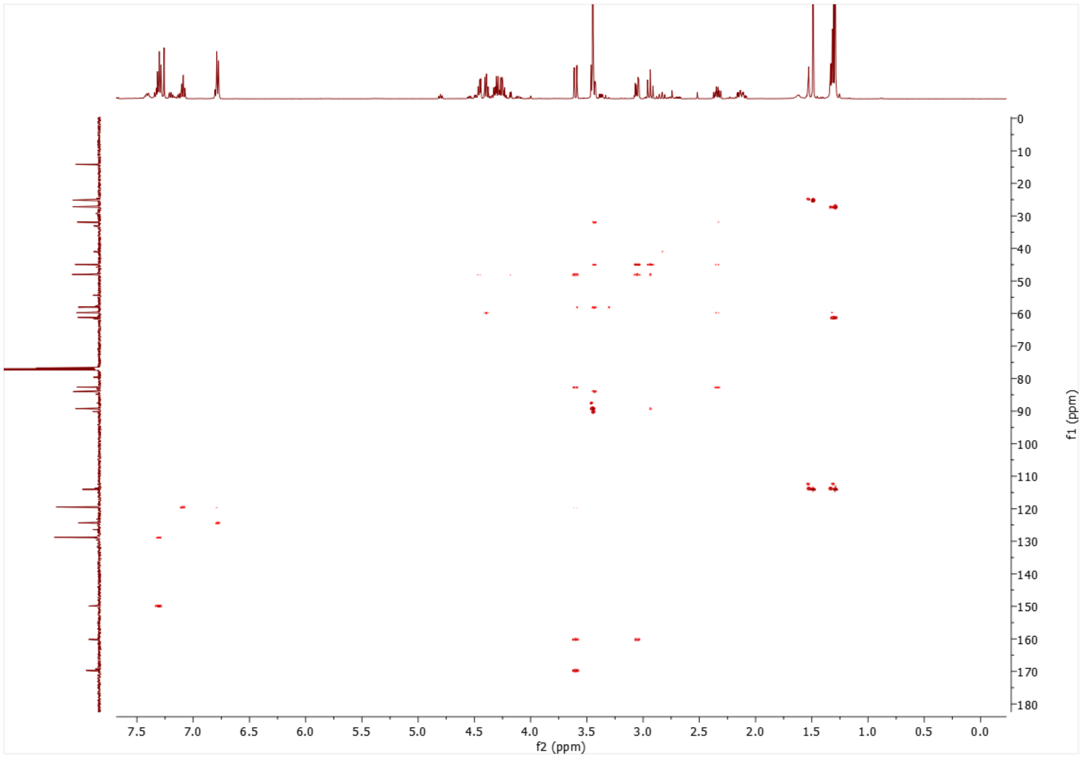

Figure S29. ^{1}H-^{13}C HMBC NMR (600 MHz spectrometer, $CDCl_3$) spectrum of compound **23**.

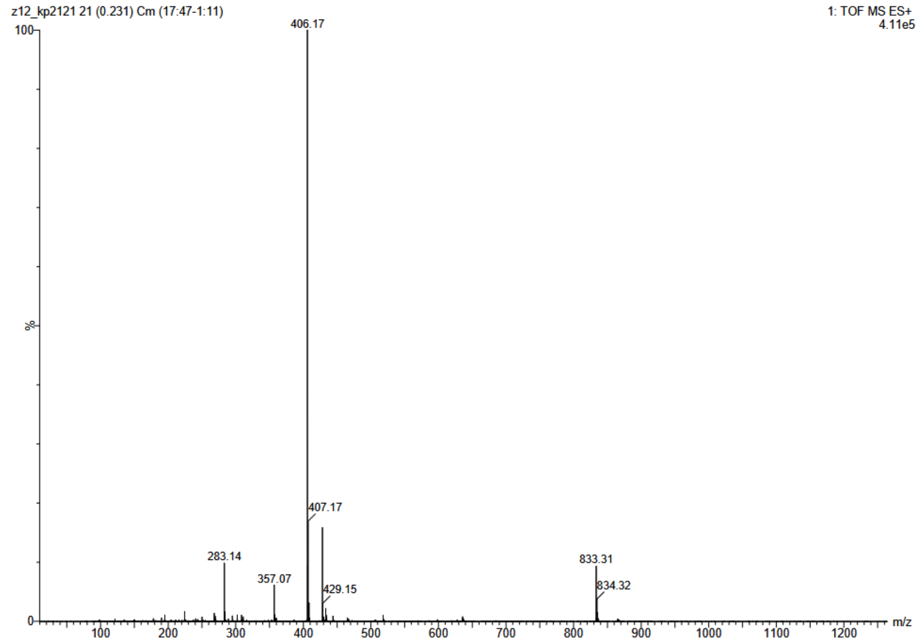

Figure S30. HR-ESI-MS spectrum of compound **23**.

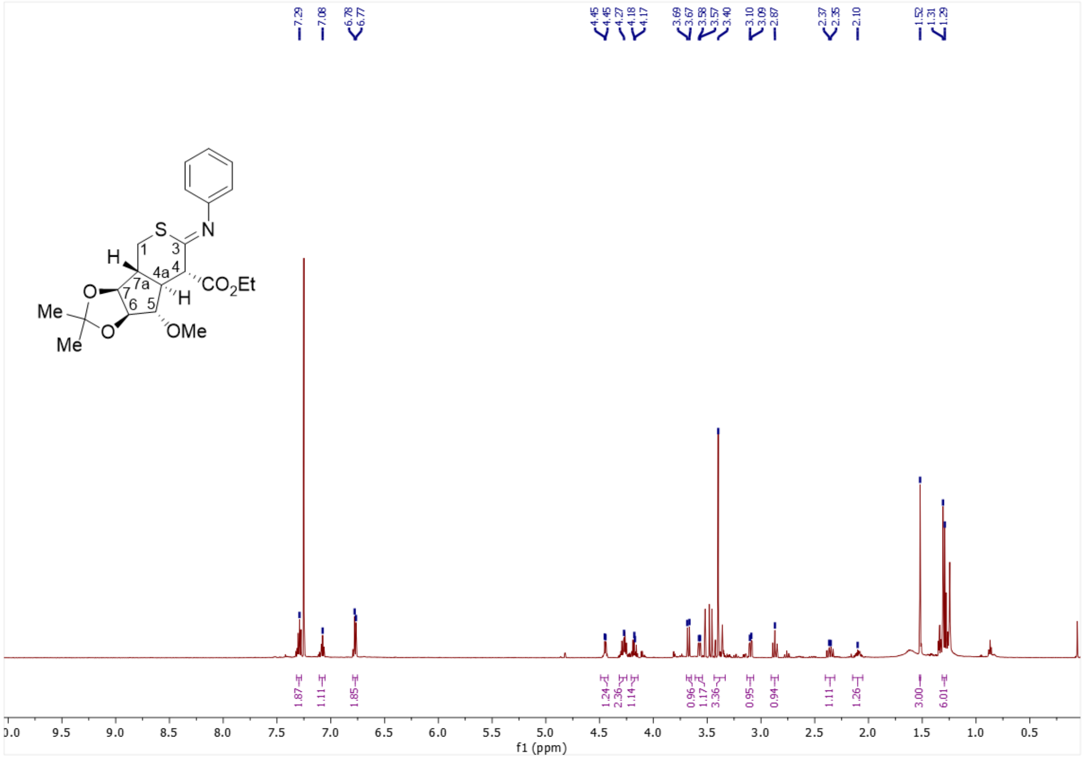

Figure S31. ^{1}H NMR (600 MHz, $CDCl_3$) spectrum of compound **27**.

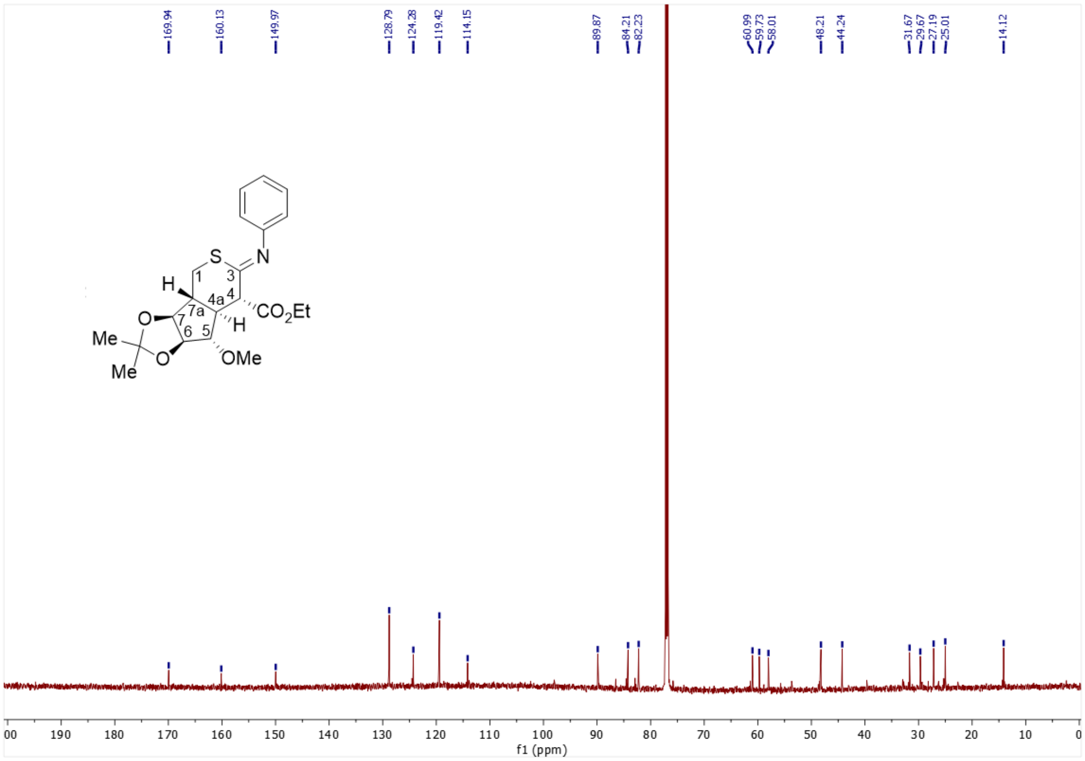

Figure S32. $^{13}C\{H\}$ NMR (151 MHz, $CDCl_3$) spectrum of compound **27**.

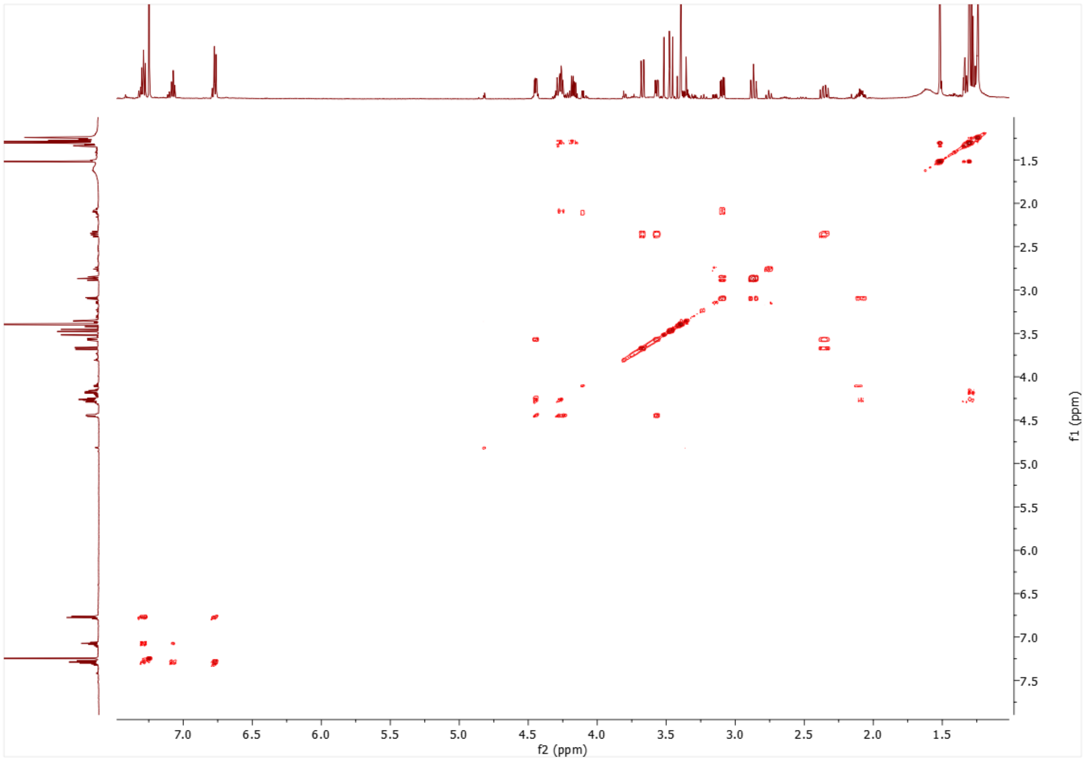

Figure S33. ^{1}H-^{1}H COSY NMR (600 MHz spectrometer, $CDCl_3$) spectrum of compound **27**.

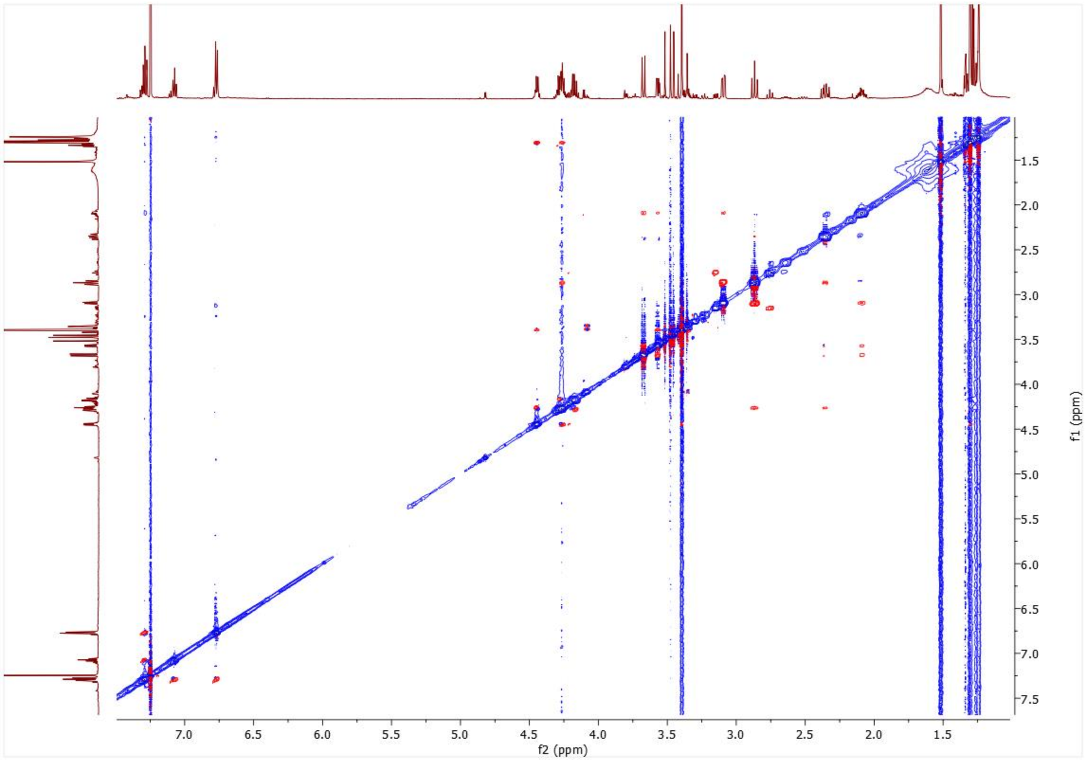

Figure S34. ^{1}H-^{1}H NOESY NMR (600 MHz spectrometer, $CDCl_3$) spectrum of compound **27**.

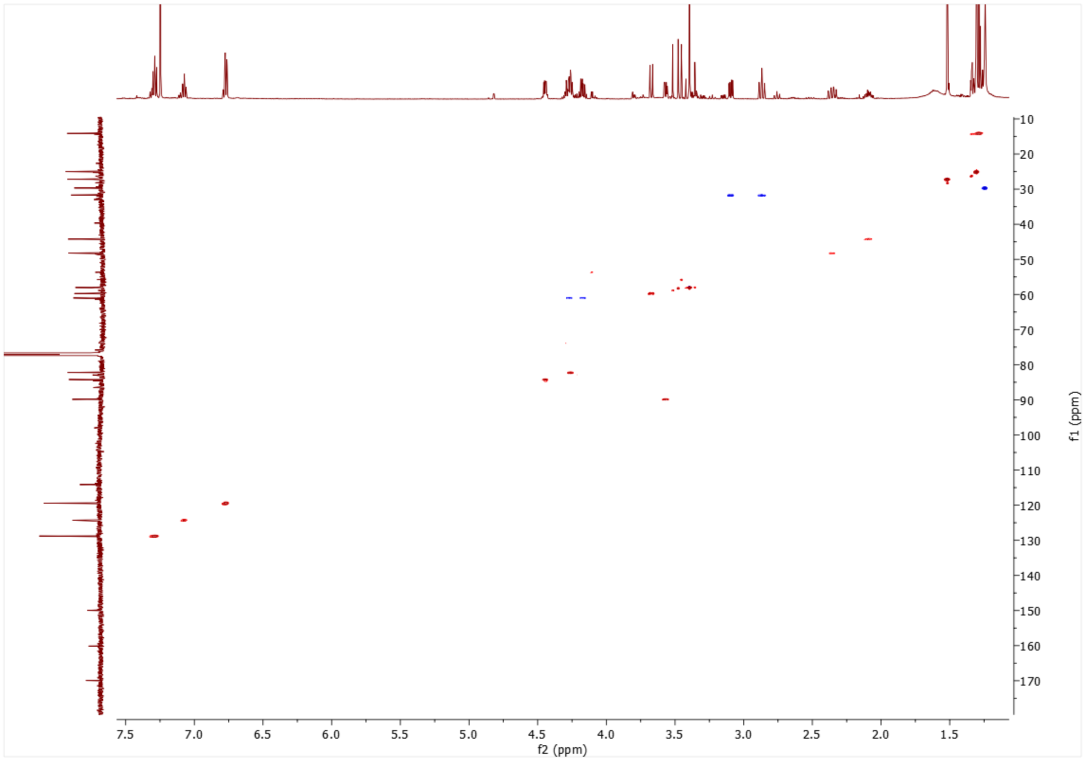

Figure S35. ^{1}H-^{13}C HSQC NMR (600 MHz spectrometer, $CDCl_3$) spectrum of compound **27**.

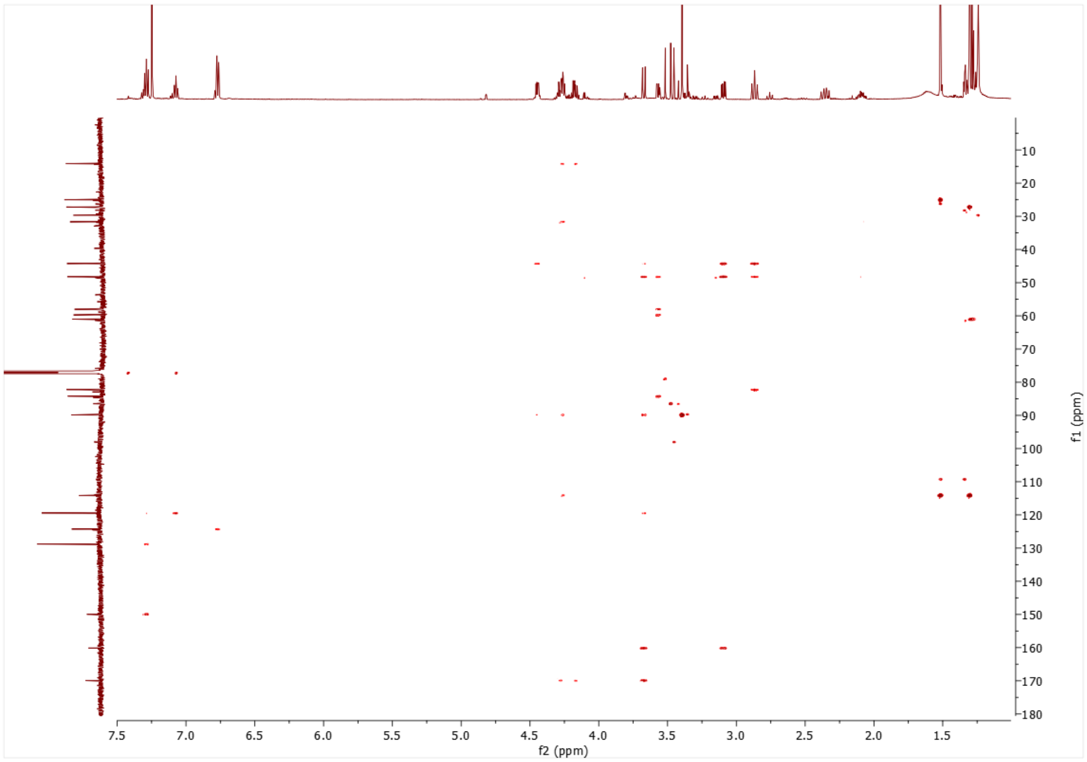

Figure S36. ^{1}H-^{13}C HMBC NMR (600 MHz spectrometer, $CDCl_3$) spectrum of compound **27**.

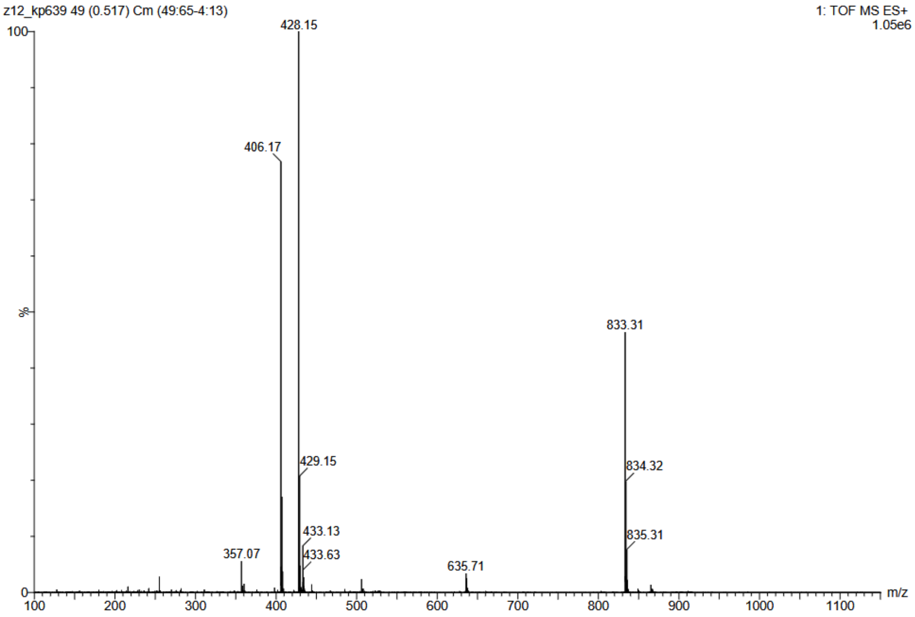

Figure S37. HR-ESI-MS spectrum of compound **27**.

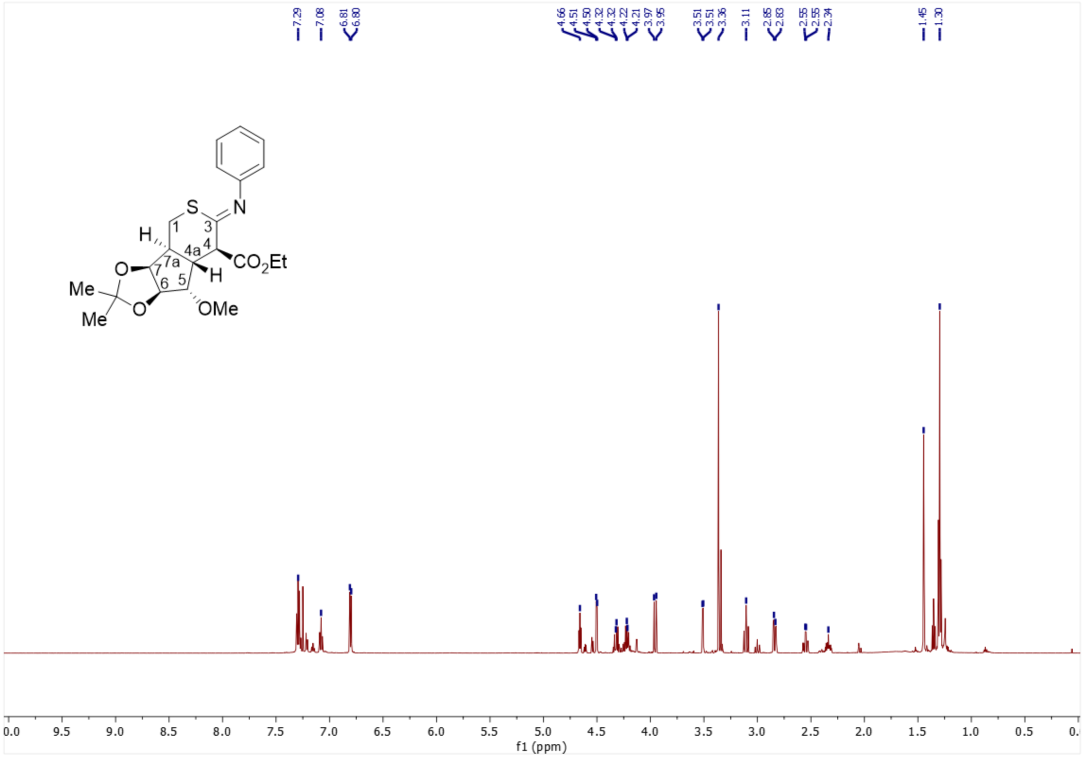

Figure S38. ^{1}H NMR (600 MHz, $CDCl_3$) spectrum of compound **25**.

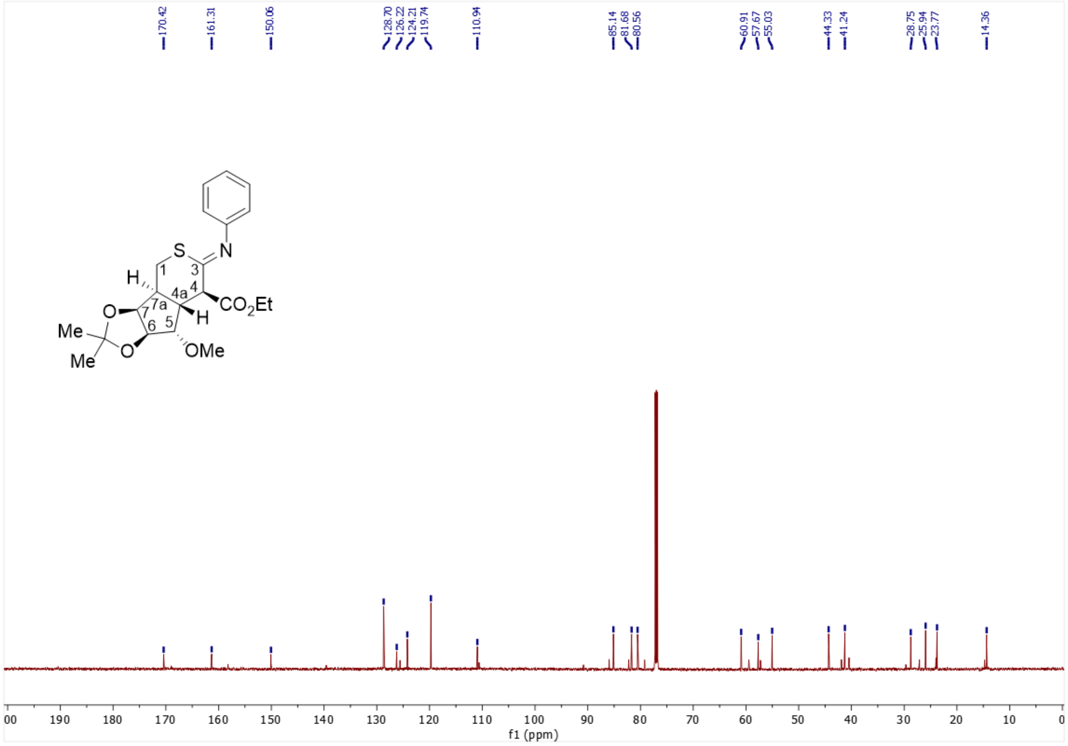

Figure S39. $^{13}C\{H\}$ NMR (151 MHz, $CDCl_3$) spectrum of compound **25**.

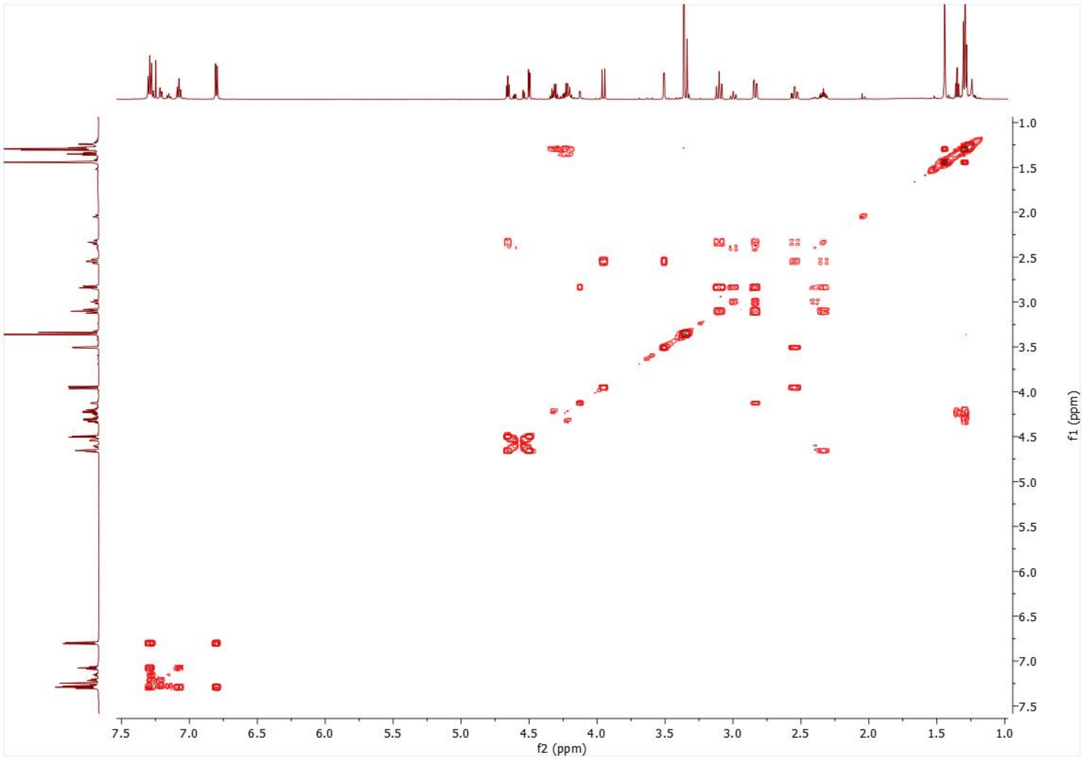

Figure S40. ^1H-^1H COSY NMR (600 MHz spectrometer, $CDCl_3$) spectrum of compound **25**.

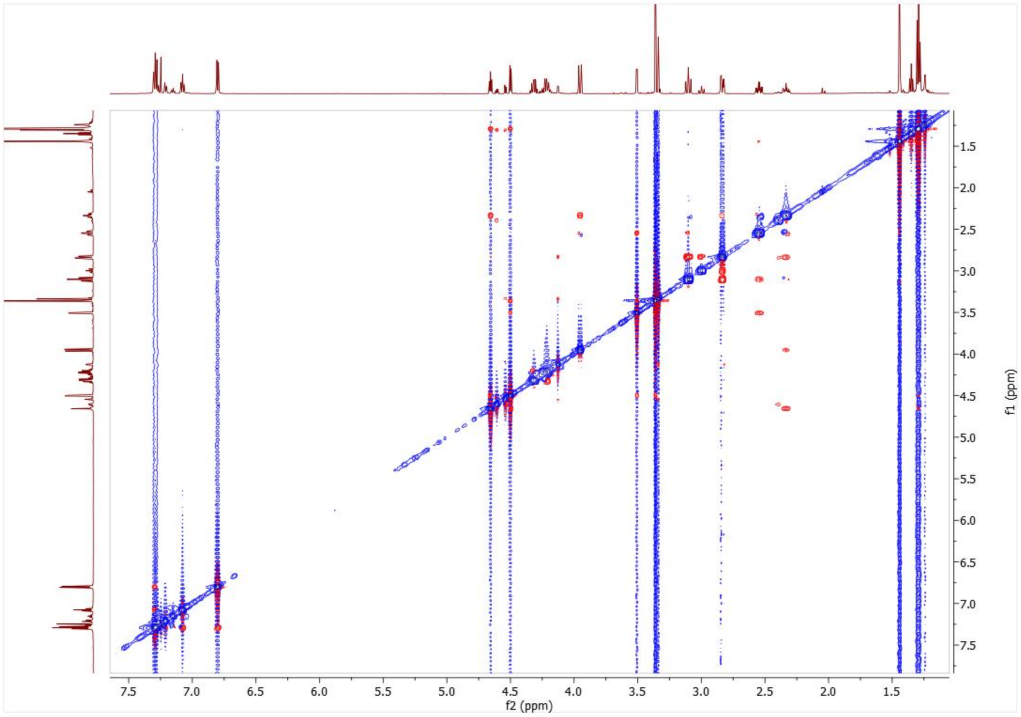

Figure S41. ^1H-^1H NOESY NMR (600 MHz spectrometer, $CDCl_3$) spectrum of compound **25**.

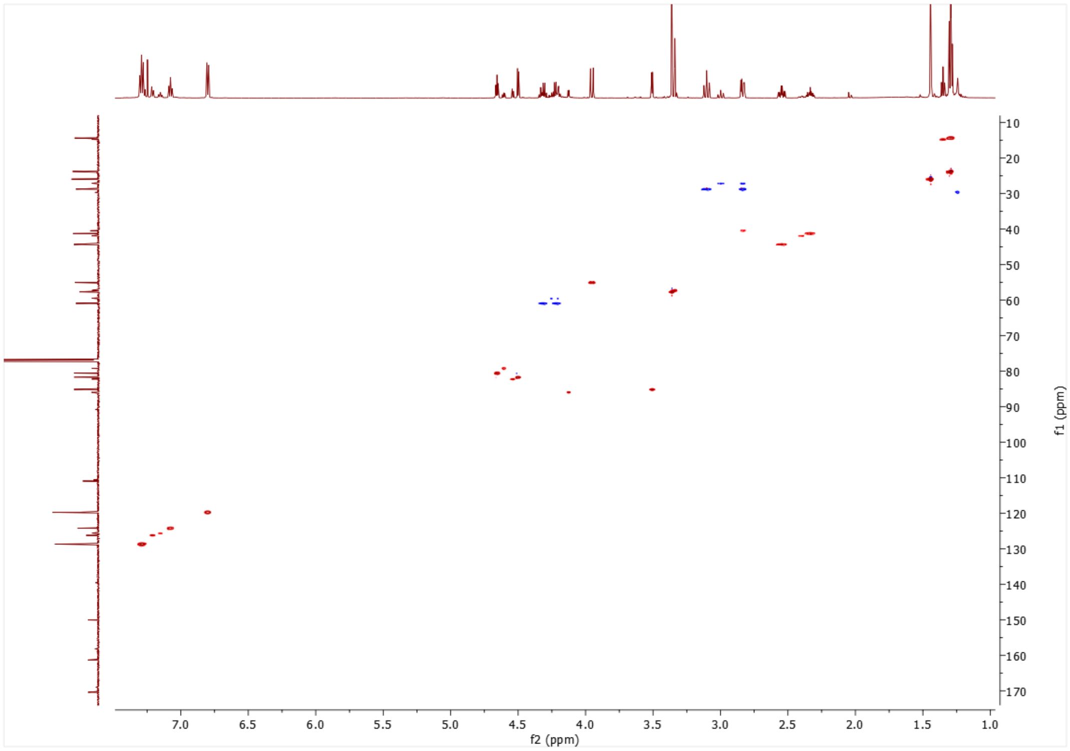

Figure S42. ^{1}H-^{13}C HSQC NMR (600 MHz spectrometer, $CDCl_3$) spectrum of compound **25**.

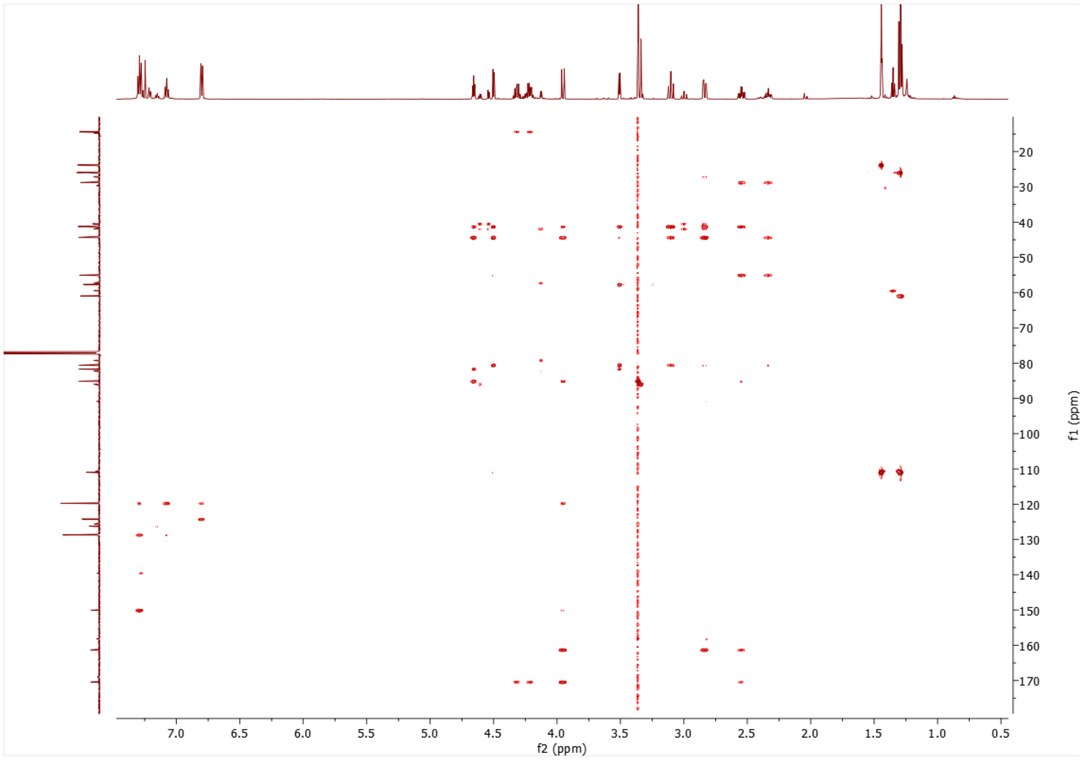

Figure S43. ^{1}H-^{13}C HMBC NMR (600 MHz spectrometer, $CDCl_3$) spectrum of compound **25**.

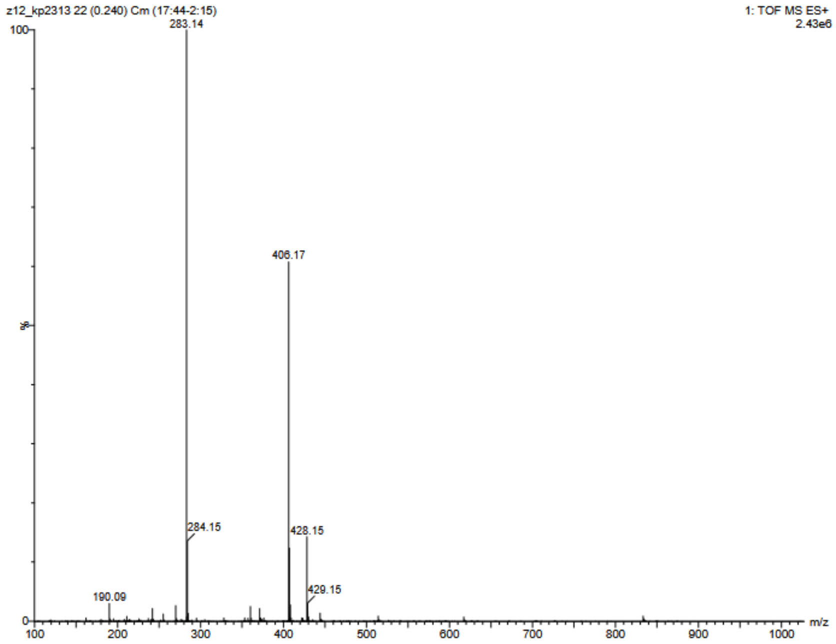

Figure S44. HR-ESI-MS spectrum of compound **25**.

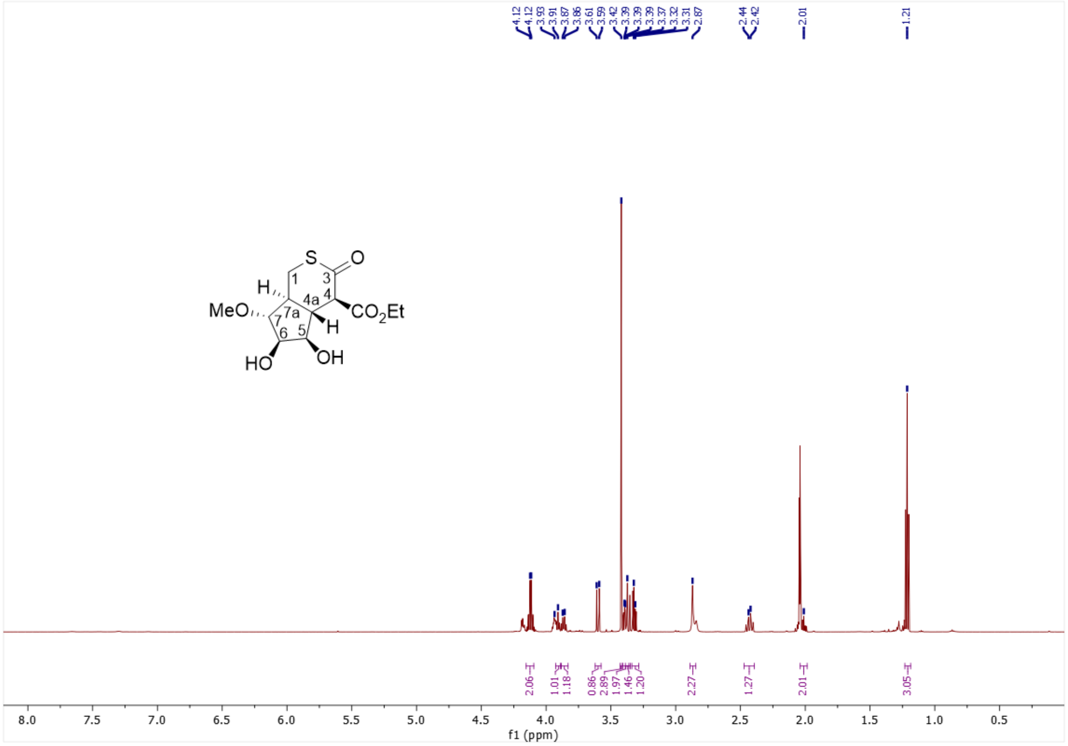

Figure S45. ^{1}H NMR (600 MHz, Acetone-d_6) spectrum of compound **28**.

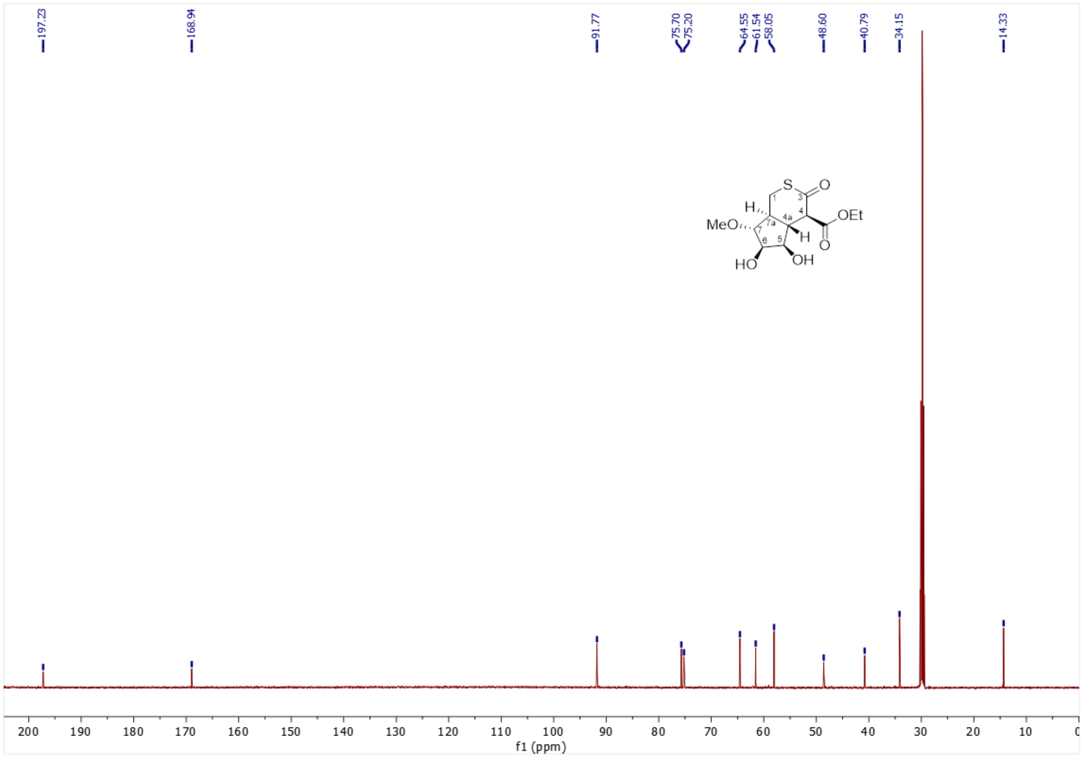

Figure S46. ^{13}C{H} NMR (151 MHz, Acetone-d_6) spectrum of compound **28**.

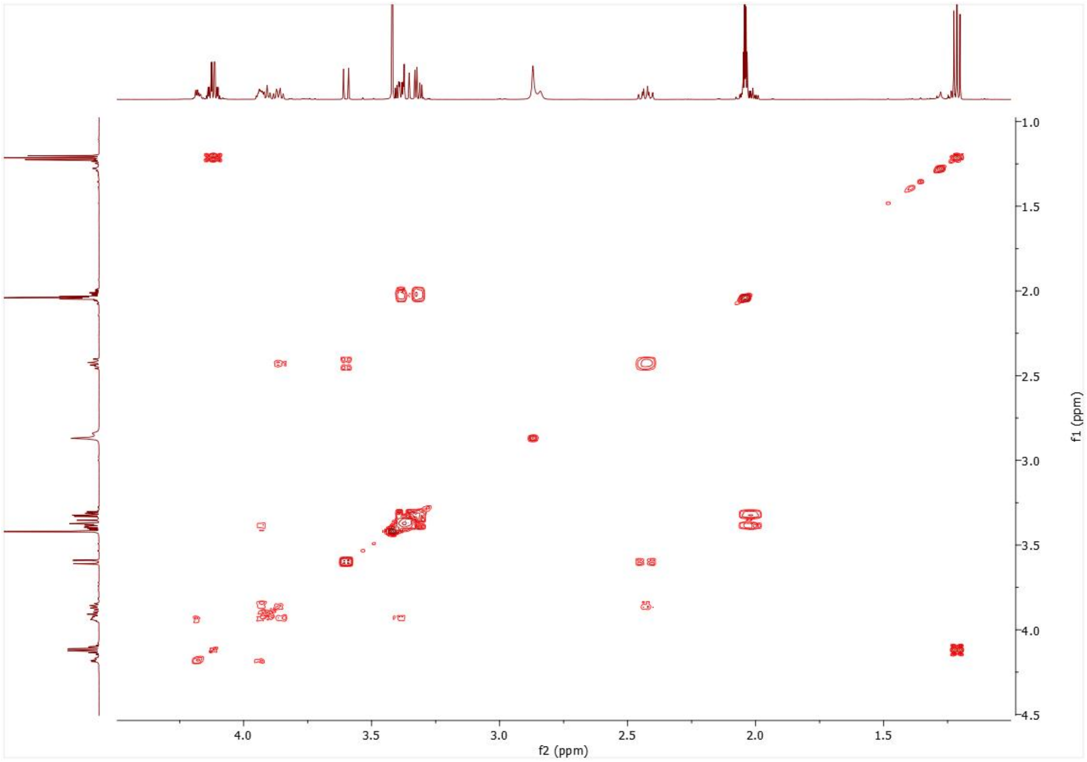

Figure S47. ^{1}H-^{1}H COSY NMR (600 MHz spectrometer, Acetone-d_6) spectrum of compound **28**.

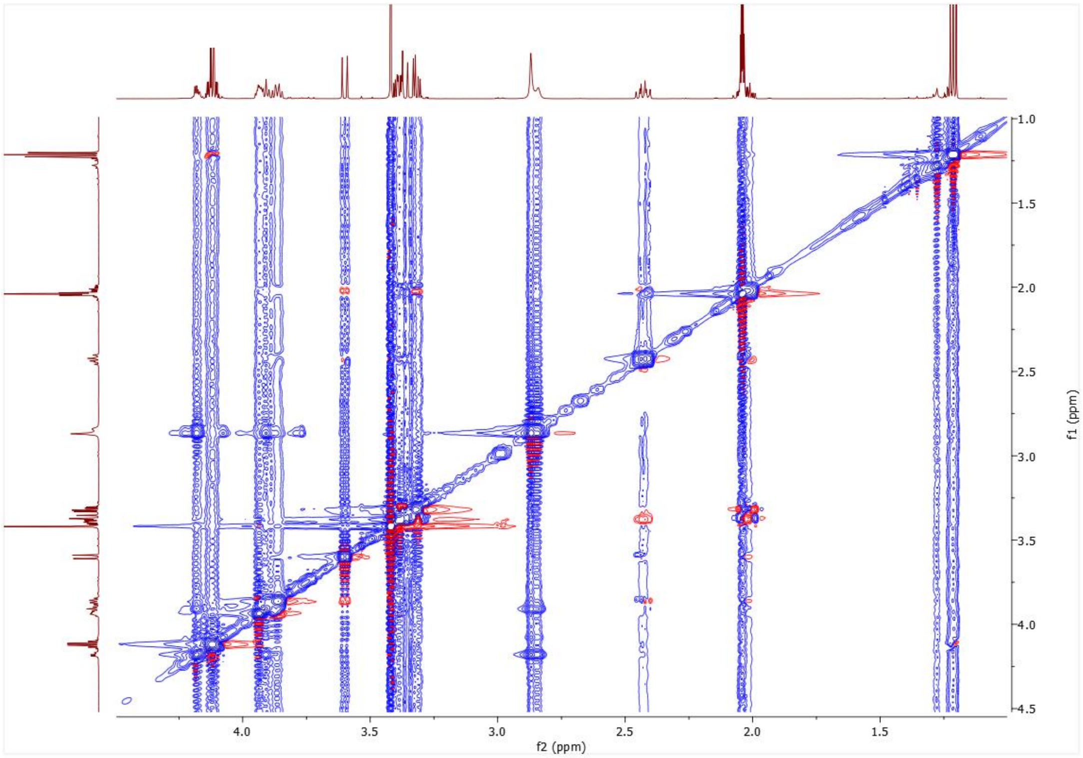

Figure S48. ^{1}H-^{1}H NOESY NMR (600 MHz spectrometer, Acetone-d_6) spectrum of compound **28**.

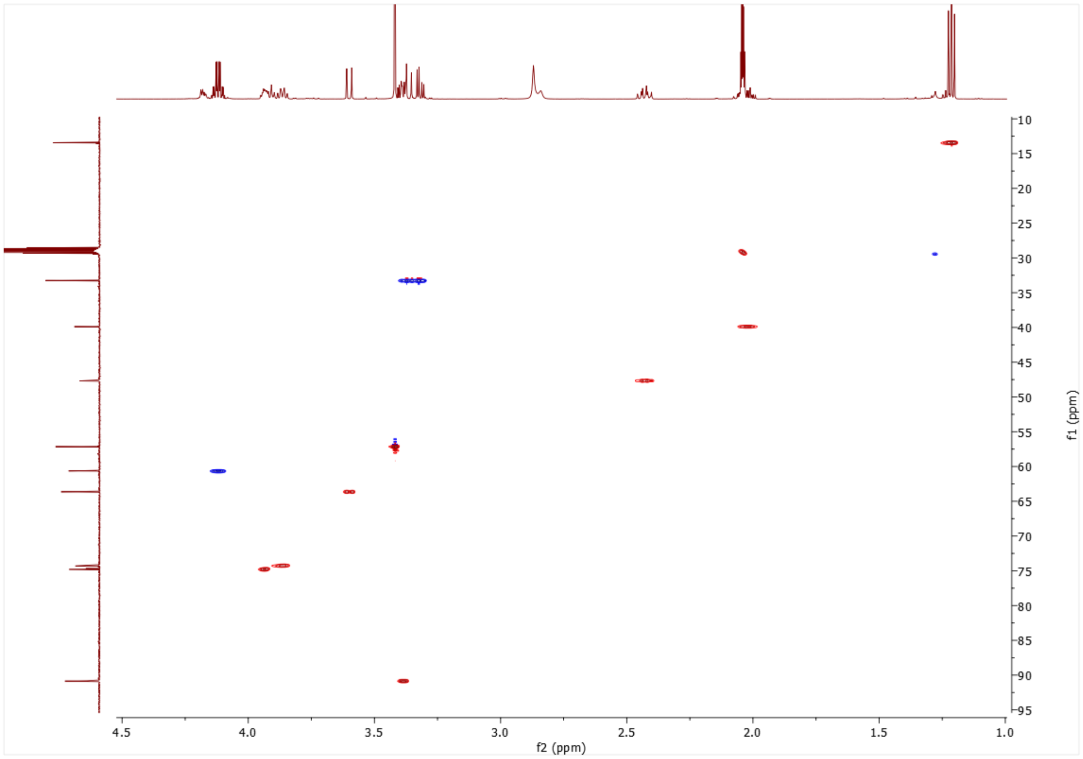

Figure S49. ^{1}H-^{13}C HSQC NMR (600 MHz spectrometer, Acetone-d_6) spectrum of compound **28**.

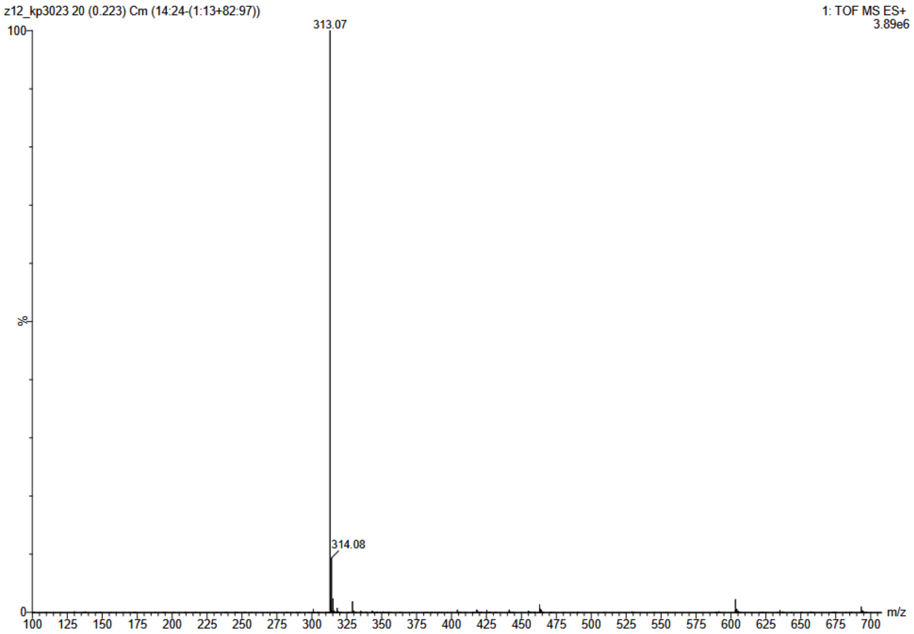

Figure S50. LR-ESI-MS spectrum of compound **28**.

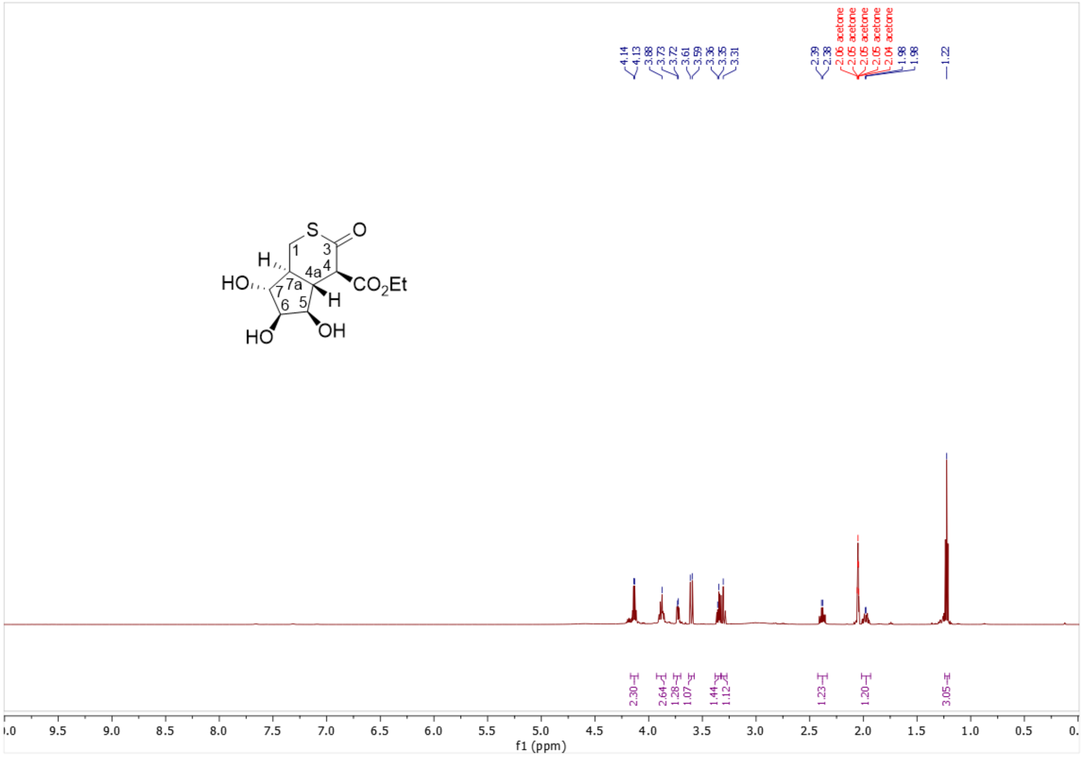

Figure S51. ^{1}H NMR (400 MHz, Acetone-d_6) spectrum of compound **29**.

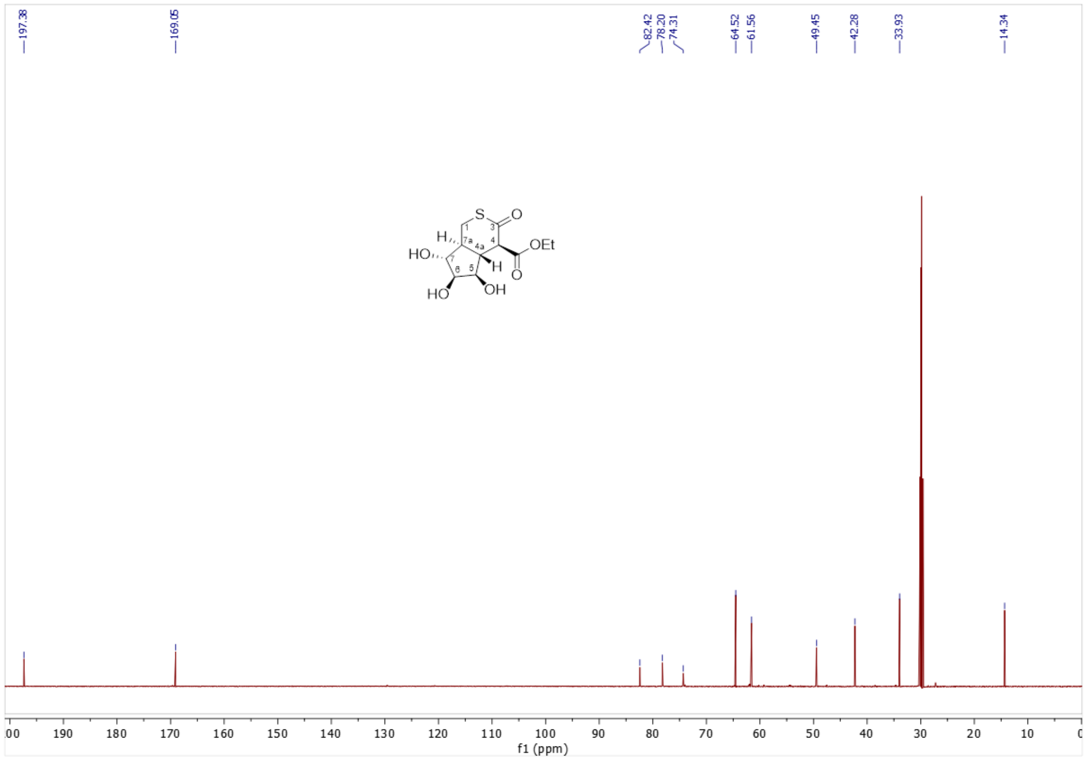

Figure S52. $^{13}C\{H\}$ NMR (101 MHz, Acetone-d_6) spectrum of compound **29**.

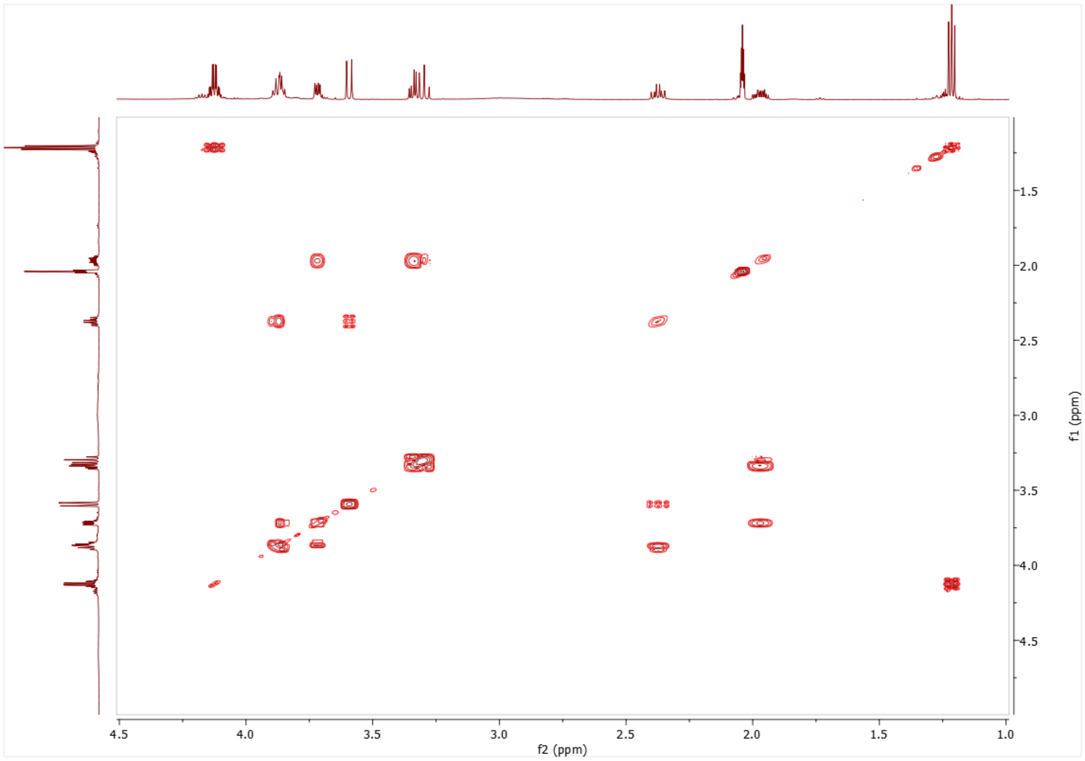

Figure S53. ^{1}H-^{1}H COSY NMR (600 MHz spectrometer, Acetone-d_6) spectrum of compound **29**.

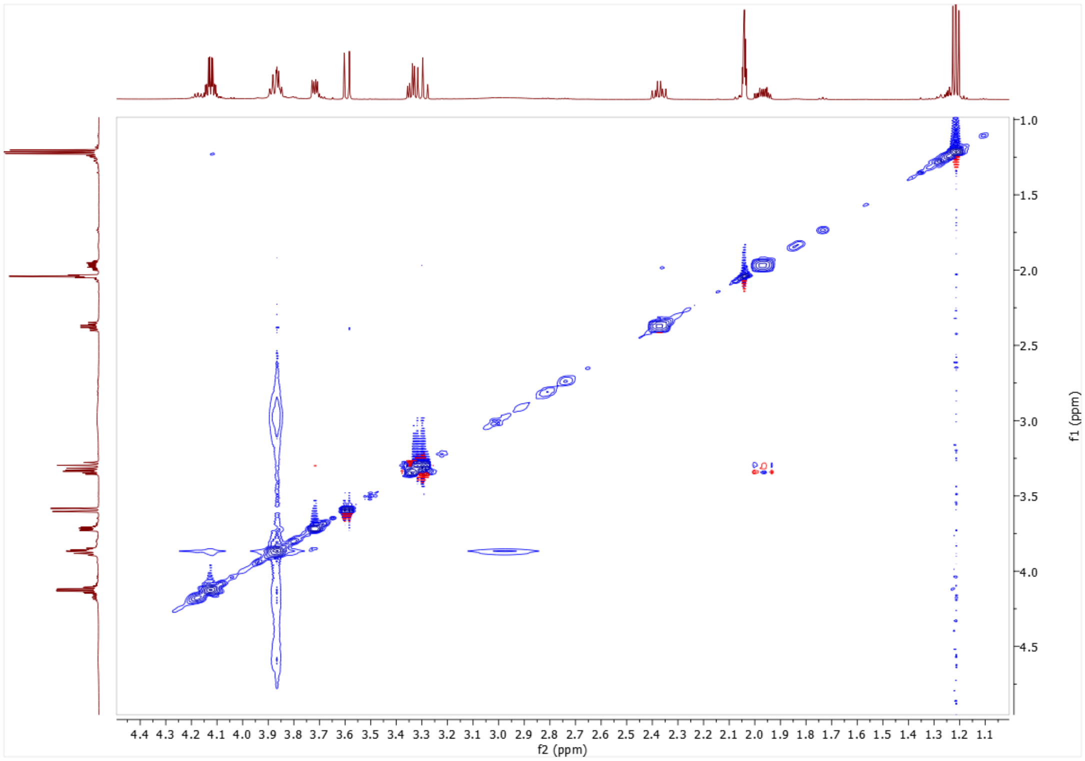

Figure S54. ^{1}H-^{1}H NOESY NMR (600 MHz spectrometer, Acetone-d_6) spectrum of compound **29**.

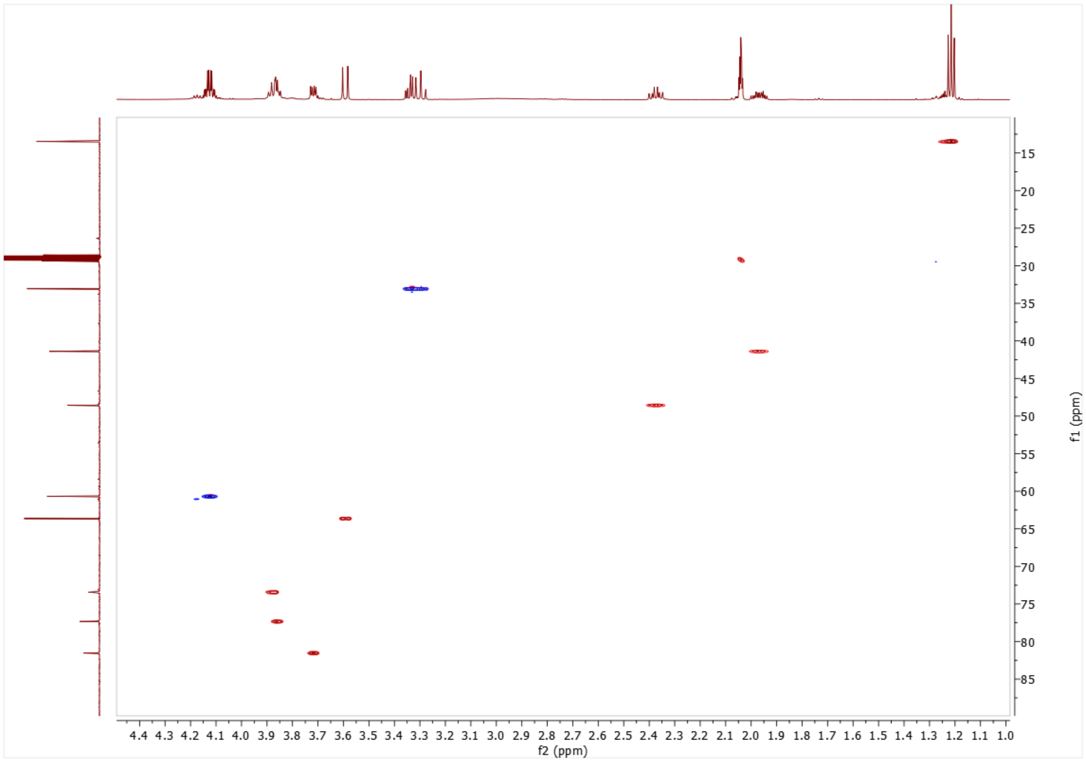

Figure S55. ^{1}H-^{13}C HSQC NMR (600 MHz spectrometer, Acetone-d_6) spectrum of compound **29**.

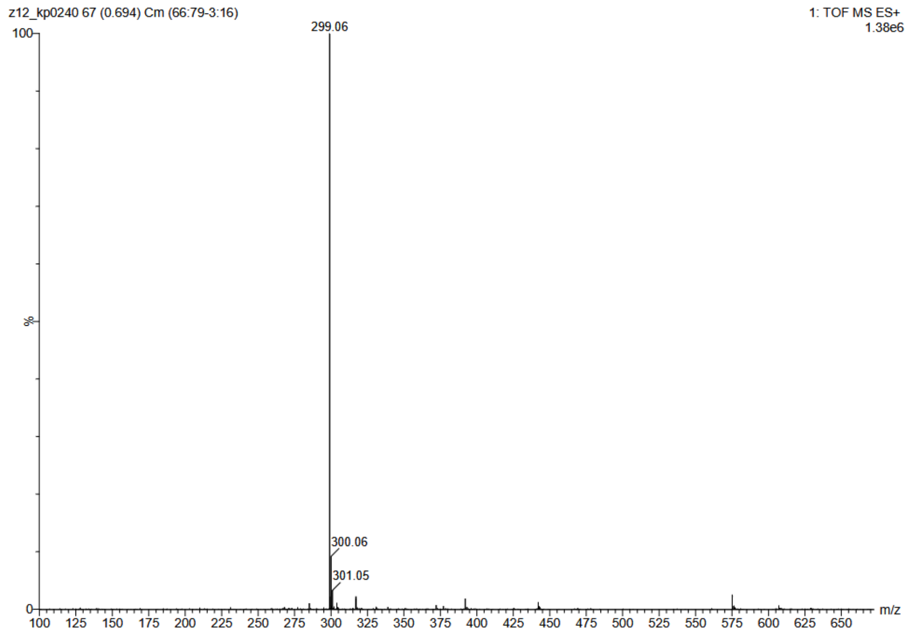

Figure S56. HR-ESI-MS spectrum of compound **29**.

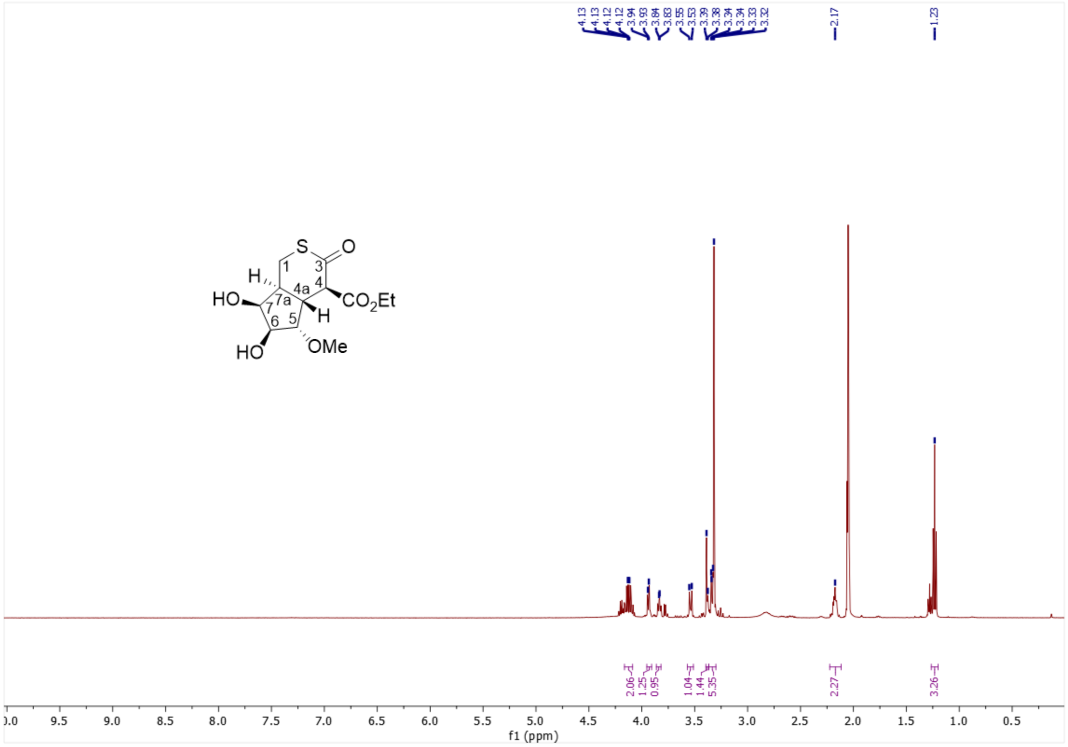

Figure S57. ^{1}H NMR (500 MHz, Acetone-d_6) spectrum of compound **30**.

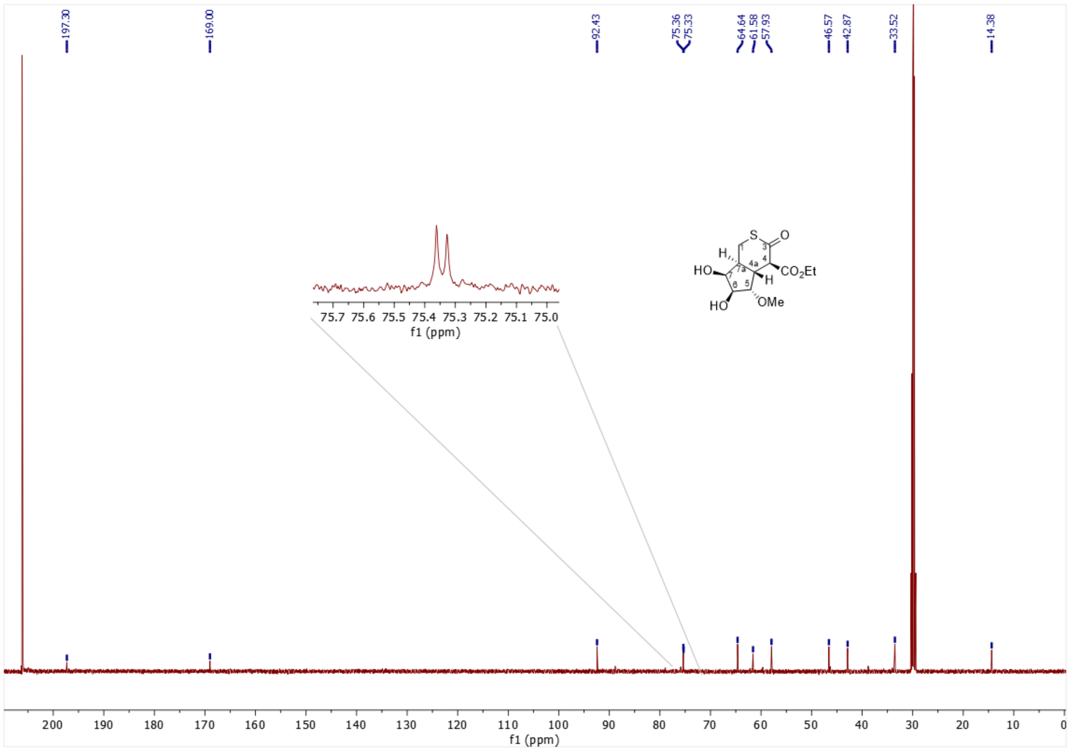

Figure S58. $^{13}C\{H\}$ NMR (126 MHz, Acetone-d_6) spectrum of compound **30**.

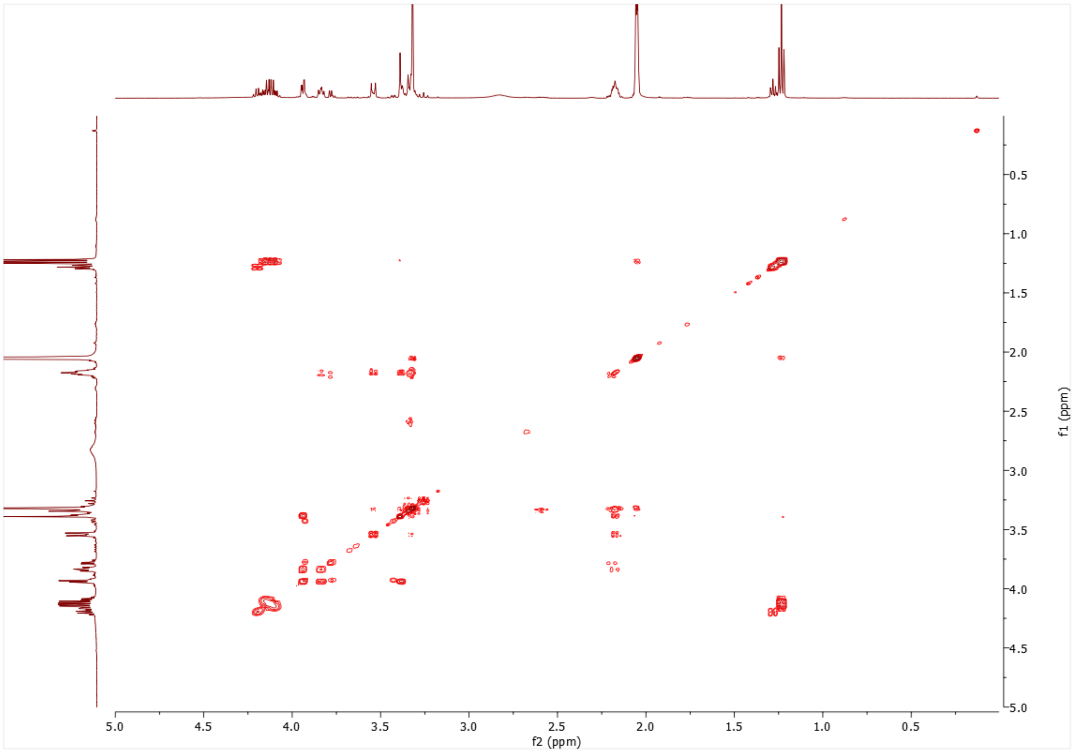

Figure S59. ^{1}H-^{1}H COSY NMR (500 MHz spectrometer, Acetone-d_6) spectrum of compound **30**.

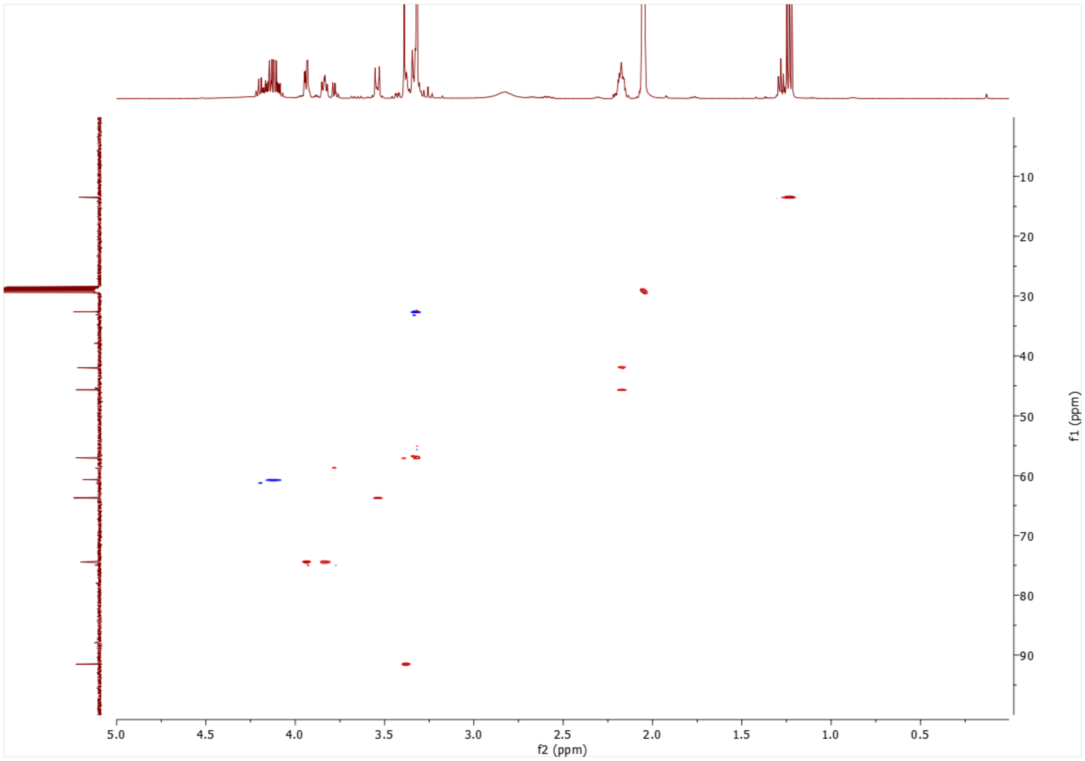

Figure S60. ^{1}H-^{13}C HSQC NMR (500 MHz spectrometer, Acetone-d_6) spectrum of compound **30**.

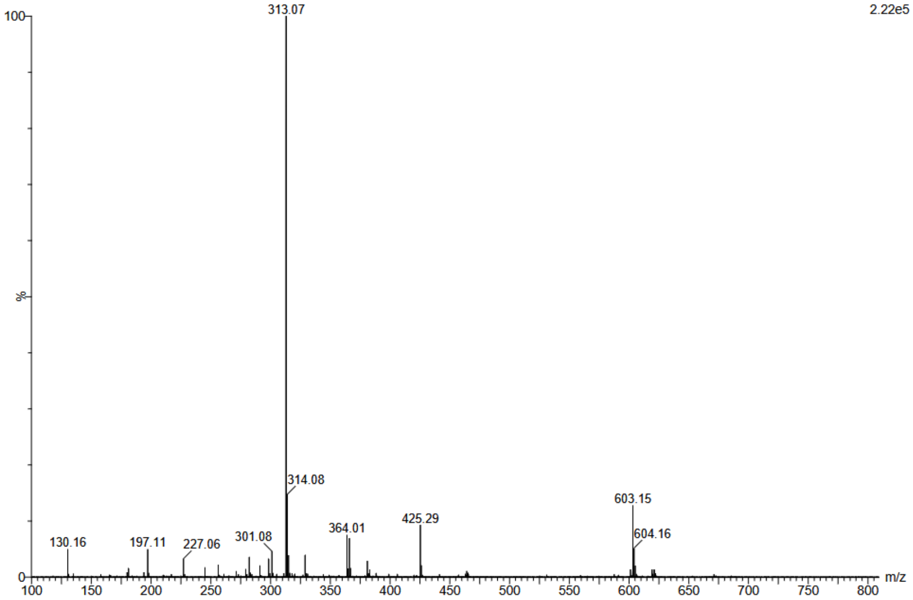

Figure S61. HR-ESI-MS spectrum of compound **30**.

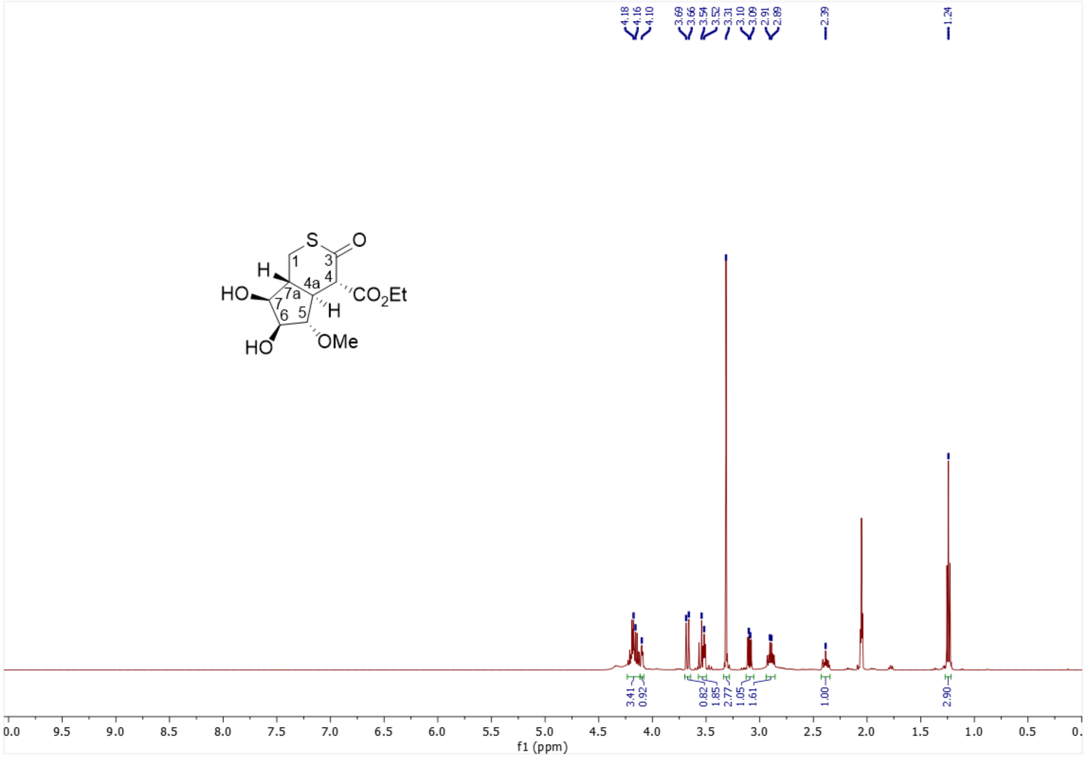

Figure S62. ^{1}H NMR (500 MHz, Acetone-d_6) spectrum of compound **32**.

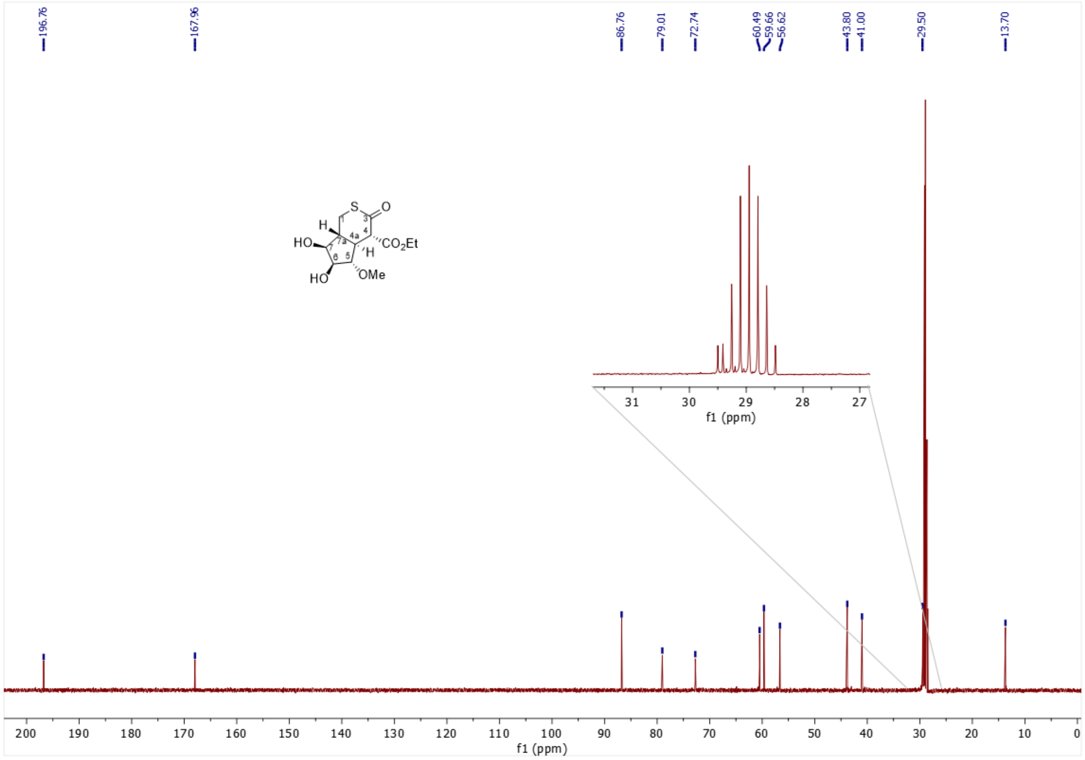

Figure S63. $^{13}C\{H\}$ NMR (126 MHz, Acetone-d_6) spectrum of compound **32**.

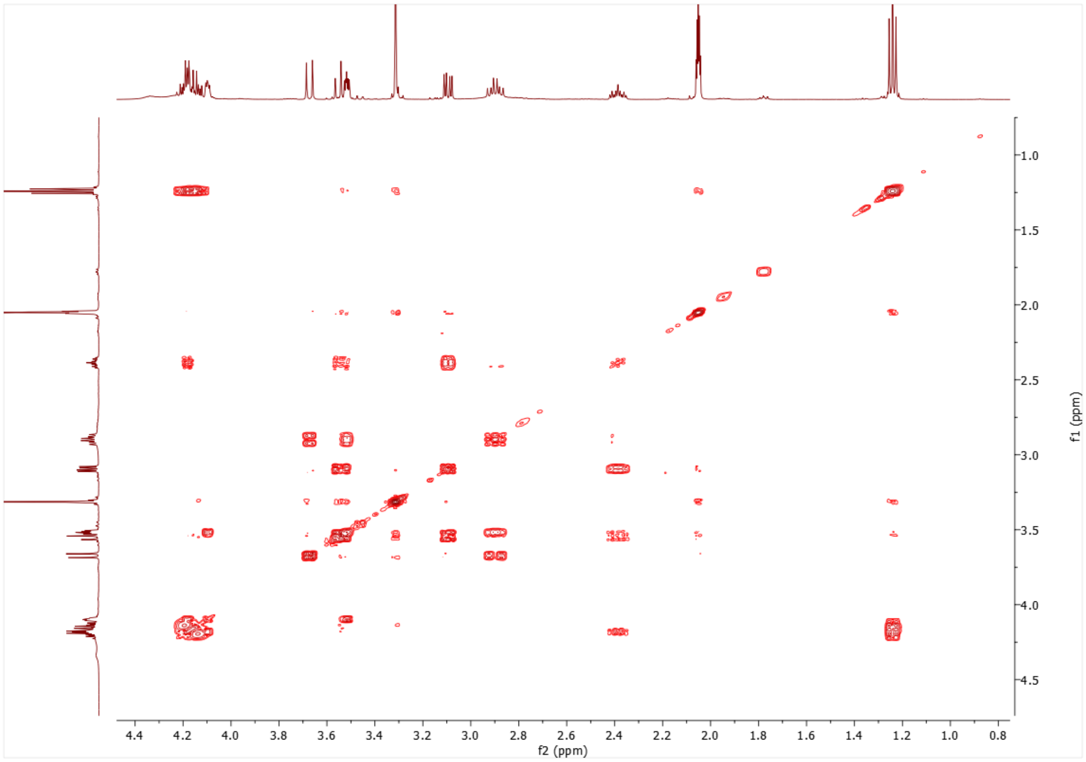

Figure S64. ^{1}H-^{1}H COSY NMR (500 MHz spectrometer, Acetone-d_6) spectrum of compound **32**.

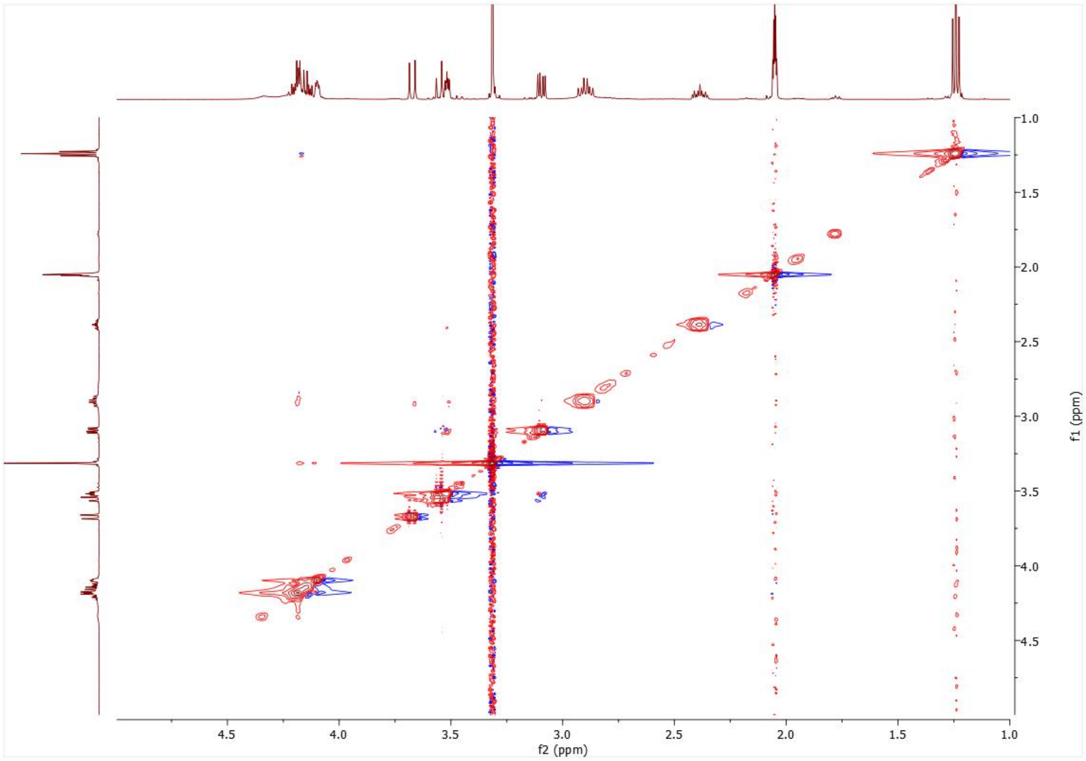

Figure S65. ^{1}H-^{1}H NOESY NMR (500 MHz spectrometer, Acetone-d_6) spectrum of compound **32**.

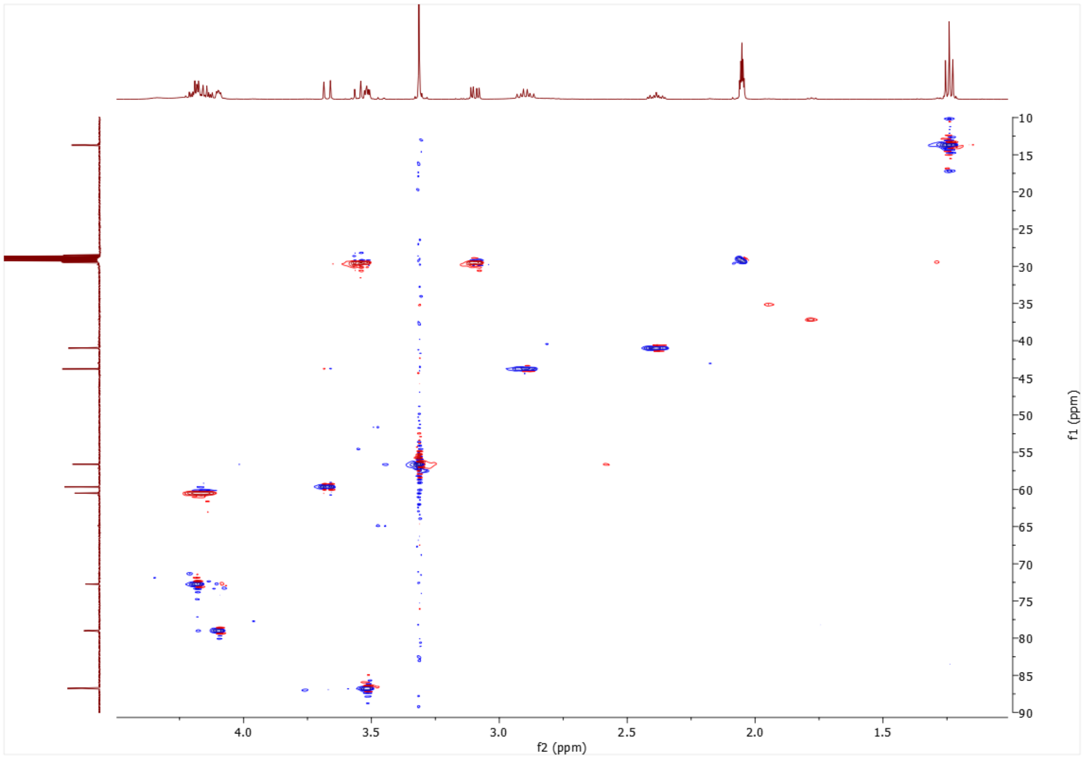

Figure S66. ^{1}H-^{13}C HSQC NMR (500 MHz spectrometer, Acetone-d_6) spectrum of compound **32**.

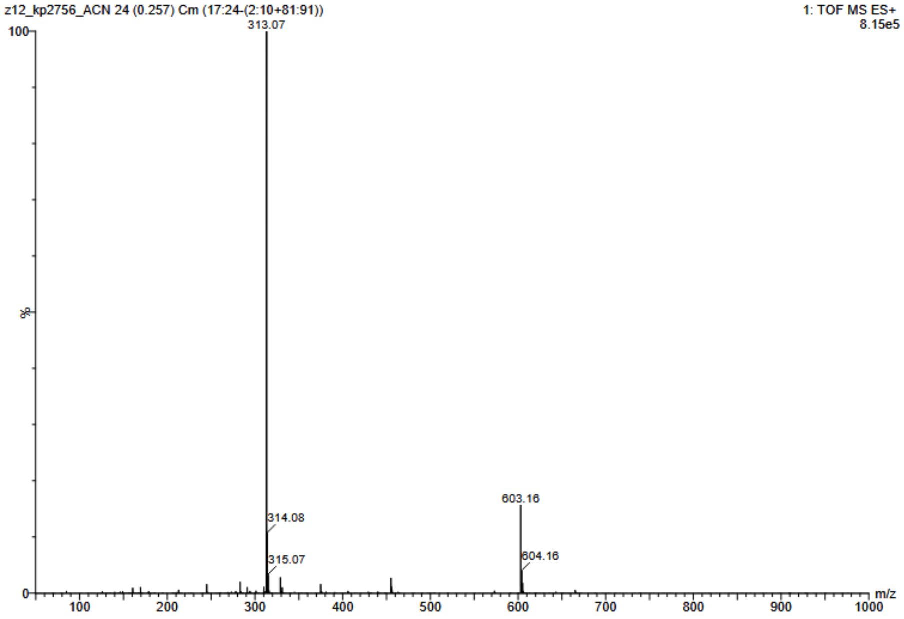

Figure S67. HR-ESI-MS spectrum of compound **32**.

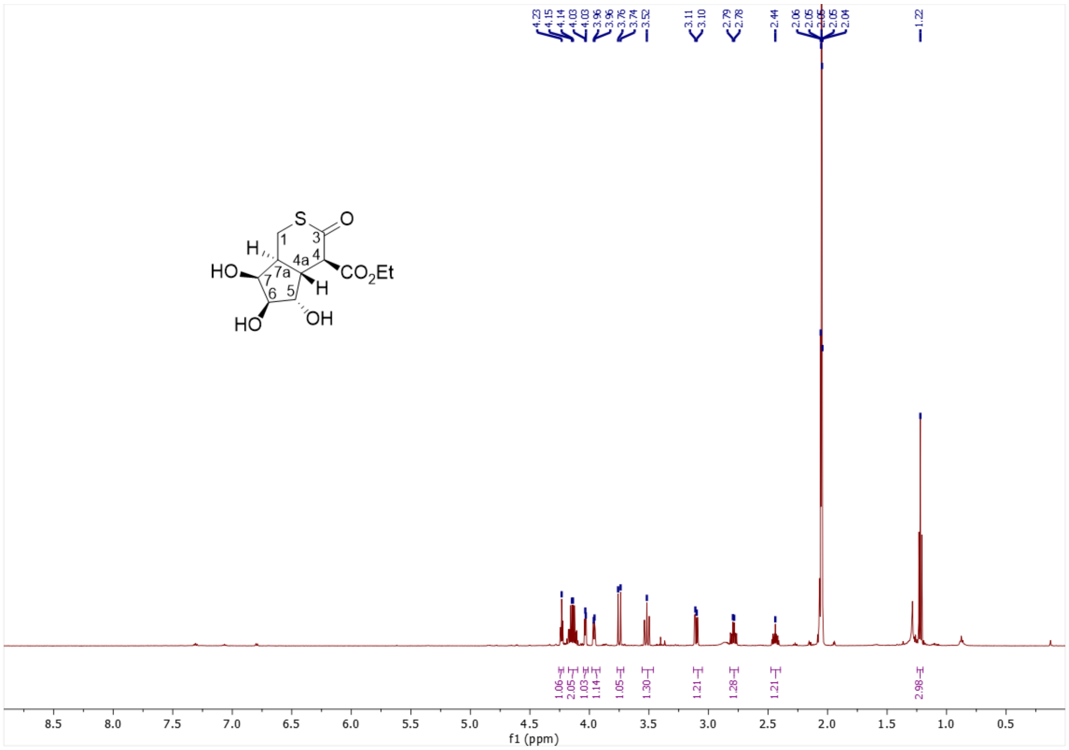

Figure S68. ^{1}H NMR (400 MHz, Acetone-d_6) spectrum of compound **31**.

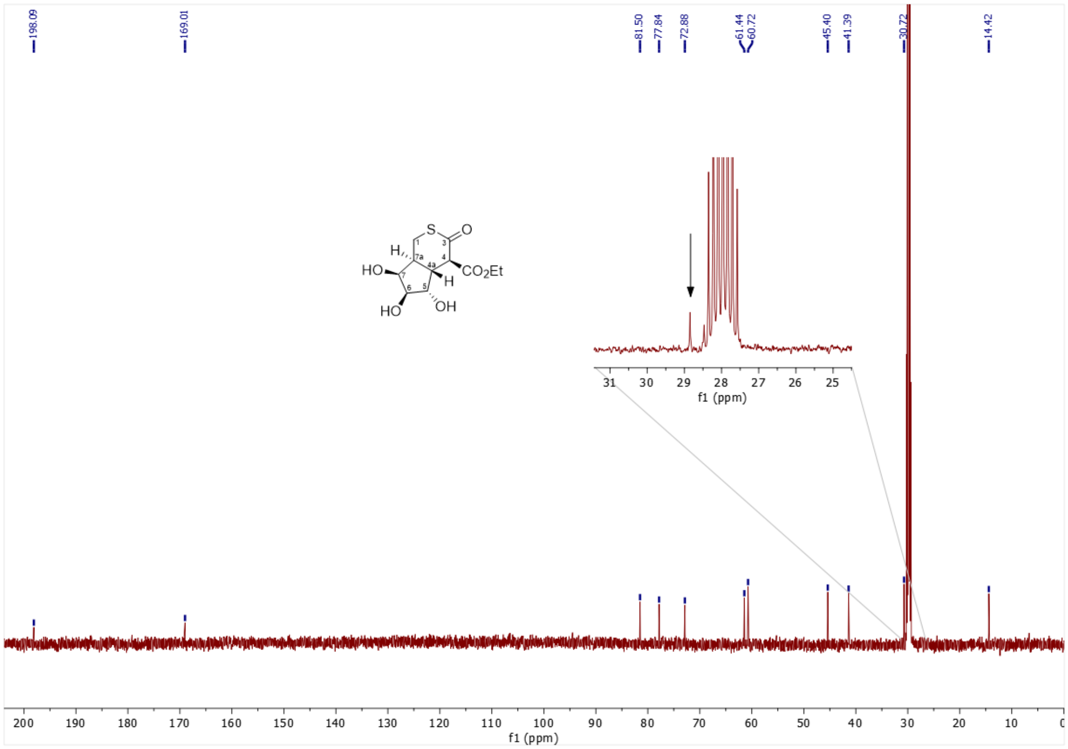

Figure S69. ^{13}C{H} NMR (101 MHz, Acetone-d_6) spectrum of compound **31**.

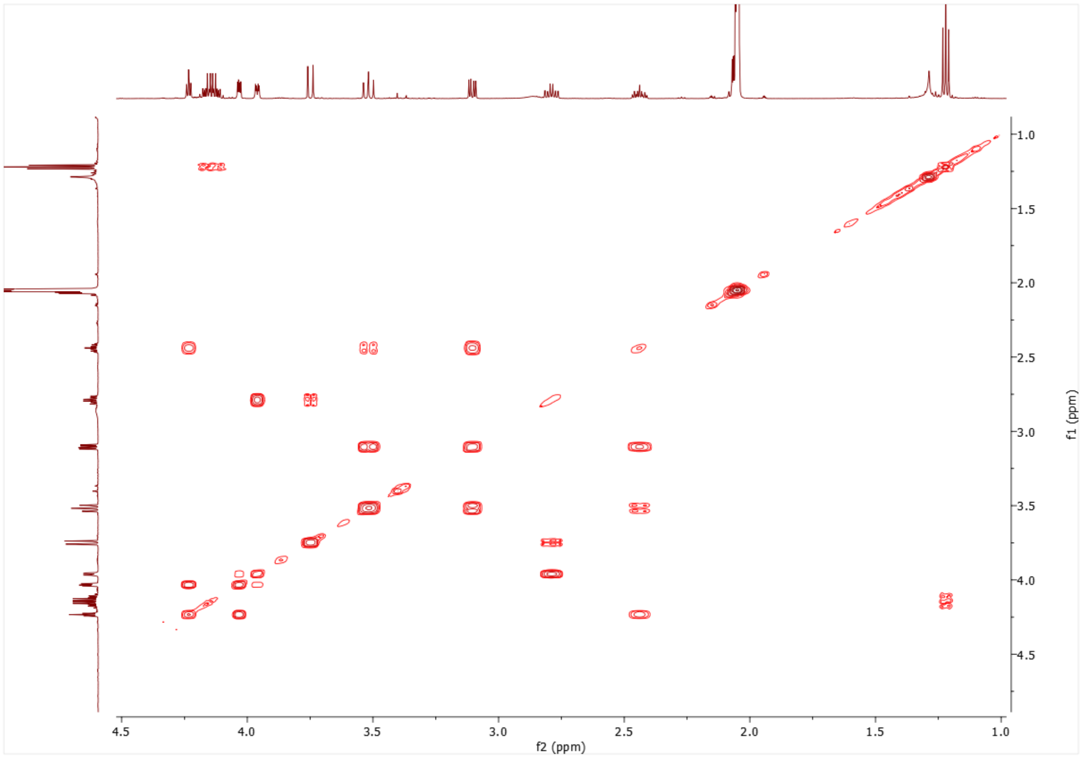

Figure S70. ^{1}H-^{1}H COSY NMR (600 MHz spectrometer, Acetone-d_6) spectrum of compound **31**.

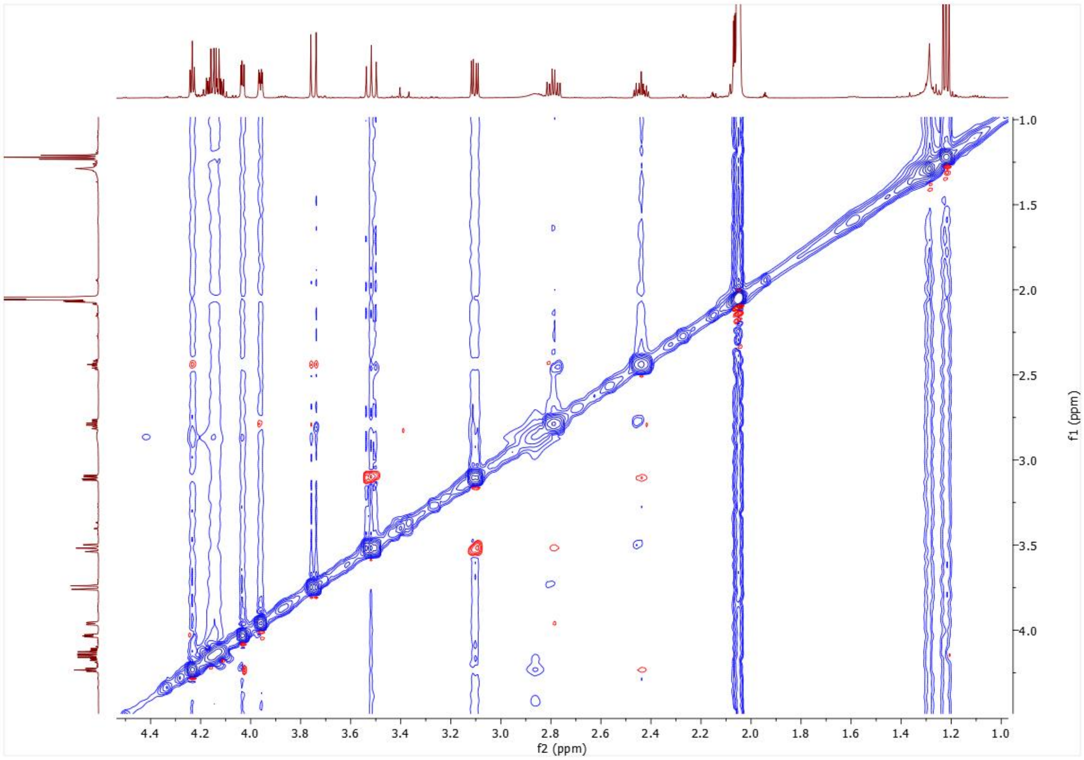

Figure S71. ^{1}H-^{1}H NOESY NMR (600 MHz spectrometer, Acetone-d_6) spectrum of compound **31**.

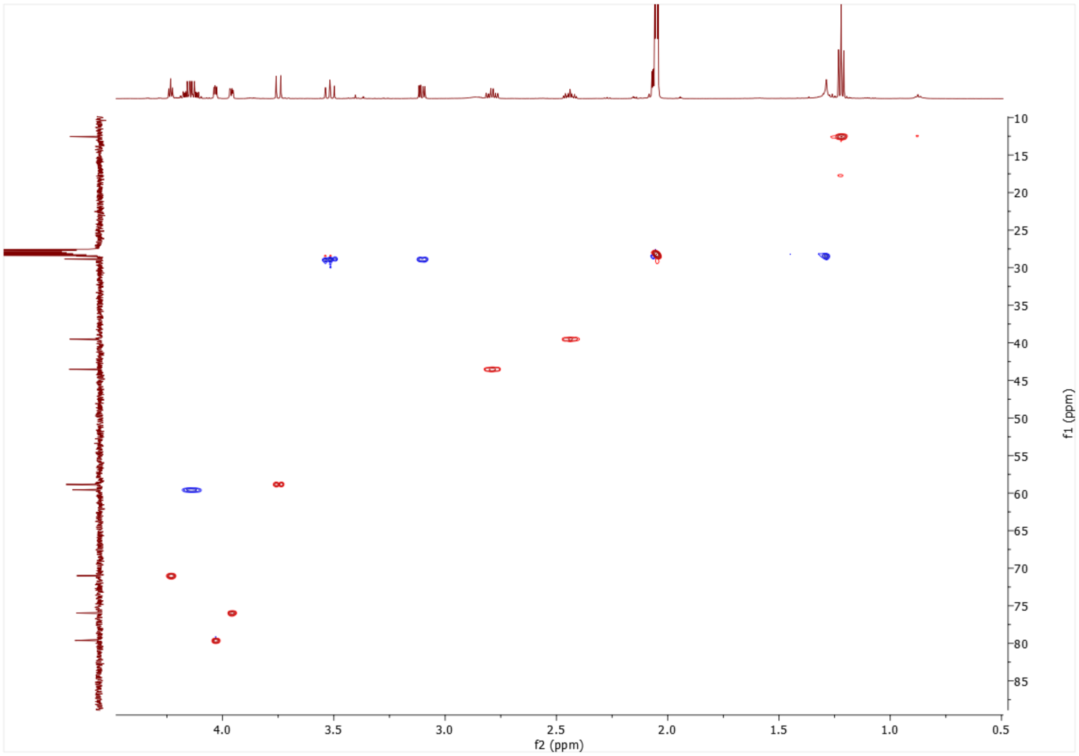

Figure S72. ^{1}H-^{13}C HSQC NMR (600 MHz spectrometer, Acetone-d_6) spectrum of compound **31**.

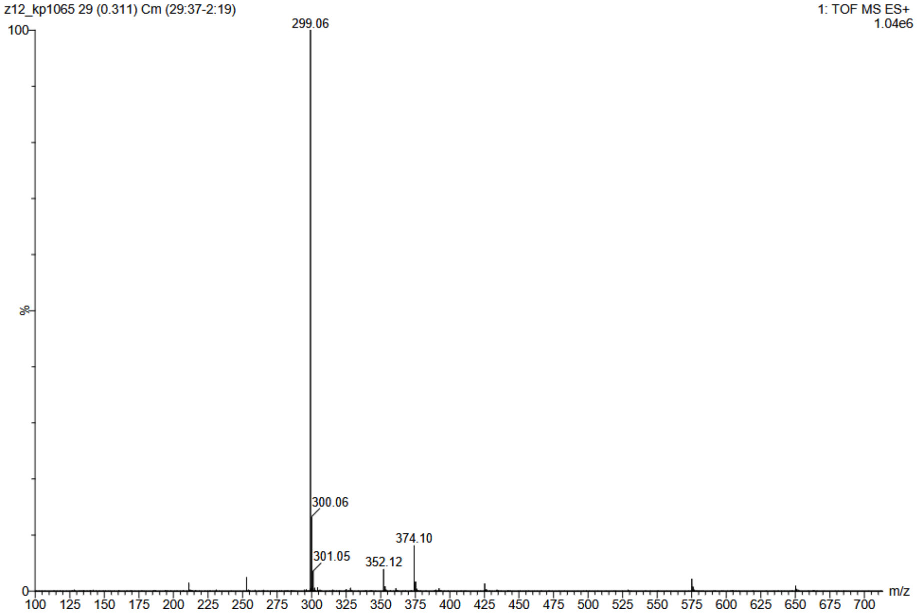

Figure S73. HR-ESI-MS spectrum of compound **31**.
