# Supplementary material for: Bicyclic δ‑Thiolactone Glycomimetics: Stereoselective Synthesis and Discovery of Stereocontrolled Antiphage Activity
Source: J Org Chem. 2026 Apr 2;91(15):5366–78. doi: 10.1021/acs.joc.6c00212 (PMC13298889; doi:10.1021/acs.joc.6c00212)
Supplement: Supplementary file 2 [file jo6c00212_si_001.zip › Cartesian Coordinates.docx]

***Supporting Information***

**Bicyclic δ-Thiolactone Glycomimetics: Stereoselective Synthesis and Discovery of Stereocontrolled Antiphage Activity**

**Karol Postrożnya, Bartosz Kamińskib, Aleksandra F. Kopera, Roman Luboradzkib, Zahra Badria*, Jan Paczesnyb* and Mykhaylo A. Potopnyka,c***

a Institute of Organic Chemistry, Polish Academy of Sciences, Kasprzaka 44/52, Warsaw, 01-224, Poland, Email: zahra.badri@icho.edu.pl; mykhaylo.potopnyk@icho.edu.pl

b Institute of Physical Chemistry, Polish Academy of Sciences, Kasprzaka 44/52, Warsaw, 01-224, Poland, Email: jpaczesny@ichf.edu.pl

c Department of Organic Chemistry, Ivan Franko National University of Lviv, Kyryla and Mefodiya 6, Lviv, 79005, Ukraine, Email: potopnyk@gmail.com

**Optimized Cartesian Coordinates of the 14 at m06-2x/def2-TZVP/SMD/ACN level of theory**

Sum of electronic and thermal Free Energies= -1645.110233 (Hartree/Particle)

6 -0.858367000 -1.741718000 2.522134000

6 -1.710073000 -0.766035000 2.245372000

6 -0.429564000 -0.400978000 -0.511937000

6 0.693600000 0.273796000 -0.246389000

6 1.989585000 -0.465027000 -0.080636000

16 2.016779000 -2.046859000 0.440171000

6 -2.854306000 -0.905219000 1.284973000

6 -2.862749000 0.213164000 0.239355000

6 -1.775307000 0.149863000 -0.850626000

7 3.073006000 0.263449000 -0.360999000

6 0.674862000 1.765816000 -0.139411000

8 1.553406000 2.503724000 -0.531510000

6 4.439993000 -0.104525000 -0.286271000

6 5.316075000 0.813014000 0.286127000

6 6.673711000 0.533090000 0.339119000

6 7.159845000 -0.659306000 -0.181350000

6 6.281888000 -1.563917000 -0.766701000

6 4.922491000 -1.292026000 -0.826443000

8 -0.422227000 2.205886000 0.452669000

6 -0.568705000 3.640317000 0.568331000

6 -1.928701000 3.908715000 1.156253000

8 -4.049022000 -0.803369000 2.051015000

6 -5.073549000 -1.705208000 1.685868000

8 -4.040693000 0.153874000 -0.549047000

8 -2.365782000 -0.740411000 -1.786307000

6 -3.755457000 -0.450382000 -1.814274000

6 -4.079974000 0.548952000 -2.907827000

6 -4.508185000 -1.752591000 -1.970403000

1 -0.928268000 -2.706674000 2.031679000

1 -0.057638000 -1.611653000 3.240161000

1 -1.629332000 0.197819000 2.743211000

1 -0.356818000 -1.481580000 -0.578036000

1 -2.808119000 -1.881978000 0.788258000

1 -2.845196000 1.172709000 0.757023000

1 -1.688471000 1.146665000 -1.290815000

1 2.904511000 1.242703000 -0.584069000

1 4.925153000 1.739795000 0.689144000

1 7.349879000 1.248396000 0.789718000

1 8.218681000 -0.880228000 -0.138033000

1 6.656853000 -2.487552000 -1.189148000

1 4.242992000 -1.987318000 -1.296311000

1 -0.457937000 4.078884000 -0.423173000

1 0.234796000 4.010997000 1.205138000

1 -2.036963000 3.420380000 2.125463000

1 -2.717565000 3.556544000 0.490413000

1 -2.050594000 4.983292000 1.295604000

1 -4.707021000 -2.737569000 1.688167000

1 -5.485717000 -1.472713000 0.702175000

1 -5.860220000 -1.611535000 2.434512000

1 -3.880255000 0.109434000 -3.885501000

1 -5.133725000 0.824273000 -2.853782000

1 -3.476380000 1.449554000 -2.788316000

1 -4.241226000 -2.435947000 -1.164209000

1 -5.582130000 -1.565510000 -1.944064000

1 -4.256946000 -2.211975000 -2.927099000

**Optimized Cartesian Coordinates of the 14a at m06-2x/def2-TZVP/SMD/ACN level of theory**

Sum of electronic and thermal Free Energies= -1645.112826 (Hartree/Particle)

6 0.402743000 -2.728816000 1.608733000

6 1.616903000 -2.214733000 1.477237000

6 0.705772000 -0.439945000 -0.798867000

6 -0.344019000 0.346522000 -0.561691000

6 -1.736857000 -0.187386000 -0.664483000

16 -2.106870000 -1.468375000 -1.660790000

6 2.543772000 -2.487474000 0.329680000

6 3.126012000 -1.202554000 -0.283717000

6 2.137895000 -0.054741000 -0.608630000

7 -2.608331000 0.439276000 0.132813000

6 -0.137621000 1.757979000 -0.100455000

8 -0.614350000 2.228870000 0.907317000

6 -3.998694000 0.206632000 0.282401000

6 -4.508703000 0.164341000 1.575667000

6 -5.871407000 -0.006660000 1.775212000

6 -6.724596000 -0.133078000 0.687159000

6 -6.209441000 -0.074919000 -0.602962000

6 -4.849410000 0.098101000 -0.812721000

8 0.609816000 2.437517000 -0.951203000

6 1.062582000 3.735071000 -0.516670000

6 1.947117000 4.289703000 -1.602166000

1 -0.012592000 -3.393879000 0.861074000

1 -0.211584000 -2.491969000 2.469156000

1 2.017861000 -1.548463000 2.234663000

1 0.521092000 -1.449152000 -1.145505000

1 3.678262000 -1.477026000 -1.185252000

1 -2.212350000 1.103624000 0.793581000

1 -3.833340000 0.263393000 2.417121000

1 -6.263157000 -0.042664000 2.783715000

1 -7.787236000 -0.269518000 0.842020000

1 -6.871635000 -0.156275000 -1.455641000

1 -4.450498000 0.159413000 -1.814727000

1 1.601252000 3.601476000 0.423434000

1 0.193588000 4.367576000 -0.334414000

1 1.388586000 4.416716000 -2.530065000

1 2.793070000 3.626933000 -1.788333000

1 2.330507000 5.262612000 -1.293195000

1 2.474514000 0.437909000 -1.529762000

8 2.293873000 0.818765000 0.496311000

8 4.005446000 -0.623602000 0.668346000

6 4.520039000 1.645329000 0.020884000

1 4.429153000 1.385232000 -1.034504000

1 4.222873000 2.685659000 0.159722000

1 5.564552000 1.535785000 0.313789000

6 3.760388000 1.065859000 2.351666000

1 4.790908000 0.936994000 2.682333000

1 3.465314000 2.101271000 2.524044000

1 3.111289000 0.406058000 2.926338000

1 3.409854000 -3.038983000 0.722414000

8 1.897902000 -3.258510000 -0.657555000

6 2.761674000 -4.137542000 -1.351848000

1 2.161268000 -4.654149000 -2.098949000

1 3.570091000 -3.601588000 -1.857551000

1 3.197039000 -4.872498000 -0.667672000

6 3.653860000 0.741689000 0.882138000

**Optimized Cartesian Coordinates of the 18 at m06-2x/def2-TZVP/SMD/ACN level of theory**

Sum of electronic and thermal Free Energies= -1645.114853 (Hartree/Particle)

6 -0.815862000 -1.842460000 2.277010000

6 -1.885614000 -1.061495000 2.253344000

6 -0.916254000 0.130058000 -0.360536000

6 0.321689000 0.618405000 -0.304086000

6 1.501583000 -0.270656000 -0.541191000

16 1.391835000 -1.618802000 -1.512262000

6 -3.042798000 -1.235039000 1.322698000

6 -3.325155000 -0.082400000 0.317942000

6 -2.169243000 0.873542000 -0.002687000

7 2.600499000 0.107688000 0.119972000

6 0.564869000 2.045253000 0.086772000

8 1.281934000 2.391329000 0.998090000

6 3.886248000 -0.488472000 0.123010000

6 4.524855000 -0.628287000 1.350835000

6 5.807573000 -1.154704000 1.406736000

6 6.454375000 -1.539013000 0.239663000

6 5.815678000 -1.382713000 -0.985339000

6 4.534288000 -0.855928000 -1.051865000

8 -0.049719000 2.890213000 -0.724993000

6 0.106352000 4.294226000 -0.426026000

6 -0.594908000 5.070509000 -1.509494000

8 -1.922295000 1.750117000 1.073408000

8 -2.862333000 -2.355588000 0.478168000

8 -3.656004000 -0.757224000 -0.884280000

6 -2.731623000 2.911825000 1.059039000

6 -3.702367000 -2.162947000 -0.643645000

6 -5.130352000 -2.590772000 -0.347831000

6 -3.111482000 -2.888717000 -1.826994000

1 -0.699709000 -2.664592000 1.580750000

1 -0.020045000 -1.673295000 2.992398000

1 -1.987943000 -0.241301000 2.955602000

1 -1.038342000 -0.905995000 -0.655512000

1 -3.943429000 -1.370912000 1.932861000

1 -4.172421000 0.511861000 0.666108000

1 -2.485147000 1.445781000 -0.887308000

1 2.480502000 0.863903000 0.789848000

1 4.010096000 -0.325119000 2.254816000

1 6.298692000 -1.265017000 2.365117000

1 7.454123000 -1.951855000 0.282126000

1 6.320953000 -1.665397000 -1.900252000

1 4.043782000 -0.720239000 -2.004607000

1 1.171606000 4.522536000 -0.390061000

1 -0.320845000 4.483022000 0.560059000

1 -1.656124000 4.822492000 -1.547723000

1 -0.149755000 4.861823000 -2.482754000

1 -0.496720000 6.137320000 -1.306311000

1 -2.437113000 3.517271000 1.914641000

1 -2.575245000 3.479457000 0.136718000

1 -3.793977000 2.668372000 1.147890000

1 -5.540724000 -2.029220000 0.492113000

1 -5.754197000 -2.407368000 -1.222906000

1 -5.157760000 -3.654967000 -0.111742000

1 -3.114357000 -3.962925000 -1.641453000

1 -3.705423000 -2.686109000 -2.718168000

1 -2.087118000 -2.554576000 -1.993127000

**Optimized Cartesian Coordinates of the 18a at m06-2x/def2-TZVP/SMD/ACN level of theory**

Sum of electronic and thermal Free Energies= -1645.112087 (Hartree/Particle)

6 -0.380666000 -1.580904000 -1.956983000

6 -1.612250000 -1.346999000 -1.528656000

6 -0.460742000 0.272522000 1.103005000

6 0.631026000 0.701310000 0.474424000

6 1.985594000 0.195863000 0.874260000

16 2.357654000 -0.076259000 2.470490000

6 -2.133556000 -1.842620000 -0.211399000

6 -2.659215000 -0.741824000 0.721893000

6 -1.886657000 0.571056000 0.728221000

7 2.791927000 -0.033662000 -0.164990000

6 0.534452000 1.611294000 -0.714132000

8 1.075621000 1.405068000 -1.776086000

6 4.122847000 -0.522987000 -0.167442000

6 4.444322000 -1.495816000 -1.108359000

6 5.743708000 -1.975342000 -1.189451000

6 6.721963000 -1.484394000 -0.334806000

6 6.396542000 -0.501483000 0.592781000

6 5.100800000 -0.013694000 0.680160000

8 -0.197186000 2.681782000 -0.458411000

6 -0.399240000 3.603703000 -1.553474000

6 -1.215336000 4.754043000 -1.026640000

1 0.321975000 -2.152083000 -1.356845000

1 -0.030166000 -1.211667000 -2.912929000

1 -2.311621000 -0.777470000 -2.135532000

1 -0.327840000 -0.398510000 1.946928000

1 2.380428000 0.094599000 -1.086400000

1 3.672123000 -1.871537000 -1.769234000

1 5.988170000 -2.735173000 -1.920653000

1 7.735360000 -1.860061000 -0.395053000

1 7.158427000 -0.103040000 1.250744000

1 4.851878000 0.761769000 1.389508000

1 -0.909856000 3.071142000 -2.356986000

1 0.576968000 3.926166000 -1.915416000

1 -0.689530000 5.262565000 -0.218113000

1 -2.181172000 4.408203000 -0.657477000

1 -1.387345000 5.469419000 -1.831287000

1 -1.958112000 1.025519000 -0.264616000

8 -2.373057000 1.467513000 1.704326000

6 -3.529846000 2.193526000 1.324848000

1 -3.599787000 3.048005000 1.997095000

1 -3.442978000 2.554443000 0.295344000

1 -4.432995000 1.587581000 1.411764000

1 -1.381523000 -2.460902000 0.281769000

1 -2.693362000 -1.126214000 1.749014000

8 -3.309287000 -2.626068000 -0.406192000

8 -3.967811000 -0.546249000 0.220074000

6 -4.467447000 -1.827014000 -0.141548000

6 -5.236338000 -2.451741000 1.006924000

1 -5.530808000 -3.468145000 0.743601000

1 -4.614957000 -2.488100000 1.902334000

1 -6.133473000 -1.868116000 1.215896000

6 -5.303512000 -1.678273000 -1.392352000

1 -5.672257000 -2.654530000 -1.707818000

1 -6.157225000 -1.029174000 -1.194282000

1 -4.701195000 -1.246634000 -2.191608000

**Optimized Cartesian Coordinates of the 19 at m06-2x/def2-TZVP/SMD/ACN level of theory**

Sum of electronic and thermal Free Energies= -1951.560983 (Hartree/Particle)

6 -0.744702000 2.304695000 2.392848000

6 0.544134000 2.024830000 2.271116000

6 -0.133124000 0.666648000 -0.387792000

6 -1.067563000 -0.282740000 -0.403054000

6 -2.517038000 0.076507000 -0.509003000

16 -3.007282000 1.487659000 -1.242431000

6 1.460520000 2.660550000 1.275833000

6 2.082030000 1.733765000 0.195640000

6 1.331273000 0.444013000 -0.144853000

7 -3.332524000 -0.814394000 0.061962000

6 -0.677713000 -1.721168000 -0.230439000

8 -1.169185000 -2.479935000 0.572380000

6 -4.747662000 -0.791687000 0.149576000

6 -5.319124000 -1.097845000 1.380105000

6 -6.700164000 -1.140296000 1.508445000

6 -7.510082000 -0.880339000 0.410885000

6 -6.931464000 -0.590317000 -0.819568000

6 -5.552052000 -0.547676000 -0.958626000

8 0.264664000 -2.066649000 -1.092167000

6 0.798080000 -3.404742000 -0.992576000

6 1.680350000 -3.618577000 -2.194342000

8 1.463475000 -0.491381000 0.934157000

8 0.791109000 3.636360000 0.501029000

8 2.072437000 2.526429000 -0.975313000

6 1.560536000 3.827530000 -0.670363000

6 2.712168000 4.788433000 -0.429978000

6 0.650995000 4.276767000 -1.786862000

1 -1.232348000 3.020874000 1.742176000

1 -1.349012000 1.819857000 3.149850000

1 1.020083000 1.309496000 2.933512000

1 -0.447397000 1.694109000 -0.526466000

1 2.287276000 3.123184000 1.826794000

1 3.104875000 1.465184000 0.472333000

1 1.785772000 0.037098000 -1.049441000

1 -2.884066000 -1.556785000 0.593563000

1 -4.676647000 -1.298506000 2.229206000

1 -7.140677000 -1.375060000 2.469016000

1 -8.587516000 -0.910023000 0.510960000

1 -7.557536000 -0.402395000 -1.682626000

1 -5.102170000 -0.338337000 -1.918128000

1 -0.031592000 -4.110363000 -0.964601000

1 1.350302000 -3.483562000 -0.052367000

1 2.474318000 -2.871248000 -2.230962000

1 1.096138000 -3.557488000 -3.113017000

1 2.135542000 -4.607845000 -2.138344000

1 3.365637000 4.426253000 0.364337000

1 3.300146000 4.887273000 -1.342622000

1 2.324027000 5.768840000 -0.152498000

1 0.228939000 5.253237000 -1.548670000

1 1.218365000 4.357823000 -2.713920000

1 -0.157384000 3.557719000 -1.921419000

6 2.566074000 -1.245460000 1.077986000

8 3.379151000 -1.154969000 0.044769000

6 4.715172000 -1.779446000 0.070274000

6 5.279169000 -1.404498000 -1.287841000

1 6.291782000 -1.797211000 -1.380666000

1 4.666159000 -1.826134000 -2.085893000

1 5.311986000 -0.319881000 -1.402165000

6 5.530348000 -1.155073000 1.189128000

1 6.555341000 -1.522250000 1.121832000

1 5.547424000 -0.068650000 1.084564000

1 5.133546000 -1.416881000 2.168322000

6 4.592662000 -3.287083000 0.207402000

1 3.922517000 -3.685746000 -0.555635000

1 5.579701000 -3.726952000 0.055890000

1 4.230269000 -3.574994000 1.191315000

8 2.706278000 -1.909000000 2.068559000

**Optimized Cartesian Coordinates of the 19a at m06-2x/def2-TZVP/SMD/ACN level of theory**

Sum of electronic and thermal Free Energies= -1951.559021 (Hartree/Particle)

6 -0.594052000 1.808093000 -2.281102000

6 0.609813000 1.884583000 -1.733979000

6 -0.453803000 0.261170000 1.093835000

6 -1.433361000 -0.388359000 0.470452000

6 -2.865827000 -0.187630000 0.870967000

16 -3.279147000 0.026978000 2.464062000

6 0.879807000 2.591046000 -0.438669000

6 1.487261000 1.699609000 0.651563000

6 0.994743000 0.263629000 0.698589000

7 -3.706080000 -0.173768000 -0.166685000

6 -1.142187000 -1.280108000 -0.701475000

8 -1.683273000 -1.188018000 -1.778063000

6 -5.113418000 0.000732000 -0.169372000

6 -5.650047000 0.841705000 -1.138990000

6 -7.024295000 1.012291000 -1.220892000

6 -7.862954000 0.343704000 -0.338636000

6 -7.320281000 -0.506609000 0.617611000

6 -5.947423000 -0.685849000 0.706572000

8 -0.233069000 -2.189049000 -0.400886000

6 0.207023000 -3.058376000 -1.469121000

6 1.024821000 -4.152581000 -0.837198000

1 -1.456287000 2.265791000 -1.804673000

1 -0.762789000 1.286990000 -3.215148000

1 1.469932000 1.425813000 -2.216276000

1 -0.715370000 0.903818000 1.929140000

1 -3.280398000 -0.218343000 -1.089355000

1 -4.984935000 1.357537000 -1.821583000

1 -7.437079000 1.670551000 -1.974618000

1 -8.935224000 0.478823000 -0.399509000

1 -7.969828000 -1.043191000 1.297517000

1 -5.527894000 -1.359759000 1.438806000

1 0.789600000 -2.455655000 -2.168846000

1 -0.669190000 -3.446735000 -1.986712000

1 0.405011000 -4.768946000 -0.185347000

1 1.838426000 -3.727246000 -0.250287000

1 1.448350000 -4.787132000 -1.616372000

1 1.166917000 -0.228232000 -0.253743000

8 1.715510000 -0.414895000 1.740002000

1 -0.024130000 3.090769000 -0.086462000

1 1.319038000 2.157024000 1.633949000

8 1.908366000 3.564327000 -0.605822000

8 2.860460000 1.725294000 0.322638000

6 3.148161000 3.052794000 -0.102996000

6 3.595605000 3.909460000 1.065761000

1 3.734221000 4.938985000 0.734303000

1 2.846064000 3.895163000 1.857458000

1 4.541180000 3.535505000 1.459616000

6 4.174132000 2.996890000 -1.211012000

1 4.332976000 3.999488000 -1.608833000

1 5.122899000 2.621066000 -0.827588000

1 3.822780000 2.345037000 -2.011258000

6 2.905264000 -0.989126000 1.487724000

8 3.060947000 -1.302203000 0.216771000

6 4.361934000 -1.746019000 -0.306681000

6 4.072554000 -1.934221000 -1.785392000

1 3.701633000 -1.004590000 -2.221205000

1 4.988211000 -2.222271000 -2.302359000

1 3.327369000 -2.717843000 -1.933492000

6 5.385039000 -0.644800000 -0.091872000

1 5.579236000 -0.482029000 0.967354000

1 6.319568000 -0.934347000 -0.574639000

1 5.032634000 0.284890000 -0.538553000

6 4.785197000 -3.058845000 0.331036000

1 5.043129000 -2.931982000 1.379564000

1 3.993832000 -3.804360000 0.243293000

1 5.661440000 -3.430981000 -0.202574000

8 3.664227000 -1.208953000 2.391596000

**Optimized Cartesian Coordinates of the 14-TS at m06-2x/def2-TZVP/SMD/ACN level of theory**

Sum of electronic and thermal Free Energies= -1645.078887 (Hartree/Particle)

6 0.032675000 -1.820338000 -1.811466000

6 0.946101000 -0.827303000 -1.545978000

6 0.604601000 -0.286975000 0.437693000

6 -0.582493000 0.442072000 0.450716000

6 -1.827025000 -0.274719000 0.346945000

16 -1.772552000 -1.930752000 0.018528000

6 2.395873000 -1.132785000 -1.267354000

6 2.952271000 0.005747000 -0.407173000

6 1.986098000 0.223687000 0.760523000

7 -2.981273000 0.432810000 0.364069000

6 -0.564729000 1.905273000 0.252150000

8 -1.468955000 2.668428000 0.535495000

6 -4.311284000 -0.022516000 0.261728000

6 -5.204268000 0.755861000 -0.472508000

6 -6.542319000 0.397141000 -0.553095000

6 -7.000686000 -0.743074000 0.093175000

6 -6.110803000 -1.511357000 0.835527000

6 -4.773433000 -1.155851000 0.928692000

8 0.561190000 2.328446000 -0.319041000

6 0.711847000 3.749464000 -0.503996000

6 2.085199000 3.979039000 -1.079837000

8 3.050978000 -1.259468000 -2.513087000

6 4.410516000 -1.643165000 -2.427135000

8 4.179244000 -0.309026000 0.221916000

8 2.543574000 -0.627606000 1.755049000

6 3.952555000 -0.560989000 1.613860000

6 4.530349000 0.584998000 2.424311000

6 4.537749000 -1.900693000 1.992732000

1 0.291851000 -2.857984000 -1.633968000

1 -0.817377000 -1.638550000 -2.453061000

1 0.770563000 0.152348000 -1.981162000

1 0.496373000 -1.324706000 0.726736000

1 2.481487000 -2.074890000 -0.706353000

1 3.070786000 0.901561000 -1.022093000

1 1.990037000 1.255843000 1.104276000

1 -2.867638000 1.441138000 0.415403000

1 -4.839289000 1.641459000 -0.979318000

1 -7.224708000 1.010100000 -1.128473000

1 -8.042773000 -1.027940000 0.025805000

1 -6.461028000 -2.392529000 1.358584000

1 -4.095865000 -1.746015000 1.528570000

1 0.586858000 4.240922000 0.461259000

1 -0.075439000 4.097071000 -1.173452000

1 2.195383000 3.465787000 -2.035837000

1 2.856967000 3.622923000 -0.395832000

1 2.234567000 5.046890000 -1.242292000

1 4.535017000 -2.504157000 -1.761679000

1 5.042244000 -0.828444000 -2.066450000

1 4.723458000 -1.920663000 -3.433173000

1 4.356493000 0.414361000 3.487255000

1 5.604248000 0.654604000 2.249006000

1 4.067949000 1.529133000 2.133207000

1 4.094354000 -2.683242000 1.376879000

1 5.616804000 -1.888618000 1.837890000

1 4.337399000 -2.111321000 3.043638000

**Optimized Cartesian Coordinates of the 14a-TS at m06-2x/def2-TZVP/SMD/ACN level of theory**

Sum of electronic and thermal Free Energies= -1645.080687 (Hartree/Particle)

6 -0.083879000 -2.599368000 1.083280000

6 1.060090000 -1.843344000 0.972562000

6 0.778403000 -0.495968000 -0.649619000

6 -0.320604000 0.317368000 -0.417378000

6 -1.620063000 -0.255731000 -0.323968000

16 -1.801603000 -1.908759000 -0.584179000

6 2.315275000 -2.372734000 0.321797000

6 3.138898000 -1.189115000 -0.201235000

6 2.202943000 -0.032306000 -0.641245000

7 -2.633409000 0.540178000 0.142725000

6 -0.118850000 1.757388000 -0.080573000

8 -0.571941000 2.320128000 0.891850000

6 -4.012055000 0.286284000 0.166411000

6 -4.755207000 0.819400000 1.220161000

6 -6.132131000 0.656125000 1.260782000

6 -6.785404000 -0.045979000 0.256291000

6 -6.045167000 -0.568483000 -0.798700000

6 -4.669555000 -0.403172000 -0.853055000

8 0.582335000 2.377336000 -1.019250000

6 1.026890000 3.711139000 -0.716491000

6 1.919283000 4.154607000 -1.846530000

1 -0.115716000 -3.595044000 0.660235000

1 -0.807648000 -2.374827000 1.853832000

1 1.223458000 -1.043674000 1.687345000

1 0.603929000 -1.392219000 -1.233011000

1 3.802780000 -1.502466000 -1.009447000

1 -2.339797000 1.363080000 0.654936000

1 -4.241533000 1.362462000 2.005151000

1 -6.693230000 1.076173000 2.086269000

1 -7.858800000 -0.180389000 0.290363000

1 -6.543964000 -1.103600000 -1.597338000

1 -4.109784000 -0.795170000 -1.690273000

1 1.560774000 3.681121000 0.234901000

1 0.155350000 4.357378000 -0.606453000

1 1.372258000 4.170681000 -2.789801000

1 2.773979000 3.484156000 -1.946654000

1 2.289238000 5.160246000 -1.644394000

1 2.474229000 0.337366000 -1.635787000

8 2.420527000 0.962991000 0.343335000

8 3.878265000 -0.647827000 0.879764000

6 4.767996000 1.412536000 -0.015290000

1 4.710866000 1.020590000 -1.032388000

1 4.612387000 2.491476000 -0.043526000

1 5.763273000 1.209936000 0.381028000

6 3.750335000 1.272093000 2.291609000

1 4.724787000 1.070240000 2.736206000

1 3.577180000 2.348347000 2.302517000

1 2.976004000 0.775350000 2.876080000

1 2.926876000 -2.903887000 1.062371000

8 1.957969000 -3.247006000 -0.723836000

6 2.966057000 -4.186679000 -1.040349000

1 2.601433000 -4.781167000 -1.876345000

1 3.900527000 -3.699035000 -1.333358000

1 3.163282000 -4.843950000 -0.187527000

6 3.720100000 0.762735000 0.872868000

**Optimized Cartesian Coordinates of the 18-TS at m06-2x/def2-TZVP/SMD/ACN level of theory**

Sum of electronic and thermal Free Energies= -1645.081122 ((Hartree/Particle)

6 0.450414000 -2.071239000 -1.614918000

6 1.339375000 -1.019974000 -1.593524000

6 0.932402000 0.100566000 0.157898000

6 -0.361347000 0.594161000 0.113645000

6 -1.460881000 -0.310261000 0.141248000

16 -1.151313000 -1.958701000 0.297219000

6 2.784452000 -1.202266000 -1.197891000

6 3.312193000 -0.034920000 -0.317370000

6 2.170645000 0.930762000 -0.000020000

7 -2.707459000 0.187601000 -0.131222000

6 -0.598920000 2.042765000 -0.160329000

8 -1.333680000 2.475958000 -1.020293000

6 -3.956116000 -0.436046000 0.002562000

6 -4.961630000 -0.078503000 -0.896695000

6 -6.236006000 -0.611845000 -0.773162000

6 -6.523935000 -1.513460000 0.243127000

6 -5.524116000 -1.862049000 1.144503000

6 -4.249237000 -1.327594000 1.035080000

8 0.035466000 2.821823000 0.708765000

6 -0.107611000 4.243187000 0.520259000

6 0.563241000 4.928036000 1.682746000

8 1.992750000 1.845506000 -1.058393000

8 2.923459000 -2.340657000 -0.366390000

8 3.730163000 -0.663232000 0.879809000

6 2.818825000 2.988774000 -0.953182000

6 3.908951000 -2.046702000 0.609425000

6 5.302526000 -2.303900000 0.060787000

6 3.609027000 -2.833905000 1.859741000

1 0.758534000 -3.050470000 -1.272763000

1 -0.412727000 -2.033630000 -2.264073000

1 1.158713000 -0.182969000 -2.257541000

1 1.080350000 -0.833306000 0.688303000

1 3.399440000 -1.308898000 -2.096516000

1 4.139995000 0.494047000 -0.793804000

1 2.392688000 1.459120000 0.935743000

1 -2.724938000 1.080799000 -0.607880000

1 -4.733397000 0.621423000 -1.692237000

1 -7.003927000 -0.323132000 -1.480009000

1 -7.516207000 -1.935385000 0.336659000

1 -5.739055000 -2.551297000 1.951798000

1 -3.488647000 -1.590846000 1.756085000

1 -1.169725000 4.483237000 0.471826000

1 0.348954000 4.509590000 -0.434946000

1 1.620929000 4.668190000 1.738278000

1 0.084187000 4.648778000 2.621754000

1 0.479412000 6.008376000 1.560469000

1 2.605296000 3.620908000 -1.813495000

1 2.602229000 3.537760000 -0.031869000

1 3.879974000 2.722827000 -0.962769000

1 5.479053000 -1.709524000 -0.837360000

1 6.049489000 -2.037475000 0.808880000

1 5.413338000 -3.359185000 -0.189833000

1 3.695807000 -3.901071000 1.655572000

1 4.318239000 -2.568635000 2.643691000

1 2.596457000 -2.611908000 2.197506000

**Optimized Cartesian Coordinates of the 18a-TS at m06-2x/def2-TZVP/SMD/ACN level of theory**

Sum of electronic and thermal Free Energies= -1645.076112 (Hartree/Particle)

6 0.218011000 -2.407589000 0.906029000

6 1.210086000 -1.461042000 0.803115000

6 0.576677000 -0.072905000 -0.586892000

6 -0.630911000 0.521853000 -0.241292000

6 -1.837714000 -0.254428000 -0.351735000

16 -1.709216000 -1.898542000 -0.715596000

6 2.481347000 -1.747164000 0.040813000

6 2.933166000 -0.545545000 -0.833586000

6 1.934820000 0.591820000 -0.644966000

7 -3.018901000 0.349114000 -0.064559000

6 -0.686755000 1.867503000 0.373794000

8 -1.607912000 2.646267000 0.222516000

6 -4.314708000 -0.202287000 -0.019993000

6 -4.605235000 -1.392885000 0.643210000

6 -5.913676000 -1.854106000 0.687948000

6 -6.941368000 -1.134128000 0.091360000

6 -6.653029000 0.064975000 -0.548582000

6 -5.347485000 0.528645000 -0.606878000

8 0.369923000 2.151494000 1.127696000

6 0.484885000 3.499532000 1.627974000

6 1.013416000 4.423008000 0.555419000

8 1.899000000 1.523905000 -1.701141000

8 3.561145000 -1.927359000 0.945195000

8 4.215544000 -0.210342000 -0.338721000

6 3.128233000 2.203240000 -1.880631000

6 4.714049000 -1.346245000 0.355856000

6 5.368304000 -2.317198000 -0.613570000

6 5.645607000 -0.884757000 1.447339000

1 0.316251000 -3.359035000 0.397761000

1 -0.516228000 -2.356248000 1.697982000

1 1.292835000 -0.714048000 1.586249000

1 0.468428000 -0.874171000 -1.311390000

1 2.340224000 -2.639748000 -0.575341000

1 2.981715000 -0.819635000 -1.892250000

1 2.180594000 1.098980000 0.290549000

1 -2.977299000 1.362501000 -0.034536000

1 -3.815757000 -1.943496000 1.132414000

1 -6.130492000 -2.780192000 1.205765000

1 -7.959216000 -1.500151000 0.132506000

1 -7.444862000 0.641205000 -1.010463000

1 -5.115370000 1.460129000 -1.109852000

1 1.179865000 3.418274000 2.460962000

1 -0.484949000 3.822740000 2.002089000

1 0.327280000 4.473767000 -0.290258000

1 1.986504000 4.080087000 0.201985000

1 1.128993000 5.426806000 0.966891000

1 2.945081000 3.015856000 -2.581984000

1 3.491688000 2.614884000 -0.933585000

1 3.894877000 1.539949000 -2.290357000

1 4.662628000 -2.623309000 -1.387845000

1 6.226072000 -1.842796000 -1.090964000

1 5.706925000 -3.204528000 -0.078097000

1 5.995401000 -1.741785000 2.022871000

1 6.508207000 -0.382539000 1.009324000

1 5.122440000 -0.194136000 2.108267000

**Optimized Cartesian Coordinates of the 19-TS at m06-2x/def2-TZVP/SMD/ACN level of theory**

Sum of electronic and thermal Free Energies= -1951.529659 (Hartree/Particle)

6 1.368727000 2.373630000 -1.649413000

6 0.138532000 1.760682000 -1.580602000

6 0.118375000 0.631102000 0.224648000

6 1.109404000 -0.336364000 0.206490000

6 2.479432000 0.061911000 0.176421000

16 2.846799000 1.703603000 0.252833000

6 -1.108433000 2.510246000 -1.178127000

6 -2.033792000 1.675999000 -0.247070000

6 -1.351530000 0.361750000 0.121990000

7 3.417984000 -0.899104000 -0.071641000

6 0.740273000 -1.772492000 0.050707000

8 1.258652000 -2.555978000 -0.713347000

6 4.816991000 -0.802428000 -0.003513000

6 5.566066000 -1.548141000 -0.913458000

6 6.951932000 -1.542184000 -0.851356000

6 7.607308000 -0.788352000 0.113313000

6 6.858992000 -0.053541000 1.026470000

6 5.473262000 -0.059830000 0.978076000

8 -0.209439000 -2.119308000 0.913130000

6 -0.729983000 -3.459047000 0.826723000

6 -1.625276000 -3.663251000 2.021677000

8 -1.554187000 -0.579408000 -0.939978000

8 -0.768598000 3.634155000 -0.386915000

8 -2.153961000 2.457130000 0.921900000

6 -1.774309000 3.790911000 0.599187000

6 -2.962878000 4.557028000 0.043814000

6 -1.168424000 4.435592000 1.819252000

1 1.481396000 3.404339000 -1.340079000

1 2.131287000 1.976178000 -2.303779000

1 -0.037045000 0.900791000 -2.215950000

1 0.358407000 1.573466000 0.703921000

1 -1.655082000 2.821716000 -2.073002000

1 -3.010095000 1.484434000 -0.699566000

1 -1.748646000 -0.020855000 1.061419000

1 3.064637000 -1.758466000 -0.475756000

1 5.051260000 -2.131434000 -1.668023000

1 7.518860000 -2.125891000 -1.565847000

1 8.688658000 -0.777367000 0.158434000

1 7.357633000 0.524989000 1.794286000

1 4.903311000 0.497687000 1.707735000

1 0.102572000 -4.162021000 0.818310000

1 -1.271703000 -3.557035000 -0.117968000

1 -2.418584000 -2.914493000 2.044187000

1 -1.050742000 -3.593702000 2.945952000

1 -2.081518000 -4.652310000 1.970792000

1 -3.377151000 4.050334000 -0.829641000

1 -3.740099000 4.633813000 0.804385000

1 -2.652521000 5.560704000 -0.247553000

1 -0.830963000 5.443029000 1.576112000

1 -1.912915000 4.497865000 2.612772000

1 -0.320393000 3.843281000 2.163618000

6 -2.701963000 -1.266946000 -1.047904000

8 -3.474728000 -1.122989000 0.010308000

8 -2.914959000 -1.923282000 -2.030464000

6 -4.836872000 -1.684759000 0.042850000

6 -5.315063000 -1.302510000 1.431726000

1 -6.342529000 -1.640272000 1.567605000

1 -4.689332000 -1.770861000 2.193295000

1 -5.281305000 -0.219880000 1.562905000

6 -4.796879000 -3.195144000 -0.116833000

1 -4.089876000 -3.634525000 0.588599000

1 -5.789367000 -3.589426000 0.107013000

1 -4.524966000 -3.488450000 -1.127697000

6 -5.672814000 -1.006900000 -1.028344000

1 -5.321472000 -1.259146000 -2.027452000

1 -6.706586000 -1.341437000 -0.930949000

1 -5.648523000 0.076689000 -0.899332000

**Optimized Cartesian Coordinates of the 19a-TS at m06-2x/def2-TZVP/SMD/ACN level of theory**

Sum of electronic and thermal Free Energies= -1951.522800 (Hartree/Particle)

6 1.379229000 2.874067000 0.757483000

6 0.154835000 2.246932000 0.757183000

6 0.327996000 0.601000000 -0.533316000

6 1.339298000 -0.272789000 -0.143351000

6 2.705204000 0.181238000 -0.303817000

16 2.984977000 1.755311000 -0.854104000

6 -1.016315000 2.821192000 -0.001978000

6 -1.777017000 1.746662000 -0.819868000

6 -1.169697000 0.395304000 -0.487611000

7 3.710818000 -0.640709000 0.068767000

6 1.060406000 -1.559389000 0.531561000

8 1.890646000 -2.422375000 0.753218000

6 5.103760000 -0.412927000 0.046004000

6 5.689242000 0.726124000 0.593992000

6 7.070432000 0.861295000 0.585129000

6 7.876917000 -0.134813000 0.047784000

6 7.291857000 -1.280091000 -0.477202000

6 5.911642000 -1.419302000 -0.480506000

8 -0.216022000 -1.721587000 0.864676000

6 -0.614217000 -3.005436000 1.383914000

6 -0.719427000 -4.032515000 0.280235000

8 -1.522897000 -0.585347000 -1.470999000

8 -2.003234000 3.296939000 0.900990000

8 -3.117659000 1.836829000 -0.391050000

6 -3.269043000 3.073353000 0.300549000

6 -3.608811000 4.183604000 -0.679942000

6 -4.302727000 2.906071000 1.384920000

1 1.525896000 3.784281000 0.189021000

1 2.100576000 2.672161000 1.537009000

1 -0.098561000 1.609568000 1.597808000

1 0.638012000 1.300719000 -1.302482000

1 -0.668095000 3.625810000 -0.655642000

1 -1.696142000 1.930027000 -1.895123000

1 -1.503267000 0.083040000 0.494036000

1 3.420826000 -1.588747000 0.292700000

1 5.068483000 1.491171000 1.036464000

1 7.518461000 1.749168000 1.013578000

1 8.953529000 -0.022107000 0.046045000

1 7.908921000 -2.067635000 -0.891142000

1 5.447348000 -2.307109000 -0.893210000

1 -1.582613000 -2.813482000 1.842626000

1 0.092491000 -3.311554000 2.153984000

1 0.255749000 -4.238323000 -0.159258000

1 -1.396042000 -3.691797000 -0.505287000

1 -1.116869000 -4.962047000 0.691069000

1 -2.850388000 4.255076000 -1.461522000

1 -4.571830000 3.978992000 -1.148465000

1 -3.665952000 5.138091000 -0.156063000

1 -4.426658000 3.842914000 1.928323000

1 -5.259880000 2.629281000 0.942388000

1 -3.984282000 2.126900000 2.076759000

6 -2.575069000 -1.398200000 -1.303693000

8 -3.112399000 -1.309320000 -0.103945000

6 -4.400606000 -1.945507000 0.206704000

6 -4.671834000 -1.465543000 1.621291000

1 -4.752267000 -0.377882000 1.645966000

1 -5.607707000 -1.895424000 1.979023000

1 -3.866642000 -1.775949000 2.289289000

6 -4.265583000 -3.457642000 0.169004000

1 -5.215712000 -3.897668000 0.476713000

1 -4.024641000 -3.813110000 -0.830790000

1 -3.495090000 -3.789476000 0.866802000

6 -5.464075000 -1.430050000 -0.748587000

1 -6.442084000 -1.755365000 -0.391269000

1 -5.450675000 -0.338652000 -0.770716000

1 -5.319275000 -1.811380000 -1.757289000

8 -2.919605000 -2.117055000 -2.204436000

**Optimized Cartesian Coordinates of the 16 at m06-2x/def2-TZVP/SMD/ACN level of theory**

Sum of electronic and thermal Free Energies= -1645.150347

6 -0.451690000 -2.282934000 0.998786000

6 0.585924000 -1.683143000 0.091161000

6 0.776567000 -0.189320000 0.352940000

6 -0.482212000 0.598750000 0.045997000

6 -1.740131000 0.020364000 0.063807000

16 -2.084495000 -1.642376000 0.552725000

6 1.989198000 -2.247260000 0.209359000

6 2.793852000 -1.245546000 -0.620567000

6 2.061676000 0.115936000 -0.449470000

7 -2.860471000 0.723307000 -0.239171000

6 -0.391333000 2.038181000 -0.136417000

8 -1.295564000 2.800032000 -0.470540000

6 -4.207980000 0.293728000 -0.192344000

6 -4.660262000 -0.772596000 -0.966290000

6 -5.998249000 -1.138720000 -0.922602000

6 -6.896309000 -0.430648000 -0.133332000

6 -6.449076000 0.651159000 0.615119000

6 -5.109063000 1.010485000 0.591693000

8 0.829146000 2.509435000 0.131404000

6 1.036288000 3.917702000 -0.033570000

6 2.480705000 4.204904000 0.290276000

8 2.023422000 -3.570133000 -0.257773000

6 3.304219000 -4.155964000 -0.144286000

8 4.102883000 -1.029013000 -0.129994000

8 3.004311000 0.885161000 0.277605000

6 4.288456000 0.379846000 -0.024857000

6 4.799841000 0.938066000 -1.344329000

6 5.212390000 0.672708000 1.130553000

1 -0.238664000 -2.062310000 2.045267000

1 -0.534899000 -3.362378000 0.871920000

1 0.273406000 -1.822777000 -0.951008000

1 1.055452000 -0.067787000 1.406785000

1 2.338905000 -2.203690000 1.251467000

1 2.815870000 -1.573085000 -1.664538000

1 1.833477000 0.583410000 -1.410410000

1 -2.687541000 1.718089000 -0.381679000

1 -3.968038000 -1.300538000 -1.609120000

1 -6.342056000 -1.971261000 -1.523507000

1 -7.940495000 -0.714947000 -0.108870000

1 -7.142813000 1.214208000 1.226721000

1 -4.748663000 1.845211000 1.180715000

1 0.359161000 4.456133000 0.631393000

1 0.791870000 4.193454000 -1.060750000

1 3.141795000 3.651477000 -0.376456000

1 2.710846000 3.921181000 1.317756000

1 2.671974000 5.272352000 0.172612000

1 3.217018000 -5.194004000 -0.461458000

1 3.659459000 -4.123414000 0.891691000

1 4.034659000 -3.647185000 -0.780968000

1 4.106806000 0.706271000 -2.155010000

1 4.909575000 2.020775000 -1.274678000

1 5.769949000 0.499652000 -1.580318000

1 6.194185000 0.238337000 0.941603000

1 5.324032000 1.750786000 1.250280000

1 4.799504000 0.248868000 2.045776000

**Optimized Cartesian Coordinates of the 16a at m06-2x/def2-TZVP/SMD/ACN level of theory**

Sum of electronic and thermal Free Energies= -1645.154524

6 -0.215777000 -2.572616000 -0.504948000

6 0.909335000 -1.710170000 -0.001865000

6 0.961537000 -0.360526000 -0.718532000

6 -0.282125000 0.468291000 -0.490655000

6 -1.496972000 -0.095640000 -0.171447000

16 -1.809386000 -1.832393000 -0.062001000

6 2.319581000 -2.260663000 -0.169792000

6 3.187492000 -1.023129000 0.101224000

6 2.299051000 0.210781000 -0.220429000

7 -2.586515000 0.655324000 0.149647000

6 -0.209273000 1.913605000 -0.659423000

8 -1.021740000 2.740627000 -0.265567000

6 -3.938271000 0.257029000 0.239817000

6 -4.679238000 0.677867000 1.342296000

6 -6.025492000 0.356596000 1.440885000

6 -6.639694000 -0.397959000 0.449278000

6 -5.902013000 -0.810249000 -0.654272000

6 -4.560821000 -0.474910000 -0.770248000

8 0.878417000 2.295380000 -1.341302000

6 1.104257000 3.705824000 -1.469286000

6 2.462343000 3.888568000 -2.098757000

8 2.487164000 -2.716349000 -1.495348000

6 3.622070000 -3.541788000 -1.656507000

8 3.450756000 -0.910759000 1.489560000

8 2.140652000 0.850065000 1.036611000

6 3.215497000 0.439944000 1.863628000

6 4.449128000 1.287726000 1.595493000

6 2.772450000 0.480905000 3.304264000

1 -0.165126000 -2.706026000 -1.584012000

1 -0.231865000 -3.554514000 -0.030093000

1 0.771425000 -1.522709000 1.068052000

1 1.098479000 -0.557833000 -1.788069000

1 2.543071000 -3.064655000 0.539695000

1 4.114127000 -1.040391000 -0.475993000

1 2.763008000 0.884784000 -0.936385000

1 -2.381290000 1.638739000 0.300120000

1 -4.189287000 1.254694000 2.117392000

1 -6.592150000 0.690023000 2.301212000

1 -7.687868000 -0.655724000 0.530636000

1 -6.376899000 -1.381748000 -1.441954000

1 -3.997422000 -0.766799000 -1.646581000

1 1.055582000 4.162123000 -0.479789000

1 0.312254000 4.137566000 -2.083273000

1 2.661124000 4.952311000 -2.233800000

1 2.507099000 3.402545000 -3.074175000

1 3.242730000 3.470181000 -1.461236000

1 3.682230000 -3.809966000 -2.710175000

1 3.526796000 -4.453052000 -1.056739000

1 4.544362000 -3.027526000 -1.369230000

1 4.242084000 2.330590000 1.837707000

1 4.737591000 1.223184000 0.544712000

1 5.281099000 0.940136000 2.208568000

1 1.885719000 -0.139980000 3.432002000

1 2.539136000 1.506347000 3.590991000

1 3.569373000 0.110090000 3.948932000

**Optimized Cartesian Coordinates of the 20 at m06-2x/def2-TZVP/SMD/ACN level of theory**

Sum of electronic and thermal Free Energies= -1645.154413

6 -0.072280000 -2.398604000 0.097286000

6 -0.941626000 -1.346439000 0.731386000

6 -0.936581000 -0.042501000 -0.069235000

6 0.430121000 0.602285000 -0.107719000

6 1.589661000 -0.128638000 0.014049000

16 1.666808000 -1.890360000 0.143140000

6 -2.417325000 -1.696536000 0.859584000

6 -3.143491000 -0.328409000 0.887100000

6 -2.074760000 0.742510000 0.602500000

7 2.817044000 0.457354000 0.101567000

6 0.535284000 2.044916000 -0.293387000

8 1.508166000 2.745447000 -0.045736000

6 4.090817000 -0.138738000 -0.013768000

6 4.417536000 -0.985403000 -1.072215000

6 5.695435000 -1.518234000 -1.162070000

6 6.664396000 -1.191805000 -0.220602000

6 6.346414000 -0.323789000 0.816503000

6 5.064660000 0.195750000 0.925624000

8 -0.576473000 2.582546000 -0.812719000

6 -0.598613000 4.008775000 -0.966093000

6 -1.951597000 4.380929000 -1.518319000

8 -1.591493000 1.318505000 1.796311000

8 -2.904134000 -2.311679000 -0.323498000

8 -4.055614000 -0.393446000 -0.198381000

6 -2.383711000 2.399250000 2.238011000

6 -4.179682000 -1.752644000 -0.591451000

6 -5.254020000 -2.445608000 0.231476000

6 -4.441729000 -1.814832000 -2.075144000

1 -0.368331000 -2.586206000 -0.933314000

1 -0.093172000 -3.340791000 0.646583000

1 -0.573999000 -1.132077000 1.739441000

1 -1.288430000 -0.276069000 -1.081991000

1 -2.625801000 -2.322416000 1.729197000

1 -3.662960000 -0.134678000 1.827984000

1 -2.496001000 1.512596000 -0.043689000

1 2.785680000 1.459044000 0.264548000

1 3.676657000 -1.212051000 -1.827363000

1 5.938960000 -2.177851000 -1.985594000

1 7.662230000 -1.603616000 -0.300542000

1 7.095321000 -0.055408000 1.551045000

1 4.805314000 0.862406000 1.739180000

1 0.206026000 4.306199000 -1.639860000

1 -0.416759000 4.470950000 0.005633000

1 -2.002416000 5.461165000 -1.659049000

1 -2.747291000 4.083943000 -0.833676000

1 -2.121107000 3.898964000 -2.481976000

1 -3.422140000 2.097802000 2.412037000

1 -1.958820000 2.757441000 3.174710000

1 -2.372475000 3.210864000 1.501611000

1 -6.222187000 -1.981433000 0.041851000

1 -5.308467000 -3.500525000 -0.039036000

1 -5.032259000 -2.366656000 1.297161000

1 -3.638441000 -1.308895000 -2.610704000

1 -4.494421000 -2.854060000 -2.399841000

1 -5.390031000 -1.328760000 -2.304453000

**Optimized Cartesian Coordinates of the 22 at m06-2x/def2-TZVP/SMD/ACN level of theory**

Sum of electronic and thermal Free Energies= -1645.144757

6 -0.037878000 -1.717644000 1.596407000

6 -1.053208000 -1.122946000 0.657336000

6 -0.852372000 0.378084000 0.475371000

6 0.548149000 0.768501000 0.083902000

6 1.611717000 -0.108243000 0.056394000

16 1.571396000 -1.728441000 0.764298000

6 -2.532089000 -1.200074000 1.011460000

6 -3.165364000 -0.121182000 0.097877000

6 -1.989800000 0.653610000 -0.528340000

7 2.825874000 0.249682000 -0.443927000

6 0.822424000 2.189596000 -0.114887000

8 1.741677000 2.672429000 -0.764524000

6 4.050925000 -0.451300000 -0.371182000

6 4.188525000 -1.740011000 -0.882148000

6 5.419408000 -2.379086000 -0.823662000

6 6.525081000 -1.731125000 -0.287522000

6 6.394064000 -0.433033000 0.190882000

6 5.162274000 0.203981000 0.155894000

8 -0.031268000 2.963766000 0.557256000

6 0.001552000 4.367502000 0.276560000

6 -1.133499000 5.004805000 1.037399000

8 -2.253245000 2.015437000 -0.728889000

8 -3.136447000 -2.416381000 0.614103000

8 -3.898200000 -0.881036000 -0.846703000

6 -3.238172000 2.227938000 -1.717928000

6 -4.253809000 -2.100890000 -0.212695000

6 -5.499453000 -1.921612000 0.639641000

6 -4.407569000 -3.170759000 -1.263744000

1 0.029416000 -1.159805000 2.531278000

1 -0.235886000 -2.766597000 1.819457000

1 -0.945848000 -1.611167000 -0.317251000

1 -1.131317000 0.874812000 1.413184000

1 -2.705716000 -1.007499000 2.073301000

1 -3.817329000 0.567919000 0.642416000

1 -1.737213000 0.183662000 -1.489474000

1 2.871849000 1.221764000 -0.741372000

1 3.341225000 -2.230685000 -1.343150000

1 5.516108000 -3.382843000 -1.218026000

1 7.484653000 -2.230711000 -0.252065000

1 7.251461000 0.084602000 0.602399000

1 5.048688000 1.211160000 0.538591000

1 -0.107055000 4.510055000 -0.800334000

1 0.968401000 4.772945000 0.578594000

1 -1.015150000 4.848924000 2.110540000

1 -2.087531000 4.580153000 0.723945000

1 -1.146622000 6.078123000 0.844482000

1 -3.337508000 3.304266000 -1.852200000

1 -2.943716000 1.771469000 -2.669877000

1 -4.207525000 1.815796000 -1.418453000

1 -5.341161000 -1.147980000 1.393133000

1 -6.343133000 -1.634219000 0.011360000

1 -5.738075000 -2.856935000 1.146589000

1 -5.236538000 -2.923836000 -1.927202000

1 -3.488967000 -3.247078000 -1.845171000

1 -4.614532000 -4.129398000 -0.788017000

**Optimized Cartesian Coordinates of the 21 at m06-2x/def2-TZVP/SMD/ACN level of theory**

Sum of electronic and thermal Free Energies= -1951.602583

6 1.564131000 2.655034000 -0.530622000

6 0.335637000 1.830615000 -0.806110000

6 0.064977000 0.828500000 0.318961000

6 1.153122000 -0.215596000 0.430484000

6 2.451668000 0.044802000 0.052896000

16 3.044420000 1.608034000 -0.519677000

6 -0.966101000 2.609726000 -0.934342000

6 -2.072359000 1.596382000 -0.547745000

6 -1.354994000 0.349684000 -0.008723000

7 3.411562000 -0.919624000 0.038126000

6 0.828149000 -1.540488000 0.942617000

8 1.486139000 -2.562323000 0.800499000

6 4.809732000 -0.767937000 -0.085369000

6 5.524818000 0.129114000 0.706490000

6 6.903146000 0.218676000 0.576253000

6 7.581939000 -0.600258000 -0.318259000

6 6.870930000 -1.514786000 -1.085317000

6 5.490131000 -1.595321000 -0.976414000

8 -0.317141000 -1.555663000 1.638535000

6 -0.833232000 -2.836443000 2.028957000

6 -2.089868000 -2.583486000 2.823203000

8 -1.242486000 -0.653815000 -1.028464000

8 -1.067596000 3.608883000 0.067590000

8 -2.770651000 2.221173000 0.513725000

6 -2.404955000 3.596270000 0.537859000

6 -3.309687000 4.397395000 -0.383776000

6 -2.429684000 4.086445000 1.963303000

1 1.481950000 3.175347000 0.421949000

1 1.756484000 3.386393000 -1.316528000

1 0.471752000 1.275199000 -1.739310000

1 -0.032117000 1.398190000 1.251626000

1 -1.098872000 3.044298000 -1.926427000

1 -2.743113000 1.347918000 -1.373120000

1 -1.884196000 -0.047577000 0.847519000

1 3.050750000 -1.864147000 0.135372000

1 5.002664000 0.741972000 1.429407000

1 7.450489000 0.921000000 1.192451000

1 8.658301000 -0.531864000 -0.410112000

1 7.390014000 -2.163508000 -1.779681000

1 4.927085000 -2.296653000 -1.580279000

1 -0.082487000 -3.364004000 2.617358000

1 -1.033880000 -3.419112000 1.126121000

1 -2.537626000 -3.534295000 3.114722000

1 -2.815900000 -2.025746000 2.229296000

1 -1.866004000 -2.014954000 3.726779000

1 -4.338392000 4.347010000 -0.026315000

1 -2.992084000 5.440258000 -0.402071000

1 -3.274025000 4.000220000 -1.399670000

1 -1.761680000 3.477371000 2.572248000

1 -2.107733000 5.127037000 2.002961000

1 -3.442419000 4.018497000 2.360394000

6 -2.246263000 -1.503537000 -1.265066000

8 -3.295998000 -1.269198000 -0.497188000

8 -2.132551000 -2.358514000 -2.102709000

6 -4.528375000 -2.059852000 -0.626756000

6 -5.434713000 -1.432698000 0.417882000

1 -6.400643000 -1.938004000 0.411597000

1 -4.996362000 -1.528956000 1.412362000

1 -5.590985000 -0.374599000 0.201968000

6 -4.247966000 -3.515431000 -0.294156000

1 -5.197146000 -4.051736000 -0.249329000

1 -3.618069000 -3.984937000 -1.046525000

1 -3.764860000 -3.594382000 0.681589000

6 -5.107488000 -1.877644000 -2.019553000

1 -4.485989000 -2.348456000 -2.778121000

1 -6.098413000 -2.333104000 -2.048374000

1 -5.212055000 -0.815184000 -2.248006000

**Optimized Cartesian Coordinates of the 21a at m06-2x/def2-TZVP/SMD/ACN level of theory**

Sum of electronic and thermal Free Energies= -1951.592272

6 -1.514116000 -2.049347000 -1.682858000

6 -0.251332000 -1.585790000 -1.010092000

6 -0.123155000 -0.065900000 -1.017275000

6 -1.325116000 0.639402000 -0.434460000

6 -2.507802000 0.000730000 -0.114161000

16 -2.913472000 -1.646861000 -0.608452000

6 1.089878000 -2.025784000 -1.580124000

6 2.070221000 -1.099116000 -0.833922000

6 1.229732000 0.071400000 -0.286795000

7 -3.512344000 0.639221000 0.541612000

6 -1.298852000 2.097385000 -0.360632000

8 -1.995617000 2.808047000 0.354336000

6 -4.834243000 0.194087000 0.775110000

6 -5.096643000 -0.978415000 1.479748000

6 -6.409204000 -1.360060000 1.721253000

6 -7.464248000 -0.564377000 1.292595000

6 -7.199857000 0.621396000 0.618070000

6 -5.891219000 0.998386000 0.354045000

8 -0.434853000 2.627792000 -1.227391000

6 -0.196313000 4.038311000 -1.136473000

6 0.864358000 4.380081000 -2.152083000

8 1.842096000 1.333017000 -0.565663000

8 1.455225000 -3.337755000 -1.204011000

8 2.550066000 -1.941336000 0.196657000

6 2.577935000 -3.260480000 -0.320638000

6 3.858126000 -3.507622000 -1.100051000

6 2.386175000 -4.229997000 0.819129000

1 -1.647701000 -1.586025000 -2.661204000

1 -1.553220000 -3.133584000 -1.792148000

1 -0.266961000 -1.932185000 0.029851000

1 0.044293000 0.256165000 -2.052383000

1 1.129123000 -1.912643000 -2.665544000

1 2.887475000 -0.728092000 -1.458849000

1 1.091870000 -0.043927000 0.788794000

1 -3.321629000 1.624155000 0.715496000

1 -4.276507000 -1.577022000 1.854381000

1 -6.605117000 -2.275496000 2.265290000

1 -8.485888000 -0.861578000 1.491569000

1 -8.014684000 1.253100000 0.287332000

1 -5.676326000 1.914985000 -0.182090000

1 0.129097000 4.271938000 -0.120719000

1 -1.128148000 4.572366000 -1.327133000

1 0.525483000 4.137044000 -3.160133000

1 1.781454000 3.829103000 -1.942222000

1 1.078487000 5.448781000 -2.109339000

1 3.954215000 -2.787959000 -1.914601000

1 4.720367000 -3.412499000 -0.438678000

1 3.845914000 -4.512345000 -1.523265000

1 3.222651000 -4.156876000 1.514749000

1 1.458748000 -4.000164000 1.343550000

1 2.339934000 -5.248775000 0.434231000

6 2.987850000 1.666058000 0.034161000

8 3.333335000 0.797449000 0.965777000

8 3.575389000 2.666498000 -0.286125000

6 4.615272000 0.897617000 1.672877000

6 5.751582000 0.769618000 0.672437000

1 5.795034000 1.626981000 0.003507000

1 6.694808000 0.704799000 1.216780000

1 5.631856000 -0.142032000 0.083094000

6 4.578215000 -0.304273000 2.600519000

1 3.737999000 -0.226108000 3.292396000

1 4.471729000 -1.222863000 2.022760000

1 5.502734000 -0.347666000 3.176719000

6 4.675663000 2.190884000 2.468125000

1 3.790307000 2.286453000 3.099463000

1 5.554426000 2.162317000 3.114270000

1 4.747575000 3.060007000 1.818342000

**Optimized Cartesian Coordinates of the 23 at m06-2x/def2-TZVP/SMD/ACN level of theory**

Sum of electronic and thermal Free Energies= -1645.165289 (Hartree/Particle)

C -0.204678000 2.587724000 -0.178535000

C 0.882418000 1.760183000 0.458032000

C 0.862090000 0.317067000 -0.030943000

C -0.355962000 -0.459706000 0.449603000

C -1.702774000 0.190320000 0.171514000

S -1.874009000 1.955404000 0.180137000

C 2.300367000 2.204641000 0.107542000

C 3.160713000 0.983276000 0.463230000

C 2.201437000 -0.230911000 0.467935000

O 2.788624000 -1.160933000 -0.420897000

O 4.105477000 0.658534000 -0.540087000

C 4.138357000 -0.758538000 -0.637833000

C 4.547798000 -1.144353000 -2.036030000

C 5.040474000 -1.350768000 0.432025000

N -2.711180000 -0.567232000 0.067172000

C -0.333487000 -1.848309000 -0.168097000

O -0.381527000 -2.055541000 -1.351944000

C -4.016294000 -0.058510000 -0.089119000

C -4.614392000 -0.069872000 -1.347576000

C -5.915792000 0.387814000 -1.497600000

C -6.632907000 0.844959000 -0.398072000

C -6.037771000 0.842810000 0.857642000

C -4.733980000 0.393666000 1.016632000

O -0.202589000 -2.799678000 0.749768000

C -0.101893000 -4.150651000 0.257876000

C 0.080256000 -5.055338000 1.448375000

O 2.629641000 3.376720000 0.803167000

C 3.882984000 3.900801000 0.414973000

H -0.214265000 3.608105000 0.202206000

H -0.072765000 2.620101000 -1.259965000

H 0.779984000 1.778358000 1.548685000

H 0.882878000 0.318290000 -1.125495000

H -0.292926000 -0.554442000 1.537179000

H 2.377838000 2.373693000 -0.975653000

H 3.646409000 1.135547000 1.430408000

H 2.098011000 -0.662098000 1.467355000

H 5.563635000 -0.803066000 -2.231995000

H 3.867970000 -0.690197000 -2.755507000

H 4.516662000 -2.227859000 -2.144347000

H 5.024246000 -2.438392000 0.368287000

H 6.063571000 -1.003144000 0.290821000

H 4.702622000 -1.055029000 1.426180000

H -4.051397000 -0.434506000 -2.197292000

H -6.371898000 0.383459000 -2.479176000

H -7.649163000 1.195742000 -0.518311000

H -6.589660000 1.192583000 1.720575000

H -4.265097000 0.386497000 1.992718000

H -1.010659000 -4.384741000 -0.296377000

H 0.743616000 -4.204305000 -0.427827000

H 0.160083000 -6.087717000 1.108725000

H 0.989224000 -4.799804000 1.992785000

H -0.769659000 -4.979768000 2.126319000

H 4.042332000 4.818689000 0.976987000

H 4.694915000 3.201715000 0.637255000

H 3.897095000 4.125787000 -0.656444000

**Optimized Cartesian Coordinates of the 23a at m06-2x/def2-TZVP/SMD/ACN level of theory**

Sum of electronic and thermal Free Energies= -1645.152477 (Hartree/Particle)

C -0.265237 -2.337629 0.883059

C 0.955112 -1.498473 0.591312

C 0.825638 -0.747797 -0.732838

C -0.232796 0.352882 -0.737514

C -1.618901 -0.140583 -0.339184

S -1.824121 -1.390380 0.897070

C 2.245472 -2.300857 0.402240

C 3.174701 -1.308374 -0.308713

C 2.255592 -0.312001 -1.069472

O 2.564459 0.946176 -0.489836

O 3.836378 -0.502274 0.649142

C 3.814526 0.833501 0.174369

C 4.954724 1.073846 -0.801517

C 3.833219 1.775432 1.351301

N -2.611555 0.460597 -0.849788

C 0.076278 1.519155 0.195930

O 0.145469 1.438734 1.396195

C -3.936439 0.155854 -0.476663

C -4.456257 0.631655 0.726122

C -5.778870 0.373405 1.058368

C -6.589508 -0.359181 0.199349

C -6.069512 -0.827161 -1.001800

C -4.750732 -0.566006 -1.346677

O 0.183007 2.659589 -0.475464

C 0.529876 3.819001 0.301847

C 0.733866 4.964961 -0.654765

O 1.986310 -3.407717 -0.435027

C 2.992562 -4.397744 -0.375345

H -0.366845 -3.128902 0.143245

H -0.218752 -2.792188 1.873210

H 1.124635 -0.781397 1.397059

H 0.525390 -1.474607 -1.494554

H -0.335320 0.756730 -1.743560

H 2.674686 -2.634293 1.352829

H 3.887838 -1.814827 -0.961780

H 2.440304 -0.287489 -2.144362

H 4.902599 0.370428 -1.634408

H 4.895970 2.088234 -1.196793

H 5.911236 0.946519 -0.293793

H 3.796799 2.807146 1.001244

H 4.751035 1.633757 1.922286

H 2.971575 1.579013 1.989271

H -3.818672 1.204391 1.389656

H -6.176403 0.746927 1.993992

H -7.620491 -0.559388 0.461312

H -6.694672 -1.396449 -1.678580

H -4.340857 -0.919811 -2.284752

H 1.436187 3.592843 0.865361

H -0.275352 4.018406 1.010361

H 1.541664 4.744924 -1.353823

H 0.996847 5.862468 -0.094021

H -0.176568 5.165718 -1.220502

H 3.971300 -4.004014 -0.665974

H 2.711089 -5.188418 -1.069076

H 3.066737 -4.813199 0.635024

**Optimized Cartesian Coordinates of the 25 at m06-2x/def2-TZVP/SMD/ACN level of theory**

Sum of electronic and thermal Free Energies= -1645.156837 (Hartree/Particle)

C 0.539814 -2.091462 -1.145540

C 1.407810 -0.858060 -1.209883

C 1.056862 0.155726 -0.122843

C -0.300175 0.795982 -0.370154

C -1.434381 -0.213419 -0.343788

S -1.244158 -1.721394 -1.272115

C 2.901589 -1.107653 -0.994422

C 3.451265 0.205149 -0.368882

C 2.238887 1.124365 -0.144284

N -2.511984 0.086742 0.246365

C -0.586970 1.898394 0.630595

O -0.277968 1.850041 1.793817

C -3.636370 -0.762365 0.238535

C -3.999441 -1.427299 1.407832

C -5.127508 -2.235314 1.419994

C -5.910240 -2.369634 0.278928

C -5.555673 -1.689720 -0.879901

C -4.422478 -0.888188 -0.905138

O -1.203606 2.930531 0.068533

C -1.567865 4.013602 0.945220

C -2.267100 5.056482 0.112830

O 2.068125 2.015709 -1.225162

O 3.112679 -2.074492 0.022899

O 3.982549 -0.198957 0.880952

C 2.866029 3.176653 -1.115221

C 4.170397 -1.606165 0.843028

C 5.518268 -1.945555 0.227688

C 3.997617 -2.163901 2.233060

H 0.704138 -2.626016 -0.211925

H 0.733582 -2.770910 -1.976160

H 1.291521 -0.375781 -2.184936

H 1.054371 -0.348714 0.848705

H 3.414477 -1.400096 -1.912127

H 4.211596 0.687228 -0.986525

H 2.344497 1.680080 0.792529

H -3.389332 -1.309440 2.294936

H -5.398136 -2.759069 2.328487

H -6.793347 -2.995352 0.294709

H -6.162509 -1.783042 -1.772041

H -4.142469 -0.352139 -1.804384

H -0.661642 4.404503 1.409358

H -2.214277 3.618742 1.730223

H -2.557170 5.892764 0.749582

H -3.165489 4.644895 -0.348241

H -1.608517 5.431607 -0.671306

H 2.585384 3.754103 -0.227860

H 3.931189 2.932956 -1.054406

H 2.690592 3.777514 -2.006107

H 6.320009 -1.545035 0.848534

H 5.632680 -3.027524 0.156592

H 5.603298 -1.516952 -0.772404

H 4.767517 -1.762408 2.891918

H 3.015001 -1.890776 2.617639

H 4.088294 -3.249891 2.210675

H -0.301087 1.235514 -1.370425

**Optimized Cartesian Coordinates of the 27 at m06-2x/def2-TZVP/SMD/ACN level of theory**

Sum of electronic and thermal Free Energies= -1645.155693 (Hartree/Particle)

C 0.248224 -2.065280 -1.010659

C 1.253468 -1.241920 -0.244471

C 0.840208 0.222840 -0.154919

C -0.371083 0.428648 0.745381

C -1.601267 -0.366979 0.337587

S -1.420617 -2.022910 -0.279011

C 2.643258 -1.164558 -0.883346

C 3.236345 0.182872 -0.405996

C 2.109084 0.907489 0.343387

N -2.740543 0.132553 0.568123

C -0.728237 1.899252 0.873952

O -0.747426 2.497885 1.918582

C -3.935400 -0.569103 0.312155

C -4.715552 -0.214741 -0.786698

C -5.915592 -0.870653 -1.021640

C -6.353736 -1.868117 -0.158132

C -5.580759 -2.208014 0.945506

C -4.374336 -1.563946 1.183841

O -0.995338 2.445678 -0.304628

C -1.281617 3.853958 -0.307800

C -1.515612 4.267396 -1.737661

O 2.045788 2.289329 0.106447

O 3.530352 -2.147895 -0.384930

O 4.301710 -0.203734 0.443002

C 3.096376 2.995097 0.734134

C 4.728783 -1.489177 0.015093

C 5.682876 -1.374748 -1.162470

C 5.326092 -2.222562 1.188890

H 0.177851 -1.715719 -2.041223

H 0.512094 -3.123192 -1.020486

H 1.374852 -1.639703 0.768485

H 0.620985 0.596298 -1.161767

H 2.577432 -1.217827 -1.973472

H 3.594166 0.800737 -1.234423

H 2.231419 0.714548 1.419351

H -4.370842 0.569871 -1.449352

H -6.512943 -0.597119 -1.882602

H -7.293570 -2.372949 -0.341207

H -5.916633 -2.979952 1.626850

H -3.768356 -1.821638 2.044388

H -0.432826 4.378730 0.133161

H -2.159022 4.032639 0.315530

H -0.630470 4.074742 -2.345013

H -1.735469 5.334745 -1.775533

H -2.360325 3.725263 -2.164320

H 3.071776 2.846249 1.819215

H 4.073496 2.675808 0.356564

H 2.958049 4.052000 0.512316

H 6.577390 -0.826696 -0.865077

H 5.973455 -2.369705 -1.500551

H 5.207871 -0.849635 -1.993004

H 6.222383 -1.705511 1.531729

H 4.600894 -2.267325 2.001050

H 5.598992 -3.235211 0.892015

H -0.108780 0.082628 1.750115

**Optimized Cartesian Coordinates of the 25a at m06-2x/def2-TZVP/SMD/ACN level of theory**

Sum of electronic and thermal Free Energies= -1645.152661 (Hartree/Particle)

C 0.247687 -1.839935 -1.245133

C 1.179797 -0.668299 -1.048612

C 0.999808 -0.020230 0.322457

C -0.344691 0.667872 0.549945

C -1.555296 -0.198133 0.227169

S -1.512378 -1.404260 -1.065141

C 2.670342 -1.045053 -1.055538

C 3.359748 -0.057340 -0.069462

C 2.244801 0.852474 0.459091

N -2.624578 0.065779 0.855687

C -0.502173 1.952693 -0.254121

O -0.765223 1.997827 -1.428015

C -3.838073 -0.589829 0.565085

C -4.341976 -1.539677 1.451016

C -5.559983 -2.151443 1.188409

C -6.289640 -1.811207 0.055307

C -5.791521 -0.852162 -0.818718

C -4.570242 -0.241993 -0.569243

O -0.367305 3.026454 0.519188

C -0.392399 4.294570 -0.159547

C -0.150030 5.370280 0.867074

O 2.124894 1.968295 -0.399489

O 2.874820 -2.314454 -0.452086

O 3.853038 -0.886059 0.965795

C 2.916003 3.064852 0.008100

C 3.968509 -2.200988 0.444220

C 5.285808 -2.368178 -0.295578

C 3.790329 -3.192468 1.565939

H 0.472106 -2.629144 -0.530652

H 0.319180 -2.252619 -2.252073

H 1.010949 0.079040 -1.826754

H 1.086681 -0.812774 1.074435

H 3.099428 -1.027508 -2.058303

H 4.160669 0.524020 -0.530810

H 2.430784 1.173460 1.488675

H -3.771928 -1.793101 2.336490

H -5.942089 -2.895308 1.876609

H -7.242060 -2.286192 -0.142141

H -6.355348 -0.576418 -1.701372

H -4.175767 0.508419 -1.244674

H -1.361121 4.411410 -0.646983

H 0.380217 4.283467 -0.930372

H 0.822357 5.241448 1.343685

H -0.923754 5.353359 1.635454

H -0.169296 6.345600 0.379651

H 3.970419 2.784784 0.103015

H 2.824614 3.837869 -0.753493

H 2.566929 3.455910 0.969421

H 6.118856 -2.234437 0.394973

H 5.342931 -3.366639 -0.729766

H 5.372884 -1.632373 -1.096963

H 3.822411 -4.208127 1.171672

H 4.592035 -3.075147 2.295070

H 2.830476 -3.024738 2.054524

H -0.435109 0.932116 1.602838

**Optimized Cartesian Coordinates of the 27a at m06-2x/def2-TZVP/SMD/ACN level of theory**

Sum of electronic and thermal Free Energies= -1645.152013 (Hartree/Particle)

C -0.121571 -2.369301 -0.044269

C 0.942145 -1.303638 0.054771

C 0.688750 -0.166396 -0.929724

C -0.545313 0.681509 -0.620147

C -1.824859 -0.108004 -0.380856

S -1.801608 -1.747242 0.283144

C 2.347710 -1.776557 -0.331797

C 3.064212 -0.531328 -0.909391

C 2.014284 0.588291 -0.942475

N -2.914254 0.506022 -0.589056

C -0.308085 1.552633 0.604446

O -0.323245 1.154087 1.741547

C -4.163711 -0.083585 -0.307643

C -4.623229 -0.163846 1.006139

C -5.877635 -0.696408 1.268683

C -6.679549 -1.152355 0.228907

C -6.220734 -1.065096 -1.080206

C -4.971151 -0.525970 -1.353306

O -0.058444 2.811790 0.265556

C 0.248030 3.716868 1.346326

C 0.473831 5.080063 0.746887

O 2.085993 1.430880 -2.059434

O 3.126094 -2.150238 0.789318

O 4.105584 -0.279613 0.015931

C 3.220539 2.272553 -2.028726

C 4.391188 -1.508639 0.670304

C 5.335747 -2.354320 -0.167748

C 4.926371 -1.216304 2.048864

H -0.112877 -2.821546 -1.036739

H 0.022800 -3.156614 0.696083

H 0.987144 -0.906322 1.072042

H 0.547615 -0.600991 -1.926657

H 2.300907 -2.591026 -1.059976

H 3.459572 -0.704203 -1.914353

H 2.131830 1.174735 -0.017990

H -3.992933 0.196094 1.811127

H -6.228862 -0.754982 2.291486

H -7.657240 -1.567757 0.436822

H -6.840199 -1.415014 -1.896770

H -4.610293 -0.446648 -2.371464

H 1.134331 3.345995 1.863369

H -0.587310 3.713324 2.046967

H -0.420128 5.427264 0.228005

H 1.306113 5.062342 0.042691

H 0.708989 5.788117 1.542043

H 4.149085 1.692723 -2.050419

H 3.178966 2.910109 -2.910148

H 3.216861 2.897560 -1.128751

H 4.909397 -2.543761 -1.154487

H 6.288116 -1.837695 -0.290219

H 5.512451 -3.310420 0.325556

H 5.873275 -0.681707 1.972940

H 4.209454 -0.606728 2.598460

H 5.094266 -2.149672 2.586167

H -0.745485 1.344285 -1.460288

**Optimized Cartesian Coordinates of the 26 at m06-2x/def2-TZVP/SMD/ACN level of theory**

Sum of electronic and thermal Free Energies= -1951.605305 Hartree/Particle)

6 -1.374785000 -2.470958000 -1.153289000

6 -0.073900000 -1.705409000 -1.166568000

6 -0.003373000 -0.674580000 -0.042557000

6 -0.971644000 0.478990000 -0.256948000

6 -2.422933000 0.031561000 -0.312259000

16 -2.840719000 -1.392863000 -1.293470000

6 1.177842000 -2.556791000 -0.944429000

6 2.210273000 -1.605726000 -0.276723000

6 1.472067000 -0.288657000 0.004368000

7 -3.300213000 0.748550000 0.249582000

6 -0.813076000 1.530867000 0.825757000

8 -0.647022000 1.273570000 1.990487000

6 -4.677097000 0.461342000 0.166676000

6 -5.352804000 0.030050000 1.306215000

6 -6.716653000 -0.218058000 1.246980000

6 -7.418291000 -0.018059000 0.063561000

6 -6.744740000 0.431286000 -1.065597000

6 -5.378125000 0.670549000 -1.019394000

8 -0.848418000 2.761412000 0.332807000

6 -0.719704000 3.836691000 1.283832000

6 -0.749742000 5.130376000 0.512880000

8 1.698954000 0.648622000 -1.059268000

8 0.950698000 -3.542575000 0.049633000

8 2.525061000 -2.229736000 0.951325000

6 2.101154000 -3.586191000 0.876887000

6 3.186860000 -4.444084000 0.248894000

6 1.699905000 -4.051166000 2.253316000

1 -1.468657000 -3.048738000 -0.235826000

1 -1.456758000 -3.148778000 -2.003434000

1 0.041648000 -1.190762000 -2.125230000

1 -0.231344000 -1.169113000 0.907192000

1 1.543386000 -3.014537000 -1.864641000

1 3.102966000 -1.443903000 -0.884177000

1 1.774272000 0.145332000 0.954304000

1 -4.800414000 -0.112470000 2.226874000

1 -7.234365000 -0.564300000 2.132934000

1 -8.483559000 -0.205682000 0.023724000

1 -7.283794000 0.596470000 -1.990258000

1 -4.848359000 1.026010000 -1.895353000

1 0.217713000 3.708151000 1.826132000

1 -1.543105000 3.766402000 1.995869000

1 -0.655450000 5.966326000 1.206569000

1 -1.689119000 5.236068000 -0.030621000

1 0.076300000 5.175939000 -0.197802000

1 4.075729000 -4.434734000 0.880035000

1 2.835718000 -5.471248000 0.147260000

1 3.455864000 -4.064940000 -0.738533000

1 2.562825000 -4.025546000 2.918642000

1 0.919926000 -3.399914000 2.647594000

1 1.326791000 -5.074080000 2.204081000

1 -0.760727000 0.943163000 -1.223653000

6 2.849906000 1.332136000 -1.128076000

8 3.654855000 1.049896000 -0.121555000

6 4.975551000 1.683884000 0.006710000

6 4.808578000 3.184090000 0.176079000

1 4.440484000 3.652352000 -0.734293000

1 4.118932000 3.397011000 0.995060000

1 5.778523000 3.617825000 0.423641000

6 5.520570000 1.053178000 1.275808000

1 6.515520000 1.448868000 1.480271000

1 4.872431000 1.279213000 2.123763000

1 5.590219000 -0.029968000 1.163576000

6 5.837346000 1.318927000 -1.189857000

1 5.474982000 1.785108000 -2.103492000

1 6.856127000 1.661839000 -1.003696000

1 5.859571000 0.235477000 -1.322344000

8 3.044572000 2.098513000 -2.032095000

**Optimized Cartesian Coordinates of the 26a at m06-2x/def2-TZVP/SMD/ACN level of theory**

Sum of electronic and thermal Free Energies= -1951.602628 (Hartree/Particle)

6 -1.394352000 -2.463112000 -0.971641000

6 -0.135645000 -1.897508000 -0.363504000

6 -0.135165000 -0.374165000 -0.375576000

6 -1.158267000 0.218023000 0.585623000

6 -2.587725000 -0.243507000 0.350049000

16 -2.910698000 -1.925763000 -0.115487000

6 1.151996000 -2.236247000 -1.120397000

6 2.146166000 -1.128130000 -0.707956000

6 1.312751000 -0.028432000 -0.037813000

7 -3.524913000 0.561183000 0.622198000

6 -1.097772000 1.735690000 0.589026000

8 -0.826847000 2.394084000 1.560224000

6 -4.881763000 0.191741000 0.525931000

6 -5.636471000 0.630300000 -0.560110000

6 -6.983529000 0.306784000 -0.639583000

6 -7.589191000 -0.439163000 0.365047000

6 -6.836503000 -0.862191000 1.453803000

6 -5.486267000 -0.550591000 1.538417000

8 -1.337057000 2.241219000 -0.612663000

6 -1.233347000 3.670413000 -0.740949000

6 -1.455052000 4.013190000 -2.190993000

8 1.581815000 1.284823000 -0.539808000

8 1.744618000 -3.444459000 -0.689636000

8 2.986592000 -1.792107000 0.213728000

6 3.049807000 -3.150482000 -0.187322000

6 4.081111000 -3.341811000 -1.286594000

6 3.319073000 -4.001420000 1.028037000

1 -1.472364000 -2.172676000 -2.019930000

1 -1.419473000 -3.551365000 -0.909978000

1 -0.020327000 -2.250038000 0.667087000

1 -0.340050000 -0.029231000 -1.395066000

1 0.977752000 -2.254660000 -2.199140000

1 2.714275000 -0.725267000 -1.551135000

1 1.477270000 -0.054200000 1.041211000

1 -5.157947000 1.217710000 -1.334355000

1 -7.563140000 0.643139000 -1.490344000

1 -8.641632000 -0.684206000 0.302227000

1 -7.300900000 -1.439142000 2.243955000

1 -4.894162000 -0.874099000 2.386295000

1 -0.244145000 3.976322000 -0.398069000

1 -1.981235000 4.131337000 -0.094010000

1 -0.698644000 3.540311000 -2.818613000

1 -1.386746000 5.093488000 -2.322253000

1 -2.441957000 3.686486000 -2.520740000

1 5.071751000 -3.072423000 -0.918022000

1 4.093120000 -4.384716000 -1.603963000

1 3.843274000 -2.718558000 -2.150318000

1 4.299585000 -3.760554000 1.439602000

1 2.554438000 -3.815357000 1.782002000

1 3.305215000 -5.055691000 0.751862000

1 -0.899794000 -0.106980000 1.598130000

6 2.766059000 1.864956000 -0.301190000

8 3.519714000 1.131449000 0.493472000

6 4.903675000 1.512637000 0.807013000

6 4.926600000 2.845976000 1.534866000

1 4.653045000 3.668650000 0.878258000

1 4.243392000 2.822614000 2.385887000

1 5.935867000 3.018920000 1.911589000

6 5.719210000 1.527683000 -0.474500000

1 6.769775000 1.677891000 -0.221831000

1 5.623474000 0.570770000 -0.991999000

1 5.404751000 2.329486000 -1.139948000

6 5.358398000 0.389861000 1.722508000

1 5.276544000 -0.570284000 1.212329000

1 6.396746000 0.555186000 2.010740000

1 4.744810000 0.361297000 2.624399000

8 3.028688000 2.930841000 -0.789890000

**Optimized Cartesian Coordinates of the studied conformers of 23 at m06-2x/def2-TZVP/SMD/ACN level of theory**

==============================

**conf000_E0.00**

C -0.179465 -2.513327 0.246203

C 0.887920 -1.745655 -0.490174

C 0.867986 -0.262956 -0.140817

C -0.362137 0.459040 -0.671475

C -1.695590 -0.161383 -0.280377

S -1.859965 -1.922457 -0.135543

C 2.317654 -2.146256 -0.138537

C 3.156553 -0.968144 -0.666420

C 2.197623 0.241251 -0.723323

O 2.851764 1.256403 0.003199

O 4.221165 -0.543545 0.161556

C 3.877707 0.680934 0.804888

C 3.393613 0.426364 2.223522

C 5.077184 1.600128 0.775124

N -2.698645 0.604637 -0.187667

C -0.336024 1.904892 -0.201219

O -0.304380 2.226418 0.957573

C -3.994173 0.116281 0.075583

C -4.752526 -0.462807 -0.940161

C -6.046620 -0.891181 -0.677726

C -6.591185 -0.747300 0.592631

C -5.833871 -0.163214 1.601470

C -4.542338 0.275717 1.346677

O -0.319763 2.758306 -1.219802

C -0.236408 4.161729 -0.894977

C 1.188421 4.565437 -0.596524

O 2.632127 -3.385902 -0.715653

C 3.908618 -3.854352 -0.330799

H -0.192345 -3.566066 -0.035489

H -0.024730 -2.442187 1.323626

H 0.759028 -1.867241 -1.572390

H 0.892286 -0.158861 0.947415

H -0.331476 0.438666 -1.765106

H 2.432838 -2.202041 0.953741

H 3.561971 -1.229565 -1.646079

H 2.047840 0.599207 -1.744275

H 3.067534 1.364691 2.674174

H 4.209964 0.015324 2.818761

H 2.564274 -0.278903 2.241711

H 4.822196 2.557560 1.230480

H 5.900168 1.155155 1.334633

H 5.387741 1.762661 -0.256659

H -4.322926 -0.569499 -1.929196

H -6.630752 -1.340254 -1.471465

H -7.600456 -1.082725 0.793859

H -6.251024 -0.044008 2.593782

H -3.948255 0.739521 2.124657

H -0.616284 4.667678 -1.780028

H -0.898560 4.365775 -0.054570

H 1.564381 4.046864 0.284736

H 1.836033 4.336996 -1.444088

H 1.227118 5.640177 -0.412691

H 4.045413 -4.836036 -0.781246

H 4.702920 -3.187415 -0.681260

H 3.980082 -3.943248 0.758918

==============================

**conf001_E0.06**

C 0.142525 -2.403941 -0.574072

C -0.913709 -1.792254 0.310363

C -0.869397 -0.268774 0.280007

C 0.366683 0.290329 0.972048

C 1.688880 -0.230137 0.428882

S 1.833261 -1.920584 -0.095575

C -2.348504 -2.092537 -0.112389

C -3.171274 -1.034772 0.645723

C -2.199215 0.129858 0.937120

O -2.831336 1.271802 0.403719

O -4.235714 -0.442537 -0.071703

C -3.866919 0.869757 -0.486598

C -3.386805 0.862611 -1.929583

C -5.047765 1.792603 -0.291124

N 2.695214 0.535383 0.479192

C 0.366504 1.809408 0.951988

O 0.374439 2.493067 1.943242

C 3.981087 0.113347 0.086351

C 4.748288 -0.697341 0.920966

C 6.032746 -1.061165 0.540206

C 6.559047 -0.622554 -0.668776

C 5.793348 0.191990 -1.495126

C 4.511728 0.568279 -1.119035

O 0.328294 2.284138 -0.287552

C 0.323085 3.716469 -0.444273

C -1.065512 4.283867 -0.265689

O -2.680934 -3.419043 0.201858

C -3.964490 -3.781607 -0.265082

H 0.144914 -3.492202 -0.513254

H -0.016442 -2.112273 -1.613107

H -0.786384 -2.138822 1.342813

H -0.877268 0.061806 -0.761995

H 0.334292 -0.006922 2.024696

H -2.463601 -1.924600 -1.192915

H -3.574953 -1.482868 1.555949

H -2.058523 0.289779 2.008180

H -3.050968 1.861389 -2.211208

H -4.209116 0.571531 -2.584427

H -2.566384 0.161476 -2.074944

H -5.877798 1.473158 -0.921493

H -5.358130 1.772896 0.753204

H -4.771334 2.811019 -0.566229

H 4.333268 -1.033503 1.863760

H 6.624007 -1.691305 1.193113

H 7.560734 -0.908784 -0.962593

H 6.196540 0.541526 -2.437532

H 3.911076 1.209412 -1.752717

H 1.029042 4.147425 0.264890

H 0.690278 3.879779 -1.455509

H -1.038267 5.359971 -0.444736

H -1.758275 3.830898 -0.975339

H -1.433205 4.107995 0.744488

H -4.040073 -3.641201 -1.349103

H -4.113000 -4.833653 -0.027427

H -4.749060 -3.192300 0.220354

==============================

**conf002_E0.08**

C -0.142408 -2.403898 0.574059

C 0.913843 -1.792091 -0.310275

C 0.869423 -0.268607 -0.279920

C -0.366726 0.290378 -0.971967

C -1.688868 -0.230285 -0.428832

S -1.833106 -1.920885 0.095212

C 2.348654 -2.092273 0.112455

C 3.171271 -1.034537 -0.645843

C 2.199224 0.130182 -0.936931

O 2.831299 1.272010 -0.403036

O 4.236125 -0.442362 0.071008

C 3.867333 0.869672 0.486708

C 3.387718 0.861943 1.929836

C 5.048013 1.792688 0.291141

N -2.695221 0.535222 -0.478890

C -0.366810 1.809426 -0.951964

O -0.374542 2.493051 -1.943259

C -3.981086 0.113124 -0.086210

C -4.748075 -0.697858 -0.920720

C -6.032539 -1.061749 -0.540043

C -6.559077 -0.622934 0.668754

C -5.793590 0.191903 1.495005

C -4.511980 0.568275 1.118986

O -0.328862 2.284245 0.287553

C -0.324056 3.716599 0.444171

C 1.064291 4.284447 0.265367

O 2.681192 -3.418799 -0.201813

C 3.964858 -3.781201 0.264951

H -0.144499 -3.492165 0.513345

H 0.016365 -2.112102 1.613092

H 0.786597 -2.138604 -1.342762

H 0.877303 0.062000 0.762076

H -0.334284 -0.006946 -2.024606

H 2.463804 -1.924424 1.192973

H 3.574526 -1.482756 -1.556197

H 2.058672 0.290509 -2.007949

H 3.052697 1.860779 2.212229

H 4.210022 0.569884 2.584248

H 2.566784 0.161394 2.075079

H 4.771557 2.811034 0.566478

H 5.878239 1.473303 0.921298

H 5.358189 1.773200 -0.753240

H -4.332938 -1.034237 -1.863381

H -6.623614 -1.692117 -1.192896

H -7.560763 -0.909246 0.962481

H -6.196918 0.541637 2.437276

H -3.911517 1.209650 1.752597

H -1.030274 4.147286 -0.264901

H -0.691160 3.879871 1.455454

H 1.036646 5.360569 0.444296

H 1.757313 3.831889 0.975022

H 1.432003 4.108643 -0.744809

H 4.113120 -4.833452 0.028032

H 4.749338 -3.192390 -0.221233

H 4.040855 -3.639997 1.348835

==============================

**conf003_E0.12**

C -0.142334 -2.404059 0.574039

C 0.913901 -1.792236 -0.310317

C 0.869413 -0.268762 -0.279934

C -0.366738 0.290173 -0.971996

C -1.688871 -0.230447 -0.428800

S -1.833071 -1.921036 0.095322

C 2.348720 -2.092340 0.112456

C 3.171342 -1.034523 -0.645738

C 2.199191 0.130078 -0.936954

O 2.831182 1.272017 -0.403260

O 4.235963 -0.442253 0.071420

C 3.867105 0.869921 0.486699

C 3.387414 0.862501 1.929809

C 5.047758 1.792937 0.290993

N -2.695190 0.535144 -0.478841

C -0.366817 1.809227 -0.952070

O -0.374886 2.492782 -1.943410

C -3.981124 0.113154 -0.086144

C -4.748043 -0.698068 -0.920494

C -6.032555 -1.061810 -0.539849

C -6.559207 -0.622577 0.668749

C -5.793804 0.192519 1.494818

C -4.512142 0.568748 1.118818

O -0.328475 2.284087 0.287414

C -0.323671 3.716447 0.443999

C 1.064759 4.284169 0.265355

O 2.681354 -3.418837 -0.201789

C 3.964973 -3.781192 0.265137

H -0.144450 -3.492324 0.513302

H 0.016499 -2.112299 1.613074

H 0.786674 -2.138761 -1.342794

H 0.877248 0.061824 0.762064

H -0.334276 -0.007186 -2.024621

H 2.463826 -1.924437 1.192974

H 3.574852 -1.482619 -1.556038

H 2.058590 0.290228 -2.007994

H 4.209810 0.570990 2.584350

H 2.566796 0.161622 2.075234

H 3.051966 1.861287 2.211863

H 5.877958 1.473746 0.921276

H 5.357994 1.773225 -0.753365

H 4.771211 2.811331 0.566074

H -4.332820 -1.034695 -1.863029

H -6.623593 -1.692342 -1.192576

H -7.560929 -0.908767 0.962473

H -6.197244 0.542559 2.436928

H -3.911750 1.210338 1.752276

H -1.029734 4.147158 -0.265208

H -0.690927 3.879765 1.455218

H 1.037117 5.360398 0.443638

H 1.757560 3.832009 0.975471

H 1.432744 4.107756 -0.744620

H 4.113412 -4.833368 0.027994

H 4.749471 -3.192163 -0.220756

H 4.040739 -3.640230 1.349068

==============================

**conf004_E0.15**

C 0.213332 -2.499614 0.250747

C 1.198326 -1.632730 -0.489998

C 1.031853 -0.155826 -0.150712

C -0.265776 0.431291 -0.684754

C -1.528971 -0.318046 -0.281000

S -1.516772 -2.087636 -0.144416

C 2.661463 -1.887577 -0.142591

C 3.375062 -0.640091 -0.693294

C 2.303510 0.472261 -0.743517

O 2.865234 1.546728 -0.024461

O 4.408394 -0.107900 0.110911

C 3.952300 1.073752 0.763689

C 3.511526 0.767790 2.186523

C 5.054896 2.106798 0.724964

N -2.598626 0.348635 -0.165911

C -0.408484 1.875235 -0.231172

O -0.329971 2.221359 0.918269

C -3.838566 -0.259103 0.114285

C -4.390771 -0.130515 1.387124

C -5.631493 -0.688377 1.660947

C -6.336142 -1.360164 0.668508

C -5.789767 -1.472132 -0.604225

C -4.545014 -0.925418 -0.885361

O -0.602674 2.700719 -1.254247

C -0.821702 4.090384 -0.936289

C -2.251685 4.320969 -0.506100

O 3.092161 -3.098286 -0.706146

C 4.413674 -3.431174 -0.332672

H 0.310628 -3.550833 -0.020810

H 0.354273 -2.402551 1.328107

H 1.079930 -1.772452 -1.571377

H 1.049424 -0.039162 0.936330

H -0.237601 0.402813 -1.778327

H 2.787764 -1.917263 0.949308

H 3.786647 -0.871151 -1.678054

H 2.111977 0.812183 -1.763674

H 3.097964 1.667373 2.644184

H 4.371435 0.438998 2.771490

H 2.757326 -0.017374 2.210864

H 4.714111 3.031379 1.191617

H 5.925578 1.740923 1.269371

H 5.333049 2.305832 -0.309662

H -3.838783 0.402202 2.151913

H -6.050856 -0.593247 2.654903

H -7.306434 -1.788639 0.884351

H -6.333669 -1.988729 -1.385395

H -4.115338 -1.005883 -1.876842

H -0.112975 4.390365 -0.165535

H -0.592639 4.620497 -1.858308

H -2.941682 4.005025 -1.289472

H -2.478059 3.770639 0.407195

H -2.406425 5.384332 -0.317524

H 4.509126 -3.489878 0.757201

H 4.639894 -4.403848 -0.766405

H 5.133491 -2.697057 -0.708627

==============================

**conf005_E0.23**

C 0.143952 -2.407653 0.550242

C 1.162770 -1.716531 -0.320096

C 1.006384 -0.200795 -0.290216

C -0.261358 0.269518 -0.989548

C -1.544863 -0.341056 -0.449992

S -1.574478 -2.039637 0.064485

C 2.610516 -1.907146 0.123237

C 3.362200 -0.791202 -0.625948

C 2.308160 0.293455 -0.938314

O 2.846836 1.485856 -0.412693

O 4.364889 -0.116516 0.107682

C 3.899090 1.173312 0.494482

C 3.402852 1.158381 1.931840

C 5.014577 2.173377 0.293992

N -2.601285 0.353675 -0.497850

C -0.369743 1.783675 -0.963440

O -0.417253 2.471494 -1.950434

C -3.852454 -0.159278 -0.099752

C -4.358680 0.156899 1.159385

C -5.610027 -0.309285 1.536727

C -6.368044 -1.079466 0.662161

C -5.864547 -1.382863 -0.596943

C -4.611206 -0.926156 -0.981598

O -0.363345 2.251205 0.279806

C -0.516798 3.673096 0.447540

C -1.971380 4.069557 0.346082

O 3.045872 -3.205372 -0.182681

C 4.346430 -3.470235 0.302364

H 0.218815 -3.492794 0.481446

H 0.273920 -2.114253 1.592775

H 1.075947 -2.070411 -1.354238

H 0.981134 0.128143 0.751939

H -0.203701 -0.023010 -2.042381

H 2.697227 -1.728401 1.204860

H 3.814505 -1.209301 -1.527580

H 2.165240 0.431061 -2.012208

H 2.976745 2.130543 2.183305

H 4.238141 0.956287 2.603518

H 2.645154 0.391422 2.085082

H 4.667399 3.173947 0.553251

H 5.859757 1.920052 0.934178

H 5.333693 2.160801 -0.747819

H -3.763258 0.760967 1.833161

H -5.995086 -0.066443 2.519421

H -7.345872 -1.436635 0.958639

H -6.449227 -1.979176 -1.286509

H -4.213077 -1.156189 -1.962741

H -0.117004 3.876449 1.438914

H 0.095121 4.183164 -0.295286

H -2.370091 3.840395 -0.642228

H -2.563982 3.544743 1.096481

H -2.067976 5.142526 0.518516

H 4.396557 -3.323556 1.387000

H 4.576507 -4.508490 0.068808

H 5.091054 -2.824327 -0.173787

==============================

**conf006_E0.25**

C 0.143973 -2.407628 0.550288

C 1.162781 -1.716518 -0.320069

C 1.006397 -0.200780 -0.290214

C -0.261359 0.269527 -0.989525

C -1.544860 -0.341064 -0.449969

S -1.574461 -2.039627 0.064544

C 2.610531 -1.907129 0.123253

C 3.362207 -0.791204 -0.625964

C 2.308171 0.293455 -0.938329

O 2.846863 1.485861 -0.412728

O 4.364919 -0.116517 0.107632

C 3.899144 1.173322 0.494424

C 3.402956 1.158431 1.931799

C 5.014629 2.173371 0.293870

N -2.601285 0.353665 -0.497858

C -0.369757 1.783686 -0.963395

O -0.417256 2.471517 -1.950381

C -3.852465 -0.159291 -0.099776

C -4.358697 0.156859 1.159360

C -5.610056 -0.309327 1.536680

C -6.368065 -1.079485 0.662092

C -5.864557 -1.382857 -0.597019

C -4.611215 -0.926144 -0.981653

O -0.363395 2.251194 0.279860

C -0.516902 3.673077 0.447632

C -1.971501 4.069480 0.346148

O 3.045877 -3.205363 -0.182648

C 4.346439 -3.470222 0.302390

H 0.218841 -3.492769 0.481504

H 0.273953 -2.114215 1.592816

H 1.075950 -2.070419 -1.354203

H 0.981165 0.128167 0.751937

H -0.203717 -0.022983 -2.042360

H 2.697254 -1.728367 1.204871

H 3.814492 -1.209328 -1.527593

H 2.165240 0.431054 -2.012222

H 4.238274 0.956389 2.603458

H 2.645287 0.391456 2.085101

H 2.976830 2.130593 2.183240

H 5.333706 2.160771 -0.747954

H 4.667467 3.173948 0.553124

H 5.859832 1.920051 0.934031

H -3.763286 0.760921 1.833153

H -5.995127 -0.066496 2.519373

H -7.345896 -1.436663 0.958553

H -6.449240 -1.979149 -1.286603

H -4.213095 -1.156124 -1.962812

H -0.117134 3.876408 1.439018

H 0.095012 4.183193 -0.295162

H -2.370170 3.840301 -0.642178

H -2.564099 3.544627 1.096524

H -2.068163 5.142445 0.518581

H 4.576529 -4.508469 0.068816

H 5.091057 -2.824299 -0.173751

H 4.396569 -3.323562 1.387027

==============================

**conf007_E0.25**

C 0.143844 -2.407566 0.550460

C 1.162658 -1.716563 -0.319977

C 1.006378 -0.200816 -0.290168

C -0.261391 0.269581 -0.989393

C -1.544905 -0.340959 -0.449830

S -1.574585 -2.039486 0.064767

C 2.610412 -1.907219 0.123309

C 3.362121 -0.791383 -0.626007

C 2.308130 0.293309 -0.938396

O 2.846895 1.485725 -0.412893

O 4.364888 -0.116711 0.107530

C 3.899230 1.173209 0.494208

C 3.403120 1.158495 1.931610

C 5.014777 2.173159 0.293474

N -2.601316 0.353790 -0.497791

C -0.369666 1.783746 -0.963195

O -0.416980 2.471638 -1.950149

C -3.852521 -0.159203 -0.099833

C -4.611037 -0.926293 -0.981709

C -5.864400 -1.383055 -0.597220

C -6.368162 -1.079513 0.661749

C -5.610388 -0.309125 1.536331

C -4.359009 0.157123 1.159149

O -0.363347 2.251199 0.280080

C -0.516610 3.673106 0.447872

C -1.971108 4.069811 0.346192

O 3.045698 -3.205497 -0.182491

C 4.346271 -3.470358 0.302519

H 0.218646 -3.492715 0.481738

H 0.273887 -2.114099 1.592966

H 1.075763 -2.070499 -1.354094

H 0.981258 0.128168 0.751976

H -0.203804 -0.022883 -2.042248

H 2.697175 -1.728367 1.204911

H 3.814356 -1.209572 -1.527633

H 2.165154 0.430840 -2.012292

H 2.977138 2.130730 2.183006

H 4.238439 0.956385 2.603245

H 2.645349 0.391635 2.084989

H 4.667711 3.173794 0.552632

H 5.860000 1.919858 0.933616

H 5.333797 2.160416 -0.748363

H -4.212704 -1.156430 -1.962744

H -6.448904 -1.979523 -1.286801

H -7.346009 -1.436738 0.958096

H -5.995650 -0.066160 2.518915

H -3.763772 0.761340 1.832954

H -0.116949 3.876338 1.439324

H 0.095521 4.183113 -0.294822

H -2.369690 3.840796 -0.642203

H -2.563923 3.545036 1.096450

H -2.067566 5.142783 0.518700

H 4.576207 -4.508711 0.069262

H 5.090924 -2.824681 -0.173902

H 4.396515 -3.323350 1.387104

==============================

**conf008_E0.28**

C 0.258085 -2.421636 -0.642374

C -0.843269 -1.826836 0.198078

C -0.833909 -0.303151 0.158846

C 0.374277 0.298765 0.863636

C 1.725291 -0.210602 0.384931

S 1.919393 -1.906331 -0.101395

C -2.253594 -2.158674 -0.284876

C -3.131104 -1.094110 0.390966

C -2.181542 0.061903 0.784164

O -2.760984 1.219438 0.215486

O -4.043497 -0.483512 -0.503872

C -4.094462 0.896169 -0.169253

C -4.455480 1.686685 -1.401113

C -5.047026 1.134403 0.990598

N 2.716649 0.571052 0.470986

C 0.344411 1.816188 0.794450

O 0.271656 2.530066 1.761364

C 4.026538 0.162552 0.149847

C 4.770197 -0.600516 1.048200

C 6.078827 -0.947896 0.741796

C 6.652661 -0.540400 -0.456397

C 5.909606 0.225674 -1.347228

C 4.603548 0.585053 -1.046069

O 0.380995 2.249762 -0.459868

C 0.362989 3.675180 -0.667545

C -1.047791 4.214039 -0.639911

O -2.582399 -3.482073 0.042457

C -3.822640 -3.881769 -0.504430

H 0.277597 -3.509434 -0.576596

H 0.133582 -2.137451 -1.688021

H -0.750019 -2.165667 1.236504

H -0.850206 0.018596 -0.887604

H 0.315069 0.034118 1.923812

H -2.316331 -2.011333 -1.372711

H -3.648456 -1.523483 1.253453

H -2.091941 0.166574 1.869545

H -3.735669 1.480362 -2.193013

H -4.446163 2.752377 -1.172309

H -5.454690 1.411179 -1.738868

H -6.059214 0.844307 0.707034

H -4.744876 0.552138 1.862915

H -5.043716 2.191019 1.259335

H 4.318073 -0.911710 1.982485

H 6.651487 -1.540366 1.444634

H 7.673340 -0.812894 -0.692123

H 6.349705 0.550474 -2.281957

H 4.020214 1.189005 -1.730455

H 0.990186 4.146227 0.088394

H 0.820327 3.813334 -1.645323

H -1.506019 4.059336 0.336220

H -1.026968 5.284509 -0.850829

H -1.659225 3.722676 -1.397175

H -3.981966 -4.922393 -0.227091

H -4.646988 -3.277989 -0.110773

H -3.813796 -3.795161 -1.596484

==============================

**conf009_E0.31**

C -0.258071 -2.421659 0.642411

C 0.843279 -1.826960 -0.198127

C 0.833969 -0.303285 -0.159055

C -0.374258 0.298660 -0.863769

C -1.725254 -0.210659 -0.384985

S -1.919374 -1.906354 0.101449

C 2.253601 -2.158779 0.284865

C 3.131157 -1.094132 -0.390828

C 2.181547 0.061623 -0.784568

O 2.760881 1.219505 -0.216481

O 4.042997 -0.483184 0.504366

C 4.094112 0.896375 0.169353

C 4.454171 1.687300 1.401237

C 5.047467 1.134301 -0.989905

N -2.716589 0.571029 -0.471028

C -0.344307 1.816074 -0.794597

O -0.271529 2.529946 -1.761520

C -4.026488 0.162610 -0.149844

C -4.770196 -0.600542 -1.048091

C -6.078825 -0.947827 -0.741619

C -6.652633 -0.540148 0.456531

C -5.909546 0.226021 1.347237

C -4.603472 0.585302 1.046006

O -0.380820 2.249664 0.459724

C -0.362606 3.675077 0.667425

C 1.048275 4.213661 0.639962

O 2.582494 -3.482146 -0.042583

C 3.822580 -3.881927 0.504596

H -0.277600 -3.509462 0.576719

H -0.133512 -2.137390 1.688030

H 0.750018 -2.165904 -1.236515

H 0.850411 0.018584 0.887358

H -0.315102 0.033975 -1.923944

H 2.316288 -2.011548 1.372712

H 3.649027 -1.523530 -1.252988

H 2.091892 0.165785 -1.869992

H 3.733821 1.481121 2.192682

H 4.444884 2.752928 1.172120

H 5.453179 1.412051 1.739802

H 6.059494 0.844398 -0.705567

H 4.745960 0.551686 -1.862211

H 5.044266 2.190813 -1.259047

H -4.318118 -0.911857 -1.982355

H -6.651526 -1.540364 -1.444365

H -7.673317 -0.812585 0.692295

H -6.349603 0.550977 2.281929

H -4.020122 1.189346 1.730295

H -0.989615 4.146260 -0.088585

H -0.820059 3.813305 1.645143

H 1.027667 5.284152 0.850809

H 1.659539 3.722214 1.397307

H 1.506581 4.058808 -0.336109

H 3.982038 -4.922472 0.227034

H 4.647031 -3.278021 0.111342

H 3.813385 -3.795597 1.596666

==============================

**conf010_E0.31**

C -0.258062 -2.421718 0.642335

C 0.843283 -1.826970 -0.198181

C 0.833942 -0.303302 -0.159055

C -0.374284 0.298642 -0.863791

C -1.725263 -0.210646 -0.384976

S -1.919383 -1.906374 0.101426

C 2.253601 -2.158791 0.284815

C 3.131148 -1.094109 -0.390835

C 2.181526 0.061669 -0.784511

O 2.760831 1.219510 -0.216300

O 4.043002 -0.483189 0.504367

C 4.094088 0.896380 0.169407

C 4.454243 1.687253 1.401293

C 5.047359 1.134369 -0.989910

N -2.716593 0.571047 -0.470997

C -0.344301 1.816050 -0.794644

O -0.271531 2.529909 -1.761575

C -4.026505 0.162643 -0.149794

C -4.603425 0.585225 1.046118

C -5.909507 0.225958 1.347350

C -6.652654 -0.540078 0.456580

C -6.078903 -0.947649 -0.741638

C -4.770270 -0.600382 -1.048109

O -0.380762 2.249655 0.459673

C -0.362507 3.675060 0.667344

C 1.048411 4.213560 0.639969

O 2.582503 -3.482137 -0.042689

C 3.822543 -3.881963 0.504563

H -0.277617 -3.509516 0.576572

H -0.133493 -2.137506 1.687966

H 0.750032 -2.165896 -1.236574

H 0.850337 0.018529 0.887370

H -0.315121 0.033942 -1.923958

H 2.316282 -2.011594 1.372667

H 3.649006 -1.523466 -1.253020

H 2.091912 0.165917 -1.869928

H 5.453265 1.411957 1.739782

H 3.733939 1.481064 2.192781

H 4.444967 2.752891 1.172220

H 6.059408 0.844455 -0.705657

H 4.745798 0.551812 -1.862238

H 5.044134 2.190901 -1.258983

H -4.020030 1.189169 1.730459

H -6.349525 0.550825 2.282092

H -7.673344 -0.812492 0.692349

H -6.651653 -1.540088 -1.444427

H -4.318219 -0.911607 -1.982417

H -0.989416 4.146261 -0.088736

H -0.820045 3.813336 1.645014

H 1.659581 3.722152 1.397416

H 1.506792 4.058557 -0.336048

H 1.027838 5.284076 0.850685

H 3.982090 -4.922447 0.226824

H 4.647013 -3.277942 0.111520

H 3.813215 -3.795838 1.596647

==============================

**conf011_E0.34**

C -0.330183 -2.541056 0.297193

C 0.783536 -1.790938 -0.387473

C 0.797687 -0.315672 -0.004357

C -0.391427 0.451897 -0.565682

C -1.755922 -0.131707 -0.233177

S -1.979178 -1.888020 -0.117526

C 2.186700 -2.246989 0.002871

C 3.080274 -1.082686 -0.447322

C 2.160356 0.161204 -0.513618

O 2.771757 1.104877 0.344070

O 4.059844 -0.729342 0.511906

C 4.118335 0.690662 0.547017

C 4.565271 1.130229 1.917679

C 5.006301 1.219234 -0.567421

N -2.740179 0.661273 -0.173037

C -0.332883 1.891947 -0.082169

O -0.352792 2.206467 1.078430

C -4.057026 0.203868 0.035564

C -4.630085 0.310170 1.301153

C -5.940949 -0.098505 1.501782

C -6.691771 -0.600918 0.445126

C -6.121210 -0.692899 -0.818585

C -4.808361 -0.292936 -1.027864

O -0.218607 2.748243 -1.092714

C -0.078602 4.143717 -0.754188

C 1.349871 4.469039 -0.385317

O 2.482956 -3.479874 -0.596029

C 3.724991 -4.002898 -0.170486

H -0.367193 -3.585618 -0.011474

H -0.205914 -2.501315 1.380009

H 0.686507 -1.884814 -1.475521

H 0.798415 -0.235455 1.087892

H -0.321248 0.441618 -1.657487

H 2.258526 -2.330059 1.097183

H 3.537722 -1.314144 -1.413472

H 2.082850 0.554757 -1.531771

H 4.555924 2.218628 1.977797

H 5.579242 0.778285 2.107789

H 3.892332 0.721232 2.670886

H 5.007065 2.309586 -0.553350

H 6.027515 0.862311 -0.431044

H 4.643596 0.883031 -1.540475

H -4.039916 0.708874 2.117317

H -6.378121 -0.020513 2.489552

H -7.715675 -0.913663 0.604602

H -6.699788 -1.078564 -1.648944

H -4.357908 -0.359585 -2.011078

H -0.388189 4.673427 -1.652676

H -0.768587 4.378419 0.055155

H 2.023577 4.213926 -1.204652

H 1.436676 5.538442 -0.187361

H 1.656261 3.922471 0.505973

H 4.553894 -3.346564 -0.454845

H 3.740264 -4.137655 0.916646

H 3.855585 -4.969200 -0.654644

==============================

**conf012_E0.47**

C 0.017494 -2.456907 0.558885

C 1.085221 -1.753750 -0.240678

C 0.960182 -0.236589 -0.156141

C -0.274057 0.286301 -0.878587

C -1.588820 -0.314871 -0.409228

S -1.672431 -2.034727 0.021937

C 2.507353 -1.995063 0.259762

C 3.314558 -0.854138 -0.377065

C 2.291056 0.251406 -0.732028

O 2.778255 1.415097 -0.091851

O 4.189983 -0.218811 0.536849

C 4.136110 1.175041 0.266294

C 4.451012 1.932726 1.530822

C 5.056097 1.535280 -0.888475

N -2.627218 0.406188 -0.461797

C -0.351048 1.800784 -0.810314

O -0.336070 2.517234 -1.778113

C -3.903569 -0.095579 -0.136043

C -4.635469 -0.820235 -1.074691

C -5.912083 -1.265888 -0.760571

C -6.465897 -0.993294 0.484446

C -5.734718 -0.264765 1.415728

C -4.460317 0.190616 1.108889

O -0.393504 2.232113 0.444443

C -0.517594 3.652240 0.646700

C -1.954415 4.091960 0.486829

O 2.935347 -3.283484 -0.091391

C 4.196004 -3.604328 0.460752

H 0.074389 -3.539696 0.448123

H 0.110923 -2.208743 1.616811

H 1.036066 -2.064847 -1.290751

H 0.921752 0.053697 0.898781

H -0.184968 0.026133 -1.937734

H 2.542043 -1.868737 1.351613

H 3.855202 -1.220133 -1.254486

H 2.216335 0.405954 -1.812607

H 3.763660 1.629439 2.320236

H 4.348856 3.003542 1.354633

H 5.474901 1.725944 1.842394

H 6.089424 1.305769 -0.626742

H 4.787194 0.973877 -1.785072

H 4.974427 2.600219 -1.107282

H -4.198507 -1.026336 -2.044596

H -6.475411 -1.829180 -1.494286

H -7.461777 -1.341834 0.726014

H -6.159102 -0.045887 2.387807

H -3.885763 0.762567 1.827318

H -0.161687 3.816236 1.661731

H 0.143505 4.165874 -0.050161

H -2.030801 5.161883 0.686248

H -2.310130 3.902358 -0.525944

H -2.597049 3.562764 1.191578

H 4.978753 -2.936848 0.085729

H 4.171750 -3.538511 1.554044

H 4.430745 -4.626058 0.166979

==============================

**conf013_E0.48**

C 0.067744 -2.551534 0.305241

C 1.103830 -1.699549 -0.381370

C 0.977173 -0.227195 -0.005335

C -0.284405 0.414386 -0.564437

C -1.584745 -0.302669 -0.226284

S -1.634551 -2.073716 -0.131218

C 2.544478 -2.017418 0.007069

C 3.320519 -0.776506 -0.455784

C 2.284962 0.373031 -0.529824

O 2.810932 1.383333 0.308770

O 4.263158 -0.322240 0.497569

C 4.190514 1.097178 0.513273

C 4.598303 1.594350 1.876527

C 5.024376 1.689416 -0.610887

N -2.636546 0.395975 -0.138942

C -0.396915 1.852866 -0.086127

O -0.367546 2.173089 1.072730

C -3.906487 -0.176916 0.071388

C -4.518724 -0.045031 1.316129

C -5.788136 -0.567239 1.519918

C -6.461463 -1.205981 0.484922

C -5.854671 -1.321143 -0.759888

C -4.581093 -0.810560 -0.970713

O -0.502050 2.705934 -1.100243

C -0.676809 4.097330 -0.763447

C -2.116512 4.383808 -0.405272

O 2.954730 -3.220984 -0.584481

C 4.242036 -3.620737 -0.160092

H 0.140253 -3.598024 0.008964

H 0.178155 -2.486755 1.388395

H 1.015566 -1.806149 -1.469130

H 0.977961 -0.139474 1.086179

H -0.217919 0.408578 -1.656646

H 2.627839 -2.085643 1.101519

H 3.796018 -0.971406 -1.421391

H 2.161836 0.742991 -1.552366

H 4.485729 2.677475 1.923624

H 5.641809 1.342846 2.066308

H 3.969991 1.132656 2.637771

H 6.073878 1.427303 -0.474088

H 4.690114 1.309812 -1.578069

H 4.927527 2.775388 -0.610380

H -3.990851 0.462710 2.114148

H -6.254898 -0.469593 2.492263

H -7.454293 -1.605966 0.645961

H -6.373599 -1.812047 -1.573898

H -4.103566 -0.894003 -1.939816

H 0.001313 4.349374 0.050787

H -0.374863 4.635113 -1.659700

H -2.776155 4.115342 -1.231344

H -2.416262 3.825835 0.481847

H -2.234214 5.449044 -0.201163

H 4.462008 -4.573998 -0.637715

H 5.004121 -2.891094 -0.452689

H 4.273509 -3.745076 0.927901

==============================

**conf014_E0.49**

C 0.067660 -2.551446 0.305432

C 1.103726 -1.699498 -0.381248

C 0.977109 -0.227142 -0.005205

C -0.284450 0.414462 -0.564309

C -1.584816 -0.302611 -0.226244

S -1.634642 -2.073613 -0.130974

C 2.544398 -2.017401 0.007054

C 3.320412 -0.776560 -0.456013

C 2.284915 0.373090 -0.529618

O 2.811046 1.383032 0.309348

O 4.263515 -0.322434 0.496944

C 4.190733 1.096998 0.513060

C 4.599212 1.593826 1.876237

C 5.023985 1.689586 -0.611364

N -2.636623 0.396058 -0.139049

C -0.396926 1.852918 -0.085928

O -0.367564 2.173071 1.072951

C -3.906568 -0.176868 0.071246

C -4.581278 -0.810159 -0.971001

C -5.854848 -1.320783 -0.760226

C -6.461531 -1.206004 0.484674

C -5.788099 -0.567614 1.519817

C -4.518687 -0.045378 1.316084

O -0.502000 2.706052 -1.099994

C -0.676641 4.097449 -0.763109

C -2.116314 4.384043 -0.404928

O 2.954505 -3.221024 -0.584507

C 4.241812 -3.620860 -0.160196

H 0.140139 -3.597946 0.009186

H 0.178118 -2.486636 1.388580

H 1.015391 -1.806101 -1.469003

H 0.977885 -0.139449 1.086310

H -0.217951 0.408703 -1.656516

H 2.627911 -2.085575 1.101495

H 3.795478 -0.971470 -1.421833

H 2.161806 0.743446 -1.552014

H 4.486767 2.676950 1.923654

H 5.642790 1.342183 2.065443

H 3.971242 1.131997 2.637678

H 4.689407 1.310086 -1.578478

H 4.926930 2.775539 -0.610613

H 6.073599 1.427657 -0.475063

H -4.103841 -0.893305 -1.940173

H -6.373862 -1.811413 -1.574347

H -7.454359 -1.606015 0.645665

H -6.254770 -0.470272 2.492235

H -3.990726 0.462084 2.114224

H 0.001513 4.349377 0.051134

H -0.374641 4.635248 -1.659334

H -2.775975 4.115703 -1.231029

H -2.416136 3.826046 0.482149

H -2.233913 5.449278 -0.200753

H 4.462044 -4.573723 -0.638493

H 5.003813 -2.890863 -0.452126

H 4.273120 -3.745994 0.927710

==============================

**conf015_E0.49**

C 0.067708 -2.551533 0.305008

C 1.103823 -1.699492 -0.381490

C 0.977151 -0.227160 -0.005362

C -0.284391 0.414457 -0.564504

C -1.584742 -0.302604 -0.226395

S -1.634572 -2.073655 -0.131435

C 2.544448 -2.017380 0.007020

C 3.320506 -0.776475 -0.455816

C 2.284974 0.373088 -0.529731

O 2.810958 1.383260 0.309022

O 4.263256 -0.322293 0.497456

C 4.190568 1.097139 0.513317

C 4.598529 1.594159 1.876573

C 5.024279 1.689514 -0.610882

N -2.636544 0.396026 -0.138987

C -0.396916 1.852927 -0.086172

O -0.367408 2.173145 1.072683

C -3.906451 -0.176916 0.071410

C -4.581306 -0.810115 -0.970799

C -5.854835 -1.320781 -0.759885

C -6.461332 -1.206142 0.485118

C -5.787754 -0.567845 1.520225

C -4.518384 -0.045564 1.316356

O -0.502268 2.705993 -1.100269

C -0.677105 4.097371 -0.763428

C -2.116770 4.383705 -0.404998

O 2.954719 -3.220968 -0.584474

C 4.242074 -3.620633 -0.160148

H 0.140223 -3.597994 0.008631

H 0.178094 -2.486862 1.388172

H 1.015621 -1.806021 -1.469261

H 0.977869 -0.139515 1.086156

H -0.217864 0.408664 -1.656709

H 2.627743 -2.085585 1.101476

H 3.795899 -0.971344 -1.421483

H 2.161894 0.743192 -1.552226

H 3.970208 1.132503 2.637833

H 4.486144 2.677299 1.923768

H 5.642009 1.342462 2.066245

H 4.927326 2.775475 -0.610303

H 6.073819 1.427494 -0.474185

H 4.689986 1.309928 -1.578062

H -4.104001 -0.893161 -1.940045

H -6.373957 -1.811348 -1.573975

H -7.454127 -1.606189 0.646223

H -6.254277 -0.470615 2.492726

H -3.990311 0.461822 2.114469

H 0.001140 4.349472 0.050685

H -0.375373 4.635190 -1.659732

H -2.776532 4.115180 -1.230957

H -2.416316 3.825707 0.482173

H -2.234535 5.448932 -0.200872

H 4.273702 -3.744641 0.927878

H 4.461942 -4.574050 -0.637508

H 5.004142 -2.891107 -0.453081

==============================

**conf016_E0.50**

C 0.067728 -2.551588 0.304928

C 1.103814 -1.699462 -0.381517

C 0.977098 -0.227158 -0.005276

C -0.284439 0.414435 -0.564478

C -1.584765 -0.302658 -0.226359

S -1.634574 -2.073690 -0.131407

C 2.544447 -2.017334 0.006945

C 3.320459 -0.776411 -0.455906

C 2.284936 0.373180 -0.529500

O 2.810962 1.383097 0.309560

O 4.263423 -0.322363 0.497225

C 4.190642 1.097055 0.513412

C 4.598930 1.593831 1.876663

C 5.024037 1.689730 -0.610875

N -2.636570 0.395981 -0.138936

C -0.396974 1.852906 -0.086173

O -0.367495 2.173137 1.072681

C -3.906488 -0.176954 0.071445

C -4.581313 -0.810254 -0.970714

C -5.854864 -1.320874 -0.759790

C -6.461404 -1.206086 0.485174

C -5.787855 -0.567679 1.520238

C -4.518473 -0.045442 1.316354

O -0.502279 2.705951 -1.100291

C -0.677005 4.097363 -0.763510

C -2.116698 4.383886 -0.405337

O 2.954716 -3.220902 -0.584604

C 4.242113 -3.620521 -0.160376

H 0.140251 -3.598014 0.008433

H 0.178149 -2.487025 1.388094

H 1.015598 -1.805907 -1.469295

H 0.977710 -0.139592 1.086247

H -0.217874 0.408620 -1.656679

H 2.627797 -2.085575 1.101394

H 3.795650 -0.971193 -1.421691

H 2.161878 0.743594 -1.551884

H 5.642482 1.342188 2.066007

H 3.970856 1.131975 2.638006

H 4.486461 2.676952 1.924101

H 6.073627 1.427758 -0.474469

H 4.689563 1.310311 -1.578058

H 4.927003 2.775683 -0.610053

H -4.103986 -0.893416 -1.939940

H -6.373966 -1.811516 -1.573848

H -7.454212 -1.606096 0.646285

H -6.254417 -0.470333 2.492708

H -3.990418 0.462036 2.114420

H 0.001127 4.349397 0.050717

H -0.375050 4.635121 -1.659775

H -2.416460 3.825928 0.481787

H -2.234373 5.449128 -0.201237

H -2.776350 4.115431 -1.231406

H 4.461936 -4.573981 -0.637671

H 5.004147 -2.891017 -0.453454

H 4.273861 -3.744431 0.927659

==============================

**conf017_E0.51**

C 0.017442 -2.456903 0.558993

C 1.085157 -1.753781 -0.240615

C 0.960132 -0.236613 -0.156109

C -0.274133 0.286304 -0.878486

C -1.588877 -0.314851 -0.409069

S -1.672489 -2.034663 0.022171

C 2.507298 -1.995027 0.259832

C 3.314489 -0.854225 -0.377241

C 2.291003 0.251392 -0.732009

O 2.778263 1.414969 -0.091672

O 4.190247 -0.218984 0.536388

C 4.136228 1.174928 0.266074

C 4.451413 1.932405 1.530657

C 5.055885 1.535377 -0.888882

N -2.627278 0.406217 -0.461732

C -0.351024 1.800797 -0.810162

O -0.335909 2.517271 -1.777939

C -3.903664 -0.095561 -0.136073

C -4.460736 0.191062 1.108596

C -5.735184 -0.264329 1.415297

C -6.466058 -0.993264 0.484118

C -5.911911 -1.266284 -0.760670

C -4.635268 -0.820646 -1.074639

O -0.393535 2.232085 0.444606

C -0.517398 3.652240 0.646885

C -1.954132 4.092192 0.486921

O 2.935291 -3.283532 -0.091052

C 4.196072 -3.604163 0.460934

H 0.074307 -3.539691 0.448209

H 0.110961 -2.208774 1.616923

H 1.035993 -2.064902 -1.290677

H 0.921765 0.053684 0.898813

H -0.185098 0.026173 -1.937647

H 2.542007 -1.868480 1.351658

H 3.854841 -1.220353 -1.254791

H 2.216251 0.406103 -1.812563

H 3.764154 1.629071 2.320129

H 4.349360 3.003260 1.354644

H 5.475327 1.725459 1.842030

H 6.089311 1.306003 -0.627424

H 4.786847 0.973971 -1.785436

H 4.974007 2.600315 -1.107607

H -3.886426 0.763331 1.826962

H -6.159818 -0.045107 2.387189

H -7.461969 -1.341808 0.725551

H -6.475029 -1.829892 -1.494303

H -4.198040 -1.027068 -2.044353

H -0.161525 3.816151 1.661943

H 0.143835 4.165763 -0.049935

H -2.030390 5.162102 0.686445

H -2.309780 3.902733 -0.525899

H -2.596910 3.563026 1.191561

H 4.430567 -4.626137 0.167817

H 4.978798 -2.937057 0.085203

H 4.172165 -3.537562 1.554187

==============================

**conf018_E0.51**

C -0.017443 -2.456952 -0.558926

C -1.085176 -1.753840 0.240667

C -0.960167 -0.236669 0.156239

C 0.274102 0.286183 0.878676

C 1.588830 -0.314904 0.409150

S 1.672461 -2.034763 -0.021996

C -2.507315 -1.995120 -0.259748

C -3.314541 -0.854207 0.377097

C -2.291030 0.251261 0.732242

O -2.778216 1.415073 0.092278

O -4.189783 -0.218797 -0.536935

C -4.135932 1.175076 -0.266523

C -4.450208 1.932613 -1.531307

C -5.056378 1.535488 0.887810

N 2.627192 0.406222 0.461572

C 0.351007 1.800683 0.810573

O 0.336074 2.517014 1.778459

C 3.903564 -0.095523 0.135850

C 4.634997 -0.821147 1.074136

C 5.911623 -1.266761 0.760081

C 6.465942 -0.993165 -0.484508

C 5.735236 -0.263715 -1.415412

C 4.460803 0.191662 -1.108617

O 0.393299 2.232170 -0.444127

C 0.517083 3.652357 -0.646154

C 1.953862 4.092267 -0.486451

O -2.935316 -3.283560 0.091393

C -4.195966 -3.604389 -0.460777

H -0.074346 -3.539743 -0.448176

H -0.110912 -2.208783 -1.616851

H -1.036014 -2.064973 1.290729

H -0.921807 0.053723 -0.898656

H 0.185076 0.025893 1.937800

H -2.542027 -1.868800 -1.351595

H -3.855339 -1.220237 1.254415

H -2.216262 0.405657 1.812842

H -4.348194 3.003460 -1.355218

H -5.473925 1.725751 -1.843383

H -3.762447 1.629237 -2.320325

H -6.089660 1.306447 0.625489

H -4.788174 0.973808 1.784440

H -4.974399 2.600356 1.106836

H 4.197660 -1.027987 2.043713

H 6.474610 -1.830777 1.493501

H 7.461842 -1.341697 -0.726003

H 6.159988 -0.044065 -2.387156

H 3.886637 0.764356 -1.826759

H 0.160924 3.816467 -1.661079

H -0.143964 4.165726 0.050957

H 2.030101 5.162221 -0.685733

H 2.309740 3.902557 0.526246

H 2.596465 3.563245 -1.191356

H -4.430376 -4.626376 -0.167632

H -4.978833 -2.937355 -0.085211

H -4.171894 -3.537854 -1.554029

==============================

**conf019_E0.53**

C 0.017431 -2.456838 0.559080

C 1.085139 -1.753777 -0.240595

C 0.960153 -0.236603 -0.156126

C -0.274103 0.286333 -0.878499

C -1.588870 -0.314824 -0.409128

S -1.672510 -2.034570 0.022303

C 2.507291 -1.995071 0.259810

C 3.314505 -0.854243 -0.377188

C 2.291024 0.251341 -0.732072

O 2.778286 1.414990 -0.091868

O 4.190117 -0.218962 0.536549

C 4.136179 1.174937 0.266098

C 4.451223 1.932505 1.530661

C 5.056029 1.535250 -0.888751

N -2.627271 0.406247 -0.461838

C -0.350984 1.800830 -0.810142

O -0.335762 2.517332 -1.777896

C -3.903641 -0.095549 -0.136151

C -4.635274 -0.820608 -1.074710

C -5.911894 -1.266289 -0.760700

C -6.465987 -0.993342 0.484125

C -5.735083 -0.264432 1.415304

C -4.460662 0.190997 1.108563

O -0.393597 2.232089 0.444629

C -0.517434 3.652243 0.646931

C -1.954150 4.092221 0.486870

O 2.935244 -3.283557 -0.091170

C 4.195994 -3.604289 0.460823

H 0.074254 -3.539633 0.448336

H 0.110991 -2.208669 1.616996

H 1.035924 -2.064934 -1.290644

H 0.921806 0.053716 0.898791

H -0.185065 0.026233 -1.937667

H 2.542018 -1.868602 1.351644

H 3.854974 -1.220339 -1.254680

H 2.216260 0.405941 -1.812641

H 4.349222 3.003347 1.354551

H 5.475091 1.725544 1.842172

H 3.763853 1.629254 2.320070

H 6.089394 1.305800 -0.627125

H 4.787067 0.973830 -1.785318

H 4.974265 2.600184 -1.107540

H -4.198083 -1.026985 -2.044451

H -6.475030 -1.829879 -1.494332

H -7.461876 -1.341923 0.725595

H -6.159676 -0.045273 2.387228

H -3.886322 0.763237 1.826929

H -0.161624 3.816131 1.662015

H 0.143849 4.165763 -0.049842

H -2.596983 3.563082 1.191478

H -2.030399 5.162139 0.686358

H -2.309732 3.902741 -0.525971

H 4.430528 -4.626195 0.167497

H 4.978732 -2.937086 0.085291

H 4.172014 -3.537932 1.554090

==============================

**conf020_E0.58**

C -0.179303 -2.513324 0.246266

C 0.888009 -1.745592 -0.490146

C 0.868032 -0.262898 -0.140790

C -0.362084 0.459042 -0.671523

C -1.695501 -0.161377 -0.280299

S -1.859867 -1.922446 -0.135321

C 2.317767 -2.146212 -0.138597

C 3.156645 -0.968048 -0.666410

C 2.197679 0.241335 -0.723290

O 2.851867 1.256481 0.003180

O 4.221229 -0.543420 0.161591

C 3.877664 0.680984 0.804987

C 3.393417 0.426285 2.223568

C 5.077143 1.600208 0.775504

N -2.698599 0.604607 -0.187720

C -0.336114 1.904950 -0.201379

O -0.304417 2.226544 0.957393

C -3.994097 0.116179 0.075579

C -4.752280 -0.463329 -0.940054

C -6.046343 -0.891774 -0.677596

C -6.591036 -0.747560 0.592672

C -5.833881 -0.163070 1.601392

C -4.542380 0.275945 1.346574

O -0.319968 2.758295 -1.220001

C -0.236847 4.161743 -0.895265

C 1.187871 4.565623 -0.596492

O 2.632198 -3.385791 -0.715856

C 3.908632 -3.854387 -0.330990

H -0.192200 -3.566048 -0.035474

H -0.024523 -2.442216 1.323683

H 0.759067 -1.867190 -1.572356

H 0.892256 -0.158771 0.947436

H -0.331433 0.438582 -1.765150

H 2.432985 -2.202100 0.953675

H 3.562097 -1.229426 -1.646066

H 2.047851 0.599284 -1.744237

H 3.067145 1.364559 2.674194

H 4.209771 0.015361 2.818879

H 2.564180 -0.279104 2.241682

H 4.822041 2.557597 1.230883

H 5.900042 1.155205 1.335111

H 5.387868 1.762826 -0.256217

H -4.322540 -0.570322 -1.928995

H -6.630354 -1.341185 -1.471234

H -7.600289 -1.083032 0.793913

H -6.251130 -0.043624 2.593636

H -3.948409 0.740054 2.124458

H -0.616561 4.667627 -1.780424

H -0.899202 4.365765 -0.055015

H 1.835724 4.337019 -1.443830

H 1.226401 5.640418 -0.412950

H 1.563630 4.047317 0.285012

H 4.702968 -3.187131 -0.680765

H 3.979806 -3.944045 0.758685

H 4.045645 -4.835737 -0.782095

==============================

**conf021_E0.59**

C -0.268089 -2.417940 0.646201

C 0.834770 -1.830414 -0.197538

C 0.829113 -0.306905 -0.165075

C -0.378174 0.297615 -0.869126

C -1.730025 -0.205502 -0.387576

S -1.928336 -1.899131 0.104837

C 2.244580 -2.162590 0.287576

C 3.124792 -1.099900 -0.388492

C 2.176283 0.052137 -0.794151

O 2.754883 1.215934 -0.237610

O 4.028961 -0.481999 0.509849

C 4.084199 0.894590 0.163788

C 4.433140 1.695737 1.392290

C 5.048379 1.122382 -0.988495

N -2.719239 0.578530 -0.476397

C -0.335288 1.814713 -0.801098

O -0.243235 2.526501 -1.767914

C -4.029980 0.174770 -0.152561

C -4.776477 -0.590015 -1.047082

C -6.085682 -0.932814 -0.737944

C -6.657281 -0.518983 0.459143

C -5.911392 0.248798 1.346118

C -4.604708 0.603559 1.042206

O -0.378186 2.249510 0.452689

C -0.317003 3.673414 0.662004

C 1.114005 4.156780 0.677674

O 2.572782 -3.486785 -0.037156

C 3.810318 -3.887635 0.514973

H -0.291629 -3.505938 0.585091

H -0.141656 -2.129834 1.690542

H 0.740712 -2.174108 -1.234249

H 0.848686 0.019002 0.880100

H -0.321533 0.031299 -1.929023

H 2.305907 -2.013615 1.375241

H 3.649627 -1.532798 -1.244626

H 2.086478 0.145549 -1.880506

H 4.421824 2.759582 1.154950

H 5.430618 1.426334 1.739999

H 3.707925 1.493032 2.180185

H 6.057420 0.833394 -0.692824

H 4.753872 0.533540 -1.859000

H 5.049013 2.176815 -1.265704

H -4.326090 -0.906228 -1.980514

H -6.660550 -1.526730 -1.437751

H -7.678407 -0.787950 0.696978

H -6.349695 0.578523 2.279962

H -4.019132 1.208768 1.723553

H -0.902030 4.166972 -0.112841

H -0.799003 3.829080 1.625214

H 1.129082 5.230306 0.872914

H 1.678401 3.652492 1.462481

H 1.599952 3.966871 -0.278791

H 3.969627 -4.928567 0.238782

H 4.637004 -3.285016 0.124418

H 3.797109 -3.800473 1.606929

==============================

**conf022_E0.66**

C -0.142291 -2.403981 0.574270

C 0.913850 -1.792201 -0.310218

C 0.869430 -0.268734 -0.279854

C -0.366730 0.290255 -0.971874

C -1.688873 -0.230347 -0.428698

S -1.833089 -1.920845 0.095752

C 2.348684 -2.092419 0.112410

C 3.171324 -1.034609 -0.645779

C 2.199184 0.130019 -0.937006

O 2.831304 1.271956 -0.403510

O 4.235932 -0.442318 0.071372

C 3.867083 0.869862 0.486578

C 3.387221 0.862509 1.929648

C 5.047808 1.792815 0.291019

N -2.695223 0.535168 -0.478921

C -0.366725 1.809296 -0.951875

O -0.374736 2.492927 -1.943157

C -3.981159 0.113174 -0.086291

C -4.512244 0.568773 1.118649

C -5.793927 0.192560 1.494584

C -6.559299 -0.622538 0.668478

C -6.032581 -1.061788 -0.540089

C -4.748043 -0.698063 -0.920662

O -0.328429 2.284108 0.287642

C -0.323362 3.716445 0.444230

C 1.065084 4.284003 0.265183

O 2.681172 -3.418909 -0.201913

C 3.964768 -3.781415 0.264968

H -0.144538 -3.492244 0.513505

H 0.016671 -2.112228 1.613281

H 0.786476 -2.138749 -1.342673

H 0.877292 0.061873 0.762137

H -0.334326 -0.007050 -2.024512

H 2.463889 -1.924548 1.192925

H 3.574837 -1.482703 -1.556075

H 2.058459 0.290057 -2.008041

H 3.051710 1.861310 2.211594

H 4.209557 0.571059 2.584298

H 2.566610 0.161611 2.075035

H 4.771356 2.811204 0.566216

H 5.877956 1.473441 0.921282

H 5.358074 1.773199 -0.753340

H -3.911869 1.210367 1.752127

H -6.197427 0.542617 2.436664

H -7.561049 -0.908700 0.962144

H -6.623586 -1.692336 -1.192835

H -4.332767 -1.034702 -1.863174

H -1.029596 4.147255 -0.264741

H -0.690277 3.879831 1.455561

H 1.037597 5.360177 0.443836

H 1.758068 3.831501 0.974912

H 1.432702 4.107848 -0.744970

H 4.749301 -3.192255 -0.220714

H 4.040481 -3.640768 1.348944

H 4.113222 -4.833520 0.027528

==============================

**conf023_E0.69**

C -0.213446 -2.499559 -0.250709

C -1.198421 -1.632629 0.490008

C -1.031891 -0.155726 0.150724

C 0.265732 0.431315 0.684842

C 1.528914 -0.318059 0.281057

S 1.516650 -2.087666 0.144534

C -2.661568 -1.887446 0.142624

C -3.375126 -0.639918 0.693287

C -2.303554 0.472421 0.743450

O -2.865227 1.546830 0.024249

O -4.408480 -0.107726 -0.110866

C -3.952349 1.073810 -0.763812

C -3.511601 0.767658 -2.186609

C -5.054905 2.106909 -0.725191

N 2.598583 0.348563 0.165866

C 0.408581 1.875275 0.231335

O 0.329974 2.221487 -0.918072

C 3.838496 -0.259206 -0.114341

C 4.544907 -0.925675 0.885226

C 5.789629 -1.472418 0.604016

C 6.336005 -1.360334 -0.668708

C 5.631392 -0.688398 -1.661065

C 4.390701 -0.130499 -1.387169

O 0.603058 2.700667 1.254434

C 0.822319 4.090301 0.936525

C 2.252268 4.320585 0.506062

O -3.092288 -3.098101 0.706301

C -4.413765 -3.431055 0.332759

H -0.310834 -3.550773 0.020836

H -0.354336 -2.402482 -1.328074

H -1.080037 -1.772333 1.571390

H -1.049411 -0.039042 -0.936317

H 0.237540 0.402775 1.778413

H -2.787890 -1.917227 -0.949270

H -3.786663 -0.870969 1.678073

H -2.112068 0.812450 1.763581

H -3.098076 1.667196 -2.644389

H -4.371505 0.438757 -2.771517

H -2.757360 -0.017470 -2.210854

H -4.714094 3.031425 -1.191949

H -5.925608 1.741003 -1.269543

H -5.333037 2.306062 0.309417

H 4.115240 -1.006236 1.876702

H 6.333505 -1.989149 1.385114

H 7.306271 -1.788845 -0.884595

H 6.050748 -0.593175 -2.655014

H 3.838722 0.402325 -2.151887

H 0.113523 4.390481 0.165911

H 0.593539 4.620414 1.858616

H 2.478314 3.770264 -0.407320

H 2.407220 5.383920 0.317515

H 2.942357 4.004424 1.289264

H -4.640224 -4.403391 0.767122

H -5.133556 -2.696568 0.708032

H -4.508972 -3.490508 -0.757096

==============================

**conf024_E0.69**

C -0.213375 -2.499542 -0.250832

C -1.198350 -1.632668 0.489941

C -1.031845 -0.155751 0.150685

C 0.265816 0.431316 0.684722

C 1.528996 -0.318070 0.280983

S 1.516730 -2.087666 0.144398

C -2.661498 -1.887490 0.142590

C -3.375034 -0.640002 0.693371

C -2.303495 0.472375 0.743456

O -2.865235 1.546742 0.024226

O -4.408525 -0.107796 -0.110595

C -3.952434 1.073667 -0.763702

C -3.511812 0.767412 -2.186517

C -5.054979 2.106772 -0.725074

N 2.598672 0.348568 0.165905

C 0.408575 1.875256 0.231131

O 0.330104 2.221377 -0.918313

C 3.838595 -0.259164 -0.114282

C 4.545019 -0.925545 0.885346

C 5.789767 -1.472261 0.604215

C 6.336182 -1.360232 -0.668499

C 5.631570 -0.688381 -1.660915

C 4.390846 -0.130513 -1.387094

O 0.602754 2.700748 1.254211

C 0.821840 4.090407 0.936253

C 2.251862 4.321005 0.506193

O -3.092194 -3.098189 0.706199

C -4.413758 -3.431025 0.332862

H -0.310733 -3.550776 0.020652

H -0.354287 -2.402407 -1.328190

H -1.079932 -1.772408 1.571317

H -1.049426 -0.039065 -0.936357

H 0.237632 0.402854 1.778296

H -2.787859 -1.917231 -0.949296

H -3.786433 -0.871117 1.678198

H -2.111995 0.812460 1.763563

H -3.098368 1.666929 -2.644415

H -4.371764 0.438436 -2.771315

H -2.757545 -0.017687 -2.210791

H -5.925736 1.740822 -1.269311

H -5.333010 2.306031 0.309540

H -4.714204 3.031240 -1.191956

H 4.115323 -1.006050 1.876815

H 6.333640 -1.988913 1.385368

H 7.306473 -1.788712 -0.884332

H 6.050954 -0.593192 -2.654856

H 3.838896 0.402260 -2.151871

H 0.113173 4.390396 0.165446

H 0.592720 4.620531 1.858251

H 2.406646 5.384393 0.317783

H 2.941787 4.004948 1.289585

H 2.478309 3.770794 -0.407151

H -4.639941 -4.403728 0.766549

H -5.133518 -2.696924 0.708953

H -4.509344 -3.489651 -0.757005

==============================

**conf025_E0.69**

C 0.213337 -2.499583 0.250930

C 1.198367 -1.632788 -0.489872

C 1.031867 -0.155852 -0.150742

C -0.265788 0.431227 -0.684782

C -1.528974 -0.318105 -0.281013

S -1.516725 -2.087742 -0.144518

C 2.661500 -1.887558 -0.142400

C 3.375071 -0.640131 -0.693276

C 2.303502 0.472202 -0.743610

O 2.865208 1.546740 -0.024626

O 4.408432 -0.107823 0.110801

C 3.952302 1.073847 0.763540

C 3.511557 0.767966 2.186399

C 5.054874 2.106915 0.724742

N -2.598620 0.348552 -0.165851

C -0.408480 1.875174 -0.231193

O -0.329888 2.221312 0.918235

C -3.838582 -0.259119 0.114289

C -4.544839 -0.925784 -0.885266

C -5.789626 -1.472440 -0.604177

C -6.336235 -1.360078 0.668420

C -5.631789 -0.687936 1.660760

C -4.391039 -0.130115 1.386975

O -0.602765 2.700660 -1.254262

C -0.821780 4.090318 -0.936283

C -2.251716 4.320904 -0.505933

O 3.092243 -3.098338 -0.705785

C 4.413809 -3.431085 -0.332376

H 0.310709 -3.550848 -0.020427

H 0.354161 -2.402324 1.328288

H 1.080004 -1.772625 -1.571240

H 1.049461 -0.039080 0.936289

H -0.237626 0.402746 -1.778357

H 2.787797 -1.917112 0.949500

H 3.786613 -0.871323 -1.678025

H 2.111985 0.812050 -1.763794

H 3.097961 1.667566 2.643993

H 4.371490 0.439265 2.771382

H 2.757396 -0.017231 2.210829

H 5.925601 1.741045 1.269080

H 5.332949 2.305966 -0.309900

H 4.714120 3.031487 1.191434

H -4.115006 -1.006546 -1.876654

H -6.333365 -1.989314 -1.385277

H -7.306548 -1.788523 0.884227

H -6.051325 -0.592482 2.654611

H -3.839226 0.402890 2.151689

H -0.112963 4.390314 -0.165613

H -0.592831 4.620435 -1.858330

H -2.406467 5.384276 -0.317422

H -2.941796 4.004885 -1.289203

H -2.477971 3.770634 0.407425

H 5.133629 -2.697421 -0.709209

H 4.509564 -3.488807 0.757521

H 4.639756 -4.404192 -0.765281

==============================

**conf026_E0.70**

C 0.213361 -2.499685 0.250731

C 1.198350 -1.632787 -0.490001

C 1.031846 -0.155883 -0.150728

C -0.265797 0.431207 -0.684756

C -1.528980 -0.318150 -0.281004

S -1.516741 -2.087760 -0.144472

C 2.661484 -1.887616 -0.142583

C 3.375079 -0.640101 -0.693231

C 2.303498 0.472215 -0.743527

O 2.865173 1.546722 -0.024488

O 4.408331 -0.107897 0.111073

C 3.952218 1.073815 0.763737

C 3.511383 0.767929 2.186566

C 5.054815 2.106864 0.724979

N -2.598617 0.348540 -0.165838

C -0.408496 1.875164 -0.231192

O -0.330091 2.221301 0.918253

C -3.838595 -0.259106 0.114314

C -4.390866 -0.130364 1.387114

C -5.631629 -0.688132 1.660932

C -6.336279 -1.359948 0.668514

C -5.789857 -1.472053 -0.604186

C -4.545054 -0.925449 -0.885308

O -0.602516 2.700653 -1.254298

C -0.821385 4.090352 -0.936385

C -2.251349 4.321131 -0.506253

O 3.092216 -3.098291 -0.706201

C 4.413664 -3.431257 -0.332573

H 0.310701 -3.550901 -0.020823

H 0.354284 -2.402617 1.328092

H 1.079972 -1.772517 -1.571382

H 1.049434 -0.039214 0.936315

H -0.237631 0.402723 -1.778332

H 2.787783 -1.917352 0.949315

H 3.786748 -0.871137 -1.677964

H 2.111986 0.812095 -1.763702

H 4.371293 0.439311 2.771629

H 2.757306 -0.017354 2.210901

H 3.097652 1.667488 2.644118

H 4.714053 3.031451 1.191638

H 5.925512 1.740992 1.269366

H 5.332945 2.305896 -0.309653

H -3.838875 0.402390 2.151875

H -6.051038 -0.592884 2.654857

H -7.306608 -1.788347 0.884340

H -6.333771 -1.988666 -1.385336

H -4.115349 -1.006016 -1.876767

H -0.112649 4.390257 -0.165608

H -0.592221 4.620423 -1.858405

H -2.405955 5.384522 -0.317732

H -2.941361 4.005244 -1.289634

H -2.477830 3.770880 0.407061

H 5.133527 -2.696971 -0.708109

H 4.508886 -3.490353 0.757300

H 4.640021 -4.403761 -0.766614

==============================

**conf027_E0.70**

C -0.213439 -2.499523 -0.250552

C -1.198423 -1.632543 0.490097

C -1.031863 -0.155667 0.150703

C 0.265781 0.431374 0.684789

C 1.528939 -0.318061 0.281059

S 1.516647 -2.087677 0.144784

C -2.661563 -1.887366 0.142724

C -3.375081 -0.639812 0.693374

C -2.303527 0.472568 0.743345

O -2.865239 1.546838 0.023946

O -4.408565 -0.107690 -0.110655

C -3.952432 1.073661 -0.763934

C -3.511773 0.767169 -2.186683

C -5.054953 2.106790 -0.725490

N 2.598614 0.348551 0.165803

C 0.408635 1.875318 0.231273

O 0.330074 2.221538 -0.918133

C 3.838498 -0.259236 -0.114439

C 4.545160 -0.925251 0.885264

C 5.789867 -1.472019 0.604054

C 6.336002 -1.360407 -0.668818

C 5.631158 -0.688913 -1.661310

C 4.390475 -0.130997 -1.387414

O 0.603059 2.700708 1.254393

C 0.822362 4.090342 0.936504

C 2.252353 4.320621 0.506186

O -3.092297 -3.097995 0.706453

C -4.413833 -3.430865 0.333048

H -0.310853 -3.550733 0.021000

H -0.354270 -2.402457 -1.327927

H -1.080046 -1.772182 1.571490

H -1.049376 -0.039070 -0.936351

H 0.237573 0.402866 1.778364

H -2.787899 -1.917208 -0.949165

H -3.786488 -0.870815 1.678223

H -2.112065 0.812792 1.763414

H -2.757509 -0.017940 -2.210811

H -3.098315 1.666607 -2.644719

H -4.371707 0.438097 -2.771456

H -5.925703 1.740790 -1.269705

H -5.333015 2.306209 0.309085

H -4.714152 3.031186 -1.192497

H 4.115671 -1.005440 1.876848

H 6.333933 -1.988381 1.385264

H 7.306258 -1.788934 -0.884711

H 6.050335 -0.594043 -2.655369

H 3.838337 0.401489 -2.152255

H 0.113633 4.390528 0.165831

H 0.593500 4.620456 1.858574

H 2.942361 4.004415 1.289443

H 2.478492 3.770343 -0.407197

H 2.407345 5.383965 0.317719

H -4.640165 -4.403372 0.767096

H -5.133584 -2.696550 0.708741

H -4.509257 -3.489919 -0.756809

==============================

**conf028_E0.72**

C -0.213354 -2.499651 -0.250605

C -1.198332 -1.632718 0.490113

C -1.031819 -0.155828 0.150762

C 0.265810 0.431282 0.684784

C 1.528994 -0.318089 0.281060

S 1.516759 -2.087680 0.144489

C -2.661471 -1.887538 0.142691

C -3.375043 -0.640014 0.693330

C -2.303477 0.472335 0.743474

O -2.865165 1.546727 0.024261

O -4.408395 -0.107846 -0.110847

C -3.952231 1.073655 -0.763844

C -3.511411 0.767446 -2.186609

C -5.054795 2.106748 -0.725343

N 2.598649 0.348572 0.165937

C 0.408528 1.875216 0.231177

O 0.330091 2.221300 -0.918284

C 3.838577 -0.259123 -0.114299

C 4.390605 -0.130784 -1.387246

C 5.631333 -0.688603 -1.661119

C 6.336172 -1.360120 -0.668631

C 5.789980 -1.471846 0.604201

C 4.545228 -0.925160 0.885393

O 0.602579 2.700732 1.254251

C 0.821482 4.090417 0.936298

C 2.251477 4.321180 0.506250

O -3.092221 -3.098210 0.706294

C -4.413763 -3.431041 0.332873

H -0.310661 -3.550853 0.021013

H -0.354324 -2.402647 -1.327967

H -1.079945 -1.772405 1.571496

H -1.049378 -0.039229 -0.936291

H 0.237620 0.402822 1.778360

H -2.787746 -1.917290 -0.949207

H -3.786597 -0.871030 1.678114

H -2.111993 0.812382 1.763599

H -4.371316 0.438634 -2.771569

H -2.757258 -0.017766 -2.210827

H -3.097772 1.666940 -2.644376

H -5.925490 1.740771 -1.269659

H -5.332934 2.306018 0.309241

H -4.713994 3.031215 -1.192206

H 3.838474 0.401722 -2.152079

H 6.050543 -0.593657 -2.655157

H 7.306469 -1.788569 -0.884503

H 6.334031 -1.988229 1.385408

H 4.115719 -1.005419 1.876962

H 0.112792 4.390305 0.165471

H 0.592270 4.620515 1.858290

H 2.406086 5.384562 0.317679

H 2.941437 4.005347 1.289698

H 2.478025 3.770870 -0.407011

H -4.509339 -3.489430 -0.757006

H -4.639891 -4.403853 0.766344

H -5.133566 -2.697064 0.709128

==============================

**conf029_E0.75**

C -0.213402 -2.499537 -0.250741

C -1.198383 -1.632630 0.489994

C -1.031895 -0.155718 0.150717

C 0.265739 0.431358 0.684789

C 1.528912 -0.318005 0.280974

S 1.516687 -2.087593 0.144448

C -2.661522 -1.887472 0.142600

C -3.375109 -0.639976 0.693299

C -2.303561 0.472382 0.743486

O -2.865276 1.546811 0.024346

O -4.408477 -0.107796 -0.110858

C -3.952371 1.073788 -0.763751

C -3.511602 0.767721 -2.186559

C -5.054961 2.106847 -0.725100

N 2.598581 0.348633 0.165813

C 0.408533 1.875319 0.231301

O 0.329865 2.221536 -0.918101

C 3.838484 -0.259171 -0.114366

C 4.390807 -0.130428 -1.387135

C 5.631491 -0.688381 -1.660951

C 6.335985 -1.360410 -0.668574

C 5.789495 -1.472531 0.604098

C 4.544781 -0.925731 0.885226

O 0.603010 2.700709 1.254403

C 0.822351 4.090334 0.936483

C 2.252312 4.320547 0.506027

O -3.092221 -3.098165 0.706215

C -4.413725 -3.431085 0.332743

H -0.310747 -3.550750 0.020826

H -0.354333 -2.402486 -1.328104

H -1.079991 -1.772345 1.571375

H -1.049455 -0.039038 -0.936326

H 0.237580 0.402801 1.778362

H -2.787842 -1.917215 -0.949293

H -3.786655 -0.871042 1.678076

H -2.112064 0.812374 1.763628

H -4.371504 0.438871 -2.771502

H -2.757381 -0.017423 -2.210853

H -3.098061 1.667279 -2.644283

H -5.333076 2.306003 0.309511

H -4.714202 3.031374 -1.191875

H -5.925665 1.740910 -1.269429

H 3.838935 0.402467 -2.151881

H 6.050936 -0.593127 -2.654860

H 7.306241 -1.788967 -0.884409

H 6.333273 -1.989329 1.385219

H 4.115027 -1.006309 1.876662

H 0.113554 4.390553 0.165882

H 0.593606 4.620457 1.858577

H 2.478360 3.770203 -0.407338

H 2.407313 5.383874 0.317473

H 2.942372 4.004382 1.289253

H -4.639939 -4.403733 0.766537

H -5.133552 -2.696951 0.708644

H -4.509162 -3.489858 -0.757127

==============================

**conf030_E0.76**

C 0.213411 -2.499530 0.250851

C 1.198357 -1.632663 -0.489977

C 1.031881 -0.155748 -0.150740

C -0.265758 0.431350 -0.684768

C -1.528935 -0.318021 -0.281016

S -1.516719 -2.087579 -0.144237

C 2.661509 -1.887491 -0.142614

C 3.375078 -0.640016 -0.693383

C 2.303527 0.472348 -0.743548

O 2.865264 1.546773 -0.024418

O 4.408485 -0.107804 0.110698

C 3.952383 1.073748 0.763635

C 3.511656 0.767635 2.186454

C 5.054960 2.106819 0.724992

N -2.598628 0.348615 -0.166016

C -0.408560 1.875282 -0.231158

O -0.330034 2.221392 0.918287

C -3.838523 -0.259163 0.114195

C -4.390670 -0.130687 1.387079

C -5.631359 -0.688599 1.660933

C -6.336054 -1.360330 0.668483

C -5.789730 -1.472211 -0.604278

C -4.545003 -0.925452 -0.885445

O -0.602870 2.700772 -1.254205

C -0.822111 4.090390 -0.936191

C -2.252098 4.320744 -0.505888

O 3.092196 -3.098206 -0.706173

C 4.413726 -3.431081 -0.332750

H 0.310701 -3.550760 -0.020664

H 0.354405 -2.402415 1.328199

H 1.079924 -1.772441 -1.571345

H 1.049475 -0.039053 0.936302

H -0.237590 0.402907 -1.778341

H 2.787852 -1.917177 0.949279

H 3.786574 -0.871118 -1.678170

H 2.112008 0.812340 -1.763683

H 2.757457 -0.017531 2.210752

H 3.098103 1.667174 2.644208

H 4.371585 0.438794 2.771365

H 4.714198 3.031331 1.191799

H 5.925680 1.740874 1.269292

H 5.333049 2.306006 -0.309622

H -3.838646 0.401992 2.151870

H -6.050664 -0.593551 2.654920

H -7.306323 -1.788845 0.884343

H -6.333652 -1.988783 -1.385451

H -4.115372 -1.005867 -1.876951

H -0.113366 4.390471 -0.165493

H -0.593221 4.620570 -1.858214

H -2.942101 4.004649 -1.289196

H -2.478320 3.770432 0.407451

H -2.406995 5.384096 -0.317370

H 5.133535 -2.697028 -0.708844

H 4.509253 -3.489656 0.757124

H 4.639893 -4.403813 -0.766381

==============================

**conf031_E0.76**

C 0.213399 -2.499562 0.250812

C 1.198348 -1.632675 -0.489989

C 1.031867 -0.155769 -0.150726

C -0.265772 0.431324 -0.684762

C -1.528955 -0.318041 -0.280998

S -1.516723 -2.087621 -0.144297

C 2.661499 -1.887513 -0.142636

C 3.375072 -0.640019 -0.693355

C 2.303518 0.472339 -0.743515

O 2.865242 1.546761 -0.024366

O 4.408456 -0.107830 0.110774

C 3.952351 1.073730 0.763702

C 3.511611 0.767617 2.186513

C 5.054932 2.106797 0.725064

N -2.598641 0.348600 -0.165972

C -0.408531 1.875266 -0.231182

O -0.330025 2.221400 0.918257

C -3.838558 -0.259139 0.114240

C -4.545034 -0.925440 -0.885396

C -5.789771 -1.472181 -0.604236

C -6.336112 -1.360254 0.668513

C -5.631440 -0.688477 1.660945

C -4.390735 -0.130596 1.387102

O -0.602764 2.700745 -1.254255

C -0.821907 4.090387 -0.936283

C -2.251885 4.320852 -0.506017

O 3.092189 -3.098201 -0.706264

C 4.413678 -3.431144 -0.332759

H 0.310712 -3.550783 -0.020731

H 0.354380 -2.402473 1.328165

H 1.079917 -1.772427 -1.571361

H 1.049452 -0.039086 0.936317

H -0.237605 0.402847 -1.778336

H 2.787851 -1.917251 0.949254

H 3.786599 -0.871089 -1.678137

H 2.112007 0.812346 -1.763646

H 3.098043 1.667149 2.644265

H 4.371536 0.438787 2.771435

H 2.757424 -0.017561 2.210802

H 4.714177 3.031311 1.191872

H 5.925648 1.740852 1.269372

H 5.333031 2.305984 -0.309546

H -4.115400 -1.005869 -1.876897

H -6.333680 -1.988773 -1.385403

H -7.306386 -1.788756 0.884378

H -6.050772 -0.593373 2.654917

H -3.838734 0.402114 2.151886

H -0.113160 4.390433 -0.165570

H -0.592943 4.620526 -1.858313

H -2.406729 5.384215 -0.317524

H -2.941897 4.004775 -1.289323

H -2.478151 3.770578 0.407336

H 4.639922 -4.403754 -0.766623

H 5.133518 -2.696974 -0.708565

H 4.509063 -3.490008 0.757112

==============================

**conf032_E0.76**

C 0.143847 -2.407735 0.550223

C 1.162710 -1.716649 -0.320094

C 1.006361 -0.200912 -0.290262

C -0.261382 0.269463 -0.989551

C -1.544890 -0.341049 -0.449957

S -1.574565 -2.039656 0.064437

C 2.610437 -1.907249 0.123324

C 3.362168 -0.791349 -0.625879

C 2.308131 0.293259 -0.938416

O 2.846804 1.485739 -0.412981

O 4.364775 -0.116580 0.107797

C 3.898958 1.173318 0.494357

C 3.402572 1.158625 1.931667

C 5.014479 2.173338 0.293827

N -2.601270 0.353746 -0.497736

C -0.369627 1.783628 -0.963404

O -0.417080 2.471475 -1.950384

C -3.852474 -0.159169 -0.099708

C -4.611149 -0.926102 -0.981585

C -5.864521 -1.382779 -0.597016

C -6.368132 -1.079312 0.662029

C -5.610199 -0.309076 1.536613

C -4.358816 0.157092 1.159353

O -0.363163 2.251129 0.279854

C -0.516299 3.673048 0.447622

C -1.970787 4.069859 0.346201

O 3.045816 -3.205492 -0.182489

C 4.346338 -3.470324 0.302675

H 0.218674 -3.492877 0.481419

H 0.273804 -2.114341 1.592759

H 1.075931 -2.070560 -1.354229

H 0.981147 0.128051 0.751884

H -0.203780 -0.023048 -2.042391

H 2.697082 -1.728439 1.204941

H 3.814570 -1.209499 -1.527439

H 2.165206 0.430701 -2.012330

H 2.976250 2.130765 2.182851

H 4.237828 0.956855 2.603485

H 2.645016 0.391551 2.085024

H 5.859623 1.920050 0.934078

H 5.333647 2.160663 -0.747966

H 4.667323 3.173943 0.552983

H -4.212938 -1.156181 -1.962683

H -6.449151 -1.979126 -1.286597

H -7.345981 -1.436477 0.958441

H -5.995345 -0.066165 2.519255

H -3.763459 0.761199 1.833149

H -0.116445 3.876283 1.438997

H 0.095736 4.182992 -0.295195

H -2.369575 3.840824 -0.642106

H -2.563499 3.545164 1.096596

H -2.067132 5.142846 0.518678

H 4.396423 -3.323413 1.387280

H 4.576366 -4.508642 0.069355

H 5.091020 -2.824561 -0.173584

==============================

**conf033_E0.76**

C 0.143879 -2.407739 0.550223

C 1.162728 -1.716646 -0.320105

C 1.006368 -0.200910 -0.290264

C -0.261378 0.269460 -0.989549

C -1.544883 -0.341061 -0.449958

S -1.574545 -2.039651 0.064477

C 2.610459 -1.907237 0.123307

C 3.362181 -0.791327 -0.625888

C 2.308134 0.293277 -0.938413

O 2.846801 1.485754 -0.412970

O 4.364783 -0.116557 0.107790

C 3.898950 1.173328 0.494370

C 3.402554 1.158605 1.931677

C 5.014460 2.173364 0.293868

N -2.601274 0.353715 -0.497772

C -0.369632 1.783625 -0.963390

O -0.417036 2.471486 -1.950360

C -3.852474 -0.159195 -0.099730

C -4.358775 0.157020 1.159362

C -5.610158 -0.309134 1.536637

C -6.368133 -1.079317 0.662040

C -5.864564 -1.382740 -0.597032

C -4.611192 -0.926072 -0.981617

O -0.363226 2.251109 0.279876

C -0.516366 3.673025 0.447649

C -1.970848 4.069837 0.346144

O 3.045844 -3.205476 -0.182505

C 4.346386 -3.470285 0.302615

H 0.218692 -3.492881 0.481402

H 0.273857 -2.114360 1.592760

H 1.075941 -2.070557 -1.354238

H 0.981147 0.128043 0.751885

H -0.203777 -0.023034 -2.042394

H 2.697099 -1.728427 1.204926

H 3.814581 -1.209465 -1.527454

H 2.165206 0.430726 -2.012325

H 4.237808 0.956830 2.603495

H 2.645001 0.391524 2.085015

H 2.976224 2.130740 2.182872

H 5.333638 2.160707 -0.747923

H 4.667284 3.173959 0.553034

H 5.859599 1.920075 0.934123

H -3.763384 0.761087 1.833165

H -5.995274 -0.066254 2.519299

H -7.345984 -1.436472 0.958463

H -6.449227 -1.979044 -1.286622

H -4.213018 -1.156115 -1.962739

H -0.116573 3.876251 1.439050

H 0.095712 4.182975 -0.295126

H -2.067194 5.142825 0.518614

H -2.369582 3.840803 -0.642186

H -2.563604 3.545145 1.096507

H 4.576430 -4.508597 0.069283

H 5.091040 -2.824503 -0.173662

H 4.396503 -3.323378 1.387220

==============================

**conf034_E0.76**

C 0.143865 -2.407722 0.550247

C 1.162718 -1.716641 -0.320087

C 1.006369 -0.200904 -0.290254

C -0.261380 0.269471 -0.989530

C -1.544886 -0.341049 -0.449938

S -1.574554 -2.039641 0.064488

C 2.610450 -1.907238 0.123318

C 3.362172 -0.791341 -0.625896

C 2.308132 0.293269 -0.938418

O 2.846810 1.485746 -0.412981

O 4.364792 -0.116574 0.107764

C 3.898972 1.173316 0.494344

C 3.402597 1.158604 1.931657

C 5.014488 2.173342 0.293819

N -2.601274 0.353733 -0.497753

C -0.369632 1.783635 -0.963369

O -0.417029 2.471497 -1.950340

C -3.852477 -0.159187 -0.099727

C -4.358822 0.157062 1.159336

C -5.610207 -0.309105 1.536588

C -6.368139 -1.079333 0.661996

C -5.864526 -1.382791 -0.597050

C -4.611152 -0.926110 -0.981612

O -0.363228 2.251117 0.279897

C -0.516365 3.673035 0.447669

C -1.970844 4.069853 0.346155

O 3.045826 -3.205484 -0.182490

C 4.346363 -3.470303 0.302638

H 0.218684 -3.492865 0.481443

H 0.273840 -2.114328 1.592780

H 1.075927 -2.070555 -1.354219

H 0.981165 0.128057 0.751893

H -0.203784 -0.023025 -2.042375

H 2.697101 -1.728423 1.204934

H 3.814559 -1.209493 -1.527463

H 2.165200 0.430717 -2.012330

H 2.976290 2.130747 2.182860

H 4.237854 0.956811 2.603465

H 2.645029 0.391539 2.085005

H 4.667328 3.173942 0.552987

H 5.859635 1.920051 0.934064

H 5.333652 2.160678 -0.747975

H -3.763466 0.761163 1.833138

H -5.995358 -0.066198 2.519230

H -7.345991 -1.436498 0.958402

H -6.449155 -1.979130 -1.286637

H -4.212941 -1.156180 -1.962713

H -0.116576 3.876258 1.439073

H 0.095721 4.182981 -0.295103

H -2.563607 3.545159 1.096511

H -2.067190 5.142839 0.518633

H -2.369571 3.840827 -0.642178

H 5.091026 -2.824536 -0.173645

H 4.396478 -3.323384 1.387241

H 4.576394 -4.508620 0.069318

==============================

**conf035_E0.83**

C -0.142223 -2.403811 0.574375

C 0.913846 -1.792061 -0.310218

C 0.869447 -0.268589 -0.279885

C -0.366711 0.290367 -0.971924

C -1.688856 -0.230270 -0.428780

S -1.833055 -1.920631 0.096047

C 2.348704 -2.092323 0.112302

C 3.171302 -1.034533 -0.645962

C 2.199220 0.130204 -0.936979

O 2.831380 1.272006 -0.403210

O 4.236113 -0.442357 0.070949

C 3.867308 0.869673 0.486606

C 3.387671 0.861968 1.929753

C 5.047993 1.792702 0.291124

N -2.695280 0.535133 -0.479239

C -0.366799 1.809422 -0.951883

O -0.374664 2.493082 -1.943141

C -3.981148 0.113041 -0.086476

C -4.748268 -0.697761 -0.921050

C -6.032726 -1.061620 -0.540322

C -6.559120 -0.622933 0.668589

C -5.793508 0.191736 1.494898

C -4.511898 0.568064 1.118828

O -0.328752 2.284191 0.287659

C -0.323996 3.716520 0.444319

C 1.064367 4.284378 0.265552

O 2.681127 -3.418817 -0.202097

C 3.964767 -3.781372 0.264620

H -0.144487 -3.492074 0.513615

H 0.016852 -2.112057 1.613370

H 0.786383 -2.138639 -1.342652

H 0.877304 0.062039 0.762101

H -0.334274 -0.006895 -2.024571

H 2.463988 -1.924517 1.192815

H 3.574582 -1.482663 -1.556345

H 2.058544 0.290486 -2.007983

H 3.052510 1.860783 2.212067

H 4.210029 0.570064 2.584171

H 2.566851 0.161300 2.075059

H 5.878181 1.473284 0.921309

H 5.358209 1.773241 -0.753252

H 4.771475 2.811032 0.566467

H -4.333229 -1.033969 -1.863820

H -6.623920 -1.691831 -1.193222

H -7.560804 -0.909203 0.962375

H -6.196759 0.541351 2.437248

H -3.911333 1.209328 1.752462

H -1.030173 4.147223 -0.264775

H -0.691132 3.879795 1.455587

H 1.757350 3.831869 0.975277

H 1.432125 4.108504 -0.744601

H 1.036661 5.360510 0.444414

H 4.113146 -4.833483 0.027162

H 4.749263 -3.192239 -0.221153

H 4.040624 -3.640733 1.348589

==============================

**conf036_E0.84**

C -0.142139 -2.403853 0.574490

C 0.913917 -1.792108 -0.310124

C 0.869458 -0.268641 -0.279851

C -0.366732 0.290253 -0.971887

C -1.688856 -0.230407 -0.428709

S -1.832980 -1.920799 0.096067

C 2.348786 -2.092292 0.112408

C 3.171342 -1.034507 -0.645905

C 2.199200 0.130163 -0.936995

O 2.831323 1.272038 -0.403343

O 4.236109 -0.442237 0.071008

C 3.867234 0.869817 0.486547

C 3.387550 0.862184 1.929675

C 5.047883 1.792879 0.291038

N -2.695266 0.535022 -0.479088

C -0.366838 1.809300 -0.951901

O -0.374827 2.492923 -1.943186

C -3.981175 0.113005 -0.086413

C -4.512383 0.568911 1.118360

C -5.794048 0.192674 1.494334

C -6.559283 -0.622756 0.668427

C -6.032452 -1.062297 -0.539984

C -4.747931 -0.698547 -0.920596

O -0.328643 2.284112 0.287623

C -0.323852 3.716443 0.444237

C 1.064509 4.284268 0.265363

O 2.681261 -3.418789 -0.201927

C 3.964911 -3.781272 0.264820

H -0.144347 -3.492120 0.513802

H 0.016900 -2.112018 1.613468

H 0.786469 -2.138730 -1.342545

H 0.877323 0.062030 0.762121

H -0.334306 -0.007049 -2.024524

H 2.464071 -1.924422 1.192912

H 3.574673 -1.482651 -1.556258

H 2.058493 0.290346 -2.008010

H 4.209928 0.570463 2.584150

H 2.566834 0.161403 2.075027

H 3.052235 1.860975 2.211887

H 4.771335 2.811211 0.566344

H 5.878080 1.473520 0.921243

H 5.358113 1.773393 -0.753333

H -3.912125 1.210786 1.751664

H -6.197643 0.542972 2.436284

H -7.561013 -0.908949 0.962131

H -6.623352 -1.693096 -1.192582

H -4.332565 -1.035424 -1.862983

H -1.030071 4.147141 -0.264820

H -0.690921 3.879751 1.455525

H 1.036837 5.360414 0.444163

H 1.757528 3.831796 0.975076

H 1.432209 4.108330 -0.744799

H 4.113328 -4.833393 0.027426

H 4.749388 -3.192145 -0.220989

H 4.040756 -3.640563 1.348780

==============================

**conf037_E0.87**

C -0.268049 -2.418017 0.646087

C 0.834777 -1.830405 -0.197639

C 0.829113 -0.306902 -0.165069

C -0.378145 0.297637 -0.869153

C -1.730004 -0.205499 -0.387634

S -1.928327 -1.899058 0.104922

C 2.244598 -2.162651 0.287408

C 3.124824 -1.099867 -0.388500

C 2.176309 0.052210 -0.794056

O 2.754888 1.215944 -0.237337

O 4.028937 -0.482052 0.509957

C 4.084177 0.894581 0.164034

C 4.433136 1.695584 1.392625

C 5.048402 1.122494 -0.988198

N -2.719241 0.578505 -0.476554

C -0.335278 1.814741 -0.801100

O -0.243185 2.526550 -1.767892

C -4.029964 0.174736 -0.152627

C -4.604413 0.603118 1.042428

C -5.911064 0.248358 1.346476

C -6.657191 -0.519041 0.459364

C -6.085867 -0.932474 -0.737990

C -4.776700 -0.589655 -1.047278

O -0.378226 2.249544 0.452681

C -0.317116 3.673457 0.661927

C 1.113831 4.157035 0.677178

O 2.572759 -3.486790 -0.037562

C 3.810213 -3.887817 0.514623

H -0.291648 -3.506002 0.584794

H -0.141546 -2.130068 1.690461

H 0.740696 -2.174035 -1.234369

H 0.848609 0.018941 0.880126

H -0.321471 0.031350 -1.929051

H 2.305944 -2.013850 1.375096

H 3.649702 -1.532649 -1.244668

H 2.086556 0.145777 -1.880400

H 4.421969 2.759449 1.155367

H 5.430553 1.426013 1.740371

H 3.707843 1.492924 2.180463

H 6.057440 0.833545 -0.692479

H 4.753978 0.533689 -1.858755

H 5.048983 2.176945 -1.265342

H -4.018634 1.208018 1.723876

H -6.349156 0.577767 2.280530

H -7.678289 -0.788019 0.697304

H -6.660930 -1.526086 -1.437894

H -4.326528 -0.905555 -1.980919

H -0.902434 4.166917 -0.112767

H -0.798853 3.829102 1.625270

H 1.599538 3.967229 -0.279427

H 1.128775 5.230564 0.872422

H 1.678538 3.652833 1.461814

H 4.636897 -3.284761 0.124741

H 3.796677 -3.801450 1.606640

H 3.969788 -4.928515 0.237714

==============================

**conf038_E0.87**

C -0.268049 -2.418007 0.646178

C 0.834762 -1.830366 -0.197547

C 0.829058 -0.306860 -0.164943

C -0.378196 0.297666 -0.869030

C -1.730046 -0.205496 -0.387507

S -1.928346 -1.899046 0.105050

C 2.244593 -2.162563 0.287497

C 3.124770 -1.099869 -0.388607

C 2.176255 0.052325 -0.793878

O 2.754890 1.215902 -0.236874

O 4.029259 -0.482207 0.509563

C 4.084349 0.894497 0.163825

C 4.433875 1.695309 1.392379

C 5.048072 1.122554 -0.988802

N -2.719287 0.578509 -0.476430

C -0.335368 1.814773 -0.800972

O -0.243307 2.526584 -1.767766

C -4.030023 0.174708 -0.152625

C -4.776459 -0.590199 -1.047090

C -6.085663 -0.933006 -0.737956

C -6.657327 -0.519066 0.459062

C -5.911495 0.248829 1.345992

C -4.604813 0.603596 1.042085

O -0.378285 2.249563 0.452814

C -0.317103 3.673470 0.662098

C 1.113865 4.156991 0.677204

O 2.572744 -3.486747 -0.037286

C 3.810328 -3.887623 0.514718

H -0.291650 -3.505990 0.584856

H -0.141534 -2.130086 1.690559

H 0.740685 -2.173988 -1.234279

H 0.848527 0.018947 0.880263

H -0.321524 0.031385 -1.928928

H 2.305970 -2.013600 1.375164

H 3.649308 -1.532707 -1.244957

H 2.086507 0.146149 -1.880201

H 3.708896 1.492596 2.180491

H 4.422687 2.759206 1.155269

H 5.431417 1.425614 1.739671

H 6.057236 0.833568 -0.693554

H 4.753276 0.533853 -1.859304

H 5.048532 2.177040 -1.265815

H -4.326020 -0.906519 -1.980462

H -6.660487 -1.527023 -1.437713

H -7.678453 -0.788045 0.696878

H -6.349847 0.578636 2.279786

H -4.019271 1.208904 1.723374

H -0.902487 4.166978 -0.112512

H -0.798723 3.829099 1.625503

H 1.678668 3.652664 1.461689

H 1.599431 3.967284 -0.279491

H 1.128869 5.230498 0.872572

H 3.969753 -4.928450 0.238207

H 4.636942 -3.284787 0.124348

H 3.797110 -3.800795 1.606703

==============================

**conf039_E0.96**

C -0.330134 -2.541078 0.297166

C 0.783561 -1.790923 -0.387497

C 0.797693 -0.315657 -0.004348

C -0.391439 0.451904 -0.565665

C -1.755917 -0.131727 -0.233147

S -1.979145 -1.888026 -0.117451

C 2.186734 -2.246963 0.002836

C 3.080289 -1.082645 -0.447349

C 2.160367 0.161249 -0.513566

O 2.771751 1.104853 0.344201

O 4.059895 -0.729354 0.511859

C 4.118350 0.690656 0.547081

C 4.565296 1.130109 1.917770

C 5.006287 1.219325 -0.567329

N -2.740188 0.661247 -0.173000

C -0.332893 1.891964 -0.082176

O -0.352758 2.206520 1.078412

C -4.057030 0.203828 0.035592

C -4.808285 -0.293172 -1.027805

C -6.121131 -0.693141 -0.818555

C -6.691781 -0.600994 0.445110

C -5.941047 -0.098394 1.501732

C -4.630182 0.310307 1.301123

O -0.218676 2.748234 -1.092756

C -0.078738 4.143727 -0.754285

C 1.349727 4.469159 -0.385488

O 2.483005 -3.479841 -0.596070

C 3.725075 -4.002832 -0.170586

H -0.367163 -3.585619 -0.011569

H -0.205821 -2.501407 1.379979

H 0.686531 -1.884782 -1.475544

H 0.798399 -0.235467 1.087904

H -0.321268 0.441623 -1.657469

H 2.258557 -2.330039 1.097150

H 3.537702 -1.314070 -1.413524

H 2.082867 0.554868 -1.531696

H 3.892374 0.721040 2.670951

H 4.555937 2.218502 1.977979

H 5.579277 0.778172 2.107845

H 4.643565 0.883195 -1.540400

H 5.007045 2.309676 -0.553170

H 6.027512 0.862407 -0.431010

H -4.357754 -0.359974 -2.010973

H -6.699629 -1.078984 -1.648886

H -7.715678 -0.913770 0.604560

H -6.378274 -0.020266 2.489469

H -4.040094 0.709176 2.117264

H -0.388393 4.673389 -1.652777

H -0.768704 4.378409 0.055081

H 1.656153 3.922770 0.505898

H 2.023430 4.213932 -1.204788

H 1.436487 5.538604 -0.187733

H 3.740401 -4.137592 0.916546

H 3.855672 -4.969129 -0.654751

H 4.553948 -3.346477 -0.454980

==============================

**conf040_E0.97**

C -0.330323 -2.541073 0.297037

C 0.783453 -1.790976 -0.387558

C 0.797642 -0.315713 -0.004428

C -0.391446 0.451923 -0.565730

C -1.755956 -0.131631 -0.233206

S -1.979278 -1.887955 -0.117727

C 2.186585 -2.247057 0.002885

C 3.080216 -1.082780 -0.447265

C 2.160316 0.161108 -0.513700

O 2.771703 1.104878 0.343889

O 4.059692 -0.729400 0.512046

C 4.118241 0.690625 0.547006

C 4.565061 1.130307 1.917665

C 5.006340 1.219024 -0.567399

N -2.740195 0.661362 -0.173033

C -0.332788 1.891955 -0.082186

O -0.352758 2.206461 1.078415

C -4.057043 0.203993 0.035594

C -4.629982 0.310020 1.301259

C -5.940859 -0.098619 1.501900

C -6.691805 -0.600733 0.445195

C -6.121357 -0.692450 -0.818591

C -4.808506 -0.292517 -1.027886

O -0.218309 2.748255 -1.092706

C -0.078102 4.143693 -0.754130

C 1.350401 4.468766 -0.385152

O 2.482875 -3.479958 -0.595953

C 3.724863 -4.002988 -0.170283

H -0.367368 -3.585622 -0.011672

H -0.206104 -2.501376 1.379859

H 0.686481 -1.884842 -1.475611

H 0.798375 -0.235510 1.087824

H -0.321275 0.441651 -1.657536

H 2.258321 -2.330101 1.097206

H 3.537757 -1.314278 -1.413360

H 2.082824 0.554545 -1.531900

H 3.892028 0.721414 2.670845

H 4.555747 2.218714 1.977674

H 5.579001 0.778351 2.107918

H 5.007161 2.309379 -0.553446

H 6.027524 0.862065 -0.430881

H 4.643713 0.882738 -1.540453

H -4.039723 0.708487 2.117474

H -6.377932 -0.020838 2.489730

H -7.715716 -0.913446 0.604679

H -6.700029 -1.077893 -1.648988

H -4.358130 -0.358958 -2.011150

H -0.387546 4.673487 -1.652616

H -0.768097 4.378479 0.055179

H 1.656614 3.922197 0.506199

H 2.024130 4.213465 -1.204410

H 1.437395 5.538168 -0.187268

H 3.855302 -4.969493 -0.654074

H 4.553837 -3.346870 -0.454943

H 3.740184 -4.137330 0.916899

==============================

**conf041_E0.98**

C 0.017379 -2.456849 0.558956

C 1.085095 -1.753739 -0.240671

C 0.960123 -0.236570 -0.156109

C -0.274097 0.286404 -0.878516

C -1.588872 -0.314731 -0.409171

S -1.672562 -2.034500 0.022217

C 2.507240 -1.995099 0.259723

C 3.314477 -0.854246 -0.377176

C 2.291017 0.251414 -0.731949

O 2.778288 1.414962 -0.091553

O 4.190120 -0.219049 0.536618

C 4.136230 1.174844 0.266210

C 4.451478 1.932387 1.530734

C 5.055941 1.535164 -0.888750

N -2.627278 0.406327 -0.461872

C -0.350966 1.800891 -0.810163

O -0.335683 2.517392 -1.777918

C -3.903614 -0.095494 -0.136152

C -4.460462 0.190716 1.108731

C -5.734824 -0.264803 1.415538

C -6.465866 -0.993482 0.484273

C -5.911958 -1.266085 -0.760704

C -4.635386 -0.820300 -1.074789

O -0.393674 2.232153 0.444612

C -0.517481 3.652306 0.646905

C -1.954173 4.092333 0.486767

O 2.935154 -3.283565 -0.091379

C 4.195838 -3.604427 0.460686

H 0.074180 -3.539635 0.448123

H 0.110946 -2.208753 1.616888

H 1.035880 -2.064833 -1.290739

H 0.921701 0.053687 0.898822

H -0.185045 0.026312 -1.937686

H 2.541973 -1.868718 1.351568

H 3.854939 -1.220256 -1.254704

H 2.216317 0.406154 -1.812501

H 4.349340 3.003227 1.354680

H 5.475432 1.725521 1.842033

H 3.764305 1.629057 2.320285

H 4.786813 0.973865 -1.785342

H 4.974252 2.600128 -1.107428

H 6.089326 1.305600 -0.627296

H -3.886016 0.762778 1.827157

H -6.159267 -0.045926 2.387592

H -7.461712 -1.342145 0.725804

H -6.475183 -1.829494 -1.494408

H -4.198340 -1.026420 -2.044652

H -0.161722 3.816190 1.662007

H 0.143858 4.165813 -0.049825

H -2.030387 5.162265 0.686199

H -2.309725 3.902826 -0.526080

H -2.597059 3.563253 1.191373

H 4.171636 -3.538666 1.553983

H 4.430569 -4.626140 0.166848

H 4.978563 -2.936923 0.085658

==============================

**conf042_E0.99**

C 0.017355 -2.456902 0.558802

C 1.085115 -1.753759 -0.240731

C 0.960129 -0.236593 -0.156147

C -0.274085 0.286386 -0.878566

C -1.588862 -0.314740 -0.409202

S -1.672550 -2.034600 0.021914

C 2.507231 -1.995108 0.259744

C 3.314487 -0.854239 -0.377107

C 2.291033 0.251381 -0.731987

O 2.778279 1.414994 -0.091708

O 4.190016 -0.219002 0.536764

C 4.136164 1.174874 0.266303

C 4.451187 1.932459 1.530856

C 5.056062 1.535165 -0.888513

N -2.627246 0.406352 -0.461706

C -0.350970 1.800879 -0.810251

O -0.335763 2.517356 -1.778024

C -3.903590 -0.095484 -0.136013

C -4.460495 0.190874 1.108807

C -5.734862 -0.264644 1.415619

C -6.465848 -0.993447 0.484411

C -5.911879 -1.266193 -0.760512

C -4.635300 -0.820428 -1.074595

O -0.393584 2.232181 0.444509

C -0.517434 3.652334 0.646748

C -1.954138 4.092313 0.486581

O 2.935189 -3.283566 -0.091321

C 4.195868 -3.604383 0.460789

H 0.074196 -3.539687 0.447971

H 0.110823 -2.208813 1.616744

H 1.035967 -2.064825 -1.290811

H 0.921703 0.053651 0.898787

H -0.185025 0.026262 -1.937726

H 2.541892 -1.868724 1.351590

H 3.855047 -1.220249 -1.254573

H 2.216333 0.406020 -1.812555

H 5.475087 1.725602 1.842339

H 3.763872 1.629153 2.320295

H 4.349075 3.003292 1.354742

H 4.787074 0.973833 -1.785127

H 4.974404 2.600123 -1.107237

H 6.089404 1.305608 -0.626880

H -3.886091 0.763035 1.827187

H -6.159353 -0.045649 2.387627

H -7.461700 -1.342098 0.725939

H -6.475065 -1.829702 -1.494170

H -4.198211 -1.026656 -2.044416

H -0.161687 3.816267 1.661845

H 0.143893 4.165831 -0.049998

H -2.309669 3.902797 -0.526272

H -2.597018 3.563214 1.191179

H -2.030378 5.162242 0.686018

H 4.430462 -4.626231 0.167311

H 4.978650 -2.937104 0.085480

H 4.171737 -3.538223 1.554064

==============================

**conf043_E1.00**

C -0.142218 -2.403940 0.574242

C 0.913917 -1.792130 -0.310233

C 0.869443 -0.268660 -0.279884

C -0.366721 0.290257 -0.971947

C -1.688852 -0.230396 -0.428781

S -1.833019 -1.920887 0.095684

C 2.348756 -2.092303 0.112406

C 3.171348 -1.034496 -0.645834

C 2.199218 0.130170 -0.936947

O 2.831302 1.272038 -0.403237

O 4.236066 -0.442253 0.071172

C 3.867198 0.869821 0.486671

C 3.387486 0.862206 1.929786

C 5.047862 1.792863 0.291158

N -2.695226 0.535086 -0.478966

C -0.366834 1.809302 -0.951972

O -0.374892 2.492915 -1.943265

C -3.981122 0.113051 -0.086284

C -4.747946 -0.698366 -0.920535

C -6.032456 -1.062140 -0.539901

C -6.559201 -0.622766 0.668608

C -5.793889 0.192513 1.494594

C -4.512239 0.568777 1.118598

O -0.328586 2.284124 0.287541

C -0.323870 3.716459 0.444138

C 1.064452 4.284359 0.265199

O 2.681282 -3.418791 -0.201904

C 3.964933 -3.781225 0.264879

H -0.144400 -3.492205 0.513496

H 0.016714 -2.112167 1.613253

H 0.786567 -2.138689 -1.342688

H 0.877262 0.061955 0.762106

H -0.334279 -0.007059 -2.024580

H 2.463956 -1.924431 1.192920

H 3.574731 -1.482611 -1.556179

H 2.058561 0.290373 -2.007967

H 2.566775 0.161412 2.075112

H 3.052152 1.860996 2.211982

H 4.209850 0.570498 2.584285

H 5.878046 1.473504 0.921381

H 5.358103 1.773348 -0.753209

H 4.771326 2.811206 0.566438

H -4.332648 -1.035111 -1.863000

H -6.623409 -1.692827 -1.192558

H -7.560923 -0.908971 0.962326

H -6.197411 0.542662 2.436631

H -3.911912 1.210509 1.751982

H -1.030142 4.147106 -0.264896

H -0.690907 3.879754 1.455439

H 1.036729 5.360504 0.443990

H 1.757527 3.831929 0.974885

H 1.432115 4.108431 -0.744979

H 4.749415 -3.192218 -0.221068

H 4.040816 -3.640305 1.348809

H 4.113309 -4.833399 0.027692

==============================

**conf044_E1.04**

C 0.153052 -2.405467 -0.689165

C -0.944150 -1.822433 0.165462

C -0.877821 -0.301169 0.219505

C 0.335693 0.208043 0.985676

C 1.672076 -0.292448 0.463403

S 1.823615 -1.967907 -0.105860

C -2.354207 -2.070287 -0.366554

C -3.208139 -1.010675 0.347525

C -2.225600 0.080832 0.833257

O -2.743724 1.293327 0.320779

O -4.067744 -0.312443 -0.535462

C -4.075988 1.046402 -0.122427

C -4.364634 1.920187 -1.316465

C -5.055259 1.255867 1.020412

N 2.676612 0.469739 0.566368

C 0.335262 1.725040 1.046898

O 0.295733 2.358550 2.070378

C 3.972999 0.060630 0.194880

C 4.720238 -0.772034 1.025698

C 6.015629 -1.121315 0.668847

C 6.572183 -0.646523 -0.512516

C 5.825802 0.189415 -1.335209

C 4.533491 0.551390 -0.982629

O 0.335087 2.266199 -0.164080

C 0.275173 3.702011 -0.229353

C 0.228151 4.087807 -1.684897

O -2.741508 -3.395859 -0.122335

C -3.978890 -3.716997 -0.724768

H 0.140689 -3.495327 -0.680817

H 0.051793 -2.063965 -1.719993

H -0.886625 -2.226716 1.182718

H -0.855037 0.084062 -0.805121

H 0.262062 -0.146449 2.018354

H -2.383994 -1.860101 -1.445492

H -3.767952 -1.466611 1.168803

H -2.161444 0.117952 1.924827

H -4.304992 2.969868 -1.028563

H -5.367501 1.717440 -1.692470

H -3.635690 1.719342 -2.101381

H -5.020770 2.294265 1.350741

H -6.068073 1.022356 0.690753

H -4.802732 0.612877 1.865436

H 4.281863 -1.135656 1.947491

H 6.591543 -1.768396 1.318883

H 7.582343 -0.921243 -0.787694

H 6.252675 0.567041 -2.256085

H 3.947703 1.209325 -1.613096

H -0.613728 4.035432 0.307642

H 1.154576 4.108969 0.271874

H 1.118272 3.736615 -2.208103

H -0.654244 3.665485 -2.167409

H 0.182799 5.173869 -1.770651

H -4.184132 -4.764653 -0.511281

H -4.792034 -3.105118 -0.320392

H -3.934612 -3.570151 -1.809496

==============================

**conf045_E1.05**

C 0.152972 -2.405476 -0.689133

C -0.944248 -1.822436 0.165464

C -0.877878 -0.301172 0.219540

C 0.335644 0.208021 0.985703

C 1.672023 -0.292516 0.463444

S 1.823520 -1.968013 -0.105713

C -2.354290 -2.070214 -0.366625

C -3.208212 -1.010612 0.347482

C -2.225653 0.080845 0.833288

O -2.743726 1.293382 0.320864

O -4.067774 -0.312313 -0.535486

C -4.075986 1.046517 -0.122375

C -4.364546 1.920370 -1.316376

C -5.055301 1.255979 1.020438

N 2.676564 0.469674 0.566346

C 0.335265 1.725023 1.046879

O 0.295767 2.358567 2.070339

C 3.972950 0.060547 0.194891

C 4.720156 -0.772160 1.025693

C 6.015551 -1.121448 0.668857

C 6.572135 -0.646632 -0.512479

C 5.825784 0.189346 -1.335161

C 4.533478 0.551338 -0.982591

O 0.335096 2.266140 -0.164118

C 0.275439 3.701961 -0.229430

C 0.228962 4.087743 -1.684995

O -2.741646 -3.395787 -0.122506

C -3.979043 -3.716829 -0.724958

H 0.140566 -3.495337 -0.680842

H 0.051784 -2.063922 -1.719950

H -0.886778 -2.226745 1.182712

H -0.855097 0.084067 -0.805083

H 0.262006 -0.146438 2.018392

H -2.384019 -1.859959 -1.445552

H -3.768052 -1.466570 1.168731

H -2.161514 0.117906 1.924861

H -4.304850 2.970030 -1.028409

H -5.367414 1.717700 -1.692418

H -3.635590 1.719529 -2.101283

H -6.068108 1.022515 0.690727

H -4.802834 0.612950 1.865448

H -5.020793 2.294363 1.350809

H 4.281762 -1.135803 1.947469

H 6.591441 -1.768556 1.318887

H 7.582296 -0.921359 -0.787646

H 6.252683 0.566992 -2.256016

H 3.947714 1.209306 -1.613045

H -0.613590 4.035530 0.307265

H 1.154740 4.108782 0.272085

H -0.653346 3.665571 -2.167796

H 0.183829 5.173812 -1.770780

H 1.119196 3.736386 -2.207898

H -3.934786 -3.569835 -1.809667

H -4.184286 -4.764514 -0.511612

H -4.792173 -3.104999 -0.320482

==============================

**conf046_E1.05**

C -0.006300 -2.549800 0.072965

C 1.022155 -1.670538 -0.590254

C 0.939744 -0.228737 -0.105153

C -0.324526 0.483981 -0.562914

C -1.627186 -0.228919 -0.229729

S -1.713134 -2.001114 -0.249387

C 2.469868 -2.037866 -0.277796

C 3.253133 -0.781503 -0.699834

C 2.241873 0.384500 -0.643541

O 2.856850 1.358814 0.168389

O 4.304709 -0.386836 0.158846

C 3.914206 0.759477 0.910413

C 3.454111 0.358899 2.303070

C 5.073446 1.728298 0.956748

N -2.662127 0.480303 -0.063530

C -0.360967 1.878331 0.041422

O -0.325101 2.094102 1.224414

C -3.933131 -0.088149 0.155395

C -4.478763 -0.074692 1.437503

C -5.747577 -0.593501 1.653271

C -6.485349 -1.112823 0.595639

C -5.943968 -1.111101 -0.684217

C -4.672148 -0.601834 -0.908688

O -0.395878 2.826530 -0.887727

C -0.393850 4.186855 -0.410474

C -0.424021 5.091622 -1.614406

O 2.834889 -3.206250 -0.963480

C 4.131280 -3.653032 -0.621915

H 0.025244 -3.571335 -0.305744

H 0.151108 -2.572249 1.152081

H 0.893116 -1.697092 -1.678844

H 0.967913 -0.225456 0.988003

H -0.299592 0.568510 -1.653625

H 2.592378 -2.185147 0.805064

H 3.661373 -0.935294 -1.700970

H 2.068645 0.824822 -1.627820

H 3.093626 1.238574 2.837783

H 4.292258 -0.068962 2.854513

H 2.654991 -0.379596 2.262166

H 4.783633 2.628896 1.498735

H 5.919774 1.269689 1.468412

H 5.366755 1.996193 -0.057963

H -3.899832 0.338853 2.254264

H -6.162452 -0.588142 2.653650

H -7.477165 -1.511079 0.766887

H -6.513311 -1.508769 -1.515275

H -4.245305 -0.595048 -1.904634

H -1.266324 4.329055 0.228233

H 0.503012 4.338940 0.191270

H -0.423322 6.130931 -1.284675

H 0.451371 4.928299 -2.243832

H -1.322487 4.917852 -2.207376

H 4.209854 -3.837752 0.455187

H 4.308228 -4.582854 -1.159953

H 4.895627 -2.923829 -0.909213

==============================

**conf047_E1.06**

C -0.006435 -2.549834 0.073018

C 1.022054 -1.670564 -0.590126

C 0.939653 -0.228792 -0.105018

C -0.324601 0.483935 -0.562852

C -1.627277 -0.228879 -0.229642

S -1.713265 -2.001165 -0.249525

C 2.469745 -2.037959 -0.277671

C 3.253044 -0.781616 -0.699728

C 2.241808 0.384441 -0.643376

O 2.856823 1.358679 0.168625

O 4.304698 -0.386942 0.158820

C 3.914252 0.759311 0.910493

C 3.454292 0.358633 2.303178

C 5.073473 1.728130 0.956783

N -2.662159 0.480357 -0.063244

C -0.360970 1.878333 0.041333

O -0.324945 2.094235 1.224293

C -3.933241 -0.087973 0.155549

C -4.671843 -0.602377 -0.908490

C -5.943742 -1.111503 -0.684192

C -6.485623 -1.112380 0.595464

C -5.748286 -0.592316 1.653030

C -4.479393 -0.073616 1.437413

O -0.396027 2.826447 -0.887914

C -0.393570 4.186806 -0.410808

C -0.422331 5.091476 -1.614848

O 2.834698 -3.206337 -0.963378

C 4.131063 -3.653204 -0.621814

H 0.025143 -3.571375 -0.305671

H 0.150869 -2.572242 1.152146

H 0.893043 -1.697085 -1.678725

H 0.967789 -0.225485 0.988136

H -0.299623 0.568318 -1.653573

H 2.592265 -2.185257 0.805185

H 3.661178 -0.935439 -1.700898

H 2.068616 0.824821 -1.627628

H 3.094070 1.238303 2.838089

H 4.292423 -0.069491 2.854442

H 2.654983 -0.379660 2.262277

H 5.919787 1.269612 1.468555

H 5.366835 1.995937 -0.057942

H 4.783606 2.628785 1.498657

H -4.244619 -0.596196 -1.904280

H -6.512761 -1.509723 -1.515209

H -7.477500 -1.510535 0.766606

H -6.163568 -0.586277 2.653238

H -3.900822 0.340548 2.254120

H -1.266455 4.329567 0.227219

H 0.502900 4.338461 0.191612

H -0.421240 6.130820 -1.285214

H 0.453431 4.927564 -2.243615

H -1.320464 4.918206 -2.208474

H 4.308032 -4.582944 -1.159987

H 4.895444 -2.923976 -0.908972

H 4.209577 -3.838087 0.455261

==============================

**conf048_E1.06**

C -0.006350 -2.549858 0.073011

C 1.022119 -1.670568 -0.590136

C 0.939678 -0.228795 -0.105033

C -0.324592 0.483899 -0.562879

C -1.627252 -0.228942 -0.229664

S -1.713195 -2.001224 -0.249514

C 2.469820 -2.037926 -0.277675

C 3.253092 -0.781558 -0.699705

C 2.241822 0.384470 -0.643380

O 2.856799 1.358735 0.168610

O 4.304708 -0.386854 0.158880

C 3.914211 0.759402 0.910526

C 3.454220 0.358734 2.303204

C 5.073410 1.728250 0.956840

N -2.662140 0.480289 -0.063270

C -0.360994 1.878301 0.041302

O -0.324973 2.094210 1.224261

C -3.933224 -0.088039 0.155545

C -4.671829 -0.602502 -0.908465

C -5.943727 -1.111616 -0.684135

C -6.485605 -1.112422 0.595524

C -5.748268 -0.592295 1.653058

C -4.479375 -0.073606 1.437408

O -0.396088 2.826408 -0.887951

C -0.393693 4.186772 -0.410860

C -0.422669 5.091426 -1.614908

O 2.834805 -3.206293 -0.963379

C 4.131196 -3.653106 -0.621843

H 0.025243 -3.571398 -0.305678

H 0.150963 -2.572264 1.152138

H 0.893112 -1.697094 -1.678735

H 0.967796 -0.225484 0.988121

H -0.299617 0.568286 -1.653599

H 2.592330 -2.185223 0.805182

H 3.661263 -0.935360 -1.700863

H 2.068625 0.824826 -1.627642

H 3.094002 1.238410 2.838107

H 4.292341 -0.069397 2.854477

H 2.654904 -0.379551 2.262296

H 5.919713 1.269759 1.468657

H 5.366807 1.996035 -0.057880

H 4.783499 2.628911 1.498679

H -4.244615 -0.596370 -1.904258

H -6.512749 -1.509883 -1.515128

H -7.477482 -1.510568 0.766690

H -6.163551 -0.586197 2.653265

H -3.900804 0.340607 2.254089

H -1.266518 4.329466 0.227263

H 0.502834 4.338512 0.191455

H 0.453017 4.927554 -2.243791

H -1.320873 4.918098 -2.208408

H -0.421589 6.130775 -1.285286

H 4.308215 -4.582809 -1.160063

H 4.895537 -2.923823 -0.908962

H 4.209723 -3.838040 0.455224

==============================

**conf049_E1.06**

C -0.006364 -2.549854 0.073016

C 1.022109 -1.670571 -0.590131

C 0.939682 -0.228796 -0.105027

C -0.324583 0.483908 -0.562874

C -1.627245 -0.228925 -0.229658

S -1.713208 -2.001199 -0.249494

C 2.469807 -2.037947 -0.277672

C 3.253091 -0.781582 -0.699690

C 2.241828 0.384454 -0.643388

O 2.856812 1.358734 0.168572

O 4.304704 -0.386871 0.158898

C 3.914207 0.759406 0.910514

C 3.454189 0.358796 2.303202

C 5.073419 1.728238 0.956817

N -2.662129 0.480317 -0.063265

C -0.360984 1.878308 0.041314

O -0.324944 2.094213 1.224272

C -3.933209 -0.088032 0.155550

C -4.671811 -0.602463 -0.908475

C -5.943705 -1.111594 -0.684158

C -6.485576 -1.112448 0.595503

C -5.748235 -0.592358 1.653055

C -4.479347 -0.073654 1.437419

O -0.396091 2.826415 -0.887939

C -0.393710 4.186780 -0.410851

C -0.422707 5.091429 -1.614903

O 2.834780 -3.206315 -0.963376

C 4.131181 -3.653124 -0.621869

H 0.025209 -3.571392 -0.305681

H 0.150952 -2.572270 1.152142

H 0.893098 -1.697095 -1.678729

H 0.967802 -0.225484 0.988127

H -0.299604 0.568304 -1.653592

H 2.592307 -2.185253 0.805186

H 3.661273 -0.935395 -1.700842

H 2.068628 0.824787 -1.627660

H 3.094013 1.238509 2.838075

H 4.292282 -0.069366 2.854493

H 2.654829 -0.379441 2.262313

H 4.783512 2.628915 1.498631

H 5.919708 1.269742 1.468651

H 5.366828 1.995993 -0.057908

H -4.244591 -0.596296 -1.904266

H -6.512730 -1.509835 -1.515162

H -7.477450 -1.510603 0.766662

H -6.163516 -0.586307 2.653264

H -3.900769 0.340526 2.254113

H -1.266534 4.329461 0.227276

H 0.502817 4.338536 0.191457

H -0.421588 6.130779 -1.285289

H 0.452953 4.927527 -2.243815

H -1.320936 4.918117 -2.208373

H 4.308199 -4.582813 -1.160112

H 4.895510 -2.923827 -0.908981

H 4.209726 -3.838082 0.455193

==============================

**conf050_E1.09**

C -0.004137 -0.662327 -2.061843

C -0.923281 -0.938044 -0.896207

C -1.117060 0.303633 -0.032697

C 0.183398 0.918258 0.489969

C 1.412733 0.061842 0.233572

S 1.709738 -0.460763 -1.446967

C -2.364671 -1.325720 -1.200384

C -3.055591 -1.130679 0.163431

C -2.148553 -0.175578 0.982690

O -3.012599 0.854786 1.423415

O -4.278093 -0.423621 0.068930

C -4.354805 0.428943 1.202285

C -5.200831 1.627537 0.854982

C -4.868957 -0.330771 2.413936

N 2.199969 -0.188735 1.190600

C 0.462078 2.283401 -0.144240

O -0.177727 2.774305 -1.036743

C 3.401382 -0.900444 1.006371

C 4.442845 -0.368520 0.248046

C 5.636741 -1.066559 0.126023

C 5.798160 -2.296391 0.751778

C 4.760262 -2.820695 1.514313

C 3.569606 -2.122724 1.654476

O 1.516049 2.858306 0.422631

C 1.957000 4.115914 -0.131748

C 2.794060 3.890987 -1.368673

O -2.428739 -2.634085 -1.700642

C -3.734584 -2.995315 -2.101511

H 0.048590 -1.501134 -2.755134

H -0.313442 0.231389 -2.604679

H -0.498358 -1.746933 -0.288323

H -1.616373 1.051091 -0.654684

H 0.143442 1.075677 1.569439

H -2.805592 -0.621832 -1.920130

H -3.193695 -2.099881 0.651052

H -1.677030 -0.676937 1.832172

H -4.786090 2.126486 -0.020642

H -5.217378 2.323822 1.693453

H -6.222418 1.312213 0.642378

H -5.879271 -0.694849 2.224789

H -4.223256 -1.182061 2.636269

H -4.887047 0.328004 3.282457

H 4.314077 0.595130 -0.231147

H 6.442687 -0.646124 -0.462598

H 6.729010 -2.839773 0.652374

H 4.879914 -3.776404 2.009356

H 2.760883 -2.517050 2.257399

H 1.084537 4.733524 -0.339410

H 2.538384 4.574382 0.665084

H 3.663504 3.274947 -1.135816

H 2.212934 3.402803 -2.151464

H 3.144615 4.851965 -1.747845

H -3.685864 -4.006567 -2.501806

H -4.431165 -2.979934 -1.256745

H -4.107093 -2.316632 -2.876667

==============================

**conf051_E1.10**

C -0.179430 -2.513316 0.246128

C 0.887940 -1.745604 -0.490225

C 0.867971 -0.262906 -0.140859

C -0.362147 0.459083 -0.671556

C -1.695585 -0.161355 -0.280432

S -1.859948 -1.922429 -0.135557

C 2.317676 -2.146205 -0.138584

C 3.156557 -0.968086 -0.666477

C 2.197629 0.241324 -0.723311

O 2.851786 1.256412 0.003281

O 4.221220 -0.543493 0.161427

C 3.877713 0.680872 0.804929

C 3.393600 0.426104 2.223528

C 5.077183 1.600096 0.775339

N -2.698669 0.604631 -0.187787

C -0.336093 1.904934 -0.201267

O -0.304419 2.226434 0.957529

C -3.994168 0.116252 0.075555

C -4.542170 0.275463 1.346750

C -5.833679 -0.163498 1.601612

C -6.591123 -0.747393 0.592761

C -6.046708 -0.891071 -0.677688

C -4.752644 -0.462660 -0.940198

O -0.319807 2.758376 -1.219822

C -0.236500 4.161780 -0.894937

C 1.188312 4.565473 -0.596368

O 2.632144 -3.385836 -0.715727

C 3.908627 -3.854315 -0.330885

H -0.192308 -3.566041 -0.035616

H -0.024687 -2.442221 1.323552

H 0.759068 -1.867191 -1.572443

H 0.892211 -0.158806 0.947373

H -0.331471 0.438722 -1.765184

H 2.432863 -2.202010 0.953693

H 3.561913 -1.229500 -1.646165

H 2.047856 0.599353 -1.744239

H 3.067517 1.364382 2.674279

H 4.209955 0.014999 2.818715

H 2.564254 -0.279157 2.241645

H 5.900145 1.155064 1.334831

H 5.387787 1.762755 -0.256411

H 4.822150 2.557468 1.230792

H -3.947990 0.739119 2.124743

H -6.250726 -0.044475 2.593992

H -7.600375 -1.082832 0.794057

H -6.630932 -1.340027 -1.471425

H -4.323118 -0.569210 -1.929281

H -0.616327 4.667782 -1.779981

H -0.898695 4.365774 -0.054551

H 1.564232 4.046813 0.284861

H 1.835965 4.337121 -1.443923

H 1.226976 5.640197 -0.412432

H 3.980062 -3.943314 0.758825

H 4.045437 -4.835957 -0.781419

H 4.702935 -3.187343 -0.681262

==============================

**conf052_E1.12**

C -0.004247 -0.662725 -2.061844

C -0.923379 -0.938291 -0.896160

C -1.117089 0.303458 -0.032754

C 0.183373 0.918147 0.489820

C 1.412709 0.061744 0.233462

S 1.709639 -0.461088 -1.447043

C -2.364786 -1.325937 -1.200257

C -3.055705 -1.130661 0.163528

C -2.148515 -0.175661 0.982740

O -3.012417 0.854763 1.423599

O -4.278045 -0.423322 0.068924

C -4.354713 0.429175 1.202308

C -5.200428 1.627972 0.855020

C -4.869063 -0.330538 2.413866

N 2.200042 -0.188666 1.190456

C 0.461982 2.283251 -0.144480

O -0.177774 2.773992 -1.037107

C 3.401509 -0.900255 1.006319

C 3.570016 -2.122234 1.654924

C 4.760722 -2.820146 1.514845

C 5.798430 -2.296059 0.751910

C 5.636756 -1.066496 0.125669

C 4.442805 -0.368546 0.247588

O 1.515831 2.858314 0.422467

C 1.956683 4.115964 -0.131894

C 2.794021 3.891103 -1.368636

O -2.428931 -2.634390 -1.700314

C -3.734791 -2.995562 -2.101188

H 0.048465 -1.501615 -2.755038

H -0.313572 0.230918 -2.604787

H -0.498472 -1.747136 -0.288205

H -1.616444 1.050866 -0.654772

H 0.143439 1.075642 1.569280

H -2.805678 -0.622164 -1.920126

H -3.194050 -2.099787 0.651219

H -1.676940 -0.677099 1.832144

H -6.222118 1.312959 0.642432

H -4.785564 2.126825 -0.020604

H -5.216785 2.324257 1.693500

H -4.223447 -1.181898 2.636195

H -4.887195 0.328162 3.282444

H -5.879391 -0.694524 2.224596

H 2.761458 -2.516381 2.258190

H 4.880542 -3.775627 2.010291

H 6.729329 -2.839368 0.652563

H 6.442560 -0.646210 -0.463257

H 4.313881 0.594893 -0.231992

H 1.084160 4.733423 -0.339749

H 2.537831 4.574586 0.665019

H 3.663413 3.275054 -1.135595

H 2.213091 3.402955 -2.151594

H 3.144687 4.852096 -1.747676

H -3.686045 -4.006644 -2.501914

H -4.431276 -2.980610 -1.256330

H -4.107450 -2.316588 -2.876012

==============================

**conf053_E1.12** The lowest energy conformer

Sum of electronic and thermal Free Energies= -1645.165289 (Hartree/Particle)

C -0.204678000 2.587724000 -0.178535000

C 0.882418000 1.760183000 0.458032000

C 0.862090000 0.317067000 -0.030943000

C -0.355962000 -0.459706000 0.449603000

C -1.702774000 0.190320000 0.171514000

S -1.874009000 1.955404000 0.180137000

C 2.300367000 2.204641000 0.107542000

C 3.160713000 0.983276000 0.463230000

C 2.201437000 -0.230911000 0.467935000

O 2.788624000 -1.160933000 -0.420897000

O 4.105477000 0.658534000 -0.540087000

C 4.138357000 -0.758538000 -0.637833000

C 4.547798000 -1.144353000 -2.036030000

C 5.040474000 -1.350768000 0.432025000

N -2.711180000 -0.567232000 0.067172000

C -0.333487000 -1.848309000 -0.168097000

O -0.381527000 -2.055541000 -1.351944000

C -4.016294000 -0.058510000 -0.089119000

C -4.614392000 -0.069872000 -1.347576000

C -5.915792000 0.387814000 -1.497600000

C -6.632907000 0.844959000 -0.398072000

C -6.037771000 0.842810000 0.857642000

C -4.733980000 0.393666000 1.016632000

O -0.202589000 -2.799678000 0.749768000

C -0.101893000 -4.150651000 0.257876000

C 0.080256000 -5.055338000 1.448375000

O 2.629641000 3.376720000 0.803167000

C 3.882984000 3.900801000 0.414973000

H -0.214265000 3.608105000 0.202206000

H -0.072765000 2.620101000 -1.259965000

H 0.779984000 1.778358000 1.548685000

H 0.882878000 0.318290000 -1.125495000

H -0.292926000 -0.554442000 1.537179000

H 2.377838000 2.373693000 -0.975653000

H 3.646409000 1.135547000 1.430408000

H 2.098011000 -0.662098000 1.467355000

H 5.563635000 -0.803066000 -2.231995000

H 3.867970000 -0.690197000 -2.755507000

H 4.516662000 -2.227859000 -2.144347000

H 5.024246000 -2.438392000 0.368287000

H 6.063571000 -1.003144000 0.290821000

H 4.702622000 -1.055029000 1.426180000

H -4.051397000 -0.434506000 -2.197292000

H -6.371898000 0.383459000 -2.479176000

H -7.649163000 1.195742000 -0.518311000

H -6.589660000 1.192583000 1.720575000

H -4.265097000 0.386497000 1.992718000

H -1.010659000 -4.384741000 -0.296377000

H 0.743616000 -4.204305000 -0.427827000

H 0.160083000 -6.087717000 1.108725000

H 0.989224000 -4.799804000 1.992785000

H -0.769659000 -4.979768000 2.126319000

H 4.042332000 4.818689000 0.976987000

H 4.694915000 3.201715000 0.637255000

H 3.897095000 4.125787000 -0.656444000

==============================

conf054_E1.13

C 0.017396 -2.456910 0.558853

C 1.085145 -1.753813 -0.240742

C 0.960156 -0.236642 -0.156208

C -0.274084 0.286334 -0.878579

C -1.588846 -0.314785 -0.409190

S -1.672523 -2.034573 0.022077

C 2.507270 -1.995128 0.259734

C 3.314519 -0.854215 -0.377075

C 2.291030 0.251312 -0.732111

O 2.778225 1.415036 -0.091985

O 4.189892 -0.218890 0.536850

C 4.136057 1.174981 0.266257

C 4.450881 1.932679 1.530796

C 5.056143 1.535170 -0.888447

N -2.627242 0.406287 -0.461920

C -0.350927 1.800831 -0.810202

O -0.335559 2.517348 -1.777947

C -3.903598 -0.095520 -0.136127

C -4.460211 0.190406 1.108908

C -5.734605 -0.265028 1.415762

C -6.465864 -0.993335 0.484394

C -5.912158 -1.265680 -0.760736

C -4.635581 -0.819970 -1.074879

O -0.393650 2.232062 0.444565

C -0.517460 3.652214 0.646898

C -1.954177 4.092192 0.486828

O 2.935281 -3.283575 -0.091368

C 4.195969 -3.604356 0.460733

H 0.074210 -3.539699 0.448045

H 0.110935 -2.208813 1.616789

H 1.035986 -2.064926 -1.290806

H 0.921799 0.053627 0.898725

H -0.185041 0.026233 -1.937749

H 2.541913 -1.868792 1.351585

H 3.855178 -1.220243 -1.254480

H 2.216321 0.405806 -1.812700

H 5.474718 1.725812 1.842470

H 3.763421 1.629476 2.320142

H 4.348840 3.003497 1.354560

H 4.974565 2.600111 -1.107261

H 6.089433 1.305585 -0.626635

H 4.787276 0.973787 -1.785062

H -3.885594 0.762180 1.827423

H -6.158886 -0.046345 2.387929

H -7.461723 -1.341935 0.725955

H -6.475576 -1.828803 -1.494510

H -4.198689 -1.025870 -2.044854

H -0.161654 3.816083 1.661986

H 0.143830 4.165735 -0.049869

H -2.309701 3.902821 -0.526053

H -2.597026 3.562934 1.191334

H -2.030480 5.162079 0.686452

H 4.430678 -4.626127 0.167075

H 4.978698 -2.936930 0.085577

H 4.171790 -3.538393 1.554019

==============================

conf055_E1.13

C 0.017400 -2.456851 0.559011

C 1.085119 -1.753765 -0.240626

C 0.960137 -0.236592 -0.156118

C -0.274111 0.286357 -0.878498

C -1.588881 -0.314798 -0.409134

S -1.672533 -2.034556 0.022242

C 2.507257 -1.995066 0.259796

C 3.314481 -0.854254 -0.377218

C 2.291017 0.251367 -0.732039

O 2.778299 1.414966 -0.091771

O 4.190159 -0.219015 0.536489

C 4.136219 1.174880 0.266108

C 4.451333 1.932406 1.530681

C 5.056004 1.535241 -0.888783

N -2.627276 0.406288 -0.461815

C -0.350983 1.800854 -0.810134

O -0.335774 2.517360 -1.777886

C -3.903648 -0.095529 -0.136124

C -4.635309 -0.820502 -1.074730

C -5.911919 -1.266209 -0.760723

C -6.465973 -0.993367 0.484146

C -5.735044 -0.264538 1.415364

C -4.460627 0.190914 1.108627

O -0.393567 2.232110 0.444639

C -0.517415 3.652264 0.646942

C -1.954133 4.092235 0.486882

O 2.935208 -3.283562 -0.091156

C 4.195967 -3.604270 0.460832

H 0.074227 -3.539641 0.448225

H 0.110954 -2.208723 1.616938

H 1.035921 -2.064885 -1.290687

H 0.921777 0.053708 0.898803

H -0.185062 0.026259 -1.937666

H 2.541982 -1.868576 1.351628

H 3.854902 -1.220355 -1.254737

H 2.216257 0.406019 -1.812601

H 5.475209 1.725411 1.842147

H 3.763985 1.629154 2.320109

H 4.349352 3.003256 1.354602

H 6.089383 1.305763 -0.627233

H 4.786981 0.973847 -1.785349

H 4.974239 2.600181 -1.107530

H -4.198144 -1.026792 -2.044500

H -6.475078 -1.829736 -1.494384

H -7.461856 -1.341969 0.725613

H -6.159609 -0.045461 2.387319

H -3.886270 0.763090 1.827029

H -0.161606 3.816152 1.662026

H 0.143866 4.165787 -0.049830

H -2.030393 5.162144 0.686409

H -2.309708 3.902787 -0.525967

H -2.596967 3.563066 1.191468

H 4.978716 -2.937144 0.085186

H 4.172030 -3.537777 1.554091

H 4.430448 -4.626222 0.167623

==============================

conf056_E1.14

C -0.004101 -0.662298 -2.061886

C -0.923235 -0.938053 -0.896247

C -1.117018 0.303614 -0.032727

C 0.183422 0.918238 0.489984

C 1.412759 0.061866 0.233557

S 1.709769 -0.460685 -1.447012

C -2.364624 -1.325716 -1.200423

C -3.055524 -1.130738 0.163413

C -2.148519 -0.175593 0.982652

O -3.012600 0.854760 1.423345

O -4.278080 -0.423765 0.068970

C -4.354796 0.428834 1.202297

C -5.200894 1.627377 0.855002

C -4.868814 -0.330896 2.413990

N 2.200011 -0.188753 1.190557

C 0.462021 2.283468 -0.144071

O -0.178036 2.774581 -1.036276

C 3.401407 -0.900462 1.006334

C 3.569638 -2.122724 1.654471

C 4.760287 -2.820715 1.514313

C 5.798177 -2.296450 0.751749

C 5.636753 -1.066630 0.125955

C 4.442871 -0.368578 0.247971

O 1.516203 2.858197 0.422591

C 1.957016 4.115944 -0.131577

C 2.793640 3.891336 -1.368852

O -2.428704 -2.634060 -1.700750

C -3.734578 -2.995298 -2.101527

H 0.048650 -1.501088 -2.755195

H -0.313445 0.231416 -2.604705

H -0.498301 -1.746952 -0.288383

H -1.616315 1.051082 -0.654716

H 0.143431 1.075579 1.569468

H -2.805546 -0.621799 -1.920140

H -3.193549 -2.099958 0.651021

H -1.676991 -0.676924 1.832148

H -5.217367 2.323721 1.693428

H -6.222505 1.312026 0.642541

H -4.786267 2.126277 -0.020704

H -5.879091 -0.695106 2.224894

H -4.222994 -1.182105 2.636310

H -4.886953 0.327887 3.282505

H 2.760928 -2.517020 2.257432

H 4.879924 -3.776407 2.009394

H 6.729024 -2.839836 0.652341

H 6.442695 -0.646223 -0.462692

H 4.314106 0.595053 -0.231261

H 1.084509 4.733652 -0.338759

H 2.538698 4.574175 0.665174

H 3.663050 3.275062 -1.136483

H 2.212195 3.403542 -2.151648

H 3.144256 4.852394 -1.747768

H -3.685769 -4.006317 -2.502403

H -4.430970 -2.980527 -1.256593

H -4.107387 -2.316250 -2.876215

==============================

conf057_E1.15

C -0.004125 -0.662166 -2.061930

C -0.923276 -0.937962 -0.896314

C -1.117039 0.303666 -0.032730

C 0.183418 0.918256 0.489988

C 1.412733 0.061851 0.233548

S 1.709744 -0.460644 -1.447031

C -2.364676 -1.325583 -1.200511

C -3.055559 -1.130678 0.163344

C -2.148524 -0.175599 0.982632

O -3.012586 0.854738 1.423401

O -4.278105 -0.423701 0.068966

C -4.354771 0.428798 1.202398

C -5.200930 1.627334 0.855251

C -4.868718 -0.331066 2.414038

N 2.199954 -0.188826 1.190560

C 0.462084 2.283477 -0.144063

O -0.177887 2.774601 -1.036325

C 3.401353 -0.900540 1.006329

C 3.569595 -2.122774 1.654518

C 4.760242 -2.820766 1.514372

C 5.798122 -2.296525 0.751772

C 5.636689 -1.066733 0.125932

C 4.442802 -0.368680 0.247932

O 1.516247 2.858181 0.422650

C 1.957133 4.115881 -0.131570

C 2.793892 3.891193 -1.368743

O -2.428779 -2.633898 -1.700905

C -3.734653 -2.995087 -2.101713

H 0.048606 -1.500919 -2.755287

H -0.313434 0.231588 -2.604703

H -0.498364 -1.746902 -0.288491

H -1.616347 1.051160 -0.654678

H 0.143438 1.075582 1.569473

H -2.805595 -0.621619 -1.920182

H -3.193567 -2.099934 0.650890

H -1.676993 -0.676988 1.832092

H -4.786342 2.126343 -0.020412

H -5.217422 2.323589 1.693750

H -6.222524 1.311945 0.642774

H -5.878997 -0.695272 2.224953

H -4.222871 -1.182282 2.636243

H -4.886823 0.327634 3.282617

H 2.760892 -2.517045 2.257503

H 4.879894 -3.776438 2.009488

H 6.728969 -2.839912 0.652378

H 6.442625 -0.646345 -0.462737

H 4.314033 0.594933 -0.231333

H 1.084648 4.733584 -0.338876

H 2.538734 4.574142 0.665222

H 3.663275 3.274931 -1.136241

H 2.212529 3.403351 -2.151568

H 3.144552 4.852224 -1.747684

H -3.685895 -4.006155 -2.502469

H -4.431091 -2.980166 -1.256820

H -4.107370 -2.316099 -2.876500

==============================

conf058_E1.16

C 0.067807 -2.551569 0.304937

C 1.103875 -1.699409 -0.381491

C 0.977112 -0.227110 -0.005222

C -0.284429 0.414453 -0.564433

C -1.584745 -0.302681 -0.226316

S -1.634500 -2.073728 -0.131441

C 2.544514 -2.017239 0.006979

C 3.320473 -0.776320 -0.455973

C 2.284956 0.373283 -0.529390

O 2.811016 1.383077 0.309789

O 4.263637 -0.322313 0.496985

C 4.190763 1.097106 0.513333

C 4.599300 1.593767 1.876552

C 5.023878 1.689934 -0.611080

N -2.636567 0.395921 -0.138875

C -0.397069 1.852925 -0.086147

O -0.367575 2.173178 1.072699

C -3.906474 -0.177016 0.071475

C -4.581186 -0.810494 -0.970660

C -5.854729 -1.321131 -0.759781

C -6.461389 -1.206195 0.485117

C -5.787959 -0.567626 1.520149

C -4.518577 -0.045362 1.316306

O -0.502509 2.705942 -1.100274

C -0.677425 4.097331 -0.763503

C -2.117154 4.383626 -0.405287

O 2.954813 -3.220847 -0.584456

C 4.242279 -3.620345 -0.160310

H 0.140373 -3.597996 0.008452

H 0.178201 -2.486997 1.388106

H 1.015663 -1.805847 -1.469270

H 0.977707 -0.139571 1.086303

H -0.217873 0.408637 -1.656635

H 2.627864 -2.085378 1.101436

H 3.795490 -0.971108 -1.421843

H 2.161883 0.743828 -1.551727

H 5.642901 1.342146 2.065654

H 3.971406 1.131797 2.637972

H 4.486800 2.676877 1.924131

H 6.073516 1.428021 -0.474941

H 4.689204 1.310569 -1.578215

H 4.926772 2.775880 -0.610145

H -4.103762 -0.893765 -1.939828

H -6.373744 -1.811904 -1.573815

H -7.454194 -1.606227 0.646187

H -6.254604 -0.470162 2.492567

H -3.990624 0.462246 2.114357

H 0.000687 4.349475 0.050707

H -0.375570 4.635122 -1.659782

H -2.776787 4.115134 -1.231358

H -2.416808 3.825548 0.481799

H -2.234988 5.448830 -0.201091

H 4.461949 -4.574028 -0.637230

H 5.004290 -2.891010 -0.453863

H 4.274237 -3.743780 0.927771

==============================

conf059_E1.17

C 0.067451 -2.551563 0.305182

C 1.103592 -1.699632 -0.381405

C 0.977014 -0.227280 -0.005336

C -0.284510 0.414432 -0.564442

C -1.584892 -0.302560 -0.226371

S -1.634821 -2.073582 -0.131222

C 2.544221 -2.017569 0.007072

C 3.320320 -0.776731 -0.455885

C 2.284858 0.372889 -0.529742

O 2.810921 1.382999 0.309036

O 4.263209 -0.322580 0.497270

C 4.190533 1.096854 0.513186

C 4.598630 1.593831 1.876422

C 5.024176 1.689235 -0.611065

N -2.636672 0.396137 -0.139121

C -0.396838 1.852877 -0.085960

O -0.367636 2.172923 1.072951

C -3.906624 -0.176736 0.071235

C -4.518662 -0.045151 1.316102

C -5.788072 -0.567353 1.519953

C -6.461579 -1.205795 0.484895

C -5.854972 -1.320670 -0.760037

C -4.581407 -0.810090 -0.970928

O -0.501527 2.706115 -1.099974

C -0.675645 4.097554 -0.763012

C -2.115312 4.384740 -0.405266

O 2.954398 -3.221216 -0.584346

C 4.241765 -3.620913 -0.160082

H 0.139860 -3.598045 0.008858

H 0.177869 -2.486837 1.388338

H 1.015349 -1.806221 -1.469166

H 0.977754 -0.139603 1.086179

H -0.217970 0.408734 -1.656644

H 2.627559 -2.085688 1.101533

H 3.795602 -0.971677 -1.421592

H 2.161781 0.743041 -1.552219

H 3.970296 1.132245 2.637714

H 4.486351 2.676982 1.923619

H 5.642092 1.342025 2.066035

H 6.073727 1.427255 -0.474385

H 4.689871 1.309591 -1.578218

H 4.927172 2.775191 -0.610515

H -3.990632 0.462343 2.114175

H -6.254670 -0.469948 2.492400

H -7.454407 -1.605775 0.645961

H -6.374050 -1.811356 -1.574084

H -4.104013 -0.893333 -1.940114

H 0.002329 4.349080 0.051504

H -0.373105 4.635322 -1.659072

H -2.774821 4.116820 -1.231622

H -2.415658 3.826719 0.481621

H -2.232499 5.449992 -0.200947

H 5.003840 -2.891428 -0.453094

H 4.273455 -3.744868 0.927949

H 4.461561 -4.574362 -0.637409

==============================

conf060_E1.16

C 0.067522 -2.551505 0.305237

C 1.103634 -1.699563 -0.381390

C 0.977139 -0.227208 -0.005309

C -0.284383 0.414533 -0.564339

C -1.584794 -0.302464 -0.226300

S -1.634772 -2.073453 -0.130958

C 2.544263 -2.017583 0.007027

C 3.320424 -0.776711 -0.455715

C 2.284946 0.372871 -0.529899

O 2.811004 1.383280 0.308532

O 4.262938 -0.322466 0.497788

C 4.190547 1.096972 0.513098

C 4.598458 1.594421 1.876213

C 5.024491 1.688788 -0.611235

N -2.636611 0.396203 -0.139265

C -0.396808 1.852956 -0.085841

O -0.367595 2.172989 1.073074

C -3.906521 -0.176738 0.071131

C -4.518219 -0.045992 1.316259

C -5.787586 -0.568293 1.520082

C -6.461374 -1.206058 0.484782

C -5.855096 -1.320124 -0.760378

C -4.581585 -0.809398 -0.971268

O -0.501642 2.706189 -1.099849

C -0.676301 4.097561 -0.762881

C -2.116038 4.384142 -0.404924

O 2.954419 -3.221151 -0.584594

C 4.241714 -3.620991 -0.160256

H 0.139901 -3.597965 0.008829

H 0.178059 -2.486857 1.388385

H 1.015335 -1.806124 -1.469150

H 0.978009 -0.139512 1.086207

H -0.217881 0.408858 -1.656544

H 2.627598 -2.085900 1.101470

H 3.796064 -0.971615 -1.421248

H 2.161801 0.742668 -1.552497

H 3.970012 1.133105 2.637578

H 4.486170 2.677588 1.923013

H 5.641894 1.342685 2.066070

H 6.073954 1.426535 -0.474391

H 4.690155 1.309016 -1.578326

H 4.927821 2.774774 -0.610980

H -3.989970 0.460952 2.114538

H -6.253936 -0.471525 2.492712

H -7.454160 -1.606134 0.645866

H -6.374370 -1.810288 -1.574614

H -4.104426 -0.892014 -1.940622

H 0.001703 4.349413 0.051509

H -0.374132 4.635433 -1.659005

H -2.233657 5.449351 -0.200630

H -2.775558 4.115906 -1.231170

H -2.416013 3.826031 0.482032

H 5.003853 -2.891448 -0.452969

H 4.273251 -3.745235 0.927746

H 4.461565 -4.574315 -0.637807

==============================

conf061_E1.17

C -0.179915 -2.513233 0.246667

C 0.887460 -1.745805 -0.489974

C 0.867783 -0.263075 -0.140769

C -0.362281 0.459103 -0.671318

C -1.695794 -0.161071 -0.280106

S -1.860390 -1.922087 -0.134811

C 2.317196 -2.146563 -0.138494

C 3.156205 -0.968584 -0.666520

C 2.197419 0.240912 -0.723463

O 2.851710 1.256052 0.002920

O 4.220885 -0.544057 0.161394

C 3.877686 0.680581 0.804549

C 3.393759 0.426306 2.223302

C 5.077275 1.599632 0.774479

N -2.698833 0.605027 -0.187891

C -0.335742 1.904972 -0.201066

O -0.304448 2.226516 0.957732

C -3.994445 0.116833 0.075288

C -4.542380 0.275513 1.346572

C -5.833992 -0.163279 1.601238

C -6.591583 -0.746498 0.592113

C -6.047233 -0.889635 -0.678427

C -4.753085 -0.461371 -0.940749

O -0.318470 2.758352 -1.219644

C -0.234175 4.161716 -0.894799

C 1.190813 4.564276 -0.595524

O 2.631444 -3.386268 -0.715572

C 3.907957 -3.854820 -0.330907

H -0.193056 -3.565995 -0.034926

H -0.024981 -2.442023 1.324055

H 0.758379 -1.867502 -1.572155

H 0.892242 -0.158879 0.947453

H -0.331735 0.438745 -1.764949

H 2.432504 -2.202293 0.953778

H 3.561541 -1.230124 -1.646182

H 2.047590 0.598781 -1.744436

H 4.210158 0.015317 2.818509

H 2.564384 -0.278912 2.241698

H 3.067783 1.364734 2.673819

H 5.900276 1.154659 1.333959

H 5.387715 1.761948 -0.257375

H 4.822421 2.557168 1.229690

H -3.948075 0.738613 2.124801

H -6.250979 -0.044659 2.593691

H -7.600909 -1.081814 0.793249

H -6.631583 -1.338042 -1.472382

H -4.323607 -0.567508 -1.929896

H -0.613117 4.667978 -1.780072

H -0.896644 4.366290 -0.054772

H 1.230249 5.639007 -0.411806

H 1.565846 4.045465 0.285986

H 1.838717 4.335204 -1.442694

H 3.979600 -3.943649 0.758804

H 4.044581 -4.836545 -0.781312

H 4.702260 -3.187976 -0.681540

==============================

conf062_E1.19

C 0.179496 -2.513460 -0.245647

C -0.887930 -1.745628 0.490495

C -0.867931 -0.262977 0.140924

C 0.362142 0.459078 0.671622

C 1.695608 -0.161355 0.280548

S 1.859986 -1.922442 0.135963

C -2.317637 -2.146249 0.138746

C -3.156580 -0.968059 0.666367

C -2.197629 0.241325 0.723223

O -2.851673 1.256385 -0.003482

O -4.221041 -0.543564 -0.161846

C -3.877503 0.680854 -0.805238

C -3.393224 0.426113 -2.223786

C -5.076992 1.600065 -0.775745

N 2.698673 0.604642 0.187806

C 0.336012 1.904932 0.201325

O 0.304328 2.226467 -0.957459

C 3.994179 0.116301 -0.075626

C 4.753053 -0.461914 0.940225

C 6.047115 -0.890235 0.677574

C 6.591146 -0.747151 -0.593115

C 5.833306 -0.163960 -1.602072

C 4.541785 0.274903 -1.347075

O 0.319651 2.758346 1.219901

C 0.236086 4.161752 0.895066

C -1.188841 4.565183 0.596665

O -2.632177 -3.385826 0.715932

C -3.908641 -3.854303 0.331011

H 0.192422 -3.566119 0.036340

H 0.024797 -2.442611 -1.323091

H -0.759162 -1.867060 1.572742

H -0.892105 -0.159031 -0.947326

H 0.331449 0.438757 1.765247

H -2.432677 -2.202115 -0.953548

H -3.562134 -1.229307 1.646017

H -2.047935 0.599379 1.744155

H -2.563860 -0.279141 -2.241771

H -3.067110 1.364393 -2.674511

H -4.209499 0.014975 -2.819059

H -5.899884 1.155036 -1.335343

H -5.387708 1.762665 0.255981

H -4.821910 2.557458 -1.231122

H 4.323818 -0.568004 1.929485

H 6.631645 -1.338672 1.471380

H 7.600407 -1.082516 -0.794494

H 6.250051 -0.045406 -2.594637

H 3.947297 0.738022 -2.125154

H 0.615910 4.667793 1.780085

H 0.898152 4.365874 0.054613

H -1.564719 4.046553 -0.284601

H -1.836357 4.336550 1.444251

H -1.227769 5.639922 0.412884

H -4.702970 -3.187350 0.681373

H -3.980023 -3.943255 -0.758708

H -4.045460 -4.835966 0.781494

==============================

conf063_E1.21

C 0.179757 -2.513239 -0.246397

C -0.887631 -1.745723 0.490128

C -0.867841 -0.262988 0.140864

C 0.362242 0.459149 0.671499

C 1.695729 -0.161121 0.280324

S 1.860253 -1.922154 0.135064

C -2.317355 -2.146452 0.138561

C -3.156342 -0.968403 0.666438

C -2.197517 0.241075 0.723383

O -2.851712 1.256169 -0.003163

O -4.220959 -0.543884 -0.161578

C -3.877556 0.680609 -0.804908

C -3.393356 0.426079 -2.223520

C -5.077106 1.599724 -0.775231

N 2.698772 0.604939 0.187906

C 0.335803 1.905005 0.201238

O 0.304340 2.226583 -0.957540

C 3.994306 0.116636 -0.075419

C 4.542458 0.276103 -1.346497

C 5.834009 -0.162800 -1.601282

C 6.591326 -0.746881 -0.592458

C 6.046766 -0.890782 0.677905

C 4.752673 -0.462431 0.940335

O 0.318844 2.758363 1.219864

C 0.234902 4.161758 0.895075

C -1.190039 4.564770 0.596216

O -2.631699 -3.386105 0.715713

C -3.908166 -3.854691 0.330944

H 0.192833 -3.565977 0.035289

H 0.024851 -2.442114 -1.323797

H -0.758631 -1.867381 1.572319

H -0.892143 -0.158861 -0.947366

H 0.331648 0.438721 1.765130

H -2.432563 -2.202272 -0.953714

H -3.561780 -1.229856 1.646082

H -2.047791 0.599044 1.744343

H -2.564090 -0.279282 -2.241719

H -3.067132 1.364406 -2.674066

H -4.209711 0.015190 -2.818858

H -5.900019 1.154727 -1.334821

H -5.387757 1.762214 0.256529

H -4.822119 2.557180 -1.230535

H 3.948383 0.739905 -2.124484

H 6.251159 -0.043573 -2.593592

H 7.600604 -1.082280 -0.793689

H 6.630911 -1.339855 1.471632

H 4.323039 -0.569139 1.929353

H 0.614279 4.667873 1.780249

H 0.897194 4.366150 0.054858

H -1.229180 5.639475 0.412276

H -1.565533 4.045915 -0.285074

H -1.837763 4.336094 1.443628

H -3.979641 -3.943727 -0.758760

H -4.044873 -4.836328 0.781519

H -4.702508 -3.187769 0.681335

==============================

conf064_E1.22

C -0.039924 -1.201386 -1.854300

C -0.854784 -1.281135 -0.586220

C -1.065586 0.100583 0.022348

C 0.224897 0.844980 0.370157

C 1.491963 0.038625 0.134844

S 1.690525 -0.758174 -1.448925

C -2.286489 -1.786616 -0.703506

C -2.886722 -1.386012 0.661266

C -2.043057 -0.190386 1.163681

O -2.984444 0.825015 1.437120

O -4.216259 -0.908762 0.645611

C -4.228263 0.505175 0.822348

C -4.383413 1.211886 -0.515236

C -5.341202 0.865010 1.780293

N 2.381581 0.019828 1.033011

C 0.363618 2.143552 -0.431490

O -0.381480 2.494554 -1.308585

C 3.623643 -0.615856 0.842427

C 4.548671 -0.113690 -0.071443

C 5.789736 -0.721307 -0.204155

C 6.114042 -1.831469 0.565819

C 5.191738 -2.325428 1.481568

C 3.954754 -1.715459 1.631797

O 1.426317 2.832996 -0.036291

C 1.685395 4.086745 -0.702910

C 0.836922 5.193757 -0.123590

O -2.302650 -3.166614 -0.955219

C -3.610373 -3.668414 -1.141329

H 0.032657 -2.163439 -2.360433

H -0.459062 -0.467131 -2.543107

H -0.342010 -1.932098 0.133518

H -1.596752 0.694260 -0.722604

H 0.243950 1.130476 1.423957

H -2.816811 -1.251963 -1.504383

H -2.835576 -2.247315 1.330895

H -1.506608 -0.415689 2.086935

H -3.617704 0.898235 -1.223001

H -4.310861 2.290734 -0.372005

H -5.361162 0.978897 -0.938891

H -5.349270 1.941573 1.953111

H -6.302835 0.571482 1.358962

H -5.189220 0.348703 2.727821

H 4.292545 0.757571 -0.663533

H 6.504838 -0.324322 -0.913991

H 7.081354 -2.305097 0.458108

H 5.438278 -3.187905 2.088322

H 3.235344 -2.085738 2.351769

H 2.744701 4.268145 -0.533981

H 1.509987 3.957900 -1.770103

H 1.091962 6.135383 -0.611901

H -0.223518 4.999056 -0.282564

H 1.023010 5.298789 0.945818

H -4.214006 -3.559863 -0.234490

H -4.115404 -3.149022 -1.963328

H -3.522557 -4.726070 -1.384483

==============================

conf065_E1.25

C 0.179796 -2.513147 -0.246752

C -0.887588 -1.745716 0.489871

C -0.867825 -0.262968 0.140722

C 0.362288 0.459073 0.671344

C 1.695783 -0.161198 0.280181

S 1.860275 -1.922234 0.135035

C -2.317328 -2.146400 0.138347

C -3.156260 -0.968430 0.666518

C -2.197463 0.241068 0.723379

O -2.851732 1.256157 -0.003133

O -4.221089 -0.543866 -0.161167

C -3.877898 0.680666 -0.804526

C -3.394184 0.426226 -2.223315

C -5.077433 1.599788 -0.774355

N 2.698794 0.604917 0.187772

C 0.335950 1.904940 0.201137

O 0.304570 2.226521 -0.957645

C 3.994394 0.116673 -0.075390

C 4.542818 0.276508 -1.346317

C 5.834400 -0.162365 -1.600974

C 6.591489 -0.746784 -0.592164

C 6.046657 -0.891080 0.678033

C 4.752517 -0.462763 0.940330

O 0.318934 2.758301 1.219756

C 0.234978 4.161682 0.894941

C -1.189949 4.564604 0.595891

O -2.631620 -3.386152 0.715328

C -3.908135 -3.854659 0.330622

H 0.192773 -3.565957 0.034681

H 0.024998 -2.441762 -1.324150

H -0.758550 -1.867451 1.572054

H -0.892271 -0.158735 -0.947498

H 0.331681 0.438658 1.764977

H -2.432635 -2.202047 -0.953928

H -3.561426 -1.230032 1.646237

H -2.047667 0.599042 1.744323

H -2.564696 -0.278860 -2.241746

H -3.068408 1.364636 -2.674014

H -4.210617 0.015053 -2.818342

H -5.900513 1.154868 -1.333762

H -5.387773 1.762128 0.257524

H -4.822572 2.557308 -1.229593

H 3.948911 0.740575 -2.124272

H 6.251777 -0.042854 -2.593155

H 7.600794 -1.082162 -0.793302

H 6.630608 -1.340441 1.471741

H 4.322665 -0.569805 1.929216

H 0.614217 4.667832 1.780155

H 0.897364 4.366104 0.054808

H -1.229097 5.639264 0.411706

H -1.565353 4.045557 -0.285330

H -1.837736 4.336087 1.443297

H -4.044864 -4.836315 0.781148

H -4.702423 -3.187717 0.681097

H -3.979690 -3.943632 -0.759084

==============================

conf066_E1.28

C 1.426891 -2.832569 0.654857

C 1.715618 -1.706031 -0.304650

C 0.883132 -0.473483 0.021764

C -0.607068 -0.621893 -0.292439

C -1.263109 -1.870346 0.292977

S -0.318392 -3.333677 0.612156

C 3.144423 -1.173113 -0.233065

C 3.052409 0.211820 -0.897902

C 1.575989 0.644537 -0.772563

O 1.619276 1.904909 -0.142671

O 3.784819 1.249014 -0.275095

C 2.898838 2.096177 0.451115

C 2.882323 1.725951 1.925952

C 3.312274 3.534023 0.236005

N -2.503023 -2.040650 0.493018

C -1.278663 0.644634 0.220050

O -1.479420 0.860760 1.387164

C -3.499397 -1.128466 0.099506

C -3.673230 -0.772994 -1.237150

C -4.711296 0.075041 -1.602714

C -5.579334 0.577764 -0.643281

C -5.413159 0.212482 0.689929

C -4.390194 -0.645382 1.059819

O -1.543706 1.494293 -0.763101

C -2.187110 2.733436 -0.400699

C -1.182003 3.764561 0.055231

O 4.024368 -2.062647 -0.868342

C 5.377351 -1.680109 -0.727399

H 1.980770 -3.736731 0.401845

H 1.687607 -2.536606 1.672072

H 1.519608 -2.023336 -1.335470

H 0.990116 -0.263013 1.090101

H -0.724565 -0.679346 -1.377681

H 3.440554 -1.042281 0.817805

H 3.380058 0.132422 -1.936461

H 1.097739 0.767980 -1.745909

H 3.865857 1.910438 2.359926

H 2.632369 0.676191 2.072159

H 2.146447 2.338682 2.448721

H 3.306349 3.760502 -0.829892

H 2.617638 4.199236 0.750088

H 4.313466 3.697522 0.635136

H -2.997070 -1.168135 -1.985870

H -4.837493 0.343267 -2.644503

H -6.384801 1.241640 -0.930097

H -6.089148 0.594087 1.445135

H -4.258526 -0.940003 2.093680

H -2.696512 3.048711 -1.309390

H -2.929175 2.523825 0.369660

H -1.703819 4.697160 0.276246

H -0.661162 3.431540 0.952193

H -0.445046 3.953091 -0.725416

H 5.984576 -2.446914 -1.205588

H 5.574316 -0.717293 -1.209851

H 5.654454 -1.606122 0.330035

==============================

conf067_E1.30

C 1.426837 -2.832455 0.655137

C 1.715544 -1.706055 -0.304544

C 0.883072 -0.473460 0.021793

C -0.607134 -0.621882 -0.292295

C -1.263176 -1.870328 0.293130

S -0.318402 -3.333628 0.612493

C 3.144352 -1.173126 -0.233093

C 3.052291 0.211747 -0.898040

C 1.575902 0.644523 -0.772596

O 1.619282 1.904893 -0.142701

O 3.784796 1.248998 -0.275449

C 2.898943 2.096128 0.450935

C 2.882604 1.725901 1.925768

C 3.312396 3.533968 0.235800

N -2.503091 -2.040699 0.493036

C -1.278681 0.644629 0.220284

O -1.479327 0.860690 1.387443

C -3.499423 -1.128444 0.099419

C -4.390110 -0.645089 1.059641

C -5.413027 0.212800 0.689559

C -5.579183 0.577804 -0.643702

C -4.711193 0.074805 -1.603068

C -3.673231 -0.773246 -1.237332

O -1.543850 1.494319 -0.762795

C -2.187174 2.733494 -0.400256

C -1.181956 3.764555 0.055559

O 4.024245 -2.062708 -0.868404

C 5.377238 -1.680132 -0.727697

H 1.980783 -3.736624 0.402281

H 1.687561 -2.536312 1.672301

H 1.519475 -2.023477 -1.335313

H 0.990111 -0.262927 1.090112

H -0.724758 -0.679321 -1.377530

H 3.440589 -1.042261 0.817741

H 3.379816 0.132216 -1.936630

H 1.097591 0.767990 -1.745912

H 3.866161 1.910490 2.359650

H 2.632759 0.676128 2.072051

H 2.146722 2.338586 2.448576

H 2.617975 4.199204 0.750131

H 4.313722 3.697339 0.634648

H 3.306194 3.760510 -0.830080

H -4.258469 -0.939428 2.093582

H -6.088972 0.594631 1.444690

H -6.384615 1.241673 -0.930636

H -4.837387 0.342843 -2.644904

H -2.997083 -1.168644 -1.985927

H -2.696694 3.048783 -1.308872

H -2.929109 2.523886 0.370230

H -1.703716 4.697155 0.276688

H -0.661028 3.431466 0.952445

H -0.445088 3.953086 -0.725165

H 5.984419 -2.447135 -1.205623

H 5.574186 -0.717504 -1.210526

H 5.654418 -1.605740 0.329690

==============================

conf068_E1.37

C -0.205438 -2.587455 0.178055

C 0.881568 -1.759906 -0.458725

C 0.861770 -0.316934 0.030850

C -0.356081 0.460334 -0.449591

C -1.703024 -0.189397 -0.171656

S -1.874932 -1.954378 -0.179494

C 2.299614 -2.204845 -0.108859

C 3.160107 -0.983497 -0.464542

C 2.201235 0.231032 -0.467948

O 2.788815 1.159988 0.421886

O 4.105555 -0.659811 0.538655

C 4.138530 0.757145 0.638510

C 4.547418 1.140694 2.037582

C 5.041084 1.350989 -0.430175

N -2.711444 0.568265 -0.068147

C -0.333255 1.848770 0.168612

O -0.382109 2.055583 1.352512

C -4.016482 0.059445 0.088578

C -4.611983 0.065159 1.348361

C -5.913302 -0.392823 1.498825

C -6.632746 -0.844752 0.398534

C -6.039999 -0.837043 -0.858383

C -4.736360 -0.387376 -1.017948

O -0.201057 2.800220 -0.748906

C -0.099420 4.151344 -0.257016

C 0.084327 5.055749 -1.447528

O 2.628225 -3.376738 -0.805290

C 3.881141 -3.902766 -0.417074

H -0.215551 -3.608240 -0.203624

H -0.073095 -2.620517 1.260107

H 0.778661 -1.777639 -1.550014

H 0.882594 -0.318365 1.126052

H -0.292782 0.555644 -1.537817

H 2.377661 -2.374049 0.974925

H 3.645648 -1.135652 -1.432657

H 2.097555 0.663784 -1.467481

H 4.516156 2.224641 2.147758

H 5.563644 0.798613 2.233662

H 3.866516 0.684969 2.756043

H 5.024625 2.439141 -0.364570

H 6.064668 1.002893 -0.288708

H 4.703552 1.056541 -1.425491

H -4.046553 0.425547 2.199104

H -6.367747 -0.392826 2.481891

H -7.649473 -1.195951 0.519209

H -6.594008 -1.182927 -1.722328

H -4.268980 -0.376013 -1.995450

H -1.009035 4.386394 0.296867

H 0.746347 4.204205 0.429564

H -0.765782 4.980907 -2.126343

H 0.164849 6.088541 -1.107255

H 0.994008 4.799266 -1.991581

H 4.694467 -3.204010 -0.638560

H 3.894420 -4.129134 0.654728

H 4.039003 -4.820707 -0.980723

==============================

conf069_E1.37

C -0.205955 -2.587319 0.179046

C 0.880923 -1.760058 -0.458357

C 0.861536 -0.317015 0.030983

C -0.356200 0.460463 -0.449408

C -1.703264 -0.189011 -0.171466

S -1.875518 -1.953899 -0.177590

C 2.298991 -2.205225 -0.108868

C 3.159659 -0.984052 -0.464782

C 2.201016 0.230639 -0.468103

O 2.788913 1.159576 0.421543

O 4.105308 -0.660494 0.538269

C 4.138543 0.756461 0.638107

C 4.547559 1.139956 2.037157

C 5.041186 1.350073 -0.430629

N -2.711670 0.568849 -0.069072

C -0.333020 1.848857 0.168883

O -0.382590 2.055630 1.352766

C -4.016769 0.060179 0.087722

C -4.736679 -0.386671 -1.018780

C -6.040348 -0.836227 -0.859184

C -6.633100 -0.843797 0.397741

C -5.913619 -0.391862 1.498001

C -4.612246 0.065985 1.347507

O -0.199521 2.800256 -0.748483

C -0.097184 4.151281 -0.256454

C 0.088072 5.055593 -1.446799

O 2.627207 -3.377205 -0.805334

C 3.880093 -3.903492 -0.417361

H -0.216506 -3.608120 -0.202572

H -0.073014 -2.620321 1.261023

H 0.777599 -1.777998 -1.549599

H 0.882526 -0.318286 1.126185

H -0.292896 0.555826 -1.537620

H 2.377289 -2.374383 0.974905

H 3.645039 -1.136365 -1.432948

H 2.097234 0.663325 -1.467649

H 3.866684 0.684239 2.755652

H 4.516322 2.223906 2.147348

H 5.563791 0.797859 2.233170

H 4.703522 1.055654 -1.425914

H 5.025012 2.438235 -0.365076

H 6.064685 1.001715 -0.289193

H -4.269265 -0.375429 -1.996267

H -6.594389 -1.182139 -1.723099

H -7.649857 -1.194905 0.518437

H -6.368059 -0.391769 2.481070

H -4.046769 0.426348 2.198231

H -1.006997 4.386995 0.296816

H 0.748153 4.203452 0.430704

H -0.761646 4.981462 -2.126184

H 0.169189 6.088305 -1.106420

H 0.997913 4.798414 -1.990262

H 4.037603 -4.821537 -0.980939

H 4.693538 -3.204959 -0.639126

H 3.893581 -4.129736 0.654464

==============================

conf070_E1.46

C -0.330303 -2.540986 0.297208

C 0.783421 -1.790897 -0.387478

C 0.797620 -0.315624 -0.004339

C -0.391424 0.451980 -0.565758

C -1.755942 -0.131515 -0.233158

S -1.979288 -1.887811 -0.117342

C 2.186573 -2.247002 0.002832

C 3.080194 -1.082738 -0.447369

C 2.160357 0.161216 -0.513472

O 2.771748 1.104663 0.344458

O 4.059914 -0.729553 0.511769

C 4.118337 0.690438 0.547264

C 4.565256 1.129652 1.918048

C 5.006257 1.219312 -0.567063

N -2.740168 0.661502 -0.172996

C -0.332797 1.892077 -0.082384

O -0.352711 2.206709 1.078182

C -4.057014 0.203996 0.035632

C -4.630393 0.311181 1.300972

C -5.941220 -0.097686 1.501596

C -6.691620 -0.601152 0.445171

C -6.120731 -0.693972 -0.818346

C -4.807946 -0.293844 -1.027611

O -0.218441 2.748267 -1.093011

C -0.078439 4.143771 -0.754614

C 1.350016 4.469107 -0.385684

O 2.482747 -3.479899 -0.596059

C 3.724872 -4.002897 -0.170739

H -0.367426 -3.585523 -0.011535

H -0.205959 -2.501340 1.380018

H 0.686342 -1.884737 -1.475523

H 0.798253 -0.235400 1.087909

H -0.321261 0.441590 -1.657562

H 2.258417 -2.330037 1.097154

H 3.537506 -1.314170 -1.413591

H 2.082960 0.554961 -1.531562

H 4.555931 2.218035 1.978446

H 5.579210 0.777630 2.108106

H 3.892275 0.720475 2.671120

H 4.643561 0.883202 -1.540155

H 5.006879 2.309661 -0.552817

H 6.027513 0.862481 -0.430764

H -4.040549 0.710689 2.116972

H -6.378659 -0.019007 2.489192

H -7.715479 -0.914056 0.604626

H -6.699025 -1.080439 -1.648528

H -4.357183 -0.361199 -2.010632

H -0.387965 4.673408 -1.653165

H -0.768459 4.378546 0.054677

H 2.023797 4.213644 -1.204848

H 1.436884 5.538579 -0.188123

H 1.656259 3.922865 0.505854

H 3.740237 -4.137922 0.916359

H 3.855519 -4.969062 -0.655153

H 4.553684 -3.346408 -0.454997

==============================

conf071_E1.49

C 0.329879 -2.541149 -0.296971

C -0.783783 -1.790847 0.387582

C -0.797815 -0.315614 0.004309

C 0.391346 0.451882 0.565642

C 1.755792 -0.131861 0.233147

S 1.978924 -1.888189 0.117676

C -2.186972 -2.246849 -0.002724

C -3.080461 -1.082454 0.447381

C -2.160485 0.161405 0.513450

O -2.771837 1.104934 -0.344426

O -4.060105 -0.729204 -0.511811

C -4.118466 0.690803 -0.547204

C -4.565439 1.130130 -1.917931

C -5.006325 1.219666 0.567179

N 2.740105 0.661046 0.172879

C 0.333042 1.891954 0.082154

O 0.352692 2.206491 -1.078444

C 4.056911 0.203533 -0.035720

C 4.629957 0.309713 -1.301321

C 5.940792 -0.099071 -1.501947

C 6.691592 -0.601476 -0.445279

C 6.121044 -0.693334 0.818452

C 4.808234 -0.293264 1.027727

O 0.219401 2.748275 1.092752

C 0.080045 4.143833 0.754326

C -1.348325 4.469977 0.385785

O -2.483299 -3.479664 0.596284

C -3.725374 -4.002650 0.170813

H 0.366801 -3.585669 0.011848

H 0.205632 -2.501547 -1.379794

H -0.686781 -1.884610 1.475641

H -0.798471 -0.235496 -1.087947

H 0.321161 0.441614 1.657447

H -2.258806 -2.330008 -1.097030

H -3.537837 -1.313777 1.413597

H -2.082990 0.555142 1.531535

H -5.579435 0.778199 -2.107931

H -3.892551 0.720954 -2.671087

H -4.556045 2.218517 -1.978262

H -6.027579 0.862811 0.430921

H -4.643599 0.883604 1.540273

H -5.006992 2.310015 0.552907

H 4.039809 0.708406 -2.117505

H 6.377944 -0.021172 -2.489733

H 7.715471 -0.914307 -0.604744

H 6.699613 -1.078991 1.648821

H 4.357787 -0.359820 2.010950

H 0.390107 4.673337 1.652771

H 0.769979 4.378208 -0.055157

H -1.434626 5.539496 0.188234

H -1.655125 3.923913 -0.505671

H -2.022028 4.214915 1.205137

H -4.554232 -3.346247 0.455146

H -3.740685 -4.137486 -0.916308

H -3.856008 -4.968908 0.655047

==============================

conf072_E1.51

C 0.330421 -2.540951 -0.297679

C -0.783284 -1.791042 0.387234

C -0.797582 -0.315714 0.004369

C 0.391524 0.451917 0.565653

C 1.756055 -0.131601 0.233098

S 1.979416 -1.887814 0.116931

C -2.186454 -2.247117 -0.003094

C -3.080076 -1.082941 0.447346

C -2.160236 0.160977 0.513834

O -2.771729 1.104811 -0.343617

O -4.059717 -0.729472 -0.511784

C -4.118287 0.690542 -0.546611

C -4.565270 1.130346 -1.917183

C -5.006274 1.218849 0.567936

N 2.740292 0.661459 0.173333

C 0.332757 1.891934 0.082077

O 0.352960 2.206426 -1.078525

C 4.057176 0.204178 -0.035247

C 4.808332 -0.293187 1.028062

C 6.121212 -0.693028 0.818827

C 6.692012 -0.600388 -0.444738

C 5.941392 -0.097421 -1.501258

C 4.630479 0.311144 -1.300663

O 0.217796 2.748221 1.092554

C 0.077231 4.143612 0.753926

C -1.351337 4.468202 0.384761

O -2.482619 -3.480121 0.595605

C -3.724629 -4.003161 0.170014

H 0.367578 -3.585551 0.010839

H 0.206033 -2.501058 -1.380474

H -0.686152 -1.885108 1.475254

H -0.798438 -0.235334 -1.087868

H 0.321378 0.441655 1.657457

H -2.258341 -2.330002 -1.097416

H -3.537482 -1.314609 1.413465

H -2.082673 0.554327 1.532060

H -5.579200 0.778329 -2.107372

H -3.892280 0.721578 -2.670469

H -4.556039 2.218757 -1.977072

H -6.027475 0.861913 0.431488

H -4.643556 0.882460 1.540919

H -5.007078 2.309203 0.554089

H 4.357683 -0.360359 2.011151

H 6.699641 -1.079126 1.649088

H 7.715950 -0.913039 -0.604172

H 6.378740 -0.018901 -2.488908

H 4.040480 0.710279 -2.116737

H 0.386412 4.673524 1.652434

H 0.767242 4.378582 -0.055315

H -1.438605 5.537496 0.186431

H -1.657346 3.921175 -0.506381

H -2.025046 4.213037 1.204075

H -3.739999 -4.137543 -0.917163

H -3.855050 -4.969647 0.653850

H -4.553586 -3.347028 0.454683

==============================

conf073_E1.52

C -0.330165 -2.541080 0.296928

C 0.783548 -1.790866 -0.387639

C 0.797706 -0.315631 -0.004380

C -0.391397 0.451988 -0.565674

C -1.755886 -0.131639 -0.233192

S -1.979168 -1.887936 -0.117600

C 2.186707 -2.246961 0.002680

C 3.080284 -1.082639 -0.447448

C 2.160396 0.161276 -0.513567

O 2.771813 1.104822 0.344239

O 4.059932 -0.729423 0.511749

C 4.118401 0.690580 0.547083

C 4.565359 1.129926 1.917808

C 5.006341 1.219329 -0.567291

N -2.740170 0.661310 -0.173076

C -0.332900 1.892012 -0.082076

O -0.352718 2.206457 1.078543

C -4.056974 0.203809 0.035585

C -4.808653 -0.292063 -1.028037

C -6.121455 -0.692134 -0.818697

C -6.691638 -0.601176 0.445263

C -5.940468 -0.099708 1.502115

C -4.629630 0.309042 1.301441

O -0.218820 2.748384 -1.092580

C -0.078983 4.143854 -0.753986

C 1.349464 4.469379 -0.385192

O 2.482953 -3.479814 -0.596286

C 3.724985 -4.002875 -0.170783

H -0.367203 -3.585585 -0.011929

H -0.205867 -2.501532 1.379747

H 0.686536 -1.884632 -1.475697

H 0.798406 -0.235508 1.087879

H -0.321216 0.441773 -1.657479

H 2.258525 -2.330089 1.096989

H 3.537658 -1.314026 -1.413651

H 2.082903 0.554960 -1.531672

H 5.579318 0.777919 2.107862

H 3.892407 0.720831 2.670950

H 4.556050 2.218315 1.978097

H 5.007078 2.309679 -0.553074

H 6.027566 0.862414 -0.430976

H 4.643628 0.883242 -1.540382

H -4.358481 -0.357919 -2.011433

H -6.700305 -1.077082 -1.649199

H -7.715515 -0.913992 0.604772

H -6.377324 -0.022534 2.490089

H -4.039176 0.706981 2.117775

H -0.388702 4.673576 -1.652422

H -0.768947 4.378411 0.055419

H 1.655947 3.922979 0.506167

H 2.023174 4.214240 -1.204515

H 1.436146 5.538820 -0.187386

H 3.855554 -4.969159 -0.654981

H 4.553895 -3.346543 -0.455128

H 3.740274 -4.137675 0.916344

==============================

conf074_E1.53

C -0.008120 -2.339383 0.716592

C 1.031133 -1.774074 -0.217730

C 0.934486 -0.256989 -0.323872

C -0.316841 0.198439 -1.063143

C -1.623090 -0.317360 -0.480653

S -1.712747 -1.955453 0.197426

C 2.473276 -1.982205 0.234918

C 3.261929 -0.970553 -0.616669

C 2.252164 0.129029 -1.011974

O 2.841674 1.336458 -0.582081

O 4.304024 -0.280877 0.043288

C 3.897005 1.053442 0.332038

C 3.425960 1.173877 1.772515

C 5.048462 1.986963 0.036379

N -2.655805 0.402743 -0.607775

C -0.358354 1.712753 -1.164588

O -0.342403 2.320570 -2.203975

C -3.927648 -0.028524 -0.179867

C -4.463428 0.481623 1.000870

C -5.732334 0.093857 1.407156

C -6.479638 -0.788875 0.635900

C -5.947986 -1.283882 -0.548688

C -4.676359 -0.907943 -0.959590

O -0.368776 2.286106 0.032411

C -0.386739 3.724944 0.057020

C -0.452191 4.154811 1.499411

O 2.853908 -3.319565 0.046733

C 4.147437 -3.591458 0.546493

H 0.024703 -3.428185 0.752687

H 0.137893 -1.952098 1.725755

H 0.921585 -2.216603 -1.214849

H 0.926405 0.162856 0.685494

H -0.269265 -0.183367 -2.087460

H 2.575334 -1.709679 1.295396

H 3.681866 -1.485801 -1.483031

H 2.110438 0.187586 -2.093130

H 3.078402 2.190317 1.961077

H 4.255975 0.956595 2.445975

H 2.615082 0.479390 1.987695

H 4.744228 3.018973 0.212932

H 5.891347 1.754744 0.687620

H 5.353344 1.873592 -1.003624

H -3.877180 1.174906 1.591818

H -6.139977 0.487393 2.330089

H -7.471356 -1.084528 0.953266

H -6.524840 -1.967855 -1.158883

H -4.256935 -1.288017 -1.883535

H 0.515481 4.089538 -0.436172

H -1.253059 4.070106 -0.508655

H -1.356495 3.774191 1.975639

H 0.415733 3.793934 2.052120

H -0.466389 5.243877 1.551633

H 4.333694 -4.655195 0.407729

H 4.912800 -3.022392 0.009017

H 4.213618 -3.348728 1.612896

==============================

conf075_E1.54

C -0.221162 -1.396139 -1.724846

C -1.025857 -1.301366 -0.451648

C -1.137451 0.144209 0.018841

C 0.198034 0.808494 0.357258

C 1.423608 -0.064664 0.120810

S 1.533281 -0.997896 -1.392859

C -2.488479 -1.717183 -0.541824

C -3.087875 -1.127251 0.751061

C -2.171576 0.054088 1.145177

O -3.041290 1.159660 1.266590

O -4.378301 -0.562665 0.639990

C -4.288737 0.859264 0.649706

C -4.354953 1.412075 -0.765651

C -5.396398 1.409016 1.519508

N 2.358183 -0.036804 0.973829

C 0.411376 2.092006 -0.453420

O -0.252056 2.425005 -1.424628

C 3.571102 -0.724422 0.776005

C 3.900530 -1.784167 1.618748

C 5.110799 -2.443375 1.458025

C 6.008628 -2.038451 0.476693

C 5.686930 -0.967384 -0.348023

C 4.472329 -0.310865 -0.203883

O 1.430483 2.797746 0.028932

C 1.766101 4.012682 -0.671721

C 2.950922 4.624811 0.028218

O -2.596811 -3.112486 -0.641800

C -3.930927 -3.541090 -0.823086

H -0.204901 -2.408058 -2.128467

H -0.614769 -0.716900 -2.481778

H -0.556974 -1.916267 0.326883

H -1.588798 0.705824 -0.800357

H 0.242133 1.092239 1.410184

H -2.965197 -1.241436 -1.410747

H -3.116472 -1.909335 1.512821

H -1.682393 -0.100815 2.108258

H -3.583611 0.979235 -1.401173

H -4.226313 2.495022 -0.743984

H -5.328899 1.182875 -1.200051

H -6.366065 1.140346 1.099940

H -5.310828 0.996521 2.524504

H -5.325594 2.495954 1.568210

H 3.200739 -2.085564 2.388435

H 5.355511 -3.274742 2.107440

H 6.955256 -2.550254 0.360142

H 6.383329 -0.639561 -1.109860

H 4.217845 0.529772 -0.839366

H 1.991291 3.760914 -1.708711

H 0.897307 4.671667 -0.656553

H 3.802900 3.944096 0.013476

H 3.235078 5.545437 -0.482397

H 2.708471 4.864090 1.063995

H -3.911230 -4.621419 -0.957188

H -4.550897 -3.300848 0.046665

H -4.374553 -3.074635 -1.709725

==============================

conf076_E1.57

C 0.213322 -2.499633 0.250576

C 1.198393 -1.632761 -0.490091

C 1.031852 -0.155850 -0.150860

C -0.265777 0.431238 -0.684940

C -1.528949 -0.318138 -0.281157

S -1.516722 -2.087747 -0.144893

C 2.661504 -1.887556 -0.142531

C 3.375115 -0.640052 -0.693185

C 2.303537 0.472264 -0.743555

O 2.865140 1.546762 -0.024427

O 4.408364 -0.107795 0.111083

C 3.952166 1.073834 0.763826

C 3.511274 0.767878 2.186620

C 5.054730 2.106929 0.725192

N -2.598579 0.348541 -0.165855

C -0.408493 1.875188 -0.231351

O -0.329958 2.221324 0.918085

C -3.838509 -0.259140 0.114383

C -4.390708 -0.130342 1.387208

C -5.631426 -0.688147 1.661146

C -6.336117 -1.360059 0.668819

C -5.789759 -1.472233 -0.603899

C -4.544998 -0.925601 -0.885142

O -0.602700 2.700672 -1.254423

C -0.821719 4.090340 -0.936458

C -2.251672 4.320899 -0.506167

O 3.092301 -3.098240 -0.706088

C 4.413738 -3.431166 -0.332377

H 0.310705 -3.550870 -0.020885

H 0.354103 -2.402490 1.327950

H 1.080121 -1.772531 -1.571476

H 1.049374 -0.039169 0.936185

H -0.237594 0.402750 -1.778513

H 2.787707 -1.917273 0.949377

H 3.786802 -0.871150 -1.677896

H 2.112122 0.812188 -1.763733

H 4.371161 0.439226 2.771695

H 2.757169 -0.017376 2.210928

H 3.097555 1.667436 2.644186

H 5.925400 1.741065 1.269626

H 5.332925 2.306009 -0.309412

H 4.713898 3.031483 1.191862

H -3.838697 0.402476 2.151910

H -6.050758 -0.592856 2.655098

H -7.306409 -1.788491 0.884743

H -6.333682 -1.988938 -1.384984

H -4.115351 -1.006242 -1.876620

H -0.112943 4.390323 -0.165747

H -0.592724 4.620458 -1.858494

H -2.406424 5.384257 -0.317579

H -2.941721 4.004942 -1.289488

H -2.477969 3.770569 0.407145

H 5.133591 -2.696801 -0.707774

H 4.508866 -3.490367 0.757499

H 4.640180 -4.403613 -0.766499

==============================

conf077_E1.58

C -0.006224 -2.549844 0.072936

C 1.022210 -1.670494 -0.590195

C 0.939730 -0.228718 -0.105066

C -0.324548 0.483928 -0.562920

C -1.627189 -0.229000 -0.229721

S -1.713084 -2.001246 -0.249510

C 2.469921 -2.037834 -0.277746

C 3.253168 -0.781410 -0.699679

C 2.241884 0.384597 -0.643325

O 2.856808 1.358823 0.168792

O 4.304809 -0.386698 0.158863

C 3.914326 0.759505 0.910600

C 3.454478 0.358877 2.303342

C 5.073496 1.728394 0.956800

N -2.662123 0.480178 -0.063349

C -0.361101 1.878330 0.041279

O -0.324883 2.094236 1.224235

C -3.933143 -0.088219 0.155541

C -4.671825 -0.602698 -0.908405

C -5.943678 -1.111882 -0.683958

C -6.485436 -1.112759 0.595747

C -5.748009 -0.592649 1.653230

C -4.479174 -0.073878 1.437465

O -0.396605 2.826426 -0.887959

C -0.394435 4.186808 -0.410914

C -0.423689 5.091398 -1.615008

O 2.834964 -3.206135 -0.963554

C 4.131373 -3.652924 -0.622058

H 0.025411 -3.571359 -0.305814

H 0.151123 -2.572304 1.152058

H 0.893200 -1.697006 -1.678791

H 0.967836 -0.225417 0.988090

H -0.299561 0.568352 -1.653639

H 2.592429 -2.185269 0.805084

H 3.661314 -0.935191 -1.700853

H 2.068766 0.825048 -1.627559

H 3.094457 1.238602 2.838293

H 4.292617 -0.069368 2.854498

H 2.655047 -0.379282 2.262575

H 5.919869 1.269925 1.468517

H 5.366774 1.996198 -0.057947

H 4.783621 2.629040 1.498680

H -4.244731 -0.596524 -1.904249

H -6.512759 -1.510150 -1.514909

H -7.477270 -1.510973 0.766991

H -6.163177 -0.586619 2.653485

H -3.900534 0.340336 2.254097

H -1.267227 4.329364 0.227288

H 0.502123 4.338756 0.191303

H -1.321900 4.917846 -2.208427

H -0.422781 6.130766 -1.285459

H 0.451983 4.927638 -2.243938

H 4.895720 -2.923794 -0.909549

H 4.210058 -3.837492 0.455059

H 4.308231 -4.582833 -1.159978

==============================

conf078_E1.62

C 0.213365 -2.499567 0.250626

C 1.198332 -1.632610 -0.490079

C 1.031838 -0.155720 -0.150705

C -0.265797 0.431363 -0.684780

C -1.528960 -0.318061 -0.281060

S -1.516738 -2.087611 -0.144512

C 2.661477 -1.887478 -0.142728

C 3.375041 -0.639955 -0.693372

C 2.303503 0.472434 -0.743414

O 2.865260 1.546763 -0.024156

O 4.408486 -0.107818 0.110711

C 3.952362 1.073616 0.763847

C 3.511626 0.767289 2.186615

C 5.054937 2.106701 0.725387

N -2.598630 0.348597 -0.165913

C -0.408589 1.875292 -0.231200

O -0.330071 2.221438 0.918235

C -3.838527 -0.259171 0.114318

C -4.545237 -0.925077 -0.885422

C -5.789956 -1.471819 -0.604218

C -6.336060 -1.360292 0.668673

C -5.631153 -0.688931 1.661208

C -4.390452 -0.131048 1.387324

O -0.602843 2.700764 -1.254277

C -0.821889 4.090422 -0.936321

C -2.251859 4.321011 -0.506093

O 3.092164 -3.098130 -0.706421

C 4.413693 -3.431048 -0.333032

H 0.310714 -3.550763 -0.021003

H 0.354306 -2.402559 1.327991

H 1.079907 -1.772263 -1.571463

H 1.049363 -0.039109 0.936347

H -0.237575 0.402878 -1.778355

H 2.787802 -1.917293 0.949163

H 3.786504 -0.870966 -1.678197

H 2.112009 0.812557 -1.763512

H 4.371544 0.438324 2.771469

H 2.757390 -0.017841 2.210809

H 3.098115 1.666774 2.644511

H 5.333054 2.306043 -0.309188

H 4.714153 3.031137 1.192323

H 5.925643 1.740682 1.269657

H -4.115792 -1.005198 -1.877028

H -6.334057 -1.988106 -1.385454

H -7.306331 -1.788798 0.884544

H -6.050281 -0.594139 2.655295

H -3.838253 0.401323 2.152202

H -0.113139 4.390414 -0.165589

H -0.592867 4.620545 -1.858347

H -2.941876 4.005041 -1.289437

H -2.478219 3.770725 0.407226

H -2.406590 5.384384 -0.317569

H 4.639924 -4.403642 -0.766938

H 5.133478 -2.696846 -0.708876

H 4.509166 -3.489937 0.756830

==============================

conf079_E1.62

C 0.213410 -2.499545 0.250786

C 1.198395 -1.632687 -0.490005

C 1.031887 -0.155771 -0.150770

C -0.265753 0.431295 -0.684813

C -1.528957 -0.318077 -0.281059

S -1.516689 -2.087682 -0.144465

C 2.661530 -1.887499 -0.142598

C 3.375109 -0.639998 -0.693301

C 2.303546 0.472348 -0.743526

O 2.865227 1.546789 -0.024365

O 4.408453 -0.107787 0.110860

C 3.952329 1.073793 0.763739

C 3.511579 0.767749 2.186563

C 5.054909 2.106858 0.725067

N -2.598617 0.348574 -0.165933

C -0.408555 1.875234 -0.231236

O -0.330067 2.221366 0.918204

C -3.838551 -0.259147 0.114306

C -4.544985 -0.925560 -0.885294

C -5.789724 -1.472279 -0.604124

C -6.336109 -1.360223 0.668602

C -5.631483 -0.688343 1.660988

C -4.390769 -0.130473 1.387129

O -0.602797 2.700706 -1.254313

C -0.822002 4.090340 -0.936358

C -2.252001 4.320711 -0.506102

O 3.092253 -3.098188 -0.706206

C 4.413741 -3.431101 -0.332670

H 0.310766 -3.550787 -0.020669

H 0.354324 -2.402384 1.328140

H 1.079998 -1.772458 -1.571377

H 1.049466 -0.039090 0.936270

H -0.237596 0.402814 -1.778385

H 2.787851 -1.917234 0.949294

H 3.786675 -0.871083 -1.678064

H 2.112070 0.812337 -1.763669

H 4.371506 0.438992 2.771523

H 2.757419 -0.017452 2.210904

H 3.097976 1.667302 2.644247

H 4.714183 3.031372 1.191895

H 5.925640 1.740894 1.269339

H 5.332973 2.306046 -0.309553

H -4.115300 -1.006084 -1.876767

H -6.333612 -1.988960 -1.385248

H -7.306391 -1.788709 0.884470

H -6.050852 -0.593139 2.654934

H -3.838799 0.402306 2.151887

H -0.113290 4.390431 -0.165633

H -0.593047 4.620479 -1.858388

H -2.941982 4.004622 -1.289432

H -2.478235 3.770363 0.407216

H -2.406923 5.384052 -0.317558

H 4.640007 -4.403725 -0.766491

H 5.133576 -2.696935 -0.708494

H 4.509112 -3.489922 0.757204

==============================

conf080_E1.62

C 0.213463 -2.499618 0.250366

C 1.198513 -1.632608 -0.490156

C 1.031925 -0.155738 -0.150781

C -0.265708 0.431293 -0.684894

C -1.528869 -0.318120 -0.281147

S -1.516591 -2.087765 -0.145109

C 2.661625 -1.887399 -0.142618

C 3.375198 -0.639843 -0.693197

C 2.303591 0.472460 -0.743441

O 2.865194 1.546904 -0.024237

O 4.408461 -0.107628 0.111071

C 3.952236 1.073917 0.763951

C 3.511345 0.767785 2.186708

C 5.054782 2.107025 0.725426

N -2.598507 0.348520 -0.165718

C -0.408584 1.875249 -0.231411

O -0.329908 2.221503 0.917978

C -3.838407 -0.259239 0.114542

C -4.545036 -0.925363 -0.885106

C -5.789749 -1.472113 -0.603860

C -6.335911 -1.360370 0.668984

C -5.631090 -0.688767 1.661424

C -4.390410 -0.130868 1.387495

O -0.603193 2.700594 -1.254524

C -0.822624 4.090212 -0.936645

C -2.252597 4.320352 -0.506189

O 3.092447 -3.098042 -0.706250

C 4.413945 -3.430878 -0.332683

H 0.310914 -3.550812 -0.021237

H 0.354193 -2.402609 1.327758

H 1.080259 -1.772232 -1.571565

H 1.049425 -0.039128 0.936268

H -0.237499 0.402756 -1.778467

H 2.787844 -1.917184 0.949286

H 3.786848 -0.870856 -1.677943

H 2.112156 0.812464 -1.763589

H 3.097682 1.667300 2.644409

H 4.371214 0.438992 2.771733

H 2.757189 -0.017424 2.210902

H 5.925457 1.741107 1.269817

H 5.332972 2.306221 -0.309158

H 4.713947 3.031524 1.192203

H -4.115535 -1.005646 -1.876676

H -6.333790 -1.988560 -1.385031

H -7.306168 -1.788879 0.884910

H -6.050287 -0.593804 2.655466

H -3.838289 0.401705 2.152287

H -0.113841 4.390501 -0.166061

H -0.593922 4.620322 -1.858756

H -2.407671 5.383684 -0.317717

H -2.942644 4.004075 -1.289384

H -2.478599 3.770061 0.407220

H 5.133740 -2.696639 -0.708442

H 4.509283 -3.489748 0.757191

H 4.640296 -4.403462 -0.766548

==============================

conf081_E1.62

C 0.213290 -2.499659 0.250646

C 1.198328 -1.632790 -0.490056

C 1.031844 -0.155879 -0.150803

C -0.265782 0.431257 -0.684825

C -1.528989 -0.318097 -0.281095

S -1.516781 -2.087711 -0.144654

C 2.661442 -1.887604 -0.142543

C 3.375064 -0.640128 -0.693245

C 2.303504 0.472204 -0.743578

O 2.865145 1.546708 -0.024488

O 4.408326 -0.107916 0.111026

C 3.952200 1.073763 0.763721

C 3.511326 0.767855 2.186525

C 5.054797 2.106804 0.724993

N -2.598626 0.348576 -0.165894

C -0.408438 1.875185 -0.231182

O -0.330056 2.221260 0.918283

C -3.838577 -0.259084 0.114310

C -4.390784 -0.130409 1.387144

C -5.631526 -0.688205 1.661005

C -6.336219 -1.359998 0.668602

C -5.789852 -1.472052 -0.604127

C -4.545076 -0.925412 -0.885297

O -0.602405 2.700747 -1.254234

C -0.821357 4.090406 -0.936176

C -2.251406 4.321102 -0.506272

O 3.092196 -3.098325 -0.706044

C 4.413670 -3.431216 -0.332434

H 0.310610 -3.550885 -0.020878

H 0.354154 -2.402568 1.328013

H 1.080009 -1.772545 -1.571440

H 1.049430 -0.039205 0.936241

H -0.237587 0.402808 -1.778400

H 2.787684 -1.917262 0.949364

H 3.786727 -0.871218 -1.677967

H 2.112046 0.812107 -1.763754

H 4.371173 0.439034 2.771569

H 2.757083 -0.017274 2.210810

H 3.097770 1.667463 2.644140

H 5.925465 1.740939 1.269431

H 5.332978 2.305806 -0.309631

H 4.714021 3.031405 1.191615

H -3.838774 0.402326 2.151905

H -6.050875 -0.592996 2.654959

H -7.306526 -1.788425 0.884471

H -6.333779 -1.988661 -1.385271

H -4.115435 -1.005926 -1.876789

H -0.112744 4.390221 -0.165250

H -0.592033 4.620568 -1.858101

H -2.406100 5.384468 -0.317674

H -2.941267 4.005269 -1.289808

H -2.478027 3.770749 0.406946

H 5.133542 -2.697170 -0.708422

H 4.509037 -3.489797 0.757453

H 4.639887 -4.403947 -0.766039

==============================

conf082_E1.63

C -0.006262 -2.549780 0.072826

C 1.022224 -1.670427 -0.590211

C 0.939744 -0.228670 -0.105059

C -0.324537 0.483986 -0.562920

C -1.627183 -0.228928 -0.229736

S -1.713092 -2.001209 -0.249846

C 2.469914 -2.037771 -0.277708

C 3.253163 -0.781387 -0.699739

C 2.241892 0.384636 -0.643350

O 2.856875 1.358849 0.168727

O 4.304841 -0.386688 0.158766

C 3.914343 0.759471 0.910550

C 3.454426 0.358705 2.303225

C 5.073527 1.728341 0.956899

N -2.662099 0.480215 -0.063164

C -0.361043 1.878389 0.041263

O -0.324799 2.094319 1.224212

C -3.933096 -0.088256 0.155698

C -4.671672 -0.602819 -0.908288

C -5.943504 -1.112071 -0.683911

C -6.485344 -1.112935 0.595764

C -5.748025 -0.592738 1.653273

C -4.479200 -0.073900 1.437580

O -0.396532 2.826486 -0.887983

C -0.394492 4.186852 -0.410880

C -0.424112 5.091499 -1.614911

O 2.834954 -3.206113 -0.963455

C 4.131305 -3.652958 -0.621817

H 0.025436 -3.571301 -0.305901

H 0.150953 -2.572214 1.151968

H 0.893275 -1.696903 -1.678819

H 0.967841 -0.225374 0.988096

H -0.299535 0.568365 -1.653644

H 2.592416 -2.185124 0.805137

H 3.661270 -0.935198 -1.700924

H 2.068721 0.825090 -1.627574

H 3.094170 1.238340 2.838164

H 4.292594 -0.069388 2.854457

H 2.655160 -0.379630 2.262343

H 5.366844 1.996268 -0.057805

H 4.783651 2.628925 1.498881

H 5.919880 1.269803 1.468588

H -4.244489 -0.596661 -1.904093

H -6.512511 -1.510402 -1.514883

H -7.477164 -1.511203 0.766963

H -6.163260 -0.586698 2.653501

H -3.900641 0.340355 2.254248

H -1.267198 4.329231 0.227480

H 0.502144 4.338907 0.191196

H 0.451526 4.928049 -2.243966

H -1.322349 4.917759 -2.208240

H -0.423464 6.130844 -1.285281

H 4.895709 -2.923815 -0.909128

H 4.209828 -3.837632 0.455293

H 4.308229 -4.582814 -1.159807

==============================

conf083_E1.64

C 0.213375 -2.499589 0.250706

C 1.198338 -1.632678 -0.490056

C 1.031856 -0.155789 -0.150726

C -0.265786 0.431308 -0.684764

C -1.528969 -0.318062 -0.281016

S -1.516737 -2.087650 -0.144402

C 2.661484 -1.887540 -0.142725

C 3.375066 -0.639998 -0.693334

C 2.303508 0.472352 -0.743473

O 2.865219 1.546756 -0.024283

O 4.408410 -0.107850 0.110885

C 3.952262 1.073683 0.763844

C 3.511438 0.767497 2.186613

C 5.054851 2.106747 0.725336

N -2.598641 0.348584 -0.165941

C -0.408519 1.875247 -0.231177

O -0.330102 2.221364 0.918270

C -3.838558 -0.259130 0.114289

C -4.545204 -0.925172 -0.885410

C -5.789947 -1.471874 -0.604228

C -6.336144 -1.360167 0.668607

C -5.631310 -0.688659 1.661100

C -4.390587 -0.130817 1.387235

O -0.602592 2.700751 -1.254266

C -0.821615 4.090415 -0.936306

C -2.251638 4.321045 -0.506272

O 3.092179 -3.098174 -0.706479

C 4.413635 -3.431194 -0.332928

H 0.310704 -3.550802 -0.020863

H 0.354354 -2.402534 1.328062

H 1.079898 -1.772372 -1.571434

H 1.049426 -0.039153 0.936321

H -0.237610 0.402835 -1.778337

H 2.787848 -1.917390 0.949159

H 3.786648 -0.871005 -1.678107

H 2.112006 0.812397 -1.763592

H 4.371356 0.438741 2.771589

H 2.757335 -0.017763 2.210843

H 3.097751 1.666982 2.644352

H 4.714099 3.031215 1.192237

H 5.925552 1.740729 1.269617

H 5.332968 2.306039 -0.309250

H -4.115681 -1.005426 -1.876974

H -6.333990 -1.988253 -1.385444

H -7.306434 -1.788631 0.884479

H -6.050524 -0.593717 2.655137

H -3.838462 0.401677 2.152081

H -0.112954 4.390360 -0.165475

H -0.592433 4.620538 -1.858291

H -2.941554 4.005032 -1.289688

H -2.478108 3.770795 0.407045

H -2.406405 5.384422 -0.317805

H 4.639880 -4.403768 -0.766873

H 5.133520 -2.697007 -0.708620

H 4.508954 -3.490169 0.756941

==============================

conf084_E1.65

C 0.213476 -2.499536 0.250811

C 1.198445 -1.632625 -0.489942

C 1.031889 -0.155714 -0.150704

C -0.265777 0.431281 -0.684773

C -1.528927 -0.318152 -0.280999

S -1.516612 -2.087752 -0.144549

C 2.661592 -1.887394 -0.142546

C 3.375097 -0.639894 -0.693347

C 2.303526 0.472454 -0.743451

O 2.865210 1.546837 -0.024190

O 4.408580 -0.107641 0.110594

C 3.952465 1.073812 0.763697

C 3.511887 0.767565 2.186527

C 5.054974 2.106954 0.725013

N -2.598599 0.348480 -0.165805

C -0.408644 1.875223 -0.231245

O -0.330116 2.221408 0.918177

C -3.838539 -0.259230 0.114362

C -4.544875 -0.925785 -0.885204

C -5.789648 -1.472466 -0.604077

C -6.336159 -1.360235 0.668566

C -5.631629 -0.688202 1.660930

C -4.390901 -0.130366 1.387112

O -0.603011 2.700636 -1.254346

C -0.822329 4.090266 -0.936425

C -2.252314 4.320550 -0.506098

O 3.092336 -3.098086 -0.706140

C 4.413851 -3.430942 -0.332655

H 0.310905 -3.550768 -0.020653

H 0.354328 -2.402376 1.328175

H 1.080064 -1.772387 -1.571317

H 1.049452 -0.039015 0.936340

H -0.237597 0.402763 -1.778346

H 2.787927 -1.917108 0.949346

H 3.786498 -0.871028 -1.678170

H 2.112046 0.812542 -1.763562

H 4.371855 0.438620 2.771319

H 2.757644 -0.017557 2.210813

H 3.098423 1.667073 2.644419

H 5.332952 2.306222 -0.309613

H 4.714204 3.031414 1.191915

H 5.925767 1.741027 1.269209

H -4.115117 -1.006457 -1.876631

H -6.333450 -1.989244 -1.385197

H -7.306458 -1.788691 0.884407

H -6.051096 -0.592856 2.654821

H -3.839018 0.402549 2.151838

H -0.113582 4.390441 -0.165763

H -0.593486 4.620392 -1.858493

H -2.942324 4.004395 -1.289374

H -2.478476 3.770250 0.407264

H -2.407269 5.383894 -0.317595

H 4.509170 -3.490150 0.757205

H 4.640276 -4.403373 -0.766823

H 5.133602 -2.696535 -0.708166

==============================

conf085_E1.66

C 0.267078 2.484089 0.524113

C -0.833893 1.884216 -0.320826

C -0.932771 0.381840 -0.071290

C 0.237857 -0.399515 -0.648530

C 1.618032 0.085122 -0.228417

S 1.908328 1.793799 0.137518

C -2.261229 2.401037 -0.016972

C -3.179156 1.197439 -0.251924

C -2.294281 0.021085 -0.667385

O -2.899424 -1.114284 -0.088277

O -3.729506 0.718854 0.965864

C -3.915492 -0.680782 0.826909

C -3.699033 -1.331139 2.172463

C -5.276480 -0.990591 0.231375

N 2.571019 -0.747086 -0.264823

C 0.106831 -1.868502 -0.275003

O 0.103529 -2.269268 0.859035

C 3.899782 -0.371701 0.018416

C 4.690766 0.214829 -0.967853

C 6.013569 0.534125 -0.693680

C 6.555402 0.272228 0.559004

C 5.764993 -0.318351 1.538229

C 4.443167 -0.646508 1.271836

O -0.021471 -2.642116 -1.348050

C -0.192664 -4.055676 -1.116833

C -1.629819 -4.382917 -0.786483

O -2.687464 3.446739 -0.857873

C -1.930725 4.631145 -0.729762

H 0.392710 3.553683 0.363769

H 0.062024 2.317058 1.582197

H -0.637011 2.063698 -1.384480

H -0.984499 0.207905 1.009565

H 0.208347 -0.305918 -1.738206

H -2.331287 2.712760 1.033566

H -3.961962 1.441475 -0.973063

H -2.226711 -0.107697 -1.750492

H -2.702244 -1.089422 2.541450

H -3.795307 -2.413157 2.079720

H -4.444378 -0.973613 2.883561

H -6.064551 -0.665817 0.911652

H -5.396410 -0.478965 -0.724662

H -5.371474 -2.064410 0.067137

H 4.262290 0.413694 -1.943032

H 6.622684 0.990087 -1.464423

H 7.587470 0.521741 0.769260

H 6.179460 -0.528418 2.516531

H 3.821837 -1.112298 2.027085

H 0.117454 -4.522616 -2.049402

H 0.484325 -4.363339 -0.320947

H -1.732289 -5.461590 -0.657819

H -1.939256 -3.888049 0.133184

H -2.290179 -4.067095 -1.594963

H -1.825309 4.920159 0.322069

H -0.936048 4.525392 -1.173313

H -2.466982 5.415326 -1.262389

==============================

conf086_E1.69

C 0.006300 -2.549826 -0.073016

C -1.022178 -1.670546 0.590134

C -0.939716 -0.228753 0.105077

C 0.324568 0.483913 0.562916

C 1.627221 -0.228963 0.229693

S 1.713130 -2.001245 0.249607

C -2.469875 -2.037870 0.277631

C -3.253131 -0.781503 0.699711

C -2.241856 0.384513 0.643420

O -2.856806 1.358796 -0.168600

O -4.304772 -0.386759 -0.158817

C -3.914280 0.759484 -0.910477

C -3.454342 0.358858 -2.303180

C -5.073462 1.728358 -0.956725

N 2.662128 0.480221 0.063249

C 0.361016 1.878317 -0.041268

O 0.324851 2.094224 -1.224224

C 3.933176 -0.088149 -0.155589

C 4.479373 -0.073614 -1.437432

C 5.748241 -0.592356 -1.653094

C 6.485519 -1.112627 -0.595593

C 5.943595 -1.111929 0.684046

C 4.671718 -0.602774 0.908388

O 0.396336 2.826431 0.887966

C 0.394105 4.186786 0.410833

C 0.423347 5.091470 1.614848

O -2.834914 -3.206247 0.963317

C -4.131297 -3.653032 0.621723

H -0.025346 -3.571379 0.305634

H -0.150976 -2.572190 -1.152151

H -0.893200 -1.697112 1.678734

H -0.967857 -0.225410 -0.988079

H 0.299606 0.568291 1.653640

H -2.592389 -2.185169 -0.805220

H -3.661274 -0.935362 1.700874

H -2.068699 0.824886 1.627683

H -3.094136 1.238553 -2.838057

H -4.292484 -0.069244 -2.854444

H -2.655031 -0.379432 -2.262339

H -4.783585 2.629003 -1.498605

H -5.919813 1.269875 -1.468468

H -5.366781 1.996169 0.058009

H 3.900858 0.340716 -2.254092

H 6.163549 -0.586174 -2.653290

H 7.477376 -1.510817 -0.766763

H 6.512567 -1.510312 1.515017

H 4.244468 -0.596735 1.904165

H 1.266887 4.329319 -0.227388

H -0.502463 4.338646 -0.191392

H 0.422508 6.130806 1.285190

H -0.452348 4.927837 2.243776

H 1.321536 4.917950 2.208314

H -4.895676 -2.923964 0.909293

H -4.209943 -3.837457 -0.455421

H -4.308146 -4.583016 1.159516

==============================

conf087_E1.70

C 1.294385 -2.985019 0.588108

C 1.660413 -1.797451 -0.265916

C 0.855689 -0.564179 0.125137

C -0.623160 -0.639172 -0.264458

C -1.344458 -1.895397 0.218950

S -0.463374 -3.415237 0.443622

C 3.101994 -1.326568 -0.089093

C 3.089407 0.111540 -0.624093

C 1.619741 0.588224 -0.532805

O 1.669458 1.743403 0.281780

O 3.794156 1.016326 0.206923

C 3.015928 2.203290 0.289917

C 3.292346 2.883497 1.606199

C 3.277236 3.098713 -0.910013

N -2.593545 -2.032128 0.384590

C -1.277809 0.622107 0.279897

O -1.534117 0.787130 1.444038

C -3.553202 -1.064623 0.036825

C -4.465178 -0.640966 1.005618

C -5.454336 0.271353 0.677292

C -5.566644 0.751364 -0.625039

C -4.678868 0.308095 -1.595451

C -3.673820 -0.594565 -1.269858

O -1.463167 1.527687 -0.672023

C -2.097574 2.763446 -0.284593

C -1.095463 3.746784 0.271967

O 3.980252 -2.194155 -0.754426

C 5.336964 -1.876801 -0.518050

H 1.826640 -3.886118 0.283206

H 1.525012 -2.780551 1.634494

H 1.499257 -2.024903 -1.325902

H 0.931546 -0.436455 1.210733

H -0.688108 -0.634992 -1.355881

H 3.348172 -1.287112 0.982162

H 3.471295 0.135310 -1.648677

H 1.205482 0.824900 -1.516888

H 4.333256 3.204233 1.647589

H 3.093266 2.192250 2.424748

H 2.651951 3.759717 1.709655

H 3.052279 2.571769 -1.839084

H 2.649843 3.988568 -0.852587

H 4.323793 3.404260 -0.924782

H -4.375716 -1.024805 2.014407

H -6.146524 0.605901 1.440066

H -6.346415 1.457493 -0.880185

H -4.763618 0.665452 -2.614435

H -2.982877 -0.944432 -2.027417

H -2.551070 3.133613 -1.202154

H -2.883938 2.536183 0.435241

H -0.317442 3.957222 -0.462149

H -1.607381 4.679546 0.514294

H -0.627456 3.356798 1.174793

H 5.587453 -0.881916 -0.900374

H 5.565010 -1.909522 0.552960

H 5.940885 -2.618911 -1.037508

==============================

conf088_E1.70

C -1.196088 -2.786589 -0.988888

C -1.583848 -1.794999 0.079755

C -0.812200 -0.490042 -0.074150

C 0.666333 -0.601078 0.308817

C 1.416388 -1.755374 -0.351811

S 0.572560 -3.195402 -0.938018

C -3.038251 -1.338067 -0.003341

C -3.061183 -0.020350 0.782456

C -1.607115 0.510345 0.769872

O -1.699008 1.782612 0.157512

O -3.803254 0.994656 0.130187

C -3.056355 2.199656 0.238079

C -3.367574 3.080281 -0.945097

C -3.324084 2.876090 1.572347

N 2.676203 -1.857011 -0.440901

C 1.297823 0.753691 0.020613

O 1.505799 1.589156 0.860168

C 3.563419 -0.941403 0.158094

C 4.378531 -0.142311 -0.643123

C 5.288264 0.725530 -0.057351

C 5.413234 0.787590 1.326645

C 4.619380 -0.028682 2.122680

C 3.695296 -0.888275 1.545333

O 1.500786 0.923737 -1.282323

C 2.023333 2.198582 -1.704309

C 0.915938 3.218492 -1.831190

O -3.888758 -2.336146 0.493697

C -5.255060 -2.019462 0.320260

H -1.704624 -3.742411 -0.862232

H -1.440783 -2.388644 -1.974601

H -1.409255 -2.216487 1.076258

H -0.891141 -0.173669 -1.119947

H 0.730474 -0.759378 1.388666

H -3.292696 -1.110270 -1.048859

H -3.432061 -0.191865 1.796982

H -1.193322 0.593748 1.778580

H -4.417560 3.372295 -0.925318

H -3.158938 2.539991 -1.868322

H -2.753583 3.980300 -0.907108

H -4.376745 3.150690 1.645284

H -3.077836 2.206347 2.398317

H -2.715763 3.776668 1.659743

H 4.279368 -0.199261 -1.720245

H 5.906364 1.354033 -0.686771

H 6.129212 1.461473 1.779319

H 4.714754 0.005358 3.200993

H 3.073103 -1.526122 2.161913

H 2.785848 2.515064 -0.992982

H 2.493482 1.996900 -2.664882

H 0.161662 2.877100 -2.541639

H 0.436880 3.391905 -0.868176

H 1.331761 4.159770 -2.193842

H -5.488638 -1.863499 -0.738637

H -5.835276 -2.860246 0.696757

H -5.530669 -1.118143 0.877278

==============================

conf089_E1.76

C 1.577446 -2.796538 0.578974

C 1.774727 -1.627983 -0.354456

C 0.924812 -0.439487 0.072710

C -0.572700 -0.621340 -0.173961

C -1.161005 -1.939823 0.319423

S -0.150401 -3.359228 0.619281

C 3.188001 -1.050439 -0.339723

C 3.015704 0.361678 -0.928192

C 1.538766 0.742583 -0.692749

O 1.584516 1.966841 0.007241

O 3.756070 1.389727 -0.300342

C 2.896397 2.170729 0.524419

C 2.989798 1.728242 1.976165

C 3.255148 3.629205 0.355199

N -2.398888 -2.174650 0.459094

C -1.252844 0.561658 0.500948

O -1.369435 0.666556 1.694809

C -3.395867 -1.257380 0.069593

C -3.604459 -0.965371 -1.277997

C -4.625447 -0.102767 -1.652803

C -5.444795 0.476029 -0.691920

C -5.245854 0.171724 0.650988

C -4.235502 -0.698314 1.032673

O -1.609729 1.492591 -0.371356

C -2.177100 2.698405 0.177061

C -2.510852 3.607235 -0.976685

O 4.056701 -1.879068 -1.066118

C 5.402553 -1.455148 -0.988617

H 2.144853 -3.672274 0.263962

H 1.886456 -2.524576 1.589263

H 1.529073 -1.913198 -1.383855

H 1.081731 -0.281367 1.144044

H -0.755554 -0.576109 -1.250592

H 3.540491 -0.958797 0.697625

H 3.280016 0.342534 -1.987425

H 1.001234 0.907231 -1.628216

H 2.277837 2.293952 2.578595

H 3.996306 1.918061 2.351127

H 2.775582 0.666016 2.085104

H 4.278091 3.801056 0.690529

H 3.167227 3.907658 -0.694657

H 2.583561 4.248267 0.950636

H -2.963491 -1.419430 -2.024403

H -4.777734 0.118776 -2.702043

H -6.237521 1.151989 -0.986034

H -5.883103 0.613099 1.407421

H -4.075167 -0.939379 2.076161

H -3.062545 2.428068 0.754353

H -1.446593 3.146748 0.851622

H -3.232666 3.135996 -1.645061

H -2.945610 4.531287 -0.594599

H -1.613588 3.856057 -1.544646

H 5.539351 -0.469143 -1.444333

H 5.738859 -1.410210 0.053141

H 6.004992 -2.182691 -1.530051

==============================

conf090_E1.77

C -0.153149 -2.405506 0.689025

C 0.944105 -1.822335 -0.165432

C 0.877678 -0.301057 -0.219322

C -0.335787 0.208141 -0.985581

C -1.672176 -0.292439 -0.463385

S -1.823666 -1.968034 0.105507

C 2.354137 -2.070087 0.366676

C 3.208026 -1.010713 -0.347769

C 2.225510 0.081156 -0.832823

O 2.743726 1.293268 -0.319524

O 4.068522 -0.312903 0.534664

C 4.076431 1.046132 0.122169

C 4.366294 1.919431 1.316254

C 5.054575 1.255997 -1.021576

N -2.676714 0.469768 -0.566147

C -0.335412 1.725142 -1.046744

O -0.296006 2.358699 -2.070201

C -3.973111 0.060627 -0.194797

C -4.720243 -0.772097 -1.025647

C -6.015670 -1.121384 -0.668913

C -6.572363 -0.646549 0.512357

C -5.826084 0.189440 1.335098

C -4.533751 0.551425 0.982639

O -0.335090 2.266249 0.164258

C -0.275297 3.702066 0.229584

C -0.228258 4.087810 1.685137

O 2.741400 -3.395752 0.122924

C 3.979075 -3.716547 0.724941

H -0.140696 -3.495364 0.680615

H -0.052058 -2.064056 1.719888

H 0.886695 -2.226513 -1.182737

H 0.854713 0.084050 0.805340

H -0.262078 -0.146305 -2.018271

H 2.383918 -1.859528 1.445547

H 3.767086 -1.466800 -1.169484

H 2.161419 0.118961 -1.924376

H 3.638157 1.718271 2.101835

H 4.306356 2.969224 1.028827

H 5.369545 1.716535 1.691147

H 6.067687 1.022190 -0.693050

H 4.801117 0.613420 -1.866634

H 5.019899 2.294547 -1.351402

H -4.281778 -1.135763 -1.947378

H -6.591499 -1.768502 -1.318987

H -7.582548 -0.921276 0.787435

H -6.253060 0.567099 2.255911

H -3.948041 1.209401 1.613131

H 0.613568 4.035578 -0.307419

H -1.154743 4.108974 -0.271610

H -0.183024 5.173874 1.770925

H -1.118317 3.736504 2.208369

H 0.654204 3.665572 2.167600

H 4.792082 -3.105186 0.319522

H 3.935454 -3.568698 1.809561

H 4.183996 -4.764440 0.512304

==============================

conf091_E1.78

C -0.152724 -2.405446 0.689182

C 0.944474 -1.822312 -0.165370

C 0.877963 -0.301043 -0.219430

C -0.335569 0.208054 -0.985619

C -1.671925 -0.292659 -0.463430

S -1.823276 -1.968171 0.105643

C 2.354527 -2.069920 0.366759

C 3.208361 -1.010429 -0.347604

C 2.225742 0.081142 -0.833068

O 2.743803 1.293510 -0.320215

O 4.068402 -0.312277 0.534989

C 4.076330 1.046663 0.122131

C 4.365613 1.920300 1.316123

C 5.054947 1.256325 -1.021247

N -2.676534 0.469446 -0.566353

C -0.335387 1.725067 -1.046688

O -0.295942 2.358680 -2.070105

C -3.972905 0.060223 -0.195013

C -4.720074 -0.772424 -1.025916

C -6.015479 -1.121758 -0.669167

C -6.572130 -0.647045 0.512177

C -5.825830 0.188873 1.334961

C -4.533512 0.550910 0.982479

O -0.335341 2.266093 0.164351

C -0.276400 3.701946 0.229751

C -0.231306 4.087722 1.685362

O 2.741919 -3.395536 0.122945

C 3.979471 -3.716319 0.725206

H -0.140209 -3.495306 0.680926

H -0.051638 -2.063857 1.719998

H 0.887078 -2.226618 -1.182623

H 0.855091 0.084151 0.805206

H -0.261862 -0.146308 -2.018338

H 2.384251 -1.859416 1.445641

H 3.767770 -1.466489 -1.169098

H 2.161630 0.118546 -1.924632

H 3.637067 1.719383 2.101389

H 4.305858 2.970016 1.028377

H 5.368664 1.717453 1.691577

H 5.020209 2.294762 -1.351421

H 6.067953 1.022820 -0.692184

H 4.801982 0.613425 -1.866207

H -4.281656 -1.135970 -1.947717

H -6.591333 -1.768809 -1.319287

H -7.582304 -0.921810 0.787260

H -6.252770 0.566447 2.255825

H -3.947795 1.208839 1.613017

H 0.612924 4.035926 -0.306207

H -1.155470 4.108374 -0.272480

H -1.121816 3.735931 2.207504

H 0.650793 3.665966 2.168904

H -0.186762 5.173810 1.771195

H 4.792445 -3.104466 0.320463

H 3.935416 -3.569142 1.809902

H 4.184789 -4.764019 0.512004

==============================

conf092_E1.77

C -0.153077 -2.405529 0.688919

C 0.944162 -1.822392 -0.165595

C 0.877797 -0.301120 -0.219538

C -0.335681 0.208114 -0.985772

C -1.672057 -0.292433 -0.463534

S -1.823611 -1.967894 0.105616

C 2.354183 -2.070192 0.366521

C 3.208130 -1.010658 -0.347651

C 2.225622 0.080983 -0.833131

O 2.743741 1.293330 -0.320295

O 4.068054 -0.312625 0.535169

C 4.076130 1.046343 0.122505

C 4.364987 1.919810 1.316717

C 5.055115 1.256140 -1.020522

N -2.676608 0.469758 -0.566451

C -0.335266 1.725109 -1.046921

O -0.295678 2.358666 -2.070373

C -3.972963 0.060582 -0.194909

C -4.720394 -0.771735 -1.025897

C -6.015734 -1.121088 -0.668928

C -6.572045 -0.646717 0.512716

C -5.825470 0.188873 1.335581

C -4.533209 0.550923 0.982891

O -0.335177 2.266218 0.164080

C -0.275467 3.702043 0.229399

C -0.228923 4.087824 1.684958

O 2.741508 -3.395804 0.122506

C 3.979001 -3.716760 0.724814

H -0.140691 -3.495387 0.680440

H -0.051854 -2.064150 1.719792

H 0.886723 -2.226605 -1.182884

H 0.854903 0.084039 0.805110

H -0.261990 -0.146338 -2.018459

H 2.383912 -1.859871 1.445435

H 3.767681 -1.466656 -1.169077

H 2.161529 0.118400 -1.924695

H 5.367940 1.717001 1.692462

H 3.636211 1.718722 2.101724

H 4.305256 2.969569 1.029115

H 6.068024 1.022600 -0.691179

H 4.802401 0.613302 -1.865608

H 5.020500 2.294602 -1.350631

H -4.282211 -1.135037 -1.947907

H -6.591798 -1.767891 -1.319108

H -7.582166 -0.921501 0.787973

H -6.252142 0.566177 2.256681

H -3.947272 1.208576 1.613511

H 0.613548 4.035582 -0.307337

H -1.154777 4.108888 -0.272082

H -0.183761 5.173893 1.770738

H -1.119144 3.736498 2.207903

H 0.653394 3.665639 2.167731

H 3.935000 -3.569347 1.809477

H 4.184067 -4.764553 0.511825

H 4.792112 -3.105185 0.319919

==============================

conf093_E1.80

C -0.152919 -2.405376 0.689201

C 0.944269 -1.822315 -0.165422

C 0.877894 -0.301041 -0.219458

C -0.335614 0.208151 -0.985653

C -1.671988 -0.292446 -0.463453

S -1.823488 -1.967805 0.105968

C 2.354327 -2.070080 0.366620

C 3.208236 -1.010585 -0.347654

C 2.225692 0.081033 -0.833130

O 2.743825 1.293391 -0.320336

O 4.068233 -0.312522 0.535036

C 4.076268 1.046433 0.122265

C 4.365322 1.919985 1.316370

C 5.055113 1.256138 -1.020908

N -2.676592 0.469657 -0.566601

C -0.335253 1.725163 -1.046734

O -0.295624 2.358766 -2.070152

C -3.972950 0.060416 -0.195105

C -4.720194 -0.772099 -1.026075

C -6.015535 -1.121537 -0.669213

C -6.572042 -0.647046 0.512297

C -5.825650 0.188714 1.335146

C -4.533372 0.550837 0.982569

O -0.335263 2.266199 0.164295

C -0.275936 3.702043 0.229695

C -0.230320 4.087789 1.685296

O 2.741636 -3.395707 0.122635

C 3.979160 -3.716641 0.724883

H -0.140521 -3.495236 0.680830

H -0.051639 -2.063909 1.720040

H 0.886765 -2.226590 -1.182681

H 0.855061 0.084164 0.805174

H -0.261918 -0.146248 -2.018357

H 2.384120 -1.859717 1.445525

H 3.767692 -1.466646 -1.169116

H 2.161553 0.118423 -1.924693

H 3.636652 1.718976 2.101496

H 4.305562 2.969715 1.028678

H 5.368322 1.717168 1.691973

H 6.068051 1.022585 -0.691668

H 4.802283 0.613266 -1.865930

H 5.020472 2.294586 -1.351056

H -4.281842 -1.135494 -1.947966

H -6.591465 -1.768491 -1.319361

H -7.582172 -0.921888 0.787463

H -6.252465 0.566094 2.256149

H -3.947568 1.208620 1.613176

H 0.613314 4.035802 -0.306519

H -1.155054 4.108691 -0.272277

H -0.185506 5.173867 1.771129

H -1.120750 3.736198 2.207706

H 0.651831 3.665829 2.168562

H 4.184349 -4.764366 0.511679

H 4.792202 -3.104881 0.320133

H 3.935135 -3.569459 1.809579

==============================

conf094_E1.81

C -0.152843 -2.405438 0.689255

C 0.944337 -1.822407 -0.165396

C 0.877937 -0.301139 -0.219513

C -0.335602 0.208014 -0.985664

C -1.671971 -0.292589 -0.463416

S -1.823415 -1.967992 0.105901

C 2.354407 -2.070129 0.366657

C 3.208291 -1.010542 -0.347525

C 2.225702 0.080894 -0.833289

O 2.743769 1.293429 -0.320858

O 4.067919 -0.312243 0.535362

C 4.076059 1.046613 0.122297

C 4.364673 1.920428 1.316324

C 5.055297 1.256136 -1.020577

N -2.676558 0.469534 -0.566455

C -0.335281 1.725028 -1.046802

O -0.295756 2.358595 -2.070243

C -3.972932 0.060379 -0.195014

C -4.720128 -0.772344 -1.025812

C -6.015525 -1.121629 -0.668987

C -6.572135 -0.646791 0.512328

C -5.825798 0.189192 1.335011

C -4.533484 0.551173 0.982458

O -0.335215 2.266118 0.164209

C -0.275823 3.701954 0.229540

C -0.229875 4.087763 1.685117

O 2.741774 -3.395713 0.122620

C 3.979264 -3.716665 0.724929

H -0.140425 -3.495298 0.680985

H -0.051622 -2.063865 1.720064

H 0.886846 -2.226742 -1.182632

H 0.855167 0.084121 0.805100

H -0.261954 -0.146406 -2.018368

H 2.384164 -1.859806 1.445572

H 3.768059 -1.466522 -1.168816

H 2.161527 0.117970 -1.924861

H 3.635738 1.719561 2.101240

H 4.304987 2.970099 1.028406

H 5.367546 1.717709 1.692320

H 6.068135 1.022713 -0.690940

H 4.802792 0.613102 -1.865571

H 5.020705 2.294520 -1.350932

H -4.281730 -1.136004 -1.947577

H -6.591407 -1.768744 -1.319016

H -7.582302 -0.921517 0.787471

H -6.252706 0.566856 2.255853

H -3.947730 1.209140 1.612920

H 0.613317 4.035671 -0.306884

H -1.155040 4.108621 -0.272248

H -0.184969 5.173841 1.770894

H -1.120216 3.736252 2.207733

H 0.652350 3.665763 2.168214

H 4.184443 -4.764395 0.511741

H 4.792337 -3.104925 0.320212

H 3.935187 -3.569474 1.809621

==============================

conf095_E1.81

C -0.152705 -2.405368 0.689396

C 0.944457 -1.822383 -0.165310

C 0.878021 -0.301118 -0.219506

C -0.335550 0.207997 -0.985622

C -1.671908 -0.292669 -0.463392

S -1.823277 -1.968065 0.105959

C 2.354542 -2.070041 0.366728

C 3.208389 -1.010464 -0.347514

C 2.225761 0.080911 -0.833335

O 2.743802 1.293495 -0.321005

O 4.067995 -0.312090 0.535332

C 4.076102 1.046745 0.122188

C 4.364678 1.920632 1.316169

C 5.055333 1.256223 -1.020696

N -2.676523 0.469414 -0.566473

C -0.335296 1.725020 -1.046715

O -0.295767 2.358619 -2.070135

C -3.972899 0.060243 -0.195089

C -4.533587 0.551199 0.982249

C -5.825912 0.189197 1.334756

C -6.572129 -0.646958 0.512148

C -6.015391 -1.121954 -0.669048

C -4.719985 -0.772662 -1.025812

O -0.335317 2.266079 0.164310

C -0.276212 3.701931 0.229658

C -0.230789 4.087756 1.685246

O 2.741935 -3.395627 0.122742

C 3.979419 -3.716538 0.725079

H -0.140244 -3.495230 0.681258

H -0.051506 -2.063677 1.720169

H 0.886951 -2.226780 -1.182519

H 0.855282 0.084177 0.805096

H -0.261904 -0.146380 -2.018341

H 2.384310 -1.859666 1.445634

H 3.768168 -1.466473 -1.168783

H 2.161560 0.117904 -1.924909

H 3.635738 1.719786 2.101086

H 4.304968 2.970287 1.028195

H 5.367551 1.717956 1.692188

H 5.020709 2.294586 -1.351109

H 6.068176 1.022845 -0.691045

H 4.802839 0.613130 -1.865648

H -3.947936 1.209316 1.612651

H -6.252914 0.566994 2.255500

H -7.582306 -0.921702 0.787241

H -6.591184 -1.769204 -1.319021

H -4.281491 -1.136449 -1.947481

H 0.613031 4.035808 -0.306498

H -1.155353 4.108427 -0.272399

H -1.121220 3.736060 2.207583

H 0.651374 3.665951 2.168625

H -0.186140 5.173844 1.771027

H 4.792440 -3.104594 0.320569

H 3.935231 -3.569596 1.809801

H 4.184763 -4.764191 0.511673

==============================

conf096_E1.87

C -0.179669 -2.513224 0.246454

C 0.887639 -1.745633 -0.490128

C 0.867852 -0.262939 -0.140773

C -0.362225 0.459153 -0.671452

C -1.695690 -0.161121 -0.280243

S -1.860204 -1.922126 -0.134805

C 2.317386 -2.146364 -0.138685

C 3.156343 -0.968281 -0.666537

C 2.197510 0.241196 -0.723289

O 2.851757 1.256217 0.003311

O 4.221006 -0.543845 0.161467

C 3.877604 0.680556 0.804971

C 3.393412 0.425760 2.223532

C 5.077141 1.599692 0.775450

N -2.698758 0.604932 -0.187905

C -0.335885 1.905024 -0.201265

O -0.304494 2.226612 0.957514

C -3.994299 0.116617 0.075373

C -4.542477 0.276059 1.346457

C -5.834016 -0.162872 1.601228

C -6.591307 -0.746980 0.592388

C -6.046720 -0.890883 -0.677957

C -4.752627 -0.462499 -0.940376

O -0.318941 2.758372 -1.219895

C -0.235027 4.161770 -0.895114

C 1.189928 4.564752 -0.596274

O 2.631692 -3.385974 -0.715961

C 3.908140 -3.854621 -0.331206

H -0.192751 -3.565940 -0.035310

H -0.024658 -2.442169 1.323842

H 0.758565 -1.867235 -1.572318

H 0.892135 -0.158825 0.947459

H -0.331583 0.438721 -1.765079

H 2.432691 -2.202246 0.953578

H 3.561722 -1.229638 -1.646228

H 2.047720 0.599262 -1.744200

H 3.067277 1.364012 2.674300

H 4.209739 0.014653 2.818756

H 2.564092 -0.279537 2.241565

H 4.822146 2.557076 1.230902

H 5.900048 1.154608 1.334982

H 5.387809 1.762345 -0.256281

H -3.948402 0.739860 2.124444

H -6.251183 -0.043657 2.593533

H -7.600579 -1.082405 0.793611

H -6.630836 -1.339981 -1.471694

H -4.322990 -0.569221 -1.929390

H -0.614423 4.667879 -1.780281

H -0.897293 4.366160 -0.054884

H 1.565405 4.045883 0.285020

H 1.837637 4.336052 -1.443694

H 1.229118 5.639454 -0.412328

H 4.044856 -4.836208 -0.781888

H 4.702500 -3.187666 -0.681499

H 3.979583 -3.943784 0.758491

==============================

conf097_E1.90

C 1.535685 -2.776914 0.624458

C 1.801432 -1.635832 -0.324801

C 0.960297 -0.416814 0.029576

C -0.532599 -0.578069 -0.281667

C -1.169954 -1.834850 0.297485

S -0.206802 -3.288944 0.606640

C 3.226560 -1.092131 -0.262931

C 3.114917 0.304850 -0.898498

C 1.638829 0.726684 -0.741280

O 1.684584 1.969073 -0.073378

O 3.852439 1.333825 -0.268868

C 2.976328 2.154574 0.497826

C 2.993647 1.745469 1.962365

C 3.377842 3.599710 0.311329

N -2.405400 -2.024462 0.503808

C -1.187325 0.688447 0.246103

O -1.462824 0.866244 1.404315

C -3.435764 -1.153416 0.112152

C -3.598857 -0.760024 -1.216582

C -4.707595 -0.009930 -1.589970

C -5.652148 0.368484 -0.646434

C -5.485619 -0.016638 0.681015

C -4.394637 -0.782513 1.057829

O -1.289175 1.608422 -0.705400

C -1.698843 2.928013 -0.293472

C -3.203481 3.023091 -0.209318

O 4.104255 -1.962259 -0.927642

C 5.456067 -1.570877 -0.799816

H 2.091349 -3.673907 0.350786

H 1.811886 -2.491567 1.640636

H 1.595053 -1.941154 -1.357088

H 1.069531 -0.228006 1.102072

H -0.649026 -0.623405 -1.367537

H 3.536788 -0.979548 0.785805

H 3.424888 0.247543 -1.943899

H 1.147880 0.876618 -1.703748

H 3.987403 1.918049 2.377526

H 2.745831 0.692289 2.086735

H 2.271349 2.344260 2.518777

H 2.693765 4.248387 0.859145

H 4.387635 3.756696 0.690933

H 3.346281 3.853870 -0.747836

H -2.874050 -1.070574 -1.959323

H -4.828792 0.278689 -2.626956

H -6.513285 0.954995 -0.940244

H -6.217373 0.272198 1.425435

H -4.270291 -1.105082 2.084278

H -1.224978 3.156298 0.660051

H -1.297773 3.589249 -1.058898

H -3.656567 2.826753 -1.181490

H -3.596421 2.308037 0.514784

H -3.485829 4.028766 0.106440

H 5.638712 -0.599657 -1.270925

H 5.747241 -1.509934 0.254666

H 6.062487 -2.325859 -1.297448

==============================

conf098_E1.90

C -0.179545 -2.513261 0.246252

C 0.887809 -1.745599 -0.490190

C 0.867902 -0.262909 -0.140799

C -0.362193 0.459097 -0.671518

C -1.695652 -0.161274 -0.280348

S -1.860061 -1.922323 -0.135320

C 2.317554 -2.146268 -0.138660

C 3.156469 -0.968119 -0.666439

C 2.197581 0.241319 -0.723205

O 2.851732 1.256334 0.003505

O 4.221120 -0.543622 0.161537

C 3.877618 0.680693 0.805142

C 3.393437 0.425823 2.223697

C 5.077104 1.599900 0.775678

N -2.698710 0.604762 -0.187777

C -0.336016 1.904988 -0.201337

O -0.304439 2.226596 0.957429

C -3.994245 0.116421 0.075530

C -4.752579 -0.462803 -0.940150

C -6.046660 -0.891181 -0.677671

C -6.591226 -0.747156 0.592676

C -5.833935 -0.162934 1.601444

C -4.542406 0.276003 1.346605

O -0.319439 2.758327 -1.219978

C -0.235720 4.161749 -0.895237

C 1.189226 4.564999 -0.596699

O 2.631959 -3.385845 -0.715968

C 3.908431 -3.854422 -0.331208

H -0.192476 -3.565993 -0.035468

H -0.024713 -2.442152 1.323662

H 0.758839 -1.867169 -1.572397

H 0.892132 -0.158817 0.947434

H -0.331531 0.438680 -1.765144

H 2.432788 -2.202219 0.953603

H 3.561855 -1.229464 -1.646132

H 2.047858 0.599438 -1.744107

H 2.564070 -0.279415 2.241731

H 3.067363 1.364075 2.674509

H 4.209755 0.014648 2.818887

H 4.822055 2.557237 1.231195

H 5.900034 1.154809 1.335170

H 5.387752 1.762632 -0.256046

H -4.322949 -0.569609 -1.929159

H -6.630777 -1.340385 -1.471345

H -7.600487 -1.082591 0.793946

H -6.251101 -0.043622 2.593738

H -3.948348 0.739931 2.124528

H -0.615379 4.667772 -1.780340

H -0.897860 4.366030 -0.054881

H 1.564965 4.046249 0.284554

H 1.836792 4.336357 -1.444243

H 1.228274 5.639715 -0.412819

H 3.979859 -3.943631 0.758488

H 4.045224 -4.835981 -0.781927

H 4.702755 -3.187397 -0.681447

==============================

conf099_E1.92

C -0.179453 -2.513282 0.246131

C 0.887953 -1.745583 -0.490190

C 0.868000 -0.262885 -0.140830

C -0.362110 0.459093 -0.671559

C -1.695560 -0.161336 -0.280466

S -1.859950 -1.922425 -0.135699

C 2.317678 -2.146219 -0.138558

C 3.156585 -0.968087 -0.666396

C 2.197672 0.241336 -0.723269

O 2.851816 1.256427 0.003350

O 4.221242 -0.543478 0.161505

C 3.877687 0.680856 0.805045

C 3.393457 0.426095 2.223605

C 5.077156 1.600070 0.775560

N -2.698624 0.604656 -0.187710

C -0.336080 1.904961 -0.201329

O -0.304407 2.226511 0.957455

C -3.994122 0.116250 0.075628

C -4.752504 -0.462868 -0.940082

C -6.046551 -0.891327 -0.677576

C -6.591046 -0.747491 0.592823

C -5.833705 -0.163374 1.601617

C -4.542204 0.275623 1.346760

O -0.319861 2.758364 -1.219919

C -0.236611 4.161789 -0.895102

C 1.188195 4.565528 -0.596589

O 2.632127 -3.385818 -0.715802

C 3.908614 -3.854323 -0.331004

H -0.192321 -3.566020 -0.035568

H -0.024780 -2.442140 1.323561

H 0.759093 -1.867158 -1.572411

H 0.892232 -0.158768 0.947401

H -0.331409 0.438689 -1.765189

H 2.432864 -2.202142 0.953708

H 3.561956 -1.229528 -1.646069

H 2.047952 0.599369 -1.744202

H 2.564049 -0.279092 2.241663

H 3.067418 1.364391 2.674355

H 4.209735 0.014916 2.818845

H 5.387790 1.762813 -0.256167

H 4.822131 2.557407 1.231094

H 5.900095 1.154976 1.335038

H -4.322930 -0.569522 -1.929133

H -6.630713 -1.340420 -1.471282

H -7.600292 -1.082959 0.794107

H -6.250808 -0.044226 2.593958

H -3.948099 0.739429 2.124722

H -0.616490 4.667744 -1.780150

H -0.898781 4.365792 -0.054703

H 1.564132 4.046951 0.284685

H 1.835841 4.337100 -1.444132

H 1.226859 5.640267 -0.412749

H 4.045435 -4.835918 -0.781636

H 4.702920 -3.187312 -0.681313

H 3.980054 -3.943430 0.758697

==============================

conf100_E1.93

C 0.075940 -2.504077 0.518703

C 1.111147 -1.806748 -0.335015

C 1.076833 -0.302512 -0.082662

C -0.165905 0.373703 -0.640993

C -1.492972 -0.235745 -0.214161

S -1.624930 -1.964086 0.147871

C 2.582338 -2.190871 -0.042648

C 3.385236 -0.909254 -0.288481

C 2.392515 0.181494 -0.693779

O 2.897227 1.368127 -0.120307

O 3.903950 -0.381084 0.922793

C 3.961266 1.029302 0.780790

C 3.705175 1.660429 2.128408

C 5.280463 1.458788 0.165619

N -2.516621 0.508381 -0.242745

C -0.159697 1.842530 -0.247339

O -0.192619 2.225410 0.892437

C -3.804748 0.016974 0.050145

C -4.553542 -0.625274 -0.934241

C -5.840672 -1.060337 -0.649608

C -6.388531 -0.859110 0.611597

C -5.640812 -0.212369 1.588945

C -4.355381 0.231339 1.312290

O -0.075008 2.639112 -1.307717

C -0.050290 4.058208 -1.053247

C -1.447663 4.588679 -0.832597

O 3.095430 -3.197045 -0.882959

C 2.448672 -4.444060 -0.746735

H 0.045565 -3.580593 0.357828

H 0.274226 -2.319746 1.575248

H 0.922623 -2.002502 -1.397303

H 1.126477 -0.127299 0.998126

H -0.138834 0.296296 -1.731883

H 2.688598 -2.490385 1.008441

H 4.178784 -1.082266 -1.018397

H 2.301495 0.303214 -1.775875

H 4.496060 1.384846 2.826575

H 2.745238 1.320070 2.516768

H 3.688040 2.746045 2.029653

H 5.430127 0.960575 -0.793301

H 5.278300 2.536865 0.002375

H 6.104352 1.203954 0.833108

H -4.120883 -0.776412 -1.916094

H -6.416952 -1.559339 -1.418933

H -7.392722 -1.199049 0.829962

H -6.060553 -0.048706 2.573835

H -3.768037 0.741659 2.066024

H 0.595479 4.248476 -0.196817

H 0.402142 4.487552 -1.944658

H -2.076468 4.380004 -1.699001

H -1.901092 4.137803 0.050130

H -1.406842 5.669220 -0.687781

H 1.449489 -4.432064 -1.192576

H 3.054533 -5.180365 -1.273115

H 2.368004 -4.733841 0.307038

==============================

conf101_E1.93

C 0.075833 -2.504127 0.518381

C 1.111103 -1.806765 -0.335237

C 1.076827 -0.302536 -0.082839

C -0.165855 0.373766 -0.641162

C -1.492946 -0.235659 -0.214385

S -1.625013 -1.963980 0.147635

C 2.582245 -2.190915 -0.042697

C 3.385207 -0.909311 -0.288449

C 2.392550 0.181446 -0.693865

O 2.897221 1.368083 -0.120371

O 3.903775 -0.381132 0.922881

C 3.961107 1.029255 0.780891

C 3.704777 1.660421 2.128442

C 5.280418 1.458708 0.165939

N -2.516575 0.508504 -0.242889

C -0.159545 1.842555 -0.247385

O -0.192223 2.225320 0.892439

C -3.804650 0.017024 0.050147

C -4.553713 -0.624765 -0.934329

C -5.840792 -1.059894 -0.649561

C -6.388309 -0.859217 0.611880

C -5.640320 -0.212920 1.589313

C -4.354942 0.230853 1.312528

O -0.075063 2.639210 -1.307728

C -0.050314 4.058299 -1.053211

C -1.447647 4.588807 -0.832367

O 3.095378 -3.197121 -0.882913

C 2.448558 -4.444097 -0.746643

H 0.045379 -3.580622 0.357378

H 0.274129 -2.319932 1.574948

H 0.922666 -2.002491 -1.397547

H 1.126447 -0.127367 0.997958

H -0.138779 0.296444 -1.732058

H 2.688348 -2.490411 1.008415

H 4.178836 -1.082348 -1.018270

H 2.301624 0.303124 -1.775973

H 2.744778 1.320065 2.516657

H 3.687628 2.746032 2.029623

H 4.495548 1.384905 2.826768

H 6.104180 1.203847 0.833574

H 5.430222 0.960457 -0.792942

H 5.278345 2.536780 0.002665

H -4.121308 -0.775475 -1.916360

H -6.417291 -1.558527 -1.418961

H -7.392454 -1.199213 0.830371

H -6.059805 -0.049685 2.574384

H -3.767382 0.740819 2.066335

H 0.595590 4.248553 -0.196882

H 0.401990 4.487650 -1.944682

H -1.406785 5.669356 -0.687579

H -2.076582 4.380135 -1.698678

H -1.900960 4.137966 0.050434

H 1.449444 -4.432112 -1.192642

H 3.054469 -5.180488 -1.272847

H 2.367715 -4.733748 0.307152

==============================

conf102_E1.96

C -0.178998 -2.513436 0.246197

C 0.888259 -1.745562 -0.490166

C 0.868141 -0.262858 -0.140821

C -0.362031 0.458959 -0.671672

C -1.695427 -0.161596 -0.280511

S -1.859604 -1.922651 -0.135197

C 2.318046 -2.146095 -0.138610

C 3.156840 -0.967846 -0.666336

C 2.197758 0.241441 -0.723304

O 2.851922 1.256711 0.003032

O 4.221292 -0.543126 0.161826

C 3.877436 0.681222 0.805191

C 3.392823 0.426435 2.223635

C 5.076859 1.600536 0.776157

N -2.698531 0.604359 -0.187756

C -0.336231 1.904839 -0.201437

O -0.304174 2.226391 0.957322

C -3.994025 0.115939 0.075601

C -4.752081 -0.464030 -0.939867

C -6.046146 -0.892434 -0.677377

C -6.590957 -0.747727 0.592791

C -5.833948 -0.162772 1.601340

C -4.542446 0.276233 1.346468

O -0.320579 2.758272 -1.220037

C -0.237591 4.161694 -0.895203

C 1.187239 4.565828 -0.597278

O 2.632578 -3.385613 -0.716001

C 3.909022 -3.854184 -0.331131

H -0.191805 -3.566119 -0.035718

H -0.024134 -2.442485 1.323612

H 0.759361 -1.867165 -1.572376

H 0.892205 -0.158677 0.947399

H -0.331322 0.438527 -1.765292

H 2.433276 -2.202085 0.953646

H 3.562474 -1.229163 -1.645931

H 2.047885 0.599323 -1.744269

H 3.066281 1.364649 2.674187

H 4.209123 0.015660 2.819126

H 2.563732 -0.279121 2.241607

H 4.821573 2.557827 1.231632

H 5.899640 1.155493 1.335900

H 5.387836 1.763347 -0.255454

H -4.322254 -0.571357 -1.928735

H -6.630059 -1.342206 -1.470878

H -7.600200 -1.083203 0.794081

H -6.251312 -0.042931 2.593487

H -3.948616 0.740759 2.124206

H -0.617975 4.667582 -1.780074

H -0.899474 4.365494 -0.054522

H 1.563697 4.047268 0.283782

H 1.834568 4.337604 -1.445122

H 1.225713 5.640561 -0.413399

H 3.980304 -3.943535 0.758559

H 4.045922 -4.835673 -0.781971

H 4.703360 -3.187073 -0.681178

==============================

conf103_E1.98

C -0.205929 -2.587361 0.178870

C 0.881037 -1.760103 -0.458368

C 0.861543 -0.317059 0.030975

C -0.356233 0.460368 -0.449407

C -1.703282 -0.189141 -0.171492

S -1.875433 -1.954080 -0.178297

C 2.299100 -2.205192 -0.108731

C 3.159718 -0.983996 -0.464668

C 2.201015 0.230659 -0.468058

O 2.788841 1.159625 0.421599

O 4.105356 -0.660341 0.538354

C 4.138524 0.756626 0.638106

C 4.547640 1.140203 2.037102

C 5.041045 1.350262 -0.430716

N -2.711670 0.568667 -0.068765

C -0.333010 1.848767 0.168856

O -0.382480 2.055583 1.352733

C -4.016786 0.060076 0.087935

C -4.612304 0.065890 1.347716

C -5.913707 -0.391854 1.498173

C -6.633217 -0.843679 0.397879

C -6.040442 -0.836111 -0.859026

C -4.736725 -0.386660 -1.018585

O -0.199553 2.800155 -0.748542

C -0.097136 4.151176 -0.256522

C 0.087573 5.055535 -1.446913

O 2.627413 -3.377186 -0.805114

C 3.880345 -3.903340 -0.417112

H -0.216336 -3.608215 -0.202618

H -0.073265 -2.620241 1.260887

H 0.777856 -1.778031 -1.549627

H 0.882509 -0.318361 1.126177

H -0.292947 0.555705 -1.537628

H 2.377312 -2.374303 0.975058

H 3.645091 -1.136332 -1.432837

H 2.097257 0.663322 -1.467618

H 3.866774 0.684625 2.755692

H 4.516574 2.224163 2.147218

H 5.563840 0.797971 2.233060

H 6.064604 1.002074 -0.289288

H 4.703415 1.055732 -1.425978

H 5.024712 2.438426 -0.365236

H -4.046809 0.426178 2.198459

H -6.368162 -0.391753 2.481235

H -7.650006 -1.194703 0.518545

H -6.594487 -1.181932 -1.722973

H -4.269336 -0.375389 -1.996084

H -1.006738 4.386810 0.297127

H 0.748478 4.203390 0.430297

H 0.168724 6.088247 -1.106539

H 0.997226 4.798462 -1.990739

H -0.762403 4.981346 -2.125968

H 4.693712 -3.204695 -0.638800

H 3.893808 -4.129648 0.654700

H 4.037986 -4.821329 -0.980742

==============================

conf104_E1.98

C -0.206078 -2.587331 0.178909

C 0.880880 -1.760090 -0.458375

C 0.861483 -0.317056 0.030992

C -0.356250 0.460450 -0.449379

C -1.703313 -0.188979 -0.171415

S -1.875586 -1.953893 -0.177956

C 2.298936 -2.205270 -0.108823

C 3.159615 -0.984102 -0.464719

C 2.200979 0.230599 -0.468042

O 2.788859 1.159490 0.421668

O 4.105286 -0.660545 0.538306

C 4.138519 0.756416 0.638149

C 4.547656 1.139894 2.037172

C 5.041065 1.350051 -0.430658

N -2.711685 0.568882 -0.068810

C -0.332929 1.848851 0.168887

O -0.382459 2.055669 1.352761

C -4.016809 0.060252 0.087894

C -4.736730 -0.386356 -1.018693

C -6.040428 -0.835865 -0.859193

C -6.633197 -0.843621 0.397718

C -5.913695 -0.391941 1.498071

C -4.612300 0.065859 1.347676

O -0.199290 2.800215 -0.748508

C -0.096734 4.151228 -0.256500

C 0.088279 5.055539 -1.446885

O 2.627158 -3.377242 -0.805303

C 3.880037 -3.903542 -0.417335

H -0.216593 -3.608139 -0.202695

H -0.073279 -2.620330 1.260906

H 0.777642 -1.777999 -1.549629

H 0.882448 -0.318360 1.126195

H -0.292971 0.555792 -1.537599

H 2.377196 -2.374457 0.974949

H 3.644970 -1.136424 -1.432900

H 2.097243 0.663328 -1.467576

H 4.516675 2.223851 2.147338

H 5.563822 0.797564 2.233132

H 3.866732 0.684342 2.755723

H 4.703408 1.055543 -1.425917

H 5.024766 2.438212 -0.365156

H 6.064611 1.001819 -0.289247

H -4.269316 -0.374963 -1.996177

H -6.594479 -1.181578 -1.723180

H -7.649977 -1.194685 0.518340

H -6.368143 -0.392011 2.481136

H -4.046806 0.426024 2.198472

H -1.006374 4.387016 0.297021

H 0.748796 4.203330 0.430431

H 0.169548 6.088242 -1.106519

H 0.997962 4.798306 -1.990583

H -0.761618 4.981468 -2.126051

H 4.693467 -3.204939 -0.638928

H 3.893462 -4.129971 0.654452

H 4.037614 -4.821482 -0.981064

==============================

conf105_E2.01

C 0.116725 2.393394 -0.755129

C 1.177173 1.850427 0.176038

C 1.098484 0.328962 0.241378

C -0.128867 -0.175624 0.983478

C -1.452575 0.319174 0.424314

S -1.578874 1.958349 -0.241150

C 2.641303 2.122154 -0.254426

C 3.420341 0.872278 0.169516

C 2.435703 -0.059706 0.872215

O 2.878160 -1.359958 0.552726

O 3.782556 0.085978 -0.956847

C 3.788771 -1.276369 -0.553116

C 3.279924 -2.120821 -1.698316

C 5.167775 -1.689769 -0.076413

N -2.470668 -0.420674 0.556355

C -0.134720 -1.693496 1.041288

O -0.104975 -2.328133 2.063757

C -3.752645 -0.007082 0.140862

C -4.531555 0.805969 0.961903

C -5.811575 1.166139 0.562891

C -6.322189 0.720126 -0.650096

C -5.544137 -0.095910 -1.463377

C -4.265741 -0.466173 -1.070633

O -0.136548 -2.224634 -0.176125

C -0.145837 -3.660362 -0.266436

C -1.544807 -4.200842 -0.079961

O 3.226111 3.236971 0.375295

C 2.602864 4.462607 0.056895

H 0.103998 3.481812 -0.794860

H 0.274207 2.014180 -1.765406

H 1.046609 2.263680 1.183082

H 1.087532 -0.063046 -0.781081

H -0.081051 0.180414 2.017019

H 2.698683 2.235749 -1.344890

H 4.294234 1.137139 0.768521

H 2.412630 0.062411 1.957427

H 3.946316 -2.027970 -2.556572

H 2.278817 -1.793523 -1.979434

H 3.243220 -3.167713 -1.395548

H 5.137785 -2.711474 0.303308

H 5.878555 -1.640363 -0.902024

H 5.501670 -1.027538 0.723682

H -4.127745 1.147996 1.907414

H -6.411862 1.798901 1.204952

H -7.320814 1.002654 -0.957588

H -5.934406 -0.449934 -2.409561

H -3.654210 -1.104653 -1.696558

H 0.231821 -3.875158 -1.264094

H 0.546936 -4.063160 0.471788

H -1.534545 -5.285382 -0.198363

H -1.925508 -3.964093 0.913521

H -2.219269 -3.778810 -0.826137

H 2.487523 4.576058 -1.027041

H 1.621345 4.552662 0.532509

H 3.245285 5.258035 0.432140

==============================

conf106_E2.01

C 0.116704 2.393328 -0.755259

C 1.177163 1.850452 0.175951

C 1.098519 0.328998 0.241440

C -0.128808 -0.175600 0.983568

C -1.452538 0.319158 0.424444

S -1.578889 1.958202 -0.241321

C 2.641273 2.122133 -0.254617

C 3.420346 0.872332 0.169516

C 2.435731 -0.059562 0.872352

O 2.878228 -1.359848 0.553114

O 3.782566 0.085841 -0.956722

C 3.788726 -1.276444 -0.552844

C 3.279711 -2.121051 -1.697864

C 5.167744 -1.689870 -0.076203

N -2.470604 -0.420713 0.556527

C -0.134622 -1.693464 1.041282

O -0.105019 -2.328190 2.063698

C -3.752588 -0.007137 0.141044

C -4.266000 -0.466878 -1.070060

C -5.544395 -0.096583 -1.462807

C -6.322134 0.720098 -0.649888

C -5.811220 1.166735 0.562760

C -4.531199 0.806573 0.961742

O -0.136284 -2.224461 -0.176212

C -0.145851 -3.660167 -0.266757

C -1.544944 -4.200456 -0.080618

O 3.226082 3.237073 0.374855

C 2.602706 4.462618 0.056350

H 0.103889 3.481745 -0.794993

H 0.274243 2.014089 -1.765517

H 1.046604 2.263803 1.182956

H 1.087592 -0.063099 -0.780985

H -0.080987 0.180405 2.017121

H 2.698585 2.235516 -1.345110

H 4.294243 1.137308 0.768460

H 2.412635 0.062764 1.957542

H 3.946060 -2.028464 -2.556187

H 2.278629 -1.793693 -1.979007

H 3.242893 -3.167879 -1.394889

H 5.501724 -1.027566 0.723800

H 5.137752 -2.711538 0.303621

H 5.878470 -1.640571 -0.901868

H -3.654743 -1.105907 -1.695698

H -5.934905 -0.451106 -2.408707

H -7.320751 1.002647 -0.957388

H -6.411266 1.799998 1.204553

H -4.127166 1.149059 1.906993

H 0.231909 -3.874846 -1.264404

H 0.546766 -4.063237 0.471466

H -2.219207 -3.778158 -0.826826

H -1.534839 -5.284975 -0.199256

H -1.925789 -3.963869 0.912844

H 1.621086 4.552538 0.531780

H 3.244946 5.258132 0.431720

H 2.487556 4.576056 -1.027606

==============================

conf107_E2.03

C 0.116741 2.393334 -0.755198

C 1.177211 1.850436 0.175984

C 1.098563 0.328983 0.241449

C -0.128751 -0.175617 0.983568

C -1.452482 0.319123 0.424424

S -1.578858 1.958196 -0.241267

C 2.641315 2.122132 -0.254576

C 3.420385 0.872322 0.169532

C 2.435782 -0.059562 0.872378

O 2.878348 -1.359831 0.553211

O 3.782554 0.085839 -0.956733

C 3.788594 -1.276479 -0.552945

C 3.279246 -2.120936 -1.697934

C 5.167657 -1.690131 -0.076628

N -2.470554 -0.420745 0.556478

C -0.134588 -1.693492 1.041314

O -0.105017 -2.328195 2.063738

C -3.752515 -0.007120 0.140987

C -4.531171 0.806430 0.961783

C -5.811194 1.166639 0.562806

C -6.322033 0.720224 -0.649942

C -5.544231 -0.096304 -1.462976

C -4.265863 -0.466657 -1.070235

O -0.136255 -2.224518 -0.176169

C -0.145987 -3.660233 -0.266660

C -1.545137 -4.200356 -0.080438

O 3.226139 3.237055 0.374910

C 2.602719 4.462609 0.056516

H 0.103928 3.481751 -0.794909

H 0.274262 2.014129 -1.765471

H 1.046661 2.263758 1.183003

H 1.087637 -0.063099 -0.780980

H -0.080953 0.180382 2.017123

H 2.698635 2.235524 -1.345066

H 4.294309 1.137270 0.768448

H 2.412644 0.062778 1.957564

H 3.945355 -2.028216 -2.556426

H 2.278071 -1.793570 -1.978736

H 3.242530 -3.167802 -1.395071

H 5.501913 -1.027870 0.723296

H 5.137579 -2.711794 0.303202

H 5.878192 -1.640941 -0.902464

H -4.127177 1.148780 1.907102

H -6.411274 1.799785 1.204683

H -7.320644 1.002795 -0.957443

H -5.934701 -0.450657 -2.408957

H -3.654547 -1.105550 -1.695954

H 0.231691 -3.874989 -1.264317

H 0.546609 -4.063358 0.471550

H -1.535118 -5.284895 -0.198897

H -1.925948 -3.963571 0.912990

H -2.219363 -3.778116 -0.826711

H 3.245059 5.258115 0.431730

H 2.487342 4.576038 -1.027419

H 1.621202 4.552551 0.532150

==============================

conf108_E2.06

C -0.206270 -2.587332 0.178869

C 0.880723 -1.760130 -0.458420

C 0.861395 -0.317107 0.031005

C -0.356296 0.460465 -0.449354

C -1.703395 -0.188894 -0.171399

S -1.875755 -1.953797 -0.177906

C 2.298759 -2.205384 -0.108886

C 3.159507 -0.984237 -0.464678

C 2.200923 0.230508 -0.467997

O 2.788831 1.159364 0.421731

O 4.105114 -0.660758 0.538435

C 4.138469 0.756194 0.638230

C 4.547603 1.139691 2.037247

C 5.041081 1.349766 -0.430555

N -2.711741 0.569006 -0.068843

C -0.332872 1.848870 0.168897

O -0.382586 2.055720 1.352757

C -4.016883 0.060442 0.087870

C -4.612272 0.065771 1.347698

C -5.913710 -0.391932 1.498073

C -6.633350 -0.843235 0.397663

C -6.040675 -0.835217 -0.859298

C -4.736948 -0.385802 -1.018781

O -0.198889 2.800188 -0.748498

C -0.096063 4.151195 -0.256525

C 0.089616 5.055389 -1.446896

O 2.626944 -3.377336 -0.805415

C 3.879815 -3.903676 -0.417477

H -0.216850 -3.608133 -0.202754

H -0.073426 -2.620367 1.260860

H 0.777461 -1.777989 -1.549670

H 0.882360 -0.318466 1.126207

H -0.293017 0.555796 -1.537576

H 2.376997 -2.374637 0.974877

H 3.644924 -1.136508 -1.432834

H 2.097213 0.663239 -1.467531

H 4.516606 2.223651 2.147390

H 5.563773 0.797381 2.233219

H 3.866682 0.684148 2.755807

H 6.064594 1.001444 -0.289122

H 4.703435 1.055328 -1.425838

H 5.024875 2.437929 -0.365023

H -4.046682 0.425643 2.198554

H -6.368074 -0.392208 2.481177

H -7.650160 -1.194219 0.518272

H -6.594834 -1.180638 -1.723333

H -4.269603 -0.374180 -1.996297

H -1.005811 4.387295 0.296687

H 0.749257 4.203069 0.430679

H 0.999398 4.797845 -1.990283

H -0.760081 4.981542 -2.126337

H 0.171090 6.088087 -1.106562

H 4.037285 -4.821704 -0.981093

H 4.693277 -3.205159 -0.639226

H 3.893309 -4.129963 0.654339

==============================

conf109_E2.09

C -0.206057 -2.587353 0.178770

C 0.880933 -1.760100 -0.458443

C 0.861492 -0.317067 0.030941

C -0.356244 0.460422 -0.449439

C -1.703305 -0.189027 -0.171480

S -1.875545 -1.953952 -0.178241

C 2.298977 -2.205259 -0.108817

C 3.159652 -0.984076 -0.464653

C 2.200995 0.230610 -0.468052

O 2.788821 1.159544 0.421649

O 4.105233 -0.660497 0.538447

C 4.138466 0.756467 0.638240

C 4.547490 1.139991 2.037280

C 5.041096 1.350083 -0.430504

N -2.711671 0.568817 -0.068754

C -0.332950 1.848829 0.168816

O -0.382460 2.055657 1.352688

C -4.016792 0.060207 0.087977

C -4.736767 -0.386363 -1.018592

C -6.040471 -0.835840 -0.859055

C -6.633199 -0.843596 0.397877

C -5.913647 -0.391955 1.498210

C -4.612244 0.065816 1.347777

O -0.199373 2.800192 -0.748587

C -0.096824 4.151214 -0.256593

C 0.088245 5.055506 -1.446982

O 2.627258 -3.377221 -0.805286

C 3.880139 -3.903481 -0.417269

H -0.216527 -3.608170 -0.202814

H -0.073341 -2.620338 1.260778

H 0.777753 -1.777996 -1.549702

H 0.882436 -0.318392 1.126145

H -0.292965 0.555753 -1.537661

H 2.377174 -2.374459 0.974957

H 3.645086 -1.136379 -1.432798

H 2.097283 0.663304 -1.467603

H 5.563651 0.797695 2.233323

H 3.866525 0.684442 2.755794

H 4.516471 2.223951 2.147411

H 6.064627 1.001840 -0.289012

H 4.703514 1.055590 -1.425791

H 5.024807 2.438246 -0.365000

H -4.269384 -0.374963 -1.996092

H -6.594559 -1.181528 -1.723029

H -7.649987 -1.194631 0.518524

H -6.368059 -0.392018 2.481291

H -4.046718 0.425957 2.198562

H -1.006487 4.387015 0.296884

H 0.748676 4.203316 0.430376

H 0.997944 4.798251 -1.990643

H -0.761629 4.981441 -2.126177

H 0.169519 6.088214 -1.106628

H 3.893576 -4.129778 0.654546

H 4.037702 -4.821495 -0.980883

H 4.693570 -3.204912 -0.638959

==============================

conf110_E2.11

C 1.449460 -2.937616 0.586298

C 1.709672 -1.728865 -0.278393

C 0.894339 -0.533388 0.196195

C -0.602518 -0.647775 -0.091878

C -1.247881 -1.981094 0.271912

S -0.296819 -3.441784 0.560048

C 3.142561 -1.207107 -0.195153

C 3.041309 0.241237 -0.693657

C 1.568641 0.664431 -0.477519

O 1.642173 1.796159 0.369083

O 3.778934 1.151548 0.102156

C 2.968835 2.305471 0.284824

C 3.328921 2.956019 1.595789

C 3.099172 3.244025 -0.903231

N -2.498629 -2.185026 0.308552

C -1.267035 0.494187 0.664568

O -1.424709 0.496826 1.858196

C -3.434285 -1.204168 -0.080039

C -3.562050 -0.840803 -1.420641

C -4.522779 0.085600 -1.801497

C -5.361558 0.658479 -0.853513

C -5.242881 0.284502 0.480930

C -4.292880 -0.649454 0.868152

O -1.557625 1.512123 -0.132024

C -2.081619 2.693356 0.505685

C -2.322869 3.719271 -0.570236

O 4.002094 -2.027937 -0.939007

C 5.360782 -1.670989 -0.784081

H 1.994870 -3.813994 0.236475

H 1.743739 -2.730301 1.615819

H 1.479990 -1.948891 -1.327119

H 1.044825 -0.434895 1.276881

H -0.759064 -0.506353 -1.163991

H 3.461880 -1.179213 0.856951

H 3.341157 0.300207 -1.743804

H 1.073646 0.916861 -1.419463

H 2.674594 3.808591 1.777800

H 4.360194 3.307466 1.564026

H 3.216691 2.235185 2.405297

H 4.127486 3.596488 -0.987658

H 2.826111 2.732485 -1.828014

H 2.439856 4.102310 -0.770968

H -2.906557 -1.290913 -2.156758

H -4.613103 0.361588 -2.844987

H -6.107410 1.383951 -1.152189

H -5.896110 0.720628 1.226724

H -4.195926 -0.945554 1.905235

H -3.000924 2.422673 1.027203

H -1.354153 3.038070 1.241650

H -2.721022 4.628797 -0.119552

H -1.392770 3.966027 -1.083874

H -3.043515 3.351892 -1.301872

H 5.553197 -0.658522 -1.153542

H 5.661046 -1.724163 0.268129

H 5.951826 -2.378353 -1.363357

==============================

conf111_E2.13

C -0.324552 -2.542721 0.291748

C 0.788350 -1.788777 -0.389975

C 0.800021 -0.314329 -0.003124

C -0.389763 0.451821 -0.564935

C -1.753415 -0.134417 -0.233067

S -1.974322 -1.891341 -0.122369

C 2.191857 -2.244074 -0.000403

C 3.083869 -1.077847 -0.448317

C 2.162823 0.165614 -0.509471

O 2.773410 1.105687 0.352771

O 4.064815 -0.726978 0.510351

C 4.121009 0.692979 0.551512

C 4.569613 1.127299 1.923293

C 5.006312 1.227624 -0.562147

N -2.738617 0.657108 -0.169399

C -0.336136 1.892279 -0.081921

O -0.350607 2.206978 1.078707

C -4.054633 0.197299 0.039201

C -4.626349 0.298498 1.305802

C -5.936462 -0.112554 1.506526

C -6.687859 -0.612281 0.449011

C -6.118628 -0.699197 -0.815662

C -4.806537 -0.296837 -1.025072

O -0.234927 2.749104 -1.093421

C -0.107445 4.146153 -0.756627

C 1.319433 4.487140 -0.395886

O 2.489404 -3.475422 -0.601836

C 3.732515 -3.997536 -0.178328

H -0.359384 -3.586461 -0.019922

H -0.201204 -2.505810 1.374768

H 0.692088 -1.879783 -1.478344

H 0.798939 -0.236448 1.089273

H -0.319107 0.441678 -1.656719

H 2.263813 -2.329285 1.093744

H 3.539835 -1.306024 -1.415960

H 2.085739 0.563663 -1.525931

H 4.559146 2.215407 1.988090

H 5.584296 0.775713 2.110241

H 3.898399 0.714284 2.675844

H 6.028237 0.871518 -0.428997

H 4.642466 0.895006 -1.536006

H 5.005663 2.317895 -0.543459

H -4.035746 0.695115 2.122669

H -6.372561 -0.038557 2.495075

H -7.711175 -0.926900 0.608572

H -6.697676 -1.082747 -1.646674

H -4.357140 -0.359481 -2.009032

H -0.427463 4.671901 -1.653777

H -0.795398 4.374270 0.056336

H 1.635516 3.947553 0.496265

H 1.991728 4.235950 -1.217578

H 1.396375 5.558090 -0.202287

H 3.863805 -4.962944 -0.664083

H 4.560308 -3.339743 -0.462528

H 3.749065 -4.133928 0.908581

==============================

conf112_E2.17

C -0.329365 -2.541322 0.296411

C 0.784242 -1.790673 -0.387845

C 0.798078 -0.315499 -0.004254

C -0.391155 0.451876 -0.565579

C -1.755527 -0.132101 -0.233135

S -1.978468 -1.888476 -0.118137

C 2.187435 -2.246597 0.002488

C 3.080829 -1.082071 -0.447400

C 2.160735 0.161737 -0.513244

O 2.772011 1.105148 0.344807

O 4.060402 -0.728920 0.511903

C 4.118688 0.691041 0.547533

C 4.565603 1.130176 1.918344

C 5.006509 1.220173 -0.566754

N -2.739925 0.660673 -0.172445

C -0.333275 1.891985 -0.082121

O -0.352327 2.206519 1.078485

C -4.056652 0.202924 0.036078

C -4.629756 0.308981 1.301674

C -5.940506 -0.100063 1.502265

C -6.691195 -0.602598 0.445566

C -6.120611 -0.694316 -0.818147

C -4.807859 -0.294003 -1.027386

O -0.220983 2.748377 -1.092813

C -0.082846 4.144095 -0.754545

C 1.345359 4.471752 -0.386725

O 2.483915 -3.479332 -0.596609

C 3.726066 -4.002181 -0.171193

H -0.366102 -3.585750 -0.012736

H -0.205182 -2.502031 1.379252

H 0.687344 -1.884186 -1.475936

H 0.798639 -0.235579 1.088016

H -0.320948 0.441630 -1.657385

H 2.259203 -2.329848 1.096792

H 3.538266 -1.313139 -1.413645

H 2.083242 0.555638 -1.531268

H 4.556178 2.218550 1.978859

H 5.579602 0.778242 2.108319

H 3.892705 0.720855 2.671413

H 6.027782 0.863343 -0.430554

H 4.643797 0.884264 -1.539908

H 5.007120 2.310518 -0.552284

H -4.039687 0.707772 2.117868

H -6.377702 -0.022275 2.490038

H -7.715025 -0.915598 0.605011

H -6.699081 -1.080053 -1.648549

H -4.357392 -0.360469 -2.010606

H -0.393871 4.673233 -1.652872

H -0.772628 4.377820 0.055256

H 1.653004 3.926575 0.504979

H 2.018980 4.216828 -1.206187

H 1.430736 5.541475 -0.189880

H 4.554868 -3.345835 -0.455822

H 3.741545 -4.136741 0.915960

H 3.856647 -4.968564 -0.655193

==============================

conf113_E2.17

C 0.218136 2.344638 0.785271

C -0.875682 1.880724 -0.151808

C -0.927084 0.354256 -0.180618

C 0.263507 -0.265348 -0.900197

C 1.629786 0.156397 -0.382967

S 1.876652 1.777348 0.285758

C -2.312274 2.273595 0.262783

C -3.202093 1.153834 -0.285092

C -2.278608 0.051833 -0.825359

O -2.849351 -1.163816 -0.382076

O -3.907565 0.482586 0.745686

C -4.052386 -0.865755 0.332966

C -4.136692 -1.741031 1.558535

C -5.246797 -1.028868 -0.591714

N 2.598448 -0.639801 -0.555500

C 0.169131 -1.782343 -0.907035

O 0.035875 -2.440291 -1.906458

C 3.917212 -0.303320 -0.189504

C 4.701059 0.492376 -1.023170

C 6.015687 0.771606 -0.675486

C 6.556036 0.263074 0.499486

C 5.772861 -0.535032 1.325511

C 4.459781 -0.825337 0.983049

O 0.231709 -2.282792 0.321395

C 0.170395 -3.715772 0.453951

C -1.260305 -4.198432 0.482118

O -2.769798 3.495311 -0.264746

C -2.061470 4.629116 0.188113

H 0.320939 3.427597 0.820306

H 0.022160 1.984904 1.796147

H -0.700086 2.262535 -1.164425

H -0.965443 -0.010657 0.851891

H 0.227805 0.052663 -1.946519

H -2.385854 2.287493 1.358636

H -3.886861 1.552364 -1.036338

H -2.204680 0.062456 -1.916212

H -3.251886 -1.592434 2.177653

H -4.199236 -2.787954 1.261817

H -5.027502 -1.487319 2.133792

H -6.165459 -0.771278 -0.063436

H -5.146807 -0.383976 -1.465997

H -5.311116 -2.063520 -0.929422

H 4.274206 0.883155 -1.939183

H 6.619743 1.390384 -1.327571

H 7.581640 0.482577 0.767024

H 6.186467 -0.938288 2.241592

H 3.844627 -1.452340 1.617119

H 0.732965 -4.166514 -0.362534

H 0.677997 -3.925463 1.393564

H -1.797446 -3.748657 1.317256

H -1.775329 -3.944212 -0.443778

H -1.272184 -5.282646 0.605668

H -1.923538 4.601955 1.274759

H -1.084222 4.714187 -0.296171

H -2.654626 5.504764 -0.072674

==============================

conf114_E2.19

C -0.266746 -2.484227 0.524216

C 0.834106 -1.884246 -0.320803

C 0.932850 -0.381860 -0.071304

C -0.237854 0.399393 -0.648522

C -1.617987 -0.085386 -0.228387

S -1.908092 -1.794015 0.137859

C 2.261512 -2.400905 -0.016945

C 3.179311 -1.197215 -0.251969

C 2.294305 -0.020969 -0.667429

O 2.899325 1.114487 -0.088355

O 3.729687 -0.718520 0.965772

C 3.915511 0.681133 0.826783

C 3.699025 1.331579 2.172287

C 5.276431 0.991045 0.231157

N -2.571054 0.746728 -0.264901

C -0.106984 1.868370 -0.274955

O -0.103582 2.269089 0.859103

C -3.899803 0.371391 0.018308

C -4.443430 0.646921 1.271485

C -5.765266 0.318855 1.537876

C -6.555509 -0.272347 0.558877

C -6.013470 -0.534943 -0.693558

C -4.690630 -0.215745 -0.967721

O 0.021018 2.642053 -1.347998

C 0.191933 4.055639 -1.116766

C 1.629041 4.383193 -0.786561

O 2.687852 -3.446625 -0.857757

C 1.931363 -4.631172 -0.729442

H -0.392342 -3.553809 0.363790

H -0.061586 -2.317251 1.582287

H 0.637216 -2.063809 -1.384442

H 0.984573 -0.207905 1.009547

H -0.208354 0.305827 -1.738199

H 2.331584 -2.712535 1.033619

H 3.962101 -1.441199 -0.973141

H 2.226695 0.107765 -1.750540

H 4.444526 0.974386 2.883387

H 2.702325 1.089682 2.541402

H 3.795000 2.413613 2.079360

H 5.396318 0.479473 -0.724914

H 5.371407 2.064876 0.066973

H 6.064552 0.666258 0.911370

H -3.822255 1.113217 2.026551

H -6.179906 0.529498 2.515980

H -7.587594 -0.521784 0.769140

H -6.622421 -0.991374 -1.464153

H -4.262042 -0.415143 -1.942743

H -0.118373 4.522538 -2.049295

H -0.485053 4.363141 -0.320814

H 1.731294 5.461905 -0.658030

H 1.938677 3.888506 0.133135

H 2.289399 4.067441 -1.595071

H 2.467818 -5.415354 -1.261871

H 1.825941 -4.919979 0.322443

H 0.936698 -4.525732 -1.173094

==============================

conf115_E2.20

C -0.266794 -2.484103 0.524507

C 0.834040 -1.884215 -0.320598

C 0.932861 -0.381822 -0.071175

C -0.237832 0.399402 -0.648454

C -1.617972 -0.085308 -0.228289

S -1.908128 -1.793941 0.138020

C 2.261446 -2.400973 -0.016935

C 3.179271 -1.197307 -0.251921

C 2.294305 -0.021030 -0.667393

O 2.899444 1.114411 -0.088411

O 3.729585 -0.718630 0.965856

C 3.915537 0.680997 0.826807

C 3.699063 1.331492 2.172294

C 5.276512 0.990782 0.231237

N -2.571021 0.746823 -0.264876

C -0.106949 1.868409 -0.275021

O -0.103706 2.269251 0.858991

C -3.899784 0.371473 0.018306

C -4.443450 0.647046 1.271447

C -5.765287 0.318948 1.537799

C -6.555476 -0.272317 0.558794

C -6.013388 -0.534945 -0.693616

C -4.690549 -0.215725 -0.967737

O 0.021230 2.641987 -1.348120

C 0.192133 4.055587 -1.116959

C 1.629198 4.383093 -0.786509

O 2.687619 -3.446577 -0.857996

C 1.931023 -4.631066 -0.729798

H -0.392367 -3.553716 0.364254

H -0.061637 -2.316971 1.582553

H 0.637032 -2.063768 -1.384219

H 0.984619 -0.207786 1.009660

H -0.208318 0.305719 -1.738122

H 2.331648 -2.712797 1.033561

H 3.962099 -1.441285 -0.973049

H 2.226638 0.107672 -1.750503

H 3.795226 2.413509 2.079393

H 4.444456 0.974160 2.883441

H 2.702304 1.089747 2.541351

H 5.371541 2.064599 0.066981

H 6.064588 0.666009 0.911511

H 5.396424 0.479142 -0.724796

H -3.822307 1.113390 2.026511

H -6.179967 0.529615 2.515882

H -7.587564 -0.521772 0.769021

H -6.622307 -0.991431 -1.464205

H -4.261900 -0.415154 -1.942726

H -0.118009 4.522441 -2.049564

H -0.484970 4.363151 -0.321135

H 1.731478 5.461792 -0.657901

H 1.938648 3.888348 0.133223

H 2.289683 4.067345 -1.594918

H 0.936256 -4.525396 -1.173169

H 2.467255 -5.415172 -1.262560

H 1.825824 -4.920131 0.322041

==============================

conf116_E2.25

C -0.267065 -2.483934 0.524589

C 0.833816 -1.884195 -0.320566

C 0.932784 -0.381802 -0.071154

C -0.237858 0.399499 -0.648444

C -1.618025 -0.085087 -0.228255

S -1.908331 -1.793706 0.137974

C 2.261167 -2.401112 -0.016997

C 3.179118 -1.197520 -0.251890

C 2.294281 -0.021133 -0.667337

O 2.899520 1.114224 -0.088268

O 3.729417 -0.718963 0.965936

C 3.915520 0.680661 0.826968

C 3.699086 1.331036 2.172523

C 5.276547 0.990373 0.231477

N -2.571014 0.747115 -0.264826

C -0.106792 1.868523 -0.275092

O -0.103608 2.269437 0.858895

C -3.899796 0.371761 0.018355

C -4.443372 0.647022 1.271600

C -5.765209 0.318898 1.537936

C -6.555459 -0.272089 0.558820

C -6.013446 -0.534432 -0.693684

C -4.690614 -0.215178 -0.967796

O 0.021703 2.641981 -1.348231

C 0.192992 4.055563 -1.117199

C 1.630099 4.382675 -0.786537

O 2.687188 -3.446673 -0.858211

C 1.930361 -4.631033 -0.730207

H -0.392690 -3.553566 0.364477

H -0.061920 -2.316691 1.582623

H 0.636729 -2.063720 -1.384175

H 0.984543 -0.207750 1.009677

H -0.208369 0.305782 -1.738112

H 2.331408 -2.713079 1.033453

H 3.961958 -1.441529 -0.973001

H 2.226672 0.107652 -1.750439

H 2.702310 1.089316 2.541540

H 3.795370 2.413052 2.079792

H 4.444440 0.973490 2.883603

H 6.064571 0.665521 0.911767

H 5.396468 0.478782 -0.724577

H 5.371618 2.064193 0.067283

H -3.822161 1.113137 2.026746

H -6.179827 0.529310 2.516098

H -7.587544 -0.521569 0.769032

H -6.622427 -0.990713 -1.464343

H -4.262001 -0.414394 -1.942842

H -0.116842 4.522394 -2.049919

H -0.484156 4.363427 -0.321527

H 2.290621 4.066728 -1.594834

H 1.732638 5.461346 -0.657921

H 1.939267 3.887840 0.133240

H 2.466325 -5.415103 -1.263291

H 1.825308 -4.920378 0.321572

H 0.935521 -4.525011 -1.173331

==============================

conf117_E2.27

C 1.415831 -2.922915 0.586738

C 1.748084 -1.729826 -0.274048

C 0.936021 -0.508973 0.139021

C -0.551273 -0.599723 -0.223944

C -1.245976 -1.872886 0.242448

S -0.339912 -3.374077 0.487448

C 3.187101 -1.241593 -0.122237

C 3.147478 0.202383 -0.640175

C 1.674336 0.659697 -0.520111

O 1.722642 1.808858 0.304895

O 3.854729 1.105223 0.190944

C 3.065229 2.282100 0.300887

C 3.355426 2.946771 1.622147

C 3.298112 3.196166 -0.890697

N -2.493960 -2.035417 0.388270

C -1.186401 0.645753 0.372905

O -1.502269 0.748018 1.529730

C -3.479923 -1.106522 0.015523

C -3.578507 -0.629105 -1.291829

C -4.645633 0.181785 -1.659169

C -5.613503 0.535646 -0.729746

C -5.511924 0.065301 0.576569

C -4.461959 -0.759364 0.946127

O -1.222841 1.634866 -0.511760

C -1.607445 2.934069 -0.019110

C -3.110613 3.073050 0.016485

O 4.062383 -2.091619 -0.813424

C 5.419820 -1.763556 -0.596419

H 1.950959 -3.817303 0.267578

H 1.670546 -2.716090 1.627003

H 1.568244 -1.956917 -1.330989

H 1.030336 -0.391688 1.224593

H -0.635346 -0.562675 -1.313038

H 3.453846 -1.209878 0.944305

H 3.512669 0.243619 -1.670251

H 1.239975 0.899816 -1.493948

H 4.395053 3.272790 1.652791

H 3.172490 2.243307 2.434036

H 2.712159 3.817855 1.746962

H 4.338821 3.520532 -0.913364

H 3.071909 2.676071 -1.823317

H 2.655103 4.073516 -0.815547

H -2.836431 -0.921611 -2.024783

H -4.716527 0.536528 -2.680173

H -6.442373 1.169272 -1.018471

H -6.262481 0.334314 1.309596

H -4.388180 -1.146306 1.955027

H -1.164768 3.076597 0.965565

H -1.156711 3.635484 -0.718425

H -3.531284 2.958977 -0.983026

H -3.552538 2.323267 0.674129

H -3.374372 4.063332 0.391369

H 5.653712 -0.761170 -0.969521

H 5.666840 -1.808294 0.469924

H 6.021820 -2.492500 -1.136351

==============================

conf118_E2.28

C -0.006220 -2.549828 0.073153

C 1.022236 -1.670557 -0.590044

C 0.939748 -0.228746 -0.105043

C -0.324558 0.483871 -0.562901

C -1.627195 -0.229031 -0.229668

S -1.713059 -2.001318 -0.249539

C 2.469945 -2.037845 -0.277555

C 3.253171 -0.781476 -0.699679

C 2.241868 0.384522 -0.643435

O 2.856816 1.358859 0.168514

O 4.304803 -0.386681 0.158838

C 3.914298 0.759608 0.910425

C 3.454375 0.359069 2.303161

C 5.073471 1.728492 0.956600

N -2.662113 0.480142 -0.063238

C -0.361040 1.878291 0.041247

O -0.324924 2.094234 1.224198

C -3.933164 -0.088226 0.155577

C -4.479567 -0.073236 1.437330

C -5.748445 -0.591954 1.652987

C -6.485528 -1.112660 0.595563

C -5.943402 -1.112415 -0.683990

C -4.671512 -0.603284 -0.908323

O -0.396332 2.826378 -0.888015

C -0.394219 4.186748 -0.410927

C -0.423835 5.091386 -1.614967

O 2.834996 -3.206233 -0.963210

C 4.131404 -3.652965 -0.621641

H 0.025461 -3.571405 -0.305429

H 0.151045 -2.572115 1.152291

H 0.893238 -1.697166 -1.678641

H 0.967898 -0.225354 0.988111

H -0.299584 0.568222 -1.653628

H 2.592481 -2.185105 0.805299

H 3.661321 -0.935362 -1.700834

H 2.068681 0.824835 -1.627720

H 3.094158 1.238793 2.837980

H 4.292526 -0.068984 2.854450

H 2.655077 -0.379237 2.262373

H 5.366769 1.996254 -0.058153

H 4.783595 2.629161 1.498440

H 5.919835 1.270041 1.468352

H -3.901195 0.341427 2.253922

H -6.163913 -0.585420 2.653114

H -7.477395 -1.510828 0.766725

H -6.512226 -1.511132 -1.514901

H -4.244111 -0.597602 -1.904039

H -1.266890 4.329176 0.227466

H 0.502450 4.338759 0.191113

H 0.451775 4.927895 -2.244049

H -1.322101 4.917672 -2.208262

H -0.423136 6.130734 -1.285347

H 4.308295 -4.582921 -1.159468

H 4.895744 -2.923850 -0.909192

H 4.210068 -3.837424 0.455496

==============================

conf119_E2.28

C -0.218123 2.344466 -0.785534

C 0.875566 1.880665 0.151758

C 0.927086 0.354194 0.180610

C -0.263476 -0.265399 0.900242

C -1.629758 0.156277 0.382992

S -1.876665 1.776896 -0.286434

C 2.312183 2.273702 -0.262598

C 3.202032 1.153927 0.285197

C 2.278602 0.051883 0.825430

O 2.849469 -1.163727 0.382269

O 3.907424 0.482749 -0.745698

C 4.052332 -0.865592 -0.333114

C 4.136348 -1.740769 -1.558775

C 5.246936 -1.028781 0.591299

N -2.598472 -0.639775 0.555922

C -0.169029 -1.782388 0.907123

O -0.035503 -2.440303 1.906526

C -3.917189 -0.303322 0.189720

C -4.701268 0.491983 1.023533

C -6.015850 0.771225 0.675693

C -6.555922 0.263071 -0.499566

C -5.772505 -0.534635 -1.325745

C -4.459456 -0.824919 -0.983155

O -0.231874 -2.282875 -0.321290

C -0.170237 -3.715848 -0.453815

C 1.260579 -4.198137 -0.482175

O 2.769546 3.495370 0.265155

C 2.061113 4.629167 -0.187568

H -0.321056 3.427415 -0.820538

H -0.021862 1.984799 -1.796380

H 0.699747 2.262454 1.164347

H 0.965447 -0.010790 -0.851870

H -0.227767 0.052655 1.946551

H 2.385906 2.287746 -1.358441

H 3.886871 1.552396 1.036413

H 2.204591 0.062541 1.916280

H 4.199176 -2.787708 -1.262184

H 5.026928 -1.486855 -2.134304

H 3.251330 -1.592258 -2.177604

H 6.165512 -0.771278 0.062831

H 5.147156 -0.383846 1.465575

H 5.311235 -2.063424 0.929028

H -4.274604 0.882480 1.939753

H -6.620086 1.389714 1.327884

H -7.581497 0.482564 -0.767218

H -6.185892 -0.937581 -2.242061

H -3.844103 -1.451584 -1.617364

H -0.732585 -4.166692 0.362766

H -0.677916 -3.925701 -1.393354

H 1.272719 -5.282372 -0.605496

H 1.797417 -3.748395 -1.317526

H 1.775750 -3.943560 0.443539

H 1.923488 4.602279 -1.274261

H 1.083724 4.713914 0.296477

H 2.654044 5.504842 0.073631

==============================

conf120_E2.29

C -0.218212 2.344299 -0.785790

C 0.875437 1.880676 0.151637

C 0.927082 0.354215 0.180621

C -0.263474 -0.265420 0.900230

C -1.629769 0.156165 0.382933

S -1.876741 1.776653 -0.286747

C 2.312060 2.273835 -0.262597

C 3.201962 1.154073 0.285141

C 2.278594 0.052065 0.825530

O 2.849543 -1.163583 0.382560

O 3.907169 0.482826 -0.745837

C 4.052221 -0.865474 -0.333155

C 4.135986 -1.740747 -1.558766

C 5.247048 -1.028555 0.590990

N -2.598465 -0.639885 0.556004

C -0.168888 -1.782405 0.907171

O -0.035296 -2.440259 1.906606

C -3.917191 -0.303460 0.189784

C -4.459445 -0.825035 -0.983105

C -5.772494 -0.534744 -1.325700

C -6.555914 0.262955 -0.499519

C -6.015850 0.771095 0.675750

C -4.701272 0.491848 1.023597

O -0.231690 -2.282953 -0.321214

C -0.169748 -3.715920 -0.453680

C 1.261193 -4.197847 -0.482187

O 2.769307 3.495481 0.265339

C 2.060751 4.629261 -0.187226

H -0.321218 3.427238 -0.820913

H -0.021846 1.984541 -1.796584

H 0.699485 2.262529 1.164177

H 0.965542 -0.010847 -0.851828

H -0.227825 0.052669 1.946530

H 2.385876 2.288026 -1.358430

H 3.886929 1.552560 1.036230

H 2.204548 0.062861 1.916374

H 3.250865 -1.592240 -2.177449

H 4.198810 -2.787666 -1.262105

H 5.026476 -1.486907 -2.134465

H 5.311453 -2.063174 0.928768

H 6.165491 -0.771057 0.062290

H 5.147450 -0.383570 1.465248

H -3.844087 -1.451682 -1.617326

H -6.185876 -0.937678 -2.242024

H -7.581486 0.482456 -0.767176

H -6.620092 1.389580 1.327939

H -4.274603 0.882349 1.939812

H -0.731883 -4.166859 0.362995

H -0.677501 -3.925923 -1.393145

H 1.797787 -3.748091 -1.317687

H 1.776417 -3.942933 0.443407

H 1.273625 -5.282098 -0.605312

H 2.653547 5.504970 0.074164

H 1.923196 4.602550 -1.273932

H 1.083316 4.713794 0.296771

==============================

conf121_E2.30

C -0.218275 2.344304 -0.785740

C 0.875403 1.880690 0.151654

C 0.927072 0.354232 0.180645

C -0.263470 -0.265398 0.900294

C -1.629768 0.156143 0.382948

S -1.876780 1.776644 -0.286655

C 2.312004 2.273905 -0.262579

C 3.201947 1.154101 0.285016

C 2.278612 0.052115 0.825505

O 2.849548 -1.163557 0.382541

O 3.906971 0.482841 -0.746066

C 4.052095 -0.865467 -0.333377

C 4.135672 -1.740729 -1.559010

C 5.247078 -1.028529 0.590564

N -2.598455 -0.639926 0.556039

C -0.168811 -1.782379 0.907333

O -0.035111 -2.440133 1.906827

C -3.917169 -0.303518 0.189742

C -4.459319 -0.825065 -0.983210

C -5.772349 -0.534800 -1.325901

C -6.555855 0.262855 -0.499754

C -6.015896 0.770969 0.675573

C -4.701337 0.491734 1.023521

O -0.231637 -2.283037 -0.320990

C -0.169553 -3.716015 -0.453367

C 1.261449 -4.197760 -0.481933

O 2.769217 3.495511 0.265527

C 2.060655 4.629320 -0.186950

H -0.321279 3.427246 -0.820849

H -0.021935 1.984563 -1.796544

H 0.699464 2.262539 1.164197

H 0.965504 -0.010843 -0.851801

H -0.227815 0.052770 1.946568

H 2.385818 2.288273 -1.358406

H 3.887030 1.552552 1.036018

H 2.204634 0.062930 1.916350

H 4.198629 -2.787650 -1.262382

H 5.026032 -1.486803 -2.134874

H 3.250417 -1.592273 -2.177514

H 5.311510 -2.063140 0.928366

H 6.165436 -0.771078 0.061693

H 5.147652 -0.383511 1.464816

H -3.843896 -1.451682 -1.617399

H -6.185649 -0.937718 -2.242269

H -7.581411 0.482342 -0.767487

H -6.620198 1.389432 1.327729

H -4.274760 0.882205 1.939792

H -0.731572 -4.166960 0.363381

H -0.677351 -3.926134 -1.392778

H 1.776692 -3.942671 0.443606

H 1.274025 -5.282019 -0.604959

H 1.797929 -3.747986 -1.317495

H 1.083236 4.713841 0.297089

H 2.653461 5.505020 0.074454

H 1.923049 4.602667 -1.273651

==============================

conf122_E2.31

C -0.006346 -2.549814 0.072902

C 1.022148 -1.670517 -0.590199

C 0.939707 -0.228739 -0.105091

C -0.324565 0.483959 -0.562916

C -1.627224 -0.228913 -0.229716

S -1.713168 -2.001189 -0.249709

C 2.469835 -2.037876 -0.277691

C 3.253116 -0.781511 -0.699727

C 2.241860 0.384522 -0.643409

O 2.856819 1.358766 0.168646

O 4.304752 -0.386816 0.158824

C 3.914280 0.759418 0.910509

C 3.454323 0.358768 2.303201

C 5.073482 1.728267 0.956782

N -2.662126 0.480269 -0.063231

C -0.360996 1.878346 0.041303

O -0.324864 2.094225 1.224265

C -3.933161 -0.088125 0.155612

C -4.671850 -0.602424 -0.908424

C -5.943707 -1.111616 -0.684068

C -6.485471 -1.112676 0.595641

C -5.748046 -0.592737 1.653199

C -4.479188 -0.073965 1.437528

O -0.396269 2.826477 -0.887916

C -0.394046 4.186828 -0.410773

C -0.423334 5.091522 -1.614779

O 2.834865 -3.206243 -0.963392

C 4.131243 -3.653046 -0.621798

H 0.025290 -3.571349 -0.305796

H 0.150917 -2.572230 1.152037

H 0.893181 -1.697038 -1.678802

H 0.967844 -0.225432 0.988065

H -0.299596 0.568371 -1.653637

H 2.592330 -2.185196 0.805159

H 3.661262 -0.935345 -1.700892

H 2.068714 0.824925 -1.627660

H 2.654992 -0.379500 2.262327

H 3.094134 1.238458 2.838097

H 4.292448 -0.069372 2.854461

H 4.783622 2.628903 1.498687

H 5.919822 1.269751 1.468514

H 5.366807 1.996099 -0.057945

H -4.244721 -0.596110 -1.904252

H -6.512795 -1.509748 -1.515080

H -7.477317 -1.510891 0.766817

H -6.163219 -0.586843 2.653453

H -3.900551 0.340090 2.254242

H -1.266811 4.329344 0.227475

H 0.502536 4.338696 0.191430

H 0.452338 4.927896 -2.243741

H -1.321545 4.918003 -2.208213

H -0.422487 6.130855 -1.285113

H 4.209918 -3.837372 0.455361

H 4.308037 -4.583089 -1.159506

H 4.895640 -2.924036 -0.909464

==============================

conf123_E2.35

C -0.218310 2.344813 -0.784866

C 0.875609 1.880759 0.152025

C 0.927022 0.354276 0.180637

C -0.263467 -0.265342 0.900380

C -1.629773 0.156334 0.383191

S -1.876758 1.777295 -0.285417

C 2.312144 2.273745 -0.262614

C 3.202053 1.153863 0.284883

C 2.278651 0.051790 0.825144

O 2.849354 -1.163789 0.381606

O 3.907284 0.482798 -0.746153

C 4.052181 -0.865631 -0.333707

C 4.136210 -1.740626 -1.559504

C 5.246838 -1.028943 0.590632

N -2.598419 -0.639899 0.555633

C -0.169030 -1.782345 0.907317

O -0.035886 -2.440203 1.906817

C -3.917104 -0.303444 0.189330

C -4.701437 0.491422 1.023309

C -6.015989 0.770663 0.675314

C -6.555763 0.262936 -0.500258

C -5.772081 -0.534334 -1.326621

C -4.459074 -0.824609 -0.983889

O -0.231500 -2.282901 -0.321056

C -0.170089 -3.715905 -0.453433

C 1.260608 -4.198639 -0.480629

O 2.769730 3.495333 0.265201

C 2.061108 4.629216 -0.186978

H -0.321172 3.427776 -0.819633

H -0.022380 1.985340 -1.795845

H 0.700098 2.262389 1.164727

H 0.965185 -0.010552 -0.851906

H -0.227687 0.052743 1.946676

H 2.385658 2.287947 -1.358466

H 3.886975 1.552248 1.036070

H 2.204884 0.062238 1.916009

H 5.026724 -1.486487 -2.135030

H 3.251131 -1.592117 -2.178249

H 4.199179 -2.787602 -1.263077

H 5.311131 -2.063643 0.928187

H 6.165380 -0.771366 0.062146

H 5.147123 -0.384153 1.465021

H -4.274998 0.881599 1.939771

H -6.620422 1.388818 1.327639

H -7.581309 0.482423 -0.768029

H -6.185242 -0.936932 -2.243193

H -3.843510 -1.450931 -1.618231

H -0.733235 -4.166536 0.362721

H -0.677063 -3.925654 -1.393371

H 1.775051 -3.944411 0.445581

H 1.272479 -5.282851 -0.604158

H 1.798303 -3.748899 -1.315425

H 2.654161 5.504844 0.074110

H 1.922991 4.602563 -1.273616

H 1.083931 4.713879 0.297520

==============================

conf124_E2.35

C -0.266883 -2.483903 0.524878

C 0.833908 -1.884183 -0.320401

C 0.932778 -0.381761 -0.071134

C -0.237923 0.399444 -0.648396

C -1.618092 -0.085291 -0.228252

S -1.908228 -1.793865 0.138265

C 2.261300 -2.400951 -0.016768

C 3.179173 -1.197367 -0.251904

C 2.294257 -0.020993 -0.667294

O 2.899296 1.114384 -0.088011

O 3.729725 -0.718728 0.965791

C 3.915989 0.680813 0.826468

C 3.700644 1.331464 2.172054

C 5.276623 0.990244 0.229913

N -2.571145 0.746816 -0.264998

C -0.107056 1.868454 -0.274916

O -0.103944 2.269280 0.859102

C -3.899921 0.371504 0.018139

C -4.443859 0.647937 1.270971

C -5.765710 0.319891 1.537325

C -6.555653 -0.272169 0.558603

C -6.013301 -0.535654 -0.693507

C -4.690438 -0.216510 -0.967618

O 0.021323 2.642034 -1.347982

C 0.192314 4.055625 -1.116794

C 1.629385 4.382967 -0.786207

O 2.687374 -3.446648 -0.857797

C 1.930657 -4.631051 -0.729508

H -0.392419 -3.553568 0.364907

H -0.061695 -2.316513 1.582881

H 0.636814 -2.063858 -1.383982

H 0.984581 -0.207652 1.009686

H -0.208437 0.305821 -1.738069

H 2.331585 -2.712723 1.033737

H 3.961874 -1.441435 -0.973140

H 2.226704 0.107854 -1.750394

H 2.704033 1.090097 2.541751

H 3.797181 2.413437 2.079085

H 4.446370 0.973819 2.882693

H 5.371713 2.064033 0.065536

H 6.065115 0.665366 0.909651

H 5.395818 0.478559 -0.726181

H -3.822916 1.114889 2.025822

H -6.180591 0.531233 2.515174

H -7.587753 -0.521571 0.768826

H -6.622017 -0.992756 -1.463890

H -4.261599 -0.416641 -1.942376

H -0.117713 4.522509 -2.049424

H -0.484820 4.363235 -0.321012

H 2.289902 4.067254 -1.594599

H 1.731750 5.461633 -0.657425

H 1.938702 3.888052 0.133481

H 2.466783 -5.415234 -1.262262

H 1.825494 -4.920071 0.322347

H 0.935866 -4.525280 -1.172804

==============================

conf125_E2.37

C -0.330058 -2.541091 0.297227

C 0.783637 -1.790950 -0.387445

C 0.797758 -0.315675 -0.004329

C -0.391383 0.451846 -0.565644

C -1.755880 -0.131802 -0.233193

S -1.979079 -1.888124 -0.117532

C 2.186816 -2.246944 0.002894

C 3.080343 -1.082625 -0.447344

C 2.160391 0.161215 -0.513680

O 2.771802 1.105015 0.343857

O 4.059890 -0.729211 0.511903

C 4.118372 0.690784 0.546918

C 4.565229 1.130461 1.917573

C 5.006401 1.219289 -0.567507

N -2.740149 0.661168 -0.173095

C -0.332941 1.891888 -0.082104

O -0.352793 2.206378 1.078501

C -4.056997 0.203752 0.035522

C -4.808363 -0.292994 -1.027906

C -6.121200 -0.692985 -0.818598

C -6.691710 -0.601080 0.445139

C -5.940860 -0.098706 1.501793

C -4.630008 0.309992 1.301138

O -0.218878 2.748226 -1.092643

C -0.079138 4.143718 -0.754092

C 1.349266 4.469338 -0.385205

O 2.483129 -3.479834 -0.595965

C 3.725177 -4.002796 -0.170393

H -0.367018 -3.585662 -0.011415

H -0.205815 -2.501322 1.380044

H 0.686612 -1.884812 -1.475493

H 0.798507 -0.235444 1.087920

H -0.321182 0.441617 -1.657450

H 2.258678 -2.329975 1.097207

H 3.537832 -1.314108 -1.413466

H 2.082792 0.554672 -1.531866

H 5.579209 0.778581 2.107747

H 3.892277 0.721486 2.670780

H 4.555828 2.218863 1.977621

H 5.007159 2.309641 -0.553508

H 6.027609 0.862378 -0.431061

H 4.643732 0.883017 -1.540550

H -4.357950 -0.359580 -2.011142

H -6.699810 -1.078611 -1.648952

H -7.715602 -0.913850 0.604637

H -6.377996 -0.020771 2.489583

H -4.039820 0.708679 2.117295

H -0.388823 4.673391 -1.652568

H -0.769172 4.378261 0.055258

H 1.435888 5.538806 -0.187527

H 1.655698 3.923059 0.506243

H 2.023052 4.214133 -1.204446

H 3.855572 -4.969348 -0.654105

H 4.554100 -3.346686 -0.455219

H 3.740640 -4.137028 0.916801

==============================

conf126_E2.38

C 0.075938 -2.504252 0.518256

C 1.111204 -1.806723 -0.335237

C 1.076798 -0.302531 -0.082674

C -0.165930 0.373700 -0.641007

C -1.492965 -0.235742 -0.214184

S -1.624941 -1.964217 0.147431

C 2.582391 -2.190793 -0.042714

C 3.385247 -0.909118 -0.288472

C 2.392476 0.181607 -0.693698

O 2.897131 1.368219 -0.120183

O 3.903963 -0.380988 0.922813

C 3.961156 1.029404 0.780944

C 3.704945 1.660450 2.128574

C 5.280356 1.459008 0.165852

N -2.516582 0.508425 -0.242412

C -0.159690 1.842527 -0.247410

O -0.192421 2.225453 0.892352

C -3.804708 0.016923 0.050463

C -4.553333 -0.625480 -0.933982

C -5.840460 -1.060574 -0.649483

C -6.388493 -0.859245 0.611652

C -5.640946 -0.212371 1.589020

C -4.355488 0.231400 1.312488

O -0.075247 2.639083 -1.307856

C -0.050482 4.058177 -1.053461

C -1.447830 4.588733 -0.832830

O 3.095617 -3.196967 -0.882874

C 2.449133 -4.444109 -0.746428

H 0.045580 -3.580733 0.357159

H 0.274143 -2.320133 1.574853

H 0.922821 -2.002336 -1.397579

H 1.126370 -0.127453 0.998137

H -0.138838 0.296251 -1.731898

H 2.688496 -2.490215 1.008422

H 4.178785 -1.082056 -1.018417

H 2.301448 0.303349 -1.775796

H 4.495896 1.385068 2.826749

H 2.745094 1.319888 2.516974

H 3.687571 2.746066 2.029815

H 5.430067 0.960816 -0.793076

H 5.278212 2.537089 0.002644

H 6.104220 1.204150 0.833367

H -4.120520 -0.776633 -1.915766

H -6.416639 -1.559662 -1.418829

H -7.392693 -1.199208 0.829938

H -6.060835 -0.048646 2.573838

H -3.768297 0.741841 2.066258

H 0.595308 4.248490 -0.197058

H 0.401956 4.487465 -1.944896

H -2.076651 4.380060 -1.699224

H -1.901284 4.137915 0.049915

H -1.406972 5.669281 -0.688040

H 1.450132 -4.432532 -1.192689

H 3.055393 -5.180464 -1.272281

H 2.368114 -4.733491 0.307424

==============================

conf127_E2.41

C 0.075306 -2.504425 0.517590

C 1.110761 -1.806797 -0.335586

C 1.076450 -0.302667 -0.082708

C -0.166177 0.373862 -0.640982

C -1.493297 -0.235468 -0.214365

S -1.625491 -1.964010 0.146929

C 2.581880 -2.190976 -0.042759

C 3.384894 -0.909419 -0.288580

C 2.392201 0.181532 -0.693483

O 2.896819 1.367936 -0.119466

O 3.904130 -0.381508 0.922563

C 3.961569 1.028869 0.780688

C 3.706621 1.659938 2.128532

C 5.280359 1.458173 0.164501

N -2.516848 0.508795 -0.242519

C -0.159608 1.842585 -0.247046

O -0.192633 2.225268 0.892784

C -3.804980 0.017307 0.050365

C -4.355268 0.230656 1.312799

C -5.640723 -0.213112 1.589340

C -6.388752 -0.858883 0.611608

C -5.841211 -1.059075 -0.649917

C -4.554099 -0.623941 -0.934447

O -0.074517 2.639347 -1.307309

C -0.049007 4.058368 -1.052572

C -1.446100 4.589725 -0.832294

O 3.095176 -3.197364 -0.882602

C 2.448903 -4.444575 -0.745681

H 0.044747 -3.580837 0.356103

H 0.273503 -2.320717 1.574263

H 0.922622 -2.002226 -1.398006

H 1.125940 -0.127842 0.998155

H -0.138999 0.296634 -1.731884

H 2.687719 -2.490189 1.008463

H 4.178147 -1.082466 -1.018808

H 2.301291 0.303604 -1.775557

H 2.746940 1.319701 2.517636

H 3.689574 2.745567 2.029867

H 4.498007 1.384228 2.826085

H 5.429160 0.960021 -0.794590

H 5.278356 2.536265 0.001373

H 6.104731 1.203064 0.831297

H -3.767734 0.740259 2.066870

H -6.060214 -0.050272 2.574475

H -7.392942 -1.198854 0.829925

H -6.417763 -1.557247 -1.419577

H -4.121658 -0.774202 -1.916532

H 0.596613 4.248082 -0.195909

H 0.403999 4.487593 -1.943751

H -2.074783 4.381548 -1.698908

H -1.900093 4.139080 0.050259

H -1.404637 5.670234 -0.687371

H 2.367403 -4.733288 0.308314

H 1.450132 -4.433490 -1.192468

H 3.055594 -5.181145 -1.270736

==============================

conf128_E2.44

C 0.143775 -2.407921 0.549967

C 1.162748 -1.716848 -0.320232

C 1.006362 -0.201114 -0.290431

C -0.261338 0.269286 -0.989760

C -1.544869 -0.341190 -0.450128

S -1.574579 -2.039868 0.063970

C 2.610426 -1.907370 0.123410

C 3.362244 -0.791388 -0.625598

C 2.308162 0.293051 -0.938494

O 2.846628 1.485694 -0.413219

O 4.364494 -0.116479 0.108457

C 3.898550 1.173537 0.494491

C 3.401788 1.159215 1.931669

C 5.014078 2.173547 0.293952

N -2.601188 0.353710 -0.497736

C -0.369513 1.783456 -0.963634

O -0.416963 2.471292 -1.950621

C -3.852409 -0.159107 -0.099600

C -4.358681 0.157411 1.159421

C -5.610083 -0.308608 1.536811

C -6.368092 -1.078948 0.662390

C -5.864549 -1.382667 -0.596620

C -4.611159 -0.926141 -0.981318

O -0.362955 2.250974 0.279615

C -0.515780 3.672932 0.447350

C -1.970225 4.069937 0.346139

O 3.045935 -3.205580 -0.182386

C 4.346331 -3.470399 0.303112

H 0.218613 -3.493066 0.481212

H 0.273602 -2.114511 1.592516

H 1.076133 -2.070783 -1.354370

H 0.981091 0.127834 0.751716

H -0.203748 -0.023262 -2.042591

H 2.696878 -1.728587 1.205049

H 3.815028 -1.209483 -1.526992

H 2.165367 0.430271 -2.012455

H 2.975079 2.131302 2.182403

H 4.236928 0.957947 2.603782

H 2.644462 0.391924 2.085074

H 4.666803 3.174203 0.552753

H 5.859085 1.920482 0.934469

H 5.333485 2.160603 -0.747763

H -3.763260 0.761596 1.833089

H -5.995178 -0.065492 2.519423

H -7.345959 -1.435991 0.958892

H -6.449246 -1.979095 -1.286072

H -4.213004 -1.156422 -1.962390

H -0.115695 3.876146 1.438639

H 0.096192 4.182716 -0.295625

H -2.369175 3.840877 -0.642100

H -2.562893 3.545353 1.096647

H -2.066425 5.142943 0.518561

H 4.576612 -4.508582 0.069436

H 5.091074 -2.824318 -0.172622

H 4.396017 -3.323928 1.387795

==============================

conf129_E2.46

C 0.075746 -2.504121 0.518395

C 1.111044 -1.806698 -0.335154

C 1.076733 -0.302491 -0.082573

C -0.165971 0.373812 -0.640857

C -1.493053 -0.235611 -0.214129

S -1.625097 -1.964076 0.147502

C 2.582186 -2.190837 -0.042646

C 3.385152 -0.909254 -0.288560

C 2.392424 0.181611 -0.693583

O 2.897111 1.368113 -0.119840

O 3.904279 -0.381195 0.922571

C 3.961694 1.029168 0.780560

C 3.706486 1.660392 2.128292

C 5.280589 1.458448 0.164569

N -2.516667 0.508555 -0.242513

C -0.159727 1.842607 -0.247119

O -0.192568 2.225407 0.892686

C -3.804805 0.017043 0.050325

C -4.355358 0.230883 1.312553

C -5.640809 -0.212927 1.589053

C -6.388555 -0.859233 0.611465

C -5.840740 -1.059937 -0.649863

C -4.553631 -0.624777 -0.934344

O -0.075142 2.639264 -1.307463

C -0.050524 4.058345 -1.052928

C -1.447924 4.588758 -0.832315

O 3.095354 -3.197138 -0.882704

C 2.448575 -4.444130 -0.746404

H 0.045370 -3.580622 0.357435

H 0.273958 -2.319894 1.574975

H 0.922592 -2.002281 -1.397490

H 1.126353 -0.127447 0.998241

H -0.138871 0.296459 -1.731755

H 2.688295 -2.490150 1.008524

H 4.178466 -1.082327 -1.018715

H 2.301398 0.303502 -1.775666

H 3.689258 2.745999 2.029434

H 4.497833 1.384926 2.825985

H 2.746818 1.320049 2.517334

H 5.429542 0.960242 -0.794469

H 5.278591 2.536531 0.001372

H 6.104845 1.203387 0.831521

H -3.767993 0.740859 2.066503

H -6.060523 -0.049690 2.574026

H -7.392743 -1.199244 0.829731

H -6.417072 -1.558597 -1.419370

H -4.120974 -0.775467 -1.916267

H 0.595215 4.248631 -0.196481

H 0.401911 4.487759 -1.944305

H -1.407143 5.669293 -0.687402

H -2.076676 4.380147 -1.698773

H -1.901406 4.137814 0.050349

H 2.367572 -4.733676 0.307407

H 1.449540 -4.432251 -1.192583

H 3.054610 -5.180544 -1.272433

==============================

conf130_E2.47

C -0.266849 -2.483937 0.524838

C 0.833919 -1.884201 -0.320459

C 0.932839 -0.381787 -0.071147

C -0.237847 0.399428 -0.648441

C -1.618002 -0.085229 -0.228225

S -1.908189 -1.793760 0.138431

C 2.261306 -2.401053 -0.016945

C 3.179203 -1.197435 -0.251879

C 2.294319 -0.021087 -0.667356

O 2.899498 1.114299 -0.088280

O 3.729496 -0.718821 0.965926

C 3.915593 0.680793 0.826861

C 3.699295 1.331262 2.172392

C 5.276575 0.990460 0.231224

N -2.571036 0.746917 -0.264980

C -0.106917 1.868469 -0.275082

O -0.103732 2.269373 0.858911

C -3.899817 0.371579 0.018218

C -4.443579 0.647499 1.271239

C -5.765420 0.319416 1.537598

C -6.555505 -0.272172 0.558708

C -6.013318 -0.535154 -0.693584

C -4.690473 -0.215961 -0.967706

O 0.021443 2.641956 -1.348204

C 0.192561 4.055554 -1.117144

C 1.629657 4.382859 -0.786611

O 2.687357 -3.446592 -0.858169

C 1.930511 -4.630947 -0.730249

H -0.392465 -3.553574 0.364753

H -0.061574 -2.316670 1.582842

H 0.636748 -2.063803 -1.384039

H 0.984622 -0.207677 1.009672

H -0.208375 0.305724 -1.738108

H 2.331599 -2.713011 1.033506

H 3.962043 -1.441444 -0.972989

H 2.226711 0.107678 -1.750460

H 4.444689 0.973743 2.883442

H 2.702530 1.089623 2.541496

H 3.795612 2.413268 2.079561

H 5.371629 2.064267 0.066939

H 6.064661 0.665658 0.911466

H 5.396419 0.478800 -0.724804

H -3.822508 1.114099 2.026201

H -6.180178 0.530341 2.515590

H -7.587598 -0.521612 0.768930

H -6.622171 -0.991893 -1.464074

H -4.261738 -0.415680 -1.942596

H -0.117420 4.522373 -2.049821

H -0.484554 4.363292 -0.321396

H 1.732095 5.461559 -0.658157

H 1.938940 3.888192 0.133216

H 2.290150 4.066872 -1.594915

H 1.825518 -4.920406 0.321505

H 0.935649 -4.524866 -1.173307

H 2.466432 -5.414962 -1.263457

==============================

conf131_E2.48

C -0.266949 -2.483953 0.524682

C 0.833883 -1.884194 -0.320520

C 0.932779 -0.381787 -0.071165

C -0.237905 0.399467 -0.648413

C -1.618059 -0.085211 -0.228220

S -1.908264 -1.793788 0.138151

C 2.261250 -2.401006 -0.016855

C 3.179160 -1.197423 -0.251920

C 2.294266 -0.021042 -0.667325

O 2.899381 1.114320 -0.088120

O 3.729656 -0.718812 0.965797

C 3.915816 0.680773 0.826625

C 3.700051 1.331303 2.172209

C 5.276604 0.990340 0.230483

N -2.571081 0.746956 -0.264878

C -0.106966 1.868484 -0.274954

O -0.103764 2.269311 0.859067

C -3.899872 0.371604 0.018244

C -4.443688 0.647527 1.271242

C -5.765532 0.319423 1.537557

C -6.555571 -0.272191 0.558646

C -6.013330 -0.535182 -0.693619

C -4.690478 -0.215970 -0.967696

O 0.021374 2.642046 -1.348021

C 0.192493 4.055630 -1.116864

C 1.629597 4.382920 -0.786354

O 2.687359 -3.446692 -0.857862

C 1.930569 -4.631062 -0.729733

H -0.392551 -3.553585 0.364568

H -0.061762 -2.316701 1.582707

H 0.636811 -2.063796 -1.384119

H 0.984565 -0.207727 1.009665

H -0.208429 0.305835 -1.738087

H 2.331472 -2.712776 1.033658

H 3.961884 -1.441489 -0.973133

H 2.226692 0.107791 -1.750425

H 2.703390 1.089764 2.541661

H 3.796432 2.413297 2.079314

H 4.445661 0.973740 2.883009

H 5.396074 0.478707 -0.725605

H 5.371681 2.064144 0.066193

H 6.064918 0.665462 0.910426

H -3.822652 1.114140 2.026224

H -6.180329 0.530342 2.515534

H -7.587665 -0.521650 0.768836

H -6.622140 -0.991945 -1.464129

H -4.261707 -0.415699 -1.942568

H -0.117525 4.522512 -2.049497

H -0.484597 4.363305 -0.321070

H 1.938939 3.888089 0.133367

H 2.290062 4.067098 -1.594745

H 1.732009 5.461598 -0.657698

H 2.466621 -5.415191 -1.262641

H 1.825429 -4.920240 0.322082

H 0.935772 -4.525162 -1.172981

==============================

conf132_E2.47

C 0.075564 -2.504320 0.517859

C 1.110956 -1.806712 -0.335427

C 1.076614 -0.302557 -0.082619

C -0.166048 0.373869 -0.640886

C -1.493144 -0.235525 -0.214251

S -1.625263 -1.964076 0.147091

C 2.582094 -2.190834 -0.042697

C 3.385063 -0.909259 -0.288661

C 2.392306 0.181624 -0.693550

O 2.896999 1.368084 -0.119760

O 3.904392 -0.381250 0.922394

C 3.961675 1.029132 0.780524

C 3.706511 1.660197 2.128337

C 5.280487 1.458563 0.164471

N -2.516726 0.508699 -0.242459

C -0.159696 1.842622 -0.247047

O -0.192530 2.225357 0.892780

C -3.804866 0.017153 0.050361

C -4.355357 0.230832 1.312643

C -5.640775 -0.213060 1.589158

C -6.388556 -0.859264 0.611528

C -5.840816 -1.059769 -0.649861

C -4.553732 -0.624534 -0.934355

O -0.075011 2.639354 -1.307340

C -0.050180 4.058404 -1.052695

C -1.447511 4.589022 -0.832155

O 3.095356 -3.197262 -0.882482

C 2.448920 -4.444391 -0.745679

H 0.045103 -3.580761 0.356542

H 0.273737 -2.320435 1.574504

H 0.922699 -2.002170 -1.397820

H 1.126172 -0.127683 0.998228

H -0.138886 0.296571 -1.731787

H 2.687997 -2.489947 1.008552

H 4.178255 -1.082333 -1.018949

H 2.301256 0.303567 -1.775628

H 3.689277 2.745821 2.029625

H 4.497885 1.384667 2.825976

H 2.746861 1.319806 2.517382

H 5.429410 0.960413 -0.794605

H 5.278431 2.536655 0.001342

H 6.104811 1.203511 0.831349

H -3.767981 0.740755 2.066620

H -6.060427 -0.049970 2.574181

H -7.392719 -1.199343 0.829807

H -6.417176 -1.558336 -1.419408

H -4.121119 -0.775073 -1.916321

H 0.595515 4.248519 -0.196179

H 0.402415 4.487822 -1.943990

H -2.076252 4.380493 -1.698643

H -1.901100 4.138152 0.050494

H -1.406598 5.669557 -0.687254

H 2.367402 -4.733212 0.308285

H 1.450144 -4.433135 -1.192456

H 3.055499 -5.180984 -1.270834

==============================

conf133_E2.49

C 0.143838 -2.407733 0.550191

C 1.162729 -1.716634 -0.320078

C 1.006385 -0.200895 -0.290236

C -0.261364 0.269473 -0.989525

C -1.544870 -0.341068 -0.449952

S -1.574545 -2.039715 0.064293

C 2.610446 -1.907227 0.123362

C 3.362186 -0.791342 -0.625854

C 2.308152 0.293260 -0.938406

O 2.846831 1.485756 -0.413006

O 4.364781 -0.116584 0.107850

C 3.899023 1.173372 0.494307

C 3.402703 1.158797 1.931636

C 5.014564 2.173341 0.293634

N -2.601244 0.353745 -0.497703

C -0.369665 1.783633 -0.963375

O -0.417138 2.471483 -1.950351

C -3.852461 -0.159182 -0.099690

C -4.358789 0.157051 1.159383

C -5.610176 -0.309110 1.536636

C -6.368117 -1.079310 0.662030

C -5.864519 -1.382748 -0.597025

C -4.611142 -0.926074 -0.981589

O -0.363207 2.251131 0.279886

C -0.516485 3.673034 0.447662

C -1.971020 4.069675 0.346254

O 3.045833 -3.205481 -0.182411

C 4.346344 -3.470302 0.302786

H 0.218720 -3.492873 0.481419

H 0.273726 -2.114319 1.592732

H 1.075980 -2.070518 -1.354226

H 0.981188 0.128072 0.751909

H -0.203735 -0.023036 -2.042366

H 2.697085 -1.728385 1.204976

H 3.814600 -1.209495 -1.527408

H 2.165220 0.430676 -2.012324

H 2.976469 2.130980 2.182801

H 4.237972 0.956991 2.603425

H 2.645094 0.391786 2.085047

H 5.859716 1.920118 0.933899

H 5.333714 2.160531 -0.748162

H 4.667436 3.173985 0.552674

H -3.763421 0.761137 1.833188

H -5.995317 -0.066220 2.519285

H -7.345971 -1.436473 0.958431

H -6.449165 -1.979068 -1.286614

H -4.212942 -1.156127 -1.962696

H -0.116636 3.876309 1.439032

H 0.095480 4.183046 -0.295166

H -2.067507 5.142639 0.518780

H -2.369771 3.840630 -0.642066

H -2.563673 3.544870 1.096620

H 5.091042 -2.824587 -0.173515

H 4.396421 -3.323309 1.387380

H 4.576354 -4.508642 0.069545

==============================

conf134_E2.51

C 0.143975 -2.407998 0.550145

C 1.162867 -1.716903 -0.320134

C 1.006452 -0.201181 -0.290433

C -0.261291 0.269186 -0.989706

C -1.544790 -0.341294 -0.450071

S -1.574439 -2.039871 0.064383

C 2.610565 -1.907325 0.123480

C 3.362344 -0.791375 -0.625620

C 2.308199 0.292945 -0.938651

O 2.846634 1.485706 -0.413658

O 4.364443 -0.116372 0.108564

C 3.898613 1.173853 0.494060

C 3.401972 1.160054 1.931282

C 5.014188 2.173731 0.293055

N -2.601167 0.353520 -0.497966

C -0.369480 1.783365 -0.963394

O -0.416744 2.471359 -1.950281

C -3.852417 -0.159190 -0.099861

C -4.611438 -0.925793 -0.981771

C -5.864863 -1.382176 -0.597110

C -6.368237 -1.078753 0.662057

C -5.610014 -0.308829 1.536633

C -4.358531 0.157037 1.159278

O -0.363154 2.250661 0.279935

C -0.516323 3.672546 0.447983

C -1.970852 4.069254 0.346706

O 3.046128 -3.205536 -0.182161

C 4.346458 -3.470301 0.303548

H 0.218780 -3.493136 0.481272

H 0.273920 -2.114653 1.592696

H 1.076219 -2.070913 -1.354245

H 0.981214 0.127845 0.751689

H -0.203733 -0.023281 -2.042561

H 2.697040 -1.728363 1.205095

H 3.815254 -1.209474 -1.526951

H 2.165295 0.429927 -2.012629

H 2.644670 0.392780 2.084926

H 2.975263 2.132214 2.181721

H 4.237159 0.959005 2.603399

H 5.859232 1.920854 0.933595

H 5.333510 2.160375 -0.748683

H 4.666978 3.174501 0.551497

H -4.213444 -1.155783 -1.962973

H -6.449797 -1.978212 -1.286702

H -7.346158 -1.435681 0.958514

H -5.994975 -0.065895 2.519342

H -3.762895 0.760830 1.833110

H -0.116427 3.875613 1.439376

H 0.095636 4.182659 -0.294779

H -2.369630 3.840415 -0.641652

H -2.563478 3.544296 1.096984

H -2.067303 5.142186 0.519449

H 4.576667 -4.508587 0.070262

H 5.091292 -2.824442 -0.172348

H 4.396056 -3.323448 1.388182

==============================

conf135_E2.51

C -0.042262 2.333575 -0.870153

C 1.043446 1.908840 0.092878

C 1.025834 0.395605 0.278870

C -0.179513 -0.100847 1.061463

C -1.522717 0.294307 0.470259

S -1.716890 1.876999 -0.307935

C 2.493719 2.200327 -0.370137

C 3.323603 1.022642 0.153030

C 2.377684 0.113344 0.934156

O 2.872123 -1.190072 0.721914

O 3.716636 0.161224 -0.905986

C 3.773973 -1.163130 -0.393989

C 3.288664 -2.113755 -1.463695

C 5.169004 -1.487710 0.103734

N -2.506257 -0.479927 0.655137

C -0.114751 -1.608505 1.235059

O -0.050099 -2.162578 2.301865

C -3.807008 -0.158770 0.218096

C -4.599542 0.729773 0.942336

C -5.897419 0.994235 0.526699

C -6.411754 0.379368 -0.608380

C -5.620254 -0.511415 -1.324370

C -4.324718 -0.788756 -0.911881

O -0.099807 -2.237099 0.065804

C 0.023432 -3.669245 0.107825

C -0.082596 -4.180128 -1.305754

O 3.036428 3.387701 0.155953

C 2.359175 4.554776 -0.257853

H -0.097315 3.413858 -0.997975

H 0.124775 1.879766 -1.847626

H 0.902067 2.395726 1.064944

H 1.030438 -0.074994 -0.710170

H -0.146779 0.334617 2.064806

H 2.541202 2.220779 -1.466832

H 4.186387 1.371310 0.724527

H 2.349949 0.323329 2.005655

H 2.272606 -1.847199 -1.754962

H 3.296000 -3.134847 -1.080608

H 3.941986 -2.062629 -2.335472

H 5.177308 -2.475845 0.564756

H 5.873290 -1.480403 -0.728889

H 5.483036 -0.751520 0.845149

H -4.193518 1.203048 1.828467

H -6.508394 1.685339 1.094151

H -7.424072 0.588180 -0.929665

H -6.014044 -0.997799 -2.208254

H -3.703898 -1.488142 -1.458736

H 0.984498 -3.917114 0.561760

H -0.769490 -4.068862 0.741097

H 0.009288 -5.266766 -1.304734

H -1.047861 -3.916002 -1.739543

H 0.709198 -3.766978 -1.931173

H 1.379607 4.645259 0.221599

H 2.971237 5.405312 0.038954

H 2.228286 4.569492 -1.345805

==============================

conf136_E2.52

C -0.042157 2.333750 -0.869894

C 1.043636 1.908858 0.092960

C 1.025898 0.395622 0.278895

C -0.179420 -0.100749 1.061539

C -1.522640 0.294460 0.470357

S -1.716766 1.877402 -0.307383

C 2.493882 2.200224 -0.370193

C 3.323708 1.022493 0.152949

C 2.377784 0.113259 0.934116

O 2.872168 -1.190165 0.721911

O 3.716621 0.161024 -0.906110

C 3.773799 -1.163340 -0.394183

C 3.288126 -2.113851 -1.463826

C 5.168853 -1.488209 0.103285

N -2.506162 -0.479853 0.654953

C -0.114833 -1.608423 1.235214

O -0.050491 -2.162464 2.302045

C -3.806897 -0.158681 0.217887

C -4.324394 -0.788285 -0.912422

C -5.619918 -0.510970 -1.324938

C -6.411641 0.379403 -0.608657

C -5.897527 0.993882 0.526721

C -4.599646 0.729436 0.942400

O -0.099673 -2.237064 0.065977

C 0.023065 -3.669244 0.108083

C -0.083144 -4.180182 -1.305467

O 3.036745 3.387557 0.155787

C 2.359403 4.554659 -0.257801

H -0.097107 3.414039 -0.997713

H 0.124638 1.879921 -1.847398

H 0.902414 2.395716 1.065066

H 1.030399 -0.074949 -0.710162

H -0.146665 0.334746 2.064866

H 2.541267 2.220606 -1.466895

H 4.186564 1.371095 0.724366

H 2.350083 0.323255 2.005614

H 3.295346 -3.134947 -1.080737

H 3.941311 -2.062819 -2.335711

H 2.272058 -1.847122 -1.754901

H 5.872993 -1.481010 -0.729464

H 5.483142 -0.752090 0.844667

H 5.177034 -2.476358 0.564282

H -3.703386 -1.487337 -1.459494

H -6.013554 -0.997036 -2.209067

H -7.423959 0.588179 -0.929977

H -6.508668 1.684658 1.094398

H -4.193787 1.202410 1.828768

H 0.984040 -3.917452 0.562022

H -0.769990 -4.068540 0.741386

H 0.008495 -5.266842 -1.304388

H -1.048357 -3.915843 -1.739243

H 0.708736 -3.767241 -1.930921

H 1.379847 4.645003 0.221696

H 2.971436 5.405172 0.039122

H 2.228484 4.569552 -1.345750

==============================

conf137_E2.53

C -0.042275 2.333579 -0.870125

C 1.043452 1.908837 0.092877

C 1.025862 0.395598 0.278869

C -0.179467 -0.100857 1.061486

C -1.522686 0.294286 0.470290

S -1.716888 1.876998 -0.307856

C 2.493713 2.200353 -0.370150

C 3.323619 1.022684 0.153013

C 2.377726 0.113396 0.934171

O 2.872254 -1.189992 0.722034

O 3.716619 0.161249 -0.906005

C 3.773891 -1.163123 -0.394044

C 3.288315 -2.113687 -1.463689

C 5.168973 -1.487855 0.103437

N -2.506225 -0.479951 0.655149

C -0.114715 -1.608518 1.235098

O -0.050136 -2.162590 2.301907

C -3.806962 -0.158789 0.218077

C -4.324610 -0.788668 -0.911993

C -5.620134 -0.511311 -1.324503

C -6.411682 0.379382 -0.608451

C -5.897406 0.994146 0.526707

C -4.599539 0.729666 0.942372

O -0.099701 -2.237116 0.065843

C 0.023415 -3.669271 0.107889

C -0.082573 -4.180169 -1.305689

O 3.036393 3.387731 0.155956

C 2.359069 4.554782 -0.257794

H -0.097327 3.413864 -0.997941

H 0.124729 1.879772 -1.847604

H 0.902077 2.395709 1.064951

H 1.030453 -0.075003 -0.710168

H -0.146723 0.334628 2.064818

H 2.541196 2.220822 -1.466845

H 4.186425 1.371360 0.724472

H 2.349956 0.323434 2.005659

H 2.272212 -1.847086 -1.754753

H 3.295676 -3.134788 -1.080626

H 3.941472 -2.062567 -2.335591

H 5.873111 -1.480640 -0.729311

H 5.483206 -0.751677 0.844779

H 5.177244 -2.475981 0.564477

H -3.703748 -1.487977 -1.458898

H -6.013882 -0.997607 -2.208455

H -7.423993 0.588201 -0.929757

H -6.508415 1.685185 1.094202

H -4.193549 1.202871 1.828556

H 0.984421 -3.917230 0.561899

H -0.769592 -4.068808 0.741107

H 0.009175 -5.266819 -1.304638

H -1.047777 -3.915933 -1.739548

H 0.709312 -3.767131 -1.931065

H 2.228249 4.569578 -1.345754

H 1.379462 4.645144 0.221602

H 2.971044 5.405341 0.039123

==============================

conf138_E2.54

C 0.116842 -2.393317 0.755427

C 1.177262 -1.850465 -0.175820

C 1.098594 -0.329010 -0.241412

C -0.128745 0.175463 -0.983602

C -1.452449 -0.319216 -0.424341

S -1.578769 -1.958211 0.241542

C 2.641392 -2.122174 0.254638

C 3.420402 -0.872298 -0.169363

C 2.435821 0.059480 -0.872374

O 2.878424 1.359784 -0.553370

O 3.782263 -0.085744 0.956947

C 3.788311 1.276579 0.553082

C 3.278583 2.121054 1.697896

C 5.167484 1.690319 0.077159

N -2.470507 0.420668 -0.556390

C -0.134537 1.693335 -1.041525

O -0.105055 2.327906 -2.064037

C -3.752511 0.007087 -0.140920

C -4.530898 -0.807038 -0.961421

C -5.810951 -1.167157 -0.562510

C -6.322103 -0.720085 0.649880

C -5.544578 0.096984 1.462608

C -4.266154 0.467257 1.069915

O -0.136046 2.224494 0.175890

C -0.145683 3.660218 0.266266

C -1.544814 4.200455 0.080163

O 3.226161 -3.236995 -0.375120

C 2.602790 -4.462592 -0.056809

H 0.104052 -3.481736 0.795182

H 0.274404 -2.014066 1.765678

H 1.046672 -2.263841 -1.182813

H 1.087614 0.063166 0.780973

H -0.080947 -0.180678 -2.017107

H 2.698835 -2.235789 1.345096

H 4.294467 -1.137141 -0.768125

H 2.412692 -0.062964 -1.957546

H 2.277312 1.793727 1.978388

H 3.241993 3.167919 1.395018

H 3.944420 2.028314 2.556594

H 5.877757 1.641315 0.903227

H 5.502068 1.028003 -0.722580

H 5.137395 2.711930 -0.302808

H -4.126667 -1.149876 -1.906459

H -6.410851 -1.800724 -1.204138

H -7.320748 -1.002605 0.957319

H -5.935277 0.451838 2.408304

H -3.655046 1.106560 1.695415

H 0.232108 3.875015 1.263872

H 0.546871 4.063237 -0.472047

H -1.925676 3.963850 -0.913283

H -2.219027 3.778122 0.826394

H -1.534735 5.284969 0.198817

H 3.244919 -5.258036 -0.432516

H 2.487864 -4.576310 1.027144

H 1.621060 -4.552337 -0.532043

==============================

conf139_E2.61

C -0.358355 -2.509854 0.533179

C 0.792037 -1.846604 -0.177989

C 0.853344 -0.348583 0.097373

C -0.302422 0.415434 -0.533706

C -1.690951 -0.099634 -0.184378

S -1.976288 -1.837680 0.034646

C 2.176954 -2.305979 0.295254

C 3.114747 -1.220329 -0.246761

C 2.236707 0.039798 -0.434585

O 2.869481 1.038305 0.341198

O 4.082518 -0.819104 0.707751

C 4.194726 0.593773 0.615922

C 4.626919 1.140221 1.952689

C 5.127396 0.989330 -0.517362

N -2.646930 0.729460 -0.185172

C -0.208874 1.883709 -0.151020

O -0.213998 2.277456 0.985292

C -3.982400 0.333022 0.029149

C -4.726558 -0.232414 -1.004649

C -6.056025 -0.571574 -0.793431

C -6.650393 -0.350710 0.443104

C -5.906724 0.220244 1.469501

C -4.579310 0.569631 1.265793

O -0.088669 2.666449 -1.218889

C 0.066754 4.080467 -0.978954

C 1.499836 4.417560 -0.639866

O 2.537300 -3.632025 -0.007363

C 2.306041 -4.047656 -1.339404

H -0.423886 -3.573569 0.301511

H -0.254369 -2.396254 1.613055

H 0.703507 -1.999446 -1.259432

H 0.848053 -0.186789 1.180353

H -0.218016 0.326816 -1.620967

H 2.205400 -2.240361 1.386665

H 3.595861 -1.534570 -1.175694

H 2.177997 0.342059 -1.484940

H 3.924571 0.821867 2.722651

H 4.652936 2.229377 1.915502

H 5.624136 0.774973 2.198302

H 4.772803 0.585879 -1.467505

H 5.172291 2.075971 -0.595984

H 6.130128 0.606432 -0.325385

H -4.257626 -0.399318 -1.967182

H -6.628769 -1.011188 -1.600661

H -7.687211 -0.616439 0.604414

H -6.362716 0.398951 2.435469

H -3.994529 1.021675 2.057592

H -0.242044 4.549240 -1.910959

H -0.617190 4.377942 -0.185206

H 2.167570 4.097436 -1.441017

H 1.597702 5.497586 -0.520281

H 1.804889 3.934798 0.287970

H 1.239980 -4.196137 -1.535234

H 2.704014 -3.338276 -2.070731

H 2.819891 -4.999756 -1.463525

==============================

conf140_E2.63

C -0.357735 -2.510144 0.533273

C 0.792700 -1.846820 -0.177744

C 0.853726 -0.348741 0.097314

C -0.302220 0.414997 -0.533742

C -1.690661 -0.100272 -0.184298

S -1.975666 -1.838523 0.033954

C 2.177620 -2.305900 0.295854

C 3.115344 -1.220020 -0.245841

C 2.236950 0.039646 -0.434968

O 2.869394 1.039204 0.339706

O 4.082047 -0.817835 0.709367

C 4.194448 0.594892 0.615943

C 4.625692 1.142887 1.952379

C 5.127954 0.989144 -0.517110

N -2.646655 0.728792 -0.184482

C -0.209102 1.883349 -0.151243

O -0.213736 2.277185 0.985034

C -3.982144 0.332375 0.029567

C -4.725500 -0.235154 -1.003675

C -6.055024 -0.574222 -0.792717

C -6.650253 -0.351239 0.443026

C -5.907394 0.221776 1.468855

C -4.579947 0.571160 1.265357

O -0.090052 2.666087 -1.219248

C 0.064118 4.080263 -0.979497

C 1.496976 4.418804 -0.640920

O 2.538307 -3.631847 -0.006726

C 2.307747 -4.047370 -1.338926

H -0.422926 -3.573947 0.301913

H -0.254151 -2.396177 1.613148

H 0.704432 -1.999896 -1.259176

H 0.848601 -0.186732 1.180265

H -0.217884 0.326273 -1.621009

H 2.205747 -2.240325 1.387274

H 3.597484 -1.534411 -1.174192

H 2.178099 0.340727 -1.485659

H 3.922848 0.825373 2.722232

H 4.651642 2.232000 1.913945

H 5.622772 0.778013 2.199096

H 5.172885 2.075693 -0.596923

H 6.130557 0.606489 -0.323964

H 4.774069 0.584639 -1.467065

H -4.255930 -0.403680 -1.965616

H -6.627137 -1.015433 -1.599521

H -7.687111 -0.616924 0.604144

H -6.364061 0.402147 2.434194

H -3.995820 1.024868 2.056683

H -0.245450 4.548674 -1.911430

H -0.619835 4.377136 -0.185526

H 2.164795 4.098865 -1.442075

H 1.593899 5.498981 -0.521944

H 1.802695 3.936836 0.287110

H 1.241830 -4.196385 -1.535141

H 2.705574 -3.337631 -2.069981

H 2.822161 -4.999165 -1.463052

==============================

conf141_E2.67

C -0.076140 -2.504267 -0.518439

C -1.111425 -1.806705 0.335018

C -1.076850 -0.302497 0.082524

C 0.165955 0.373534 0.640905

C 1.492960 -0.236010 0.214044

S 1.624761 -1.964600 -0.147211

C -2.582600 -2.190639 0.042487

C -3.385354 -0.908920 0.288273

C -2.392505 0.181778 0.693490

O -2.896989 1.368394 0.119775

O -3.904059 -0.380701 -0.923007

C -3.961499 1.029617 -0.780765

C -3.706131 1.661089 -2.128333

C -5.280430 1.458800 -0.164807

N 2.516579 0.508151 0.242144

C 0.159852 1.842369 0.247392

O 0.192545 2.225269 -0.892394

C 3.804757 0.016791 -0.050659

C 4.355667 0.231303 -1.312610

C 5.641221 -0.212333 -1.588984

C 6.388761 -0.859067 -0.611531

C 5.840628 -1.060368 0.649586

C 4.553431 -0.625424 0.933913

O 0.075535 2.638887 1.307842

C 0.051132 4.058023 1.053537

C 1.448564 4.588296 0.832805

O -3.095868 -3.196766 0.882714

C -2.449097 -4.443782 0.746592

H -0.046009 -3.580788 -0.357565

H -0.274136 -2.319908 -1.575035

H -0.923082 -2.002359 1.397363

H -1.126352 -0.127357 -0.998278

H 0.138832 0.295988 1.731793

H -2.688765 -2.490119 -1.008625

H -4.178902 -1.081812 1.018205

H -2.301541 0.303605 1.775582

H -3.688977 2.746684 -2.029279

H -4.497336 1.385724 -2.826229

H -2.746354 1.320893 -2.517246

H -6.104655 1.203806 -0.831834

H -5.429432 0.960483 0.794169

H -5.278467 2.536867 -0.001492

H 3.768576 0.741711 -2.066485

H 6.061177 -0.048582 -2.573772

H 7.393023 -1.198925 -0.829694

H 6.416796 -1.559317 1.419033

H 4.120567 -0.776493 1.915693

H -0.594762 4.248554 0.197267

H -0.401062 4.487343 1.945076

H 1.407918 5.668918 0.688468

H 2.077481 4.379173 1.699025

H 1.901783 4.137727 -0.050181

H -3.055031 -5.180104 1.272869

H -2.368225 -4.733538 -0.307169

H -1.450005 -4.431806 1.192643

==============================

conf142_E2.67

C 0.148701 -2.418054 0.399437

C 1.183451 -1.695776 -0.426985

C 1.042186 -0.179513 -0.329335

C -0.222683 0.341138 -0.998062

C -1.512655 -0.270771 -0.479131

S -1.566664 -1.992172 -0.055467

C 2.639250 -1.905678 0.013110

C 3.380801 -0.786464 -0.726672

C 2.343578 0.328506 -0.969128

O 2.909181 1.484237 -0.390523

O 4.425373 -0.159639 -0.011626

C 3.988680 1.111215 0.460919

C 3.538797 1.028652 1.911295

C 5.110417 2.107326 0.275954

N -2.557418 0.443147 -0.482479

C -0.305080 1.854367 -0.905778

O -0.346921 2.585085 -1.861775

C -3.813150 -0.070533 -0.099479

C -4.296579 0.173205 1.184505

C -5.550795 -0.294959 1.549696

C -6.334138 -0.994968 0.639224

C -5.853401 -1.225402 -0.644057

C -4.597513 -0.766225 -1.017193

O -0.282946 2.267442 0.356300

C -0.411635 3.682849 0.586934

C -1.860488 4.106057 0.516608

O 3.170316 -3.165856 -0.317319

C 3.186818 -4.084975 0.757707

H 0.193361 -3.497952 0.258100

H 0.283889 -2.197475 1.459427

H 1.101624 -1.999568 -1.477445

H 1.032271 0.103749 0.726852

H -0.175729 0.093826 -2.062945

H 2.720683 -1.735656 1.093429

H 3.792050 -1.191215 -1.652695

H 2.188918 0.523732 -2.032454

H 4.385162 0.751558 2.540999

H 2.750979 0.289137 2.045248

H 3.164493 2.001124 2.234029

H 5.970572 1.814350 0.878253

H 5.399322 2.141396 -0.774165

H 4.783002 3.097900 0.592716

H -3.681495 0.722702 1.886747

H -5.918000 -0.109340 2.551523

H -7.313894 -1.354096 0.926834

H -6.457730 -1.766518 -1.361648

H -4.216911 -0.939790 -2.016731

H 0.000218 3.836723 1.582258

H 0.201726 4.215079 -0.138944

H -2.454898 3.558287 1.248985

H -1.938471 5.171897 0.736201

H -2.271310 3.926313 -0.476997

H 3.832080 -3.727002 1.566375

H 2.185117 -4.263128 1.159252

H 3.584119 -5.023465 0.373754

==============================

conf143_E2.74

C -0.225690 -2.536582 0.382207

C 0.868750 -1.859780 -0.412055

C 0.952524 -0.383174 -0.040319

C -0.224459 0.437093 -0.548377

C -1.600575 -0.092901 -0.180213

S -1.873560 -1.825153 0.066854

C 2.301993 -2.382860 -0.146064

C 3.207448 -1.153857 -0.278449

C 2.310228 0.041953 -0.600418

O 2.896722 1.134045 0.074490

O 3.747641 -0.769675 0.976960

C 3.918000 0.638576 0.952789

C 3.693190 1.175954 2.345812

C 5.275379 1.010180 0.385186

N -2.565194 0.726412 -0.178057

C -0.079763 1.861736 -0.037856

O -0.163421 2.171227 1.121590

C -3.890506 0.314098 0.068051

C -4.683941 -0.157640 -0.976085

C -6.003332 -0.515163 -0.734216

C -6.539500 -0.404265 0.543068

C -5.746537 0.072423 1.580713

C -4.427744 0.436282 1.348047

O 0.203265 2.711704 -1.018021

C 0.469176 4.071923 -0.624372

C 0.833443 4.841646 -1.866961

O 2.741880 -3.352947 -1.066172

C 1.991130 -4.547762 -1.039586

H -0.346299 -3.588946 0.129857

H -0.016001 -2.460794 1.449721

H 0.674313 -1.955242 -1.486893

H 1.002021 -0.300655 1.051684

H -0.188933 0.445650 -1.641803

H 2.372181 -2.777013 0.876382

H 3.995613 -1.329320 -1.013554

H 2.244814 0.258377 -1.669373

H 4.448025 0.779670 3.025589

H 2.702806 0.885264 2.696389

H 3.766014 2.263744 2.337633

H 5.360243 2.094623 0.310252

H 6.066931 0.637734 1.036349

H 5.399376 0.580297 -0.609780

H -4.259565 -0.239330 -1.969653

H -6.614311 -0.882026 -1.549784

H -7.569067 -0.682592 0.727864

H -6.156442 0.164652 2.578921

H -3.804140 0.813042 2.149775

H -0.423105 4.473393 -0.142637

H 1.281356 4.065630 0.103494

H 1.041316 5.878723 -1.601954

H 1.723239 4.419669 -2.335530

H 0.014571 4.826602 -2.586917

H 1.881175 -4.921775 -0.015360

H 0.998595 -4.410545 -1.479215

H 2.534619 -5.283373 -1.630922

==============================

conf144_E2.75

C 0.218181 2.344602 0.785273

C -0.875581 1.880695 -0.151882

C -0.927063 0.354222 -0.180635

C 0.263480 -0.265392 -0.900292

C 1.629767 0.156294 -0.383040

S 1.876692 1.777023 0.286096

C -2.312172 2.273726 0.262568

C -3.202050 1.153904 -0.285091

C -2.278634 0.051823 -0.825304

O -2.849417 -1.163760 -0.381909

O -3.907411 0.482809 0.745849

C -4.052302 -0.865594 0.333360

C -4.136370 -1.740634 1.559121

C -5.246886 -1.028876 -0.591070

N 2.598464 -0.639814 -0.555834

C 0.169047 -1.782395 -0.907169

O 0.035742 -2.440296 -1.906613

C 3.917174 -0.303346 -0.189575

C 4.459338 -0.824795 0.983415

C 5.772368 -0.534500 1.326058

C 6.555863 0.263072 0.499818

C 6.015891 0.771087 -0.675541

C 4.701323 0.491824 -1.023444

O 0.231646 -2.282881 0.321244

C 0.170218 -3.715865 0.453776

C -1.260496 -4.198521 0.481374

O -2.769600 3.495358 -0.265259

C -2.061168 4.629209 0.187320

H 0.321083 3.427558 0.820153

H 0.022019 1.985038 1.796175

H -0.699869 2.262435 -1.164506

H -0.965352 -0.010689 0.851874

H 0.227755 0.052653 -1.946601

H -2.385829 2.287879 1.358411

H -3.886892 1.552339 -1.036326

H -2.204752 0.062342 -1.916161

H -4.199262 -2.787604 1.262660

H -5.026935 -1.486573 2.134607

H -3.251341 -1.592092 2.177925

H -5.147089 -0.384047 -1.465419

H -5.311161 -2.063559 -0.928678

H -6.165475 -0.771317 -0.062655

H 3.843921 -1.451354 1.617665

H 6.185694 -0.937329 2.242452

H 7.581422 0.482577 0.767523

H 6.620186 1.389483 -1.327763

H 4.274722 0.882220 -1.939733

H 0.733116 -4.166618 -0.362482

H 0.677422 -3.925563 1.393603

H -1.798045 -3.748460 1.316091

H -1.775063 -3.944605 -0.444852

H -1.272428 -5.282693 0.605282

H -2.654231 5.504842 -0.073724

H -1.923289 4.602326 1.273979

H -1.083886 4.714045 -0.296939

==============================

conf145_E2.75

C 0.218251 2.344577 0.785253

C -0.875516 1.880644 -0.151872

C -0.927029 0.354170 -0.180588

C 0.263502 -0.265490 -0.900219

C 1.629802 0.156205 -0.383017

S 1.876755 1.776918 0.286125

C -2.312099 2.273691 0.262579

C -3.201994 1.153949 -0.285212

C -2.278585 0.051801 -0.825307

O -2.849460 -1.163728 -0.381884

O -3.907585 0.482910 0.745610

C -4.052492 -0.865479 0.333093

C -4.136960 -1.740531 1.558823

C -5.246865 -1.028621 -0.591629

N 2.598499 -0.639898 -0.555841

C 0.169047 -1.782493 -0.907005

O 0.035655 -2.440459 -1.906393

C 3.917224 -0.303383 -0.189650

C 4.459289 -0.824410 0.983572

C 5.772325 -0.534074 1.326165

C 6.555920 0.263122 0.499651

C 6.016042 0.770718 -0.675931

C 4.701467 0.491417 -1.023786

O 0.231737 -2.282904 0.321431

C 0.170222 -3.715873 0.454044

C -1.260536 -4.198385 0.481979

O -2.769473 3.495390 -0.265123

C -2.061053 4.629189 0.187622

H 0.321202 3.427530 0.820059

H 0.022084 1.985087 1.796180

H -0.699819 2.262346 -1.164513

H -0.965354 -0.010717 0.851928

H 0.227764 0.052493 -1.946546

H -2.385771 2.287722 1.358422

H -3.886679 1.552448 -1.036556

H -2.204629 0.062270 -1.916160

H -4.199816 -2.787494 1.262324

H -5.027680 -1.486441 2.134056

H -3.252104 -1.592040 2.177888

H -6.165549 -0.770978 -0.063422

H -5.146794 -0.383789 -1.465947

H -5.311157 -2.063295 -0.929264

H 3.843785 -1.450657 1.618048

H 6.185576 -0.936569 2.242740

H 7.581485 0.482649 0.767317

H 6.620417 1.388811 -1.328370

H 4.274932 0.881486 -1.940246

H 0.732903 -4.166707 -0.362317

H 0.677611 -3.925575 1.393770

H -1.797819 -3.748272 1.316842

H -1.775312 -3.944404 -0.444116

H -1.272547 -5.282553 0.605886

H -2.654180 5.504849 -0.073186

H -1.923069 4.602097 1.274263

H -1.083822 4.714158 -0.296713

==============================

conf146_E2.77

C -0.017540 -2.456904 -0.558979

C -1.085189 -1.753774 0.240703

C -0.960153 -0.236610 0.156184

C 0.274104 0.286345 0.878546

C 1.588858 -0.314897 0.409207

S 1.672443 -2.034562 -0.022400

C -2.507345 -1.995008 -0.259724

C -3.314519 -0.854178 0.377316

C -2.291015 0.251419 0.732074

O -2.778257 1.415002 0.091732

O -4.190226 -0.218952 -0.536373

C -4.136209 1.174956 -0.266080

C -4.451335 1.932430 -1.530681

C -5.055933 1.535425 0.888827

N 2.627310 0.406085 0.462144

C 0.351043 1.800833 0.810040

O 0.335751 2.517444 1.777708

C 3.903677 -0.095627 0.136335

C 4.635708 -0.820129 1.075016

C 5.912303 -1.265734 0.760838

C 6.465984 -0.993277 -0.484285

C 5.734695 -0.264936 -1.415589

C 4.460277 0.190413 -1.108681

O 0.393753 2.231954 -0.444781

C 0.517671 3.652087 -0.647224

C 1.954373 4.092052 -0.486954

O -2.935366 -3.283494 0.091172

C -4.196176 -3.604075 -0.460769

H -0.074328 -3.539689 -0.448120

H -0.111197 -2.208835 -1.616909

H -1.035999 -2.064912 1.290760

H -0.921791 0.053658 -0.898748

H 0.185031 0.026337 1.937730

H -2.542061 -1.868482 -1.351552

H -3.854910 -1.220265 1.254859

H -2.216256 0.406142 1.812625

H -4.349253 3.003279 -1.354659

H -5.475242 1.725493 -1.842082

H -3.764053 1.629082 -2.320130

H -6.089329 1.305993 0.627302

H -4.786924 0.974048 1.785406

H -4.974096 2.600371 1.107519

H 4.198816 -1.026163 2.044963

H 6.475742 -1.828934 1.494535

H 7.461867 -1.341795 -0.725876

H 6.158972 -0.046149 -2.387733

H 3.885593 0.762160 -1.827165

H 0.162055 3.815864 -1.662391

H -0.143722 4.165705 0.049370

H 2.309749 3.902759 0.525993

H 2.597325 3.562754 -1.191335

H 2.030694 5.161927 -0.686645

H -4.430410 -4.626264 -0.168191

H -4.978966 -2.937329 -0.084531

H -4.172481 -3.536860 -1.553990

==============================

conf147_E2.78

C 0.017273 -2.456767 0.559101

C 1.085036 -1.753805 -0.240585

C 0.960118 -0.236617 -0.156178

C -0.274120 0.286365 -0.878555

C -1.588919 -0.314747 -0.409196

S -1.672620 -2.034534 0.022167

C 2.507170 -1.995148 0.259837

C 3.314442 -0.854353 -0.377152

C 2.291015 0.251244 -0.732137

O 2.778309 1.414919 -0.092010

O 4.190001 -0.219054 0.536627

C 4.136171 1.174818 0.266084

C 4.451153 1.932466 1.530618

C 5.056119 1.535008 -0.888725

N -2.627292 0.406359 -0.461853

C -0.350958 1.800861 -0.810197

O -0.335824 2.517352 -1.777959

C -3.903661 -0.095455 -0.136163

C -4.460526 0.190819 1.108692

C -5.734923 -0.264628 1.415469

C -6.465963 -0.993298 0.484196

C -5.912022 -1.265977 -0.760747

C -4.635415 -0.820263 -1.074804

O -0.393415 2.232132 0.444578

C -0.516967 3.652306 0.646887

C -1.953662 4.092498 0.487226

O 2.935072 -3.283651 -0.091148

C 4.195788 -3.604449 0.460885

H 0.074099 -3.539577 0.448501

H 0.110768 -2.208475 1.616995

H 1.035815 -2.064999 -1.290623

H 0.921797 0.053740 0.898730

H -0.185098 0.026273 -1.937727

H 2.541891 -1.868686 1.351673

H 3.854966 -1.220486 -1.254595

H 2.216273 0.405758 -1.812721

H 3.763751 1.629258 2.320015

H 4.349142 3.003296 1.354436

H 5.475011 1.725546 1.842194

H 4.974452 2.599937 -1.107567

H 6.089453 1.305490 -0.627032

H 4.787167 0.973568 -1.785283

H -3.886066 0.762862 1.827117

H -6.159401 -0.045688 2.387493

H -7.461837 -1.341898 0.725702

H -6.475251 -1.829382 -1.494450

H -4.198353 -1.026433 -2.044647

H -0.160828 3.816165 1.661861

H 0.144185 4.165711 -0.050093

H -2.309587 3.902902 -0.525474

H -2.596361 3.563577 1.192119

H -2.029683 5.162461 0.686559

H 4.430384 -4.626283 0.167361

H 4.978536 -2.937144 0.085553

H 4.171713 -3.538318 1.554163

==============================

conf148_E2.78

C 0.017482 -2.457035 0.558478

C 1.085215 -1.753826 -0.241064

C 0.960286 -0.236668 -0.156436

C -0.273874 0.286341 -0.878844

C -1.588651 -0.314701 -0.409392

S -1.672466 -2.034384 0.022073

C 2.507343 -1.995267 0.259392

C 3.314670 -0.854058 -0.376824

C 2.291171 0.251214 -0.732434

O 2.778179 1.415325 -0.092882

O 4.189126 -0.218519 0.537867

C 4.135636 1.175250 0.266878

C 4.449201 1.933235 1.531571

C 5.056871 1.535149 -0.886999

N -2.627071 0.406347 -0.462096

C -0.350762 1.800847 -0.810533

O -0.335609 2.517335 -1.778307

C -3.903330 -0.095527 -0.136041

C -4.636029 -0.818750 -1.075199

C -5.912498 -1.264568 -0.760804

C -6.465412 -0.993516 0.484974

C -5.733433 -0.266454 1.416742

C -4.459107 0.189047 1.109665

O -0.393418 2.232093 0.444221

C -0.517939 3.652194 0.646568

C -1.954849 4.091582 0.486531

O 2.935407 -3.283527 -0.092285

C 4.195875 -3.604679 0.460092

H 0.074209 -3.539797 0.447361

H 0.111187 -2.209240 1.616470

H 1.036057 -2.064830 -1.291159

H 0.921948 0.053619 0.898491

H -0.184849 0.026173 -1.938000

H 2.541942 -1.869418 1.351296

H 3.856142 -1.219803 -1.253837

H 2.216454 0.405182 -1.813095

H 5.472665 1.726285 1.844417

H 3.760826 1.630330 2.320236

H 4.347517 3.004025 1.354964

H 6.089917 1.305683 -0.624120

H 4.788876 0.973492 -1.783707

H 4.975450 2.600020 -1.106199

H -4.199715 -1.023639 -2.045648

H -6.476470 -1.826761 -1.494868

H -7.461205 -1.342179 0.726720

H -6.157066 -0.048820 2.389427

H -3.883903 0.759782 1.828544

H -0.162244 3.816140 1.661679

H 0.143170 4.166052 -0.050131

H -2.597498 3.561932 1.190925

H -2.031581 5.161401 0.686352

H -2.310272 3.902279 -0.526391

H 4.171208 -3.539680 1.553425

H 4.430805 -4.626170 0.165642

H 4.978712 -2.936855 0.085864

==============================

conf149_E2.80

C 0.024849 -2.529043 0.543800

C 1.102350 -1.762023 -0.176801

C 1.025541 -0.263205 0.090451

C -0.205412 0.383915 -0.527397

C -1.536098 -0.264176 -0.169041

S -1.652369 -2.021718 0.046649

C 2.527150 -2.087441 0.288377

C 3.354911 -0.921111 -0.264442

C 2.359669 0.246294 -0.465973

O 2.904401 1.318164 0.278130

O 4.276165 -0.416916 0.686804

C 4.263200 0.997606 0.565366

C 4.640988 1.607309 1.891229

C 5.161476 1.449689 -0.574428

N -2.564440 0.473572 -0.155909

C -0.269114 1.852316 -0.139439

O -0.260312 2.240628 0.998825

C -3.857774 -0.040205 0.064781

C -4.483032 0.183212 1.289803

C -5.774211 -0.279575 1.500347

C -6.455502 -0.950139 0.490958

C -5.834732 -1.157304 -0.734882

C -4.539906 -0.706217 -0.951952

O -0.302469 2.646014 -1.205347

C -0.412166 4.062562 -0.957430

C -1.844229 4.444863 -0.664274

O 3.008000 -3.374880 -0.013236

C 2.816523 -3.810890 -1.345177

H 0.063844 -3.595732 0.319617

H 0.120455 -2.397840 1.622434

H 1.022171 -1.928319 -1.256877

H 1.021181 -0.095521 1.172498

H -0.123826 0.308235 -1.615947

H 2.556770 -2.015713 1.379322

H 3.866185 -1.196968 -1.189510

H 2.260060 0.517532 -1.521715

H 5.665514 1.335960 2.146080

H 3.966624 1.244518 2.666547

H 4.571732 2.693461 1.831536

H 4.848656 0.993905 -1.515536

H 5.109281 2.533730 -0.678322

H 6.193641 1.162925 -0.371024

H -3.948241 0.714694 2.067536

H -6.251544 -0.110728 2.457725

H -7.464976 -1.303794 0.656886

H -6.359470 -1.673666 -1.529199

H -4.051521 -0.861175 -1.906783

H 0.253936 4.327753 -0.137491

H -0.054951 4.528298 -1.873430

H -1.909848 5.524837 -0.524478

H -2.492358 4.163999 -1.495301

H -2.200369 3.955899 0.242489

H 1.768826 -4.057077 -1.541639

H 3.147949 -3.067881 -2.076400

H 3.416086 -4.711549 -1.468701

==============================

conf150_E2.82

C -0.000364 -2.393852 -0.818748

C -1.101672 -1.782146 0.008478

C -1.017419 -0.260806 0.051053

C 0.190454 0.234630 0.834633

C 1.529658 -0.298390 0.351475

S 1.666816 -1.976424 -0.211433

C -2.509426 -1.998927 -0.563858

C -3.358985 -0.942416 0.155454

C -2.369611 0.138164 0.648384

O -2.869022 1.355349 0.129475

O -4.202166 -0.236432 -0.739197

C -4.200684 1.120967 -0.324576

C -4.472092 1.999005 -1.519614

C -5.186627 1.340817 0.810973

N 2.548271 0.439872 0.486233

C 0.230644 1.751568 0.880940

O 0.177238 2.392997 1.898697

C 3.844699 -0.004171 0.156337

C 4.427331 0.399084 -1.043460

C 5.721177 0.001818 -1.349794

C 6.445585 -0.784923 -0.461656

C 5.865405 -1.174920 0.739305

C 4.569329 -0.788162 1.051903

O 0.289202 2.276474 -0.337080

C 0.381270 3.710184 -0.430531

C 1.803580 4.170008 -0.209261

O -3.009829 -3.313130 -0.520803

C -2.880612 -3.982723 0.718571

H -0.031387 -3.483843 -0.798121

H -0.078656 -2.065390 -1.855849

H -1.056385 -2.161723 1.035099

H -0.975477 0.118618 -0.974958

H 0.087935 -0.105807 1.869587

H -2.492413 -1.730604 -1.623852

H -3.939842 -1.382364 0.969213

H -2.314717 0.175359 1.740742

H -4.405627 3.047711 -1.229856

H -5.473762 1.805460 -1.903603

H -3.739078 1.792751 -2.299229

H -5.145323 2.379394 1.139999

H -6.198973 1.116083 0.473819

H -4.946888 0.697248 1.659211

H 3.857624 1.015874 -1.727812

H 6.166004 0.312147 -2.287208

H 7.456614 -1.087636 -0.702035

H 6.423312 -1.784322 1.439518

H 4.111497 -1.085680 1.987847

H 0.040395 3.941400 -1.437619

H -0.305238 4.154548 0.289054

H 2.145579 3.911859 0.793065

H 2.471610 3.710972 -0.939100

H 1.857135 5.253478 -0.325641

H -3.220199 -3.369219 1.558235

H -3.506586 -4.871996 0.661732

H -1.847335 -4.291561 0.902276

==============================

conf151_E2.82

C -0.193271 -2.470712 0.274970

C 0.889891 -1.765889 -0.511071

C 0.913957 -0.278483 -0.156793

C -0.289842 0.489277 -0.685667

C -1.643214 -0.068999 -0.278358

S -1.862705 -1.809803 -0.044413

C 2.335721 -2.197980 -0.193937

C 3.183249 -1.018377 -0.684622

C 2.256437 0.209921 -0.715975

O 2.923551 1.187605 0.051435

O 4.257883 -0.640896 0.151584

C 3.930070 0.561825 0.840749

C 3.426836 0.261871 2.244361

C 5.146180 1.459141 0.859324

N -2.623494 0.730074 -0.229264

C -0.197359 1.936236 -0.226047

O -0.183985 2.266884 0.930439

C -3.931066 0.288612 0.057784

C -4.444321 0.442283 1.344175

C -5.746567 0.049599 1.619206

C -6.547692 -0.485233 0.616858

C -6.036364 -0.625865 -0.667660

C -4.732937 -0.241459 -0.951250

O -0.098837 2.776599 -1.251016

C 0.062099 4.175561 -0.935887

C 1.502036 4.493537 -0.608075

O 2.791604 -3.362263 -0.835470

C 2.223763 -4.568650 -0.368249

H -0.290755 -3.523255 0.017958

H 0.008288 -2.393114 1.344432

H 0.722093 -1.888125 -1.587600

H 0.934801 -0.183294 0.932629

H -0.266422 0.460246 -1.779013

H 2.447281 -2.305122 0.893774

H 3.585641 -1.268910 -1.667060

H 2.127464 0.603340 -1.726450

H 4.231106 -0.182876 2.831753

H 2.587098 -0.431252 2.230502

H 3.110277 1.187870 2.726189

H 5.470341 1.656552 -0.162113

H 4.903310 2.402305 1.349762

H 5.955164 0.977498 1.408806

H -3.815136 0.865686 2.117689

H -6.137308 0.165582 2.622615

H -7.564702 -0.785197 0.834402

H -6.654316 -1.037296 -1.456224

H -4.328403 -0.346027 -1.950972

H -0.266090 4.695397 -1.833535

H -0.605096 4.427153 -0.112627

H 1.601344 5.564080 -0.422418

H 1.828180 3.952247 0.279326

H 2.152106 4.226131 -1.442252

H 2.898176 -5.375852 -0.652855

H 2.118735 -4.561467 0.721848

H 1.247189 -4.756699 -0.821905

==============================

conf152_E2.84

C -0.000354 -2.393949 -0.818703

C -1.101703 -1.782282 0.008497

C -1.017435 -0.260947 0.051178

C 0.190447 0.234445 0.834775

C 1.529647 -0.298520 0.351522

S 1.666792 -1.976604 -0.211245

C -2.509430 -1.999030 -0.563926

C -3.359010 -0.942400 0.155212

C -2.369641 0.137974 0.648549

O -2.868917 1.355365 0.130021

O -4.201703 -0.236170 -0.739723

C -4.200357 1.121144 -0.324838

C -4.471015 1.999401 -1.519892

C -5.186892 1.340874 0.810214

N 2.548234 0.439810 0.486134

C 0.230574 1.751390 0.881205

O 0.177256 2.392724 1.899028

C 3.844677 -0.004150 0.156194

C 4.569216 -0.788554 1.051473

C 5.865312 -1.175197 0.738814

C 6.445601 -0.784681 -0.461925

C 5.721282 0.002456 -1.349784

C 4.427422 0.399620 -1.043378

O 0.288991 2.276416 -0.336768

C 0.380877 3.710148 -0.430084

C 1.803136 4.170150 -0.208848

O -3.009898 -3.313203 -0.520748

C -2.880856 -3.982639 0.718734

H -0.031391 -3.483941 -0.798159

H -0.078567 -2.065405 -1.855784

H -1.056479 -2.161935 1.035091

H -0.975502 0.118557 -0.974806

H 0.087953 -0.106085 1.869702

H -2.492337 -1.730843 -1.623953

H -3.940287 -1.382304 0.968697

H -2.314798 0.174825 1.740923

H -5.472457 1.805956 -1.904522

H -3.737520 1.793231 -2.299075

H -4.404672 3.048054 -1.229917

H -5.145725 2.379410 1.139380

H -6.199072 1.116203 0.472520

H -4.947579 0.697214 1.658500

H 4.111305 -1.086469 1.987252

H 6.423155 -1.784911 1.438806

H 7.456647 -1.087305 -0.702346

H 6.166188 0.313183 -2.287027

H 3.857789 1.016718 -1.727512

H 0.039916 3.941416 -1.437131

H -0.305648 4.154348 0.289587

H 2.471187 3.711288 -0.938776

H 1.856524 5.253639 -0.325124

H 2.145234 3.911948 0.793429

H -1.847684 -4.291853 0.902398

H -3.220122 -3.368860 1.558323

H -3.507190 -4.871672 0.662100

==============================

conf153_E2.88

C 0.266963 -2.483925 -0.524836

C -0.833863 -1.884239 0.320425

C -0.932820 -0.381829 0.071144

C 0.237839 0.399432 0.648421

C 1.618003 -0.085161 0.228203

S 1.908268 -1.793730 -0.138288

C -2.261231 -2.401098 0.016822

C -3.179157 -1.197518 0.251868

C -2.294288 -0.021186 0.667415

O -2.899500 1.114246 0.088472

O -3.729477 -0.718807 -0.965887

C -3.915588 0.680792 -0.826713

C -3.699294 1.331389 -2.172184

C -5.276566 0.990406 -0.231044

N 2.570986 0.747046 0.264826

C 0.106849 1.868462 0.275021

O 0.103649 2.269329 -0.858978

C 3.899790 0.371673 -0.018261

C 4.443688 0.647754 -1.271182

C 5.765541 0.319642 -1.537446

C 6.555495 -0.272110 -0.558550

C 6.013163 -0.535255 0.693644

C 4.690301 -0.216043 0.967668

O -0.021547 2.641978 1.348133

C -0.192708 4.055561 1.117034

C -1.629818 4.382764 0.786465

O -2.687277 -3.446742 0.857918

C -1.930387 -4.631055 0.729903

H 0.392595 -3.553564 -0.364778

H 0.061750 -2.316619 -1.582845

H -0.636738 -2.063869 1.384008

H -0.984647 -0.207703 -1.009672

H 0.208370 0.305761 1.738090

H -2.331483 -2.712943 -1.033666

H -3.961980 -1.441610 0.972968

H -2.226638 0.107487 1.750527

H -2.702537 1.089774 -2.541325

H -3.795596 2.413387 -2.079247

H -4.444698 0.973951 -2.883262

H -5.371628 2.064200 -0.066674

H -6.064651 0.665650 -0.911308

H -5.396400 0.478671 0.724945

H 3.822706 1.114488 -2.026134

H 6.180416 0.530676 -2.515364

H 7.587598 -0.521569 -0.768696

H 6.621914 -0.992132 1.464132

H 4.261450 -0.415882 1.942481

H 0.117254 4.522439 2.049689

H 0.484386 4.363302 0.321269

H -1.939039 3.888039 -0.133354

H -2.290305 4.066744 1.594761

H -1.732343 5.461452 0.657979

H -2.466284 -5.415140 1.263034

H -1.825365 -4.920414 -0.321875

H -0.935537 -4.524979 1.172991

==============================

conf154_E2.92

C 0.266828 -2.483887 -0.524924

C -0.833928 -1.884179 0.320412

C -0.932871 -0.381764 0.071105

C 0.237807 0.399419 0.648462

C 1.617965 -0.085229 0.228242

S 1.908160 -1.793772 -0.138421

C -2.261306 -2.401091 0.017007

C -3.179213 -1.197458 0.251801

C -2.294372 -0.021089 0.667292

O -2.899573 1.114277 0.088189

O -3.729383 -0.718889 -0.966090

C -3.915553 0.680710 -0.827068

C -3.699137 1.331172 -2.172582

C -5.276605 0.990335 -0.231580

N 2.571003 0.746910 0.265026

C 0.106921 1.868477 0.275175

O 0.103782 2.269436 -0.858798

C 3.899781 0.371582 -0.018238

C 4.690651 -0.215439 0.967809

C 6.013486 -0.534636 0.693600

C 6.555440 -0.272151 -0.558891

C 5.765132 0.318939 -1.537911

C 4.443305 0.647003 -1.271476

O -0.021484 2.641908 1.348333

C -0.192580 4.055519 1.117346

C -1.629658 4.382864 0.786772

O -2.687312 -3.446486 0.858443

C -1.930264 -4.630750 0.730908

H 0.392390 -3.553551 -0.364953

H 0.061565 -2.316520 -1.582916

H -0.636685 -2.063750 1.383983

H -0.984634 -0.207628 -1.009709

H 0.208301 0.305653 1.738123

H -2.331641 -2.713244 -1.033385

H -3.962132 -1.441425 0.972837

H -2.226797 0.107695 1.750395

H -4.444422 0.973590 -2.883716

H -2.702313 1.089596 -2.541567

H -3.795547 2.413174 -2.079791

H -5.371714 2.064137 -0.067292

H -6.064607 0.665522 -0.911917

H -5.396549 0.478658 0.724426

H 4.262111 -0.414768 1.942864

H 6.622501 -0.990981 1.464196

H 7.587519 -0.521588 -0.769181

H 6.179708 0.529472 -2.516064

H 3.822063 1.113210 -2.026540

H 0.117368 4.522271 2.050066

H 0.484575 4.363306 0.321651

H -1.938932 3.888176 -0.133047

H -2.290190 4.066945 1.595073

H -1.732056 5.461562 0.658259

H -2.465922 -5.414618 1.264596

H -1.825436 -4.920667 -0.320738

H -0.935327 -4.524284 1.173707

==============================

conf155_E2.91

C 0.219370 -2.469761 0.279143

C 1.219945 -1.654969 -0.509067

C 1.081583 -0.170254 -0.167041

C -0.204863 0.455022 -0.685638

C -1.486470 -0.252410 -0.272662

S -1.511337 -2.010849 -0.068090

C 2.704451 -1.918575 -0.188715

C 3.410700 -0.665921 -0.718874

C 2.355143 0.454203 -0.752740

O 2.925090 1.511644 -0.013194

O 4.454460 -0.157854 0.085931

C 4.010067 1.013707 0.763406

C 3.569865 0.685472 2.181713

C 5.121818 2.037457 0.742181

N -2.543471 0.439783 -0.194602

C -0.294375 1.901012 -0.222496

O -0.236257 2.232931 0.932321

C -3.796765 -0.136046 0.094636

C -4.518617 -0.797014 -0.897608

C -5.775096 -1.312698 -0.610349

C -6.318315 -1.174773 0.661251

C -5.598026 -0.508976 1.646406

C -4.344724 0.017058 1.366635

O -0.413894 2.743714 -1.242960

C -0.558219 4.141707 -0.918607

C -1.984639 4.455783 -0.531950

O 3.285481 -3.043014 -0.798818

C 2.876937 -4.292142 -0.279697

H 0.247206 -3.530206 0.037500

H 0.398949 -2.354311 1.349191

H 1.069009 -1.803183 -1.585011

H 1.107702 -0.063045 0.921062

H -0.187078 0.431600 -1.779376

H 2.830049 -1.979987 0.900917

H 3.818069 -0.892315 -1.705230

H 2.168214 0.813761 -1.766914

H 2.809876 -0.094244 2.194734

H 3.164059 1.579933 2.656047

H 4.428647 0.340274 2.758805

H 5.399148 2.254361 -0.289061

H 4.790587 2.955630 1.228012

H 5.990533 1.653022 1.276889

H -4.090857 -0.898023 -1.888031

H -6.330874 -1.825187 -1.385861

H -7.298166 -1.578429 0.881704

H -6.014680 -0.393800 2.639394

H -3.780175 0.544537 2.125912

H 0.142188 4.391357 -0.122742

H -0.267714 4.664154 -1.827626

H -2.273859 3.911412 0.367065

H -2.080257 5.524737 -0.335988

H -2.666598 4.190341 -1.340681

H 1.936377 -4.625728 -0.725470

H 3.652681 -5.016064 -0.528191

H 2.765006 -4.252149 0.808807

==============================

conf156_E2.93

C 0.266959 -2.483922 -0.524842

C -0.833856 -1.884244 0.320444

C -0.932803 -0.381832 0.071164

C 0.237847 0.399431 0.648471

C 1.618012 -0.085146 0.228253

S 1.908264 -1.793700 -0.138347

C -2.261236 -2.401118 0.016887

C -3.179148 -1.197512 0.251826

C -2.294291 -0.021183 0.667385

O -2.899497 1.114232 0.088408

O -3.729380 -0.718844 -0.965995

C -3.915490 0.680757 -0.826894

C -3.699034 1.331274 -2.172370

C -5.276524 0.990412 -0.231379

N 2.571024 0.747024 0.265005

C 0.106824 1.868471 0.275140

O 0.103673 2.269407 -0.858840

C 3.899817 0.371691 -0.018200

C 4.443536 0.647552 -1.271258

C 5.765366 0.319463 -1.537639

C 6.555482 -0.272074 -0.558738

C 6.013339 -0.534990 0.693582

C 4.690495 -0.215786 0.967728

O -0.021687 2.641922 1.348269

C -0.192919 4.055502 1.117192

C -1.630006 4.382663 0.786509

O -2.687309 -3.446664 0.858088

C -1.930409 -4.630992 0.730237

H 0.392617 -3.553560 -0.364795

H 0.061716 -2.316622 -1.582847

H -0.636709 -2.063881 1.384022

H -0.984583 -0.207703 -1.009652

H 0.208366 0.305698 1.738137

H -2.331495 -2.713059 -1.033573

H -3.962033 -1.441553 0.972874

H -2.226681 0.107504 1.750499

H -2.702215 1.089687 -2.541359

H -3.795425 2.413274 -2.079544

H -4.444319 0.973737 -2.883529

H -6.064555 0.665697 -0.911729

H -5.396477 0.478661 0.724587

H -5.371575 2.064206 -0.067003

H 3.822428 1.114114 -2.026213

H 6.180101 0.530332 -2.515653

H 7.587567 -0.521526 -0.768983

H 6.622215 -0.991688 1.464077

H 4.261794 -0.415448 1.942645

H 0.116937 4.522351 2.049895

H 0.484248 4.363299 0.321509

H -1.732503 5.461343 0.657932

H -1.939177 3.887872 -0.133291

H -2.290557 4.066716 1.594784

H -2.466303 -5.415003 1.263478

H -1.825382 -4.920494 -0.321501

H -0.935564 -4.524852 1.173318

==============================

conf157_E2.94

C 0.218111 2.344593 0.785242

C -0.875643 1.880649 -0.151894

C -0.927077 0.354165 -0.180633

C 0.263459 -0.265416 -0.900307

C 1.629729 0.156290 -0.383050

S 1.876638 1.777001 0.286114

C -2.312244 2.273656 0.262527

C -3.202086 1.153830 -0.285172

C -2.278647 0.051709 -0.825288

O -2.849410 -1.163844 -0.381775

O -3.907559 0.482776 0.745727

C -4.052382 -0.865649 0.333296

C -4.136608 -1.740653 1.559076

C -5.246835 -1.028974 -0.591296

N 2.598452 -0.639774 -0.555917

C 0.169113 -1.782429 -0.907138

O 0.035506 -2.440357 -1.906523

C 3.917144 -0.303253 -0.189655

C 4.701406 0.491621 -1.023709

C 6.015949 0.770946 -0.675775

C 6.555795 0.263304 0.499808

C 5.772188 -0.533959 1.326239

C 4.459176 -0.824316 0.983568

O 0.232182 -2.282877 0.321259

C 0.170731 -3.715861 0.453860

C -1.259969 -4.198553 0.481707

O -2.769692 3.495294 -0.265273

C -2.061423 4.629193 0.187461

H 0.321023 3.427548 0.820088

H 0.021954 1.985066 1.796157

H -0.699949 2.262366 -1.164531

H -0.965344 -0.010743 0.851877

H 0.227721 0.052591 -1.946628

H -2.385939 2.287771 1.358368

H -3.886852 1.552247 -1.036485

H -2.204759 0.062122 -1.916145

H -4.199366 -2.787633 1.262621

H -5.027284 -1.486643 2.134410

H -3.251689 -1.592029 2.178022

H -6.165482 -0.771293 -0.063048

H -5.146850 -0.384269 -1.465719

H -5.311115 -2.063697 -0.928785

H 4.274905 0.881728 -1.940170

H 6.620333 1.389101 -1.328147

H 7.581340 0.482854 0.767529

H 6.185400 -0.936494 2.242815

H 3.843666 -1.450622 1.617980

H 0.733553 -4.166647 -0.362434

H 0.678037 -3.925497 1.393645

H -1.271808 -5.282697 0.605849

H -1.797417 -3.748357 1.316420

H -1.774688 -3.944867 -0.444491

H -1.923594 4.602220 1.274125

H -1.084132 4.714203 -0.296741

H -2.654591 5.504776 -0.073511

==============================

conf158_E2.94

C 0.218056 2.344429 0.785470

C -0.875605 1.880598 -0.151840

C -0.927146 0.354119 -0.180617

C 0.263368 -0.265522 -0.900273

C 1.629680 0.156245 -0.383138

S 1.876598 1.776699 0.286598

C -2.312197 2.273715 0.262456

C -3.202125 1.153921 -0.285189

C -2.278739 0.051713 -0.825225

O -2.849533 -1.163779 -0.381527

O -3.907681 0.482974 0.745705

C -4.052600 -0.865461 0.333280

C -4.137110 -1.740425 1.559061

C -5.246941 -1.028670 -0.591486

N 2.598485 -0.639638 -0.556391

C 0.169071 -1.782541 -0.906989

O 0.035621 -2.440558 -1.906335

C 3.917131 -0.303118 -0.189937

C 4.701615 0.491439 -1.024076

C 6.016107 0.770801 -0.675954

C 6.555686 0.263471 0.499888

C 5.771847 -0.533474 1.326415

C 4.458874 -0.823824 0.983584

O 0.232062 -2.282914 0.321447

C 0.171129 -3.715925 0.454020

C -1.259375 -4.199197 0.481500

O -2.769511 3.495350 -0.265482

C -2.060977 4.629167 0.187017

H 0.321051 3.427377 0.820356

H 0.021687 1.984907 1.796346

H -0.699738 2.262339 -1.164439

H -0.965447 -0.010776 0.851899

H 0.227574 0.052370 -1.946620

H -2.385936 2.287955 1.358292

H -3.886817 1.552384 -1.036537

H -2.204905 0.062024 -1.916083

H -4.199833 -2.787409 1.262597

H -5.027901 -1.486406 2.134215

H -3.252306 -1.591821 2.178185

H -5.311386 -2.063433 -0.928839

H -6.165621 -0.770725 -0.063418

H -5.146698 -0.384116 -1.465992

H 4.275299 0.881308 -1.940729

H 6.620656 1.388729 -1.328390

H 7.581198 0.483022 0.767744

H 6.184844 -0.935750 2.243204

H 3.843184 -1.449881 1.618073

H 0.734320 -4.166464 -0.362154

H 0.678298 -3.925385 1.393914

H -1.270765 -5.283355 0.605604

H -1.797210 -3.749303 1.316129

H -1.773977 -3.945736 -0.444829

H -1.923267 4.602439 1.273705

H -1.083610 4.713782 -0.297100

H -2.653852 5.504859 -0.074258

==============================

conf159_E2.95

C 1.637320 -2.583215 1.043309

C 1.799811 -1.638376 -0.126658

C 0.979343 -0.376750 0.116853

C -0.519824 -0.574838 -0.085509

C -1.106599 -1.815654 0.583641

S -0.096873 -3.074674 1.301980

C 3.245680 -1.123895 -0.343616

C 3.103331 0.350518 -0.729338

C 1.608987 0.664672 -0.809252

O 1.488863 1.989387 -0.335912

O 3.509824 1.198052 0.334908

C 2.735962 2.384542 0.251681

C 2.497295 2.904093 1.649440

C 3.401970 3.404687 -0.652930

N -2.345755 -2.074532 0.646460

C -1.223172 0.670916 0.442675

O -1.332223 0.914003 1.617292

C -3.314696 -1.253637 0.033143

C -3.465906 -1.254210 -1.353307

C -4.453049 -0.478606 -1.944459

C -5.295525 0.303030 -1.163121

C -5.153402 0.290475 0.219937

C -4.176051 -0.490559 0.819919

O -1.646350 1.457898 -0.535404

C -2.298461 2.685456 -0.149449

C -1.296119 3.759087 0.203616

O 3.930341 -1.786332 -1.380667

C 4.161393 -3.154386 -1.121160

H 2.168296 -3.523859 0.901852

H 2.007528 -2.110030 1.953391

H 1.473638 -2.117924 -1.056767

H 1.158626 -0.049073 1.147245

H -0.719268 -0.669257 -1.155591

H 3.818952 -1.200655 0.589410

H 3.654771 0.570394 -1.645964

H 1.204462 0.594268 -1.821357

H 3.448252 3.148778 2.123728

H 1.982654 2.146249 2.240783

H 1.885335 3.805408 1.609410

H 2.757033 4.277459 -0.760489

H 4.354299 3.719309 -0.224664

H 3.577769 2.976417 -1.640703

H -2.807388 -1.865976 -1.958412

H -4.562196 -0.484828 -3.022048

H -6.062176 0.910393 -1.626965

H -5.808647 0.891612 0.838476

H -4.060303 -0.505211 1.896554

H -2.888335 2.962006 -1.021134

H -2.969623 2.471839 0.682255

H -1.831791 4.679955 0.440562

H -0.701548 3.466679 1.068050

H -0.624513 3.949752 -0.633241

H 4.633096 -3.294144 -0.141857

H 3.235171 -3.736428 -1.157272

H 4.832785 -3.520790 -1.896385

==============================

conf160_E2.96

C -0.225317 -2.474856 0.496187

C 0.876996 -1.805191 -0.281588

C 0.920775 -0.301392 -0.038983

C -0.274409 0.431549 -0.631564

C -1.637514 -0.108092 -0.221655

S -1.877074 -1.843872 0.057988

C 2.292447 -2.226134 0.131253

C 3.178208 -1.141677 -0.503591

C 2.274200 0.100911 -0.649470

O 2.970607 1.135050 0.007426

O 4.264269 -0.706531 0.291665

C 3.976542 0.573803 0.845303

C 3.489308 0.447528 2.280309

C 5.214219 1.436092 0.745460

N -2.612980 0.697964 -0.212862

C -0.197525 1.903408 -0.257579

O -0.172524 2.299501 0.878095

C -3.930123 0.275658 0.058334

C -4.705889 -0.299664 -0.946295

C -6.018738 -0.664934 -0.681815

C -6.565222 -0.460513 0.579436

C -5.790131 0.119757 1.576941

C -4.478891 0.494589 1.320353

O -0.130296 2.686290 -1.329319

C -0.006350 4.104805 -1.095547

C 1.423897 4.483029 -0.789841

O 2.663623 -3.554016 -0.155233

C 2.360949 -4.015085 -1.457495

H -0.280497 -3.544388 0.290419

H -0.074165 -2.335316 1.567527

H 0.740324 -1.981744 -1.354612

H 0.946506 -0.118808 1.038855

H -0.232306 0.337790 -1.720858

H 2.376826 -2.135864 1.217779

H 3.576799 -1.484007 -1.459170

H 2.140693 0.392334 -1.693746

H 3.219360 1.432395 2.663836

H 4.285585 0.034525 2.900845

H 2.621384 -0.205919 2.351181

H 5.526246 1.507929 -0.296155

H 5.002703 2.434788 1.128798

H 6.020319 0.998885 1.334951

H -4.274085 -0.453622 -1.928115

H -6.616165 -1.111883 -1.466829

H -7.589330 -0.746217 0.782368

H -6.208522 0.285380 2.562070

H -3.870036 0.953421 2.089833

H -0.349258 4.562321 -2.021138

H -0.679903 4.385342 -0.286836

H 2.083121 4.175391 -1.602643

H 1.493832 5.566288 -0.680182

H 1.761878 4.016810 0.134921

H 2.880036 -4.964393 -1.581328

H 1.287407 -4.182940 -1.585518

H 2.705298 -3.326194 -2.234014

==============================

conf161_E2.97

C -0.225288 -2.474858 0.496146

C 0.877019 -1.805171 -0.281616

C 0.920791 -0.301375 -0.038980

C -0.274413 0.431575 -0.631516

C -1.637506 -0.108094 -0.221606

S -1.877046 -1.843892 0.057938

C 2.292475 -2.226109 0.131212

C 3.178217 -1.141643 -0.503641

C 2.274200 0.100942 -0.649489

O 2.970607 1.135074 0.007424

O 4.264291 -0.706500 0.291596

C 3.976543 0.573802 0.845288

C 3.489276 0.447465 2.280275

C 5.214217 1.436104 0.745503

N -2.612970 0.697966 -0.212717

C -0.197540 1.903423 -0.257488

O -0.172482 2.299475 0.878199

C -3.930123 0.275633 0.058395

C -4.705862 -0.299592 -0.946312

C -6.018724 -0.664873 -0.681910

C -6.565252 -0.460554 0.579338

C -5.790192 0.119624 1.576918

C -4.478939 0.494474 1.320408

O -0.130391 2.686336 -1.329215

C -0.006403 4.104855 -1.095455

C 1.423893 4.483113 -0.790000

O 2.663686 -3.554000 -0.155238

C 2.360952 -4.015190 -1.457444

H -0.280451 -3.544388 0.290362

H -0.074143 -2.335330 1.567488

H 0.740344 -1.981695 -1.354644

H 0.946551 -0.118813 1.038862

H -0.232324 0.337853 -1.720814

H 2.376858 -2.135823 1.217737

H 3.576793 -1.483967 -1.459229

H 2.140680 0.392390 -1.693757

H 4.285536 0.034433 2.900814

H 2.621346 -0.205981 2.351102

H 3.219323 1.432319 2.663833

H 5.002709 2.434772 1.128916

H 6.020319 0.998849 1.334957

H 5.526240 1.508016 -0.296107

H -4.274030 -0.453464 -1.928133

H -6.616126 -1.111747 -1.466985

H -7.589369 -0.746268 0.782210

H -6.208616 0.285167 2.562047

H -3.870113 0.953245 2.089947

H -0.349460 4.562357 -2.020998

H -0.679833 4.385403 -0.286645

H 1.762035 4.016913 0.134711

H 2.082982 4.175468 -1.602908

H 1.493841 5.566375 -0.680371

H 1.287410 -4.183091 -1.585399

H 2.705241 -3.326366 -2.234049

H 2.880058 -4.964495 -1.581221

==============================

conf162_E3.00

C 0.266859 -2.484007 -0.524715

C -0.833938 -1.884213 0.320514

C -0.932824 -0.381811 0.071159

C 0.237886 0.399423 0.648418

C 1.618013 -0.085262 0.228186

S 1.908182 -1.793842 -0.138233

C -2.261331 -2.401028 0.016939

C -3.179209 -1.197393 0.251908

C -2.294289 -0.021057 0.667347

O -2.899424 1.114336 0.088249

O -3.729567 -0.718783 -0.965870

C -3.915570 0.680843 -0.826872

C -3.699227 1.331222 -2.172436

C -5.276504 0.990644 -0.231204

N 2.571042 0.746902 0.264843

C 0.106891 1.868448 0.275040

O 0.103647 2.269344 -0.858957

C 3.899844 0.371577 -0.018291

C 4.690496 -0.215853 0.967689

C 6.013363 -0.535028 0.693621

C 6.555567 -0.272118 -0.558670

C 5.765484 0.319382 -1.537622

C 4.443632 0.647438 -1.271320

O -0.021439 2.641932 1.348164

C -0.192610 4.055541 1.117118

C -1.629731 4.382799 0.786688

O -2.687425 -3.446608 0.858090

C -1.930648 -4.631000 0.730082

H 0.392453 -3.553636 -0.364558

H 0.061643 -2.316786 -1.582737

H -0.636825 -2.063814 1.384104

H -0.984586 -0.207719 -1.009667

H 0.208431 0.305727 1.738087

H -2.331604 -2.712922 -1.033532

H -3.962015 -1.441391 0.973060

H -2.226698 0.107729 1.750452

H -2.702456 1.089542 -2.541492

H -3.795551 2.413235 -2.079710

H -4.444601 0.973644 -2.883478

H -6.064655 0.665941 -0.911420

H -5.396368 0.478974 0.724815

H -5.371445 2.064457 -0.066897

H 4.261760 -0.415509 1.942590

H 6.622202 -0.991693 1.464163

H 7.587669 -0.521543 -0.768860

H 6.180269 0.530262 -2.515611

H 3.822569 1.113980 -2.026322

H 0.117433 4.522361 2.049775

H 0.484448 4.363288 0.321325

H -2.290155 4.066848 1.595064

H -1.732193 5.461491 0.658182

H -1.939097 3.888052 -0.133067

H -0.935828 -4.525044 1.173266

H -2.466681 -5.415056 1.263116

H -1.825559 -4.920315 -0.321701

**Optimized Cartesian Coordinates of the studied conformers 23a at m06-2x/def2-TZVP/SMD/ACN level of theory**

==============================

**conf000_E0.00**

C 0.035545 -2.260646 -1.006772

C -1.128381 -1.368658 -0.649434

C -0.963005 -0.755126 0.740125

C 0.157006 0.275324 0.845927

C 1.514698 -0.262622 0.407207

S 1.653399 -1.420986 -0.923840

C -2.475785 -2.086800 -0.547293

C -3.340316 -1.091400 0.236207

C -2.365374 -0.264757 1.119578

O -2.574928 1.074942 0.703397

O -3.888869 -0.133703 -0.650946

C -3.793302 1.134568 -0.024636

C -4.961756 1.353858 0.922036

C -3.678411 2.201430 -1.083897

N 2.539678 0.243438 0.955788

C -0.056193 1.536113 0.012272

O -0.156397 1.553030 -1.188417

C 3.844385 -0.102550 0.548227

C 4.610341 -0.964130 1.330429

C 5.911110 -1.267066 0.952606

C 6.460782 -0.705383 -0.193911

C 5.698579 0.164683 -0.964529

C 4.394377 0.467470 -0.598914

O -0.023369 2.625022 0.773196

C -0.147663 3.891028 0.098737

C 1.185917 4.339882 -0.452654

O -2.314855 -3.276382 0.195964

C -3.383128 -4.185863 0.028923

H 0.074420 -3.119796 -0.340081

H -0.027434 -2.620326 -2.034329

H -1.234562 -0.570326 -1.386029

H -0.709042 -1.566598 1.429557

H 0.276056 0.584341 1.883171

H -2.910341 -2.305603 -1.528197

H -4.120351 -1.592377 0.812595

H -2.562805 -0.361789 2.187948

H -5.897481 1.361691 0.362293

H -5.004953 0.558967 1.668700

H -4.847966 2.309016 1.435166

H -4.579485 2.213064 -1.697395

H -2.810076 1.998744 -1.710524

H -3.564539 3.178242 -0.613183

H 4.177554 -1.392135 2.226348

H 6.498901 -1.943614 1.560661

H 7.477598 -0.939812 -0.481597

H 6.119912 0.611366 -1.856666

H 3.794012 1.146673 -1.193282

H -0.510987 4.574595 0.863571

H -0.898958 3.799210 -0.684852

H 1.923633 4.415561 0.347293

H 1.076970 5.321814 -0.915476

H 1.550826 3.641198 -1.205611

H -3.455415 -4.509886 -1.014475

H -4.341331 -3.750938 0.328923

H -3.175481 -5.048979 0.659443

==============================

**conf001_E0.21**

C -0.265900 -2.337356 0.883108

C 0.954595 -1.498408 0.591325

C 0.825248 -0.747648 -0.732803

C -0.233090 0.353140 -0.737468

C -1.619264 -0.140169 -0.339187

S -1.824734 -1.390025 0.896866

C 2.244763 -2.301103 0.402170

C 3.174209 -1.308944 -0.309015

C 2.255274 -0.312022 -1.069261

O 2.564407 0.945768 -0.488898

O 3.836845 -0.503367 0.648597

C 3.814901 0.832630 0.174360

C 4.954484 1.073112 -0.802205

C 3.834605 1.774077 1.351665

N -2.611796 0.461279 -0.849765

C 0.076221 1.519300 0.196024

O 0.144777 1.438873 1.396331

C -3.936736 0.156659 -0.476673

C -4.456519 0.632388 0.726152

C -5.779156 0.374233 1.058353

C -6.589861 -0.358185 0.199248

C -6.069899 -0.826103 -1.001934

C -4.751084 -0.565038 -1.346766

O 0.184102 2.659596 -0.475383

C 0.531985 3.818705 0.301945

C 0.737733 4.964313 -0.654718

O 1.985274 -3.407997 -0.434943

C 2.991298 -4.398253 -0.375179

H -0.367531 -3.128715 0.143393

H -0.219530 -2.791789 1.873320

H 1.124301 -0.781379 1.397079

H 0.525016 -1.474396 -1.494585

H -0.335546 0.757041 -1.743500

H 2.674024 -2.634521 1.352743

H 3.886721 -1.815763 -0.962486

H 2.440002 -0.286960 -2.144138

H 4.895599 2.087622 -1.197151

H 5.911311 0.945515 -0.295144

H 4.901730 0.369926 -1.635249

H 3.798351 2.805954 1.002078

H 4.752703 1.631817 1.922052

H 2.973292 1.577674 1.990086

H -3.818871 1.204976 1.389755

H -6.176663 0.747689 1.994014

H -7.620867 -0.558313 0.461183

H -6.695102 -1.395270 -1.678776

H -4.341230 -0.918798 -2.284866

H 1.437802 3.591520 0.865844

H -0.273310 4.019105 1.010099

H 1.001508 5.861595 -0.093988

H -0.172209 5.166044 -1.220900

H 1.545593 4.743218 -1.353374

H 2.709692 -5.188872 -1.068918

H 3.065314 -4.813698 0.635205

H 3.970140 -4.004736 -0.665750

==============================

**conf002_E0.59**

C -0.266402 -2.337082 0.883179

C 0.954237 -1.498355 0.591357

C 0.825020 -0.747520 -0.732737

C -0.233207 0.353394 -0.737413

C -1.619424 -0.139744 -0.339118

S -1.825050 -1.389471 0.897067

C 2.244222 -2.301332 0.402116

C 3.173864 -1.309410 -0.309154

C 2.255114 -0.312076 -1.069102

O 2.564527 0.945464 -0.488355

O 3.837024 -0.504189 0.648381

C 3.815199 0.831937 0.174468

C 4.954534 1.072400 -0.802401

C 3.835495 1.773098 1.351995

N -2.611907 0.461688 -0.849771

C 0.076319 1.519545 0.196012

O 0.144630 1.439243 1.396337

C -3.936859 0.157056 -0.476716

C -4.750996 -0.565225 -1.346516

C -6.069819 -0.826252 -1.001700

C -6.589979 -0.357763 0.199175

C -5.779472 0.375209 1.057989

C -4.456826 0.633359 0.725799

O 0.184744 2.659723 -0.475513

C 0.533209 3.818710 0.301726

C 0.739712 4.964095 -0.655034

O 1.984383 -3.408200 -0.434950

C 2.990187 -4.398678 -0.375270

H -0.368216 -3.128398 0.143443

H -0.220028 -2.791568 1.873367

H 1.124132 -0.781378 1.397122

H 0.524739 -1.474191 -1.494571

H -0.335619 0.757264 -1.743464

H 2.673471 -2.634849 1.352658

H 3.886031 -1.816415 -0.962857

H 2.439826 -0.286739 -2.143977

H 5.911485 0.944465 -0.295663

H 4.901357 0.369409 -1.635583

H 4.895755 2.087010 -1.197102

H 2.974363 1.576728 1.990669

H 3.799359 2.805067 1.002672

H 4.753753 1.630493 1.922032

H -4.340981 -0.919447 -2.284371

H -6.694872 -1.395863 -1.678307

H -7.620991 -0.557882 0.461093

H -6.177137 0.749109 1.993405

H -3.819320 1.206371 1.389167

H 1.438836 3.591050 0.865743

H -0.272038 4.019673 1.009776

H 1.547527 4.742494 -1.353579

H 1.003916 5.861279 -0.094348

H -0.170055 5.166282 -1.221333

H 3.969088 -4.005399 -0.665968

H 2.708320 -5.189257 -1.068949

H 3.064233 -4.814112 0.635118

==============================

**conf003_E0.60**

C -0.036083 -2.260240 1.007018

C 1.128101 -1.368624 0.649629

C 0.962829 -0.755193 -0.740025

C -0.156925 0.275556 -0.845819

C -1.514729 -0.262136 -0.407163

S -1.653602 -1.419905 0.924452

C 2.475371 -2.086998 0.547532

C 3.340046 -1.091729 -0.235924

C 2.365327 -0.265251 -1.119658

O 2.575255 1.074632 -0.704245

O 3.888203 -0.133845 0.651274

C 3.793362 1.134165 0.024376

C 4.962267 1.352706 -0.921907

C 3.678410 2.201522 1.083150

N -2.539617 0.243579 -0.956161

C 0.056631 1.536315 -0.012216

O 0.157130 1.553379 1.188442

C -3.844319 -0.102458 -0.548561

C -4.394497 0.467994 0.598272

C -5.698641 0.165039 0.963946

C -6.460568 -0.705612 0.193704

C -5.910668 -1.267789 -0.952447

C -4.609937 -0.964705 -1.330336

O 0.023797 2.625167 -0.773254

C 0.147850 3.891253 -0.098866

C -1.185629 4.339663 0.453153

O 2.314327 -3.276522 -0.195818

C 3.382519 -4.186091 -0.028743

H -0.075392 -3.119277 0.340201

H 0.026905 -2.620122 2.034507

H 1.234466 -0.570233 1.386102

H 0.708639 -1.566686 -1.429329

H -0.275880 0.584709 -1.883032

H 2.909914 -2.305927 1.528413

H 4.120310 -1.592713 -0.812012

H 2.562701 -0.362898 -2.187985

H 5.005566 0.557497 -1.668216

H 4.848902 2.307674 -1.435474

H 5.897747 1.360535 -0.361757

H 4.579202 2.212948 1.697064

H 2.809678 1.999442 1.709411

H 3.565221 3.178182 0.611976

H -3.794342 1.147643 1.192337

H -6.120171 0.612046 1.855827

H -7.477340 -0.940145 0.481465

H -6.498218 -1.944844 -1.560169

H -4.176937 -1.393121 -2.225956

H 0.510436 4.574931 -0.863947

H 0.899631 3.799776 0.684309

H -1.076769 5.321735 0.915694

H -1.549920 3.641016 1.206421

H -1.923729 4.414948 -0.346480

H 3.175203 -5.048865 -0.659842

H 3.454296 -4.510684 1.014514

H 4.340856 -3.750961 -0.328020

==============================

**conf004_E0.93**

C 0.035392 -2.280667 -0.911940

C -1.151414 -1.406575 -0.586152

C -0.988206 -0.746841 0.784014

C 0.106607 0.312597 0.855364

C 1.475382 -0.203696 0.426523

S 1.633679 -1.399414 -0.868545

C -2.486650 -2.152944 -0.436243

C -3.358088 -1.145968 0.316601

C -2.395948 -0.279268 1.172923

O -2.640400 1.048349 0.736150

O -3.910482 -0.221992 -0.602922

C -3.851775 1.062501 -0.006068

C -5.034712 1.277480 0.923492

C -3.751760 2.105795 -1.090163

N 2.490299 0.343549 0.953560

C -0.140421 1.542645 -0.013560

O -0.257352 1.519768 -1.212659

C 3.801252 0.018123 0.549205

C 4.598761 -0.783411 1.363199

C 5.904948 -1.065091 0.987748

C 6.428794 -0.540592 -0.188052

C 5.635069 0.270450 -0.990687

C 4.325152 0.550849 -0.627664

O -0.115277 2.655567 0.712095

C -0.260522 3.897888 -0.001333

C 1.062932 4.342926 -0.579571

O -2.357038 -3.301395 0.377938

C -2.333329 -4.515243 -0.346758

H 0.101343 -3.108869 -0.206774

H -0.014734 -2.684026 -1.923763

H -1.279120 -0.637997 -1.350514

H -0.708204 -1.535125 1.490402

H 0.221507 0.654419 1.882823

H -2.936978 -2.412984 -1.398112

H -4.133655 -1.647659 0.897982

H -2.577624 -0.361077 2.245241

H -5.066437 0.501895 1.690722

H -4.948624 2.248122 1.412371

H -5.964767 1.248490 0.355034

H -4.647892 2.082364 -1.710548

H -2.873919 1.908038 -1.704895

H -3.664106 3.095992 -0.642328

H 4.186007 -1.181854 2.281891

H 6.517392 -1.695366 1.620712

H 7.449957 -0.757787 -0.473862

H 6.036080 0.688166 -1.905909

H 3.700287 1.184074 -1.247068

H -0.624315 4.601565 0.744798

H -1.017875 3.773030 -0.774453

H 1.428109 3.624574 -1.313706

H 1.807311 4.451249 0.210408

H 0.939343 5.308521 -1.072137

H -2.161261 -5.317052 0.369967

H -1.534023 -4.528658 -1.093752

H -3.289180 -4.684407 -0.852811

==============================

**conf005_E0.96**

C -0.265742 -2.337490 0.882964

C 0.954717 -1.498457 0.591277

C 0.825357 -0.747674 -0.732831

C -0.233022 0.353080 -0.737496

C -1.619175 -0.140287 -0.339219

S -1.824548 -1.390121 0.896960

C 2.244960 -2.301044 0.402151

C 3.174338 -1.308780 -0.308946

C 2.255363 -0.311942 -1.069267

O 2.564386 0.945894 -0.488982

O 3.836737 -0.503109 0.648777

C 3.814808 0.832838 0.174481

C 4.954508 1.073371 -0.801941

C 3.834274 1.774351 1.351738

N -2.611753 0.461030 -0.849801

C 0.076190 1.519293 0.195965

O 0.144871 1.438919 1.396265

C -3.936669 0.156394 -0.476666

C -4.456374 0.632051 0.726228

C -5.778997 0.373907 1.058478

C -6.589761 -0.358436 0.199359

C -6.069872 -0.826284 -1.001877

C -4.751066 -0.565222 -1.346767

O 0.183787 2.659605 -0.475476

C 0.531446 3.818803 0.301815

C 0.736687 4.964477 -0.654869

O 1.985566 -3.407942 -0.434989

C 2.991754 -4.398041 -0.375410

H -0.367395 -3.128720 0.143114

H -0.219329 -2.792103 1.873094

H 1.124322 -0.781439 1.397061

H 0.525143 -1.474403 -1.494635

H -0.335489 0.756954 -1.743539

H 2.674205 -2.634459 1.352734

H 3.887018 -1.815452 -0.962343

H 2.440121 -0.286950 -2.144142

H 4.895654 2.087885 -1.196875

H 5.911272 0.945785 -0.294756

H 4.901886 0.370199 -1.635006

H 4.752342 1.632252 1.922212

H 2.972928 1.577861 1.990088

H 3.797909 2.806204 1.002095

H -3.818676 1.204572 1.389839

H -6.176454 0.747313 1.994180

H -7.620757 -0.558560 0.461337

H -6.695115 -1.395395 -1.678728

H -4.341277 -0.918923 -2.284918

H 1.437425 3.591891 0.865569

H -0.273783 4.018973 1.010108

H 1.544554 4.743701 -1.353614

H 1.000185 5.861849 -0.094155

H -0.173389 5.165882 -1.220952

H 3.066255 -4.813242 0.635040

H 3.970421 -4.004470 -0.666494

H 2.709953 -5.188863 -1.068837

==============================

**conf006_E1.12**

C 0.266419 2.349301 0.792436

C -0.977048 1.535521 0.526583

C -0.853245 0.745111 -0.776541

C 0.172419 -0.384769 -0.744438

C 1.570031 0.080686 -0.353669

S 1.800983 1.362762 0.844448

C -2.251018 2.363598 0.289621

C -3.189225 1.365031 -0.391239

C -2.288055 0.339412 -1.131635

O -2.634449 -0.908417 -0.550322

O -3.843171 0.588228 0.595371

C -3.867639 -0.753030 0.137242

C -5.032708 -0.973900 -0.813403

C -3.889368 -1.678759 1.326999

N 2.548963 -0.561395 -0.840287

C -0.171823 -1.515165 0.220664

O -0.266748 -1.393518 1.415811

C 3.879318 -0.279591 -0.468143

C 4.376035 -0.718454 0.758268

C 5.702364 -0.482180 1.091914

C 6.539768 0.191587 0.210727

C 6.042914 0.622007 -1.014044

C 4.720402 0.382211 -1.360143

O -0.273097 -2.677062 -0.413619

C -0.629031 -3.811091 0.396582

C -0.805807 -4.990750 -0.523790

O -2.024055 3.428045 -0.612467

C -1.916765 4.691604 0.013145

H 0.394369 3.107586 0.020419

H 0.236526 2.840729 1.765354

H -1.168184 0.849667 1.354288

H -0.522080 1.446554 -1.549455

H 0.267803 -0.820271 -1.737983

H -2.692047 2.736165 1.218023

H -3.903611 1.871918 -1.042221

H -2.456797 0.312152 -2.208928

H -4.977523 -0.282821 -1.656379

H -5.009263 -1.994346 -1.196487

H -5.975413 -0.814122 -0.289204

H -4.791858 -1.504514 1.913215

H -3.009950 -1.498111 1.944988

H -3.887983 -2.715433 0.989923

H 3.717508 -1.244997 1.439385

H 6.081707 -0.826530 2.046106

H 7.573570 0.374947 0.473908

H 6.689243 1.144591 -1.708388

H 4.328198 0.707021 -2.316091

H -1.548002 -3.572569 0.933784

H 0.163023 -3.980215 1.127452

H 0.117542 -5.203986 -1.063432

H -1.600313 -4.800865 -1.246442

H -1.074304 -5.870373 0.062095

H -1.673097 5.417132 -0.761687

H -1.129391 4.704586 0.772720

H -2.863612 4.973174 0.484863

==============================

**conf007_E1.12**

C -0.265585 -2.337502 0.882961

C 0.954829 -1.498418 0.591247

C 0.825409 -0.747623 -0.732837

C -0.233062 0.353039 -0.737483

C -1.619178 -0.140435 -0.339210

S -1.824433 -1.390203 0.897065

C 2.245105 -2.300954 0.402086

C 3.174419 -1.308662 -0.309061

C 2.255380 -0.311815 -1.069300

O 2.564388 0.946001 -0.488954

O 3.836872 -0.503020 0.648641

C 3.814853 0.832955 0.174412

C 4.954482 1.073552 -0.802074

C 3.834368 1.774424 1.351693

N -2.611786 0.460793 -0.849859

C 0.076090 1.519227 0.196023

O 0.144798 1.438793 1.396314

C -3.936695 0.156219 -0.476695

C -4.751403 -0.564611 -1.347167

C -6.070209 -0.825621 -1.002256

C -6.589819 -0.358472 0.199377

C -5.778768 0.373124 1.058857

C -4.456132 0.631191 0.726589

O 0.183612 2.659579 -0.475377

C 0.531198 3.818759 0.301965

C 0.736205 4.964539 -0.654641

O 1.985726 -3.407887 -0.435010

C 2.991995 -4.397908 -0.375512

H -0.367241 -3.128720 0.143099

H -0.219103 -2.792140 1.873076

H 1.124436 -0.781409 1.397043

H 0.525253 -1.474372 -1.494648

H -0.335567 0.756928 -1.743514

H 2.674401 -2.634334 1.352658

H 3.887061 -1.815310 -0.962517

H 2.440093 -0.286746 -2.144179

H 4.901775 0.370442 -1.635188

H 4.895605 2.088095 -1.196938

H 5.911288 0.945924 -0.294980

H 4.752562 1.632503 1.922009

H 2.973169 1.577789 1.990196

H 3.797755 2.806273 1.002057

H -4.341837 -0.917744 -2.285630

H -6.695678 -1.394130 -1.679405

H -7.620821 -0.558539 0.461374

H -6.176001 0.746002 1.994866

H -3.818231 1.203156 1.390485

H 1.437254 3.591932 0.865625

H -0.273997 4.018767 1.010344

H 0.999866 5.861841 -0.093885

H -0.174000 5.166036 -1.220483

H 1.543904 4.743831 -1.353604

H 3.066712 -4.812998 0.634967

H 3.970583 -4.004305 -0.666818

H 2.710127 -5.188827 -1.068802

==============================

**conf008_E1.32** The lowest energy conformer of 23a

Sum of electronic and thermal Free Energies= -1645.152477 (Hartree/Particle)

C -0.265237 -2.337629 0.883059

C 0.955112 -1.498473 0.591312

C 0.825638 -0.747797 -0.732838

C -0.232796 0.352882 -0.737514

C -1.618901 -0.140583 -0.339184

S -1.824121 -1.390380 0.897070

C 2.245472 -2.300857 0.402240

C 3.174701 -1.308374 -0.308713

C 2.255592 -0.312001 -1.069472

O 2.564459 0.946176 -0.489836

O 3.836378 -0.502274 0.649142

C 3.814526 0.833501 0.174369

C 4.954724 1.073846 -0.801517

C 3.833219 1.775432 1.351301

N -2.611555 0.460597 -0.849788

C 0.076278 1.519155 0.195930

O 0.145469 1.438734 1.396195

C -3.936439 0.155854 -0.476663

C -4.456257 0.631655 0.726122

C -5.778870 0.373405 1.058368

C -6.589508 -0.359181 0.199349

C -6.069512 -0.827161 -1.001800

C -4.750732 -0.566006 -1.346677

O 0.183007 2.659589 -0.475464

C 0.529876 3.819001 0.301847

C 0.733866 4.964961 -0.654765

O 1.986310 -3.407717 -0.435027

C 2.992562 -4.397744 -0.375345

H -0.366845 -3.128902 0.143245

H -0.218752 -2.792188 1.873210

H 1.124635 -0.781397 1.397059

H 0.525390 -1.474607 -1.494554

H -0.335320 0.756730 -1.743560

H 2.674686 -2.634293 1.352829

H 3.887838 -1.814827 -0.961780

H 2.440304 -0.287489 -2.144362

H 4.902599 0.370428 -1.634408

H 4.895970 2.088234 -1.196793

H 5.911236 0.946519 -0.293793

H 3.796799 2.807146 1.001244

H 4.751035 1.633757 1.922286

H 2.971575 1.579013 1.989271

H -3.818672 1.204391 1.389656

H -6.176403 0.746927 1.993992

H -7.620491 -0.559388 0.461312

H -6.694672 -1.396449 -1.678580

H -4.340857 -0.919811 -2.284752

H 1.436187 3.592843 0.865361

H -0.275352 4.018406 1.010361

H 1.541664 4.744924 -1.353823

H 0.996847 5.862468 -0.094021

H -0.176568 5.165718 -1.220502

H 3.971300 -4.004014 -0.665974

H 2.711089 -5.188418 -1.069076

H 3.066737 -4.813199 0.635024

==============================

**conf009_E2.07**

C -0.042210 2.003242 -1.188325

C 1.127501 1.180586 -0.706258

C 0.981916 0.791383 0.764024

C -0.157496 -0.171900 1.087387

C -1.512982 0.270404 0.538332

S -1.651951 1.174951 -0.977340

C 2.469830 1.917917 -0.726587

C 3.350890 1.069321 0.200357

C 2.385795 0.342367 1.178507

O 2.587608 -1.032239 0.894716

O 3.948530 0.020076 -0.539164

C 3.829376 -1.170365 0.222406

C 4.965388 -1.278813 1.225716

C 3.748499 -2.347699 -0.715938

N -2.540244 -0.137001 1.159743

C 0.015132 -1.622045 0.640926

O -0.025920 -2.551925 1.402532

C -3.844011 0.123722 0.691168

C -4.381596 -0.634744 -0.347571

C -5.684836 -0.407728 -0.767386

C -6.458653 0.573301 -0.158810

C -5.921712 1.323054 0.881307

C -4.622115 1.097576 1.313448

O 0.112429 -1.741556 -0.681014

C 0.155605 -3.077807 -1.215763

C -1.239650 -3.643421 -1.347506

O 2.299643 3.211148 -0.186621

C 3.361149 4.088161 -0.503058

H -0.083933 2.952285 -0.657772

H 0.017029 2.206397 -2.258130

H 1.236247 0.280905 -1.314513

H 0.768717 1.711685 1.317905

H -0.281348 -0.227809 2.168067

H 2.897759 1.978474 -1.732512

H 4.103088 1.672944 0.711740

H 2.597675 0.547741 2.228868

H 4.979852 -0.407389 1.882935

H 4.838138 -2.174209 1.834591

H 5.919548 -1.339218 0.701626

H 2.923694 -2.199718 -1.413027

H 3.584103 -3.263695 -0.148130

H 4.680677 -2.443763 -1.272925

H -3.772671 -1.400242 -0.814396

H -6.096146 -1.001123 -1.574674

H -7.474683 0.748260 -0.488534

H -6.518526 2.087263 1.363750

H -4.199456 1.672360 2.128415

H 0.784584 -3.694625 -0.575358

H 0.631821 -2.970782 -2.188454

H -1.844340 -3.015559 -2.003273

H -1.725633 -3.712502 -0.373890

H -1.188314 -4.643847 -1.779803

H 3.422893 4.246252 -1.584766

H 4.324111 3.708627 -0.148090

H 3.154803 5.037984 -0.012260

==============================

**conf010_E2.65**

C 0.155813 1.917588 1.228777

C -1.032992 1.154908 0.697719

C -0.887171 0.834893 -0.789032

C 0.235783 -0.131919 -1.159165

C 1.595081 0.236698 -0.566729

S 1.738927 1.045741 1.001482

C -2.352475 1.932434 0.744293

C -3.250577 1.165393 -0.237023

C -2.298248 0.436439 -1.226515

O -2.542689 -0.938212 -0.976050

O -3.914564 0.116332 0.444004

C -3.811605 -1.052906 -0.352343

C -4.915456 -1.087353 -1.396455

C -3.807918 -2.261364 0.548847

N 2.617444 -0.167045 -1.198529

C 0.015310 -1.601558 -0.810211

O 0.013991 -2.481843 -1.629921

C 3.921606 0.013433 -0.695608

C 4.761085 0.962216 -1.275332

C 6.060522 1.110597 -0.810674

C 6.536768 0.307030 0.218695

C 5.702024 -0.649816 0.783992

C 4.398256 -0.799031 0.332294

O -0.073391 -1.808635 0.499628

C -0.236109 -3.173160 0.923974

C -0.312570 -3.171365 2.428828

O -2.134585 3.248380 0.281565

C -3.172583 4.138677 0.636353

H 0.234673 2.886899 0.741007

H 0.088796 2.076055 2.305678

H -1.173498 0.229372 1.259743

H -0.655850 1.777685 -1.295854

H 0.372166 -0.121377 -2.239692

H -2.790778 1.949976 1.747414

H -3.961310 1.825609 -0.737673

H -2.496211 0.673152 -2.273067

H -4.880050 -0.193644 -2.022145

H -4.797038 -1.964893 -2.032510

H -5.888307 -1.133643 -0.906425

H -4.757332 -2.330135 1.080183

H -2.994851 -2.174076 1.270138

H -3.672409 -3.166296 -0.043678

H 4.385564 1.578499 -2.083035

H 6.705172 1.856155 -1.259568

H 7.552860 0.421661 0.573732

H 6.066027 -1.284825 1.582282

H 3.741665 -1.544630 0.766172

H 0.613681 -3.753786 0.562344

H -1.143499 -3.571037 0.467625

H 0.601946 -2.762772 2.860842

H -0.437876 -4.193412 2.787769

H -1.159384 -2.577007 2.774750

H -2.931912 5.107756 0.202071

H -3.241966 4.237529 1.724641

H -4.142567 3.809464 0.251334

==============================

**conf011_E2.75**

C -1.459921 -2.507655 -1.260689

C -1.830039 -1.159540 -0.689298

C -0.933557 -0.767750 0.484778

C 0.529130 -0.489798 0.123997

C 1.179956 -1.669045 -0.595233

S 0.277982 -2.606592 -1.790534

C -3.232623 -1.098261 -0.077565

C -3.181805 0.159707 0.798722

C -1.688789 0.372097 1.177445

O -1.370852 1.630139 0.603955

O -3.505674 1.296897 0.019871

C -2.584204 2.321965 0.351804

C -3.038461 3.066569 1.596548

C -2.395930 3.222446 -0.842707

N 2.402887 -1.983522 -0.492603

C 0.721310 0.742464 -0.757389

O 0.330272 0.835743 -1.892694

C 3.306635 -1.249637 0.304595

C 4.136704 -0.302050 -0.290090

C 5.073190 0.377443 0.476685

C 5.195763 0.114218 1.835795

C 4.372882 -0.838551 2.424779

C 3.433617 -1.523136 1.665860

O 1.416512 1.680683 -0.124325

C 1.602025 2.928389 -0.816671

C 2.803245 2.874991 -1.731978

O -3.440289 -2.240887 0.725643

C -4.802161 -2.468732 1.024506

H -1.635713 -3.291130 -0.526352

H -2.035160 -2.739051 -2.157943

H -1.768607 -0.389935 -1.461966

H -0.923401 -1.610797 1.183360

H 1.088643 -0.322036 1.043197

H -4.016843 -1.019487 -0.837578

H -3.834786 0.076256 1.669446

H -1.513869 0.392333 2.254173

H -2.295806 3.815635 1.872131

H -3.989363 3.564421 1.404655

H -3.166172 2.376186 2.432285

H -3.344368 3.692130 -1.104116

H -2.033694 2.636371 -1.687431

H -1.673512 4.004204 -0.607735

H 4.036162 -0.102193 -1.350313

H 5.710234 1.117145 0.007258

H 5.929118 0.643951 2.430061

H 4.460985 -1.053159 3.482712

H 2.791846 -2.269412 2.118602

H 1.740460 3.662705 -0.025214

H 0.689502 3.161200 -1.364226

H 3.707580 2.648864 -1.165976

H 2.932772 3.845096 -2.214549

H 2.670556 2.119414 -2.506446

H -4.850298 -3.341997 1.673159

H -5.370989 -2.666166 0.109955

H -5.254760 -1.617829 1.542666

==============================

**conf012_E2.79**

C 0.042296 2.003279 1.188239

C -1.127431 1.180625 0.706210

C -0.981862 0.791405 -0.764065

C 0.157527 -0.171921 -1.087421

C 1.513015 0.270326 -0.538344

S 1.651968 1.174834 0.977402

C -2.469745 1.917978 0.726535

C -3.350835 1.069420 -0.200418

C -2.385736 0.342384 -1.178518

O -2.587555 -1.032205 -0.894604

O -3.948610 0.020224 0.539087

C -3.829384 -1.170272 -0.222392

C -4.965308 -1.278768 -1.225792

C -3.748557 -2.347544 0.716038

N 2.540286 -0.137055 -1.159706

C -0.015162 -1.622068 -0.640980

O 0.025852 -2.551957 -1.402583

C 3.844027 0.123666 -0.691156

C 4.622088 1.097594 -1.313349

C 5.921688 1.323080 -0.881211

C 6.458672 0.573274 0.158827

C 5.684892 -0.407842 0.767324

C 4.381670 -0.634874 0.347512

O -0.112441 -1.741592 0.680951

C -0.155949 -3.077839 1.215657

C 1.239198 -3.643649 1.347663

O -2.299527 3.211222 0.186567

C -3.360923 4.088304 0.503206

H 0.084098 2.952265 0.657586

H -0.016988 2.206570 2.258018

H -1.236166 0.280956 1.314488

H -0.768635 1.711695 -1.317960

H 0.281403 -0.227802 -2.168104

H -2.897654 1.978545 1.732468

H -4.102971 1.673092 -0.711836

H -2.597644 0.547663 -2.228894

H -4.979657 -0.407406 -1.883092

H -4.838080 -2.174225 -1.834582

H -5.919528 -1.339069 -0.701805

H -4.680773 -2.443630 1.272959

H -2.923815 -2.199494 1.413181

H -3.584089 -3.263581 0.148318

H 4.199423 1.672442 -2.128265

H 6.518444 2.087357 -1.363615

H 7.474689 0.748251 0.488571

H 6.096224 -1.001283 1.574563

H 3.772794 -1.400446 0.814271

H -0.784862 -3.694549 0.575074

H -0.632403 -2.970783 2.188233

H 1.725391 -3.712563 0.374136

H 1.187681 -4.644176 1.779695

H 1.843820 -3.016003 2.003693

H -3.154769 5.038003 0.012088

H -3.422241 4.246648 1.584899

H -4.324010 3.708666 0.148694

==============================

**conf013_E2.89**

C -1.623940 -2.695986 -0.870085

C -1.891578 -1.246091 -0.542497

C -0.976285 -0.732293 0.567320

C 0.510434 -0.636787 0.209121

C 1.081265 -1.916019 -0.401539

S 0.093639 -3.003841 -1.374443

C -3.291872 -0.988803 0.026864

C -3.158256 0.375529 0.716111

C -1.649235 0.559906 1.043941

O -1.272443 1.672633 0.248302

O -3.445370 1.409071 -0.208336

C -2.445564 2.404591 -0.066199

C -2.797147 3.357717 1.064566

C -2.250576 3.102169 -1.388458

N 2.312057 -2.220528 -0.373454

C 0.846372 0.494057 -0.759093

O 0.590890 0.483325 -1.936271

C 3.277318 -1.364387 0.198722

C 4.032458 -0.540864 -0.635279

C 5.013015 0.280079 -0.096885

C 5.257444 0.279590 1.271617

C 4.515282 -0.554836 2.098915

C 3.531027 -1.378546 1.569006

O 1.507590 1.467168 -0.147203

C 1.837934 2.610973 -0.949079

C 2.481527 3.629313 -0.044101

O -3.595400 -1.987670 0.977632

C -4.971794 -2.058435 1.287748

H -1.848513 -3.325525 -0.011844

H -2.223590 -3.036795 -1.715079

H -1.767414 -0.624199 -1.433220

H -1.046449 -1.443364 1.396953

H 1.068762 -0.447089 1.125175

H -4.059842 -0.961479 -0.753029

H -3.795704 0.446147 1.599150

H -1.456600 0.765043 2.098162

H -3.719004 3.889741 0.828045

H -2.937601 2.811317 1.998886

H -1.994726 4.083338 1.200670

H -1.469744 3.857489 -1.298694

H -3.177084 3.593497 -1.686046

H -1.964994 2.374477 -2.148075

H 3.836125 -0.545856 -1.701134

H 5.588329 0.923545 -0.751421

H 6.023844 0.919864 1.689159

H 4.701803 -0.566999 3.165720

H 2.949754 -2.030898 2.209617

H 0.919811 2.989432 -1.401470

H 2.510464 2.293525 -1.748352

H 3.386944 3.225252 0.410909

H 1.793989 3.929571 0.747740

H 2.750049 4.513175 -0.623474

H -5.349880 -1.113881 1.690856

H -5.094950 -2.836111 2.039901

H -5.555509 -2.319555 0.398868

Optimized Cartesian Coordinates of the studied conformers of 25 at m06-2x/def2-TZVP/SMD/ACN level of theory.

==============================

**conf000_E0.00**

C 0.606510 -2.133864 -1.005206

C 1.475895 -0.910057 -1.164117

C 1.098980 0.198676 -0.184036

C -0.252606 0.809410 -0.518205

C -1.387382 -0.198033 -0.435234

S -1.171785 -1.781136 -1.222888

C 2.963767 -1.135082 -0.890717

C 3.498820 0.231836 -0.379152

C 2.280695 1.164577 -0.266671

N -2.476790 0.152742 0.103417

C -0.571802 1.986670 0.383198

O -0.277923 2.037019 1.550140

C -3.611035 -0.679678 0.145785

C -4.065675 -1.143789 1.378750

C -5.206912 -1.930228 1.442592

C -5.914909 -2.239069 0.286590

C -5.471474 -1.756642 -0.938740

C -4.323189 -0.980293 -1.014071

O -1.195386 2.956810 -0.275692

C -1.657214 4.080884 0.499025

C -2.947659 3.747818 1.211512

O 2.134787 1.953594 -1.427811

O 3.149941 -2.000891 0.218313

O 4.005458 -0.052329 0.913243

C 2.936940 3.116685 -1.409121

C 4.190521 -1.456937 1.013303

C 5.550691 -1.854185 0.462877

C 3.986773 -1.879663 2.446111

H 0.742388 -2.574220 -0.019296

H 0.825013 -2.891013 -1.758861

H 1.382980 -0.521282 -2.182503

H 1.075503 -0.214613 0.829371

H 3.498128 -1.512099 -1.764225

H 4.271031 0.656637 -1.023418

H 2.364405 1.803397 0.617991

H -3.515019 -0.887693 2.275626

H -5.547987 -2.298012 2.402401

H -6.809477 -2.845997 0.341346

H -6.020542 -1.985711 -1.843704

H -3.976727 -0.595797 -1.966171

H -1.799549 4.874776 -0.231115

H -0.873984 4.374294 1.196874

H -3.711663 3.442562 0.495427

H -3.305161 4.630526 1.743857

H -2.799675 2.944394 1.933139

H 2.643816 3.773525 -0.583176

H 3.999501 2.875455 -1.307036

H 2.781014 3.634797 -2.353927

H 6.339794 -1.395728 1.059325

H 5.664189 -2.937988 0.498884

H 5.658338 -1.524116 -0.571772

H 4.076210 -2.962795 2.529465

H 4.743223 -1.416911 3.079937

H 2.996733 -1.571531 2.782123

H -0.229830 1.166285 -1.550663

==============================

**conf001_E0.47**

C 0.283790 -2.219523 -0.980187

C 1.242019 -1.068545 -1.167159

C 0.983506 0.064916 -0.177481

C -0.326439 0.784174 -0.460341

C -1.538764 -0.127210 -0.369279

S -1.465218 -1.726185 -1.148009

C 2.715086 -1.411344 -0.934250

C 3.369931 -0.092967 -0.434413

C 2.234237 0.935187 -0.299771

N -2.597067 0.317267 0.162484

C -0.511879 1.961468 0.479059

O -0.239306 1.930082 1.651886

C -3.793669 -0.424228 0.206191

C -4.544168 -0.638292 -0.948527

C -5.748598 -1.324425 -0.873098

C -6.210381 -1.802290 0.347264

C -5.463062 -1.579848 1.498300

C -4.263972 -0.884693 1.434476

O -0.989932 3.028296 -0.151059

C -1.195716 4.216849 0.638241

C 0.094547 4.986733 0.797877

O 2.121573 1.722744 -1.465362

O 2.862034 -2.294274 0.167224

O 3.875086 -0.417961 0.848678

C 3.024579 2.809344 -1.488714

C 3.957043 -1.832672 0.941297

C 5.271823 -2.327527 0.360736

C 3.752478 -2.244913 2.377002

H 0.413152 -2.669669 0.002162

H 0.419022 -2.991465 -1.738487

H 1.151182 -0.673677 -2.183258

H 0.962522 -0.347460 0.836427

H 3.194709 -1.825500 -1.822559

H 4.162299 0.266701 -1.093558

H 2.391775 1.573802 0.575844

H -4.181985 -0.258734 -1.896731

H -6.327076 -1.486604 -1.774189

H -7.148931 -2.338635 0.402450

H -5.817371 -1.944885 2.454370

H -3.681116 -0.696719 2.327815

H -1.617891 3.931100 1.600764

H -1.933349 4.790296 0.080769

H 0.499803 5.259068 -0.177406

H 0.835438 4.399019 1.340212

H -0.097164 5.902686 1.358815

H 2.831295 3.489078 -0.652299

H 4.065054 2.474410 -1.436834

H 2.870156 3.339923 -2.426896

H 6.105102 -1.935392 0.944285

H 5.303075 -3.417106 0.387085

H 5.383739 -1.997700 -0.673537

H 3.763732 -3.332014 2.455954

H 4.553832 -1.841312 2.995867

H 2.794791 -1.866420 2.734253

H -0.302826 1.163042 -1.484841

==============================

**conf002_E0.83**

C 0.606549 -2.134073 -1.004820

C 1.475918 -0.910295 -1.163999

C 1.099003 0.198597 -0.184097

C -0.252600 0.809284 -0.518267

C -1.387364 -0.198181 -0.435293

S -1.171715 -1.781341 -1.222866

C 2.963801 -1.135199 -0.890630

C 3.498853 0.231794 -0.379420

C 2.280693 1.164523 -0.266886

N -2.476783 0.152624 0.103304

C -0.571809 1.986513 0.383198

O -0.278056 2.036750 1.550174

C -3.611098 -0.679680 0.145719

C -4.323107 -0.980587 -1.014143

C -5.471520 -1.756760 -0.938732

C -5.915201 -2.238726 0.286682

C -5.207338 -1.929591 1.442701

C -4.066008 -1.143310 1.378778

O -1.195289 2.956725 -0.275686

C -1.657155 4.080777 0.499040

C -2.947680 3.747691 1.211383

O 2.134656 1.953519 -1.428013

O 3.150043 -2.000768 0.218637

O 4.005775 -0.052087 0.912968

C 2.936749 3.116655 -1.409407

C 4.190719 -1.456659 1.013404

C 5.550849 -1.854117 0.463024

C 3.987042 -1.879070 2.446326

H 0.742287 -2.574115 -0.018752

H 0.825113 -2.891471 -1.758207

H 1.382942 -0.521714 -2.182455

H 1.075548 -0.214532 0.829374

H 3.498135 -1.512470 -1.764042

H 4.270924 0.656542 -1.023882

H 2.364474 1.803337 0.617771

H -3.976476 -0.596437 -1.966323

H -6.020487 -1.986032 -1.843705

H -6.809857 -2.845520 0.341508

H -5.548622 -2.297013 2.402574

H -3.515472 -0.886948 2.275651

H -1.799401 4.874713 -0.231067

H -0.873997 4.374127 1.196990

H -2.799758 2.944247 1.933004

H -3.711597 3.442438 0.495204

H -3.305240 4.630386 1.743709

H 2.643817 3.773375 -0.583299

H 3.999359 2.875488 -1.307657

H 2.780520 3.634874 -2.354103

H 6.339956 -1.395371 1.059250

H 5.664388 -2.937902 0.499468

H 5.658429 -1.524469 -0.571767

H 4.743798 -1.416600 3.079993

H 2.997189 -1.570474 2.782465

H 4.075997 -2.962239 2.529732

H -0.229855 1.166226 -1.550700

==============================

**conf003_E1.06**

C 0.540009 -2.091350 -1.145738

C 1.407929 -0.857880 -1.209963

C 1.056858 0.155792 -0.122843

C -0.300179 0.796034 -0.370269

C -1.434317 -0.213430 -0.343886

S -1.244001 -1.721418 -1.272179

C 2.901727 -1.107384 -0.994470

C 3.451275 0.205362 -0.368714

C 2.238854 1.124477 -0.144035

N -2.511914 0.086633 0.246322

C -0.587063 1.898499 0.630388

O -0.277756 1.850415 1.793543

C -3.636232 -0.762549 0.238566

C -3.999276 -1.427378 1.407927

C -5.127292 -2.235468 1.420168

C -5.909997 -2.369976 0.279106

C -5.555456 -1.690169 -0.879795

C -4.422324 -0.888551 -0.905104

O -1.204124 2.930371 0.068336

C -1.568529 4.013421 0.944996

C -2.268415 5.055928 0.112687

O 2.068112 2.016049 -1.224722

O 3.112900 -2.074372 0.022689

O 3.982486 -0.198884 0.881121

C 2.866155 3.176879 -1.114659

C 4.170525 -1.606037 0.842977

C 5.518468 -1.945140 0.227649

C 3.997721 -2.164084 2.232888

H 0.704423 -2.626001 -0.212197

H 0.733782 -2.770682 -1.976451

H 1.291628 -0.375536 -2.184980

H 1.054233 -0.348781 0.848635

H 3.414674 -1.399616 -1.912209

H 4.211644 0.687549 -0.986225

H 2.344385 1.679996 0.792901

H -3.389188 -1.309378 2.295028

H -5.397892 -2.759135 2.328719

H -6.793063 -2.995752 0.294937

H -6.162273 -1.783641 -1.771934

H -4.142359 -0.352570 -1.804403

H -0.662307 4.404714 1.408804

H -2.214546 3.618415 1.730248

H -2.558599 5.892180 0.749426

H -3.166792 4.643939 -0.348049

H -1.610220 5.431196 -0.671707

H 2.690170 3.778251 -2.005095

H 2.586132 3.753865 -0.226796

H 3.931342 2.933084 -1.054677

H 6.320109 -1.544517 0.848557

H 5.633077 -3.027082 0.156466

H 5.603445 -1.516439 -0.772407

H 4.088270 -3.250080 2.210185

H 4.767667 -1.762862 2.891857

H 3.015129 -1.890967 2.617536

H -0.301037 1.235522 -1.370556

==============================

**conf004_E1.24**

C 0.283869 -2.219482 -0.980359

C 1.242024 -1.068415 -1.167173

C 0.983473 0.064890 -0.177325

C -0.326469 0.784144 -0.460221

C -1.538760 -0.127267 -0.369169

S -1.465197 -1.726232 -1.147864

C 2.715110 -1.411186 -0.934380

C 3.369908 -0.092864 -0.434340

C 2.234193 0.935218 -0.299426

N -2.597087 0.317206 0.162570

C -0.511937 1.961480 0.479102

O -0.239136 1.930256 1.651886

C -3.793674 -0.424309 0.206233

C -4.264013 -0.884806 1.434491

C -5.463097 -1.579975 1.498258

C -6.210379 -1.802405 0.347196

C -5.748570 -1.324492 -0.873139

C -4.544149 -0.638339 -0.948511

O -0.990247 3.028171 -0.151046

C -1.195877 4.216836 0.638108

C 0.094401 4.986812 0.797159

O 2.121451 1.723134 -1.464761

O 2.862151 -2.294304 0.166937

O 3.875108 -0.418052 0.848686

C 3.024624 2.809600 -1.487963

C 3.957147 -1.832782 0.941058

C 5.271953 -2.327437 0.360381

C 3.752650 -2.245270 2.376705

H 0.413356 -2.669830 0.001881

H 0.419075 -2.991244 -1.738846

H 1.151141 -0.673386 -2.183207

H 0.962429 -0.347617 0.836525

H 3.194718 -1.825168 -1.822777

H 4.162242 0.266947 -1.093447

H 2.391734 1.573561 0.576388

H -3.681193 -0.696847 2.327857

H -5.817432 -1.945032 2.454313

H -7.148923 -2.338761 0.402344

H -6.327021 -1.486649 -1.774251

H -4.181954 -0.258721 -1.896686

H -1.617723 3.931242 1.600822

H -1.933726 4.790130 0.080764

H -0.097173 5.902950 1.357837

H 0.499384 5.258814 -0.178333

H 0.835450 4.399285 1.339483

H 2.870224 3.340379 -2.426034

H 2.831517 3.489194 -0.651396

H 4.065047 2.474489 -1.436223

H 6.105215 -1.935356 0.943989

H 5.303274 -3.417018 0.386532

H 5.383834 -1.997414 -0.673833

H 2.794960 -1.866881 2.734058

H 3.763953 -3.332384 2.455469

H 4.554007 -1.841740 2.995611

H -0.302826 1.162989 -1.484727

==============================

**conf005_E1.27**

C 0.437126 -2.263013 -0.623978

C 1.373465 -1.161200 -1.057153

C 1.064013 0.165037 -0.366852

C -0.256752 0.763967 -0.829211

C -1.450931 -0.137074 -0.566485

S -1.318815 -1.872029 -0.932217

C 2.847446 -1.397288 -0.712993

C 3.456145 0.019726 -0.510422

C 2.296331 1.014728 -0.673829

N -2.542099 0.392153 -0.206401

C -0.517142 2.131574 -0.225875

O -0.809201 3.106518 -0.868196

C -3.722452 -0.356812 -0.031123

C -4.462757 -0.780913 -1.133008

C -5.653316 -1.468897 -0.942147

C -6.111798 -1.738791 0.341510

C -5.373069 -1.309376 1.438130

C -4.186688 -0.612687 1.257536

O -0.356337 2.124165 1.094673

C -0.673437 3.338834 1.801623

C -2.164652 3.460279 2.012991

O 2.212811 1.463507 -2.008898

O 2.977533 -2.001422 0.564869

O 3.903788 0.018622 0.833202

C 3.059824 2.561941 -2.277982

C 4.023573 -1.333154 1.251153

C 5.376052 -1.897136 0.847234

C 3.768141 -1.414002 2.734815

H 0.553274 -2.468390 0.438293

H 0.608782 -3.186056 -1.178750

H 1.302470 -1.018797 -2.139103

H 1.035176 -0.003298 0.714219

H 3.365234 -1.988578 -1.469785

H 4.272560 0.237985 -1.201458

H 2.408194 1.864206 0.009072

H -4.102019 -0.563323 -2.131379

H -6.224092 -1.795116 -1.802792

H -7.040487 -2.275511 0.486666

H -5.723636 -1.513321 2.442308

H -3.608902 -0.268624 2.106603

H -0.273326 4.185385 1.245229

H -0.143208 3.251242 2.747666

H -2.690235 3.522160 1.059964

H -2.377140 4.364483 2.585378

H -2.542910 2.602269 2.570040

H 4.108188 2.327889 -2.067897

H 2.956018 2.800968 -3.335108

H 2.765606 3.430852 -1.679931

H 5.521928 -1.812008 -0.231029

H 6.171871 -1.348902 1.351960

H 5.438229 -2.949019 1.127301

H 3.804427 -2.453279 3.061324

H 4.531171 -0.851841 3.273199

H 2.786277 -0.997968 2.960608

H -0.211216 0.908412 -1.911127

==============================

**conf006_E1.34**

C 0.437106 -2.263256 -0.623850

C 1.373481 -1.161450 -1.056992

C 1.064011 0.164839 -0.366800

C -0.256779 0.763731 -0.829238

C -1.450904 -0.137219 -0.566290

S -1.318856 -1.872250 -0.932059

C 2.847446 -1.397485 -0.712794

C 3.456145 0.019602 -0.510459

C 2.296291 1.014541 -0.673912

N -2.542037 0.391931 -0.206006

C -0.517057 2.131507 -0.226277

O -0.809139 3.106278 -0.868824

C -3.722432 -0.356935 -0.030748

C -4.187413 -0.611826 1.257818

C -5.373974 -1.308309 1.438252

C -6.112153 -1.738412 0.341553

C -5.652880 -1.469563 -0.942078

C -4.462149 -0.781875 -1.132761

O -0.356023 2.124482 1.094282

C -0.673053 3.339298 1.800939

C -2.164252 3.460941 2.012344

O 2.212650 1.463239 -2.008961

O 2.977493 -2.001320 0.565224

O 3.903822 0.018689 0.833151

C 3.059542 2.561757 -2.278117

C 4.023557 -1.333058 1.251315

C 5.376010 -1.897122 0.847401

C 3.768283 -1.413576 2.735011

H 0.553263 -2.468649 0.438415

H 0.608738 -3.186274 -1.178665

H 1.302545 -1.019097 -2.138952

H 1.035118 -0.003383 0.714276

H 3.365255 -1.988945 -1.469420

H 4.272516 0.237779 -1.201567

H 2.408185 1.864041 0.008958

H -3.610106 -0.267173 2.106978

H -5.725146 -1.511437 2.442392

H -7.040997 -2.274904 0.486584

H -6.223225 -1.796379 -1.802787

H -4.100745 -0.565118 -2.131080

H -0.272860 4.185724 1.244426

H -0.142861 3.251889 2.747013

H -2.689917 3.522855 1.059362

H -2.376554 4.365207 2.584721

H -2.542589 2.603016 2.569479

H 2.764988 3.430765 -1.680375

H 4.107881 2.327926 -2.067674

H 2.956037 2.800507 -3.335338

H 5.438184 -2.948966 1.127648

H 5.521802 -1.812193 -0.230897

H 6.171879 -1.348819 1.351980

H 2.786400 -0.997575 2.960833

H 3.804706 -2.452762 3.061816

H 4.531322 -0.851184 3.273155

H -0.211249 0.907913 -1.911182

==============================

**conf007_E1.47**

C 0.116689 -2.316087 -0.587497

C 1.118538 -1.294215 -1.067315

C 0.929778 0.058960 -0.386778

C -0.358112 0.751772 -0.808926

C -1.607838 -0.054920 -0.504068

S -1.613150 -1.798730 -0.854003

C 2.581468 -1.633226 -0.764333

C 3.298410 -0.261822 -0.605224

C 2.208975 0.812961 -0.746208

N -2.647174 0.557336 -0.122622

C -0.476952 2.138701 -0.203818

O -0.696059 3.136133 -0.840701

C -3.874656 -0.100466 0.090507

C -4.651067 -0.525184 -0.986082

C -5.881865 -1.124868 -0.756484

C -6.344641 -1.306393 0.541129

C -5.570610 -0.874603 1.612237

C -4.344160 -0.264102 1.392195

O -0.280209 2.117691 1.111768

C -0.328102 3.378802 1.806568

C 0.997378 4.097336 1.708927

O 2.113798 1.250605 -2.083956

O 2.707735 -2.225282 0.519558

O 3.790048 -0.276266 0.722773

C 3.029817 2.280550 -2.394699

C 3.821983 -1.626596 1.161153

C 5.115383 -2.296299 0.726311

C 3.608964 -1.664375 2.653239

H 0.248728 -2.512007 0.474738

H 0.201831 -3.258538 -1.129492

H 1.024181 -1.161431 -2.148713

H 0.926788 -0.095046 0.696703

H 3.030029 -2.272753 -1.526008

H 4.104405 -0.113712 -1.326428

H 2.405190 1.660485 -0.079492

H -4.286481 -0.377402 -1.995797

H -6.480415 -1.452426 -1.597529

H -7.304021 -1.775905 0.716664

H -5.924983 -1.008612 2.626806

H -3.739250 0.084471 2.220354

H -0.560996 3.115143 2.836156

H -1.142542 3.975139 1.397583

H 1.232140 4.345205 0.673607

H 1.798288 3.479837 2.117857

H 0.950491 5.024000 2.282911

H 2.816862 3.176927 -1.802646

H 4.063938 1.973373 -2.210021

H 2.912845 2.511858 -3.452148

H 5.965292 -1.800813 1.196189

H 5.108610 -3.345221 1.023723

H 5.232771 -2.239571 -0.357308

H 2.669169 -1.171278 2.901851

H 3.577214 -2.698040 2.997554

H 4.429117 -1.153188 3.157319

H -0.337370 0.889240 -1.892576

==============================

**conf008_E1.48**

C 0.116736 -2.316018 -0.587800

C 1.118621 -1.294129 -1.067495

C 0.929808 0.058988 -0.386861

C -0.358061 0.751801 -0.809016

C -1.607796 -0.054940 -0.504209

S -1.613066 -1.798675 -0.854502

C 2.581556 -1.633172 -0.764444

C 3.298431 -0.261791 -0.605021

C 2.209038 0.813006 -0.746130

N -2.647072 0.557295 -0.122537

C -0.476961 2.138698 -0.203816

O -0.696251 3.136138 -0.840626

C -3.874588 -0.100504 0.090578

C -4.344119 -0.264005 1.392268

C -5.570585 -0.874456 1.612334

C -6.344601 -1.306316 0.541243

C -5.881815 -1.124890 -0.756370

C -4.650994 -0.525252 -0.985992

O -0.280045 2.117634 1.111727

C -0.328019 3.378719 1.806610

C 0.997378 4.097376 1.708837

O 2.114008 1.250666 -2.083919

O 2.707780 -2.225559 0.519276

O 3.789655 -0.276340 0.723137

C 3.030023 2.280662 -2.394544

C 3.821749 -1.626688 1.161323

C 5.115363 -2.296119 0.726727

C 3.608243 -1.664772 2.653340

H 0.248664 -2.511964 0.474446

H 0.201948 -3.258468 -1.129791

H 1.024328 -1.161262 -2.148886

H 0.926778 -0.095116 0.696609

H 3.030159 -2.272495 -1.526277

H 4.104663 -0.113628 -1.325947

H 2.405183 1.660543 -0.079415

H -3.739215 0.084609 2.220409

H -5.924981 -1.008376 2.626904

H -7.303992 -1.775795 0.716798

H -6.480366 -1.452475 -1.597401

H -4.286418 -0.377523 -1.995714

H -0.560784 3.114966 2.836205

H -1.142569 3.974970 1.397714

H 0.950520 5.023994 2.282889

H 1.231970 4.345322 0.673498

H 1.798394 3.479909 2.117608

H 2.816986 3.177012 -1.802483

H 4.064135 1.973513 -2.209802

H 2.913110 2.511998 -3.451994

H 5.108721 -3.345053 1.024080

H 5.232937 -2.239319 -0.356867

H 5.965088 -1.800500 1.196794

H 3.576417 -2.698513 2.997410

H 4.428203 -1.153671 3.157820

H 2.668344 -1.171769 2.901719

H -0.337286 0.889320 -1.892664

==============================

**conf009_E1.59**

C -0.606458 -2.134141 1.004493

C -1.475875 -0.910424 1.163897

C -1.099019 0.198602 0.184111

C 0.252618 0.809266 0.518235

C 1.387415 -0.198188 0.435239

S 1.171768 -1.781409 1.222662

C -2.963744 -1.135347 0.890444

C -3.498858 0.231713 0.379385

C -2.280719 1.164482 0.267123

N 2.476880 0.152759 -0.103187

C 0.571776 1.986447 -0.383284

O 0.278012 2.036595 -1.550260

C 3.611214 -0.679519 -0.145588

C 4.323076 -0.980652 1.014290

C 5.471459 -1.756876 0.938876

C 5.915268 -2.238632 -0.286561

C 5.207559 -1.929252 -1.442613

C 4.066249 -1.142964 -1.378678

O 1.195182 2.956751 0.275543

C 1.656862 4.080846 -0.499270

C 2.947491 3.747971 -1.211517

O -2.134789 1.953119 1.428525

O -3.149895 -2.000731 -0.218963

O -4.005674 -0.051997 -0.913046

C -2.936973 3.116191 1.410276

C -4.190667 -1.456595 -1.013549

C -5.550726 -1.854033 -0.462988

C -3.987178 -1.878849 -2.446536

H -0.742161 -2.573986 0.018333

H -0.825030 -2.891711 1.757708

H -1.382880 -0.521985 2.182402

H -1.075661 -0.214435 -0.829402

H -3.498119 -1.512748 1.763775

H -4.270971 0.656325 1.023893

H -2.364480 1.803597 -0.617326

H 3.976359 -0.596658 1.966500

H 6.020283 -1.986350 1.843883

H 6.809906 -2.845447 -0.341398

H 5.548951 -2.296507 -2.402511

H 3.515810 -0.886424 -2.275559

H 1.798903 4.874867 0.230786

H 0.873662 4.373957 -1.197276

H 3.304913 4.630691 -1.743894

H 2.799778 2.944443 -1.933082

H 3.711431 3.442940 -0.495270

H -2.780700 3.634180 2.355090

H -2.644177 3.773151 0.584310

H -3.999572 2.874964 1.308555

H -5.658198 -1.524228 0.571763

H -6.339934 -1.395422 -1.059176

H -5.664227 -2.937828 -0.499261

H -4.743815 -1.416034 -3.080091

H -2.997248 -1.570490 -2.782661

H -4.076472 -2.961972 -2.530163

H 0.229891 1.166156 1.550684

==============================

**conf010_E1.66**

C 0.437052 -2.263226 -0.623694

C 1.373421 -1.161460 -1.056947

C 1.064005 0.164869 -0.366841

C -0.256789 0.763752 -0.829215

C -1.450934 -0.137202 -0.566257

S -1.318886 -1.872247 -0.931993

C 2.847394 -1.397507 -0.712766

C 3.456132 0.019564 -0.510529

C 2.296290 1.014535 -0.674026

N -2.542092 0.391956 -0.206121

C -0.517047 2.131502 -0.226190

O -0.809242 3.106278 -0.868681

C -3.722479 -0.356884 -0.030845

C -4.462389 -0.781559 -1.132821

C -5.653123 -1.469232 -0.942089

C -6.112212 -1.738320 0.341552

C -5.373825 -1.308499 1.438224

C -4.187255 -0.612057 1.257747

O -0.355928 2.124429 1.094356

C -0.672882 3.339239 1.801065

C -2.164063 3.460902 2.012584

O 2.212659 1.463122 -2.009101

O 2.977455 -2.001288 0.565271

O 3.903892 0.018708 0.833049

C 3.059662 2.561510 -2.278402

C 4.023572 -1.333010 1.251303

C 5.375981 -1.897178 0.847396

C 3.768286 -1.413420 2.735002

H 0.553194 -2.468511 0.438595

H 0.608720 -3.186304 -1.178403

H 1.302458 -1.019195 -2.138918

H 1.035205 -0.003274 0.714256

H 3.365153 -1.989022 -1.469387

H 4.272464 0.237696 -1.201698

H 2.408181 1.864086 0.008784

H -4.101140 -0.564618 -2.131156

H -6.223608 -1.795848 -1.802779

H -7.041066 -2.274785 0.486614

H -5.724833 -1.511832 2.442380

H -3.609771 -0.267649 2.106885

H -0.272720 4.185668 1.244531

H -0.142604 3.251796 2.747089

H -2.689789 3.522694 1.059627

H -2.376336 4.365242 2.584852

H -2.542363 2.603050 2.569853

H 2.765600 3.430462 -1.680333

H 4.108060 2.327427 -2.068499

H 2.955686 2.800504 -3.335518

H 5.438104 -2.949014 1.127678
[truncated: 302,867 more chars]
